# 1000 fold Ultra‐Photosensitized Fluorescent Protein Mimics Toward Photocatalytic Proximity Labeling and Proteomic Profiling Functions

**DOI:** 10.1002/advs.202413063

**Published:** 2025-02-22

**Authors:** Rui Sun, Yanan Huang, Huan Feng, Nan Zhao, Wang Wan, Di Shen, Bowen Zhong, Yukui Zhang, Xin Zhang, Qun Zhao, Lihua Zhang, Yu Liu

**Affiliations:** ^1^ State Key Laboratory of Medical Proteomics National Chromatographic R. & A. Center CAS Key Laboratory of Separation Science for Analytical Chemistry Dalian Institute of Chemical Physics Chinese Academy of Sciences 457 Zhongshan Road Dalian 116023 China; ^2^ University of Chinese Academy of Sciences Beijing 100049 China; ^3^ Department of Chemistry and Westlake Laboratory of Life Science and Biomedicine Westlake University 600 Dunyu Road Hangzhou 310030 China

**Keywords:** chromophores, fluorescent proteins, phase separation, photosensitizers, proximity labeling

## Abstract

Photosensitizing fluorescent proteins (FP) (e.g. KillerRed) have been shown not capable of photo‐catalytic protein proximity labeling for downstream proteomic profiling applications. To acquire such a function, FP chromophores are engineered in a 12 × 12 combinatorial matrix of synthetic analoges, achieving up to 1000 fold enhancement of reactive oxygen species (ROS) production compared to the natural FPs. FP chromophores are shown with larger dipole moments exhibit higher ROS yield toward protein labeling. By conjugating the ultra‐photosensitized FP chromophore to HaloTag (namely upsFP tag), its photo‐catalytic protein proximity labeling function is demonstrated using nucleophilic amino substrates. Through photochemical characterizations, theoretical calculation, and tandem mass spectrometry, a radical‐mediated labeling mechanism is revealed with expanded reactivity toward diverse protein residues via a type I photosensitization pathway. Finally, a proteomic profiling application is showcased using the upsFP tag to resolve the dynamic interactome variations upon TAR DNA‐binding protein 43 (TDP43) phase separation and suborganellar translocation. Together, this work demonstrates three orders of magnitude ultra‐photosensitization of fluorescent protein chromophore enables photocatalytic protein proximity labeling and profiling functions that are impractical for natural fluorescent proteins.

## Graphical abstract

While natural fluorescent proteins are widely used for imaging, in this work, an ultra‐photosensitized fluorescent protein mimic (upsFP) tag is developed to enable its protein proximity labeling (PPL) function and demonstrate its proteomic applications.

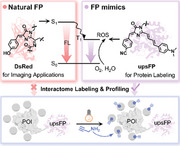

## 1 Introduction

Fluorescent proteins (FPs) have been widely applied for biological imaging for decades.^[^ ^1^ ^]^ Tsien et al., pioneered in evolving green fluorescent protein (GFP) family harboring various functions spanning the entire visible spectrum.^[^ ^2^ ^]^ Toward super‐resolution imaging techniques, photoactivatable FPs (e.g. PA‐GFP), photoshiftable FPs (e.g. Eos), and photoswitching FPs (e.g. Dronpa) were serially developed.^[^ ^3^ ^]^ To snapshot endogenous ions (Ca^2+^, Zn^2+^) and metabolites (NAD^+^/NADH), FPs are endowed with analyte‐induced conformational and photophysical changes.^[^ ^4^ ^]^ Beyond singlet excited state fluorescence properties, photosensitized FPs (e.g. KillerRed^[^ ^5^ ^]^ and SuperNova^[^ ^6^ ^]^) have been leveraged for chromophore‐assisted light inactivation (CALI) and photodynamic therapy (PDT) applications via triplet excited state chemistries.^[^ ^7^ ^]^

Synthetic FP chromophores have transcended the scaffold limit of naturally evolved FPs and expanded their application scope. Jaffrey and others first transformed them for RNA imaging in the cells.^[^ ^8^ ^]^ Subsequently, these synthetic FP chromophores also demonstrated their vital roles in visualizing DNA,^[^ ^9^ ^]^ lipid droplets,^[^ ^10^ ^]^ and aggregated proteins.^[^ ^11^ ^]^ Besides serving as biosensors and latent fingerprint stains,^[^ ^12^ ^]^ they were also widely used to construct functional materials, such as metal‐organic frameworks (MOFs),^[^ ^13^ ^]^ nanoparticles,^[^ ^14^ ^]^ and polymers.^[^ ^15^ ^]^

**Figure 1:**
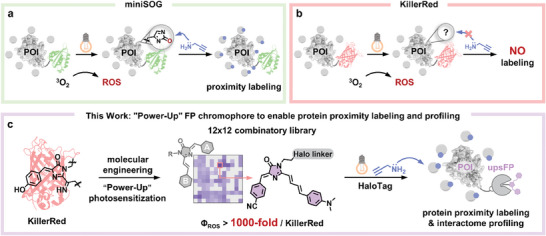
Power‐up photosensitization of fluorescent protein (FP) chromophore to enable protein proximity labeling (PPL) function and downstream proteomic profiling application. a) Photosensitized miniSOG enzyme can perform PPL function via photo‐oxidation of histidine and nucleophilic modification by amino alkyne substrates. These modifications facilitate downstream protein enrichment and proteomic identification. b) Photosensitized fluorescent proteins (e.g. KillerRed) can produce ROS but are commonly known as not capable of PPL. c) This work highlights an ultra‐photosensitized fluorescent protein mimic (upsFP) with three orders of magnitude (>1000 fold) enhancement in ROS yield, enabling photocatalytic proximity labeling function and proteomic profiling application.

Protein proximity labeling (PPL) technologies have emerged as a powerful approach to in situ map and profile protein interactions. Ting and others developed enzyme‐catalyzed PPL methods using peroxidases (HRP or APEX) and biotin ligases (BioID or TurboID).^[^ ^16^ ^]^ In recent years, photo‐catalyzed PPL strategy uses a gallery of photosensitizers (e.g. DBF, bipyridyl‐Ru/Ir complexes) and photosensitized proteins (e.g. miniSOG) to achieve improved spatiotemporal resolution (**Figure** **1**a).^[^ ^17^ ^]^ Although photosensitized FPs (e.g. KillerRed) ubiquitously photo‐catalyze ROS production, growing evidence reaches a consensus that they are not capable of performing photo‐catalytic PPL and downstream proteomic profiling applications (Figure 1b).^[^ ^17^, ^18^ ^]^

Herein, we created an ultra‐photosensitized fluorescent protein mimic (upsFP) tag to enable its PPL function and demonstrate its proteomic application (Figure 1c). To this end, we first constructed a 12 × 12 combinatory library of synthetic FP chromophores to improve their ROS production. Among these probes, we observed a general pattern that FP chromophores with larger dipole moments showed higher ROS production efficiency (Φ_ROS_). The optimal probe (A7B3, i.e. P0) exhibited a more than 1000 fold increase in Φ_ROS_ as compared to that of the natural KillerRed FP. When conjugated to HaloTag, the ultra‐photosensitized FP mimics (namely upsFP) now enabled photo‐catalytic protein proximity labeling by using nucleophilic amino substrates. Mechanistic studies, including photochemical characterizations, electron paramagnetic resonance spectroscopy, and theoretical calculation, collectively revealed that the upsFP tag catalyzed radical‐based labeling via the type I photosensitization pathway. Tandem mass spectrometry pinpointed modification sites and labeling mechanisms, highlighting its expanded reactivity toward diverse protein residues. Finally, we exemplified a proteomic profiling application using upsFP to resolve the dynamic changes of TAR DNA‐binding protein 43 (TDP43) interactome upon its phase separation and suborganellar translocation.

## 2 Results and Discussion

As evidenced by previous literature,^[^ ^17^, ^18^ ^]^ photosensitized fluorescent proteins, such as KillerRed, cannot perform the protein proximity labeling (PPL) function. We reasoned that KillerRed failed to catalyze PPL likely due to its relatively low yield of ROS production (Φ_ROS_), although some also suggested the lack of specific ROS type (e.g. ^1^O_2_). Instead of randomly evolving FPs for such a function, we rationally designed an artificial FP mimic, consisting of an ultra‐photosensitized FP chromophore in conjugation with HaloTag, to enable its PPL function (Figure 1c).

### 2.1 Maximize FP Chromophores’ Photosensitization by a 12 × 12 Combinatorial Matrix

**Figure 2:**
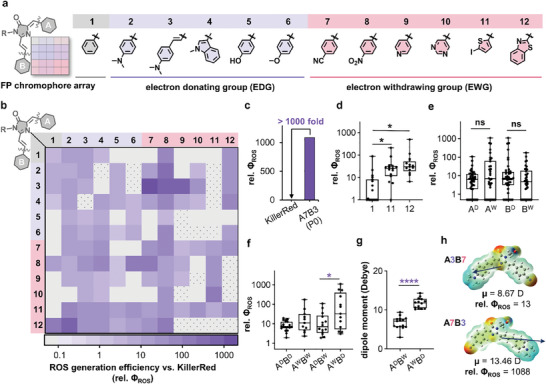
Construction of FP chromophore combinatorial array to maximize the ROS production. a) Structural variations of ring‐A and ring‐B on FP chromophore scaffold. EDGs: 2‐6; EWGs: 7‐12; Heavy atoms (S/I atoms): 11, 12. b) Relative ROS yields (Φ_ROS_) measured by DCFH assay normalized to KillerRed. 124 analogs were obtained in total. Illumination intensity: 3.3 mW•cm^−2^. c) A7B3 (P0) exhibited the highest Φ_ROS_, over 1000 fold enhancement compared to KillerRed. d) Heavy atom (I/S) heterocyclic ring substitution showed higher median Φ_ROS_. *n* = 19 in substituent 1 group, *n* = 15 in substituent 11 group, *n* = 13 in substituent 12 group. ^*^, *p* < 0.05 (unpaired *t*‐test). e) Statistical analysis of single‐end substitution of EDGs and EWGs to vary electronic effect resulted in no significant impact on averaged Φ_ROS_. *n* = 46 in A^D^ group, *n* = 34 in A^W^ group, *n* = 42 in B^D^ group, *n* = 38 in B^W^ group. *ns*, no significant (unpaired *t*‐test). f) Directionality of the push‐pull effect dictated Φ_ROS_. Group A^W^B^D^ exhibited higher Φ_ROS_ than those of group A^D^B^W^. *n* = 19 in A^D^B^D^ group, *n* = 14 in A^W^B^W^ group, *n* = 14 in A^D^B^W^ group, *n* = 13 in A^W^B^D^ group. ^*^, *p*< 0.05 (unpaired *t*‐test). g) The averaged dipole moment (µ) of probes in group A^W^B^D^ was larger than that of the A^D^B^W^ group shown by DFT calculation. *n* = 14 in A^D^B^W^ group, *n* = 13 in A^W^B^D^ group. ****, *p* < 0.0001 (unpaired *t*‐test). h) A7B3 as an example exhibited much higher relative Φ_ROS_ than A3B7 due to its larger dipole moment (µ).

To maximize photosensitization, we constructed a 12 × 12 combinatorial library consisting of 124 FP chromophore analogs through a facile two‐step synthetic route (**Figure** **2**a, 20 were not obtained due to synthetic inaccessibility and stability issues). In this array, we employed common chemical strategies^[^ ^19^ ^]^ to improve the triplet excited state photosensitization of FP analogs, including modulation of electronic effect and substitution of heavy atoms. For example, compared to substituent 1, substituent 2 with additional dimethylamino as an electron donor affected the excited state charge distribution via intramolecular charge transfer (ICT) or twisted intramolecular charge transfer (TICT). Subsequently, compared to substituent 2, substituent 3 with extended π‐conjugation affected the electron delocalization through the molecule. Meanwhile, substituent 11 and substituent 12 with sulfur and iodine heavy atoms potentially enhanced spin‐orbit coupling and improved the intersystem crossing (ISC) efficiency. These structural variations will consequently impact the distribution of excited state energy. The photosensitization ability as manifested by the relative ROS production yield (Φ_ROS_) was quantified by DCFH assay (Figure S1a, Supporting Information).^[^ ^20^ ^]^ Among all probes, we identified an A7B3 (P0) probe that exhibited more than 1000 fold enhancement in Φ_ROS_ as compared to that of natural KillerRed FP (Figure 2c).

We next investigated chromophores’ structural features that improve the Φ_ROS_, focusing on three key factors: 1) heavy atom effect; 2) electronic effect; and 3) directionality of push‐pull effect. As expected, the introduction of sulfur/iodine‐containing heterocycles (column A11 & A12, row B11 & B12) obviously enhanced triplet properties shown by the median Φ_ROS_ distribution (Figure 2d, Φ_ROS, A1&B1_ = 8, Φ_ROS, A11&B11_ = 37, Φ_ROS, A12&B12_ = 72). Second, the single‐end substitution of EDGs and EWGs to vary electronic effect resulted in no significant impact on averaged Φ_ROS_ (Figure 2e, Φ_ROS_A^D^ vs Φ_ROS_A^W^ and Φ_ROS_B^D^ vs Φ_ROS_B^W^). Third, the directionality of the “push‐pull” effect, however, exerted an impact on Φ_ROS_. Ring‐swapping of A and B substitutions categorized these probes into 4 groups: A^D^B^D^, A^W^B^W^, A^D^B^W^, and A^W^B^D^. Statistically, group A^W^B^D^ exhibited higher averaged Φ_ROS_ than group A^D^B^W^ (Figure 2f). This difference was possibly due to asymmetric charge distribution, as evidenced by larger calculated dipole moments (µ) of group A^W^B^D^ (µ_avg_ = 11.31) compared to that of A^D^B^W^ (µ_avg_ = 6.56) (Figure 2g). Thus, FP chromophores with larger dipole moment (µ) likely exhibited higher ROS yield (Φ_ROS_) (Figure S2, Supporting Information), as exemplified by the A3B7 and A7B3 pair with swapped directionality between EDG and EWG (Figure 2h).

### 2.2 Ultra‐Photosensitized FP Chromophore Photocatalyzed Protein Proximity Labeling (PPL)

**Figure 3:**
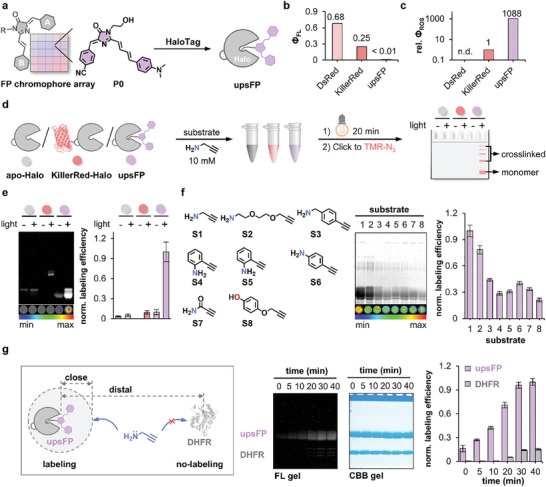
Ultra‐photosensitized FP chromophore enabled photocatalytic protein proximity labeling (PPL) function. a) The ultra‐photosensitized FP chromophore (P0) was conjugated to HaloTag mimicking photosensitized FPs, namely upsFP tag. b,c) The upsFP tag diverted excited state energy to triplet state ROS production without showing fluorescence. d) Schematic to quantify photocatalytic protein labeling efficiency using fluorescent SDS‐PAGE gel. Apo‐Halo in grey, KillerRed‐Halo conjugate in red, and upsFP (P0‐Halo conjugate) in purple were chosen as models. e) The upsFP outperformed KillerRed in protein labeling efficiency using propargylamine (PA) as a labeling substrate. Data are shown as the mean ± SEMs, *n* = 3 per group. f) Optimization of labeling efficiency using different nucleophilic substrates. Data are shown as the mean ± SEMs, *n* = 3 per group. g) Selective labeling of proteins in proximity. Data are shown as the mean ± SEMs, *n* = 3 per group. [upsFP] & [DHFR] = 50 µm; [GSH] = 5 mM to mimic cellular environment. Unless otherwise noted, experimental conditions were: [upsFP] = 30 µm; [substrate] = 10 mM; Illumination intensity: 10 mW•cm^−2^; Illumination time: 20 min. Error bars: standard error (n = 3). Dot experiments were performed on nitrocellulose membrane and fluorescently quantified.

We examined whether the ultra‐photosensitized FP chromophore developed above (A7B3, P0) upon conjugation to HaloTag (Figure S3a,b, Supporting Information), namely the upsFP tag, can perform photo‐catalytic protein proximity labeling (PPL) (**Figure** **3**a). First, compared to natural FPs (DsRed^[^ ^21^ ^]^ and KillerRed^[^ ^5^, ^22^ ^]^), the upsFP tag effectively ablated all singlet state fluorescence (Φ_FL_) while maximizing its triplet state photosensitization (Φ_ROS_) (Figure 3b,c). Such enhanced ROS production enabled upsFP for self‐labeling of HaloTag protein in proximity using the nucleophilic aminoalkyne substrate upon photo‐illumination (Figure 3d, e, right two lanes in purple) with sustained photostability (Figure S3c, Supporting Information). In contrast, KillerRed in conjugation with Halo protein failed to perform effective PPL function as reported previously (Figure 3e; Figure S4, Supporting Information middle two lanes in red).^[^ ^17^, ^18^ ^]^ To optimize protein labeling efficiency, we down‐selected a collection of nucleophilic substrates (Figure 3f; Figure S5, Supporting Information), including aliphatic amine, benzylamine, aniline, amide, and phenol. Among them, the mini‐sized S1 (PA) outperformed others possibly due to its better nucleophilicity and less steric hindrance. The efficiency of protein labeling photocatalyzed by P0 chromophore was shown to be dependent on illumination time (Figure S6, Supporting Information), light intensity (Figure S7, Supporting Information), substrate concentration (Figure S8, Supporting Information), and P1 concentration (Figure S9, Supporting Information).

Importantly, the distance‐controlled labeling (i.e. proximity effect) catalyzed by upsFP was validated in the presence of a non‐interacting protein (DHFR) (Figure 3g). Within the initial 10 min, protein labeling only occurred on HaloTag in proximity to the P0 probe but not on non‐interacting DHFR proteins in the distal end. As photo‐illumination prolonged, selective labeling was still confined within HaloTag protein, with minimal off‐target labeling on non‐interacting DHFR (Figure 3g). It also estimated the labeling radius as less than 100 nm referring to the size of the HaloTag protein (≈85 nm). Experiments in more complicated environments, such as cell lysates, further confirmed the selectivity of labeling mediated by upsFP (Figure S10, Supporting Information) and applicability in interactome studies (Figure S11, Supporting Information). Overall, the upsFP probe enabled robust and selective photo‐catalytic protein proximity labeling, which was previously impractical for natural FPs.

### 2.3 Mechanism of Photocatalyzed Protein Labeling

**Figure 4:**
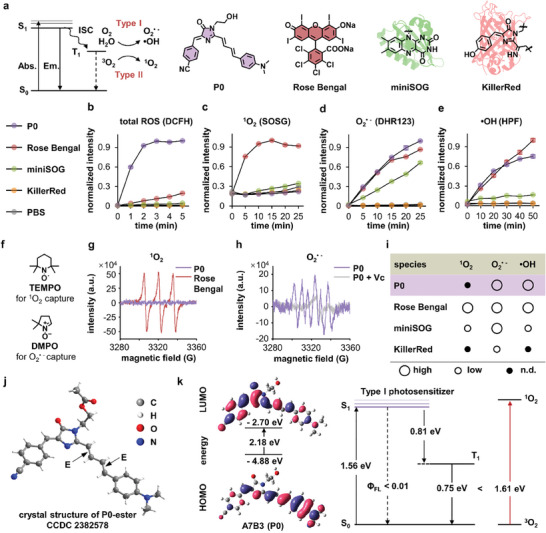
Mechanistic insights into the triplet excited state photosensitization pathway of P0. a) Photosensitization pathways: type I via radials and type II via single oxygen. Selected photosensitizers (3 µm): P0 from this work, Rose Bengal small molecule photosensitizer, miniSOG photosensitized protein, and KillerRed photosensitized FP. b) Total ROS measurement using DCFH assay (9 µm, λ_ex_: 488 nm, λ_em_: 525 nm). c) Singlet oxygen (^1^O_2_) detected by SOSG assay (10 µm, λ_ex_: 504 nm, λ_em_: 525 nm). d) Superoxide anions (O_2_^•−^) detected by DHR123 assay (6 µm, λ_ex_: 480 nm, λ_em_: 525 nm). e) Hydroxyl radicals (•OH) detected by HPF assay (6 µm, λ_ex_: 488 nm, λ_em_: 514 nm). Illumination intensity: 3.3 mW•cm^−2^; Data are shown as the mean ± SEMs, *n* = 3 per group. f) Structure of TEMPO (^1^O_2_ scavenger) and DMPO (O_2_^•−^ and •OH scavenger). g) EPR spectra using TEMPO (20 mM) as the spin trap to detect ^1^O_2_. h) O_2_^•−^ radicals generated by P0 (1 mM) were detected by EPR using DMPO as the spin trap (25 mM in methanol). Illumination: 10 mW•cm^−2^, 10 min. i) Summary of different ROS species produced by common photosensitizers. j) Ground state conformation of P0‐ester shown by X‐ray crystallography. k) TD‐DFT calculation demonstrates a mismatched energy gap that forbids ^1^O_2_ production.

**Figure 5:**
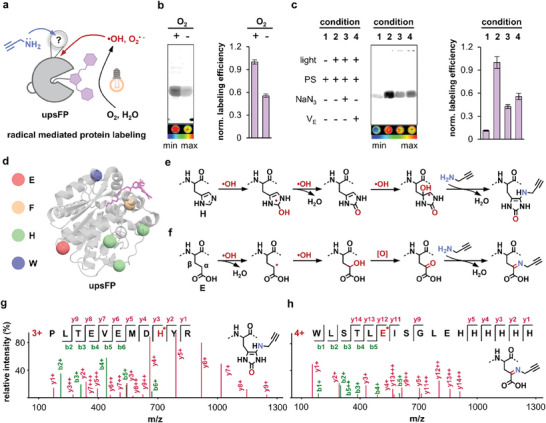
Radical mediated protein labeling and its modification mechanisms. a) Radial‐mediated protein labeling by aminoalkyne. b) The labeling efficiency of upsFP was quenched by the depletion of oxygen. Data are shown as the mean ± SEMs, *n* = 3 per group. c) The labeling efficiency of upsFP was quenched by ROS scavengers. Data are shown as the mean ± SEMs, *n* = 3 per group. [NaN_3_]: 10 mM; [V_E_]: 10 mM. d) Modification sites and amino acid types on Halo protein identified by LC‐MS/MS. PDB code: 6U32. e,f) Plausible labeling mechanisms on H and E residues. g,h) LC‐MS/MS spectra of modified peptides on H and E residues. [upsFP]: 30 µm; [PA]: 10 mM; Illumination intensity: 10 mW•cm^−2^; Illumination time: 20 min. Error bars: standard error (n = 3).

We next investigated two fundamental questions regarding the protein labeling mechanism catalyzed by P0: 1) the type of ROS species produced by P0 that mediated protein labeling (**Figure** **4**); 2) the labeling residues and linkage on a protein (**Figure** **5**).

To identify which photosensitization pathway P0 underwent (Type I via radicals vs Type II via singlet oxygen), we identified the ROS species using a collection of ROS sensors (Figure 4b–e; Figure S1, Supporting Information), including DCFH (for total ROS), SOSG (for ^1^O_2_), DHR123 (for O_2_^•−^), and HPF (for •OH). Compared to Rose Bengal photosensitizer, miniSOG photosensitized protein, and KillerRed FP (Figure 4a), our ultra‐photosensitized P0 excelled in total ROS production (Figure 4b). While Rose Bengal and miniSOG were shown to produce ^1^O_2_ in the SOSG assay (Figure 4c), P0 did not undergo a Type II singlet oxygen pathway. On the other hand, P0 produced O_2_^•−^ and •OH as evidenced by DHR123 and HPF assays (Figure 4d, e). Electron paramagnetic resonance (EPR) spectroscopy also supported Type I radial pathway using TEMPO and DMPO as respective scavengers (Figure 4f–i). Finally, based on the ground state (S_0_) conformation obtained from X‐ray crystallography (Figure 4j), TD‐DFT calculation revealed that the energy gap of P0 between the triplet excited state (T_1_) and ground state (S_0_) did not match between triplet oxygen (^3^O_2_) and singlet oxygen (^1^O_2_) (Figure 4k), highlighting its incapability of producing ^1^O_2_. Together, photochemical, spectroscopic, and theoretical data all suggested that P0 underwent an effective Type I radial pathway to mediate protein labeling.

We further dissected how these radicals mediated protein labeling with aminoalkyne substrate (Figure 5a). Upon oxygen depletion in the buffer, labeling efficiency was halved, confirming that covalent labeling involved the photo‐oxygenation step (Figure 5b; Figure S12, Supporting Information). Furthermore, depletion of •OH and O_2_^•−^ radicals by NaN_3_ and V_E_ scavengers also compromised labeling efficiency, supporting radical‐mediated labeling mechanism (Figure 5c; Figure S13, Supporting Information). Next, we exploited tandem mass spectrometry (LC‐MS/MS) to identify modified amino acid residues and deduce plausible labeling mechanisms based on identified labeling products.

First, we showed that multiple types of residues on Halo protein were oxidized by •OH and O_2_^•−^, including H, E, F, W, and M (Figures S14–S19, Supporting Information), and modified by the aminoalkyne substrate, including H, E, F, and W (Figure 5d; Figure S20–S23, Supporting Information). The expanded scope of labeling sites compared to miniSOG (Figure S24, Supporting Information, only on H) may assist in better enrichment and coverage in downstream proteomic applications. Second, the modification adduct on H residue via upsFP photo‐catalysis was the same as that by miniSOG enzyme (Figure 5g; Figure S24, Supporting Information). Although the final labeling product was the same as the miniSOG scenario, we proposed an alternative plausible reaction mechanism for upsFP that underwent a radial‐based pathway (Figure 5e)^[^ ^23^ ^]^ due to the absence of ^1^O_2_ (Figure 4). Meanwhile, a special modification on glutamic acid was identified, which may be initiated by extracting α‐H via •OH radical and oxidation to α‐carbonyl acid intermediate (Figure 5f,h).^[^ ^24^ ^]^ Likewise, the labeling on phenylalanine may also undergo similar extraction of α‐H by •OH radical (Figure S22, Supporting Information). Overall, deduced by the labeled peptide products, our tandem MS results confirmed that the labeling reactions of upsFP were mediated by radicals on diverse amino acid residues.

### 2.4 The upsFP Tag Labeled and Profiled TDP43 Interactome Upon Phase Separation

**Figure 6:**
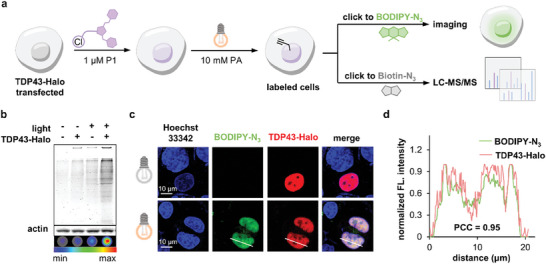
The upsFP tag enabled photocatalytic proximity labeling of the TDP43 interactome in the cell. a) Scheme for proximity labeling of TDP43‐Halo interactome for downstream imaging and proteome enrichment. b) Fluorescent gel and dot assay to show selective labeling of TDP43 interacting proteome in transfected cells upon photo‐illumination. c,d) The proximity labeling region (green fluorescence upon clicking to BODIPY‐N3) colocalized well with TDP43‐Halo (red fluorescence upon conjugating to TMR‐Halo probe). For imaging, 0.1 µm TMR Halo probe was mixed with 1 µm P1. For details see Supporting Information.

Given its proximity labeling function, we exemplified a proteomic profiling application using the upsFP tag (HaloTag‐P0 conjugate) to resolve the interactome variations of TAR DNA‐binding protein 43 (TDP43) upon its phase separation and suborganellar translocation. The phase separation process of the TDP43 protein is associated with the pathology of amyotrophic lateral sclerosis (ALS) and frontotemporal dementia (FTD).^[^ ^25^ ^]^ Unraveling the dynamic changes of TDP43's interacting partners is of significance to deciphering its pathogenicity. To capture its interactome with temporal resolution, we expressed TDP43‐Halo in HEK293T cells, labeled it with P1, and photo‐illuminated the cells at different stressed stages. First, at basal conditions, fluorescent gel and dot analysis validated that TDP43‐upsFP achieved labeling of interacting proteome upon photo‐illumination selectively in transfected cells (**Figure** **6**b; Figure S25, Supporting Information). Further confocal co‐localization imaging of labeled region (green fluorescence, click to BODIPY‐N_3_) and TDP43‐Halo proteins (red fluorescence, conjugated to TMR‐Halo probe) showed satisfactory spatial alignment (Pearson's correlation coefficient: 0.95) and confinement in the nucleus (Figure 6c,d; Figure S26, Supporting Information). These results verified that the labeling actually occurred in proximity to TDP43 proteins.

**Figure 7:**
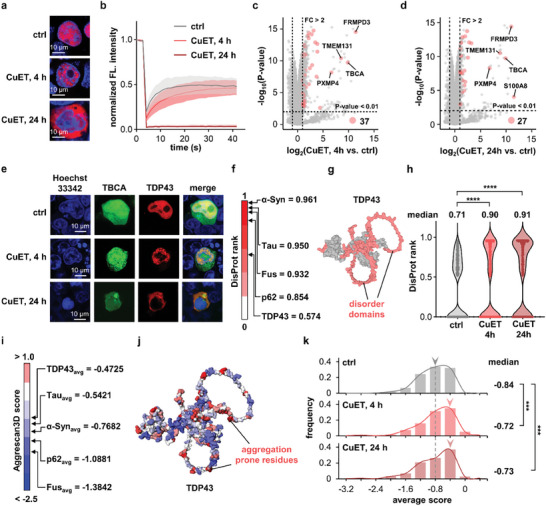
Application of upsFP to profile TDP43 interactome upon phase separation. a) Confocal images of TDP43‐Halo‐TMR upon acute CuET stress (5 µm, 4 h) and chronic stress (24 h). TDP43 phase‐separated into droplets within the cellular nucleus at 4 h and subsequently translocated into the cytoplasm at 24 h. b) FRAP assay revealed the differences in TDP43's fluidity. c,d) Volcano plot of the differentially enriched phase separated interactome upon CuET treatment. Featured cell skeleton‐relevant proteins were marked in red. e) Imaging validation of TBCA co‐condensated with TDP43 during the phase separation process. f) The DisProt rank described the intrinsic disorderness of common intrinsically disordered proteins. g) Using TDP43 as an example to illustrate its disordered domain. The disordered domain highlighted in red was predicted by ESpritz (http://old.protein.bio.unipd.it/espritz). h) DisProt rank distribution of TDP43 interactome upon phase separation. The DisProt rank of each protein was collected from PhaSePred (https://predict.phasep.pro). i) The average Aggrescan3D score of common phase separated proteins. j) The aggregation‐prone residues of TDP43 were highlighted in red and predicted by A3D Server (https://biocomp.chem.uw.edu.pl/A3D2/MODB). k) Aggrescan3D score distribution of interactome upon phase separation stress.

Copper diethyldithiocarbamate (CuET) was reported to cause phase separation of TDP43.^[^ ^26^ ^]^ Upon CuET acute stress (5 µm, 4 h), the TDP43 phase separated into droplets in the cellular nucleus and later translocated to cytoplasm forming solid aggregates after chronic stress (24 h). Fluorescence recovery after photobleaching (FRAP) assay measuring the fluidity distinguished these two‐phase separation processes as liquid (LLSP) and solid (LSPS) states (**Figure** **7**a,b). During the labeling period, the morphologies of TDP43 and cell viability (PI staining assay) were not significantly compromised (Figure S27, Supporting Information). After biotin‐streptavidin enrichment and LC‐MS/MS comparative analysis, we identified 356 differentially enriched interacting proteins in the LLPS group (CuET, 4 h) versus 218 hits in the LSPS group (CuET, 24 h) in comparison to the basal control group (Figure 7c,d). Gene ontology (GO) analysis revealed that these proteins were functionally enriched in nucleic acid binding and protein degradation (Figures S28 and S29, Supporting Information). Notably, proteins related to the cell skeleton (highlighted in Figure 7c,d) were prominent in both groups. This finding was well‐aligned with recent literature reports,^[^ ^27^ ^]^ which highlighted the adhesion of phase‐separated stress granules toward the tubulin network.^[^ ^28^ ^]^ These observations indicated that cell skeleton‐related proteins likely participated in TDP43's phase separation and translocation processes. To validate this notion, we co‐transfected one of the identified cytoskeleton‐related proteins TBCA‐eGFP and TDP43‐Halo together in HEK293T cells. As expected, upon CuET stress for 4 h, TBCA partially condensed together with TDP43 into the droplets. After an additional 20 h prolonged stress, the translocated TDP43 in cytoplasm still colocalized well with TBCA (Figure 7e). Similar observations were also made in both HeLa and U‐2OS cell lines (Figures S30 and S31, Supporting Information), confirming the reliability of the proteomic findings. The small molecule regulation nature of the upsFP tag renders it suited for potential pulse‐chase proximity labeling experiments to resolve interactome variations during the basal‐stress‐recovery process (Figures S32–S34).

Finally, we analyzed the physical properties of TDP43 interacting proteins entrapped within TDP43 condensates during liquid‐liquid phase separation (LLPS, 4 h stress) and liquid‐solid translocation (LSPS, 24 h stress). We first evaluated the DisProt rank of proteins enriched in phase‐separated interactome. This biophysical parameter considers the intrinsically disordered regions of proteins that drive phase separation, as highlighted in TDP43 protein and other common intrinsically disordered proteins (IDPs) (Figure 7f,g).^[^ ^29^ ^]^ Upon stress, this parameter describing the average disorderness of TDP43 interactome slightly increased, with median values from 0.71 to 0.90 and 0.91, respectively (Figure 7h), indicating exaggerated phase separation propensity. Second, we also examined the Aggrescan3D score, which described protein intrinsic aggregation propensity. Interestingly, this score exhibited a similar trend with the above disorder rank, with median values from −0.84 to −0.72 and −0.73, respectively. Together, increased DisProt rank and Aggrescan3D score of TDP43 interactome upon stress confirmed their higher phase separation and aggregation propensity (Figure 7i,j,k), consistent with previous results.^[^ ^30^ ^]^ These results also supported that the interactome profiled by our method was intrinsically prone to protein phase separation.

## 3 Conclusion

In conclusion, this work enhanced the photosensitizing properties of synthetic FP chromophores up to 1000 fold (Φ_ROS_) as compared to the natural KillerRed FP. Coupled with HaloTag technology, the ultra‐photosensitized FP chromophore enabled the protein proximity labeling (PPL) function. Mechanistic experiments confirmed radical‐mediated protein modification through the type I photosensitization pathway. Finally, we exemplified its proteomic profiling application in resolving TDP43 protein interactome upon its phase separation. We revealed the functional and biophysical characteristics of phase‐separated interactome. Overall, to the best of our knowledge, we for the first time constructed an artificial FP mimic with a protein proximity labeling function for interactome profiling of phase‐separated proteins.

## 4 Experimental Section

### ROS Quantum Yield Definition

The overall reactive oxygen species (ROS) production from the probes under white light illumination was determined using the 2,7‐dichlorodihydrofluorescein (DCFH) assay.

(1) $$\Phi_{\mathrm{ROS}}=K_{\mathrm{probe}}/A_{\mathrm{probe}}$$

where K_probe_ was the oxidation rate constant of DCFH by the ROS produced by probes versus irradiation time in PBS buffer. A_probe_ was the absorbance of probes in the PBS buffer. The fluorescence of DCF and the absorbance of probes in PBS buffer were quantified by Tecan Spark Fluorescence Plate Reader in BeyoGold 96‐Well transparent plate.

### ROS Definition

For singlet oxygen (^1^O_2_) detection, a commercially available kit, Singlet Oxygen Sensor Green (SOSG) was utilized. Specifically, a 5 mm stock solution of SOSG was freshly prepared in methanol in darkness. In practice use, the working concentration was 10 µm and the fluorescence at 525 nm of SOSG‐EP which was the reaction product of SOSG and ^1^O_2_ was recorded at different light illumination times under the excitation of 504 nm;

For superoxide anions (O_2_^•−^) detection, a commercially available kit, dihydrorhodamine 123 (DHR 123) was utilized. Specifically, a 1.0 mm stock solution of DHR123 was prepared in DMSO and stored at −20 °C in darkness. In practice use, the working concentration was 6.0 µm and the fluorescence at 525 nm of rhodamine123 which was the reaction product of DHR 123 and superoxide anions was recorded at different light illumination times under the excitation of 480 nm.

For hydrogen radical (•OH) detection, a commercially available kit, hydroxyphenyl fluorescein (HPF) was utilized. Specifically, 1.0 mm stock solution of HPF was prepared in DMF and stored at −20 °C with light‐free. In practice use, the working concentration was 3.0 µm, and the fluorescence at 514 nm of fluorescein which was the reaction product of HPF and •OH was recorded at different light illumination times under the excitation of 488 nm.

For type I reactive oxygen scavenging, sorbitol was used. Specifically, a 100.0 mm stock solution of sorbitol was prepared from distilled water and stored in the dark at −20 °C. In practice use, the working concentration was 10.0 mm to eradicate type I reactive oxygens produced by the 20.0 µm probe; For type II reactive oxygen scavenging, NaN_3_ was used. Specifically, 100.0 mm stock solution of sorbitol was prepared in distilled water and stored in the dark at −20 °C. In practice use, the working concentration was 10.0 mm to eradicate type II reactive oxygens produced by 20.0 µM probe.

### Calculation Method

The initial molecular structure selected in this article was constructed using GaussView6.0 software. The compound used in this study adopts the B3LYP‐D3 method of density functional theory (DFT), and further optimizes the molecular structure to convergence using the 6‐31G (d) basis set. The solvation model was selected as SMD, and the solvent was selected as water. The calculation of excited states was carried out using time‐dependent density functional theory (TD‐DFT) at the same fundamental level. All the above calculations were completed on the Gaussian 16 program.

### General Procedure of Photocatalysis Labeling In Vitro

A solution of 30 µm HaloTag and 30 µm P1 was mixed in 300 µL of PBS and incubated at room temperature for 30 min to prepare upsFP. Subsequently, 10 mm propargylamine (PA) was introduced. Following illumination with white LEDs (10 mW•cm^−2^) for 20 min, a 4 fold excess of precooled acetone was added to the sample, which was then incubated at −20 °C overnight. Next, the protein precipitates were redissolved in 200 µL of 10% SDS, and 50 µL of click reagent was added to each sample (consisting of 1300 µL of 1.7 mm TBTA, 440 µL of 50 mm CuSO_4_, 440 µL of 50 mm TCEP, and 12 µL of 50 mm TMR‐N_3_). The sample was shaken at room temperature for 1 h. Subsequently, a 4 fold excess of precooled acetone was added to the sample, which was then incubated at −20 °C overnight. The sample was then washed three times with precooled acetone to completely remove excess click reagents.

Ultimately, the sample was redissolved in 10% SDS, thus rendering it suitable for SDS‐PAGE and dots experiments.

### Procedure for Cell Culture, Transfection of Cells, and Cell Imaging Experiments

HEK293T cells were seeded on 35 mm confocal culture dishes and transiently transfected when the cell density reached 70%. In 200 µL opti‐MEM medium, 3 µL of X‐tremegene 9 DNA transfection reagent (Roche) was added. The plasmid of TDP43‐Halo (1 µg) was then introduced to the solution. The mixture was fully mixed at room temperature for 25 min in darkness. Subsequently, it was then dripped into the cell medium and expressed for 48 h. 0.5 µm TMR Halo probes were added. Next, the transfected cells were treated with 5 µm CuET for 4h or 24 h. Hoechst 33342 (1000×) was added to the cell medium 30 min before imaging. Images were collected using Olympus FV1000MPE.

### General Procedure of Photocatalysis Labeling In Vivo (for Imaging)

After transfection and incubation with 1 µm P1 (with or without 0.1 µm TMR‐Halo probe) for 48 h on 35 mm confocal culture dishes, HEK293T cells were washed with PBS three times, and then fixed with 4% paraformaldehyde fix solution for 30 min in darkness. Next, the fixed cells were washed with HBS three times. Subsequently, they were incubated with propargylamine (PA, 10 mM in HBS) in the dark for 20 min, followed by white light illumination for 20 min (10 mW•cm^−2^). The cells were washed with methanol and PBS three times. 500 µL PBS was added to keep it wet, and 50 µL of click reagent (consisting of 1300 µL of 1.7 mm TBTA, 440 µL of 50 mm CuSO_4_, 440 µL of 50 mm TCEP, and 12 µL of 50 mm BODIPY‐N_3_) were added. The sample was kept at room temperature for 1 h. Next, the cells were washed with methanol and PBS three times. Finally, Hoechst 33342 (1000×) was added before imaging. Images were collected using Olympus FV1000MPE.

### Statistical Analysis

All statistical analyses were performed using GraphPad Prism 10.1.2 software. To compare the two groups, an unpaired *t*‐test was used as appropriate. Data are presented as the mean ± SEMs or as individual data points. ^*^ *p* < 0.05, ^**^ *p* < 0.01, ^***^ *p* < 0.001, and ^****^ *p* < 0.0001 are considered statistically significant.

## Conflict of Interest

The authors declare no conflict of interest.

## Supporting information



Supporting Information

## Data Availability

The data that support the findings of this study are available in the supplementary material of this article.
