# Supplementary material for: 1000 fold Ultra‐Photosensitized Fluorescent Protein Mimics Toward Photocatalytic Proximity Labeling and Proteomic Profiling Functions
Source: Adv Sci (Weinh). 2025 Feb 22;12(15):2413063. doi: 10.1002/advs.202413063 (PMC12005797; doi:10.1002/advs.202413063)
Supplement: Supplementary file 1 — Supporting Information [file ADVS-12-2413063-s001.pdf]

ADVANCED SCIENCE
Open Access

Supporting Information

for *Adv. Sci.*, DOI 10.1002/advs.202413063

1000 fold Ultra-Photosensitized Fluorescent Protein Mimics Toward Photocatalytic Proximity Labeling and Proteomic Profiling Functions

Rui Sun, Yanan Huang, Huan Feng, Nan Zhao, Wang Wan, Di Shen, Bowen Zhong, Yukui Zhang, Xin Zhang, Qun Zhao, Lihua Zhang* and Yu Liu**

Supporting Information
©Wiley-VCH 2021
69451 Weinheim, Germany

1000-fold Ultra-Photosensitized Fluorescent Protein Mimics towards Photocatalytic Proximity Labeling and Proteomic Profiling Functions

Rui Sun, Yanan Huang, Huan Feng, Nan Zhao, Wang Wan, Di Shen, Bowen Zhong, Yukui Zhang, Xin Zhang, Qun Zhao, Lihua Zhang, Yu Liu

Abstract: Photosensitizing fluorescent proteins (FP) (e.g. KillerRed) have been shown not capable of photo-catalytic protein proximity labeling for downstream proteomic profiling applications. To acquire such function, we engineer FP chromophores in a 12×12 combinatorial matrix of synthetic analogues, achieving up to 1000-fold enhancement of ROS production compared to the natural FPs. We show FP chromophores with larger dipole moment exhibit higher ROS yield towards protein labeling. By conjugating the ultra-photosensitized FP chromophore to HaloTag (namely upsFP tag), we demonstrate its photo-catalytic protein proximity labeling function using nucleophilic amino substrates. Through photochemical characterizations, theoretical calculation, and tandem mass spectrometry, we reveal radical mediated labeling mechanism with expanded reactivity towards diverse protein residues via type I photosensitization pathway. Finally, we showcase a proteomic profiling application using the upsFP tag to resolve the dynamic interactome variations upon TAR DNA-binding protein 43 (TDP43) phase separation and suborganellar translocation. Together, this work demonstrates 3 orders of magnitude ultra-photosensitization of fluorescent protein chromophore enables photocatalytic protein proximity labeling and profiling functions that were impractical for natural fluorescent proteins.

Table of Contents

Table of Contents 2
1. Supporting Figures and Tables 3
2. Unmodified Gel Images 39
3. Experimental Procedures 52
3.1 Plasmids construction and protein purification 52
3.2 ROS quantum yield definition 52
3.3 General procedure of photocatalysis labeling in vitro 52
3.4 Procedure for cell culture, transfection of cells, and cell imaging experiments 52
3.5 General procedure of photocatalysis labeling in vivo (for imaging) 52
3.6 Cell Sample Preparation for Proteomic Analysis 53
3.7 Nano-LC-MS/MS Analysis 53
3.8 MS data analysis 54
3.9 Calculation method 54
3.10 LC-MS/MS Analysis of upsFP 54
4. Synthetic Procedures 56
5. NMR Spectra 75
6. References 208
7. Author Contributions 209

1. Supporting Figures and Tables

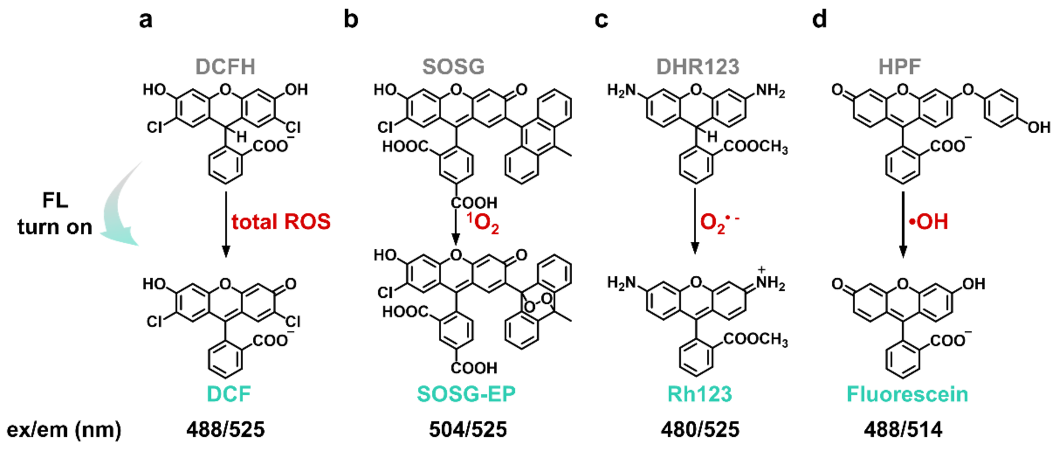

Figure S1. Mechanism of commercially available probes to detect reactive oxygen species. a. Total ROS measurement using DCFH assay (9 μM, λ_{ex}: 488 nm, λ_{em}: 525 nm). c. Singlet oxygen ($^1O_2$) detected by SOSG assay (10 μM, λ_{ex}: 504 nm, λ_{em}: 525 nm). d. Superoxide anions ($O_2^{\bullet -}$) detected by DHR123 assay (6 μM, λ_{ex}: 480 nm, λ_{em}: 525 nm). e. Hydroxyl radicals (•OH) detected by HPF assay (6 μM, λ_{ex}: 488 nm, λ_{em}: 514 nm).

a

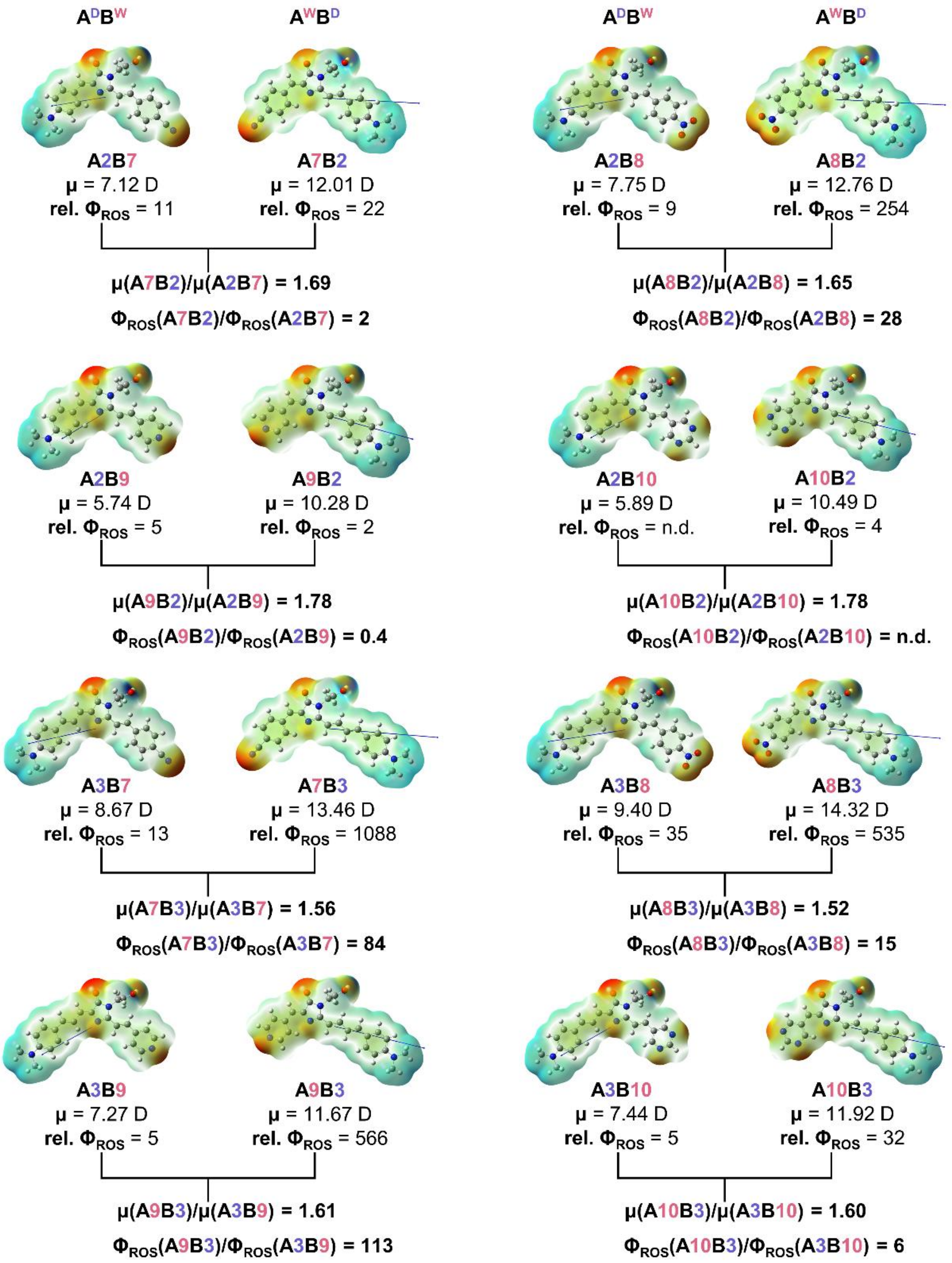
A^D B^W
A^W B^D
A^D B^W
A^W B^D
A2B7
μ = 7.12 D
rel. Φ_ROS = 11
A7B2
μ = 12.01 D
rel. Φ_ROS = 22
μ(A7B2)/μ(A2B7) = 1.69
Φ_ROS(A7B2)/Φ_ROS(A2B7) = 2
A2B8
μ = 7.75 D
rel. Φ_ROS = 9
A8B2
μ = 12.76 D
rel. Φ_ROS = 254
μ(A8B2)/μ(A2B8) = 1.65
Φ_ROS(A8B2)/Φ_ROS(A2B8) = 28
A2B9
μ = 5.74 D
rel. Φ_ROS = 5
A9B2
μ = 10.28 D
rel. Φ_ROS = 2
μ(A9B2)/μ(A2B9) = 1.78
Φ_ROS(A9B2)/Φ_ROS(A2B9) = 0.4
A2B10
μ = 5.89 D
rel. Φ_ROS = n.d.
A10B2
μ = 10.49 D
rel. Φ_ROS = 4
μ(A10B2)/μ(A2B10) = 1.78
Φ_ROS(A10B2)/Φ_ROS(A2B10) = n.d.
A3B7
μ = 8.67 D
rel. Φ_ROS = 13
A7B3
μ = 13.46 D
rel. Φ_ROS = 1088
μ(A7B3)/μ(A3B7) = 1.56
Φ_ROS(A7B3)/Φ_ROS(A3B7) = 84
A3B8
μ = 9.40 D
rel. Φ_ROS = 35
A8B3
μ = 14.32 D
rel. Φ_ROS = 535
μ(A8B3)/μ(A3B8) = 1.52
Φ_ROS(A8B3)/Φ_ROS(A3B8) = 15
A3B9
μ = 7.27 D
rel. Φ_ROS = 5
A9B3
μ = 11.67 D
rel. Φ_ROS = 566
μ(A9B3)/μ(A3B9) = 1.61
Φ_ROS(A9B3)/Φ_ROS(A3B9) = 113
A3B10
μ = 7.44 D
rel. Φ_ROS = 5
A10B3
μ = 11.92 D
rel. Φ_ROS = 32
μ(A10B3)/μ(A3B10) = 1.60
Φ_ROS(A10B3)/Φ_ROS(A3B10) = 6

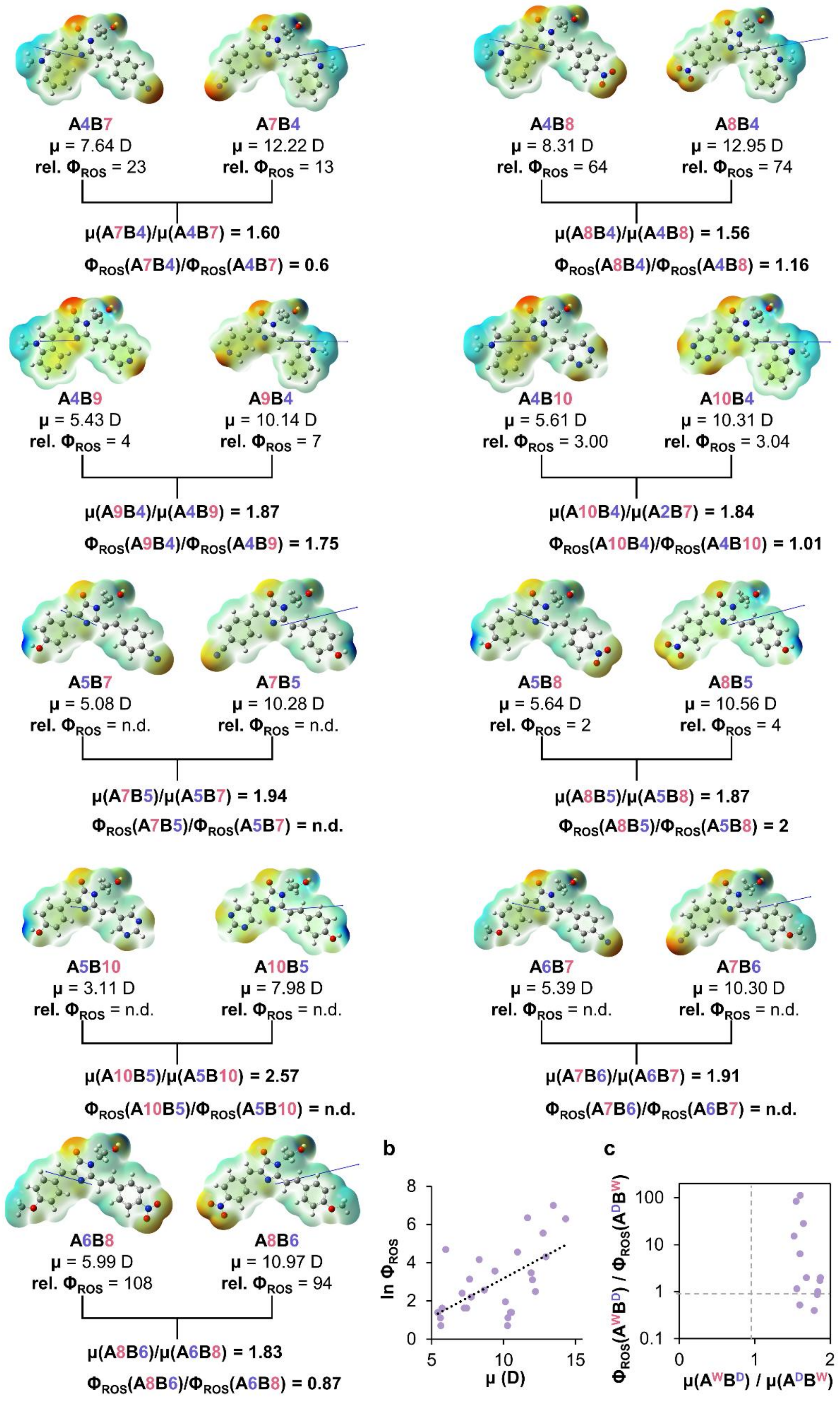

Figure S2. Dipole moments measurement by DFT calculation. a. Dipole moment (μ) and relative ROS generation efficiency (rel. Φ_{ROS}) of each selected group of A^DB^W and A^WB^D. **b.** Among the selected molecules, μ and rel. Φ_{ROS} exhibited positive correlation. **c.** For each

selected group of $A^{D}B^{W}$ and $A^{W}B^{D}$, $A^{W}B^{D}$ tend to exhibited higher μ and rel. Φ_{ROS} simultaneously, indicating the Φ_{ROS} of FP chromophores was likely controlled by its innate dipole moment. μ was calculated by Gaussian09, (B3LYP, 6-31g(d)).

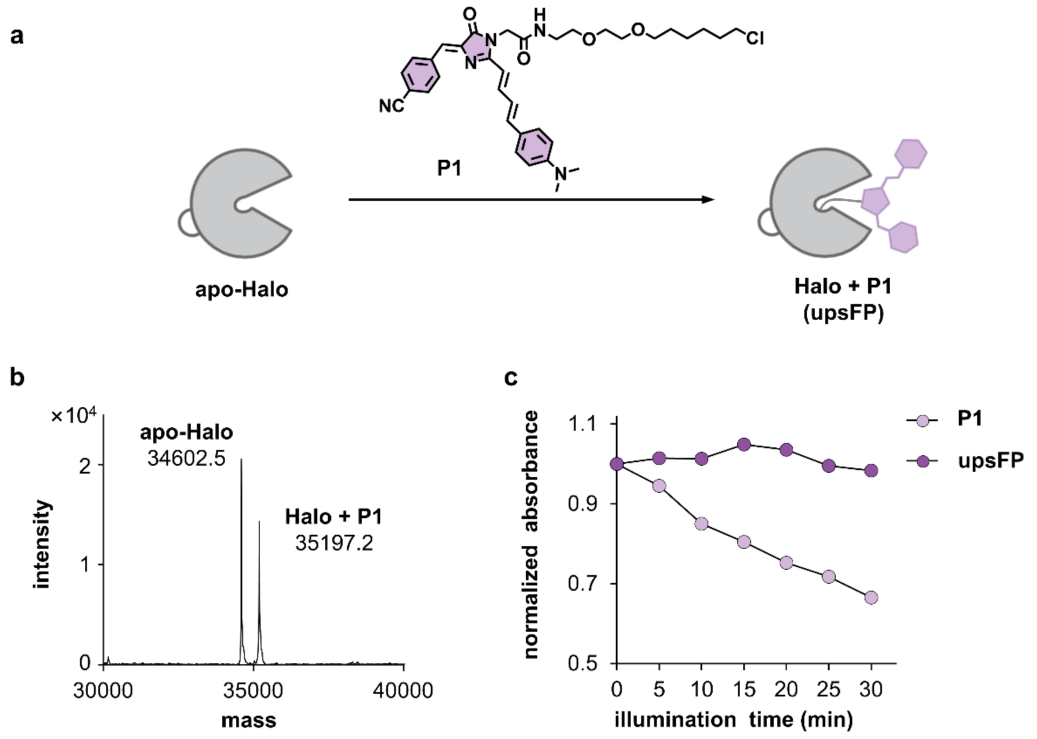

Figure S3. Conjugation of P1 to HaloTag protein and its photostability. **a.** By conjugation to HaloTag, we obtained upsFP. **b.** Complete conjugation of P1 to HaloTag shown by LC-MS/MS. The mass spectrometry was obtained by AB SCIEX TripleTOF 5600 Mass Spectrometer. **c.** After conjugation to HaloTag, the photostability of P1 was improved and sustained up to 30 min illumination. Illumination intensity: 10 mW•cm^{-2}.

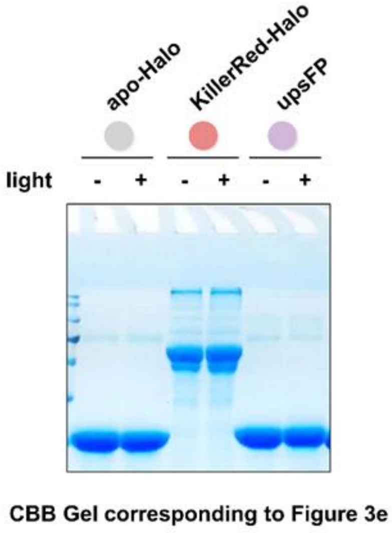

Figure S4. Coomassie Brilliant Blue (CBB) gel corresponding to Figure 3e.

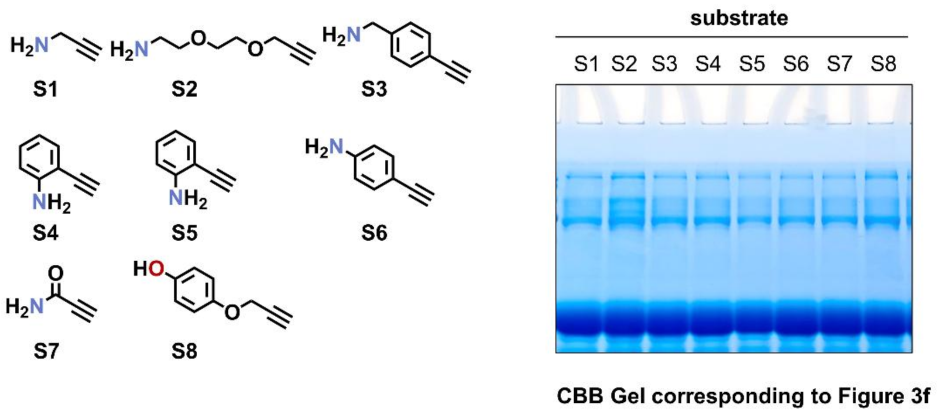

Figure S5. CBB gel corresponding to Figure 3f.

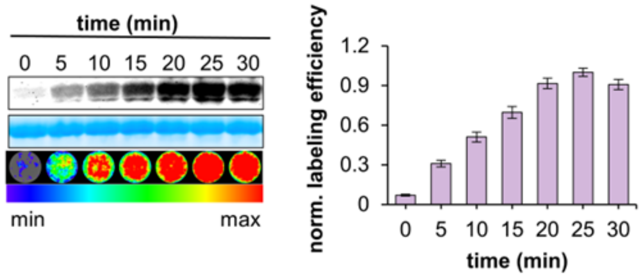

Figure S6. The labeling efficiency of upsFP was shown to be dependent on illumination time. Data are shown as the mean ± SEMs, *n* = 3 per group. [upsFP]: 30 μM; Illumination intensity: 30 mW•cm^{-2}. [PA]: 10 mM.

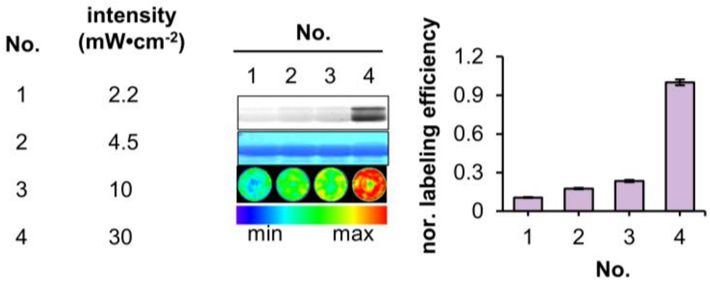

Figure S7. The labeling efficiency of upsFP was shown to be dependent on light intensity. Data are shown as the mean ± SEMs, n = 3 per group. [upsFP]: 30 μM; Illumination time: 10 min. [PA]: 10 mM.

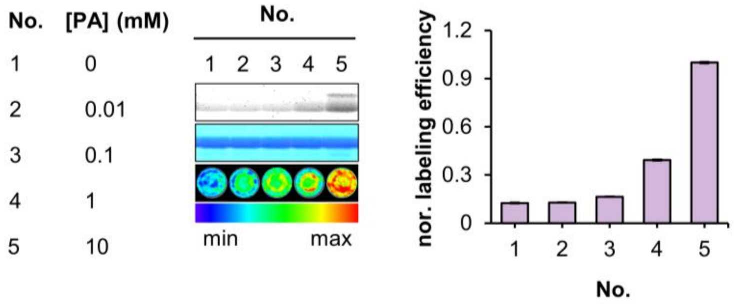

Figure S8. The labeling efficiency of upsFP was shown to be dependent on substrate concentration. Data are shown as the mean ± SEMs, n = 3 per group. [upsFP]: 30 μM; Illumination intensity: 10 mW•cm^{-2}; Illumination time: 10 min.

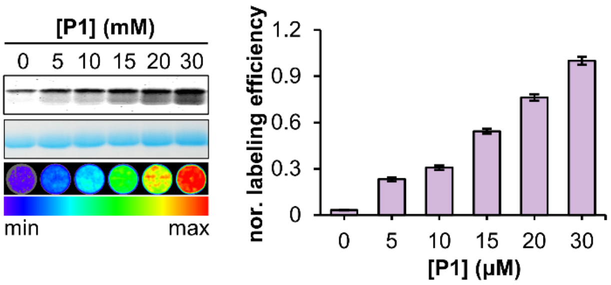

Figure S9. The labeling efficiency of Halo was shown to be dependent on P1 concentration. Data are shown as the mean ± SEMs, n = 3 per group. [Halo]: 30 μM; [PA]: 10 mM; Illumination intensity: 10 mW•cm^{-2}; Illumination time: 20 min.

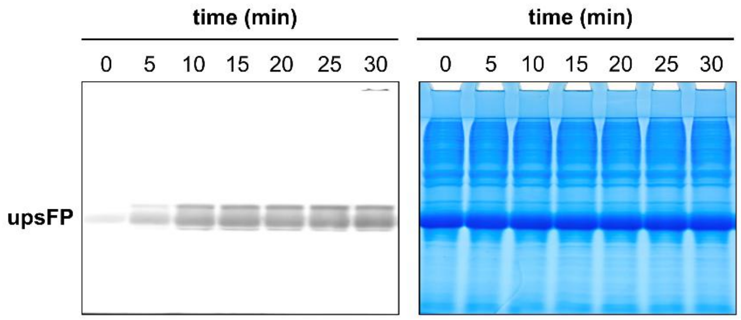

Figure S10. UpsFP selectively labeled itself in HEK293T cell lysate. [Halo]: 30 μM, approximate 1 mg•mL^{-1}; [Lysate]: 2 mg•mL^{-1}; [PA]: 10 mM; [GSH]: 5 mM; Illumination intensity: 10 mW•cm^{-2}.

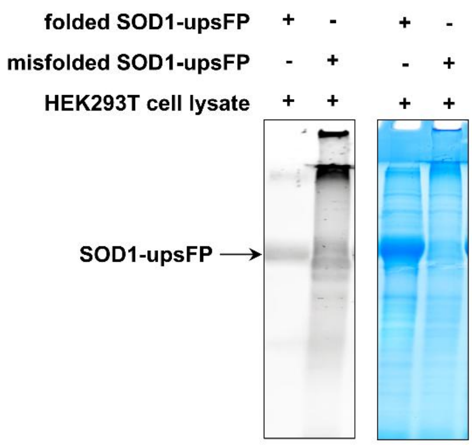

Figure S11. UpsFP labeled the interactome of misfolded SOD1 in HEK293T cell lysate. [SOD1-upsFP]: 20 μM, approximate 1 mg•mL^{-1}; [Lysate]: 2 mg•mL^{-1}; [PA]: 10 mM; [GSH]: 5 mM; Illumination intensity: 10 mW•cm^{-2}; Illumination time: 20 min.

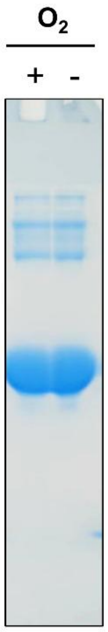

Figure S12. CBB gel corresponding to Figure 5b.

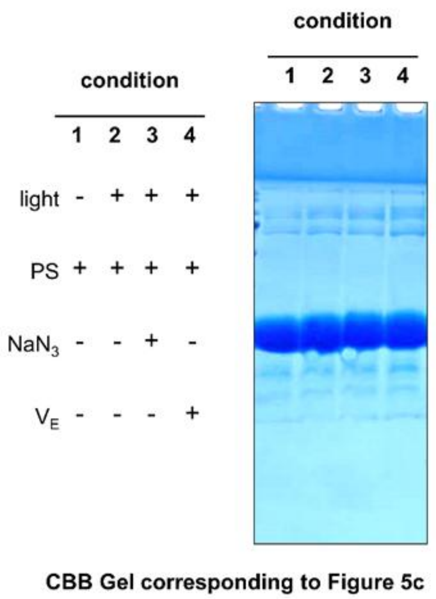

Figure S13. CBB gel corresponding to Figure 5c.

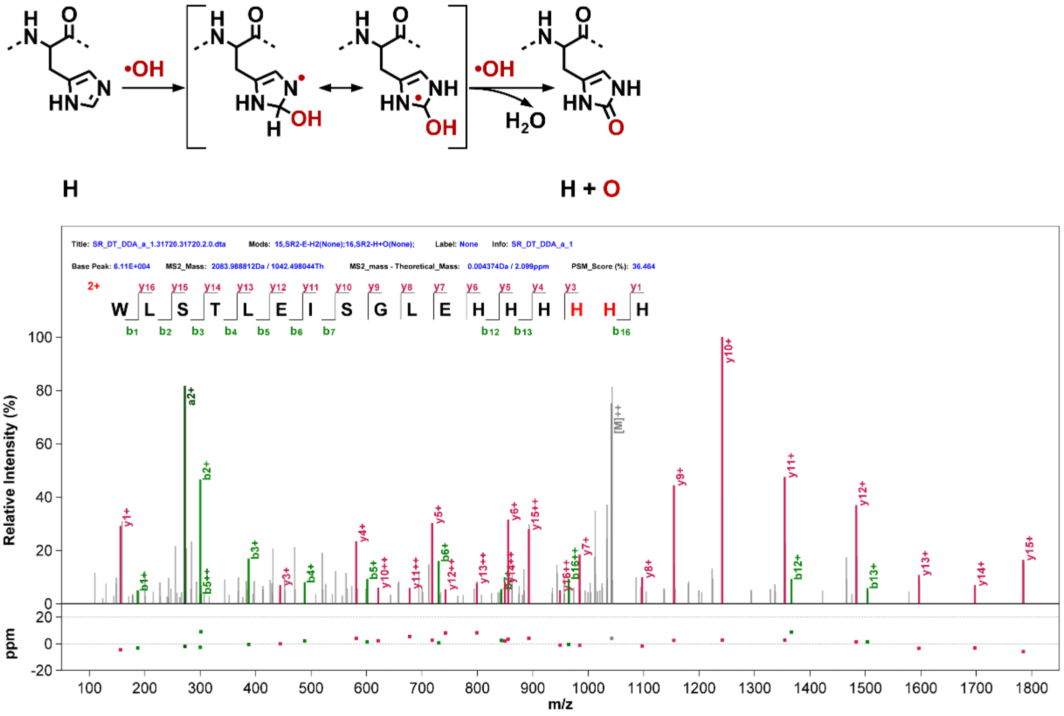

Figure S14. Proposed labeling mechanism of histidine oxidized by •OH on upsFP and corresponding MS spectrum.

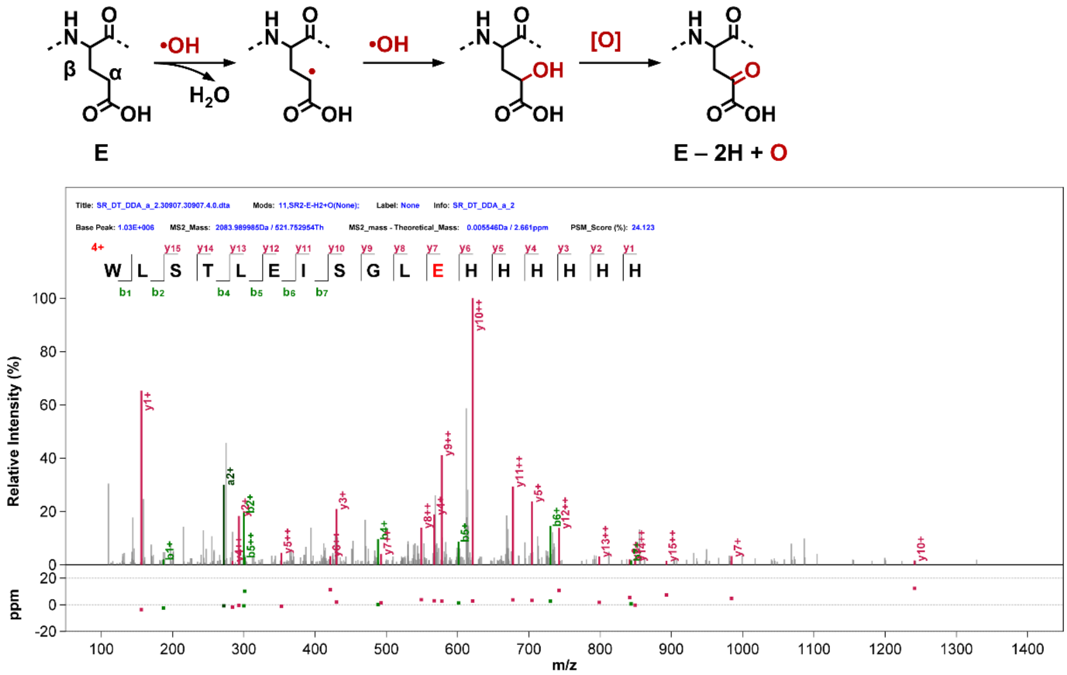

Figure S15. Proposed labeling mechanism of glutamic acid oxidized by •OH on upsFP and corresponding MS spectrum.

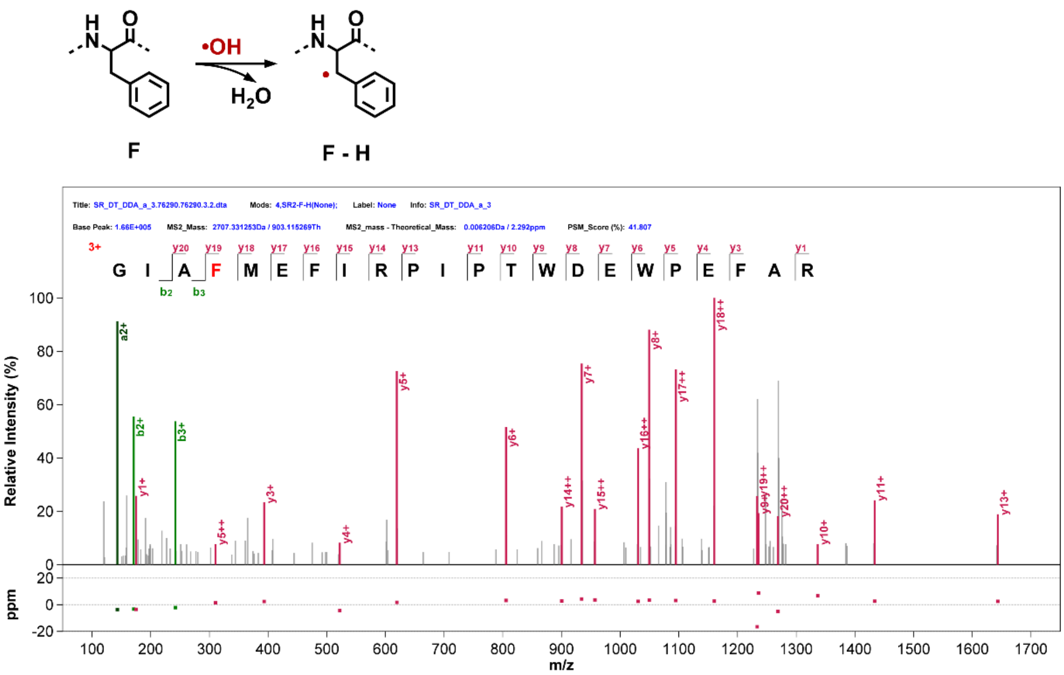

Figure S16. Proposed labeling mechanism of phenylalanine oxidized by •OH on upsFP and corresponding MS spectrum.

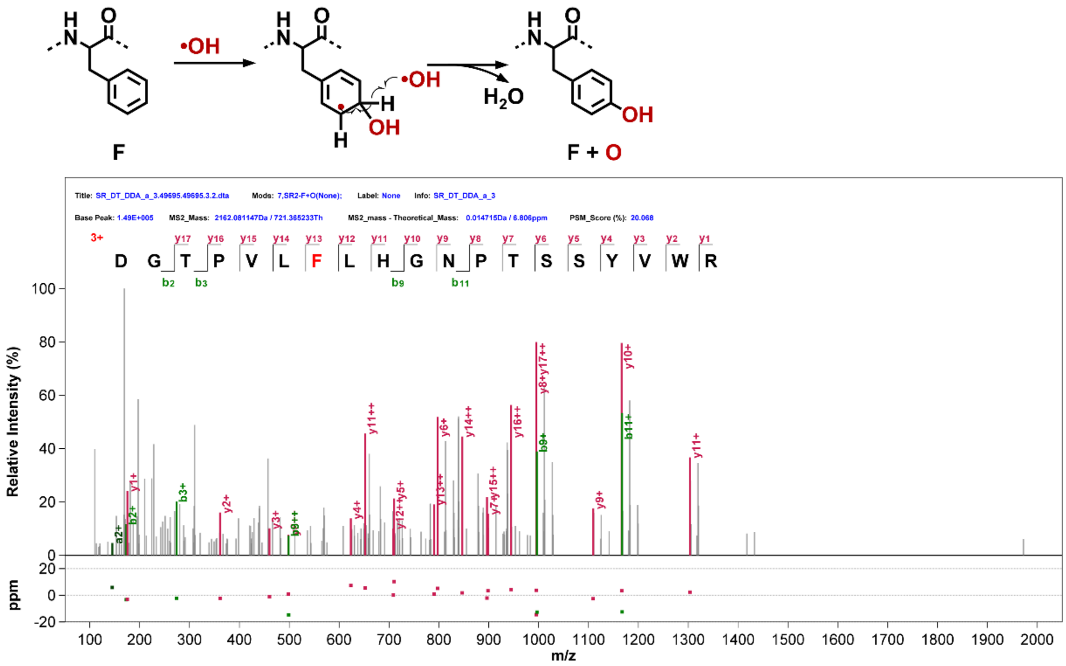

Figure S17. Proposed labeling mechanism of phenylalanine oxidized by •OH on upsFP and corresponding MS spectrum.

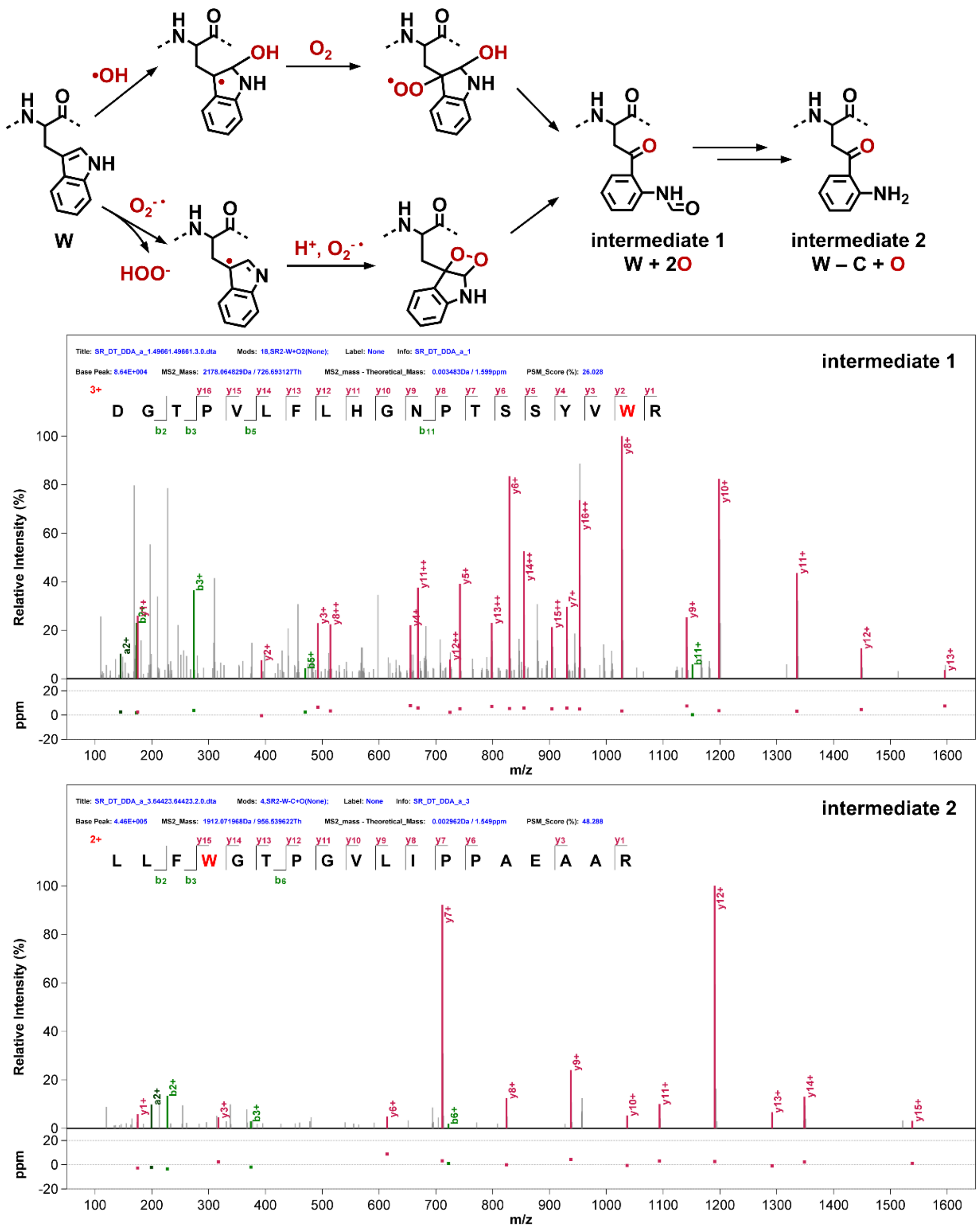

Figure S18. Proposed labeling mechanism of tryptophan oxidized by •OH and $O_2^{•-}$ on upsFP and corresponding MS spectrum.

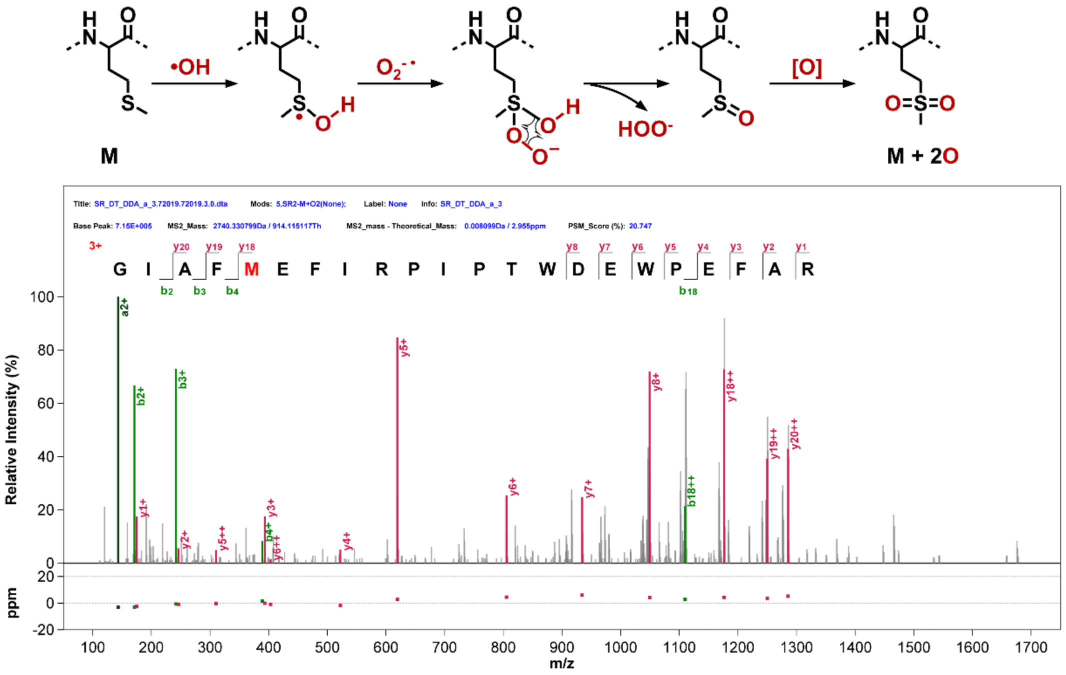

Figure S19. Proposed labeling mechanism of methionine oxidized by •OH and $O_2^{•-}$ on upsFP and corresponding MS spectrum.

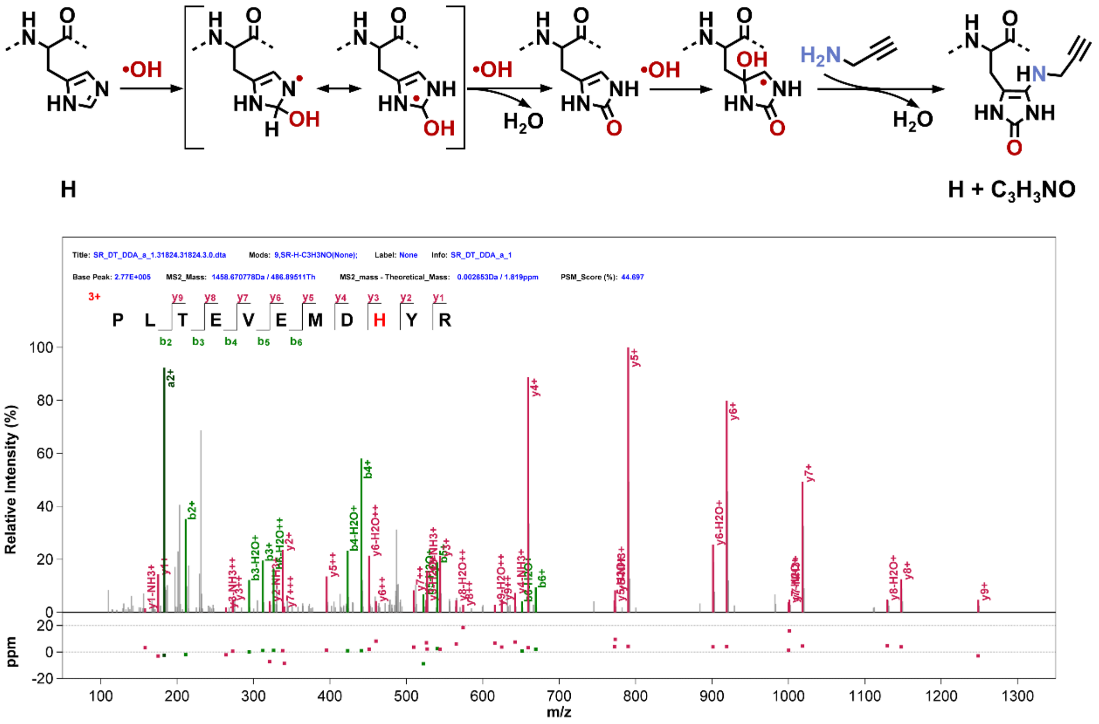

Figure S20. Proposed labeling mechanism of histidine labeled by propylamine (PA) on upsFP and corresponding MS spectrum.

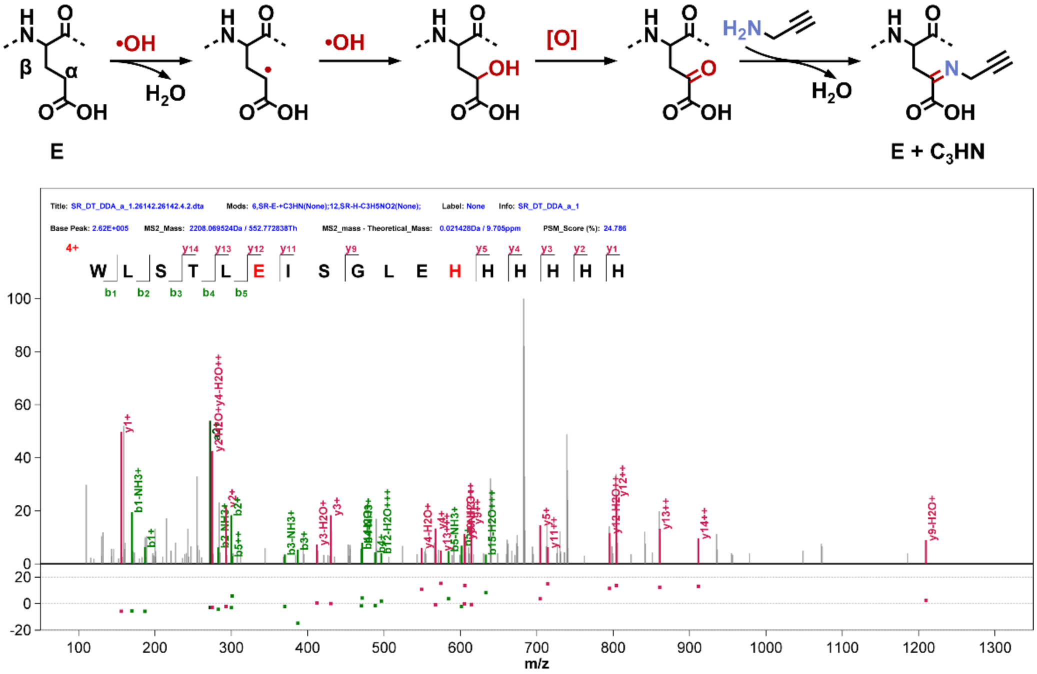

Figure S21. LC-MS/MS spectrum of glutamate acid labeled by propylamine (PA) on upsFP and proposed labeling mechanism.

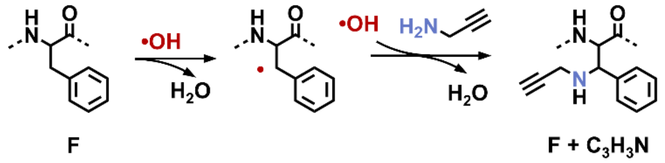

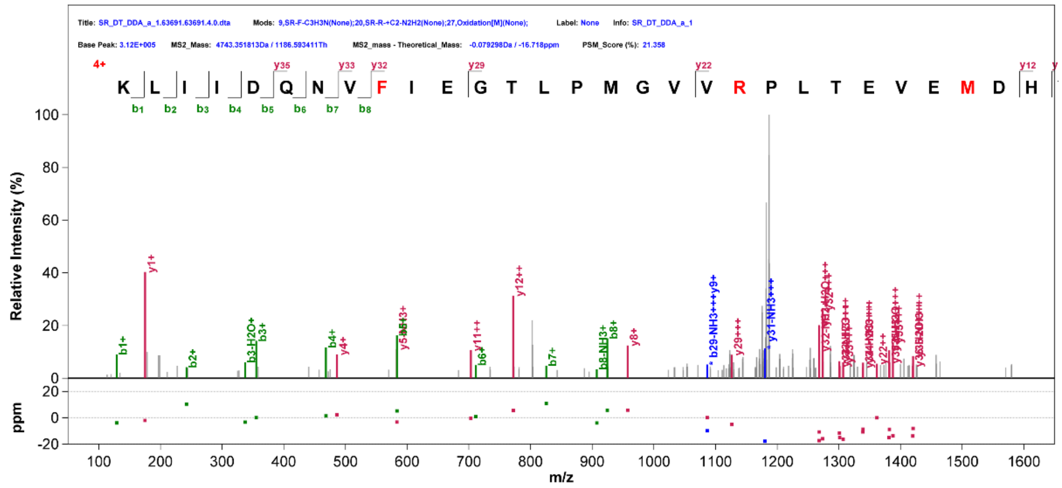

Figure S22. LC-MS/MS spectrum of phenylalanine labeled by propylamine (PA) on upsFP and proposed labeling mechanism.

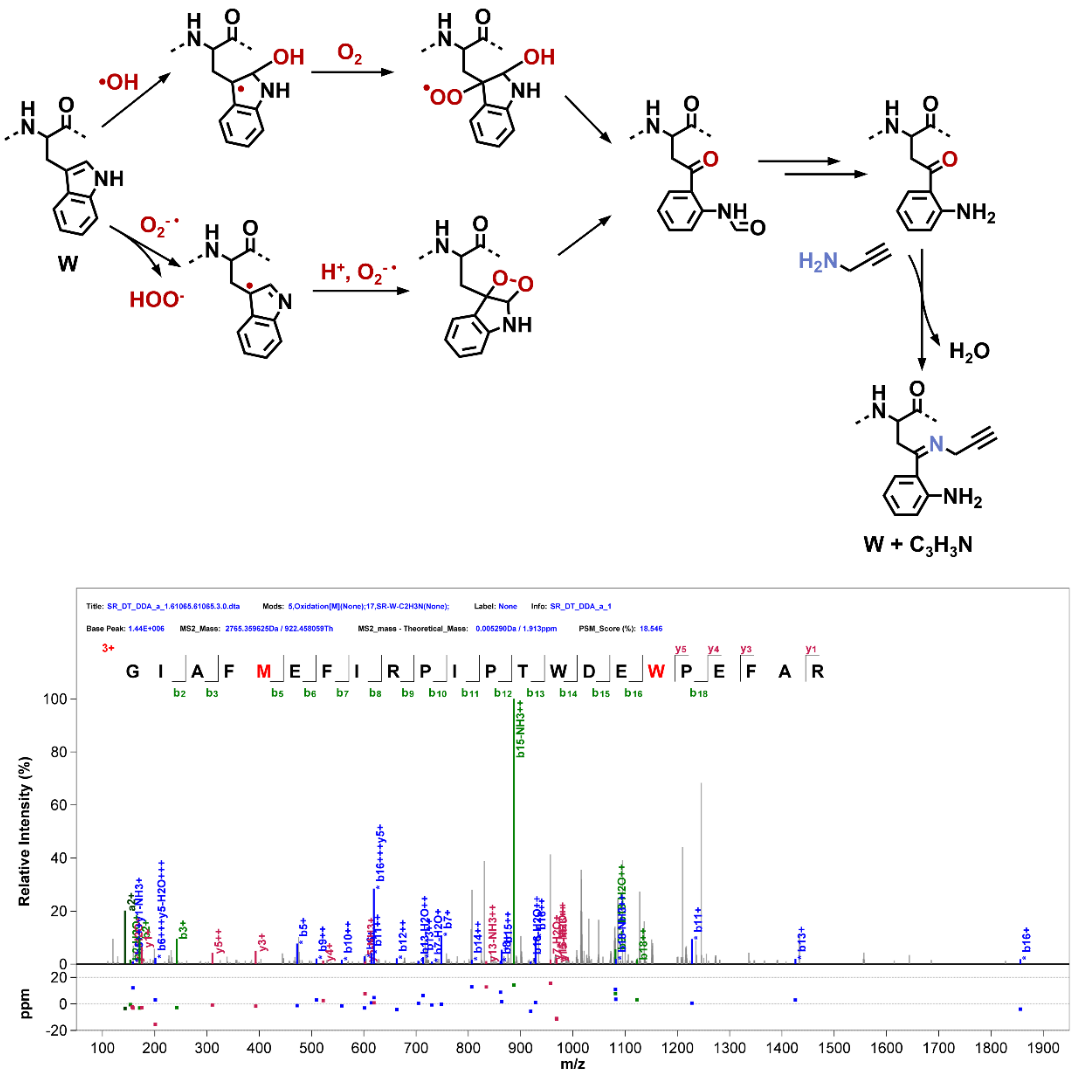

Figure S23. LC-MS/MS spectrum of tryptophan labeled with PA on upsFP and proposed labeling mechanism.

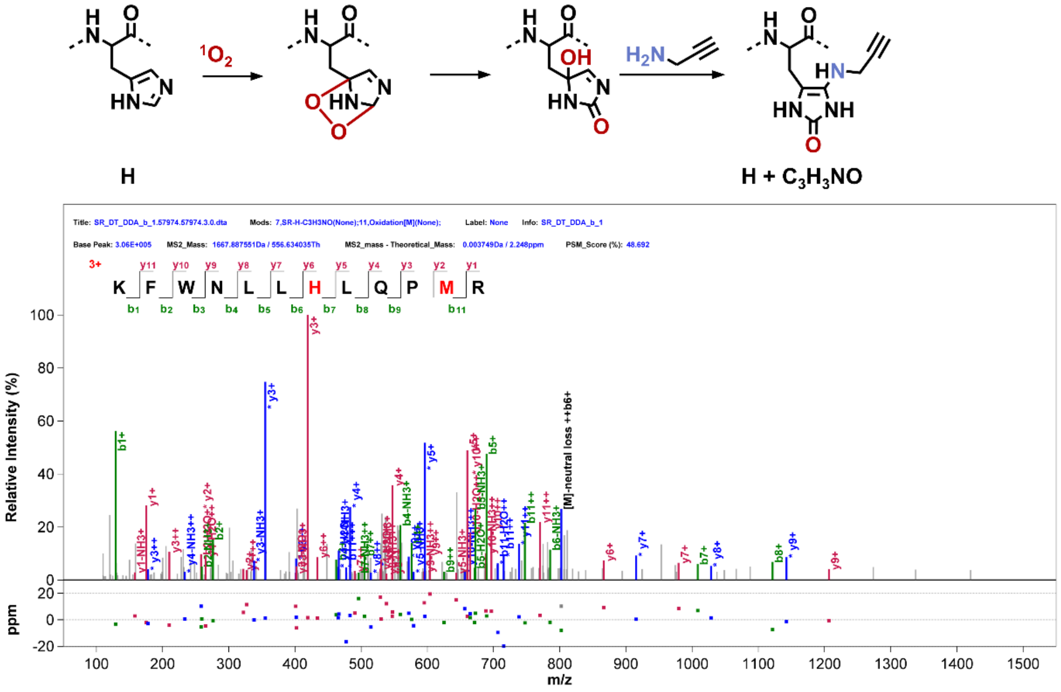

Figure S24. Proposed labeling mechanism of histidine labeled by propylamine (PA) on miniSOG and corresponding MS spectrum.

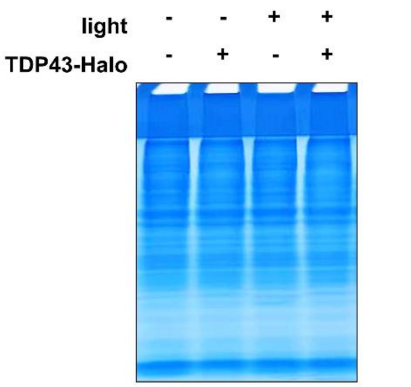

Figure S25. CBB gel corresponding to Figure 6b.

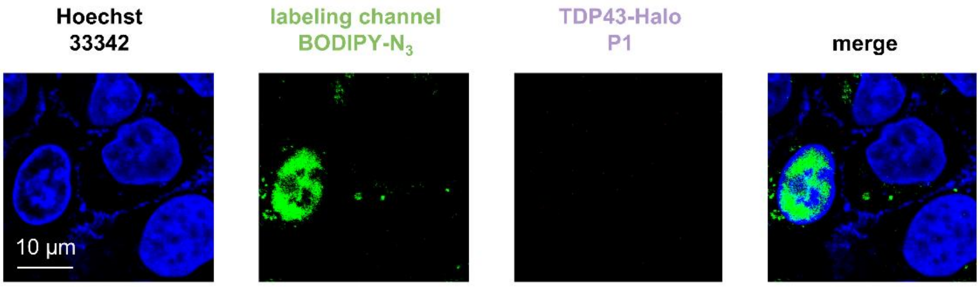

Figure S26. Proximity labeling of TDP43-Halo by 1 µM P1 without the presence of TMR Halo probe.

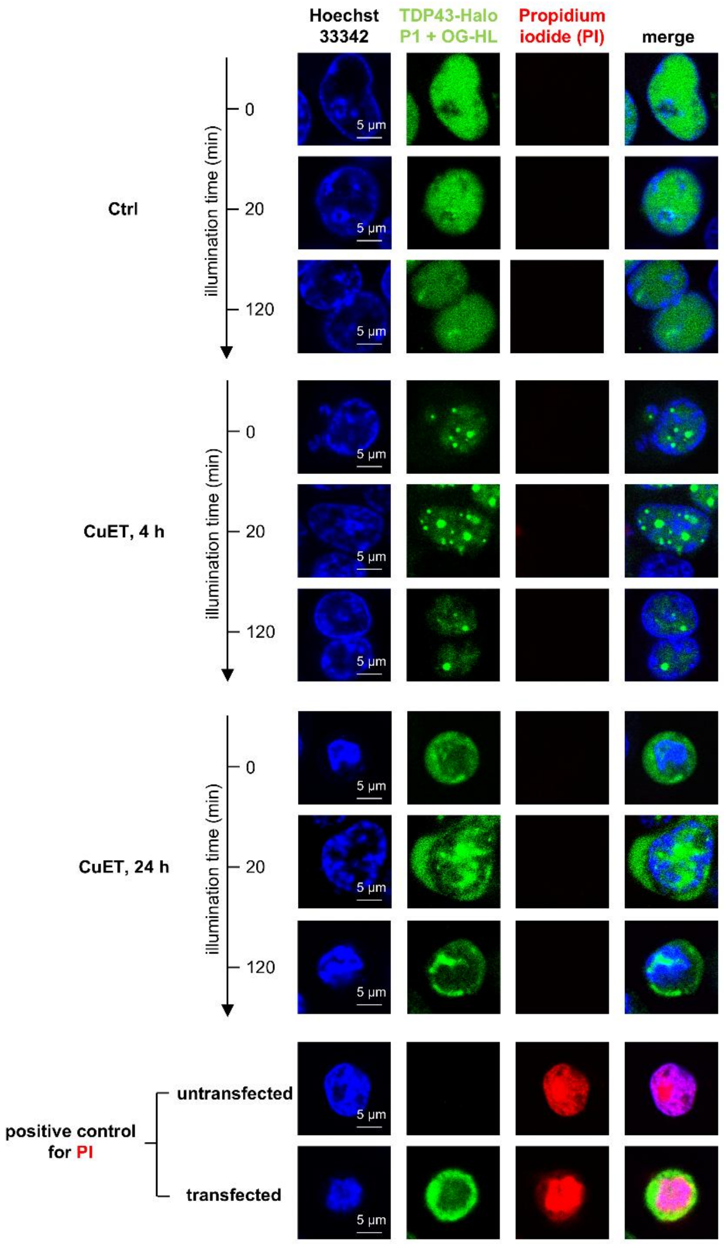

Figure S27. The morphologies of TDP43 and cell viability were not significantly compromised during the labeling period (20 min). [P1]: 1 µM; [Oregon Green-HL]: 0.1 µM for imaging. PI (0.5 µM) was used as the dead cell indicator to show that no significant cell death was triggered under the indicated experimental conditions.

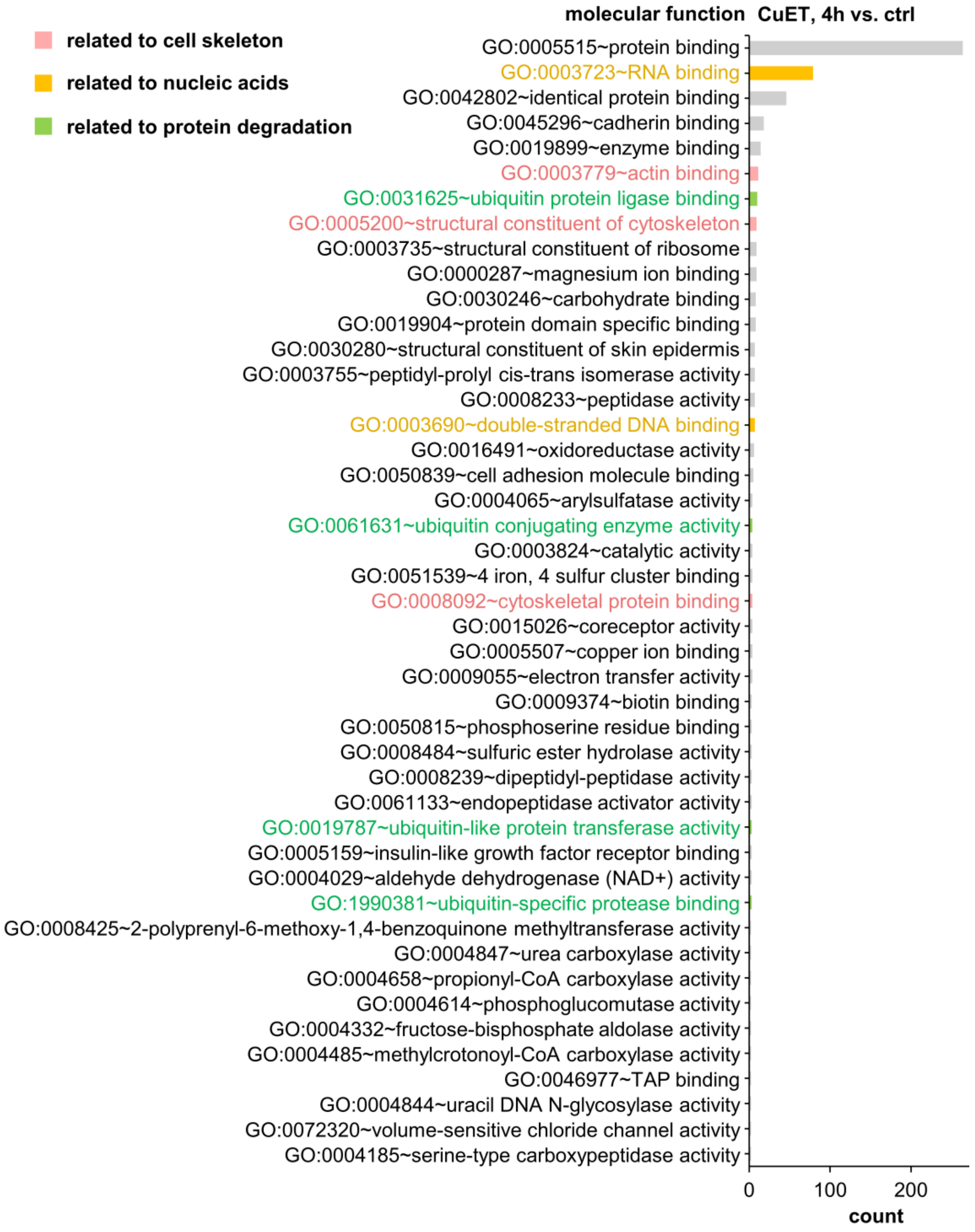

Figure S28. GO analysis of molecular functions on proteins enriched in the LLPS group (CuET, 4h) versus control group (basal, no stress).

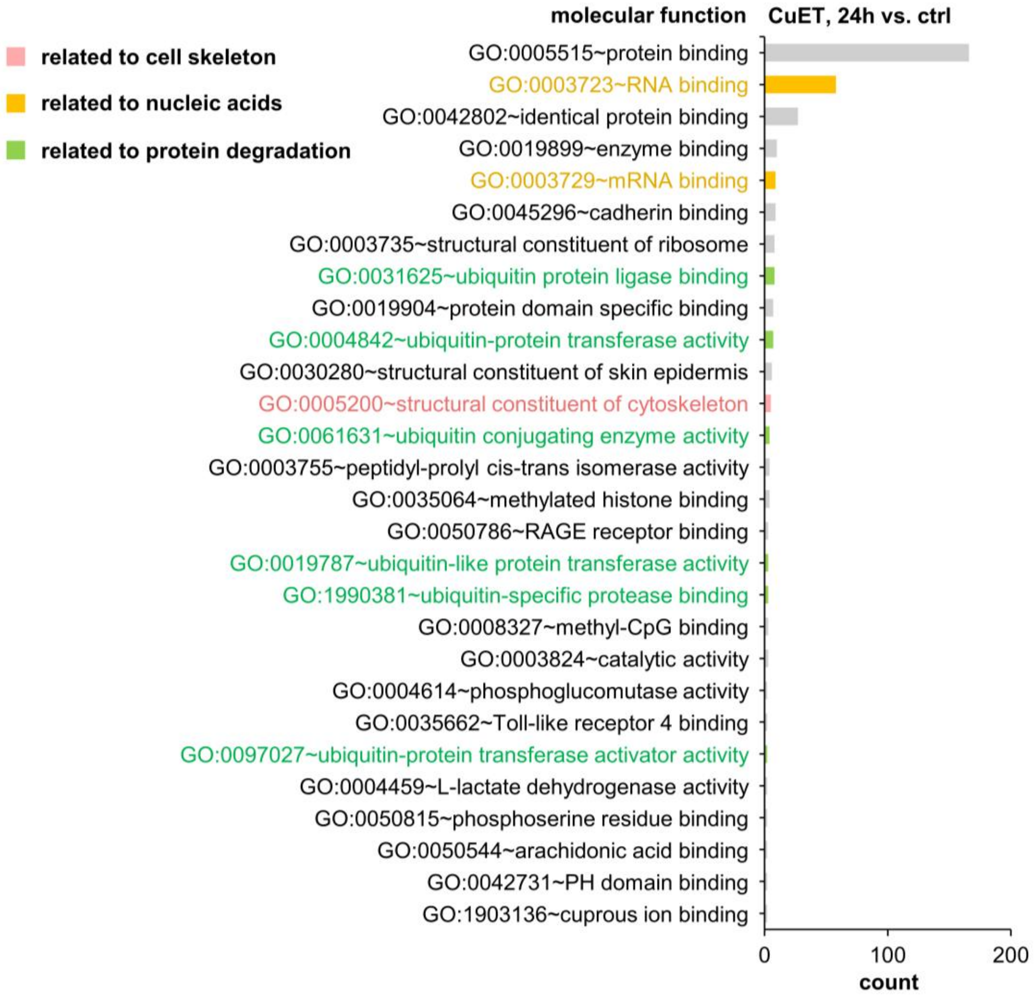

Figure S29. GO analysis of molecular function on proteins enriched in LSPS group (CuET, 24h) versus control group (basal, no stress)..

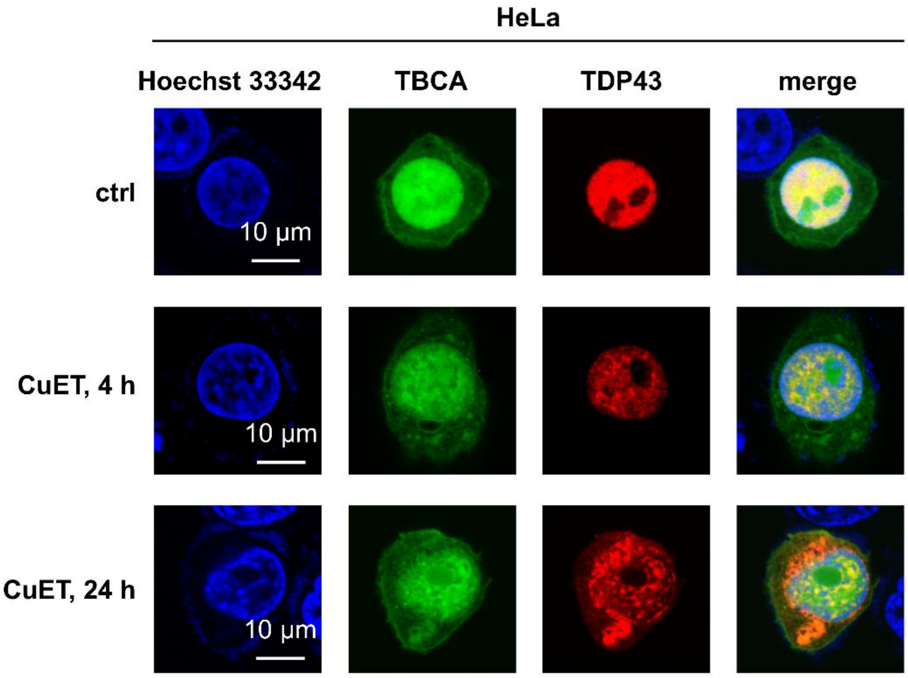

Figure S30. TBCA participated in the phase separation process of TDP43 in HeLa cells.

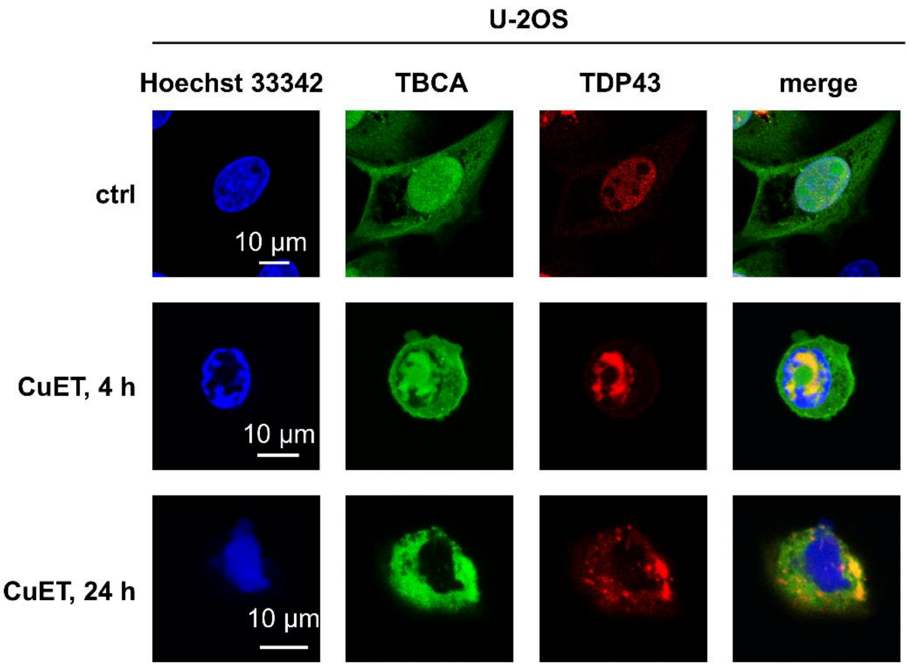

Figure S31. TBCA participated in the phase separation process of TDP43 in U-2OS cells.

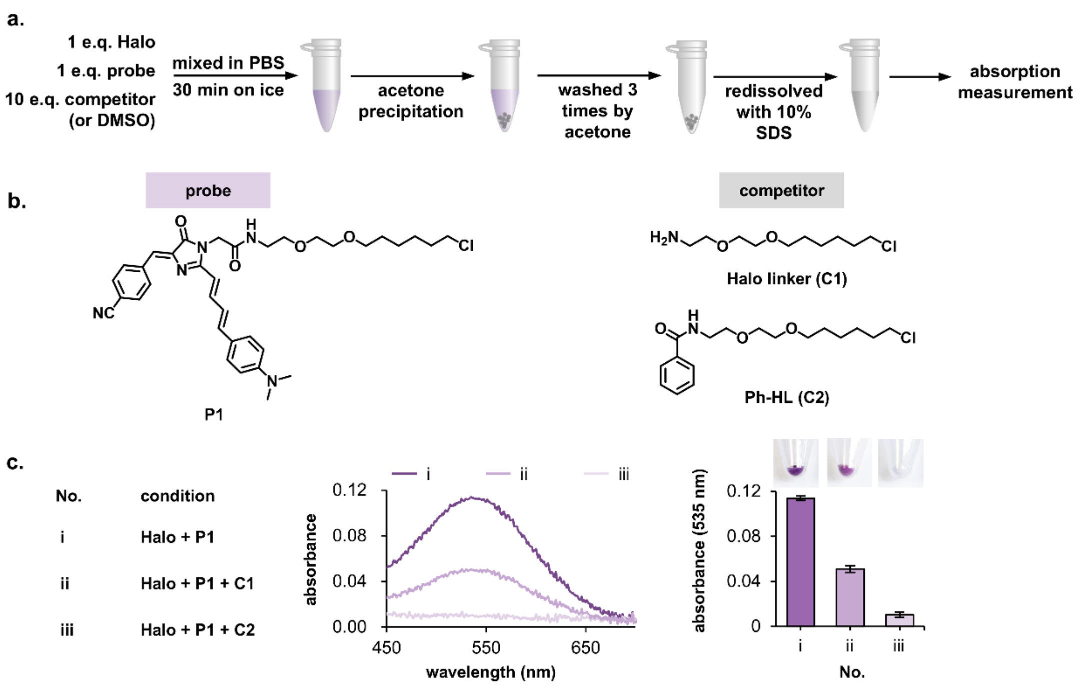

Figure S32. Ph-HL (C2) can sever as an effective competitor to P1. (a). A simplified procedure to remove the unreacted probe and competitor. (b). Structure of P1 (probe), Halo linker (competitor 1, C1) and Ph-HL (competitor 2, C2). (c). Addition a C2 efficiently prevented the covalently binding of P1 to Halo. Data are shown as the mean ± SEMs, n = 3 per group. [Halo]: 20 μM; [P1]: 20 μM; [competitor]: 200 μM. The absorption spectrum was measured by Tecan Spark Fluorescence Plate Reader in NEST^{TM} 96-Well flat bottom transparent plate.

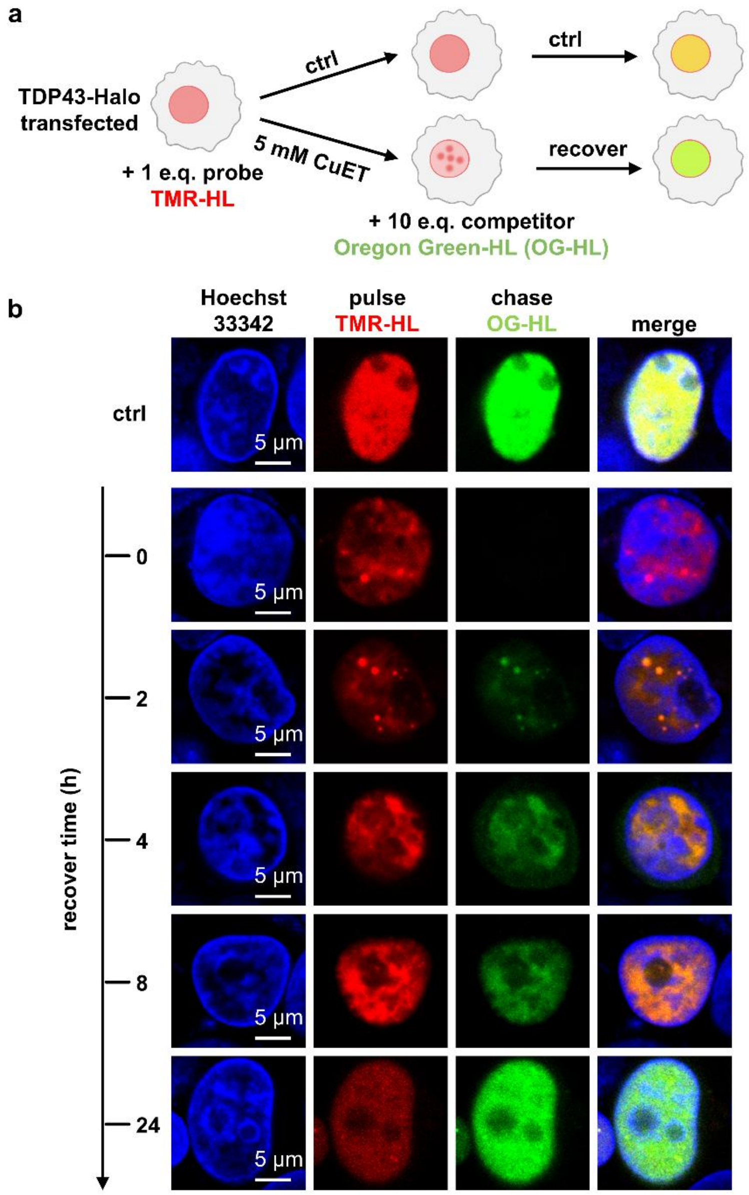

Figure S33. Potential application of upsFP for mapping the mapping the interactomes with TDP43 in stressed cells during its recovery process estimated by TMR-HL (probe) and Oregon Green-HL (competitor). (a). Scheme for pulse-chase assay to track the recovery process of phase-separated TDP43-Halo. (b) Representative images of the recovered cells at the given time points. [TMR-HL]: 0.2 μM; [Oregon Green-HL]: 2 μM.

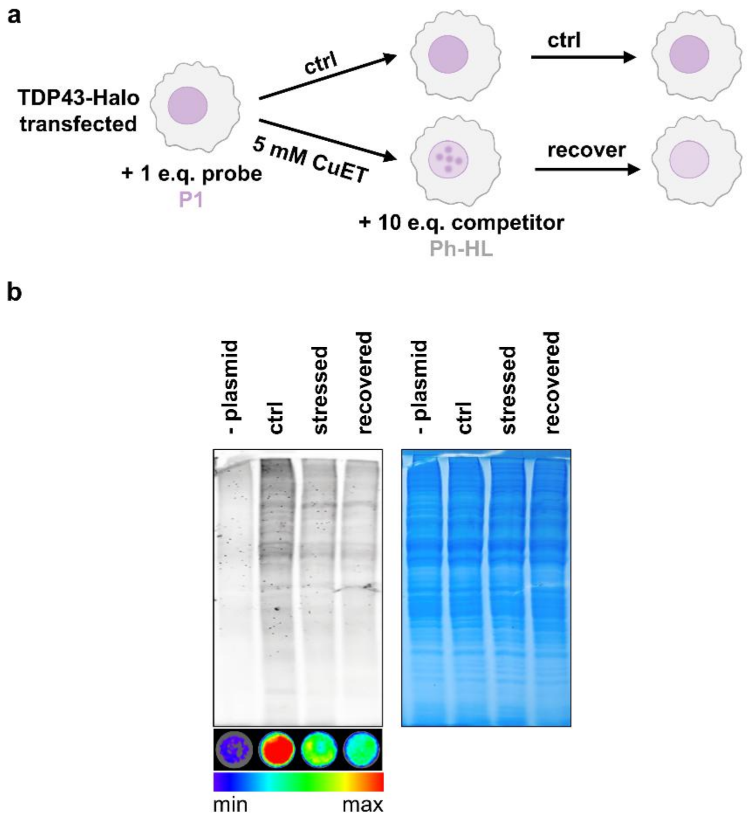

Figure S34. Fluorescent SDS-Page gel to show TDP43 interactome captured by upsFP tag upon after-stress recovery. [P1]: 1 µM; [Ph-HL]: 10 µM.

2. Unmodified Gel Images

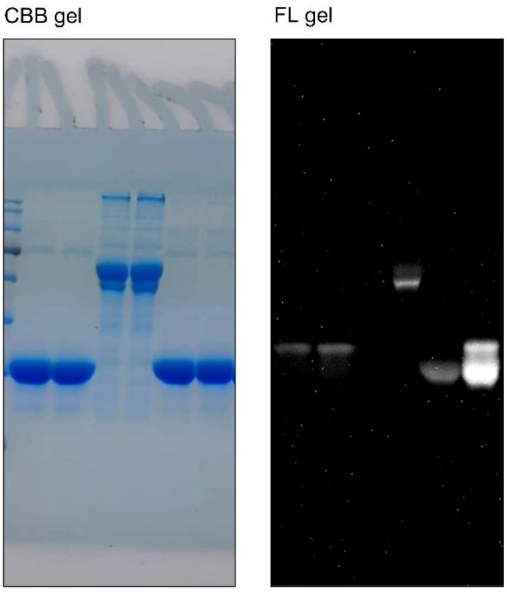

corresponding to Figure 3e

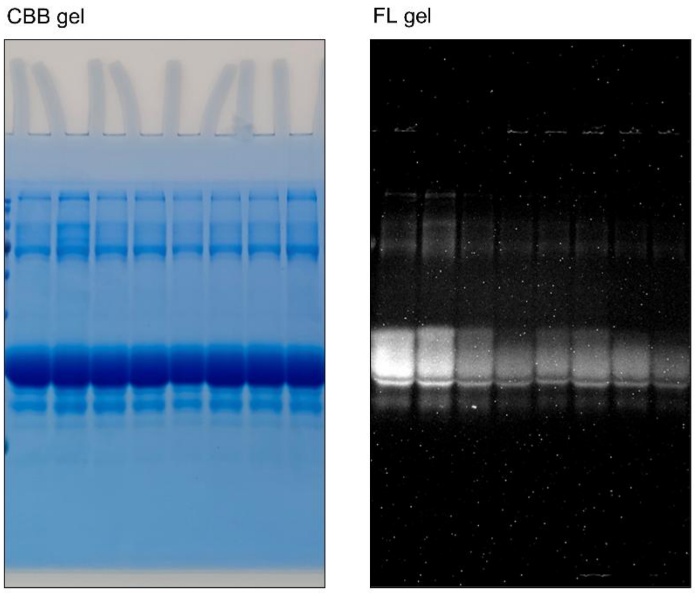

corresponding to Figure 3f

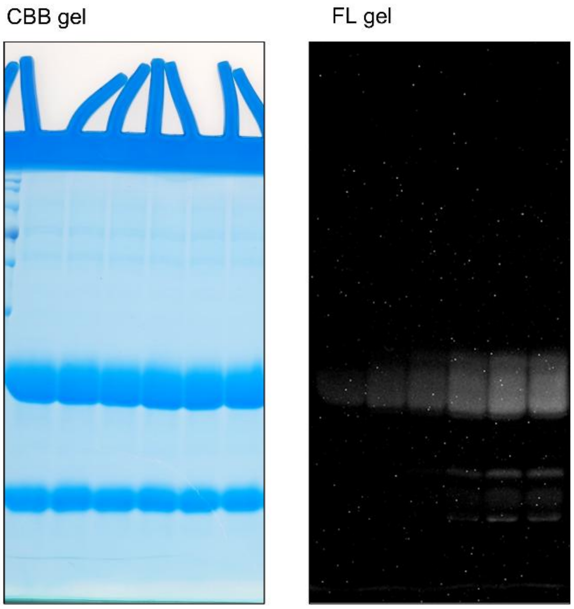

corresponding to Figure 3g

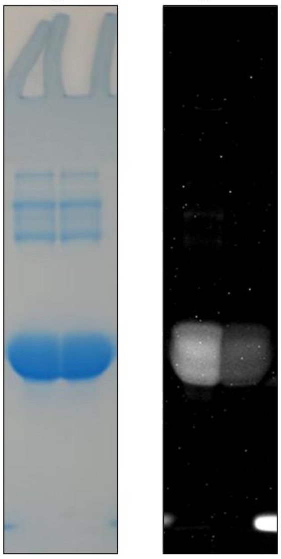

corresponding to Figure 5b

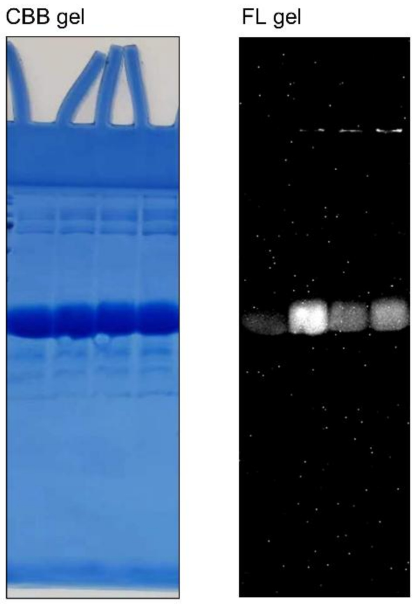

corresponding to Figure 5c

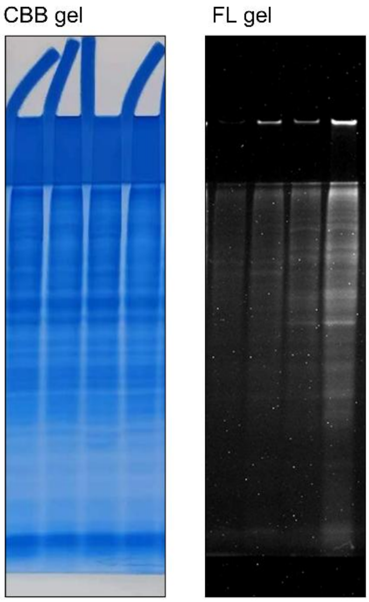

corresponding to Figure 6b

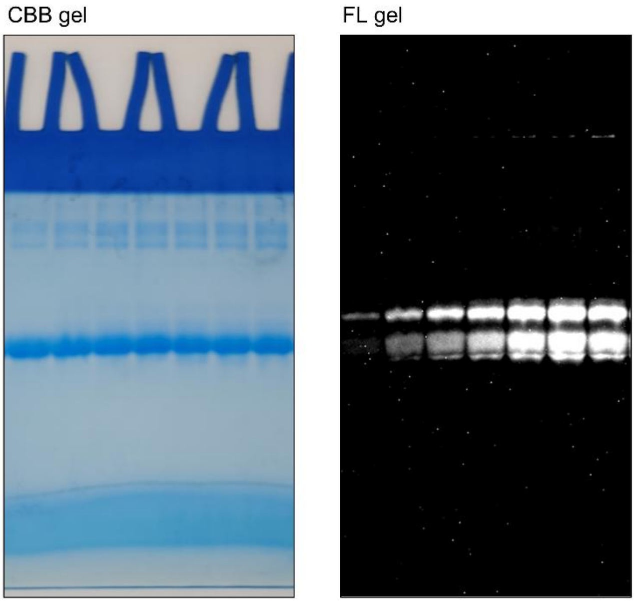

corresponding to Figure S6

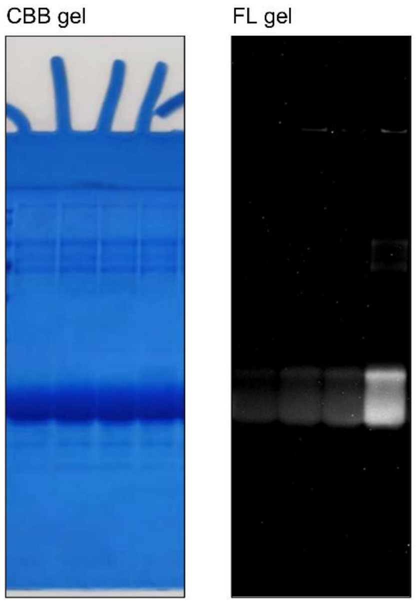

corresponding to Figure S7

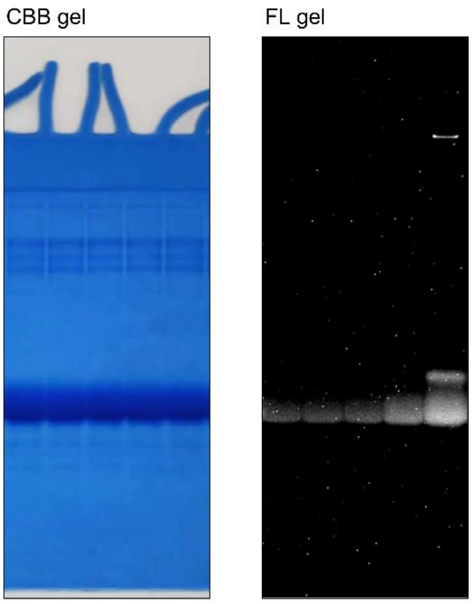

corresponding to Figure S8

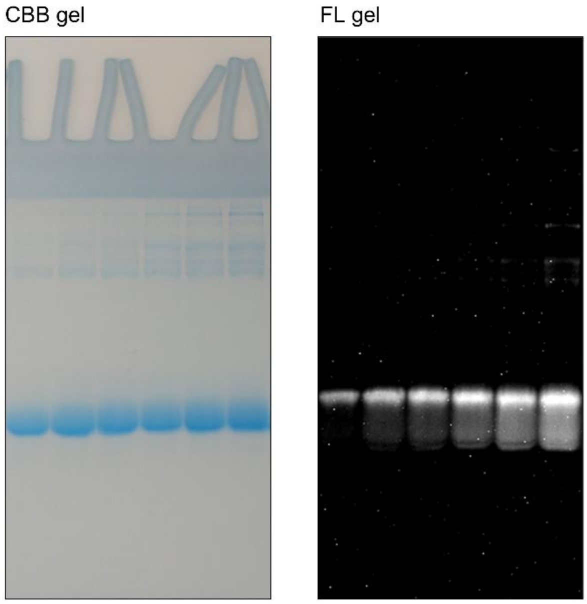

corresponding to Figure S9

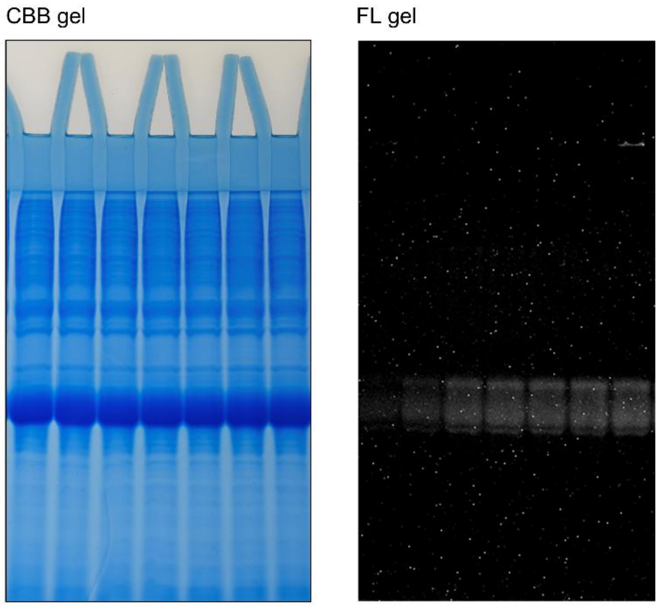

corresponding to Figure S10

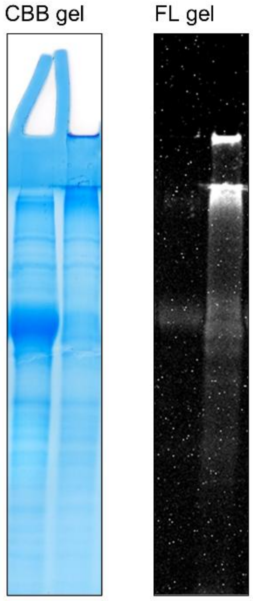

corresponding to Figure S11

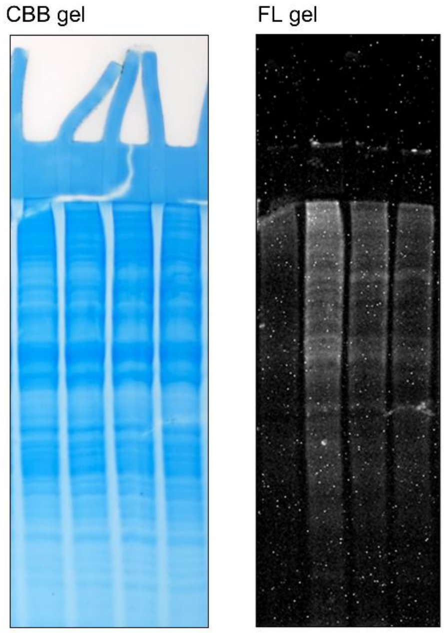

corresponding to Figure S34b

3. Experimental Procedures

3.1 Plasmids construction and protein purification.

Genes of *E. coli* HaloTag, KillerRed, KillerRed-Halo, miniSOG[1] and wild type dihydrofolate reductase (WT-DHFR) were all optimized and synthesized by GenScript, Nanjing, China, and sub-cloned into pET-29b (+) vectors. They were cloned with His-tag at their C-termini for easy purification purpose.

TDP43-Halo was a gift from prof. Zhang lab and sub-cloned into pHTC vectors.

TBCA-EGFP was synthesized by GenScript, Nanjing, China, and sub-cloned into pcDNA3.1(+) vectors.

As previously reported. Briefly, for WT-DHFR, HaloTag and miniSOG: the plasmids were transformed into BL21(DE3) E. coli cells. Cells were then grown in Luria Bertani (LB) media and incubated at 37 °C under continuous shaking until the OD600 was in the range of 0.6-0.8. Protein synthesis was subsequently induced by isopropyl β-Dthiogalactoside (IPTG) at various conditions:

Halo: 37 °C for 4 h;
KillerRed: 22 °C for 12 h;
KillerRed-Halo: 22 °C for 12 h;
miniSOG: 18 °C for 12 h;
WT-DHFR: 37 °C for 4 h.

Then, cells re-suspended in buffer A (50.0 mM Tris·HCl, 100.0 mM NaCl, pH = 8.00) and lysed by sonication at 4 °C. The supernatant obtained by centrifugation (12,000 rpm, 30 min) was loaded into a 10 mL Ni-NTA column and then was eluted by buffer A (50.0 mM Tris·HCl, 100.0 mM NaCl, pH = 8.00) with gradient increasing of buffer B (50.0 mM Tris·HCl, 100.0 mM NaCl, 500.0 mM imidazole, pH = 8.00). Ni-NTA column pre-purified proteins were further purified by using a 120 mL Superdex 200 size-exclusion column in phosphate buffer (10.0 mM sodium phosphates, 100.0 mM KCl, 1.0 mM EDTA, pH = 7.40). The protein containing fractions were identified by SDS-PAGE electrophoresis gel analysis, then pooled, and concentrated. No significant impurity was identified and the purity was estimated to be at least 98% based on SDS-PAGE gel.

3.2 ROS quantum yield definition.

The overall reactive oxygen species (ROS) production from the probes under white light illumination was determined using the 2,7-dichlorodihydrofluorescein (DCFH) assay.

$$\Phi_{ROS} = K_{probe}/A_{probe}$$

Where K_{probe} was the oxidation rate constant of DCFH by the ROS produced by probes versus irradiation time. A_{probe} was the absorption ability of probes.

3.3 General procedure of photocatalysis labeling in vitro.

A solution of 30 μM HaloTag and 30 μM P1 was mixed in 300 μL of PBS and incubated at room temperature for 30 min to prepare upsFP. Subsequently, 10 mM propargylamine (PA) was introduced. Following illumination with white LEDs (10 mW•cm^{-2}) for 20 min, a 4-fold excess of precooled acetone was added to the sample, which was then incubated at -20 °C overnight. Next, the protein precipitates were redissolved in 200 μL of 10% SDS, and 50 μL of click reagent was added to each sample (consisting of 1300 μL of 1.7 mM TBTA, 440 μL of 50 mM $CuSO_4$, 440 μL of 50 mM TCEP, and 12 μL of 50 mM TMR-N_3). The sample was shaken at room temperature for 1 h. Subsequently, a 4-fold excess of precooled acetone was added to the sample, which was then incubated at -20 °C overnight. The sample was then washed three times with precooled acetone to completely remove excess click reagents.

Ultimately, the sample was redissolved in 10% SDS, thus rendering it suitable for SDS-PAGE and dots experiment.

3.4 Procedure for cell culture, transfection of cells, and cell imaging experiments.

HEK293T cells were seeded on 35 mm confocal culture dishes and transiently transfected when the cell density reached 70%. In 200 μL opti-MEM medium, 3 μL of X-tremegene 9 DNA transfection reagent (Roche) was added. The plasmid of TDP43-Halo (1 μg) was then introduced to the solution. The mixture was fully mixed at room temperature for 25 min in darkness. Subsequently, it was then dripped into the cell medium and expressed for 48 h. 0.5 μM TMR Halo probe were added. Next, the transfected cells were treated with 5 μM CuET for 4h or 24 h. Hoechst 33342 (1000×) was added to the cell medium 30 min before imaging. Images were collected using Olympus FV1000MPE.

3.5 General procedure of photocatalysis labeling in vivo (for imaging).

After transfection and incubation with 1 μM P1 (with or without 0.1 μM TMR-Halo probe) for 48 h on 35 mm confocal culture dishes, HEK293T cells were washed with PBS for three times, and then fixed with 4% paraformaldehyde fix solution for 30 min in darkness. Next, the fixed cells were washed with HBS for three times. Subsequently, they were incubated with propargylamine (PA, 10 mM in HBS) in the dark for 20 min, followed by white light illumination for 20 min (10 mW•cm^{-2}). The cells were washed by methanol and PBS for three times. 500 μL PBS was added to keep wet, and 50 μL of click reagent (consisting 1300 μL of 1.7 mM TBTA, 440 μL of 50 mM $CuSO_4$, 440 μL of 50 mM TCEP, and 12 μL of 50 mM BODIPY-N_3) were added. The sample was kept at room temperature for 1 h. Next, the cells were washed by methanol and PBS for three times. Finally, Hoechst 33342 (1000×) was added before imaging. Images were collected using Olympus FV1000MPE.

3.6 Cell Sample Preparation for Proteomic Analysis.

HEK293T cells were seeded and cultured in 10 cm culture dishes and then transfected with TDP43-Halo for 48 h. Meanwhile, 1 μM P1 was added to each group. In the control group, HEK293T cells were treated with P1 for 48 h. After treatment, the cells were washed by fresh medium and HBS for three times. Then they were incubated with propargylamine (PA, 10 mM in HBS) in the dark for 20 min, followed by white light illumination for 20 min (10 mW•cm^{-2}). Finally, the cells were harvested by centrifugation and stored at -80 °C. For the click reaction of the proteins, cells (about 1*10^8) from the experimental group and control group were resuspended in 10 mL 1% (w/v) SDS/PBS by sonication at 80 W on ice. Then, 1 mL click regents (consisting 1300 μL of 1.7 mM TBTA, 440 μL of 50 mM $CuSO_4$, 440 μL 50 mM of TCEP, and 12 μL of 50 mM biotin-N_3) was added in series to initiate the click reaction. The sample was kept at room temperature for 1 h with constant shaking. A 4-fold excess of precooled acetone was added to the sample followed by incubation at -20 °C overnight and washed three times with precooled acetone to completely remove the excess click regents.

Next, the labeled proteins dissolved in 8.0 M urea and diluted to 1.0 M urea with 50.0 mM NH_4HCO_3. Then the sample was incubated with streptavidin agarose resin (17511301, cytiva) 4℃ overnight end-by-end. The beads were then washed twice with 8.0 M urea and 2.0 M NaCl solution, and 50mM NH4HCO3 solution once, and resuspended in 0.3 M urea. Next, 10.0 mM dithiothreitol was added and incubated at room temperature for 1 h. Subsequently, 20.0 mM iodoacetamide aqueous solution was added and incubated at room temperature for 30 min in the dark. After centrifugation, beads were washed twice with NH_4HCO_3 solution (50.0 mM) followed by the treatment of trypsin at 37 °C for 12 h at an enzyme to protein ratio of 1:30 (w/w), and the released peptides in supernatant were collected by centrifugation. Then beads were washed twice with 0.1% formic acid (FA), and the collected supernatant was merged with the above released peptides for component identification. Thereafter, the peptides binding on beads were released by eluting with 5-fold volume of 10% formic acid (FA) for three times, and the collected peptides in supernatant were merged for site identification.

For the three experimental groups (CuET treatment at 0, 4, and 24 hours), the digested peptides were partially taken (one-third) and pooled to form a single sample, which was then fractionated for library construction using data-dependent acquisition (DDA) mode. Specifically, homemade C18 tips (5 μm, 100 Å; Durashell) were used as separation columns, with solvent A (water with ammonia added until the pH reached 10.0) and solvent B (acetonitrile with the same volume of ammonia added as in solvent A) for washing. The peptides were separated into 9 eluates (6%, 9%, 12%, 15%, 18%, 21%, 25%, 30%, 80% B), with 6% and 25% combined for fraction 1, 9% and 30% for fraction 2, 12% and 80% for fraction 3, and 15%, 18%, and 21% forming fractions 4, 5, and 6, respectively. The remaining samples from each group were desalted and used for DIA analysis, with each sample analyzed in triplicate.

3.7 Nano-LC-MS/MS Analysis.

The dried tryptic digestion samples were initially re-dissolved in a solution containing 0.1% formic acid (FA). These samples were later analyzed using an Easy-nano LC 1200 system coupled to an Orbitrap Fusion Lumos mass spectrometer (Thermo Fisher Scientific). For the mass spectrometry (MS) analysis, two mobile phases were employed. Mobile phase A consisted of 0.1% FA in HPLC H2O, while mobile phase B consisted of acetonitrile with 20% water and 0.1% formic acid (FA). Separation of peptides using nano-RPLC (reversed-phase liquid chromatography) was carried out using a C18 capillary column with dimensions of 150 μm i.d. × 300 mm, packed with C18 silica particles (1.9 μm, 120 Å) from Dr. Maisch GmbH, Beim Brueckle, Germany. The column was heated to 55 °C, and peptide separation was performed at a flow rate of 600 nL·min-1. The following gradient was used for the separation: starting from 3% B (mobile phase B) and increasing to 14% B over 30 min, followed by a gradient from 14% to 36% B in the next 65 min, then an increase from 36% to 46%

B over 20 mins, further increasing to 95% B in 1 min, and maintaining 95% B for 4 min. The mass spectrometer was operated in positive ion mode.

DDA mode: The MS1 scans were performed with a resolution of 60,000 (at 200 m/z) from m/z 350 to 1500. The MS2 scans were conducted with a resolution of 15,000, and the first mass was set at 110. The maximum injection times for MS1 and MS2 were 50 and 30 ms. In each full MS scan, the most intense ions with charge states ranging from 2 to 7 were selected for monomer oxidation sequencing. The selection was performed in a cycle time of 1 second, using an isolation window of 1.6 m/z. The fragmentation of precursor ions was achieved using the data-dependent HCD (higher-energy collisional dissociation) mode, with a normalized collision energy set to 30%.

DIA mode: MS data acquisition in DIA mode was performed on the Orbitrap Fusion Lumos Tribrid using 28 variable windows covering a mass range of 350–1350 m/z. The resolution was set to 60,000 for MS1 and 30,000 for MS2. The AGC was set to 4e5 for MS1 and 5e5 for MS2, with a maximum injection time of 100 ms for MS1 and 50 ms for MS2. The HCD collision energy was set to 30%.

3.8 MS data analysis.

Spectral library generation in FragPipe. We used the FragPipe computational platform (version 15) with MSFragger (version 3.2), Philosopher (version 3.4.13), and EasyPQP (version 0.1.9) to build spectral libraries. Peptide identification from tandem mass spectra (MS/MS) was performed using the MSFragger search engine, with DDA raw files as input. The protein sequence databases used were H. sapiens (UP000005640) from UniProt (reviewed sequences only, downloaded on February 16, 2023) and common contaminant proteins, totaling 20,375 (H. sapiens) sequences. Reversed protein sequences were appended to the original databases as decoys. For the MSFragger analysis, both precursor and fragment mass tolerances were set to 20 ppm. Up to two missed trypsin cleavages were allowed. Peptide length was set from 7 to 50 amino acids, and peptide mass was set from 500 to 5000 Da. Oxidation of methionine and acetylation of protein N-termini were set as variable modifications, and carbamidomethylation of cysteine was set as a fixed modification. The maximum number of variable modifications per peptide was set to 3.

DIA data analysis in DIA-NN For the analysis of DIA data, the search parameters in DIA-NN (version 1.8.1) were set as follows: precursor FDR 1%; mass accuracy at MS1 and MS2 set to 5 ppm and 15 ppm, respectively; scan window set to 0; isotopologues and match-between-runs (MBR) enabled; protein inference at the gene level; heuristic protein inference enabled; quantification strategy set to Robust LC (high precision); neural network classifier in double-pass mode; cross-run normalization turned off. The universal library was used, and protein re-annotation was performed. In the library-based mode, the speclib file generated from FragPipe was used as the spectral library. The main search settings were as follows: Trypsin/P with a maximum of 2 missed cleavages; protein N-terminal methionine excision enabled; oxidation of methionine and acetylation of protein N-termini set as variable modifications; carbamidomethylation of cysteine set as a fixed modification; peptide length from 7 to 30 amino acids; precursor charge from 1 to 4; precursor m/z from 350 to 1350; fragment m/z from 200 to 1800. The search results were further filtered with a q-value < 0.01 for protein groups at the library level.

The mass spectrometry data and searching results have been deposited in the ProteomeXchange Consortium repository of open source under the dataset identifier PXD055096. The data can be accessed through the following link: https://proteomecentral.proteomexchange.org/cgi/GetDataset?ID=PXD055096.

3.9 Calculation method.

The initial molecular structure selected in this article was constructed using GaussView6.0 software. The compound used in this study adopts the B3LYP-D3 method of density functional theory (DFT), and further optimizes the molecular structure to convergence using the 6-31G (d) basis set. The solvation model is selected as SMD, and the solvent is selected as water. The calculation of excited states is carried out using time-dependent density functional theory (TD-DFT) at the same fundamental level. All the above calculations were completed on the Gaussian 16 program.

3.10 LC-MS/MS Analysis of upsFP.

A solution of 60 μM HaloTag and 30 μM P1 was prepared in 500 μL of PBS and incubated at room temperature for 30 min. Subsequently, a 4-fold excess of precooled acetone was added to the sample, which was then incubated at -20 °C overnight. After centrifugation, the protein precipitates were redissolved in 80 μL of 8 M urea and diluted to 500 μL with dd water.

UPLC: Ultimate 3000 (Thermo Fisher Scientific, USA)

Column：ACQUITY UPLC Protein BEH C4 Column (300Å, 1.7 µm, 2.1 mm×50 mm);
Mobile phase: A: 0.1% formic acid in water; B: 0.1% formic acid in acetonitrile.
Total flow rate: 0.300 mL/min
LC linear gradient: from 5% to 100% B for 10 min, from 100% to 100% B for 3 min, from 100% to 5% B for 1 min, from 5% to 5% B for 1 min.

Mass spectrometry: AB SCIEX TripleTOF 5600 Mass Spectrometer.

4. Synthesis and Characterizations

All reagents are commercial grade unless otherwise stated. Reactions were carried out in Synthware® round bottom flask and monitored via thin layer chromatography. Products were purified with flash column chromatography (200-300 mesh). NMR spectra were recorded on Bruker 700 MHz spectrometers and were calibrated using residual solvent as an internal reference (DMSO-*d*6: 2.50 ppm for ^{1}H NMR and 39.52 ppm for ^{13}C NMR). HRMS data were obtained with Agilent 6540 Accurate-MS spectrometer (Q-TOF).

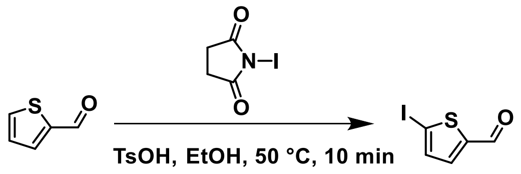

5-iodothiophene-2-carbaldehyde

***Synthesis of 5-iodothiophene-2-carbaldehyde*[2]:** Thiophene-2-carbaldehyde (11.22 g, 100 mmol) was dissolved in 200 mL EtOH and heated to 50 °C. Add N-iodosuccinimide (24.75 g, 110 mmol) and p-toluenesulfonic acid (1.72 g, 0.1 mmol). The resulting mixture was stirred at 50 °C in the dark for another 10 min. Add 100 mL of 1 M aqueous HCl and 100 mL EtOAc to the mixture. The organic layer was extracted with EtOAc, washed with saturated aqueous $Na_2S_2O_3$ and Na_2CO_3 solutions, dried over anhydrous Na_2SO_4 and filtered. All solvent was removed under vacuum to obtain pale white to dark brown crystals. The product was stored at -20 °C with inserted Ar atmosphere. (yield: 95%) ^{1}H-NMR (700 MHz, $(CD_3)_2SO$) δ 9.80 (s, 1H), 7.71 (d, J = 7.0 Hz, 1H), 7.60 (d, J = 7.0 Hz, 1H) ppm. HRMS (m/z) Anal. Calc'd for C_5H_3IOS $(M+H)^+$: 238.9022, Found $(M+H)^+$: 238.9015.

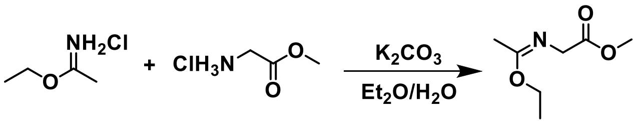

imidate intermediate

***Synthesis of imidate intermediate*:** Ethyl acetimidate hydrochloride (37.0 g, 300 mmol), glycine methyl ester hydrochloride (38.0 g, 300 mmol) and potassium carbonate (42.0 g, 300 mmol) were mixed in 500 mL diethyl ether. 100 mL dd water was introduced to the mixture stepwise under vigorously shaking in 15 min. Then the organic layer was dried over anhydrous Na_2SO_4 and filtered. The solvent was removed under reduced pressure. This product was freshly prepared before used.

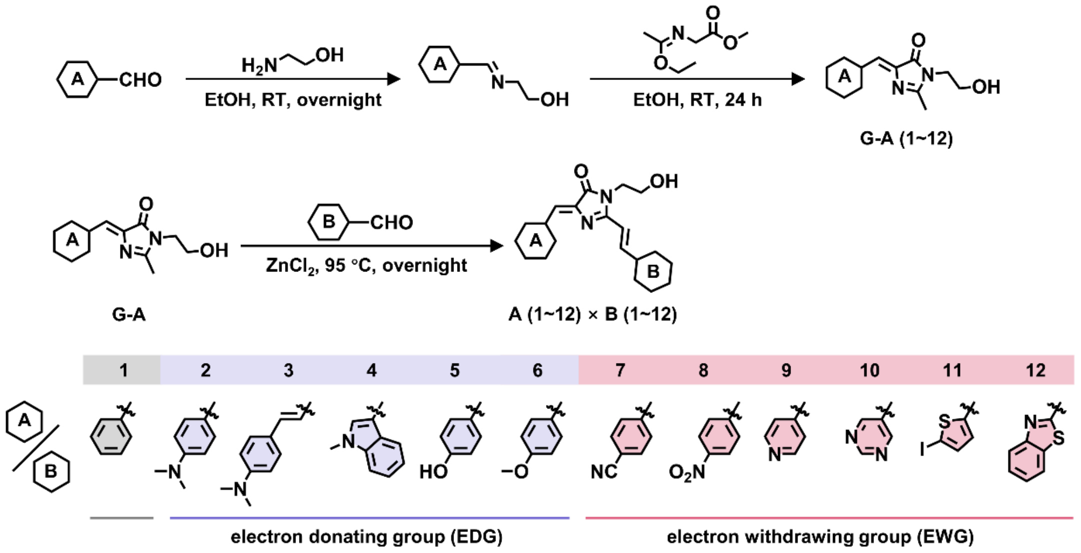

***Synthesis of G-A1~G-A12*[3]:** The aldehyde (100 mmol) and ethanolamine (110 mmol) were mixed in 500 mL round bottom with 150 ~ 200 mL ethanol under protection of Ar atmosphere. The reaction mixture was kept stirring at room temperature overnight, and then the freshly prepared imidate intermediate (100 mmol) was introduced to the reaction mixture. After stirred at room temperature for another 24 h, the product crystallized out of the reaction mixture. The reaction mixture was filtered, and the residue solid was washed with cold ethanol. Then the product was dried under vacuum. All of these twelve products were stored at 2 ~ 8 °C protected from light.

Note: G-A1 cannot crystallized out of the reaction mixture, therefore, when the reaction was completed, the solution was concentrated under reduced pressure. Then the mixture was redissolved in 200 mL DCM, washed by dd H_2O and brine, dried over anhydrous Na_2SO_4 and filtered. The solvent was removed under reduced pressure. The crude residual was purified by column chromatography.

The yield and characterization of these products were listed below.

G-A1: 7.8 g, 34%. ^{1}H-NMR (700 MHz, $(CD_3)_2SO$) δ 8.21 (d, *J* = 7.6 Hz, 2H), 7.46 (m, 2H), 7.41 (m, 1H), 6.96 (s, 1H), 4.99 (t, *J* = 5.5 Hz, 1H), 3.64 (t, *J* = 5.5 Hz, 2H), 3.55 (m, 2H), 2.40 (s, 3H) ppm. HRMS (m/z) Anal. Calc'd for $C_{13}H_{14}N_2O_2$ (M+H) $^+$: 231.1128, Found (M+H) $^+$: 231.1124.

G1-A2: 17.8 g, 65%. ^{1}H-NMR (700 MHz, $(CD_3)_2SO$) δ 8.06 (d, *J* = 8.5 Hz, 2 H), 6.85 (s, 1H), 6.75 (d, *J* = 8.6 Hz, 2H), 4.95 (t, *J* = 5.5 Hz, 1H), 3.61 (t, *J* = 5.7 Hz, 2H), 3.52 (m, 2H), 2.35 (s, 3H) ppm. HRMS (m/z) Anal. Calc'd for $C_{15}H_{19}N_3O_2$ (M+H) $^+$: 274.1550, Found (M+H) $^+$: 274.1533.

G1-A3: 16.8 g, 56%. ^{1}H-NMR (700 MHz, $(CD_3)_2SO$) δ 7.45 (d, *J* = 8.9 Hz, 2 H), 7.27 (dd, *J1* = 15.5 Hz, *J2* = 11.5 Hz, 1H), 7.10 (d, *J* = 15.5 Hz, 1H), 6.84 (d, *J* = 11.4 Hz, 1H), 6.72 (d, *J* = 8.9 Hz, 2H), 4.95 (t, *J* = 5.4 Hz, 1H), 3.59 (t, *J* = 5.6 Hz, 2H), 3.51 (m, 2H), 2.97 (s, 6H), 2.32 (s, 3H) ppm. HRMS (m/z) Anal. Calc'd for $C_{17}H_{21}N_3O_2$ (M+H) $^+$: 300.1707, Found (M+H) $^+$: 300.1692.

G1-A4: 19.9 g, 55%. ^{1}H-NMR (700 MHz, $(CD_3)_2SO$) δ 8.38 (s, 1H), 8.17 (d, *J* = 7.9 Hz, 1H), 7.53 (d, *J* = 8.2 Hz, 1H), 7.28 (m, 2H), 7.22 (t, *J* = 7.6 Hz, 1H), 4.97 (t, *J* = 6.1 Hz, 1H), 3.90 (s, 3H), 3.64 (t, *J* = 5.6 Hz, 2H), 3.55 (m, 2H), 2.38 (s, 3H) ppm. HRMS (m/z) Anal. Calc'd for $C_{11}H_{11}N_2O_2SI$ (M+H) $^+$: 362.9659 Found (M+H) $^+$: 362.9666.

G1-A5: 11.6 g, 47%. ^{1}H-NMR (700 MHz, $(CD_3)_2SO$) δ 8.08 (d, *J* = 6.9 Hz, 2 H), 6.88 (s, 1H), 6.84 (d, *J* = 7.0 Hz, 2H), 4.93 (s, 1H), 3.62 (t, *J* = 5.6 Hz, 2H), 3.53 (t, *J* = 5.8 Hz, 1H), 2.36 (s, 3H) ppm. HRMS (m/z) Anal. Calc'd for $C_{13}H_{14}N_2O_3$ (M+H) $^+$: 247.1077, Found (M+H) $^+$: 247.1069.

G1-A6: 13.5 g, 52%. ^{1}H-NMR (700 MHz, $(CD_3)_2SO$) δ 8.19 (d, *J* = 7.0 Hz, 2 H), 7.02 (d, *J* = 7.0 Hz, 2H), 6.93 (s, 1H), 4.97 (t, *J* = 5.6 Hz, 1H), 3.81 (s, 3H), 3.63 (t, *J* = 5.5 Hz, 2H), 3.54 (m, 2H), 2.37 (s, 3H) ppm. HRMS (m/z) Anal. Calc'd for $C_{14}H_{16}N_2O_3$ (M+H) $^+$: 261.1234, Found (M+H) $^+$: 261.1225.

G1-A7: 17.9 g, 70%. ^{1}H-NMR (700 MHz, $(CD_3)_2SO$) δ 8.38 (d, *J* = 8.0 Hz, 2 H), 7.90 (d, *J* = 7.6 Hz, 2 H), 7.02 (s, 1H), 4.99 (t, *J* = 5.7 Hz, 1H), 3.65 (t, *J* = 5.4 Hz, 2H), 3.55 (m, 2H), 2.42 (s, 3H) ppm. HRMS (m/z) Anal. Calc'd for $C_{14}H_{13}N_3O_2$ (M+H) $^+$: 256.1081, Found (M+H) $^+$: 256.1071.

G1-A8: 10.2 g, 37%. ^{1}H-NMR (700 MHz, $(CD_3)_2SO$) δ 8.44 (d, *J* = 8.5 Hz, 2 H), 8.28 (d, *J* = 8.5 Hz, 2 H), 7.06 (s, 1H), 5.01 (t, *J* = 5.7 Hz, 1H), 3.66 (t, *J* = 5.6 Hz, 2H), 3.56 (m, 2H), 2.43 (s, 3H) ppm. HRMS (m/z) Anal. Calc'd for $C_{13}H_{13}N_3O_4$ (M+H) $^+$: 276.0979, Found (M+H) $^+$: 276.0970.

G1-A9: 10.4 g, 45%. ^{1}H-NMR (700 MHz, $(CD_3)_2SO$) δ 8.64 (d, *J* = 5.2 Hz, 2 H), 8.08 (d, *J* = 5.0 Hz, 2 H), 6.92 (s, 1H), 5.01 (t, *J* = 5.6 Hz, 1H), 3.65 (t, *J* = 5.5 Hz, 2H), 3.55 (m, 2H), 2.42 (s, 3H) ppm. HRMS (m/z) Anal. Calc'd for $C_{12}H_{13}N_3O_2$ (M+H) $^+$: 232.1081, Found (M+H) $^+$: 232.1089.

G1-A10: 12.8 g, 55%. ^{1}H-NMR (700 MHz, $(CD_3)_2SO$) δ 9.47 (s, 2H), 9.12 (s, 1H), 6.98 (s, 1H), 5.00 (t, *J* = 5.6 Hz, 1H), 3.65 (t, *J* = 5.5 Hz, 2H), 3.55 (m, 2H), 2.42 (s, 3H) ppm. HRMS (m/z) Anal. Calc'd for $C_{11}H_{12}N_4O_2$ (M+H) $^+$: 233.1033, Found (M+H) $^+$: 233.1030.

G1-A11: 11.6 g, 32%. ^{1}H-NMR (700 MHz, $(CD_3)_2SO$) δ 7.38 (d, *J* = 3.9 Hz, 1H), 7.31 (d, *J* = 3.9 Hz, 1H), 7.27 (s, 1H), 4.97 (t, *J* = 5.6 Hz, 1H), 3.61 (t, *J* = 4.9 Hz, 2H), 3.52 (m, 2H), 2.36 (s, 3H) ppm. HRMS (m/z) Anal. Calc'd for $C_{11}H_{11}N_2O_2SI$ (M+H) $^+$: 362.9659, Found (M+H) $^+$: 362.9666.

G1-A12: 12.1 g, 42%. ^{1}H-NMR (700 MHz, $(CD_3)_2SO$) δ 8.18 (d, *J* = 8.1 Hz, 1H), 8.08 (d, *J* = 8.1 Hz, 1H), 7.57 (m, 1H), 7.51 (m, 1H), 7.19 (s, 1H), 5.03 (t, *J* = 6.0 Hz, 1H), 3.68 (t, *J* = 5.5 Hz, 2H), 3.57 (m, 2H), 2.47 (s, 3H) ppm. HRMS (m/z) Anal. Calc'd for $C_{14}H_{13}N_3O_2S$ (M+H) $^+$: 288.0801, Found (M+H) $^+$: 288.0770.

Synthesis of A1B1~A12B12**:** To the 10 mL heavy-wall pressure tube 1 mmol G-A and 5 mmol aryl aldehyde were dissolved in 3 mL anhydrous 1,4-dioxane with inserted argon atmosphere. After added 0.1 mL $ZnCl_2$ solution (1.0 M in THF), the reaction vessel was sealed up with Teflon screw cap with Viton O-ring and then kept heating in oil bath at 95°C overnight. After cooling down to room temperature, the reaction mixture was poured into 50 mL DCM. The organic mixture was washed with dd water and brine. The crude product was concentrated by vacuum evaporation and purified via silica gel chromatography to yield desired product.

Note: some products exhibited high solubility in water, such as A5B5, so they were concentrated and purified via silica gel chromatography directly.

The yield and characterization of these products were listed below.

A1B1: 184.7 mg, 58%. ^{1}H-NMR (700 MHz, $(CD_3)_2SO$) δ 8.32 (d, *J* = 7.6 Hz, 2H), 8.05 (d, *J* = 15.7 Hz, 1H), 7.85 (d, *J* = 7.5 Hz, 2H), 7.46 (m, 6H), 7.30 (d, *J* = 15.7 Hz, 1H), 7.04 (s, 1H), 4.96 (t, *J* = 5.7 Hz, 1H), 3.86 (t, *J* = 5.5 Hz, 2H), 3.58 (q, *J* = 5.7 Hz, 2H) ppm. ^{13}C-NMR (176 MHz, $(CD_3)_2SO$) δ 170.2, 161.0, 140.0, 139.5, 135.2, 134.6, 132.2, 130.2, 130.0, 129.0, 128.8, 128.4, 124.7, 114.6, 59.3, 42.4 ppm. HRMS (m/z) Anal. Calc'd for $C_{20}H_{18}N_2O_2$ (M+H) $^+$: 319.1441, Found (M+H) $^+$: 319.1448.

A1B2: 61.9 mg, 17%. ^{1}H-NMR (700 MHz, $(CD_3)_2SO$) δ 8.30 (d, *J* = 7.8 Hz, 2H), 7.99 (d, *J* = 15.4 Hz, 1H), 7.68 (d, *J* = 8.4 Hz, 2H), 7.48 (t, *J* = 7.6 Hz, 2H), 7.40 (t, *J* = 7.4 Hz, 1H), 6.96 (d, *J* = 15.5 Hz, 1H), 6.90 (s, 1H), 6.77 (d, *J* = 8.4 Hz, 2H), 4.94 (t, *J* = 5.7 Hz, 1H), 3.83 (t, *J* = 5.7 Hz, 2H), 3.58 (m, 2H), 3.01 (s, 6H) ppm. ^{13}C-NMR (176 MHz, $(CD_3)_2SO$) δ 170.3, 161.6, 151.7, 141.2, 139.9, 135.0, 131.8, 130.2, 129.4, 128.7, 122.7, 122.2, 111.8, 107.9, 59.3, 42.2 ppm. HRMS (m/z) Anal. Calc'd for $C_{22}H_{23}N_3O_2$ (M+H) $^+$: 362.1863, Found (M+H) $^+$: 362.1848.

A1B3: 41.6 mg, 11%. ^{1}H-NMR (700 MHz, $(CD_3)_2SO$) δ 8.37 ~ 6.18 (m, 14H), 4.95 (m, 1H), 3.75 (m, 2H), 3.55 (m, 2H), 3.02 (m, 6H) ppm. ^{13}C-NMR (176 MHz, $(CD_3)_2SO$) δ 170.2, 169.6, 161.1, 160.5, 151.2, 151.0, 143.7,

143.0, 142.4, 141.3, 140.0, 139.9, 134.9, 134.9, 131.9, 131.7, 130.0, 129.6, 128.9, 128.9, 128.7, 128.7, 123.9, 123.8, 123.4, 123.0, 122.9, 122.3, 113.9, 112.2, 112.1, 109.4, 59.1, 59.1, 42.4, 42.3 ppm. HRMS (m/z) Anal. Calc'd for $C_{24}H_{28}N_4O_2$ (M+H) $^+$: 388.2020, Found (M+H) $^+$: 388.2040.

A1B4: 78.0 mg, 21%. ^{1}H-NMR (700 MHz, $(CD_3)_2SO$) δ 9.71 – 6.11 (m, 13H), 5.04 (m, 1H), 3.97 – 3.73 (m, 5H), 3.60 (m, 2H) ppm. ^{13}C-NMR (176 MHz, $(CD_3)_2SO$) δ 170.5, 169.8, 162.1, 160.6, 140.0, 140.0, 138.1, 136.5, 136.0, 135.8, 135.1, 134.8, 132.5, 131.8, 130.9, 130.0, 129.4, 129.0, 128.8, 128.5, 125.4, 123.8, 123.0, 122.7, 121.8, 121.5, 121.1, 120.5, 118.2, 112.6, 111.5, 111.0, 110.7, 107.3, 105.9, 59.5, 59.2, 42.7, 42.5, 33.4, 33.1 ppm. HRMS (m/z) Anal. Calc'd for $C_{23}H_{21}N_3O_2$ (M+H) $^+$: 372.1707, Found (M+H) $^+$: 372.1711.

A1B5: 96.8 mg, 29%. ^{1}H-NMR (700 MHz, $(CD_3)_2SO$) δ 10.08 (s, 1H), 8.30 (d, *J* = 7.7 Hz, 2H), 7.98 (d, *J* = 15.6 Hz, 1H), 7.70 (d, *J* = 8.1 Hz, 2H), 7.47 (t, *J* = 7.5 Hz, 2H), 7.41 (t, *J* = 7.4 Hz, 1H), 7.05 (d, *J* = 15.6 Hz, 1H), 6.96 (s, 1H), 6.86 (d, *J* = 8.7 Hz, 2H), 4.94 (s, 1H), 3.83 (t, *J* = 5.8 Hz, 2H), 3.56 (m, 2H) ppm. ^{13}C-NMR (176 MHz, $(CD_3)_2SO$) δ 170.3, 161.4, 159.9, 140.6, 139.7, 134.8, 132.0, 130.5, 129.7, 128.7, 126.4, 123.4, 115.9, 110.6, 59.3, 42.3 ppm. HRMS (m/z) Anal. Calc'd for $C_{20}H_{18}N_2O_3$ (M+H) $^+$: 335.1390, Found (M+H) $^+$: 335.1389.

A1B6: 72.2 mg, 21%. ^{1}H-NMR (700 MHz, $(CD_3)_2SO$) δ 8.35 – 6.34 (m, 12H), 4.96 (dt, *J* = 15.9, 5.7 Hz, 1H), 3.87 – 3.67 (m, 5H), 3.56 (m, 2H) ppm. ^{13}C-NMR (176 MHz, $(CD_3)_2SO$) δ 170.4, 169.7, 161.3, 161.1, 160.4, 160.0, 141.9, 140.1, 139.7, 139.3, 134.8, 134.4, 132.8, 132.1, 131.9, 130.3, 129.9, 128.8, 128.7, 128.1, 128.0, 125.5, 123.9, 114.6, 113.4, 112.8, 111.8, 59.3, 59.0, 55.5, 55.4, 42.8, 42.4 ppm. HRMS (m/z) Anal. Calc'd for $C_{21}H_{20}N_2O_3$ (M+H) $^+$: 349.1547, Found (M+H) $^+$: 349.1562.

A1B7: 263.3 mg, 77%. ^{1}H-NMR (700 MHz, $(CD_3)_2SO$) δ 8.33 (d, *J* = 7.6 Hz, 2H), 8.11 – 8.03 (m, 3H), 7.94 (d, *J* = 7.9 Hz, 2H), 7.52 – 7.41 (m, 4H), 7.09 (s, 1H), 4.95 (t, *J* = 5.9 Hz, 1H), 3.88 (t, *J* = 5.7 Hz, 2H), 3.57 (q, *J* = 5.7 Hz, 2H) ppm. ^{13}C-NMR (176 MHz, $(CD_3)_2SO$) δ 170.1, 160.6, 139.7, 139.4, 137.7, 134.4, 132.8, 132.3, 130.2, 128.9, 128.8, 125.8, 118.7, 118.2, 111.8, 59.2, 42.4 ppm. HRMS (m/z) Anal. Calc'd for $C_{21}H_{17}N_3O_2$ (M+H) $^+$: 344.1394, Found (M+H) $^+$: 344.1394.

A1B8: 249.5 mg, 69 %. ^{1}H-NMR (700 MHz, $(CD_3)_2SO$) δ 8.32 (dd, *J* = 25.2, 8.2 Hz, 4H), 8.19 – 8.05 (m, 3H), 7.55 – 7.41 (m, 4H), 7.10 (s, 1H), 4.97 (t, *J* = 5.7 Hz, 1H), 3.89 (t, *J* = 5.4 Hz, 2H), 3.58 (q, *J* = 5.6 Hz, 2H) ppm. ^{13}C-NMR (176 MHz, $(CD_3)_2SO$) δ 170.1, 160.5, 147.7, 141.7, 139.4, 137.2, 134.5, 132.4, 130.3, 129.4, 128.9, 126.1, 124.1, 119.0, 59.3, 42.4 ppm. HRMS (m/z) Anal. Calc'd for $C_{20}H_{17}N_3O_4$ (M+H) $^+$: 364.1292, Found (M+H) $^+$: 364.1296.

A1B9: 195.2 mg, 61%. ^{1}H-NMR (700 MHz, $(CD_3)_2SO$) δ 8.61 (m, 2H), 8.24 (m, 2H), 8.04 – 6.96 (m, 8H), 4.97 (t, *J* = 5.7 Hz, 1H), 3.91 – 3.64 (m, 2H), 3.61 – 3.48 (m, 2H) ppm. ^{13}C-NMR (176 MHz, $(CD_3)_2SO$) δ 170.1, 170.1, 165.0, 160.5, 153.3, 150.4, 149.5, 142.3, 139.4, 138.6, 137.1, 134.4, 134.1, 132.4, 132.1, 130.4, 130.1, 128.9, 128.8, 126.2, 125.3, 122.2, 121.4, 119.4, 69.1, 59.3, 58.9, 42.8, 42.5 ppm. HRMS (m/z) Anal. Calc'd for $C_{19}H_{17}N_3O_2$ (M+H) $^+$: 320.1394, Found (M+H) $^+$: 320.1398.

A1B10: 40.6 mg, 13%. ^{1}H-NMR (700 MHz, $(CD_3)_2SO$) δ 9.29 (s, 2H), 9.19 (s, 1H), 8.33 (d, *J* = 7.6 Hz, 2H), 8.03 (d, *J* = 16.0 Hz, 1H), 7.70 – 7.34 (m, 4H), 7.10 (s, 1H), 4.93 (t, *J* = 6.0 Hz, 1H), 3.88 (t, *J* = 5.6 Hz, 2H), 3.58 (q, *J* = 5.5 Hz, 2H) ppm. ^{13}C-NMR (176 MHz, $(CD_3)_2SO$) δ 170.1, 160.4, 158.4, 156.1, 139.3, 134.4, 133.0, 132.3, 130.3, 129.3, 128.8, 125.9, 118.3, 59.2, 42.3 ppm. HRMS (m/z) Anal. Calc'd for $C_{18}H_{18}N_4O_2$ (M+H) $^+$: 321.1346, Found (M+H) $^+$: 321.1347.

A1B11: 118.9 mg, 26%. ^{1}H-NMR (700 MHz, $(CD_3)_2SO$) δ 8.37 – 6.80 (m, 10H), 4.98 (m, 1H), 3.82 (m, 2H), 3.56 (m, 2H) ppm. ^{13}C-NMR (176 MHz, $(CD_3)_2SO$) δ 170.3, 170.2, 161.1, 160.7, 146.3, 140.1, 139.6, 139.5, 138.5, 135.2, 134.6, 134.6, 133.3, 132.2, 132.2, 131.6, 130.3, 130.1, 130.0, 129.1, 128.9, 128.9, 128.4, 124.8, 124.6, 114.6, 113.8, 81.6, 59.3, 59.3, 42.5, 42.4 ppm. HRMS (m/z) Anal. Calc'd for $C_{18}H_{15}N_2O_2SI$ (M+H) $^+$: 450.9972, Found (M+H) $^+$: 450.9979.

A1B12: 183.2 mg, 49%. ^{1}H-NMR (700 MHz, $(CD_3)_2SO$) δ 8.36 (d, *J* = 7.3 Hz, 2H), 8.27 – 8.16 (m, 2H), 8.10 (d, *J* = 8.1 Hz, 1H), 7.69 – 7.57 (m, 2H), 7.56 – 7.42 (m, 4H), 7.17 (s, 1H), 5.08 (t, *J* = 5.5 Hz, 1H), 3.87 (t, *J* = 5.3 Hz, 2H), 3.59 (q, *J* = 5.4 Hz, 2H) ppm. ^{13}C-NMR (176 MHz, $(CD_3)_2SO$) δ 169.9, 164.0, 159.8, 153.4, 139.2, 135.2, 134.3, 132.5, 131.1, 130.5, 128.9, 127.1, 126.9, 126.5, 123.4, 122.6, 122.3, 59.2, 42.6 ppm. HRMS (m/z) Anal. Calc'd for $C_{21}H_{17}N_3O_2S$ (M+H) $^+$: 376.1114, Found (M+H) $^+$: 376.1106.

A2B1: 262.7 mg, 73%. ^{1}H-NMR (700 MHz, $(CD_3)_2SO$) δ 8.19 (d, *J* = 8.4 Hz, 2H), 7.91 (d, *J* = 15.7 Hz, 1H), 7.80 (d, *J* = 7.5 Hz, 2H), 7.50 – 7.37 (m, 3H), 7.25 (d, *J* = 15.7 Hz, 1H), 6.95 (s, 1H), 6.80 (d, *J* = 8.6 Hz, 2H), 4.93 (t, *J* = 5.7 Hz, 1H), 3.83 (t, *J* = 5.6 Hz, 2H), 3.55 (q, *J* = 5.7 Hz, 2H), 3.04 (s, 6H) ppm. ^{13}C-NMR (176 MHz, $(CD_3)_2SO$) δ 169.2, 157.1, 151.3, 137.8, 135.5, 135.2, 134.2, 129.7, 128.9, 128.0, 126.8, 122.2, 115.0, 111.8, 59.4, 42.2 ppm. HRMS (m/z) Anal. Calc'd for $C_{22}H_{23}N_3O_2$ (M+H) $^+$: 362.1863, Found (M+H) $^+$: 362.1864.

A2B2: 65.7 mg, 16%. ^{1}H-NMR (700 MHz, $(CD_3)_2SO$) δ 8.16 (d, *J* = 8.5 Hz, 2H), 7.84 (d, *J* = 15.5 Hz, 1H), 7.62 (d, *J* = 8.9 Hz, 2H), 6.91 (d, *J* = 15.6 Hz, 1H), 6.83 (s, 1H), 6.77 (m, 4H), 4.91 (t, *J* = 5.7 Hz, 1H), 3.79 (t, *J* = 5.8 Hz, 2H), 3.54 (q, *J* = 5.8 Hz, 2H), 3.03 (s, 6H), 3.00 (s, 6H) ppm. ^{13}C-NMR (176 MHz, $(CD_3)_2SO$) δ 170.0, 158.0, 151.3, 151.0, 138.9, 135.7, 133.4, 129.7, 124.6, 123.1, 122.6, 111.9, 111.8, 108.6, 59.4, 42.1 ppm. HRMS (m/z) Anal. Calc'd for $C_{24}H_{28}N_4O_2$ (M+H) $^+$: 405.2285, Found (M+H) $^+$: 405.2280.

A2B3: 48.6 mg, 11%. ^{1}H-NMR (700 MHz, $(CD_3)_2SO$) δ 8.16 (m, 2H), 7.88 – 7.35 (m, 3H), 7.20 – 6.39 (m, 8H), 4.92 (t, *J* = 6.0 Hz, 1H), 3.75 (m, 2H), 3.53 (m, 2H), 3.11 – 2.90 (m, 12H) ppm. ^{13}C-NMR (176 MHz, $(CD_3)_2SO$) δ 169.9, 157.4, 151.2, 150.8, 140.2, 139.8, 135.8, 134.0, 129.8, 128.7, 125.4, 124.1, 123.3, 122.6, 114.6, 112.2, 111.9, 59.3, 42.2 ppm. HRMS (m/z) Anal. Calc'd for $C_{26}H_{30}N_4O_2$ (M+H) $^+$: 431.2442, Found (M+H) $^+$: 431.2455.

A2B4: 83.4 mg, 20%. ^{1}H-NMR (700 MHz, $(CD_3)_2SO$) δ 9.76 – 6.02 (m, 12H), 5.31 – 4.74 (m, 1H), 4.05 – 3.51 (m, 7H), 3.06 – 2.96 (m, 6H) ppm. ^{13}C-NMR (176 MHz, $(CD_3)_2SO$) δ 170.2, 158.5, 151.0, 138.0, 135.8, 134.8, 133.8, 132.5, 125.5, 124.3, 122.8, 122.7, 121.2, 120.4, 112.6, 112.0, 111.9, 111.4, 110.8, 108.1, 59.6, 42.4, 33.0 ppm. HRMS (m/z) Anal. Calc'd for $C_{25}H_{26}N_4O_2$ (M+H) $^+$: 415.2129, Found (M+H) $^+$: 415.2129.

A2B5: 175.0 mg, 46%. ^{1}H-NMR (700 MHz, $(CD_3)_2SO$) δ 9.98 (s, 1H), 8.16 (d, *J* = 8.4 Hz, 2H), 7.84 (d, *J* = 15.6 Hz, 1H), 7.64 (d, *J* = 8.2 Hz, 2H), 7.00 (d, *J* = 15.7 Hz, 1H), 6.88 (s, 1H), 6.84 (d, *J* = 8.1 Hz, 2H), 6.78 (d, *J* = 8.6 Hz, 2H), 4.91 (s, 1H), 3.80 (t, *J* = 5.8 Hz, 2H), 3.54 (m, 2H), 3.03 (s, 6H) ppm. ^{13}C-NMR (176 MHz, $(CD_3)_2SO$) δ 169.9, 159.3, 157.6, 151.2, 138.3, 135.5, 134.0, 129.9, 126.7, 125.6, 122.4, 115.8, 111.8, 111.1, 59.4, 42.1 ppm. HRMS (m/z) Anal. Calc'd for $C_{22}H_{23}N_3O_3$ (M+H) $^+$: 378.1812, Found (M+H) $^+$: 378.1817.

A2B6: 205.3 mg, 53%. ^{1}H-NMR (700 MHz, $(CD_3)_2SO$) δ 8.17 (d, *J* = 8.6 Hz, 2H), 7.88 (d, *J* = 15.7 Hz, 1H), 7.76 (d, *J* = 8.7 Hz, 2H), 7.08 (d, *J* = 15.7 Hz, 1H), 7.05 – 6.99 (m, 2H), 6.90 (s, 1H), 6.79 (d, *J* = 9.3 Hz, 2H), 4.91 (t, *J* = 5.7 Hz, 1H), 3.82 (m, 5H), 3.55 (q, *J* = 5.7 Hz, 2H), 3.03 (s, 6H) ppm. ^{13}C-NMR (176 MHz, $(CD_3)_2SO$) δ 169.9, 160.6, 157.5, 151.2, 137.8, 135.4, 134.1, 129.7, 128.2, 126.0, 122.3, 114.4, 112.2, 111.8, 59.4, 55.4, 42.1 ppm. HRMS (m/z) Anal. Calc'd for $C_{23}H_{25}N_3O_3$ (M+H) $^+$: 392.1969, Found (M+H) $^+$: 392.1966.

A2B7: 218.1 mg, 56%. ^{1}H-NMR (700 MHz, $(CD_3)_2SO$) δ 8.20 (d, *J* = 8.4 Hz, 2H), 8.00 (d, *J* = 8.0 Hz, 2H), 7.92 (m, 3H), 7.42 (d, *J* = 15.8 Hz, 1H), 7.00 (s, 1H), 6.79 (d, *J* = 8.6 Hz, 2H), 4.92 (t, *J* = 5.7 Hz, 1H), 3.85 (t, *J* = 5.7 Hz, 2H), 3.55 (q, *J* = 5.8 Hz, 2H), 3.05 (s, 6H) ppm. ^{13}C-NMR (176 MHz, $(CD_3)_2SO$) δ 169.6, 156.5, 151.5, 140.1, 135.5, 135.1, 134.5, 132.7, 128.6, 127.8, 122.1, 118.8, 118.6, 111.8, 111.2, 59.4, 42.2 ppm. HRMS (m/z) Anal. Calc'd for $C_{23}H_{22}N_4O_2$ (M+H) $^+$: 388.1894, Found (M+H) $^+$: 388.1860.

A2B8: 294.6 mg, 73%. ^{1}H-NMR (700 MHz, $(CD_3)_2SO$) δ 8.29 (d, *J* = 8.8 Hz, 2H), 8.20 (d, *J* = 8.5 Hz, 2H), 8.07 (d, *J* = 8.8 Hz, 2H), 7.99 (d, *J* = 15.8 Hz, 1H), 7.46 (d, *J* = 15.8 Hz, 1H), 7.01 (s, 1H), 6.79 (d, *J* = 9.3 Hz, 2H), 4.93 (t, *J* = 5.6 Hz, 1H), 3.86 (t, *J* = 5.6 Hz, 2H), 3.56 (q, *J* = 5.6 Hz, 2H), 3.05 (s, 6H) ppm. ^{13}C-NMR (176 MHz, $(CD_3)_2SO$) δ 169.6, 156.4, 151.6, 147.3, 142.1, 135.1, 134.9, 134.6, 128.9, 128.1, 124.1, 122.1, 119.4, 111.8, 59.4, 42.2 ppm. HRMS (m/z) Anal. Calc'd for $C_{22}H_{22}N_4O_4$ (M+H) $^+$: 407.1714, Found (M+H) $^+$: 407.1712.

A2B9: 161.3 mg, 45%. ^{1}H-NMR (700 MHz, $(CD_3)_2SO$) δ 8.65 (d, *J* = 6.1 Hz, 2H), 8.20 (d, *J* = 8.5 Hz, 2H), 7.85 (d, *J* = 15.8 Hz, 1H), 7.75 (d, *J* = 6.1 Hz, 2H), 7.49 (d, *J* = 15.8 Hz, 1H), 7.01 (s, 1H), 6.79 (d, *J* = 9.2 Hz, 2H), 4.93 (t, *J* = 5.6 Hz, 1H), 3.85 (t, *J* = 5.6 Hz, 2H), 3.55 (q, *J* = 5.5 Hz, 2H), 3.05 (s, 6H) ppm. ^{13}C-NMR (176 MHz, $(CD_3)_2SO$) δ 169.6, 156.4, 151.6, 150.3, 142.6, 135.0, 134.7, 134.5, 128.1, 122.0, 121.8, 119.6, 111.8, 59.4, 42.2 ppm. HRMS (m/z) Anal. Calc'd for $C_{21}H_{22}N_4O_2$ (M+H) $^+$: 363.1816, Found (M+H) $^+$: 363.1834.

A2B10: 102.8 mg, 28%. ^{1}H-NMR (700 MHz, $(CD_3)_2SO$) δ 9.36 – 9.05 (m, 3H), 8.29 (m, 2H), 7.80 (m, 1H), 7.46 (m, 1H), 7.16 (m, 1H), 6.77 (m, 2H), 4.89 (m, 1H), 3.86 (m, 2H), 3.57 (m, 2H), 3.05 (m, 6H) ppm. ^{13}C-NMR (176 MHz, $(CD_3)_2SO$) δ 169.6, 158.1, 158.1, 156.4, 155.8, 155.7, 151.6, 151.5, 135.0, 134.5, 130.6, 129.6, 128.0, 122.0, 122.0, 118.6, 111.8, 111.8, 59.4, 42.1 ppm. HRMS (m/z) Anal. Calc'd for $C_{21}H_{20}N_5O_2$ (M+H) $^+$: 364.1768, Found (M+H) $^+$: 364.1772.

A2B11: 330.3 mg, 67%. ^{1}H-NMR (700 MHz, $(CD_3)_2SO$) δ 8.25 (m, 2H), 7.90 (m, 1H), 7.38 (m, 1H), 7.28 – 6.67 (m, 5H), 4.96 (m, 1H), 3.76 (m, 2H), 3.53 (m, 2H), 3.03 (m, 6H) ppm. ^{13}C-NMR (176 MHz, $(CD_3)_2SO$) δ 169.7, 156.7, 151.4, 146.7, 138.3, 135.3, 134.3, 132.3, 129.4, 126.9, 122.2, 114.4, 111.9, 80.3, 59.5, 42.3 ppm. HRMS (m/z) Anal. Calc'd for $C_{20}H_{20}N_3O_2SI$ (M+H) $^+$: 494.0394, Found (M+H) $^+$: 494.0392.

A2B12: 264.5 mg, 63%. ^{1}H-NMR (700 MHz, $(CD_3)_2SO$) δ 8.45 – 7.84 (m, 5H), 7.70 – 7.46 (m, 3H), 7.07 (s, 1H), 6.83 (m, 2H), 5.05 (t, *J* = 5.4 Hz, 1H), 3.84 (t, *J* = 5.5 Hz, 2H), 3.57 (m, 2H), 3.05 (m, 6H) ppm. ^{13}C-NMR (176 MHz, $(CD_3)_2SO$) δ 169.4, 164.4, 155.5, 153.5, 151.8, 135.0, 134.9, 134.8, 129.0, 128.9, 127.0, 126.2, 123.2, 122.7, 122.5, 122.0, 111.9, 59.4, 42.4 ppm. HRMS (m/z) Anal. Calc'd for $C_{23}H_{22}N_4O_2S$ (M+H) $^+$: 419.1536, Found (M+H) $^+$: 419.1513.

A3B1: 222.9 mg, 58%. ^{1}H-NMR (700 MHz, $(CD_3)_2SO$) δ 8.13 – 6.66 (m, 14H), 4.93 (t, *J* = 5.7 Hz, 1H), 3.82 (m, 2H), 3.55 (m, 2H), 2.99 (m, 6H) ppm. ^{13}C-NMR (176 MHz, $(CD_3)_2SO$) δ 168.8, 167.9, 156.9, 156.1, 151.1, 143.6, 142.9, 138.1, 137.8, 137.4, 137.3, 135.5, 135.5, 134.7, 129.7, 129.6, 129.2, 129.0, 128.9, 128.9, 128.6, 128.0, 127.9, 124.0, 124.0, 119.5, 118.7, 114.7, 112.2, 112.1, 59.4, 59.3, 54.9, 42.2, 42.0 ppm. HRMS (m/z) Anal. Calc'd for $C_{24}H_{25}N_3O_2$ (M+H) $^+$: 388.2020, Found (M+H) $^+$: 388.1996.

A3B2: 58.8 mg, 14%. ^{1}H-NMR (700 MHz, $(CD_3)_2SO$) δ 8.12 – 6.64 (m, 13H), 4.91 (t, *J* = 5.9 Hz, 1H), 3.87 – 3.64 (m, 2H), 3.54 (m, 2H), 3.09 – 2.90 (m, 12H) ppm. ^{13}C-NMR (176 MHz, $(CD_3)_2SO$) δ 169.0, 168.8, 168.1, 157.7, 157.1, 156.8, 151.4, 151.3, 151.0, 150.9, 150.9, 142.1, 141.9, 141.5, 139.4, 132.6, 129.7, 129.7, 129.6, 129.0, 129.0, 128.8, 128.6, 126.9, 126.3, 124.3, 124.2, 123.1, 123.0, 119.8, 119.0, 112.2, 112.1, 111.9, 111.9, 59.4, 59.3, 59.2, 54.9, 42.1, 41.9 ppm. HRMS (m/z) Anal. Calc'd for $C_{26}H_{30}N_4O_2$ (M+H) $^+$: 431.2442, Found (M+H) $^+$: 431.2413.

A3B3: 68.6 mg, 15%. ^{1}H-NMR (700 MHz, $(CD_3)_2SO$) δ 8.76 – 6.02 (m, 15H), 4.94 (m, 1H), 3.75 – 3.64 (m, 2H), 3.53 (m, 2H), 2.97 (m, 12H) ppm. ^{13}C-NMR (176 MHz, $(CD_3)_2SO$) δ 168.9, 168.0, 157.2, 156.3, 151.1, 151.1, 150.8, 150.8, 142.7, 142.1, 140.5, 140.0, 139.8, 139.6, 138.3, 137.8, 133.4, 129.1, 129.0, 128.7, 128.6, 128.6, 127.1, 124.3, 124.3, 124.1, 124.1, 123.4, 123.3, 119.8, 119.0, 114.5, 114.3, 112.3, 112.2, 112.2, 59.3, 59.2, 42.2, 42.0 ppm. HRMS (m/z) Anal. Calc'd for $C_{28}H_{32}N_4O_2$ (M+H) $^+$: 457.2598, Found (M+H) $^+$: 457.2564.

A3B4: not obtained.

A3B5: 65.7 mg, 16%. ^{1}H-NMR (700 MHz, $(CD_3)_2SO$) δ 9.97 (m, 1H), 8.32 – 6.62 (m, 13H), 4.90 (t, *J* = 5.7 Hz, 1H), 3.78 (m, 2H), 3.54 (m, 2H), 2.99 (m, 6H) ppm. ^{13}C-NMR (176 MHz, $(CD_3)_2SO$) δ 168.9, 168.0, 159.4, 159.3, 157.4, 156.5, 151.0, 151.0, 142.8, 142.2, 138.6, 138.1, 137.9, 137.5, 133.6, 130.0, 129.8, 129.1, 128.9, 127.4, 126.7, 126.7, 124.1, 119.6, 118.9, 115.8, 115.8, 112.2, 112.1, 110.9, 110.9, 59.4, 59.3, 42.1, 41.9 ppm. HRMS (m/z) Anal. Calc'd for $C_{24}H_{25}N_3O_3$ (M+H) $^+$: 404.1969, Found (M+H) $^+$: 404.1955.

A3B6: 163.1 mg, 39%. ^{1}H-NMR (700 MHz, $(CD_3)_2SO$) δ 8.18 – 6.52 (m, 13H), 4.94 (t, *J* = 5.6 Hz, 1H), 3.93 – 3.68 (m, 5H), 3.55 (m, 2H), 2.98 (s, 6H) ppm. ^{13}C-NMR (176 MHz, $(CD_3)_2SO$) δ 169.0, 168.1, 160.8, 160.7,

157.4, 156.5, 151.2, 151.1, 143.2, 142.5, 138.2, 138.0, 137.5, 137.5, 134.0, 129.8, 129.7, 129.2, 129.0, 128.3, 128.2, 127.9, 124.2, 124.2, 119.6, 118.9, 114.5, 114.5, 112.3, 112.2, 112.0, 112.0, 59.4, 59.4, 55.4, 55.4, 42.2, 42.0 ppm. HRMS (m/z) Anal. Calc'd for $C_{25}H_{27}N_3O_3$ (M+H) $^+$: 418.2165, Found (M+H) $^+$: 418.2100.

A3B7: 320.6 mg, 78%. ^{1}H-NMR (700 MHz, $(CD_3)_2SO$) δ 8.21 – 6.61 (m, 13H), 4.92 (t, *J* = 5.7 Hz, 1H), 3.84 (m, 2H), 3.55 (m, 2H), 3.00 (s, 6H) ppm. ^{13}C-NMR (176 MHz, $(CD_3)_2SO$) δ 168.7, 167.8, 156.4, 155.5, 151.3, 144.5, 143.7, 140.1, 140.1, 137.7, 137.2, 135.8, 135.7, 135.1, 132.8, 132.7, 129.6, 129.4, 129.3, 128.6, 128.5, 124.0, 123.9, 119.5, 118.8, 118.7, 118.4, 112.2, 112.1, 111.3, 111.2, 59.4, 59.4, 42.2, 42.0 ppm. HRMS (m/z) Anal. Calc'd for $C_{25}H_{25}N_4O_2$ (M+H) $^+$: 413.1972, Found (M+H) $^+$: 413.1965.

A3B8: 306.4 mg, 71%. ^{1}H-NMR (700 MHz, $(CD_3)_2SO$) δ 8.60 – 6.46 (m, 13H), 4.93 (t, *J* = 5.6 Hz, 1H), 3.85 (m, 2H), 3.56 (m, 2H), 3.00 (s, 6H) ppm. ^{13}C-NMR (176 MHz, $(CD_3)_2SO$) δ 168.6, 167.7, 156.3, 155.4, 151.3, 147.4, 147.3, 144.7, 143.9, 142.1, 142.0, 137.7, 137.2, 136.0, 135.2, 134.5, 129.9, 129.4, 129.3, 128.9, 128.8, 124.1, 124.0, 123.9, 123.9, 119.5, 119.2, 119.2, 118.7, 112.2, 112.1, 59.4, 59.3, 42.2, 42.0 ppm. HRMS (m/z) Anal. Calc'd for $C_{24}H_{24}N_4O_4$ (M+H) $^+$: 433.1870, Found (M+H) $^+$: 433.1877.

A3B9: 223.4 mg, 58%. ^{1}H-NMR (700 MHz, $(CD_3)_2SO$) δ 8.78 – 6.44 (m, 13H), 4.93 (t, *J* = 5.7 Hz, 1H), 3.91 – 3.78 (m, 2H), 3.55 (m, 2H), 3.00 (m, 6H) ppm. ^{13}C-NMR (176 MHz, $(CD_3)_2SO$) δ 168.6, 167.7, 156.2, 155.3, 151.3, 150.3, 150.3, 144.6, 143.8, 142.5, 142.5, 137.6, 137.1, 135.9, 135.1, 134.3, 129.9, 129.4, 129.3, 123.9, 123.9, 121.8, 121.7, 119.4, 119.4, 118.6, 112.2, 112.1, 59.4, 59.3, 54.9, 42.2, 42.0 ppm. HRMS (m/z) Anal. Calc'd for $C_{23}H_{24}N_4O_2$ (M+H) $^+$: 389.1972, Found (M+H) $^+$: 389.1975.

A3B10: 84.5 mg, 22%. ^{1}H-NMR (700 MHz, $(CD_3)_2SO$) δ 9.39 – 8.99 (m, 3H), 8.13 – 6.94 (m, 7H), 6.75 (d, *J* = 8.2 Hz, 2H), 4.89 (t, *J* = 5.6 Hz, 1H), 3.84 (m, 2H), 3.56 (m, 2H), 3.00 (s, 6H) ppm. ^{13}C-NMR (176 MHz, $(CD_3)_2SO$) δ 168.6, 167.7, 158.1, 158.0, 156.2, 155.8, 155.7, 155.3, 151.3, 144.5, 143.7, 137.6, 137.1, 135.8, 131.0, 130.2, 129.7, 129.6, 129.5, 129.3, 129.2, 123.9, 119.4, 118.6, 118.4, 112.2, 112.1, 59.3, 59.3, 42.2, 42.0 ppm. HRMS (m/z) Anal. Calc'd for $C_{22}H_{23}N_5O_2$ (M+H) $^+$: 390.1925, Found (M+H) $^+$: 390.1933.

A3B11: 292.3 mg, 56%. ^{1}H-NMR (700 MHz, $(CD_3)_2SO$) δ 8.16 – 6.54 (m, 11H), 4.95 (t, *J* = 5.6 Hz, 1H), 3.74 (m, 2H), 3.52 (m, 2H), 2.98 (m, 6H) ppm. ^{13}C-NMR (176 MHz, $(CD_3)_2SO$) δ 168.8, 156.5, 151.3, 146.6, 143.1, 138.4, 137.9, 132.5, 129.8, 129.3, 128.6, 124.1, 118.8, 114.1, 112.2, 80.5, 59.4, 55.0, 42.3 ppm. HRMS (m/z) Anal. Calc'd for $C_{22}H_{22}N_3O_2SI$ (M+H) $^+$: 520.0550, Found (M+H) $^+$: 520.0561.

A3B12: 126.1 mg, 28%. ^{1}H-NMR (700 MHz, $(CD_3)_2SO$) δ 8.20 – 8.13 (m, 1H), 8.10 – 7.91 (m, 3H), 7.62 – 7.41 (m, 6H), 7.28 – 7.04 (m, 2H), 6.75 (m, 2H), 5.05 (m, 1H), 3.83 (m, 2H), 3.57 (m, 2H), 3.00 (m, 6H) ppm. ^{13}C-NMR (176 MHz, $(CD_3)_2SO$) δ 168.5, 167.5, 164.4, 164.3, 155.4, 153.5, 151.5, 151.4, 145.5, 144.6, 137.5, 137.0, 137.0, 135.1, 135.0, 130.9, 129.7, 129.5, 129.2, 128.4, 127.0, 127.0, 126.2, 126.2, 123.9, 123.8, 123.2, 123.1, 122.7, 122.5, 122.5, 119.5, 118.7, 112.2, 112.1, 59.4, 59.3, 42.4, 42.3 ppm. HRMS (m/z) Anal. Calc'd for $C_{25}H_{24}N_4O_2S$ (M+H) $^+$: 445.1693, Found (M+H) $^+$: 445.1698.

A4B1: 261.3 mg, 70%. ^{1}H-NMR (700 MHz, $(CD_3)_2SO$) δ 9.38 – 7.16 (m, 13H), 4.97 (m, 1H), 3.95 (m, 3H), 3.92 – 3.82 (m, 2H), 3.63 – 3.55 (m, 2H) ppm. ^{13}C-NMR (176 MHz, $(CD_3)_2SO$) δ 169.3, 156.3, 138.0, 137.1, 137.0, 135.7, 134.7, 129.7, 129.0, 128.1, 127.5, 122.8, 121.4, 119.6, 119.3, 114.9, 110.9, 110.8, 59.6, 42.3, 33.4 ppm. HRMS (m/z) Anal. Calc'd for $C_{23}H_{21}N_3O_2$ (M+H) $^+$: 372.1707, Found (M+H) $^+$: 372.1705.

A4B2: not obtained.

A4B3: not obtained.

A4B4: 53.3 mg, 13%. ^{1}H-NMR (700 MHz, $(CD_3)_2SO$) δ 9.55 – 6.14 (m, 13H), 5.11 – 4.96 (m, 1H), 3.99 – 3.68 (m, 8H), 3.68 – 3.55 (m, 2H) ppm. ^{13}C-NMR (176 MHz, $(CD_3)_2SO$) δ 169.6, 169.0, 167.3, 157.7, 156.5, 154.7, 137.9, 137.9, 137.3, 137.0, 136.8, 136.5, 136.1, 135.4, 135.2, 135.0, 134.9, 134.5, 134.0, 132.5, 130.0, 128.3, 127.4, 127.1, 125.6, 125.4, 123.3, 123.0, 122.7, 122.6, 122.3, 121.3, 121.1, 121.0, 121.0, 120.6, 120.4, 120.2,

120.1, 119.6, 118.5, 118.3, 116.6, 112.6, 112.6, 111.3, 111.0, 111.0, 110.8, 110.7, 110.6, 110.6, 110.5, 108.1, 107.0, 59.7, 59.6, 59.3, 42.6, 42.5, 42.4, 33.5, 33.4, 33.3, 33.2, 33.0, 32.9 ppm. HRMS (m/z) Anal. Calc'd for $C_{26}H_{24}N_4O_2$ (M+H) $^+$: 425.1972, Found (M+H) $^+$: 425.1975.

A4B5: 41.5 mg, 11%. ^{1}H-NMR (700 MHz, $(CD_3)_2SO$) δ 9.97 (s, 1H), 8.95 (m, 1H), 8.23 – 6.70 (m, 11H), 4.96 (m, 1H), 4.00 – 3.54 (m, 7H) ppm. ^{13}C-NMR (176 MHz, $(CD_3)_2SO$) δ 169.4, 167.1, 159.3, 159.1, 156.9, 153.8, 138.5, 137.0, 136.8, 136.8, 136.6, 135.8, 135.4, 134.9, 130.0, 129.6, 128.4, 127.5, 127.0, 126.9, 124.7, 122.9, 122.7, 121.3, 121.2, 119.5, 118.4, 118.1, 115.9, 115.9, 115.9, 111.3, 111.1, 110.9, 110.9, 110.7, 110.6, 59.5, 59.5, 42.3, 42.2, 33.5, 33.4 ppm. HRMS (m/z) Anal. Calc'd for $C_{23}H_{21}N_3O_3$ (M+H) $^+$: 388.1656, Found (M+H) $^+$: 388.1649.

A4B6: 149.5 mg, 37%. ^{1}H-NMR (700 MHz, $(CD_3)_2SO$) δ 8.97 (m, 1H), 8.22 – 7.68 (m, 4H), 7.56 (m, 1H), 7.37 – 6.97 (m, 6H), 4.96 (m, 1H), 3.99 – 3.78 (m, 8H), 3.64 – 3.53 (m, 2H) ppm. ^{13}C-NMR (176 MHz, $(CD_3)_2SO$) δ 169.4, 167.1, 160.7, 160.5, 156.7, 153.7, 138.0, 137.0, 136.8, 136.7, 136.3, 135.9, 135.4, 134.8, 129.8, 129.5, 128.5, 128.4, 128.4, 127.5, 125.0, 122.9, 122.8, 121.4, 121.3, 119.5, 118.4, 118.4, 114.5, 114.5, 112.4, 112.2, 110.9, 110.9, 110.7, 110.6, 59.9, 59.5, 59.5, 55.4, 55.4, 42.3, 42.2, 33.5, 33.4 ppm. HRMS (m/z) Anal. Calc'd for $C_{24}H_{23}N_3O_3$ (M+H) $^+$: 402.1812, Found (M+H) $^+$: 402.1811.

A4B7: 241.7 mg, 61%. ^{1}H-NMR (700 MHz, $(CD_3)_2SO$) δ 9.61 – 7.09 (m, 12H), 4.97 (m, 1H), 4.05 – 3.81 (m, 5H), 3.59 (m, 2H) ppm. ^{13}C-NMR (176 MHz, $(CD_3)_2SO$) δ 169.1, 166.8, 155.7, 152.7, 140.3, 140.3, 137.4, 137.1, 136.9, 136.6, 135.7, 135.1, 134.5, 134.0, 132.8, 132.8, 128.6, 128.4, 128.4, 127.5, 126.7, 123.1, 122.9, 121.6, 121.5, 120.4, 119.6, 118.9, 118.7, 118.5, 111.3, 111.0, 111.0, 110.8, 110.8, 59.5, 59.5, 42.4, 42.3, 33.6, 33.5 ppm. HRMS (m/z) Anal. Calc'd for $C_{24}H_{20}N_4O_2$ (M+H) $^+$: 397.1659, Found (M+H) $^+$: 397.1656.

A4B8: 230.1 mg, 55%. ^{1}H-NMR (700 MHz, $(CD_3)_2SO$) δ 9.03 (m, 1H), 8.37 – 7.21 (m, 11H), 4.98 (m, 1H), 3.97 (s, 3H), 3.94 – 3.84 (m, 2H), 3.67 – 3.55 (m, 2H) ppm. ^{13}C-NMR (176 MHz, $(CD_3)_2SO$) δ 169.1, 155.6, 147.4, 142.3, 137.5, 137.1, 135.1, 134.5, 128.9, 127.5, 124.1, 124.1, 122.9, 121.6, 120.6, 119.6, 119.3, 111.1, 110.8, 59.5, 42.3, 33.5 ppm. HRMS (m/z) Anal. Calc'd for $C_{23}H_{20}N_4O_4$ (M+H) $^+$: 417.1557, Found (M+H) $^+$: 417.1556.

A4B9: 107.6 mg, 29%. ^{1}H-NMR (700 MHz, $(CD_3)_2SO$) δ 9.53 – 8.53 (m, 3H), 8.31 – 7.41 (m, 7H), 7.38 – 7.21 (m, 2H), 4.95 (t, *J* = 5.7 Hz, 1H), 4.01 – 3.83 (m, 5H), 3.59 (m, 2H) ppm. ^{13}C-NMR (176 MHz, $(CD_3)_2SO$) δ 169.0, 155.5, 150.3, 142.7, 137.1, 134.8, 134.4, 127.4, 122.8, 121.8, 121.5, 119.5, 110.9, 110.8, 59.5, 42.2, 33.4 ppm. HRMS (m/z) Anal. Calc'd for $C_{22}H_{20}N_4O_2$ (M+H) $^+$: 373.1659, Found (M+H) $^+$: 373.1654.

A4B10: 150.3 mg, 40%. ^{1}H-NMR (700 MHz, $(CD_3)_2SO$) δ 9.44 – 8.58 (m, 4H), 8.27 – 7.42 (m, 5H), 7.29 (m, 2H), 4.92 (t, *J* = 5.7 Hz, 1H), 4.00 – 3.83 (m, 5H), 3.60 (m, 2H) ppm. ^{13}C-NMR (176 MHz, $(CD_3)_2SO$) δ 169.0, 158.1, 155.7, 155.5, 137.3, 137.1, 134.4, 130.7, 129.7, 127.4, 122.9, 121.5, 120.4, 119.6, 118.6, 111.0, 110.8, 59.4, 42.2, 33.4 ppm. HRMS (m/z) Anal. Calc'd for $C_{21}H_{19}N_5O_2$ (M+H) $^+$: 374.1612, Found (M+H) $^+$: 374.1614.

A4B11: 160.7 mg, 32%. ^{1}H-NMR (700 MHz, $(CD_3)_2SO$) δ 8.98 (m, 1H), 8.21 – 7.16 (m, 8H), 6.92 – 6.79 (m, 1H), 4.97 (m, 1H), 3.95 (s, 3H), 3.85 – 3.75 (m, 2H), 3.55 (m, 2H) ppm. ^{13}C-NMR (176 MHz, $(CD_3)_2SO$) δ 169.1, 155.9, 146.8, 138.3, 137.0, 136.9, 134.7, 132.1, 129.5, 127.5, 122.8, 121.4, 119.5, 119.1, 114.3, 110.9, 110.8, 80.1, 59.5, 42.3, 33.4 ppm. HRMS (m/z) Anal. Calc'd for $C_{21}H_{18}N_3O_2SI$ (M+H) $^+$: 504.0237, Found (M+H) $^+$: 504.0234.

A4B12: 162.6 mg, 38%. ^{1}H-NMR (700 MHz, $(CD_3)_2SO$) δ 9.09 (m, 1H), 8.38 – 7.44 (m, 9H), 7.40 – 7.18 (m, 2H), 5.07 (m, 1H), 4.04 – 3.83 (m, 5H), 3.61 (m, 2H) ppm. ^{13}C-NMR (176 MHz, $(CD_3)_2SO$) δ 168.8, 164.6, 154.7, 153.5, 137.9, 137.2, 134.9, 134.3, 129.0, 127.5, 127.0, 126.2, 123.1, 123.0, 122.9, 122.5, 121.6, 121.5, 119.5, 111.1, 110.9, 59.5, 42.4, 33.4 ppm. HRMS (m/z) Anal. Calc'd for $C_{24}H_{20}N_4O_2S$ (M+H) $^+$: 429.1380, Found (M+H) $^+$: 429.1350.

A5B1: 67.7 mg, 20%. ^{1}H-NMR (700 MHz, $(CD_3)_2SO$) δ 10.19 (s, 1H), 8.20 (d, *J* = 8.6 Hz, 2H), 7.98 (d, *J* = 15.8 Hz, 1H), 7.83 (d, *J* = 7.3 Hz, 2H), 7.50 – 7.38 (m, 3H), 7.26 (d, *J* = 15.8 Hz, 1H), 6.97 (s, 1H), 6.88 (d, *J* = 8.9 Hz, 2H), 4.94 (s, 1H), 3.84 (t, *J* = 5.6 Hz, 2H), 3.55 (m, 2H) ppm. ^{13}C-NMR (176 MHz, $(CD_3)_2SO$) δ 170.1, 159.8, 159.0, 138.9, 136.8, 135.4, 134.5, 129.9, 128.9, 128.2, 125.9, 125.8, 115.9, 114.8, 59.3, 42.3 ppm. HRMS (m/z) Anal. Calc'd for $C_{20}H_{18}N_2O_3$ $(M+H)^+$: 335.1390, Found $(M+H)^+$: 335.1378.

A5B2: 47.9 mg, 13%. ^{1}H-NMR (700 MHz, $(CD_3)_2SO$) δ 10.10 (s, 1H), 8.17 (d, *J* = 8.3 Hz, 2H), 7.90 (d, *J* = 15.5 Hz, 1H), 7.65 (d, *J* = 8.4 Hz, 2H), 6.94 – 6.82 (m, 4H), 6.76 (d, *J* = 8.4 Hz, 2H), 4.92 (s, 1H), 3.80 (t, *J* = 5.8 Hz, 2H), 3.55 (t, *J* = 5.8 Hz, 2H), 3.01 (s, 6H) ppm. ^{13}C-NMR (176 MHz, $(CD_3)_2SO$) δ 170.2, 159.6, 159.3, 151.5, 140.0, 137.2, 134.1, 129.9, 126.2, 123.5, 122.9, 115.8, 111.9, 108.3, 59.3, 42.2 ppm. HRMS (m/z) Anal. Calc'd for $C_{22}H_{23}N_3O_3$ $(M+H)^+$: 378.1812, Found $(M+H)^+$: 378.1817.

A5B3: 51.3 mg, 13%. ^{1}H-NMR (700 MHz, $(CD_3)_2SO$) δ 10.24 (m, 1H), 8.75 – 5.97 (m, 13H), 4.94 (s, 1H), 3.71 (m, 2H), 3.52 (m, 2H), 2.99 (m, 6H) ppm. ^{13}C-NMR (176 MHz, $(CD_3)_2SO$) δ 170.2, 159.5, 159.2, 150.9, 141.2, 140.5, 137.3, 134.2, 128.7, 126.3, 124.2, 124.0, 123.2, 115.9, 114.3, 112.2, 59.2, 42.2 ppm. HRMS (m/z) Anal. Calc'd for $C_{24}H_{25}N_3O_3$ $(M+H)^+$: 404.1969, Found $(M+H)^+$: 404.1975.

A5B4: 94.5 mg, 24%. ^{1}H-NMR (700 MHz, $(CD_3)_2SO$) δ 10.09 (s, 1H), 9.71 – 6.11 (m, 12H), 5.13 – 4.90 (m, 1H), 3.98 – 3.73 (m, 5H), 3.66 – 3.52 (m, 2H) ppm. ^{13}C-NMR (176 MHz, $(CD_3)_2SO$) δ 170.3, 160.1, 159.2, 138.0, 137.3, 135.3, 134.0, 133.5, 126.3, 125.4, 123.0, 122.7, 121.2, 120.4, 115.8, 112.5, 110.8, 107.7, 59.5, 42.4, 33.0 ppm. HRMS (m/z) Anal. Calc'd for $C_{23}H_{21}N_3O_3$ $(M+H)^+$: 388.1656, Found $(M+H)^+$: 388.1642.

A5B5: 53.5 mg, 15%. ^{1}H-NMR (700 MHz, $(CD_3)_2SO$) δ 10.17 (s, 2H), 8.17 (d, *J* = 8.7 Hz, 2H), 7.90 (d, *J* = 15.6 Hz, 1H), 7.67 (d, *J* = 8.6 Hz, 2H), 7.01 (d, *J* = 15.7 Hz, 1H), 6.90 – 6.81 (m, 5H), 4.92 (s, 1H), 3.80 (t, *J* = 5.6 Hz, 2H), 3.54 (t, *J* = 5.7 Hz, 2H) ppm. ^{13}C-NMR (176 MHz, $(CD_3)_2SO$) δ 170.2, 159.5, 159.4, 139.4, 137.0, 134.3, 130.2, 126.5, 126.0, 124.6, 115.9, 115.9, 110.8, 59.3, 42.2 ppm. HRMS (m/z) Anal. Calc'd for $C_{20}H_{18}N_2O_4$ $(M+H)^+$: 351.1339, Found $(M+H)^+$: 351.1330.

A5B6: 100.8 mg, 28%. ^{1}H-NMR (700 MHz, $(CD_3)_2SO$) δ 10.27 (s, 1H), 8.29 – 7.66 (m, 5H), 7.13 – 6.77 (m, 6H), 4.94 (s, 1H), 3.82 (m, 5H), 3.55 (m, 2H) ppm. ^{13}C-NMR (176 MHz, $(CD_3)_2SO$) δ 170.2, 169.6, 160.9, 160.2, 160.0, 159.6, 159.3, 158.0, 140.7, 139.0, 137.0, 136.5, 134.4, 134.3, 132.7, 130.0, 128.2, 128.1, 126.5, 126.1, 125.7, 125.0, 116.0, 115.9, 114.5, 113.3, 113.0, 112.0, 59.4, 59.1, 55.4, 55.4, 42.7, 42.3 ppm. HRMS (m/z) Anal. Calc'd for $C_{21}H_{20}N_2O_4$ $(M+H)^+$: 365.1496, Found $(M+H)^+$: 365.1500.

A5B7: 24.3 mg, 7%. ^{1}H-NMR (700 MHz, $(CD_3)_2SO$) δ 10.29 (s, 1H), 8.21 (d, *J* = 8.7 Hz, 2H), 8.06 – 7.98 (m, 3H), 7.93 (d, *J* = 8.4 Hz, 2H), 7.43 (d, *J* = 15.9 Hz, 1H), 7.02 (s, 1H), 6.88 (d, *J* = 8.9 Hz, 2H), 4.93 (s, 1H), 3.85 (t, *J* = 5.5 Hz, 2H), 3.55 (t, *J* = 5.5 Hz, 2H) ppm. ^{13}C-NMR (176 MHz, $(CD_3)_2SO$) δ 169.9, 160.1, 158.5, 139.9, 136.7, 136.6, 134.8, 132.7, 128.8, 126.9, 125.8, 118.8, 118.4, 116.0, 111.5, 59.3, 42.2 ppm. HRMS (m/z) Anal. Calc'd for $C_{21}H_{17}N_3O_3$ $(M+H)^+$: 360.1343, Found $(M+H)^+$: 360.1320.

A5B8: 135.8 mg, 36%. ^{1}H-NMR (700 MHz, $(CD_3)_2SO$) δ 10.29 (s, 1H), 8.29 (d, *J* = 8.7 Hz, 2H), 8.21 (d, *J* = 8.4 Hz, 2H), 8.12 – 8.03 (m, 3H), 7.47 (d, *J* = 15.8 Hz, 1H), 7.03 (s, 1H), 6.88 (d, *J* = 8.5 Hz, 2H), 4.97 (s, 1H), 3.86 (t, *J* = 5.5 Hz, 2H), 3.56 (m, 2H) ppm. ^{13}C-NMR (176 MHz, $(CD_3)_2SO$) δ 170.0, 160.2, 158.5, 147.6, 141.9, 136.8, 136.2, 134.9, 129.2, 127.2, 125.9, 124.1, 119.2, 116.1, 59.4, 42.3 ppm. HRMS (m/z) Anal. Calc'd for $C_{20}H_{17}N_3O_5$ $(M+H)^+$: 380.1241, Found $(M+H)^+$: 380.1230.

A5B9: 30.2 mg, 9%. ^{1}H-NMR (700 MHz, $(CD_3)_2SO$) δ 10.30 (s, 1H), 8.66 (s, 2H), 8.21 (d, *J* = 8.5 Hz, 2H), 7.92 (d, *J* = 15.8 Hz, 1H), 7.78 (d, *J* = 5.2 Hz, 2H), 7.50 (d, *J* = 15.8 Hz, 1H), 7.03 (s, 1H), 6.88 (d, *J* = 8.5 Hz, 2H), 4.96 (t, *J* = 5.7 Hz, 1H), 3.85 (t, *J* = 5.5 Hz, 2H), 3.55 (q, *J* = 5.3 Hz, 2H) ppm. ^{13}C-NMR (176 MHz, $(CD_3)_2SO$) δ 170.0, 160.1, 158.4, 150.4, 142.5, 136.7, 136.0, 134.9, 127.2, 125.8, 122.0, 119.5, 116.1, 59.4, 42.3 ppm. HRMS (m/z) Anal. Calc'd for $C_{19}H_{17}N_3O_3$ $(M+H)^+$: 336.1343, Found $(M+H)^+$: 336.1328.

A5B10: 17.7 mg, 5%. ^{1}H-NMR (700 MHz, $(CD_3)_2SO$) δ 10.30 (s, 1H), 8.66 (s, 2H), 8.21 (d, *J* = 8.5 Hz, 2H), 7.92 (d, *J* = 15.8 Hz, 1H), 7.78 (d, *J* = 5.2 Hz, 2H), 7.50 (d, *J* = 15.8 Hz, 1H), 7.03 (s, 1H), 6.88 (d, *J* = 8.5 Hz, 2H), 4.96 (t, *J* = 5.7 Hz, 1H), 3.85 (t, *J* = 5.5 Hz, 2H), 3.55 (q, *J* = 5.3 Hz, 2H) ppm. ^{13}C-NMR (176 MHz, $(CD_3)_2SO$) δ 170.0, 160.1, 158.4, 150.4, 142.5, 136.7, 136.0, 134.9, 127.2, 125.8, 122.0, 119.5, 116.1, 59.4, 42.3 ppm. HRMS (m/z) Anal. Calc'd for $C_{19}H_{17}N_3O_3$ (M+H) $^{+}$: 336.1343, Found (M+H) $^{+}$: 336.1328.

A5B11: 197.3 mg, 42%. ^{1}H-NMR (700 MHz, $(CD_3)_2SO$) δ 10.31 (s, 1H), 8.17 (d, *J* = 8.4 Hz, 2H), 8.06 (d, *J* = 15.5 Hz, 1H), 7.41 (d, *J* = 3.8 Hz, 1H), 7.30 (d, *J* = 3.8 Hz, 1H), 6.94 (s, 1H), 6.91 – 6.80 (m, 3H), 4.97 (s, 1H), 3.77 (t, *J* = 5.5 Hz, 2H), 3.52 (m, 2H) ppm. ^{13}C-NMR (176 MHz, $(CD_3)_2SO$) δ 170.0, 159.8, 158.6, 146.5, 138.4, 136.9, 134.6, 132.8, 130.5, 126.0, 125.8, 116.0, 114.1, 80.9, 59.4, 42.4 ppm. HRMS (m/z) Anal. Calc'd for $C_{15}H_{18}N_2O_3SI$ (M+H) $^{+}$: 466.9921, Found (M+H) $^{+}$: 466.9918.

A5B12: 130.0 mg, 33%. ^{1}H-NMR (700 MHz, $(CD_3)_2SO$) δ 10.31 (s, 1H), 8.24 (d, *J* = 8.3 Hz, 2H), 8.21 – 8.13 (m, 2H), 8.09 (d, *J* = 8.1 Hz, 1H), 7.67 – 7.47 (m, 3H), 7.10 (s, 1H), 6.91 (d, *J* = 8.7 Hz, 2H), 5.06 (t, *J* = 5.4 Hz, 1H), 3.85 (t, *J* = 5.4 Hz, 2H), 3.58 (q, *J* = 5.4 Hz, 2H) ppm. ^{13}C-NMR (176 MHz, $(CD_3)_2SO$) δ 169.7, 164.2, 160.3, 157.7, 153.4, 136.6, 135.1, 135.0, 130.1, 128.0, 127.0, 126.4, 125.7, 123.3, 122.6, 122.6, 116.1, 59.3, 42.5 ppm. HRMS (m/z) Anal. Calc'd for $C_{21}H_{17}N_3O_3S$ (M+H) $^{+}$: 392.1063, Found (M+H) $^{+}$: 392.1074.

A6B1: 160.2 mg, 46%. ^{1}H-NMR (700 MHz, $(CD_3)_2SO$) δ 8.30 (d, *J* = 8.7 Hz, 2H), 7.99 (d, *J* = 15.7 Hz, 1H), 7.82 (d, *J* = 7.3 Hz, 2H), 7.51 – 7.40 (m, 3H), 7.27 (d, *J* = 15.8 Hz, 1H), 7.09 – 6.98 (m, 3H), 4.96 (s, 1H), 3.84 (m, 5H), 3.56 (t, *J* = 5.6 Hz, 2H) ppm. ^{13}C-NMR (176 MHz, $(CD_3)_2SO$) δ 170.2, 160.9, 159.7, 139.3, 137.6, 135.3, 134.2, 130.1, 129.0, 128.3, 127.4, 125.2, 114.8, 114.5, 59.4, 55.4, 42.3 ppm. HRMS (m/z) Anal. Calc'd for $C_{21}H_{20}N_2O_3$ (M+H) $^{+}$: 349.1547, Found (M+H) $^{+}$: 349.1549.

A6B2: not obtained.

A6B3: not obtained.

A6B4: 34.3 mg, 9%. ^{1}H-NMR (700 MHz, $(CD_3)_2SO$) δ 10.04 (s, 1H), 8.28 (d, *J* = 8.4 Hz, 2H), 7.92 (d, *J* = 15.6 Hz, 1H), 7.68 (d, *J* = 8.3 Hz, 2H), 7.09 – 6.99 (m, 3H), 6.94 (s, 1H), 6.85 (d, *J* = 8.3 Hz, 2H), 4.86 (s, 1H), 3.82 (m, 5H), 3.55 (t, *J* = 5.6 Hz, 2H). ppm. ^{13}C-NMR (176 MHz, $(CD_3)_2SO$) δ 170.1, 160.6, 160.0, 159.6, 139.7, 137.7, 133.9, 130.2, 127.6, 126.5, 123.9, 115.9, 114.4, 110.8, 59.3, 55.3, 42.2 ppm. HRMS (m/z) Anal. Calc'd for $C_{21}H_{20}N_2O_4$ (M+H) $^{+}$: 365.1496, Found (M+H) $^{+}$: 365.1507.

A6B5: 44.0 mg, 12%. ^{1}H-NMR (700 MHz, $(CD_3)_2SO$) δ 8.30 (d, *J* = 8.8 Hz, 2H), 7.96 (d, *J* = 15.7 Hz, 1H), 7.79 (d, *J* = 8.8 Hz, 2H), 7.11 (d, *J* = 15.7 Hz, 1H), 7.04 (m, 4H), 6.97 (s, 1H), 4.93 (t, *J* = 5.7 Hz, 1H), 3.83 (m, 8H), 3.56 (q, *J* = 5.7 Hz, 2H) ppm. ^{13}C-NMR (176 MHz, $(CD_3)_2SO$) δ 170.1, 160.9, 160.7, 159.9, 139.2, 137.7, 134.0, 130.0, 128.0, 127.5, 124.2, 114.5, 114.4, 112.0, 59.3, 55.4, 55.3, 42.2 ppm. HRMS (m/z) Anal. Calc'd for $C_{22}H_{22}N_2O_4$ (M+H) $^{+}$: 379.1652, Found (M+H) $^{+}$: 379.1657.

A6B6: 157.3 mg, 42%. ^{1}H-NMR (700 MHz, $(CD_3)_2SO$) δ 8.32 (d, *J* = 8.8 Hz, 2H), 8.06 – 7.96 (m, 3H), 7.93 (d, *J* = 8.2 Hz, 2H), 7.44 (d, *J* = 15.8 Hz, 1H), 7.15 – 6.97 (m, 3H), 4.94 (t, *J* = 5.8 Hz, 1H), 3.85 (m, 5H), 3.56 (q, *J* = 5.6 Hz, 2H) ppm. ^{13}C-NMR (176 MHz, $(CD_3)_2SO$) δ 169.9, 161.0, 159.1, 139.8, 137.5, 136.9, 134.4, 132.7, 128.8, 127.3, 126.2, 118.7, 118.3, 114.5, 111.6, 59.3, 55.4, 42.3 ppm. HRMS (m/z) Anal. Calc'd for $C_{22}H_{19}N_3O_3$ (M+H) $^{+}$: 374.1499, Found (M+H) $^{+}$: 374.1465.

A6B7: 234.7 mg, 60%. ^{1}H-NMR (700 MHz, $(CD_3)_2SO$) δ 8.31 (m, 4H), 8.15 – 8.04 (m, 3H), 7.49 (d, *J* = 15.8 Hz, 1H), 7.14 – 7.01 (m, 3H), 4.95 (t, *J* = 5.7 Hz, 1H), 3.86 (m, 5H), 3.57 (q, *J* = 5.6 Hz, 2H) ppm. ^{13}C-NMR (176 MHz, $(CD_3)_2SO$) δ 169.9, 161.1, 159.0, 147.6, 141.8, 137.5, 136.4, 134.4, 129.2, 127.3, 126.4, 124.0, 119.2, 114.5, 59.3, 55.4, 42.3 ppm. HRMS (m/z) Anal. Calc'd for $C_{21}H_{19}N_3O_5$ (M+H) $^{+}$: 394.1397, Found (M+H) $^{+}$: 394.1373.

A6B8: 113.6 mg, 33%. ^{1}H-NMR (700 MHz, $(CD_3)_2SO$) δ 8.66 (d, *J* = 6.0 Hz, 2H), 8.32 (d, *J* = 8.8 Hz, 2H), 7.94 (d, *J* = 15.8 Hz, 1H), 7.78 (d, *J* = 6.2 Hz, 2H), 7.52 (d, *J* = 15.8 Hz, 1H), 7.14 – 7.00 (m, 3H), 4.95 (t, *J* = 5.7 Hz, 1H), 3.85 (m, 5H), 3.56 (q, *J* = 5.5 Hz, 2H) ppm. ^{13}C-NMR (176 MHz, $(CD_3)_2SO$) δ 169.9, 161.1, 159.0, 150.3, 142.3, 137.4, 136.2, 134.4, 127.2, 126.5, 122.0, 119.4, 114.5, 59.3, 55.4, 42.3 ppm. HRMS (m/z) Anal. Calc'd for $C_{20}H_{19}N_3O_3$ (M+H) $^+$: 350.1499, Found (M+H) $^+$: 350.1504.

A6B9: 203.6 mg, 58%. ^{1}H-NMR (700 MHz, $(CD_3)_2SO$) δ 9.27 (s, 2H), 9.18 (s, 1H), 8.32 (d, *J* = 8.4 Hz, 2H), 7.98 (d, *J* = 16.0 Hz, 1H), 7.53 (d, *J* = 16.0 Hz, 1H), 7.13 – 7.01 (m, 3H), 4.91 (t, *J* = 5.8 Hz, 1H), 3.86 (m, 5H), 3.56 (q, *J* = 5.6 Hz, 2H) ppm. ^{13}C-NMR (176 MHz, $(CD_3)_2SO$) δ 169.9, 161.1, 158.9, 158.3, 156.0, 137.4, 134.4, 132.2, 129.7, 129.6, 129.4, 127.8, 127.2, 126.3, 118.4, 114.5, 59.2, 55.4, 42.3 ppm. HRMS (m/z) Anal. Calc'd for $C_{19}H_{18}N_4O_3$ (M+H) $^+$: 351.1452, Found (M+H) $^+$: 351.1454.

A6B10: 24.3 mg, 5%. ^{1}H-NMR (700 MHz, $(CD_3)_2SO$) δ 8.27 (d, *J* = 8.8 Hz, 2H), 8.07 (d, *J* = 15.5 Hz, 1H), 7.42 (d, *J* = 3.8 Hz, 1H), 7.30 (d, *J* = 3.8 Hz, 1H), 7.04 (d, *J* = 9.0 Hz, 2H), 6.99 (s, 1H), 6.88 (d, *J* = 15.5 Hz, 1H), 4.97 (t, *J* = 5.6 Hz, 1H), 3.83 (s, 3H), 3.77 (t, *J* = 5.5 Hz, 2H), 3.53 (q, *J* = 5.5 Hz, 2H) ppm. ^{13}C-NMR (176 MHz, $(CD_3)_2SO$) δ 170.0, 160.9, 159.2, 146.4, 138.4, 137.6, 134.2, 132.9, 130.8, 127.5, 125.1, 114.5, 114.0, 81.1, 59.3, 55.4, 42.4 ppm. HRMS (m/z) Anal. Calc'd for $C_{19}H_{17}N_2O_3SI$ (M+H) $^+$: 481.0077, Found (M+H) $^+$: 481.0065.

A6B11: 235.5 mg, 58%. ^{1}H-NMR (700 MHz, $(CD_3)_2SO$) δ 8.42 (m, 2H), 8.23 – 7.92 (m, 3H), 7.66 – 7.13 (m, 4H), 7.07 (m, 2H), 5.07 (t, *J* = 5.5 Hz, 1H), 3.92 – 3.79 (m, 5H), 3.58 (q, *J* = 5.4 Hz, 2H) ppm. ^{13}C-NMR (176 MHz, $(CD_3)_2SO$) δ 169.7, 164.1, 161.3, 158.3, 153.4, 137.3, 135.1, 134.6, 130.4, 127.3, 127.2, 127.1, 126.4, 123.3, 122.6, 122.5, 114.6, 59.3, 55.4, 42.5 ppm. HRMS (m/z) Anal. Calc'd for $C_{22}H_{19}N_3O_3S$ (M+H) $^+$: 406.1220, Found (M+H) $^+$: 406.1220.

A6B12: 177.6 mg, 44%. ^{1}H-NMR (700 MHz, $(CD_3)_2SO$) δ 9.65 – 6.12 (m, 12H), 5.15 – 4.87 (m, 1H), 3.92 – 3.74 (m, 8H), 3.58 (m, 2H) ppm. ^{13}C-NMR (176 MHz, $(CD_3)_2SO$) δ 170.4, 160.8, 160.4, 138.1, 138.0, 135.4, 133.9, 133.7, 127.9, 125.5, 122.9, 122.4, 121.4, 120.4, 114.4, 112.5, 110.9, 107.6, 59.5, 55.4, 42.5, 33.1 ppm. HRMS (m/z) Anal. Calc'd for $C_{24}H_{23}N_3O_3$ (M+H) $^+$: 402.1812, Found (M+H) $^+$: 402.1797.

A7B1: 144.9 mg, 42%. ^{1}H-NMR (700 MHz, $(CD_3)_2SO$) δ 8.49 (d, *J* = 8.4 Hz, 2H), 8.11 (d, *J* = 15.7 Hz, 1H), 7.91 (d, *J* = 8.3 Hz, 2H), 7.86 (d, *J* = 6.8 Hz, 2H), 7.48 (m, 3H), 7.32 (d, *J* = 15.8 Hz, 1H), 7.08 (s, 1H), 4.96 (t, *J* = 5.7 Hz, 1H), 3.87 (t, *J* = 5.5 Hz, 2H), 3.57 (q, *J* = 5.6 Hz, 2H) ppm. ^{13}C-NMR (176 MHz, $(CD_3)_2SO$) δ 170.1, 162.9, 141.7, 141.2, 139.1, 135.0, 132.4, 132.3, 130.5, 129.0, 128.6, 121.7, 118.9, 114.4, 111.2, 59.2, 42.5 ppm. HRMS (m/z) Anal. Calc'd for $C_{21}H_{17}N_3O_2$ (M+H) $^+$: 344.1394, Found (M+H) $^+$: 344.1365.

A7B2: 37.2 mg, 10%. ^{1}H-NMR (700 MHz, $(CD_3)_2SO$) δ 8.46 (d, *J* = 8.4 Hz, 2H), 8.05 (d, *J* = 15.4 Hz, 1H), 7.88 (d, *J* = 8.4 Hz, 2H), 7.69 (d, *J* = 8.9 Hz, 2H), 6.96 (d, *J* = 15.4 Hz, 1H), 6.91 (s, 1H), 6.78 (d, *J* = 8.9 Hz, 2H), 4.93 (t, *J* = 5.7 Hz, 1H), 3.84 (t, *J* = 5.6 Hz, 2H), 3.57 (q, *J* = 5.7 Hz, 2H), 3.03 (s, 6H) ppm. ^{13}C-NMR (176 MHz, $(CD_3)_2SO$) δ 170.2, 163.4, 152.0, 142.7, 142.2, 139.7, 132.3, 131.9, 130.6, 122.5, 119.0, 118.9, 111.9, 110.4, 107.4, 59.2, 42.3 ppm. HRMS (m/z) Anal. Calc'd for $C_{24}H_{28}N_4O_2$ (M+H) $^+$: 387.1816, Found (M+H) $^+$: 387.1806.

A7B3: 53.0 mg, 13%. ^{1}H-NMR (700 MHz, $(CD_3)_2SO$) δ 8.52 – 6.16 (m, 13H), 4.94 (m, 1H), 3.74 (m, 2H), 3.54 (m, 2H), 3.02 (s, 6H) ppm. ^{13}C-NMR (176 MHz, $(CD_3)_2SO$) δ 170.2, 163.0, 151.2, 143.9, 142.4, 142.3, 139.6, 132.4, 132.0, 129.2, 123.7, 123.0, 119.7, 119.1, 113.5, 112.1, 110.7, 59.1, 42.4 ppm. HRMS (m/z) Anal. Calc'd for $C_{25}H_{24}N_4O_2$ (M+H) $^+$: 413.1972, Found (M+H) $^+$: 413.1994.

A7B4: 105.4 mg, 27%. ^{1}H-NMR (700 MHz, $(CD_3)_2SO$) δ 9.68 – 6.06 (m, 12H), 5.04 (m, 1H), 3.91 – 3.76 (m, 5H), 3.60 (m, 2H) ppm. ^{13}C-NMR (176 MHz, $(CD_3)_2SO$) δ 170.4, 164.0, 142.3, 139.8, 138.2, 136.6, 136.2, 132.4, 131.9, 125.5, 123.1, 121.7, 120.5, 119.1, 118.5, 112.7, 111.1, 110.4, 106.9, 59.4, 42.6, 33.2 ppm. HRMS (m/z) Anal. Calc'd for $C_{24}H_{20}N_4O_2$ (M+H) $^+$: 397.1659, Found (M+H) $^+$: 397.1672.

A7B5: 76.5 mg, 21%. ^{1}H-NMR (700 MHz, $(CD_3)_2SO$) δ 10.15 (s, 1H), 8.46 (d, *J* = 8.4 Hz, 2H), 8.04 (d, *J* = 15.6 Hz, 1H), 7.89 (d, *J* = 8.5 Hz, 2H), 7.72 (d, *J* = 8.6 Hz, 2H), 7.07 (d, *J* = 15.6 Hz, 1H), 6.99 (s, 1H), 6.87 (d, *J* = 8.6 Hz, 2H), 4.94 (s, 1H), 3.84 (t, *J* = 5.5 Hz, 2H), 3.56 (t, *J* = 5.5 Hz, 2H) ppm. ^{13}C-NMR (176 MHz, $(CD_3)_2SO$) δ 170.2, 163.3, 160.1, 142.0, 141.9, 139.4, 132.4, 132.1, 130.8, 126.3, 120.3, 119.0, 116.0, 110.8, 110.3, 59.2, 42.4 ppm. HRMS (m/z) Anal. Calc'd for $C_{21}H_{17}N_3O_3$ $(M+H)^+$: 360.1343, Found $(M+H)^+$: 360.1340.

A7B6: 55.6 mg, 15%. ^{1}H-NMR (700 MHz, $(CD_3)_2SO$) δ 8.71 – 6.15 (m, 11H), 4.96 (m, 1H), 3.91 – 3.49 (m, 7H) ppm. ^{13}C-NMR (176 MHz, $(CD_3)_2SO$) δ 170.2, 163.2, 161.4, 141.9, 141.4, 139.4, 132.4, 132.2, 130.6, 127.8, 120.8, 119.0, 114.6, 111.5, 111.0, 59.2, 55.5, 42.5 ppm. HRMS (m/z) Anal. Calc'd for $C_{22}H_{19}N_3O_3$ $(M+H)^+$: 374.1499, Found $(M+H)^+$: 374.1501.

A7B7: 217.4 mg, 56%. ^{1}H-NMR (700 MHz, $(CD_3)_2SO$) δ 8.20 (d, *J* = 8.4 Hz, 2H), 8.00 (d, *J* = 8.0 Hz, 2H), 7.92 (m, 3H), 7.42 (d, *J* = 15.8 Hz, 1H), 7.00 (s, 1H), 6.79 (d, *J* = 8.6 Hz, 2H), 4.92 (t, *J* = 5.7 Hz, 1H), 3.85 (t, *J* = 5.7 Hz, 2H), 3.55 (q, *J* = 5.8 Hz, 2H), 3.05 (s, 6H) ppm. ^{13}C-NMR (176 MHz, $(CD_3)_2SO$) δ 169.6, 156.5, 151.5, 140.1, 135.5, 135.1, 134.5, 132.7, 128.6, 127.8, 122.1, 118.8, 118.6, 111.8, 111.2, 59.4, 42.2 ppm. HRMS (m/z) Anal. Calc'd for $C_{23}H_{22}N_4O_2$ $(M+H)^+$: 388.1894, Found $(M+H)^+$: 388.1860.

A7B8: 148.2 mg, 38%. ^{1}H-NMR (700 MHz, $(CD_3)_2SO$) δ 8.50 (d, *J* = 8.5 Hz, 2H), 8.31 (d, *J* = 8.8 Hz, 2H), 8.18 (d, *J* = 15.8 Hz, 1H), 8.13 (d, *J* = 8.8 Hz, 2H), 7.91 (d, *J* = 8.4 Hz, 2H), 7.53 (d, *J* = 15.8 Hz, 1H), 7.14 (s, 1H), 4.97 (t, *J* = 5.7 Hz, 1H), 3.90 (t, *J* = 5.3 Hz, 2H), 3.58 (q, *J* = 5.6 Hz, 2H) ppm. ^{13}C-NMR (176 MHz, $(CD_3)_2SO$) δ 170.0, 162.4, 147.9, 141.6, 141.4, 139.0, 138.4, 132.5, 132.5, 129.5, 124.1, 123.1, 118.9, 118.8, 111.5, 59.2, 42.5 ppm. HRMS (m/z) Anal. Calc'd for $C_{21}H_{16}N_4O_4$ $(M+H)^+$: 389.1244, Found $(M+H)^+$: 389.1234.

A7B9: 74.3 mg, 22%. ^{1}H-NMR (700 MHz, $(CD_3)_2SO$) δ 8.78 – 6.90 (m, 11H), 4.97 (t, *J* = 5.7 Hz, 1H), 3.94 – 3.48 (m, 4H) ppm. ^{13}C-NMR (176 MHz, $(CD_3)_2SO$) δ 170.0, 162.4, 150.5, 142.1, 141.5, 138.9, 138.2, 132.5, 132.5, 123.2, 122.2, 119.1, 118.9, 111.6, 59.2, 42.6 ppm. HRMS (m/z) Anal. Calc'd for $C_{20}H_{16}N_4O_2$ $(M+H)^+$: 345.1346, Found $(M+H)^+$: 345.1344.

A7B10: 24.5 mg, 7%. ^{1}H-NMR (700 MHz, $(CD_3)_2SO$) δ 9.29 (s, 2H), 9.20 (s, 1H), 8.48 (d, *J* = 8.1 Hz, 2H), 8.08 (d, *J* = 15.9 Hz, 1H), 7.91 (d, *J* = 8.1 Hz, 2H), 7.57 (d, *J* = 15.9 Hz, 1H), 7.14 (s, 1H), 4.94 (t, *J* = 5.8 Hz, 1H), 3.89 (t, *J* = 5.4 Hz, 2H), 3.58 (q, *J* = 5.4 Hz, 2H) ppm. ^{13}C-NMR (176 MHz, $(CD_3)_2SO$) δ 169.9, 162.2, 158.6, 156.2, 141.5, 138.9, 134.2, 132.4, 129.1, 123.0, 118.8, 118.1, 111.5, 59.1, 42.5 ppm. HRMS (m/z) Anal. Calc'd for $C_{19}H_{15}N_5O_2$ $(M+H)^+$: 346.1299, Found $(M+H)^+$: 346.1298.

A7B11: 197.1 mg, 41%. ^{1}H-NMR (700 MHz, $(CD_3)_2SO$) δ 8.61 – 8.37 (m, 2H), 8.23 – 7.98 (m, 1H), 7.88 (m, 2H), 7.51 – 7.33 (m, 2H), 7.03 (m, 1H), 6.95 – 6.34 (m, 1H), 4.98 (m, 1H), 3.85 – 3.73 (m, 2H), 3.54 (m, 2H) ppm. ^{13}C-NMR (176 MHz, $(CD_3)_2SO$) δ 170.0, 162.5, 146.2, 141.7, 139.2, 138.6, 133.7, 132.7, 132.5, 132.3, 121.6, 119.0, 113.5, 111.2, 82.4, 59.2, 42.6 ppm. HRMS (m/z) Anal. Calc'd for $C_{19}H_{14}N_3O_2SI$ $(M+H)^+$: 475.9924, Found $(M+H)^+$: 475.9935.

A7B12: 146.7 mg, 37%. ^{1}H-NMR (700 MHz, $(CD_3)_2SO$) δ 8.51 (d, *J* = 8.4 Hz, 2H), 8.28 (d, *J* = 15.5 Hz, 1H), 8.19 (d, *J* = 7.9 Hz, 1H), 8.11 (d, *J* = 8.1 Hz, 1H), 7.94 (d, *J* = 8.4 Hz, 2H), 7.64 (d, *J* = 15.5 Hz, 1H), 7.57 (m, 2H), 7.20 (s, 1H), 5.08 (t, *J* = 5.5 Hz, 1H), 3.88 (t, *J* = 5.3 Hz, 2H), 3.59 (q, *J* = 5.4 Hz, 2H) ppm. ^{13}C-NMR (176 MHz, $(CD_3)_2SO$) δ 169.8, 163.8, 161.7, 153.4, 141.3, 138.8, 135.3, 132.6, 132.5, 132.2, 127.1, 126.6, 123.9, 123.4, 122.6, 122.0, 118.8, 111.6, 59.1, 42.7 ppm. HRMS (m/z) Anal. Calc'd for $C_{22}H_{16}N_4O_2S$ $(M+H)^+$: 401.1067, Found $(M+H)^+$: 401.1060.

A8B1: 105.5 mg, 29%. ^{1}H-NMR (700 MHz, $(CD_3)_2SO$) δ 8.57 (d, *J* = 8.9 Hz, 2H), 8.30 (d, *J* = 8.9 Hz, 2H), 8.16 (d, *J* = 15.7 Hz, 1H), 7.87 (d, *J* = 7.0 Hz, 2H), 7.49 (m, 3H), 7.33 (d, *J* = 15.7 Hz, 1H), 7.12 (s, 1H), 4.97 (t, *J* = 5.7 Hz, 1H), 3.88 (t, *J* = 5.5 Hz, 2H), 3.58 (q, *J* = 5.6 Hz, 2H) ppm. ^{13}C-NMR (176 MHz, $(CD_3)_2SO$) δ 170.2,

163.4, 147.0, 142.2, 141.7, 141.2, 135.1, 132.8, 130.6, 129.1, 128.7, 123.8, 121.0, 114.3, 59.2, 42.5 ppm. HRMS (m/z) Anal. Calc'd for $C_{20}H_{17}N_3O_4$ (M+H) $^+$: 364.1292, Found (M+H) $^+$: 364.1318.

A8B2: 37.9 mg, 9%. ^{1}H-NMR (700 MHz, $(CD_3)_2SO$) δ 8.54 (d, *J* = 8.8 Hz, 2H), 8.27 (d, *J* = 9.0 Hz, 2H), 8.11 (d, *J* = 15.4 Hz, 1H), 7.70 (d, *J* = 8.8 Hz, 2H), 6.99 – 6.92 (m, 2H), 6.78 (d, *J* = 8.9 Hz, 2H), 4.94 (t, *J* = 5.7 Hz, 1H), 3.84 (t, *J* = 5.6 Hz, 2H), 3.57 (q, *J* = 5.7 Hz, 2H), 3.03 (s, 6H) ppm. ^{13}C-NMR (176 MHz, $(CD_3)_2SO$) δ 170.3, 164.0, 152.0, 146.5, 143.2, 142.8, 141.8, 132.2, 130.7, 123.7, 122.5, 118.0, 111.9, 107.2, 59.2, 42.4 ppm. HRMS (m/z) Anal. Calc'd for $C_{22}H_{22}N_4O_4$ (M+H) $^+$: 407.1714, Found (M+H) $^+$: 407.1737.

A8B3: 72.9 mg, 17%. ^{1}H-NMR (700 MHz, $(CD_3)_2SO$) δ 8.76 – 6.12 (m, 13H), 5.11 – 4.82 (m, 1H), 3.83 (m, 2H), 3.66 – 3.51 (m, 2H), 3.01 (m, 6H) ppm. ^{13}C-NMR (176 MHz, $(CD_3)_2SO$) δ 170.1, 170.0, 163.4, 162.9, 151.2, 147.9, 147.2, 146.6, 144.3, 142.8, 142.7, 142.0, 141.7, 141.4, 141.0, 138.7, 133.0, 132.3, 129.6, 129.3, 129.2, 124.1, 123.8, 123.7, 123.7, 123.6, 122.9, 122.3, 118.8, 113.4, 112.1, 59.1, 59.0, 42.5, 42.4, 40.0 ppm. HRMS (m/z) Anal. Calc'd for $C_{24}H_{24}N_4O_4$ (M+H) $^+$: 433.1870, Found (M+H) $^+$: 433.1897.

A8B4: 109.8 mg, 26%. ^{1}H-NMR (700 MHz, $(CD_3)_2SO$) δ 8.54 (d, *J* = 8.6 Hz, 2H), 8.40 (d, *J* = 15.4 Hz, 1H), 8.27 (d, *J* = 8.8 Hz, 2H), 8.16 – 8.05 (m, 2H), 7.58 (d, *J* = 8.1 Hz, 1H), 7.32 (m, 2H), 7.17 – 6.85 (m, 2H), 5.08 (t, *J* = 5.5 Hz, 1H), 3.87 (m, 5H), 3.62 (q, *J* = 5.5 Hz, 2H) ppm. ^{13}C-NMR (176 MHz, $(CD_3)_2SO$) δ 170.4, 164.5, 146.4, 142.9, 142.0, 138.2, 136.6, 136.6, 132.2, 125.5, 123.8, 123.2, 121.7, 120.5, 117.6, 112.8, 111.1, 106.7, 59.4, 42.6, 33.2 ppm. HRMS (m/z) Anal. Calc'd for $C_{23}H_{20}N_4O_4$ (M+H) $^+$: 417.1557, Found (M+H) $^+$: 417.1543.

A8B5: 65.9 mg, 17%. ^{1}H-NMR (700 MHz, $(CD_3)_2SO$) δ 10.21 (s, 1H), 8.55 (d, *J* = 8.5 Hz, 2H), 8.28 (d, *J* = 8.5 Hz, 2H), 8.10 (d, *J* = 15.6 Hz, 1H), 7.73 (d, *J* = 8.2 Hz, 2H), 7.07 (d, *J* = 15.5 Hz, 1H), 7.03 (s, 1H), 6.87 (d, *J* = 8.3 Hz, 2H), 4.95 (s, 1H), 3.85 (t, *J* = 5.6 Hz, 2H), 3.57 (m, 2H) ppm. ^{13}C-NMR (176 MHz, $(CD_3)_2SO$) δ 170.2, 163.8, 160.2, 146.7, 142.5, 142.3, 141.5, 132.5, 130.9, 126.3, 123.7, 119.5, 116.0, 110.2, 59.2, 42.4 ppm. HRMS (m/z) Anal. Calc'd for $C_{20}H_{17}N_3O_5$ (M+H) $^+$: 380.1241, Found (M+H) $^+$: 380.1261.

A8B6: 83.6 mg, 21%. ^{1}H-NMR (700 MHz, $(CD_3)_2SO$) δ 10.21 (s, 1H), 8.55 (d, *J* = 8.5 Hz, 2H), 8.28 (d, *J* = 8.5 Hz, 2H), 8.10 (d, *J* = 15.6 Hz, 1H), 7.73 (d, *J* = 8.2 Hz, 2H), 7.07 (d, *J* = 15.5 Hz, 1H), 7.03 (s, 1H), 6.87 (d, *J* = 8.3 Hz, 2H), 4.95 (s, 1H), 3.85 (t, *J* = 5.6 Hz, 2H), 3.57 (m, 2H) ppm. ^{13}C-NMR (176 MHz, $(CD_3)_2SO$) δ 170.2, 163.8, 160.2, 146.7, 142.5, 142.3, 141.5, 132.5, 130.9, 126.3, 123.7, 119.5, 116.0, 110.2, 59.2, 42.4 ppm. HRMS (m/z) Anal. Calc'd for $C_{21}H_{19}N_3O_5$ (M+H) $^+$: 394.1397, Found (M+H) $^+$: 394.1426.

A8B7: 34.0 mg, 9%. ^{1}H-NMR (700 MHz, $(CD_3)_2SO$) δ 8.58 (d, *J* = 8.5 Hz, 2H), 8.29 (d, *J* = 8.5 Hz, 2H), 8.18 (d, *J* = 15.5 Hz, 1H), 8.06 (d, *J* = 8.1 Hz, 2H), 7.96 (d, *J* = 8.1 Hz, 2H), 7.49 (d, *J* = 15.8 Hz, 1H), 7.17 (s, 1H), 4.97 (t, *J* = 5.7 Hz, 1H), 3.89 (t, *J* = 5.4 Hz, 2H), 3.58 (q, *J* = 5.4 Hz, 2H) ppm. ^{13}C-NMR (176 MHz, $(CD_3)_2SO$) δ 170.0, 163.0, 161.4, 147.1, 142.0, 141.0, 139.5, 139.3, 132.9, 132.8, 132.3, 129.1, 128.1, 123.7, 122.1, 118.7, 117.9, 112.1, 59.2, 42.5 ppm. HRMS (m/z) Anal. Calc'd for $C_{21}H_{16}N_4O_4$ (M+H) $^+$: 389.1244, Found (M+H) $^+$: 389.1254.

A8B8: 92.4 mg, 23%. ^{1}H-NMR (700 MHz, $(CD_3)_2SO$) δ 8.59 (d, *J* = 8.5 Hz, 2H), 8.36 – 8.21 (m, 5H), 8.14 (d, *J* = 8.4 Hz, 2H), 7.54 (d, *J* = 15.8 Hz, 1H), 7.20 (s, 1H), 5.00 (t, *J* = 5.7 Hz, 1H), 3.91 (t, *J* = 5.4 Hz, 2H), 3.58 (q, *J* = 5.6 Hz, 2H) ppm. ^{13}C-NMR (176 MHz, $(CD_3)_2SO$) δ 170.1, 162.9, 148.0, 147.2, 142.0, 141.4, 141.0, 138.7, 133.0, 129.6, 124.2, 123.8, 122.3, 118.7, 59.2, 42.6 ppm. HRMS (m/z) Anal. Calc'd for $C_{20}H_{16}N_4O_6$ (M+H) $^+$: 409.1143, Found (M+H) $^+$: 409.1136.

A8B9: 72.6 mg, 20%. ^{1}H-NMR (700 MHz, $(CD_3)_2SO$) δ 8.69 (d, *J* = 5.3 Hz, 2H), 8.58 (d, *J* = 8.8 Hz, 2H), 8.30 (d, *J* = 8.9 Hz, 2H), 8.09 (d, *J* = 15.8 Hz, 1H), 7.81 (d, *J* = 6.1 Hz, 2H), 7.57 (d, *J* = 15.8 Hz, 1H), 7.20 (s, 1H), 4.98 (t, *J* = 5.7 Hz, 1H), 3.90 (t, *J* = 5.4 Hz, 2H), 3.58 (q, *J* = 5.5 Hz, 2H) ppm. ^{13}C-NMR (176 MHz, $(CD_3)_2SO$) δ 170.0, 162.8, 150.5, 147.2, 142.0, 141.9, 140.9, 138.5, 133.0, 123.8, 122.4, 122.2, 119.0, 59.1, 42.5 ppm. HRMS (m/z) Anal. Calc'd for $C_{19}H_{16}N_4O_4$ (M+H) $^+$: 365.1244, Found (M+H) $^+$: 365.1243.

A8B10: 36.6 mg, 10%. ^{1}H-NMR (700 MHz, $(CD_3)_2SO$) δ 8.69 (d, *J* = 5.3 Hz, 2H), 8.58 (d, *J* = 8.8 Hz, 2H), 8.30 (d, *J* = 8.9 Hz, 2H), 8.09 (d, *J* = 15.8 Hz, 1H), 7.81 (d, *J* = 6.1 Hz, 2H), 7.57 (d, *J* = 15.8 Hz, 1H), 7.20 (s, 1H),

4.98 (t, *J* = 5.7 Hz, 1H), 3.90 (t, *J* = 5.4 Hz, 2H), 3.58 (q, *J* = 5.5 Hz, 2H) ppm. ^{13}C-NMR (176 MHz, $(CD_3)_2SO$) δ 170.0, 162.8, 150.5, 147.2, 142.0, 141.9, 140.9, 138.5, 133.0, 123.8, 122.4, 122.2, 119.0, 59.1, 42.5 ppm. HRMS (m/z) Anal. Calc'd for $C_{18}H_{15}N_5O_4$ (M+H) $^+$: 366.1197, Found (M+H) $^+$: 366.1197.

A8B11: 124.4 mg, 25%. ^{1}H-NMR (700 MHz, $(CD_3)_2SO$) δ 8.52 (d, *J* = 8.9 Hz, 2H), 8.35 – 8.19 (m, 3H), 7.45 (d, *J* = 3.8 Hz, 1H), 7.35 (d, *J* = 3.8 Hz, 1H), 7.07 (s, 1H), 6.91 (d, *J* = 15.4 Hz, 1H), 4.99 (t, *J* = 5.7 Hz, 1H), 3.80 (t, *J* = 5.4 Hz, 2H), 3.55 (q, *J* = 5.5 Hz, 2H) ppm. ^{13}C-NMR (176 MHz, $(CD_3)_2SO$) δ 170.1, 163.1, 147.0, 146.2, 142.2, 141.3, 138.6, 133.8, 133.0, 132.7, 123.8, 120.8, 113.4, 82.7, 59.2, 42.6 ppm. HRMS (m/z) Anal. Calc'd for $C_{18}H_{14}N_3O_4SI$ (M+H) $^+$: 495.9822, Found (M+H) $^+$: 498.9828.

A8B12: 96.5 mg, 23%. ^{1}H-NMR (700 MHz, $(CD_3)_2SO$) δ 8.59 (d, *J* = 7.7 Hz, 2H), 8.44 – 8.24 (m, 3H), 8.20 (d, *J* = 8.0 Hz, 1H), 8.11 (d, *J* = 8.1 Hz, 1H), 7.73 – 7.47 (m, 3H), 7.25 (s, 1H), 5.09 (t, *J* = 5.5 Hz, 1H), 3.89 (t, *J* = 5.3 Hz, 2H), 3.59 (q, *J* = 5.4 Hz, 2H) ppm. ^{13}C-NMR (176 MHz, $(CD_3)_2SO$) δ 169.8, 163.8, 162.2, 153.4, 147.3, 141.8, 140.8, 135.3, 133.1, 132.4, 127.2, 126.6, 123.8, 123.5, 123.2, 122.7, 122.0, 59.1, 42.7 ppm. HRMS (m/z) Anal. Calc'd for $C_{21}H_{16}N_4O_4S$ (M+H) $^+$: 421.0965, Found (M+H) $^+$: 421.0973.

A9B1: 92.9 mg, 29%. ^{1}H-NMR (700 MHz, $(CD_3)_2SO$) δ 8.76 – 8.58 (m, 2H), 8.24 – 8.19 (m, 2H), 8.15 (d, *J* = 15.7 Hz, 1H), 7.88 (d, *J* = 6.9 Hz, 2H), 7.53 – 7.43 (m, 3H), 7.32 (d, *J* = 15.8 Hz, 1H), 6.98 (s, 1H), 4.98 (s, 1H), 3.88 (t, *J* = 5.4 Hz, 2H), 3.58 (t, *J* = 5.5 Hz, 2H) ppm. ^{13}C-NMR (176 MHz, $(CD_3)_2SO$) δ 170.0, 163.3, 150.0, 143.0, 141.6, 141.5, 135.0, 130.6, 129.0, 128.7, 125.2, 120.8, 114.3, 59.2, 42.5 ppm. HRMS (m/z) Anal. Calc'd for $C_{19}H_{17}N_3O_2$ (M+H) $^+$: 320.1394, Found (M+H) $^+$: 320.1403.

A9B2: 40.6 mg, 11%. ^{1}H-NMR (700 MHz, $(CD_3)_2SO$) δ 8.68 (d, *J* = 5.5 Hz, 2H), 8.30 (d, *J* = 5.6 Hz, 2H), 8.12 (d, *J* = 15.3 Hz, 1H), 7.72 (d, *J* = 8.6 Hz, 2H), 6.97 (d, *J* = 15.4 Hz, 1H), 6.91 – 6.69 (m, 3H), 4.96 (s, 1H), 3.85 (t, *J* = 5.5 Hz, 2H), 3.57 (t, *J* = 5.5 Hz, 3H), 3.04 (s, 6H) ppm. ^{13}C-NMR (176 MHz, $(CD_3)_2SO$) δ 174.5, 170.1, 164.5, 152.2, 148.2, 144.3, 143.7, 130.9, 129.7, 129.7, 125.3, 122.5, 116.7, 111.9, 107.1, 59.2, 42.4 ppm. HRMS (m/z) Anal. Calc'd for $C_{21}H_{22}N_4O_2$ (M+H) $^+$: 363.1816, Found (M+H) $^+$: 363.1811.

A9B3: 34.4 mg, 9%. ^{1}H-NMR (700 MHz, $(CD_3)_2SO$) δ 8.83 – 6.07 (m, 13H), 4.96 (m, 1H), 3.74 (m, 2H), 3.54 (m, 2H), 3.00 (m, 6H) ppm. ^{13}C-NMR (176 MHz, $(CD_3)_2SO$) δ 170.0, 169.5, 163.3, 162.6, 151.4, 151.2, 150.1, 150.0, 145.5, 144.3, 144.2, 143.4, 143.4, 142.6, 141.9, 141.7, 129.3, 129.2, 125.0, 124.9, 123.8, 123.7, 122.9, 122.2, 119.4, 118.7, 113.4, 112.2, 112.1, 108.9, 59.1, 59.1, 42.5, 42.4 ppm. HRMS (m/z) Anal. Calc'd for $C_{23}H_{24}N_4O_2$ (M+H) $^+$: 389.1972, Found (M+H) $^+$: 389.1972.

A9B4: 18.7 mg, 5%. ^{1}H-NMR (700 MHz, $(CD_3)_2SO$) δ 8.65 (d, *J* = 5.1 Hz, 2H), 8.39 (d, *J* = 15.5 Hz, 1H), 8.19 (d, *J* = 5.3 Hz, 2H), 8.15 (s, 1H), 8.10 (d, *J* = 7.8 Hz, 1H), 7.59 (d, *J* = 8.2 Hz, 1H), 7.32 (m, 2H), 7.00 (d, *J* = 15.5 Hz, 1H), 6.80 (s, 1H), 5.06 (t, *J* = 5.6 Hz, 1H), 3.88 (m, 5H), 3.62 (q, *J* = 5.2 Hz, 2H) ppm. ^{13}C-NMR (176 MHz, $(CD_3)_2SO$) δ 170.2, 164.2, 150.0, 143.4, 141.9, 138.1, 136.8, 136.5, 125.4, 124.8, 123.0, 121.6, 120.5, 117.3, 112.6, 111.0, 106.7, 59.3, 42.6, 33.1 ppm. HRMS (m/z) Anal. Calc'd for $C_{22}H_{20}N_4O_2$ (M+H) $^+$: 373.1659, Found (M+H) $^+$: 373.1674.

A9B5: 74.1 mg, 22%. ^{1}H-NMR (700 MHz, $(CD_3)_2SO$) δ 8.65 (d, *J* = 5.9 Hz, 2H), 8.18 (d, *J* = 5.9 Hz, 2H), 8.08 (d, *J* = 15.6 Hz, 1H), 7.73 (d, *J* = 8.6 Hz, 2H), 7.06 (d, *J* = 15.6 Hz, 1H), 6.90 – 6.83 (m, 3H), 4.96 (s, 1H), 3.84 (t, *J* = 5.5 Hz, 2H), 3.56 (t, *J* = 5.6 Hz, 2H) ppm. ^{13}C-NMR (176 MHz, $(CD_3)_2SO$) δ 170.1, 163.6, 160.2, 150.1, 143.2, 142.2, 141.6, 130.9, 126.3, 125.1, 119.5, 116.0, 110.3, 59.2, 42.5 ppm. HRMS (m/z) Anal. Calc'd for $C_{19}H_{17}N_3O_3$ (M+H) $^+$: 336.1343, Found (M+H) $^+$: 336.1369.

A9B6: 104.9 mg, 30%. ^{1}H-NMR (700 MHz, $(CD_3)_2SO$) δ 8.66 (d, *J* = 6.1 Hz, 2H), 8.19 (d, *J* = 6.1 Hz, 2H), 8.13 (d, *J* = 15.6 Hz, 1H), 7.85 (d, *J* = 8.8 Hz, 2H), 7.15 (d, *J* = 15.6 Hz, 1H), 7.05 (d, *J* = 8.8 Hz, 2H), 6.91 (s, 1H), 4.98 (t, *J* = 5.9 Hz, 1H), 3.86 (m, 5H), 3.58 (q, 5.25, 2H) ppm. ^{13}C-NMR (176 MHz, $(CD_3)_2SO$) δ 170.1, 163.5,

161.4, 150.1, 143.1, 141.7, 141.6, 130.6, 127.8, 125.1, 119.8, 114.6, 111.4, 59.2, 55.5, 42.5 ppm. HRMS (m/z) Anal. Calc'd for $C_{20}H_{19}N_3O_3$ (M+H) $^+$: 350.1499, Found (M+H) $^+$: 350.1508.

A9B7: 160.0 mg, 47%. ^{1}H-NMR (700 MHz, $(CD_3)_2SO$) δ 8.68 (d, *J* = 6.0 Hz, 2H), 8.25 – 8.14 (m, 3H), 8.07 (d, *J* = 8.1 Hz, 2H), 7.95 (d, *J* = 8.3 Hz, 2H), 7.48 (d, *J* = 15.8 Hz, 1H), 7.03 (s, 1H), 4.97 (t, *J* = 5.7 Hz, 1H), 3.89 (m, 2H), 3.57 (q, *J* = 5.5 Hz, 2H) ppm. ^{13}C-NMR (176 MHz, $(CD_3)_2SO$) δ 169.9, 162.8, 150.2, 142.7, 141.1, 139.5, 139.2, 132.8, 129.2, 125.3, 122.0, 118.7, 117.9, 112.1, 59.2, 42.5 ppm. HRMS (m/z) Anal. Calc'd for $C_{20}H_{16}N_4O_2$ (M+H) $^+$: 345.1346, Found (M+H) $^+$: 345.1344.

A9B8: 39.3 mg, 11%. ^{1}H-NMR (700 MHz, $(CD_3)_2SO$) δ 8.68 (d, *J* = 6.1 Hz, 2H), 8.36 – 8.08 (m, 7H), 7.53 (d, *J* = 15.8 Hz, 1H), 7.05 (s, 1H), 5.00 (t, *J* = 5.6 Hz, 1H), 3.90 (t, *J* = 5.4 Hz, 2H), 3.58 (q, *J* = 5.4 Hz, 2H) ppm. ^{13}C-NMR (176 MHz, $(CD_3)_2SO$) δ 169.9, 162.8, 150.2, 148.0, 142.8, 141.5, 141.2, 138.7, 129.6, 125.4, 124.1, 122.2, 118.8, 59.2, 42.5 ppm. HRMS (m/z) Anal. Calc'd for $C_{19}H_{16}N_4O_4$ (M+H) $^+$: 365.1244, Found (M+H) $^+$: 365.1259.

A9B9: 34.6 mg, 11%. ^{1}H-NMR (700 MHz, $(CD_3)_2SO$) δ 8.71 – 8.64 (m, 4H), 8.23 – 8.16 (m, 2H), 8.09 (d, *J* = 15.8 Hz, 1H), 7.86 – 7.78 (m, 2H), 7.56 (d, *J* = 15.9 Hz, 1H), 7.06 (s, 1H), 4.98 (t, *J* = 5.7 Hz, 1H), 3.89 (t, *J* = 5.4 Hz, 2H), 3.57 (q, *J* = 5.5 Hz, 2H) ppm. ^{13}C-NMR (176 MHz, $(CD_3)_2SO$) δ 169.9, 162.7, 150.4, 150.2, 142.7, 142.0, 141.1, 138.5, 125.3, 122.3, 122.2, 119.0, 59.1, 42.5 ppm. HRMS (m/z) Anal. Calc'd for $C_{18}H_{16}N_4O_2$ (M+H) $^+$: 321.1346, Found (M+H) $^+$: 321.1371.

A9B10: not obtained.

A9B11: 126.0 mg, 28%. ^{1}H-NMR (700 MHz, $(CD_3)_2SO$) δ 8.74 – 8.59 (m, 2H), 8.24 (d, *J* = 15.4 Hz, 1H), 8.21 – 8.13 (m, 2H), 7.45 (d, *J* = 3.7 Hz, 1H), 7.39 (d, *J* = 3.8 Hz, 1H), 6.95 (s, 1H), 6.92 (d, *J* = 15.4 Hz, 1H), 4.97 (t, *J* = 5.7 Hz, 1H), 3.81 (t, *J* = 5.4 Hz, 2H), 3.55 (q, *J* = 5.5 Hz, 2H) ppm. ^{13}C-NMR (176 MHz, $(CD_3)_2SO$) δ 169.9, 162.8, 150.1, 146.1, 142.8, 141.3, 138.5, 133.8, 132.9, 125.1, 120.6, 113.4, 82.6, 59.1, 42.5 ppm. HRMS (m/z) Anal. Calc'd for $C_{17}H_{14}N_3O_2SI$ (M+H) $^+$: 451.9924, Found (M+H) $^+$: 451.9927.

A9B12: not obtained.

A10B1: 120 mg, 38%. ^{1}H-NMR (700 MHz, $(CD_3)_2SO$) δ 9.61 (s, 2H), 9.15 (s, 1H), 8.15 (d, *J* = 15.7 Hz, 1H), 7.88 (d, *J* = 6.5 Hz, 2H), 7.55 – 7.40 (m, 3H), 7.31 (d, *J* = 15.7 Hz, 1H), 7.05 (s, 1H), 4.97 (t, *J* = 5.7 Hz, 1H), 3.88 (t, *J* = 5.5 Hz, 2H), 3.58 (q, *J* = 5.5 Hz, 2H) ppm. ^{13}C-NMR (176 MHz, $(CD_3)_2SO$) δ 169.5, 163.0, 158.7, 157.5, 142.2, 141.6, 135.1, 130.5, 129.3, 129.0, 128.6, 117.1, 114.2, 59.2, 42.5 ppm. HRMS (m/z) Anal. Calc'd for $C_{18}H_{16}N_4O_2$ (M+H) $^+$: 321.1346, Found (M+H) $^+$: 321.1351.

A10B2: 17.3 mg, 5%. ^{1}H-NMR (700 MHz, $(CD_3)_2SO$) δ 9.59 (s, 2H), 9.12 (s, 1H), 8.08 (d, *J* = 15.4 Hz, 1H), 7.72 (d, *J* = 8.9 Hz, 2H), 6.95 (d, *J* = 15.4 Hz, 1H), 6.88 (s, 1H), 6.76 (d, *J* = 8.9 Hz, 2H), 4.94 (s, 1H), 3.84 (t, *J* = 5.6 Hz, 2H), 3.57 (t, *J* = 5.5 Hz, 2H), 3.03 (s, 6H) ppm. ^{13}C-NMR (176 MHz, $(CD_3)_2SO$) δ 169.7, 163.5, 158.3, 157.0, 152.0, 142.9, 142.7, 130.7, 129.7, 122.6, 114.1, 111.8, 107.2, 59.2, 42.4 ppm. HRMS (m/z) Anal. Calc'd for $C_{20}H_{21}N_5O_2$ (M+H) $^+$: 364.1768, Found (M+H) $^+$: 364.1774.

A10B3: 20.4 mg, 5%. ^{1}H-NMR (700 MHz, $(CD_3)_2SO$) δ 9.58 (s, 2H), 9.13 (s, 1H), 7.94 (m, 1H), 7.46 (d, *J* = 8.5 Hz, 2H), 7.27 – 7.00 (m, 2H), 6.91 (s, 1H), 6.74 (d, *J* = 8.5 Hz, 2H), 6.62 (d, *J* = 14.7 Hz, 1H), 4.96 (t, *J* = 5.6 Hz, 1H), 3.75 (t, *J* = 5.6 Hz, 2H), 3.55 (q, *J* = 5.6 Hz, 2H), 2.98 (s, 6H) ppm. ^{13}C-NMR (176 MHz, $(CD_3)_2SO$) δ 169.5, 163.0, 158.4, 157.1, 151.1, 144.1, 142.6, 129.6, 129.1, 123.7, 122.8, 114.8, 113.2, 112.1, 59.0, 42.4 ppm. HRMS (m/z) Anal. Calc'd for $C_{22}H_{23}N_5O_2$ (M+H) $^+$: 390.1925, Found (M+H) $^+$: 390.1931.

A10B4: 30.2 mg, 8%. ^{1}H-NMR (700 MHz, $(CD_3)_2SO$) δ 9.61 (s, 2H), 9.12 (s, 1H), 8.38 (d, *J* = 15.5 Hz, 1H), 8.17 (s, 1H), 8.09 (d, *J* = 7.8 Hz, 1H), 7.58 (d, *J* = 8.1 Hz, 1H), 7.32 (m, 2H), 6.99 (d, *J* = 15.5 Hz, 1H), 6.87 (s, 1H), 5.07 (m, 1H), 3.87 (m, 5H), 3.62 (q, *J* = 5.2 Hz, 2H) ppm. ^{13}C-NMR (176 MHz, $(CD_3)_2SO$) δ 169.7, 164.0, 158.3, 157.0, 142.7, 138.2, 137.0, 136.5, 129.8, 125.3, 123.0, 121.6, 120.6, 113.5, 112.7, 111.0, 106.6, 59.3, 42.6, 33.1 ppm. HRMS (m/z) Anal. Calc'd for $C_{21}H_{19}N_5O_2$ (M+H) $^+$: 374.1612, Found (M+H) $^+$: 374.1615.

A10B5: 25.2 mg, 7%. ^{1}H-NMR (700 MHz, $(CD_3)_2SO$) δ 10.13 (s, 1H), 9.59 (s, 2H), 9.13 (s, 1H), 8.08 (d, *J* = 15.6 Hz, 1H), 7.74 (d, *J* = 8.3 Hz, 2H), 7.06 (d, *J* = 15.6 Hz, 1H), 6.96 (s, 1H), 6.85 (d, *J* = 8.3 Hz, 2H), 4.94 (s, 1H), 3.85 (t, *J* = 5.5 Hz, 2H), 3.57 (t, *J* = 5.5 Hz, 2H) ppm. ^{13}C-NMR (176 MHz, $(CD_3)_2SO$) δ 169.6, 163.3, 160.2, 158.5, 157.3, 142.4, 142.1, 130.8, 129.5, 126.3, 115.9, 115.6, 110.1, 59.2, 42.4 ppm. HRMS (m/z) Anal. Calc'd for $C_{18}H_{16}N_4O_3$ (M+H) $^+$: 337.1295, Found (M+H) $^+$: 337.1299.

A10B6: not obtained.

A10B7: 76.9 mg, 22%. ^{1}H-NMR (700 MHz, $(CD_3)_2SO$) δ 9.62 (s, 2H), 9.17 (s, 1H), 8.20 (d, *J* = 15.8 Hz, 1H), 8.09 (d, *J* = 8.0 Hz, 2H), 7.94 (d, *J* = 7.9 Hz, 2H), 7.48 (d, *J* = 15.8 Hz, 1H), 7.11 (s, 1H), 4.96 (s, 1H), 3.89 (t, *J* = 5.4 Hz, 2H), 3.57 (s, 2H) ppm. ^{13}C-NMR (176 MHz, $(CD_3)_2SO$) δ 169.4, 162.5, 158.9, 157.7, 142.0, 139.6, 139.2, 132.8, 129.2, 129.1, 118.7, 118.2, 117.8, 112.0, 59.1, 42.5 ppm. HRMS (m/z) Anal. Calc'd for $C_{19}H_{15}N_5O_2$ (M+H) $^+$: 346.1299, Found (M+H) $^+$: 346.1301.

A10B8: 46.7 mg, 13%. ^{1}H-NMR (700 MHz, $(CD_3)_2SO$) δ 9.62 (s, 2H), 9.17 (s, 1H), 8.36 – 8.21 (m, 3H), 8.16 (d, *J* = 8.7 Hz, 2H), 7.52 (d, *J* = 15.8 Hz, 1H), 7.12 (s, 1H), 4.98 (s, 1H), 3.90 (t, *J* = 5.4 Hz, 2H), 3.58 (m, 2H) ppm. ^{13}C-NMR (176 MHz, $(CD_3)_2SO$) δ 169.4, 162.5, 158.9, 157.7, 147.9, 142.0, 141.5, 138.7, 130.7, 129.6, 129.1, 124.3, 124.0, 118.6, 118.5, 59.1, 42.5 ppm. HRMS (m/z) Anal. Calc'd for $C_{18}H_{15}N_5O_4$ (M+H) $^+$: 366.1197, Found (M+H) $^+$: 366.1198.

A10B9: not obtained.

A10B10: 28.3 mg, 9%. ^{1}H-NMR (700 MHz, $(CD_3)_2SO$) δ 9.62 (s, 2H), 9.32 (s, 2H), 9.18 (d, *J* = 16.2 Hz, 2H), 8.16 (d, *J* = 15.9 Hz, 1H), 7.58 (d, *J* = 16.0 Hz, 1H), 7.13 (s, 1H), 4.95 (s, 1H), 3.90 (t, *J* = 5.5 Hz, 2H), 3.64 – 3.54 (m, 2H) ppm. ^{13}C-NMR (176 MHz, $(CD_3)_2SO$) δ 169.4, 162.3, 158.9, 158.6, 157.7, 156.3, 142.0, 134.6, 129.1, 129.1, 118.4, 117.8, 59.1, 42.5 ppm. HRMS (m/z) Anal. Calc'd for $C_{16}H_{14}N_6O_2$ (M+H) $^+$: 323.1251, Found (M+H) $^+$: 323.1253.

A10B11: 64.3 mg, 14%. ^{1}H-NMR (700 MHz, $(CD_3)_2SO$) δ 9.60 (m, 2H), 9.17 (m, 1H), 8.41 – 7.26 (m, 3H), 7.10 (m, 1H), 6.94 – 6.33 (m, 1H), 4.97 (m, 1H), 3.78 (m, 2H), 3.55 (m, 2H) ppm. ^{13}C-NMR (176 MHz, $(CD_3)_2SO$) δ 169.4, 162.6, 158.7, 157.5, 146.2, 142.2, 138.5, 133.9, 133.0, 129.3, 116.8, 113.2, 82.5, 59.1, 42.5 ppm. HRMS (m/z) Anal. Calc'd for $C_{16}H_{13}N_4O_2SI$ (M+H) $^+$: 452.9877, Found (M+H) $^+$: 452.9875.

A10B12: not obtained.

A11B1: 302.6 mg, 67%. ^{1}H-NMR (700 MHz, $(CD_3)_2SO$) δ 7.96 (d, *J* = 15.7 Hz, 1H), 7.88 – 7.81 (m, 2H), 7.52 – 7.32 (m, 6H), 7.25 (d, *J* = 15.7 Hz, 1H), 4.97 (t, *J* = 5.7 Hz, 1H), 3.83 (t, *J* = 5.5 Hz, 2H), 3.55 (q, *J* = 5.6 Hz, 2H) ppm. ^{13}C-NMR (176 MHz, $(CD_3)_2SO$) δ 169.1, 159.8, 144.1, 140.0, 137.7, 137.5, 135.9, 135.2, 130.3, 129.1, 128.5, 117.9, 114.6, 88.4, 59.3, 42.4 ppm. HRMS (m/z) Anal. Calc'd for $C_{18}H_{15}N_2O_2SI$ (M+H) $^+$: 450.9972, Found (M+H) $^+$: 450.9971.

A11B2: not obtained.

A11B3: not obtained.

A11B4: not obtained.

A11B5: 210.9 mg, 45%. ^{1}H-NMR (700 MHz, $(CD_3)_2SO$) δ 10.27 (s, 1H), 7.90 (d, *J* = 15.6 Hz, 1H), 7.69 (d, *J* = 8.4 Hz, 2H), 7.40 (d, *J* = 3.8 Hz, 1H), 7.33 (d, *J* = 3.8 Hz, 1H), 7.27 (s, 1H), 7.01 (d, *J* = 15.6 Hz, 1H), 6.85 (d, *J* = 8.2 Hz, 2H), 4.95 (s, 1H), 3.80 (t, *J* = 5.6 Hz, 2H), 3.54 (m, 3H) ppm. ^{13}C-NMR (176 MHz, $(CD_3)_2SO$) δ 169.2, 160.2, 159.9, 144.3, 140.5, 137.9, 137.4, 135.3, 130.5, 126.4, 116.7, 116.0, 110.7, 87.7, 59.3, 42.4 ppm. HRMS (m/z) Anal. Calc'd for $C_{18}H_{15}N_2O_3SI$ (M+H) $^+$: 466.9921, Found (M+H) $^+$: 466.9929.

A11B6: not obtained.

A11B7: 361.8 mg, 76%. ^{1}H-NMR (700 MHz, $(CD_3)_2SO$) δ 8.04 (d, *J* = 8.4 Hz, 2H), 7.97 (d, *J* = 15.8 Hz, 1H), 7.91 (d, *J* = 8.4 Hz, 2H), 7.48 – 7.34 (m, 4H), 4.96 (t, *J* = 5.7 Hz, 1H), 3.84 (t, *J* = 5.4 Hz, 2H), 3.55 (q, *J* = 5.5

Hz, 2H) ppm. ^{13}C-NMR (176 MHz, $(CD_3)_2SO$) δ 169.0, 159.3, 144.0, 139.7, 137.6, 137.6, 136.4, 132.8, 129.0, 118.9, 118.8, 118.2, 111.8, 89.1, 59.3, 42.4 ppm. HRMS (m/z) Anal. Calc'd for $C_{19}H_{14}N_3O_2SI$ (M+H) $^+$: 475.9924, Found (M+H) $^+$: 475.9923.

A11B8: 300.1 mg, 61%. ^{1}H-NMR (700 MHz, $(CD_3)_2SO$) δ 8.28 (d, *J* = 8.5 Hz, 2H), 8.11 (d, *J* = 8.5 Hz, 2H), 8.02 (d, *J* = 15.8 Hz, 1H), 7.55 – 7.31 (m, 4H), 4.98 (t, *J* = 5.7 Hz, 1H), 3.85 (t, *J* = 5.4 Hz, 2H), 3.56 (q, *J* = 5.5 Hz, 2H) ppm. ^{13}C-NMR (176 MHz, $(CD_3)_2SO$) δ 169.0, 159.2, 147.7, 144.0, 141.6, 137.6, 137.5, 137.0, 136.5, 129.4, 124.1, 119.1, 119.0, 89.3, 59.3, 42.4 ppm. HRMS (m/z) Anal. Calc'd for $C_{18}H_{14}N_3O_4SI$ (M+H) $^+$: 495.9822, Found (M+H) $^+$: 495.9829.

A11B9: 216.7 mg, 48%. ^{1}H-NMR (700 MHz, $(CD_3)_2SO$) δ 8.80 – 8.62 (m, 2H), 7.99 – 7.76 (m, 3H), 7.57 – 6.36 (m, 4H), 4.95 (t, *J* = 5.7 Hz, 1H), 3.85 (t, *J* = 5.4 Hz, 2H), 3.55 (m, 2H) ppm. ^{13}C-NMR (176 MHz, $(CD_3)_2SO$) δ 168.9, 159.1, 150.3, 143.9, 142.2, 137.5, 137.4, 136.8, 136.4, 122.1, 119.3, 119.1, 89.2, 59.2, 42.4 ppm. HRMS (m/z) Anal. Calc'd for $C_{17}H_{14}N_3O_2SI$ (M+H) $^+$: 451.9924, Found (M+H) $^+$: 451.9934.

A11B10: 30.2 mg, 7%. ^{1}H-NMR (700 MHz, $(CD_3)_2SO$) δ 9.30 (m, 2H), 9.18 (m, 1H), 7.93 (m, 1H), 7.70 – 7.32 (m, 4H), 4.92 (t, *J* = 5.7 Hz, 1H), 3.86 (t, *J* = 5.5 Hz, 2H), 3.56 (q, *J* = 5.5 Hz, 2H) ppm. ^{13}C-NMR (176 MHz, $(CD_3)_2SO$) δ 168.9, 159.0, 158.4, 156.1, 143.8, 137.5, 137.4, 136.3, 132.7, 129.3, 118.9, 118.3, 89.1, 59.2, 42.3 ppm. HRMS (m/z) Anal. Calc'd for $C_{16}H_{13}N_4O_2SI$ (M+H) $^+$: 452.9877, Found (M+H) $^+$: 452.9876.

A11B11: 91.1 mg, 18%. ^{1}H-NMR (700 MHz, $(CD_3)_2SO$) δ 8.03 (d, *J* = 15.5 Hz, 1H), 7.48 – 7.22 (m, 5H), 6.86 (d, *J* = 15.5 Hz, 1H), 4.97 (t, *J* = 5.7 Hz, 1H), 3.76 (t, *J* = 5.4 Hz, 2H), 3.52 (q, *J* = 5.5 Hz, 2H) ppm. ^{13}C-NMR (176 MHz, $(CD_3)_2SO$) δ 169.0, 159.3, 146.4, 144.1, 138.5, 137.7, 137.5, 135.9, 133.5, 131.3, 117.7, 113.9, 88.4, 81.6, 59.3, 42.5 ppm. HRMS (m/z) Anal. Calc'd for $C_{21}H_{18}N_3O_2SI$ (M+H) $^+$: 504.0237, Found (M+H) $^+$: 504.0245.

A11B12: not obtained.

A12B1: 37.6 mg, 10%. ^{1}H-NMR (700 MHz, $(CD_3)_2SO$) δ 8.26 – 8.15 (m, 2H), 8.08 (d, *J* = 8.1 Hz, 1H), 7.90 (d, *J* = 6.2 Hz, 2H), 7.59 – 7.46 (m, 5H), 7.37 (d, *J* = 15.7 Hz, 1H), 7.24 (s, 1H), 5.01 (t, *J* = 5.8 Hz, 1H), 3.90 (t, *J* = 5.4 Hz, 2H), 3.60 (q, *J* = 5.5 Hz, 2H) ppm. ^{13}C-NMR (176 MHz, $(CD_3)_2SO$) δ 169.4, 164.0, 162.2, 152.7, 144.2, 142.7, 137.7, 134.9, 131.0, 129.2, 128.9, 126.8, 126.4, 123.5, 122.3, 116.1, 114.3, 59.2, 42.7 ppm. HRMS (m/z) Anal. Calc'd for $C_{21}H_{17}N_3O_2S$ (M+H) $^+$: 376.1114, Found (M+H) $^+$: 376.1102.

A12B2: 18.6 mg, 4%. ^{1}H-NMR (700 MHz, $(CD_3)_2SO$) δ 8.21 – 8.13 (m, 2H), 8.04 (d, *J* = 8.1 Hz, 1H), 7.74 (d, *J* = 8.6 Hz, 2H), 7.51 (m, 2H), 7.08 (s, 1H), 6.99 (d, *J* = 15.3 Hz, 1H), 6.80 (d, *J* = 8.5 Hz, 2H), 4.98 (s, 1H), 3.86 (t, *J* = 5.5 Hz, 2H), 3.59 (m, 2H), 3.05 (s, 6H) ppm. ^{13}C-NMR (176 MHz, $(CD_3)_2SO$) δ 169.5, 164.5, 162.6, 152.7, 152.4, 144.9, 144.4, 137.5, 131.2, 126.6, 126.0, 123.1, 122.4, 122.3, 113.0, 112.0, 106.9, 59.2, 42.6 ppm. HRMS (m/z) Anal. Calc'd for $C_{23}H_{22}N_4O_2S$ (M+H) $^+$: 419.1536, Found (M+H) $^+$: 419.1534.

A12B3: 37.1 mg, 8%. ^{1}H-NMR (700 MHz, $(CD_3)_2SO$) δ 8.79 – 6.61 (m, 13H), 5.00 (m, 1H), 3.77 (m, 2H), 3.57 (m, 2H), 3.00 (m, 6H) ppm. ^{13}C-NMR (176 MHz, $(CD_3)_2SO$) δ 169.4, 164.0, 162.5, 152.7, 151.4, 145.5, 144.8, 143.6, 137.5, 129.7, 129.5, 126.7, 126.2, 123.6, 123.2, 122.9, 122.2, 113.8, 113.1, 112.2, 59.0, 42.7 ppm. HRMS (m/z) Anal. Calc'd for $C_{25}H_{24}N_4O_2S$ (M+H) $^+$: 445.1693, Found (M+H) $^+$: 445.1693.

A12B4: 28.9 mg, 7%. ^{1}H-NMR (700 MHz, $(CD_3)_2SO$) δ 8.49 (d, *J* = 15.4 Hz, 1H), 8.24 (s, 1H), 8.17 (d, *J* = 7.9 Hz, 1H), 8.11 (d, *J* = 7.8 Hz, 1H), 8.05 (d, *J* = 8.0 Hz, 1H), 7.61 (d, *J* = 8.0 Hz, 1H), 7.58 – 7.52 (m, 1H), 7.48 (t, *J* = 7.5 Hz, 1H), 7.35 (m, 2H), 7.08 (s, 1H), 7.03 (d, *J* = 15.3 Hz, 1H), 5.08 (t, *J* = 5.7 Hz, 1H), 3.90 (m, 5H), 3.70 – 3.60 (m, 2H) ppm. ^{13}C-NMR (176 MHz, $(CD_3)_2SO$) δ 169.5, 165.0, 162.6, 152.7, 144.9, 138.3, 137.8, 137.6, 137.4, 126.6, 125.9, 125.4, 123.2, 123.1, 122.1, 121.9, 120.6, 112.9, 112.4, 111.2, 106.3, 59.3, 42.8, 33.2 ppm. HRMS (m/z) Anal. Calc'd for $C_{24}H_{20}N_4O_2S$ (M+H) $^+$: 445.1693, Found (M+H) $^+$: 445.1698.

A12B5: not obtained.

A12B6: 57.5 mg, 14%. ^{1}H-NMR (700 MHz, $(CD_3)_2SO$) δ 8.19 (s, 2H), 8.06 (d, *J* = 8.1 Hz, 1H), 7.87 (d, *J* = 8.4 Hz, 2H), 7.52 (m, 2H), 7.19 (d, *J* = 16.1 Hz, 2H), 7.07 (d, *J* = 8.4 Hz, 2H), 5.00 (t, *J* = 5.8 Hz, 1H), 3.86 m, 5H), 3.59 (q, *J* = 5.6 Hz, 2H) ppm. ^{13}C-NMR (176 MHz, $(CD_3)_2SO$) δ 169.5, 164.3, 162.3, 161.7, 152.7, 144.4, 142.8, 137.6, 130.9, 127.7, 126.7, 126.3, 123.4, 122.3, 115.1, 114.7, 111.3, 59.2, 55.6, 42.7 ppm. HRMS (m/z) Anal. Calc'd for $C_{22}H_{19}N_3O_3S$ (M+H) $^+$: 406.1220, Found (M+H) $^+$: 406.1223.

A12B7: 35.4 mg, 9%. ^{1}H-NMR (700 MHz, $(CD_3)_2SO$) δ 8.23 – 8.12 (m, 2H), 8.08 (m, 3H), 7.96 (d, *J* = 8.1 Hz, 2H), 7.60 – 7.47 (m, 3H), 7.28 (s, 1H), 5.01 (t, *J* = 5.8 Hz, 1H), 3.90 (t, *J* = 5.4 Hz, 2H), 3.59 (q, *J* = 5.5 Hz, 2H) ppm. ^{13}C-NMR (176 MHz, $(CD_3)_2SO$) δ 169.3, 163.5, 162.0, 152.7, 144.0, 140.1, 139.3, 137.8, 132.9, 129.4, 126.9, 126.6, 123.6, 122.3, 118.7, 117.9, 117.2, 112.4, 59.2, 42.7 ppm. HRMS (m/z) Anal. Calc'd for $C_{22}H_{16}N_4O_2S$ (M+H) $^+$: 401.1067, Found (M+H) $^+$: 401.1074.

A12B8: not obtained.

A12B9: not obtained.

A12B10: not obtained.

A12B11: 17.6 mg, 3%. ^{1}H-NMR (700 MHz, $(CD_3)_2SO$) δ 8.27 (d, *J* = 15.3 Hz, 1H), 8.16 (d, *J* = 8.1 Hz, 1H), 8.08 (d, *J* = 8.8 Hz, 1H), 7.59 – 7.54 (m, 1H), 7.52 – 7.43 (m, 3H), 7.21 (s, 1H), 6.98 (d, *J* = 15.3 Hz, 1H), 4.99 (t, *J* = 5.7 Hz, 1H), 3.84 (t, *J* = 5.4 Hz, 2H), 3.57 (q, *J* = 5.5 Hz, 2H) ppm. ^{13}C-NMR (176 MHz, $(CD_3)_2SO$) δ 169.2, 163.6, 162.1, 152.7, 146.0, 144.1, 138.7, 137.6, 134.3, 133.7, 126.7, 126.3, 123.4, 122.2, 115.7, 113.3, 83.5, 59.1, 42.7 ppm. HRMS (m/z) Anal. Calc'd for $C_{19}H_{14}N_3O_2S_2I$ (M+H) $^+$: 507.9645, Found (M+H) $^+$: 507.9648.

A12B12: not obtained.

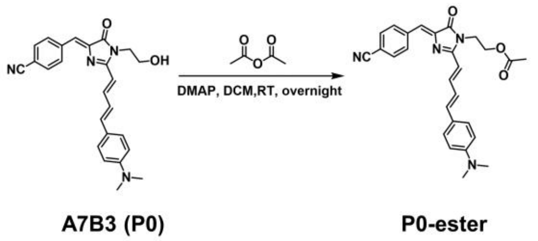

***Synthesis of P0-ester*:** Acetic anhydride (26 µL, 1.1 eq.) and a catalytic amount of DMAP were added to a solution of P0 (103 mg, 0.25 mM, 1 eq.) in DCM. The reaction mixture was left to stand overnight at room temperature. Then the mixture was poured into ice-cold water, and the crude product was extracted with DCM. The organic layer was washed with brine and dried over Na_2SO_4. The solvent was removed under reduced pressure, and the crude residue purified by column chromatography to yield a dark red product (104.4 mg, 92%). ^{1}H-NMR (400 MHz, $(CD_3)_2SO$) δ 8.44 (d, *J* = 8.2 Hz, 2H), 7.96 (m, 3H), 7.49 (d, *J* = 8.8 Hz, 2H), 7.19 (m, 2H), 6.97 (s, 1H), 6.76 (d, *J* = 8.8 Hz, 2H), 6.63(d, *J* = 14.6 Hz, 1H), 4.22 (m, 2H), 3.97 (m, 2H), 2.99 (s, 6H), 1.93 (s, 3H) ppm. ^{13}C NMR (176 MHz, DMSO) δ 170.1, 169.9, 162.1, 151.2, 144.2, 142.7, 141.8, 139.4, 132.4, 132.0, 129.2, 123.6, 122.7, 120.1, 119.0, 112.8, 112.1, 110.8, 61.6, 54.9, 20.6 ppm. HRMS (m/z) Anal. Calc'd for $C_{27}H_{26}N_4O_3$ (M+H) $^+$: 455.2078, Found (M+H) $^+$: 455.2093.

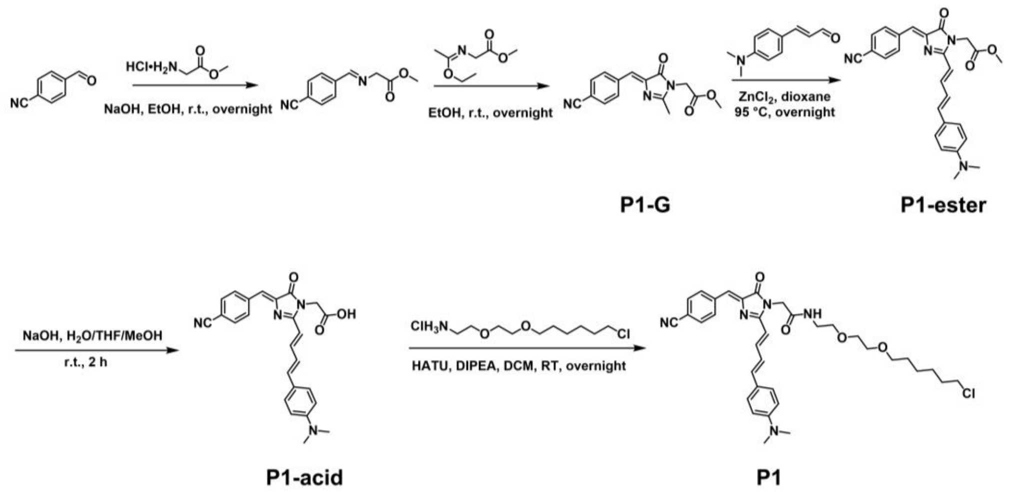

***Synthesis of P1*:** The same synthetic methods previously described were employed to obtain P1-ester. Subsequently, P1-ester (88 mg, 0.2 mM, 1 e.q.) was dissolved in 5 mL of a methanol/tetrahydrofuran (1:1) solution, followed by the addition of 2.5 mL of 4 M sodium hydroxide. The mixture was stirred for 2 h at room temperature. Once the hydrolysis process was complete, the reaction was quenched by the addition of 1 M HCl. Subsequently, the pH was adjusted to a range of 4 to 5, and extracted with EA. The organic layer was then washed with acidified brine and dried over Na_2SO_4. The solvent was removed under vacuum, yielding P1-acid. The crude product was utilized for the subsequent step without any further purification. Ultimately, the P1-acid (43 mg, 0.1 mM, 1 e.q.), Halo linker (39 mg, 0.15 mM, 1.5 e.q.), HATU (42 mg, 0.11 mM, 1.1 e.q.), and DIPEA (52 µL, 0.3 mM, 3 e.q.) were combined in 5 mL of DCM and stirred for 12 h at room temperature. Subsequently, the reaction mixture was diluted with DCM, washed with a saturated solution of $NaHCO_3$, and then with brine, dried over Na_2SO_4. The solvent was then removed by rotary evaporation. Subsequently, the crude product was purified by column chromatography, yielding 23 mg (36%) of the purified product. ^{1}H-NMR (700 MHz, $(CD_3)_2SO$) δ 8.46 (d, J = 8.1 Hz, 2H), 8.36 (t, J = 5.7 Hz, 1H), 7.90 (m, 3H), 7.47 (d, J = 8.5 Hz, 2H), 7.15 (m, 1H), 7.02 (m, 2H), 6.74 (d, J = 8.5 Hz, 2H), 6.39 (d, J = 14.7 Hz, 1H), 4.37 (s, 2H), 3.60 (m, 2H), 3.52 (m, 2H), 3.47 (m, 5H), 3.26 (m, 2H), 2.98 (s, 6H), 1.69 (m, 2H), 1.48 (m, 2H), 1.37 (m, 2H), 1.27 (m, 2H) ppm. ^{13}C-NMR (176 MHz, $(CD_3)_2SO$) δ 169.8, 166.5, 162.4, 151.2, 143.9, 142.5, 142.0, 139.5, 132.4, 132.0, 129.1, 123.7, 122.8, 120.0, 113.1, 112.1, 110.8, 70.2, 69.6, 69.4, 68.9, 45.4, 32.0, 29.1, 26.1, 24.9 ppm. HRMS (m/z) Anal. Calc'd for $C_{35}H_{42}N_5O_4Cl$ $(M+H)^+$: 632.2998, Found $(M+H)^+$: 632.3003.

5. NMR Spectra

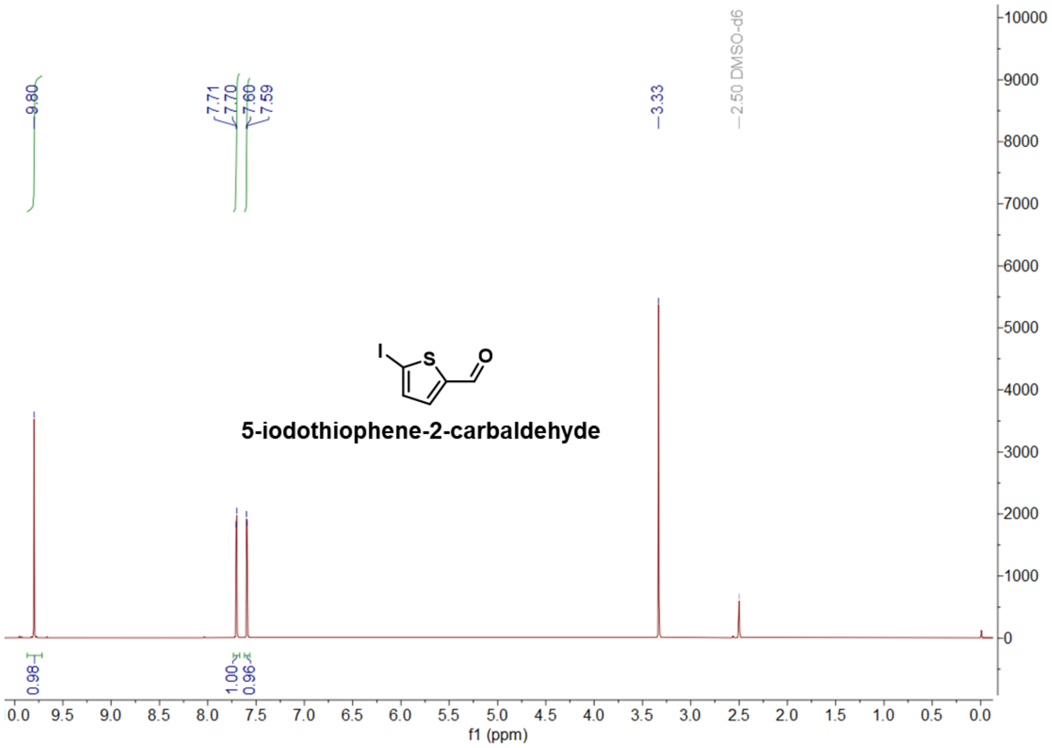

^{1}H NMR spectrum of 5-iodothiophene-2-carbaldehyde ($(CD_3)_2SO$)

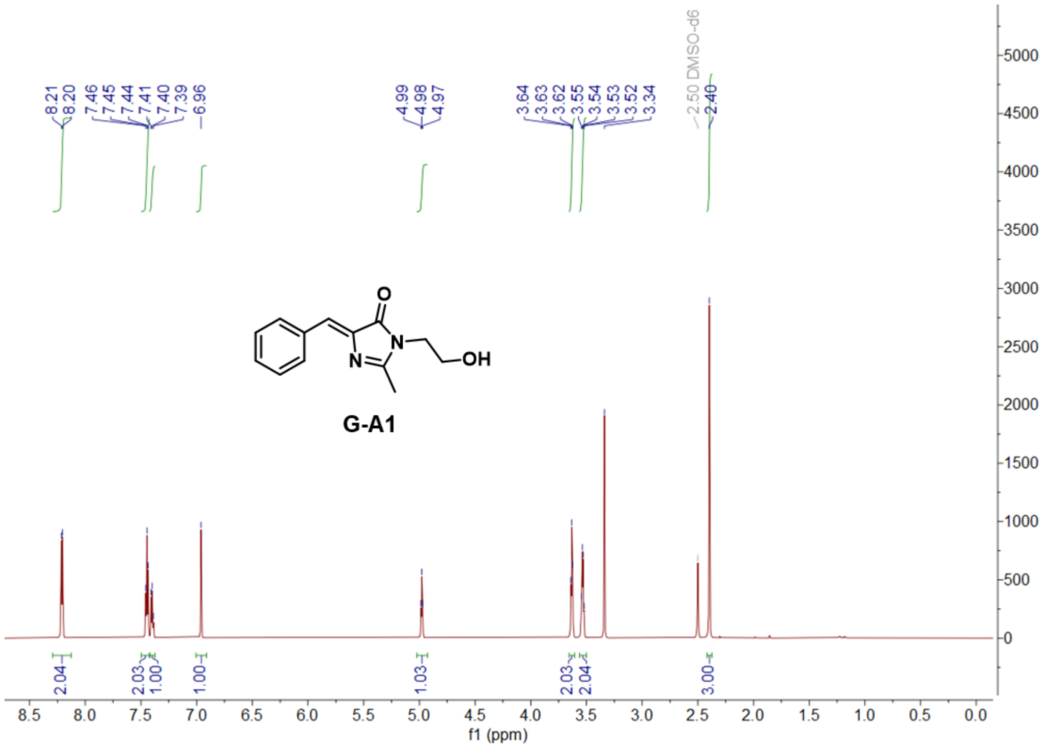

^{1}H NMR spectrum of G-A1 ($(CD_3)_2SO$)

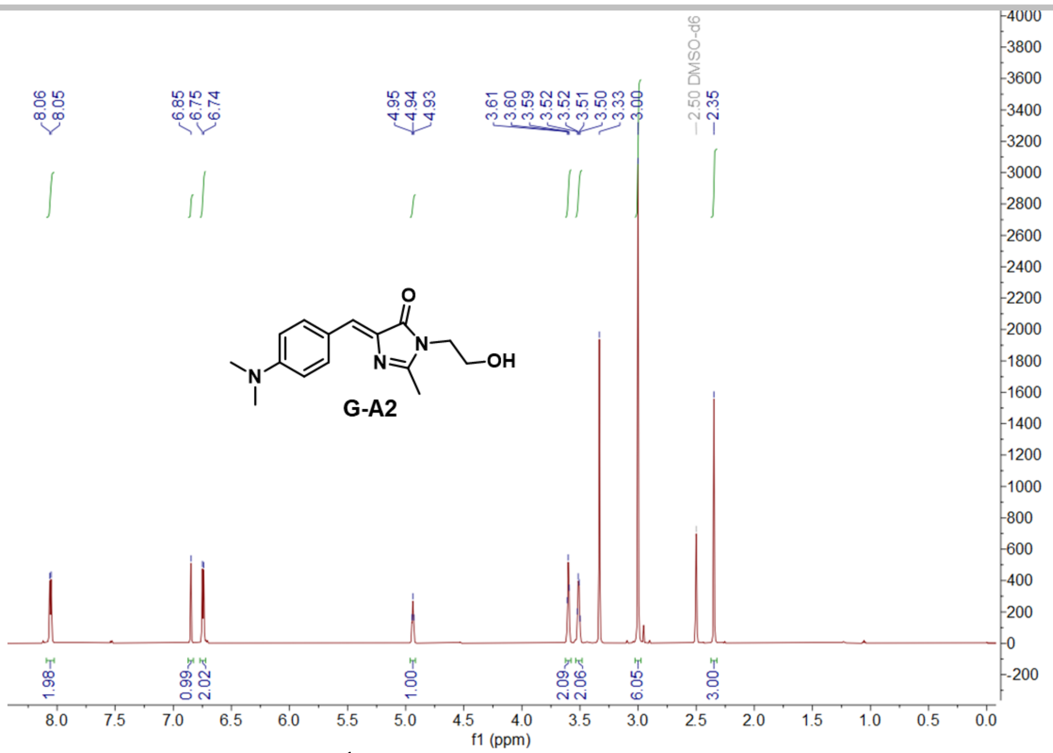

^{1}H NMR spectrum of G-A2 ($(CD_3)_2SO$)

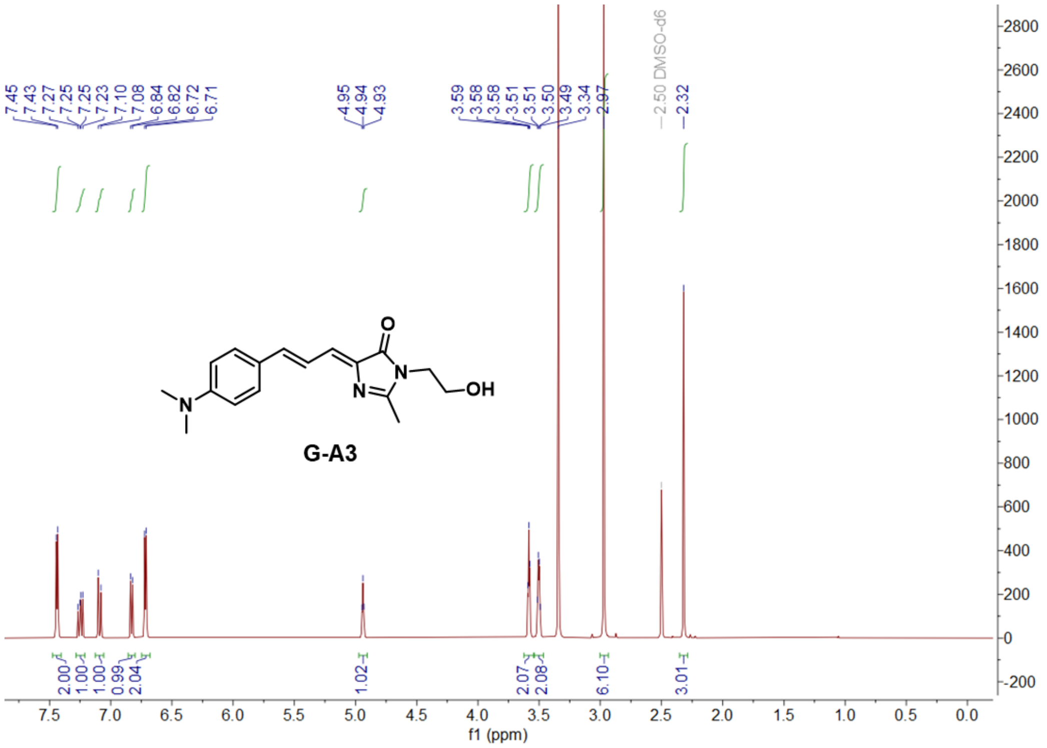

^{1}H NMR spectrum of G-A3 ($(CD_3)_2SO$)

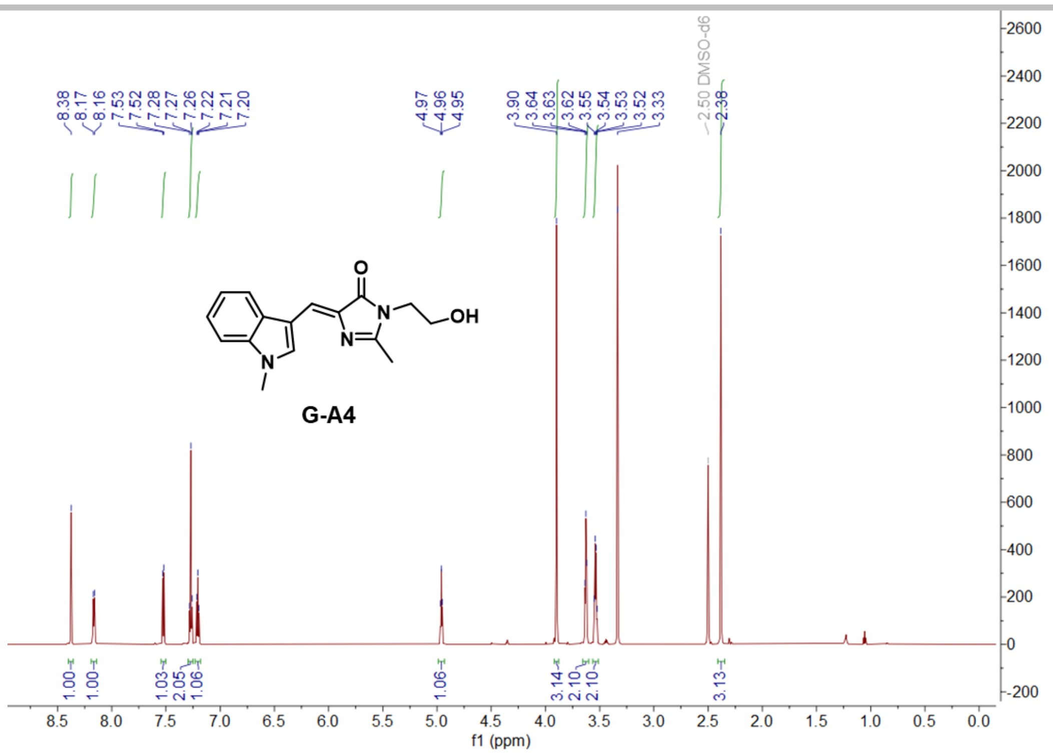

^1H NMR spectrum of G-A4 ($(CD_3)_2SO$)

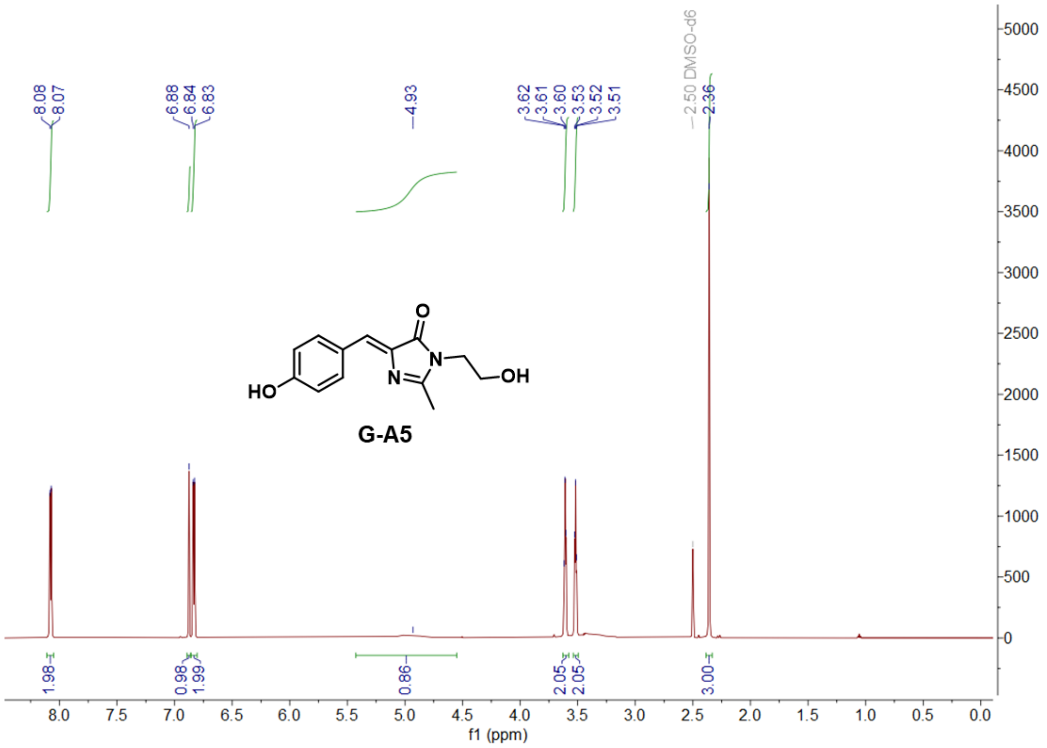

^1H NMR spectrum of G-A5 ($(CD_3)_2SO$)

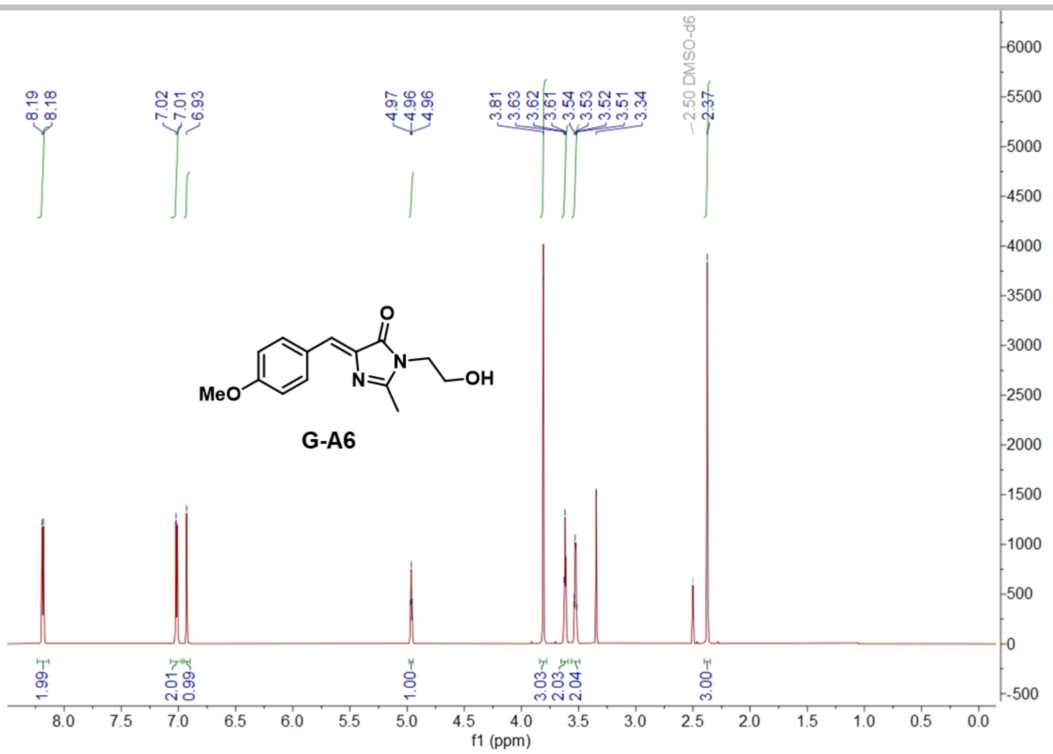

^{1}H NMR spectrum of G-A6 ($(CD_3)_2SO$)

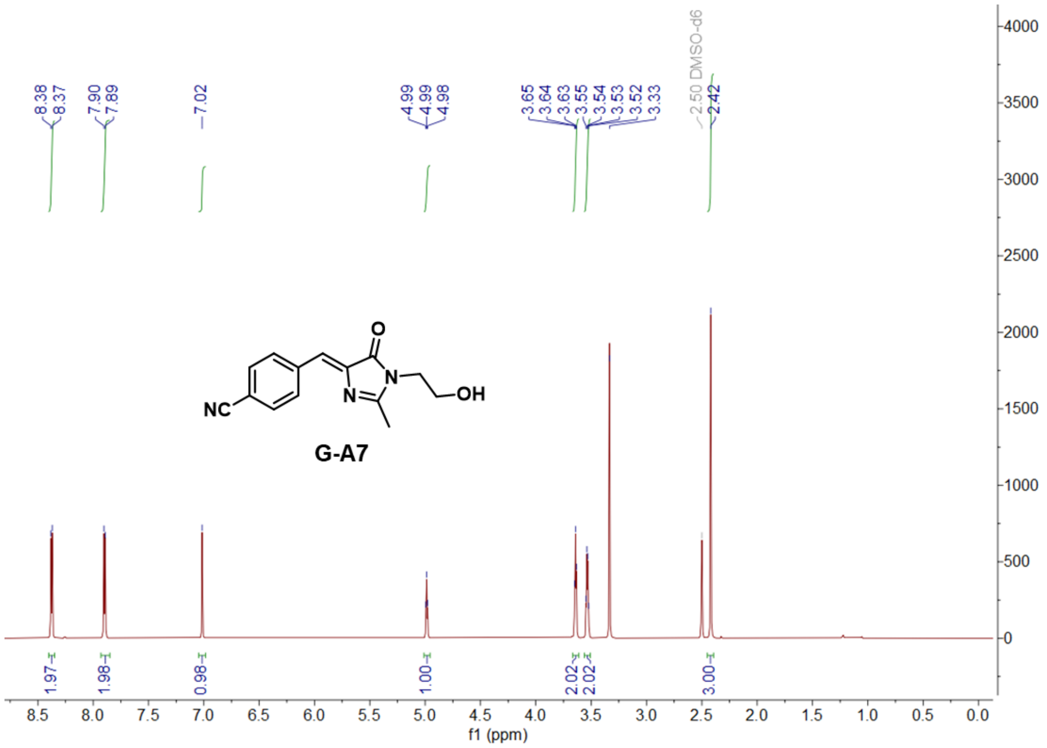

^{1}H NMR spectrum of G-A7 ($(CD_3)_2SO$)

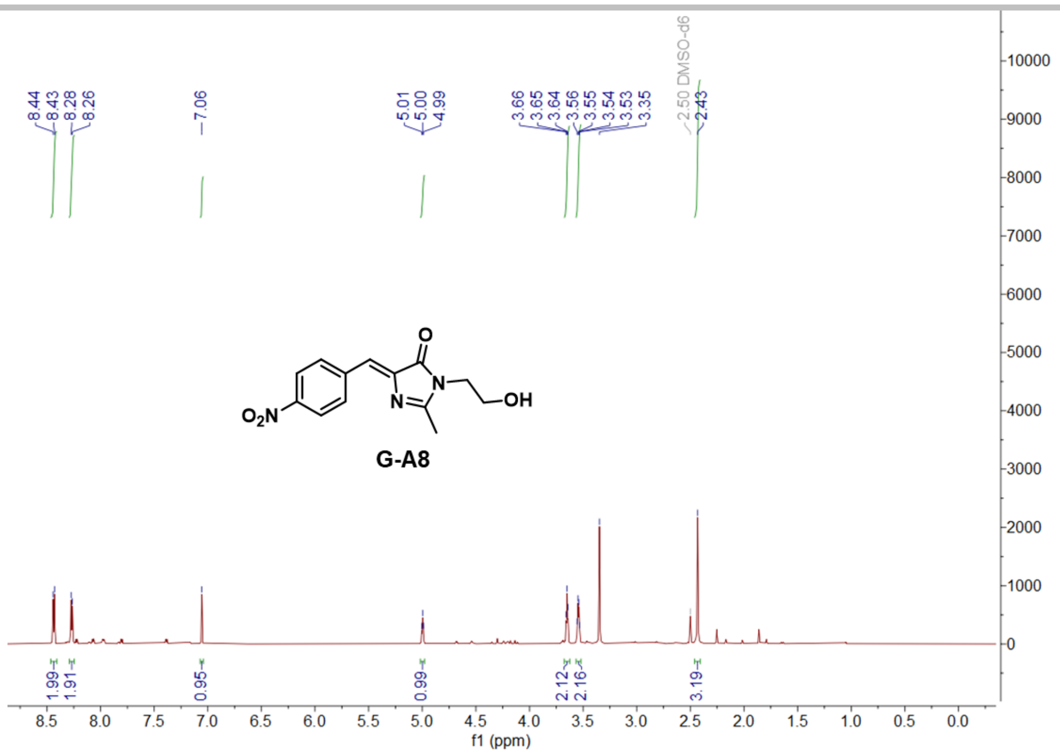

^1H NMR spectrum of G-A8 ($(CD_3)_2SO$)

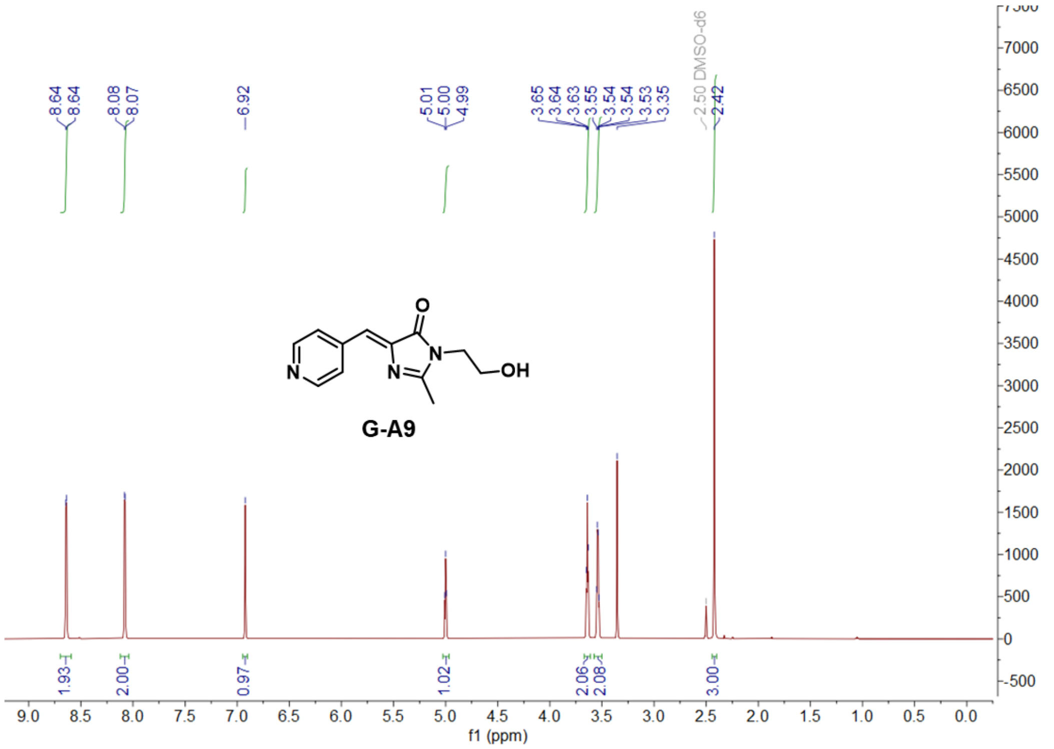

^1H NMR spectrum of G-A9 ($(CD_3)_2SO$)

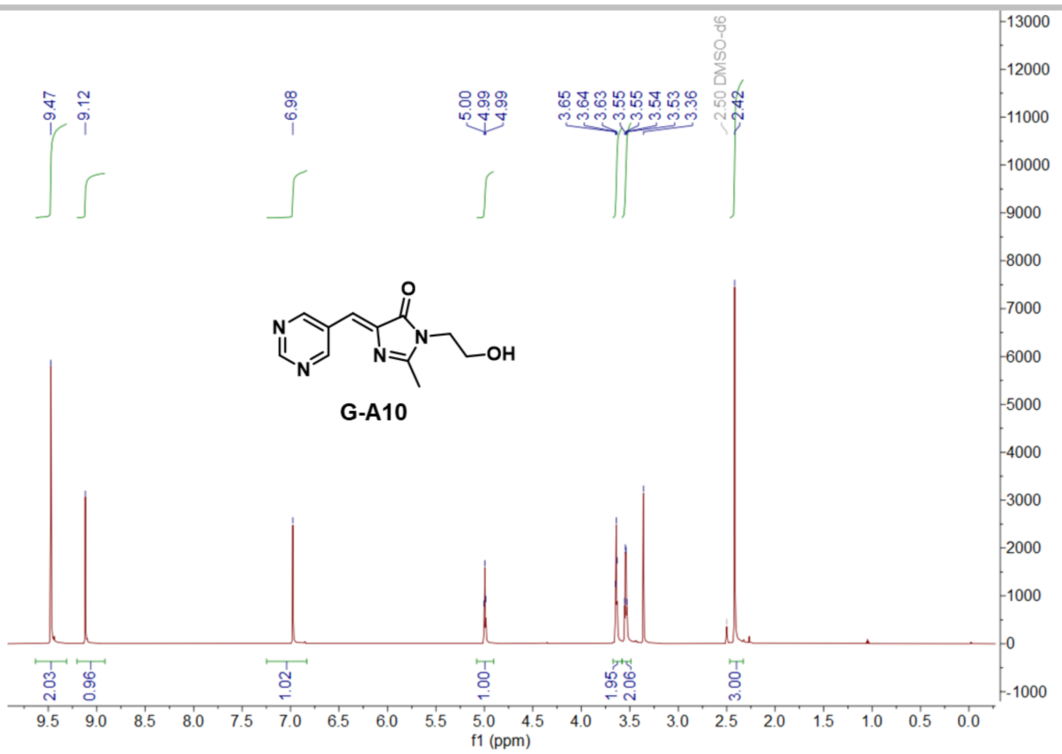

^{1}H NMR spectrum of G-A10 ($(CD_3)_2SO$)

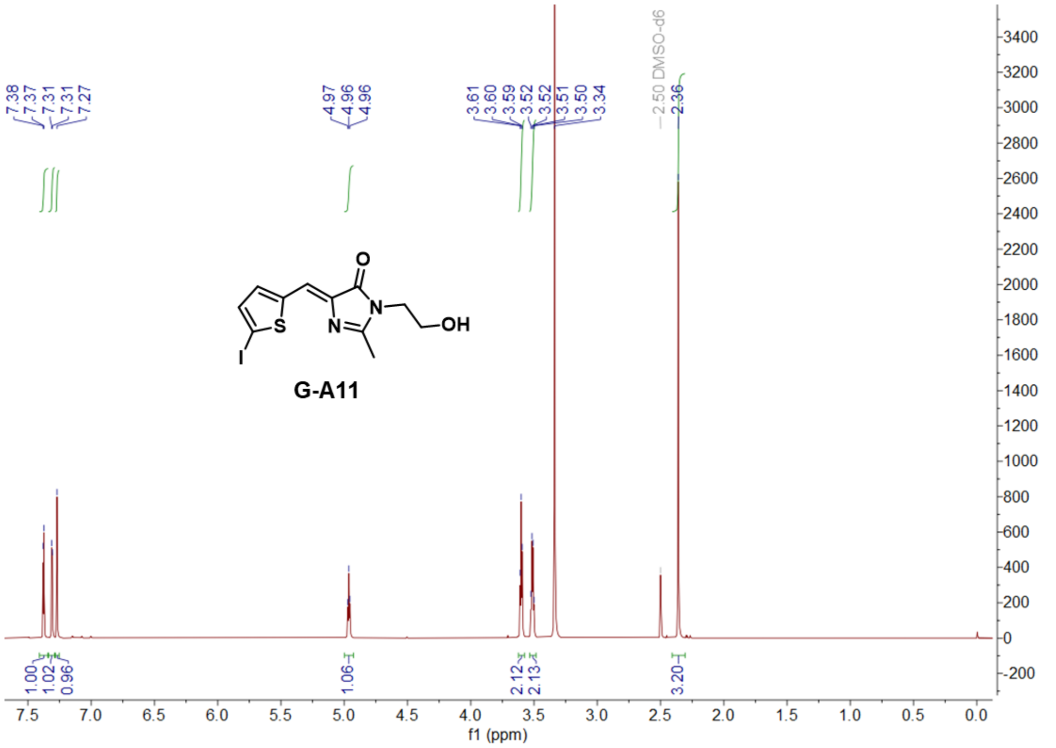

^{1}H NMR spectrum of G-A11 ($(CD_3)_2SO$)

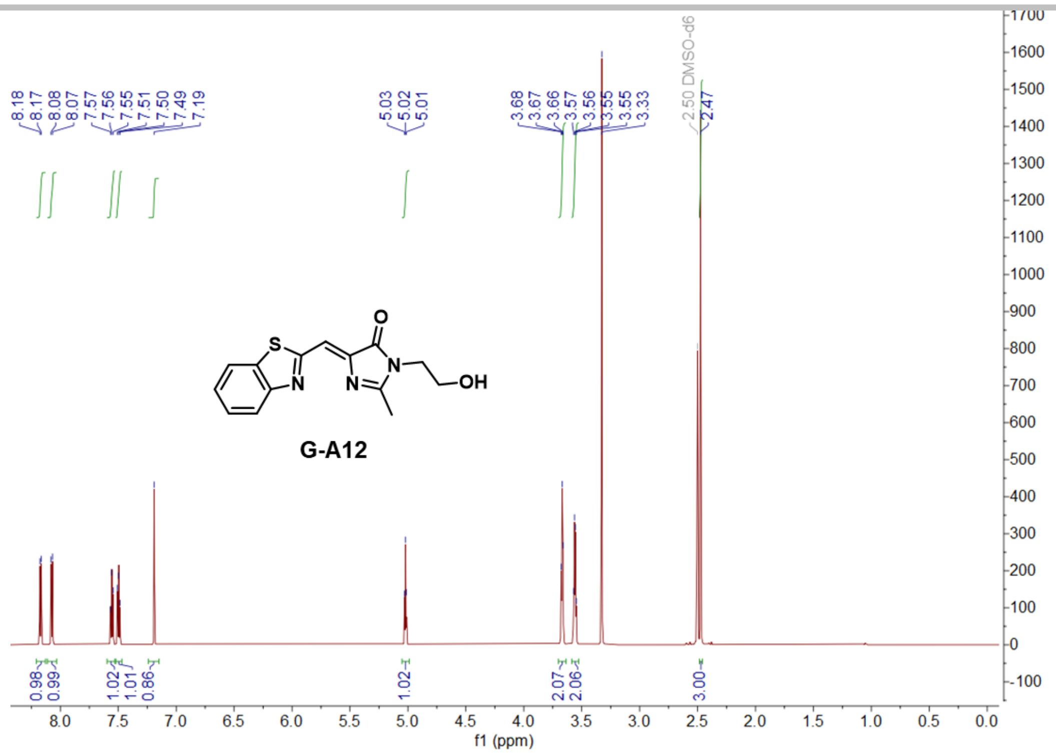

^{1}H NMR spectrum of G-A12 ($(CD_3)_2SO$)

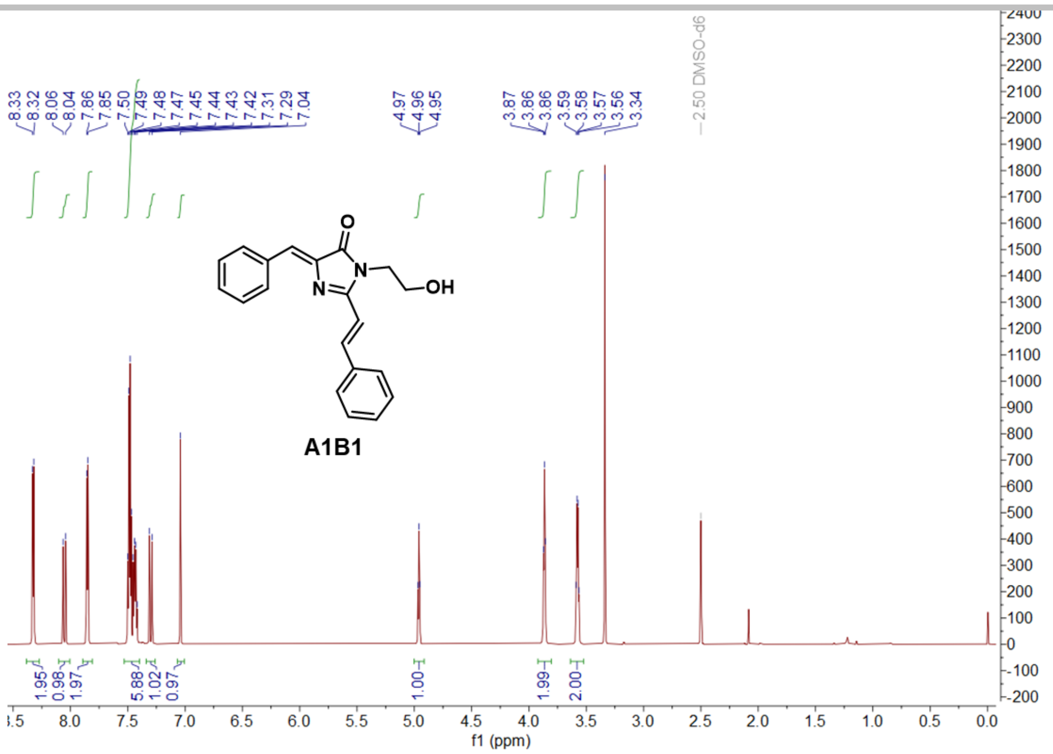

^{1}H NMR spectrum of A1B1 ($(CD_3)_2SO$)

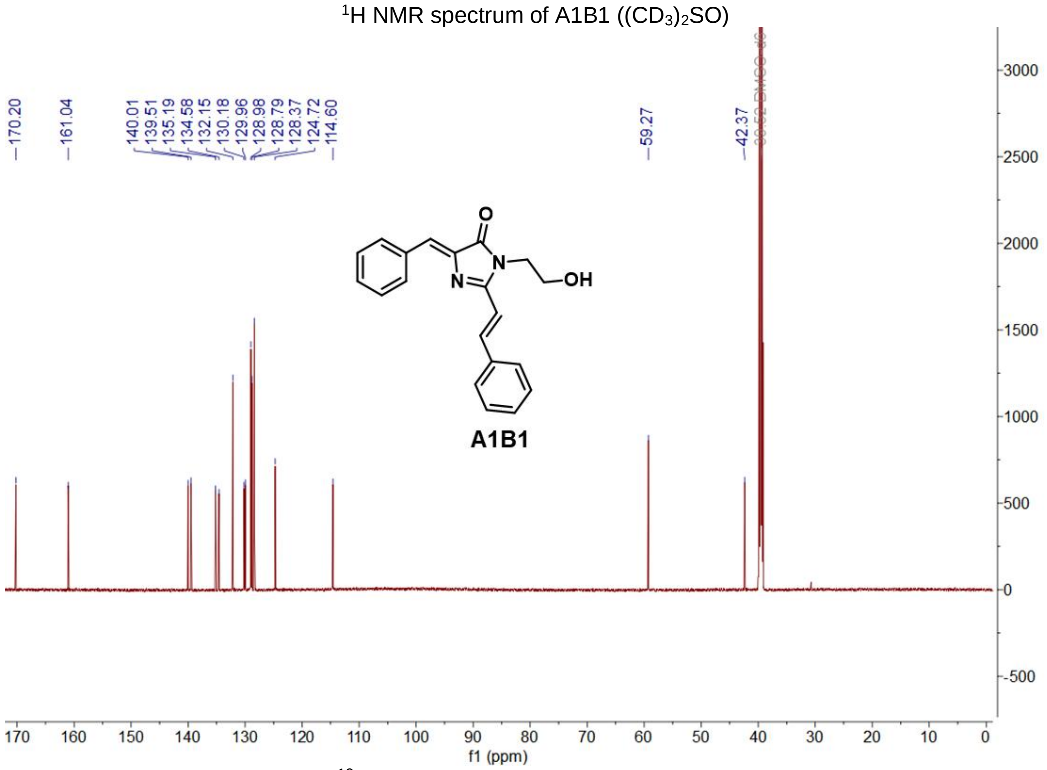

^{13}C NMR spectrum of A1B1 ($(CD_3)_2SO$)

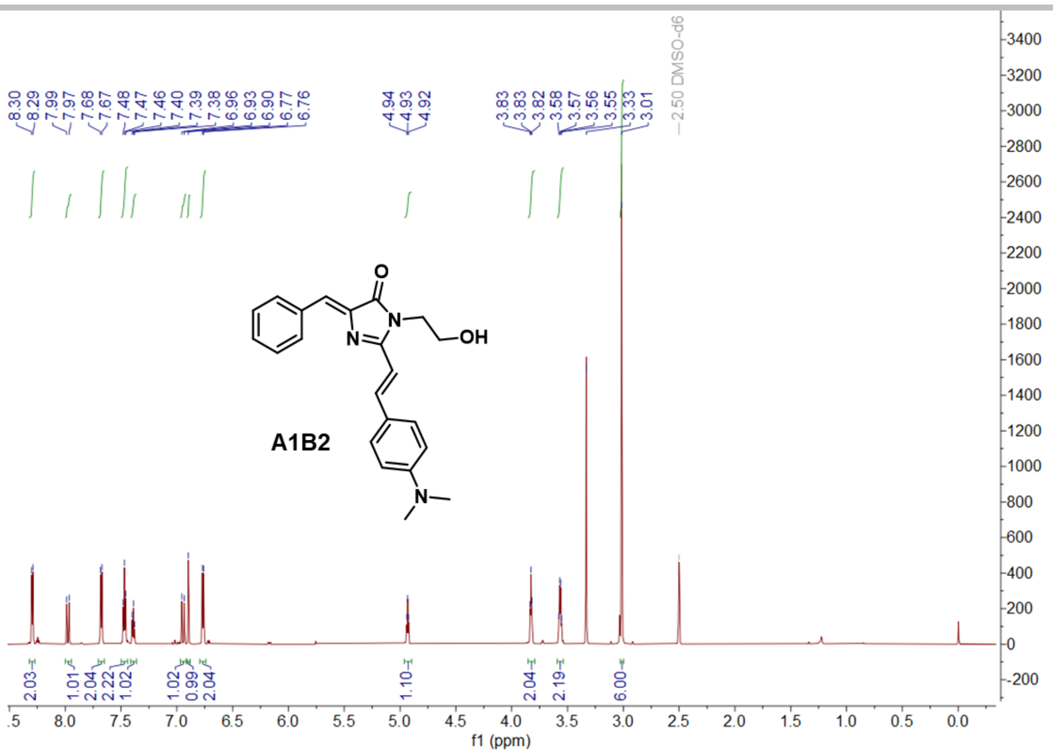

^{1}H NMR spectrum of A1B2 ($(CD_3)_2SO$)

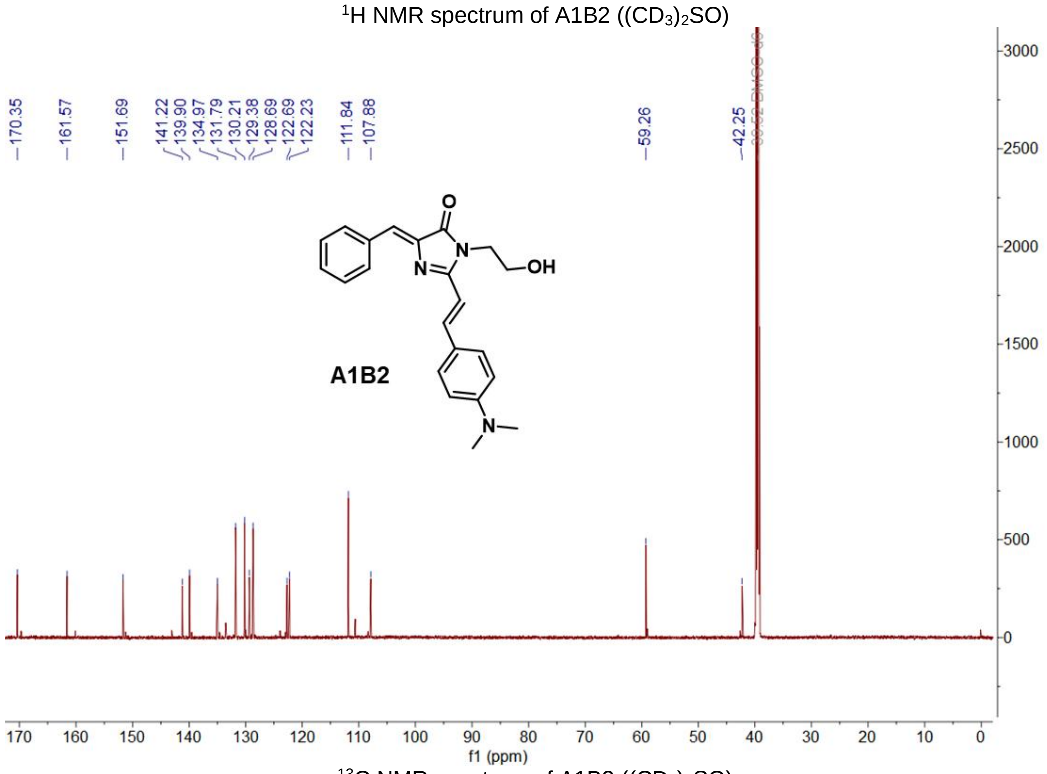

^{13}C NMR spectrum of A1B2 ($(CD_3)_2SO$)

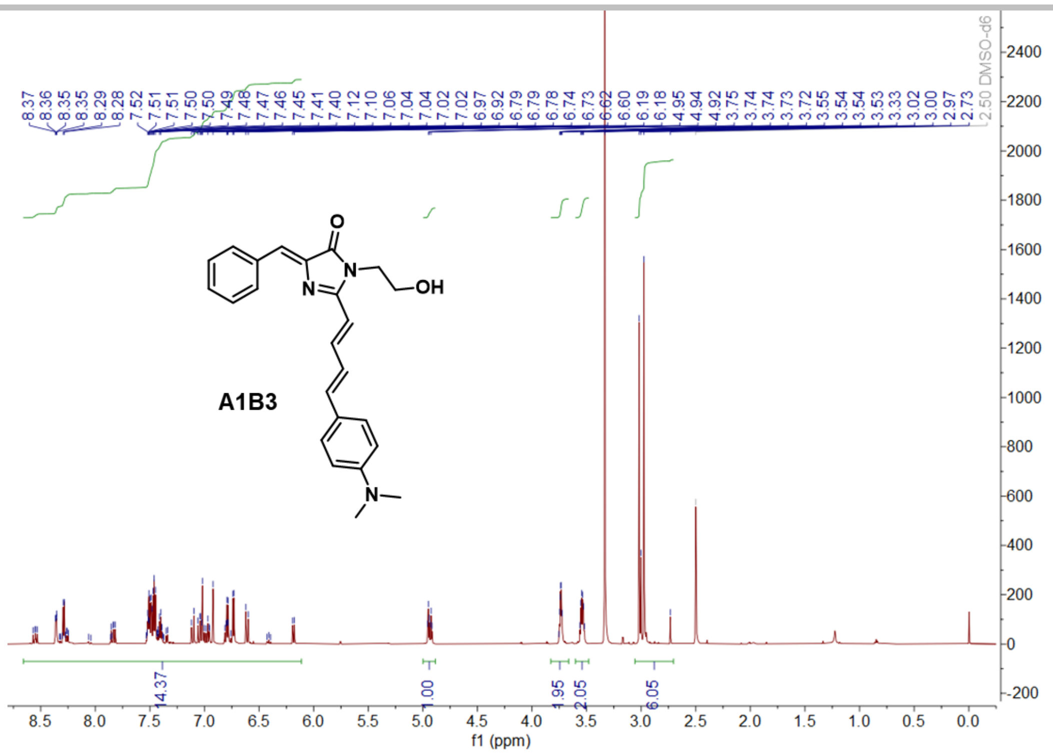

^{1}H NMR spectrum of A1B3 (($(CD_3)_2SO$))

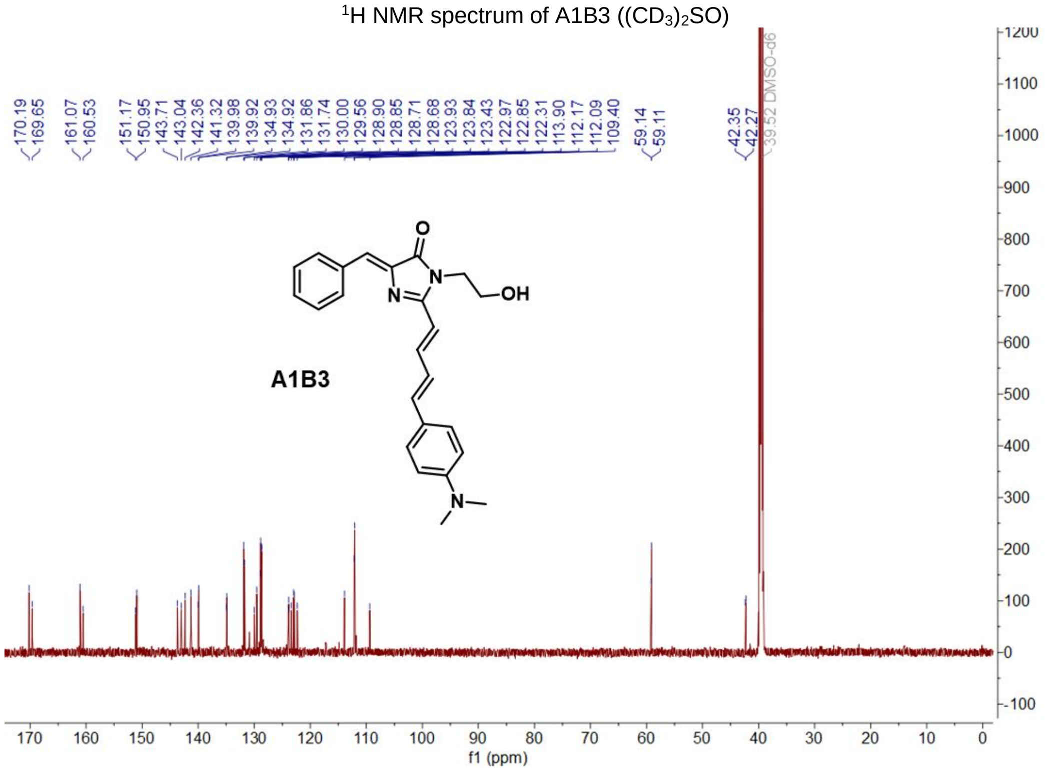

^{13}C NMR spectrum of A1B3 (($(CD_3)_2SO$))

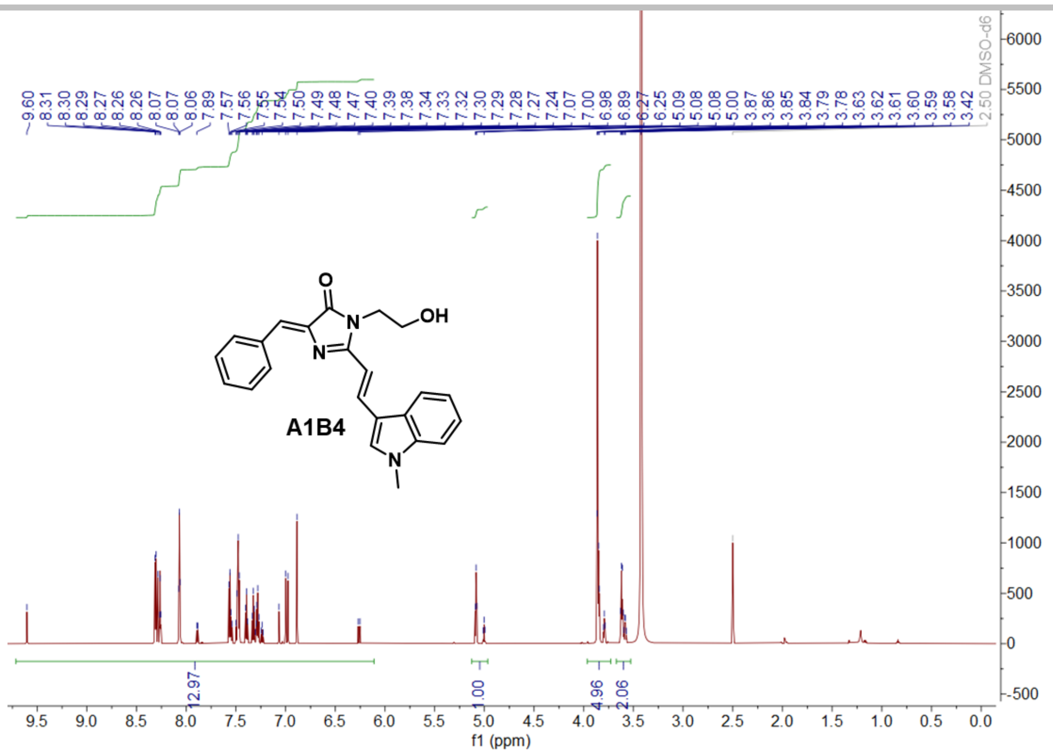

^{1}H NMR spectrum of A1B4 ($(CD_3)_2SO$)

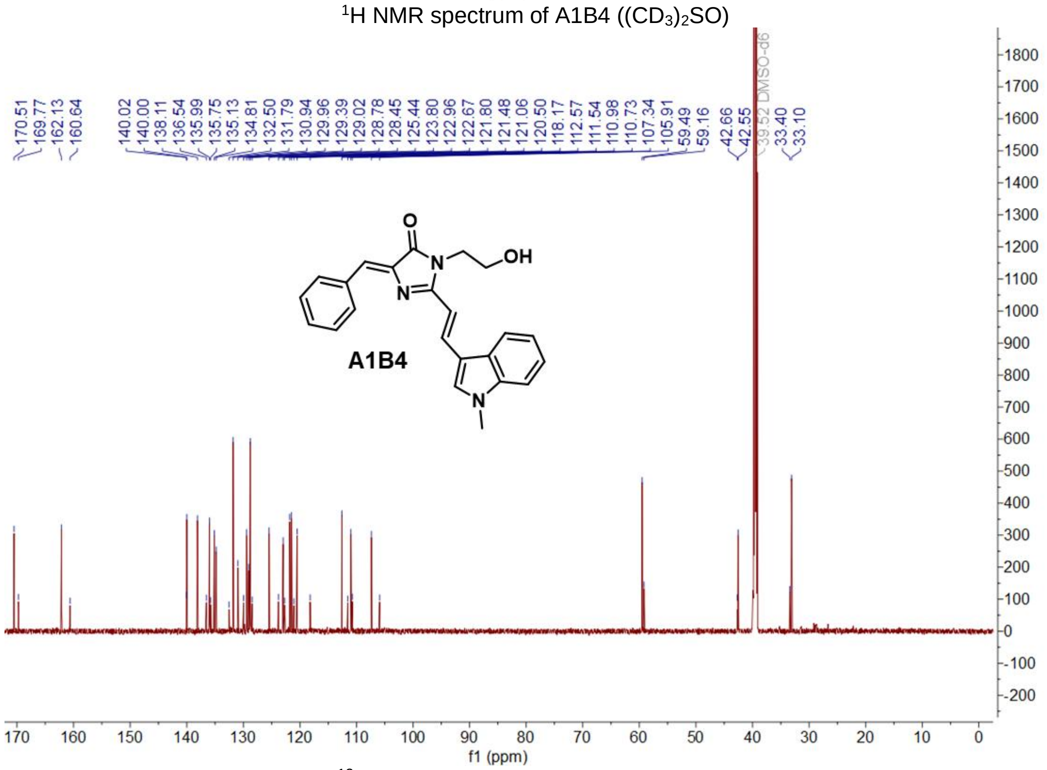

^{13}C NMR spectrum of A1B4 ($(CD_3)_2SO$)

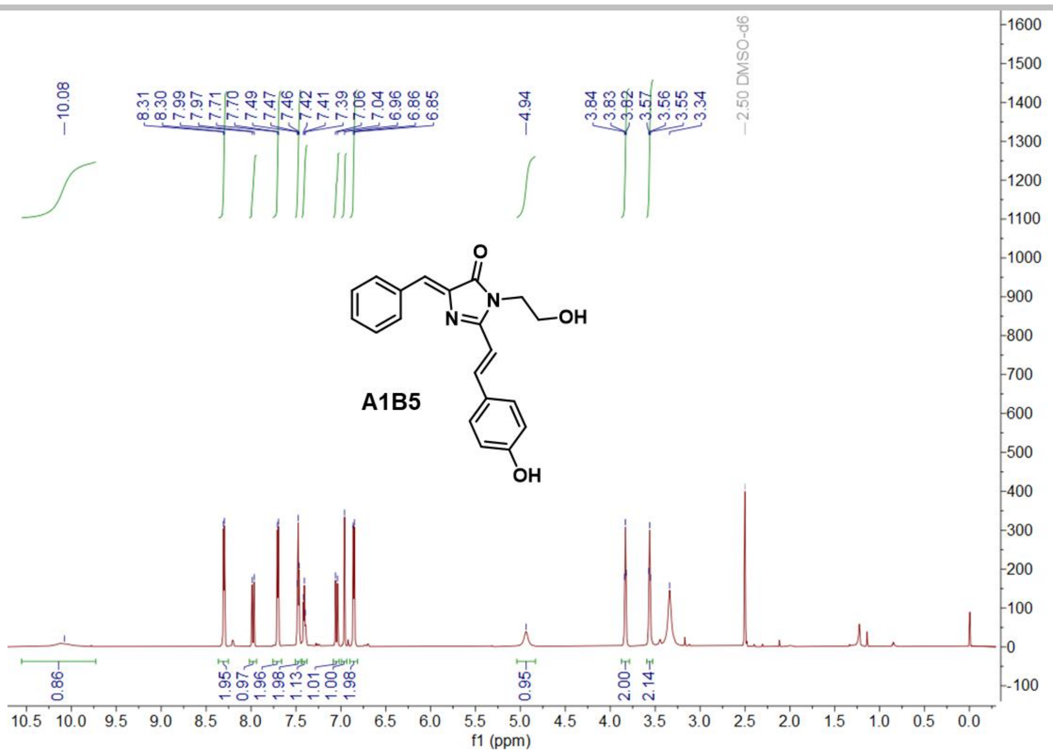

^1H NMR spectrum of A1B5 ($(CD_3)_2SO$)

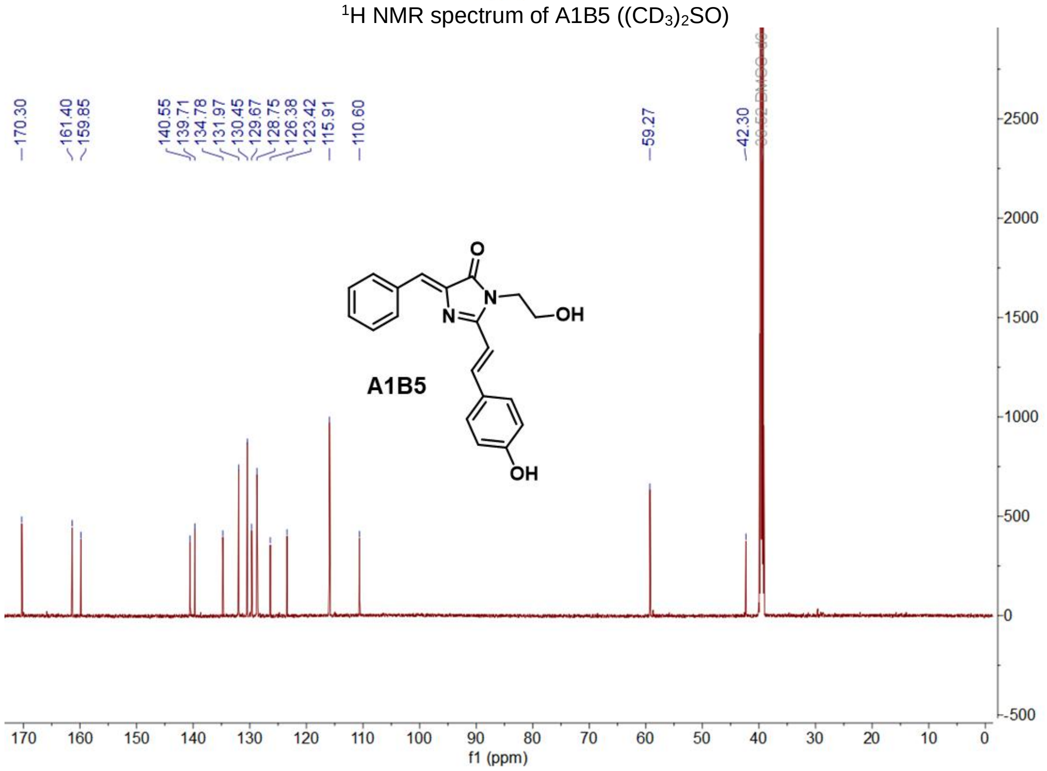

^{13}C NMR spectrum of A1B5 ($(CD_3)_2SO$)

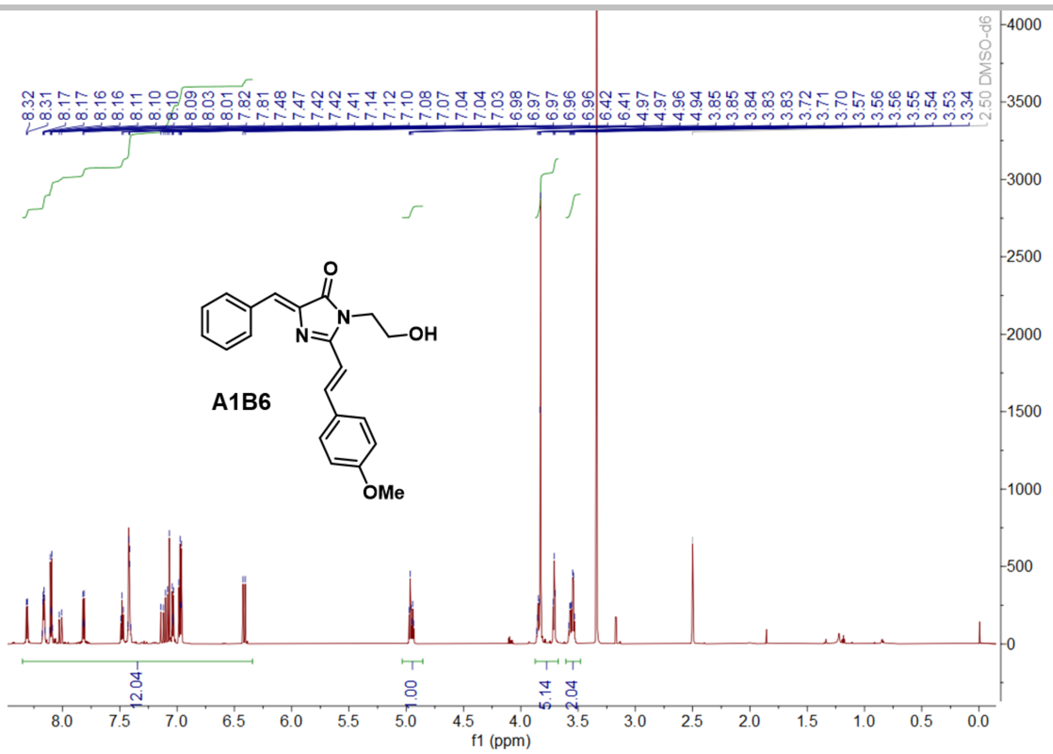

^{1}H NMR spectrum of A1B6 ($(CD_3)_2SO$)

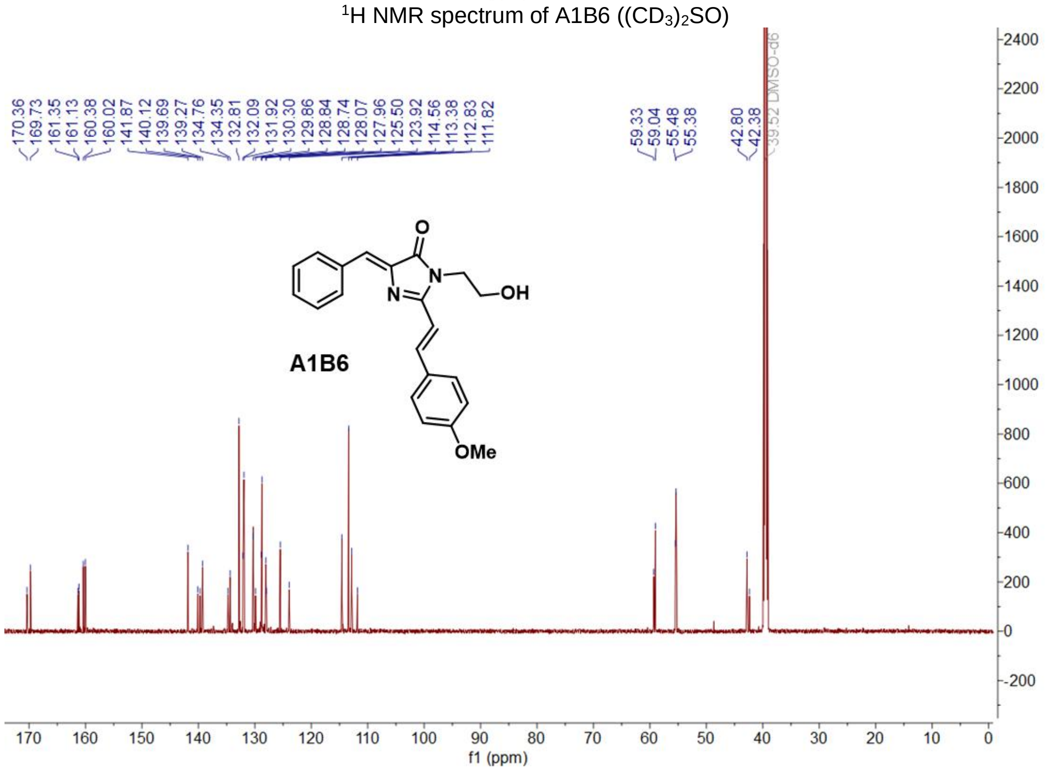

^{13}C NMR spectrum of A1B6 ($(CD_3)_2SO$)

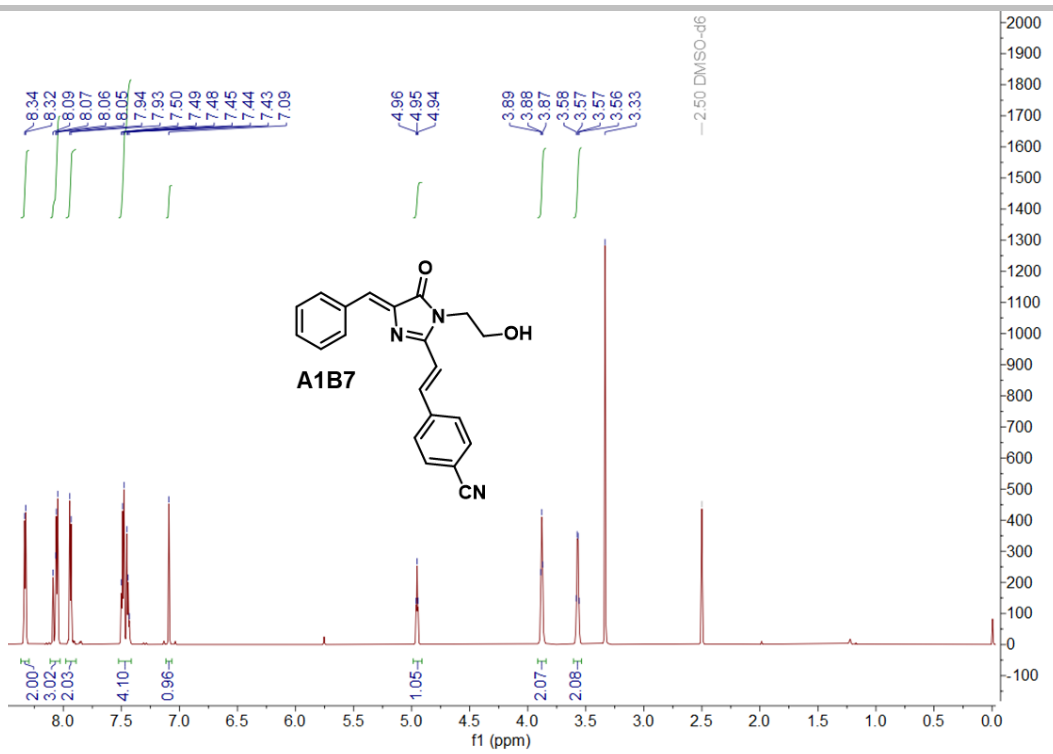

^{1}H NMR spectrum of A1B7 ($(CD_3)_2SO$)

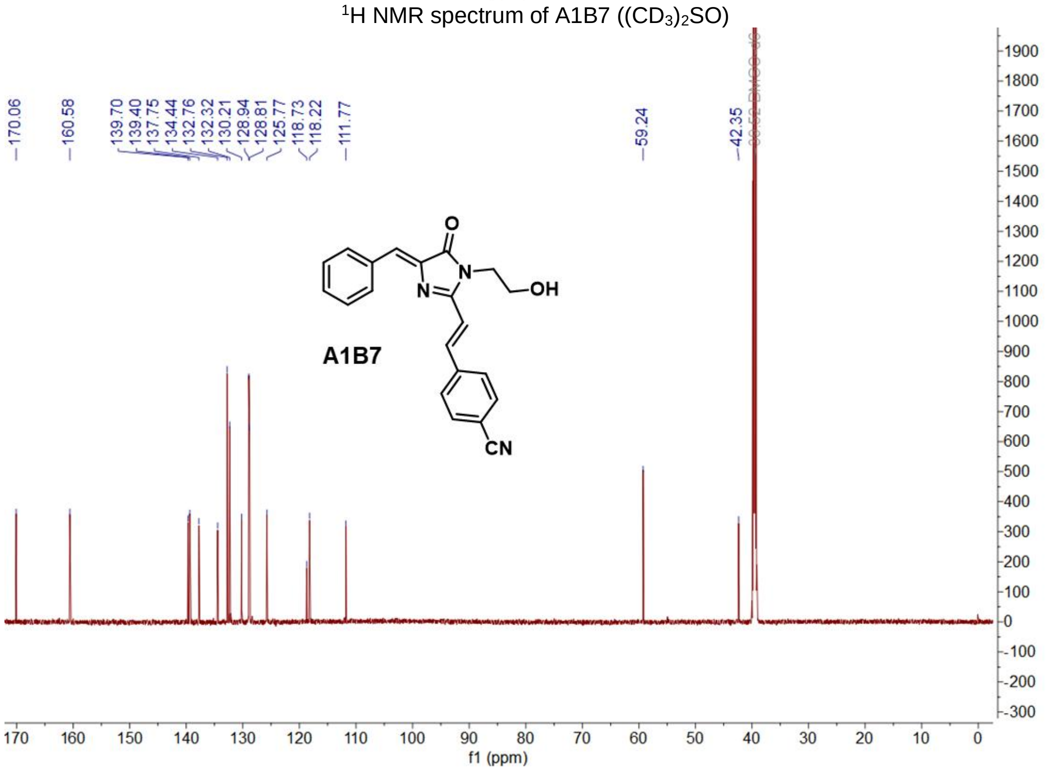

^{13}C NMR spectrum of A1B7 ($(CD_3)_2SO$)

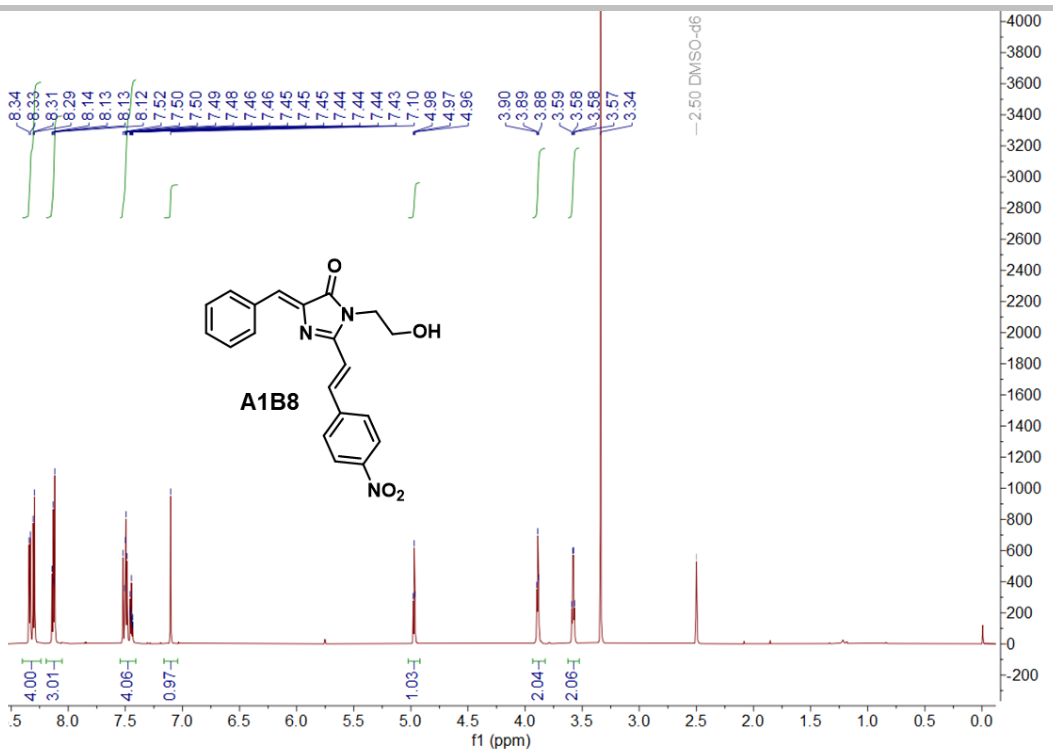

^{1}H NMR spectrum of A1B8 ($(CD_3)_2SO$)

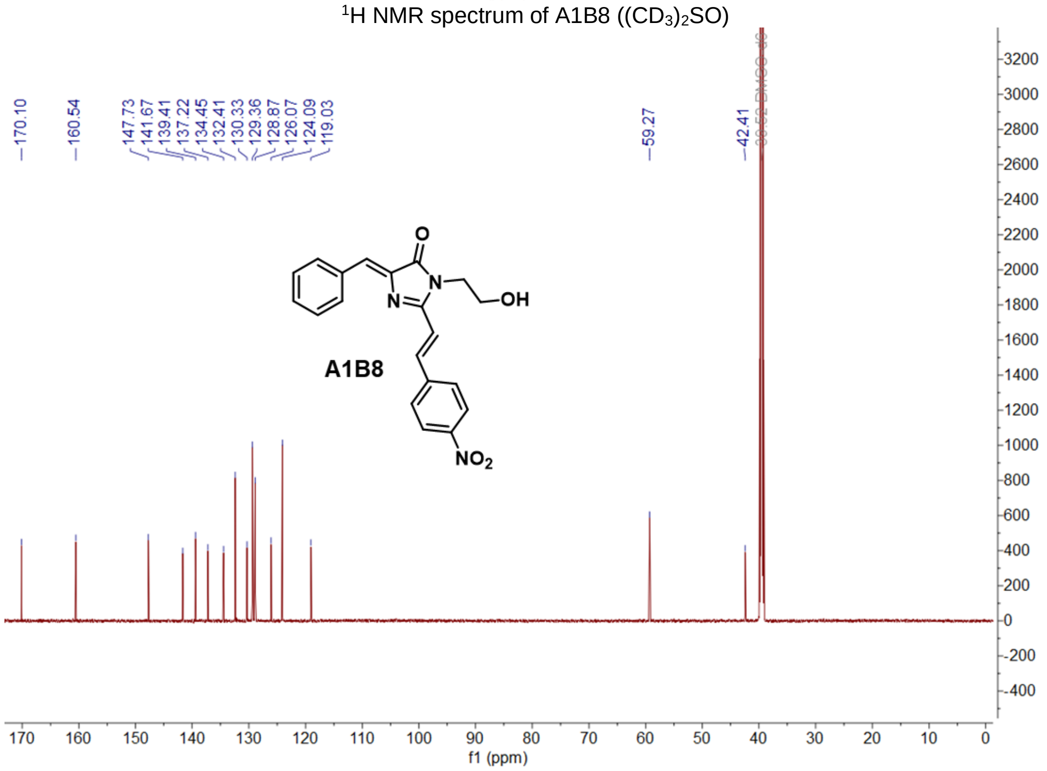

^{13}C NMR spectrum of A1B8 ($(CD_3)_2SO$)

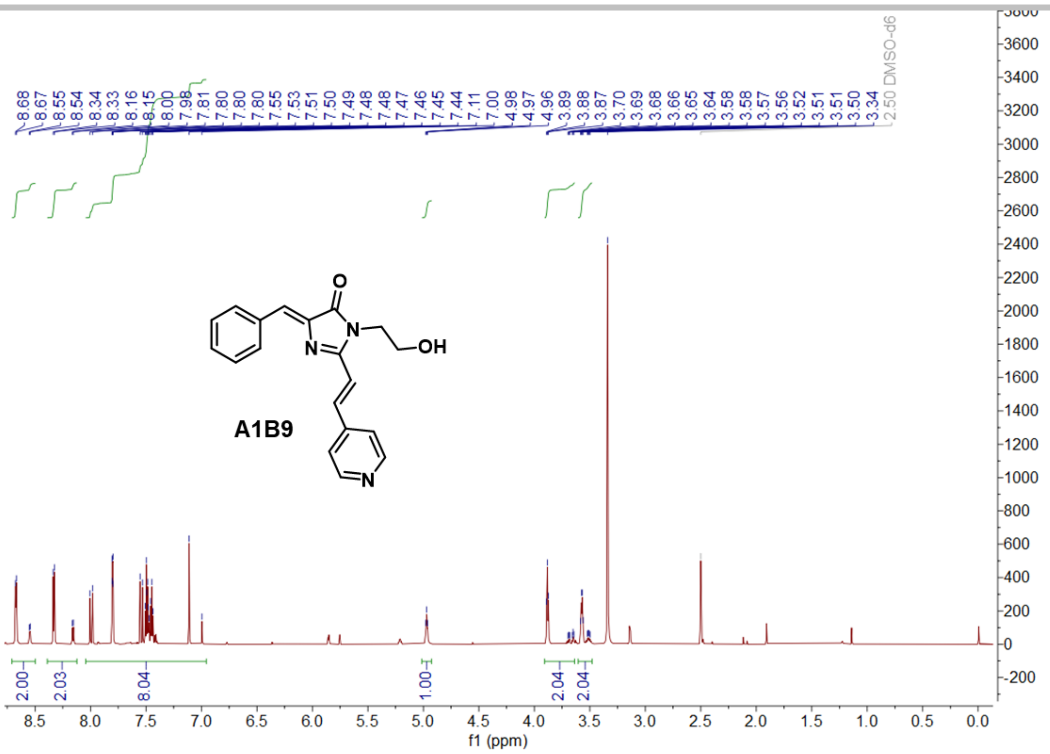

^{1}H NMR spectrum of A1B9 ($(CD_3)_2SO$)

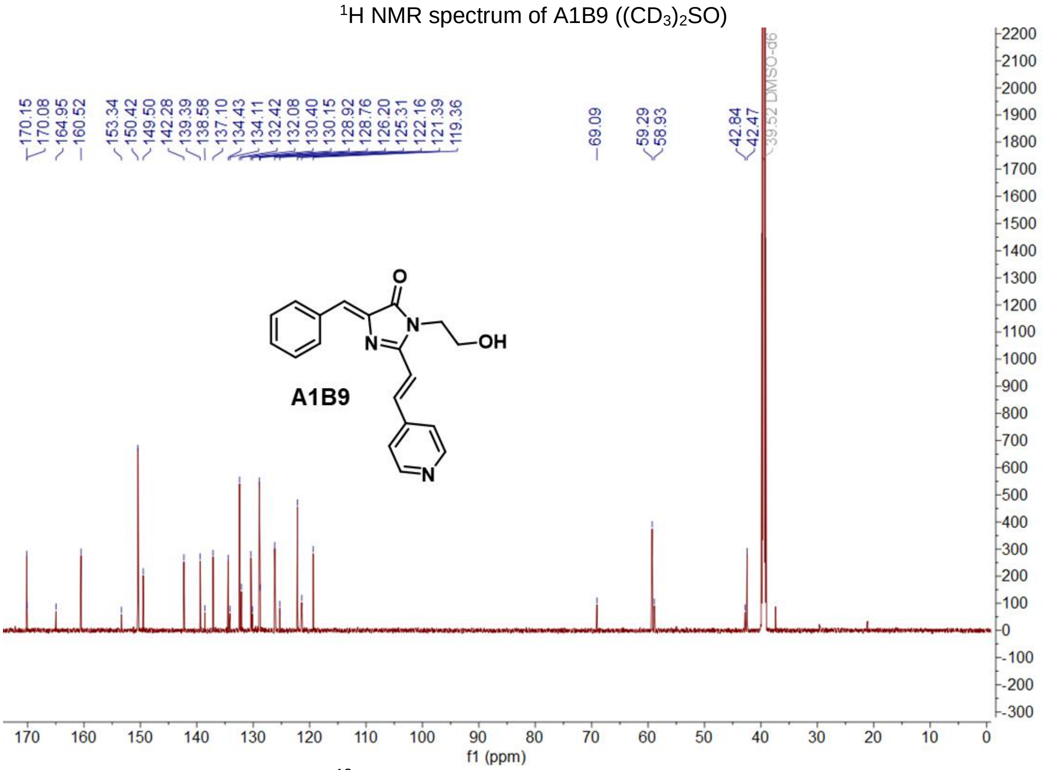

^{13}C NMR spectrum of A1B9 ($(CD_3)_2SO$)

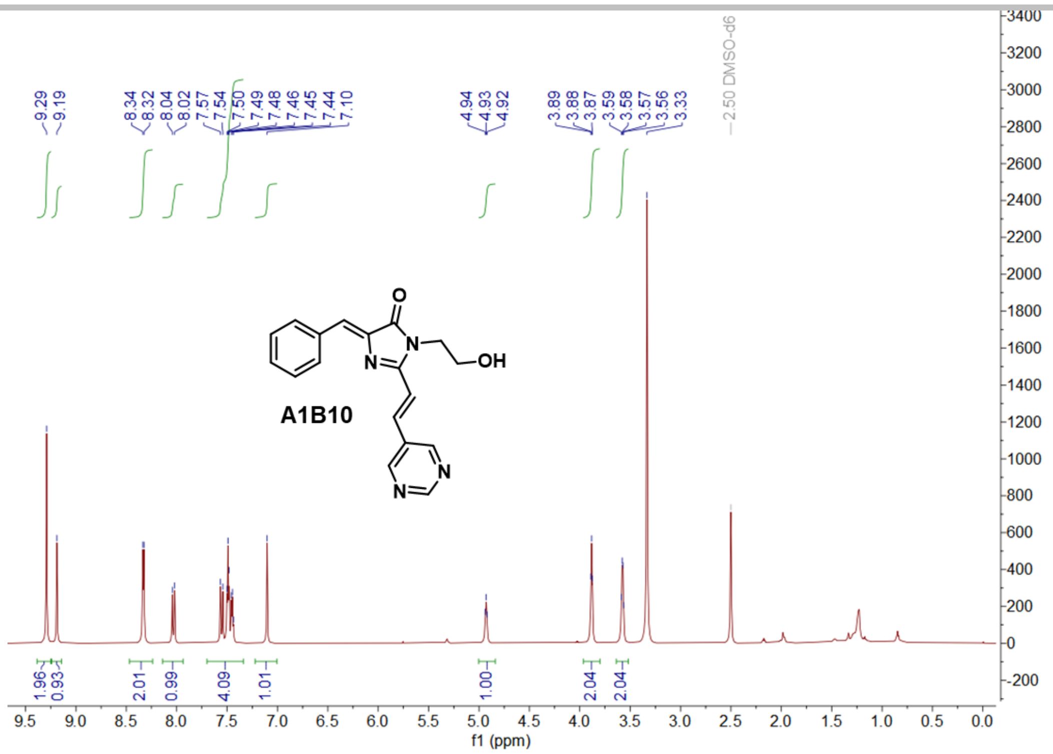

^{1}H NMR spectrum of A1B10 ($(CD_3)_2SO$)

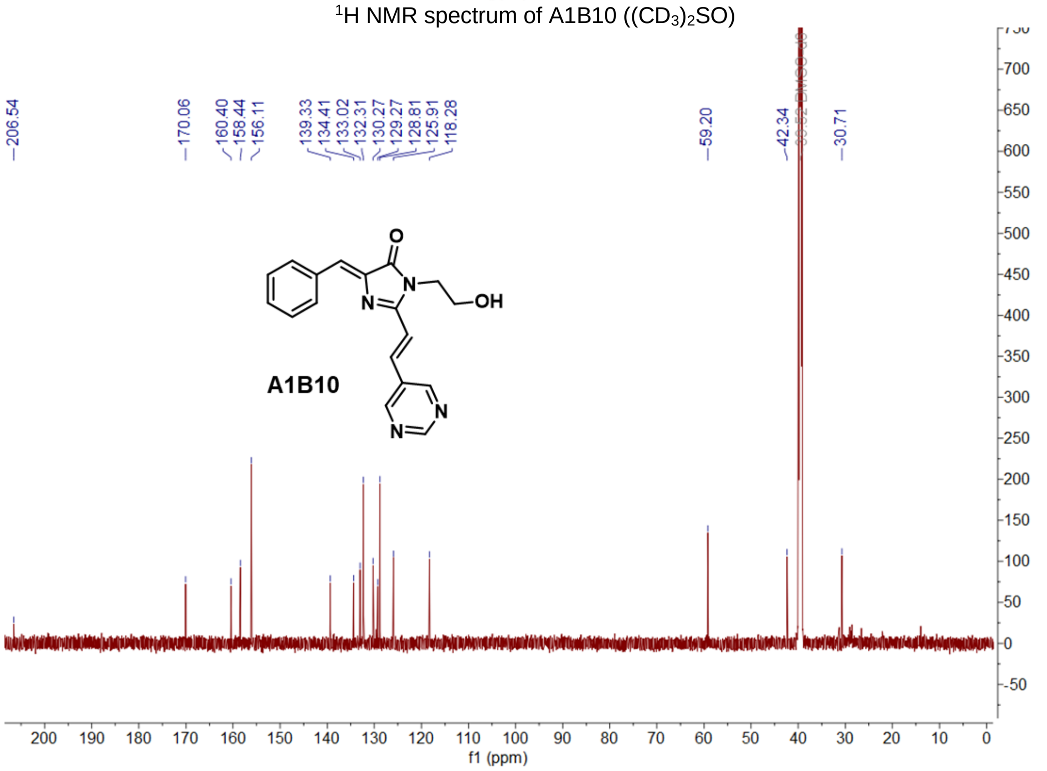

^{13}C NMR spectrum of A1B10 ($(CD_3)_2SO$)

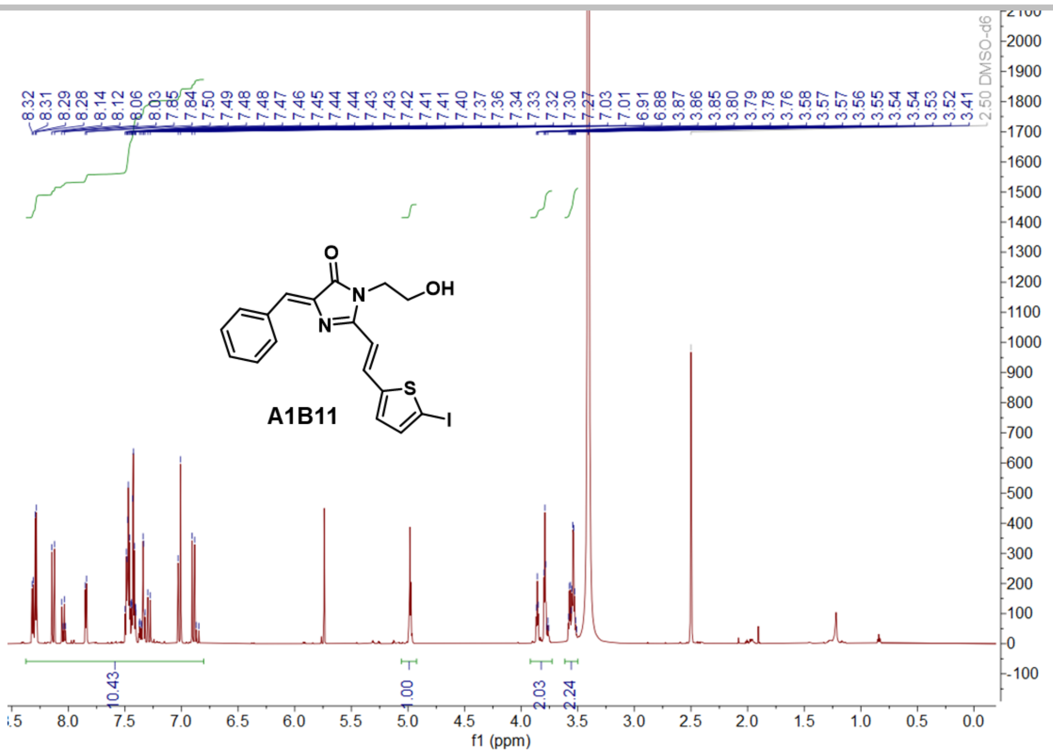

^{1}H NMR spectrum of A1B11 ($(CD_3)_2SO$)

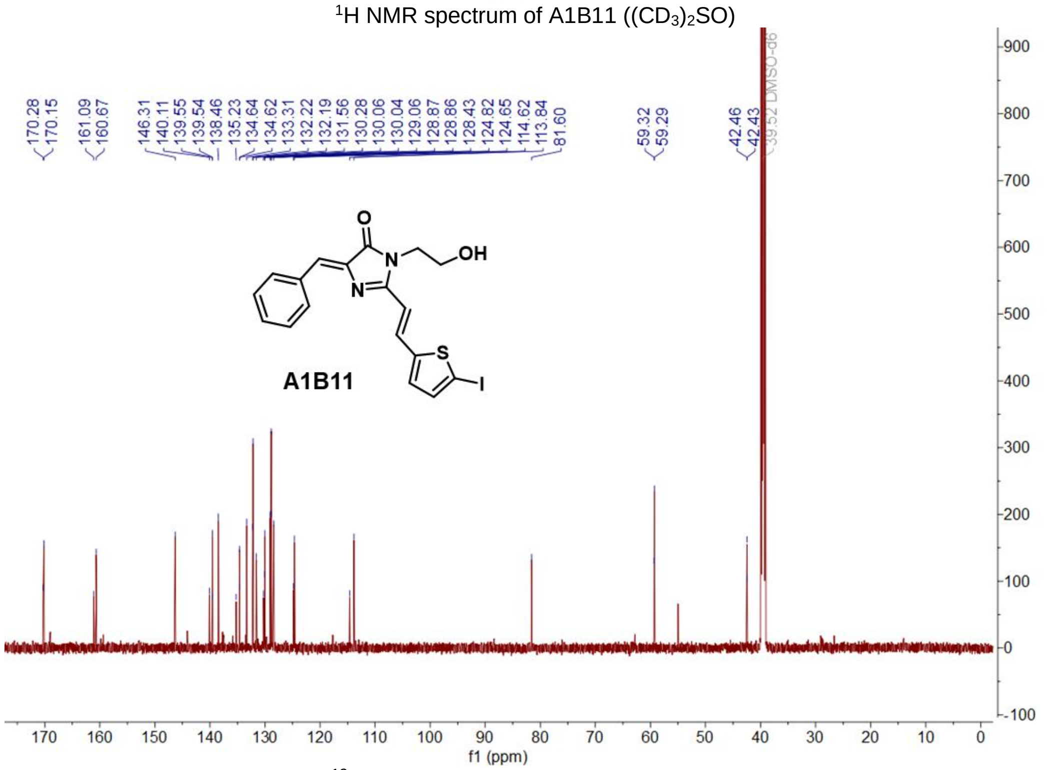

^{13}C NMR spectrum of A1B11 ($(CD_3)_2SO$)

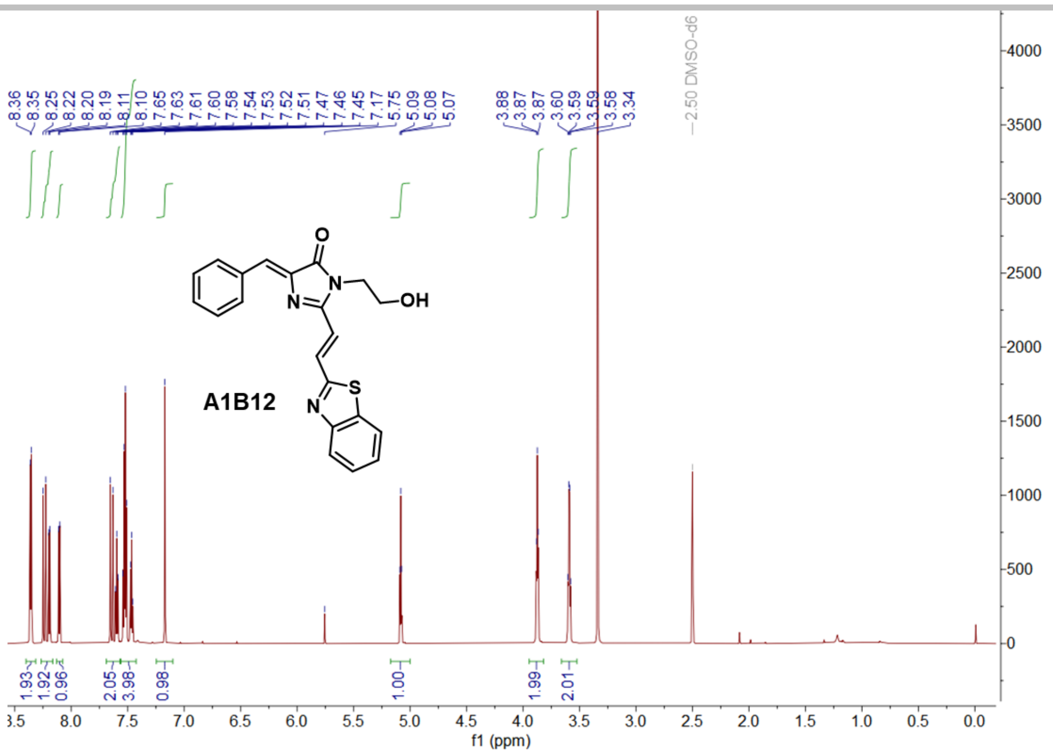

^{1}H NMR spectrum of A1B12 ($(CD_3)_2SO$)

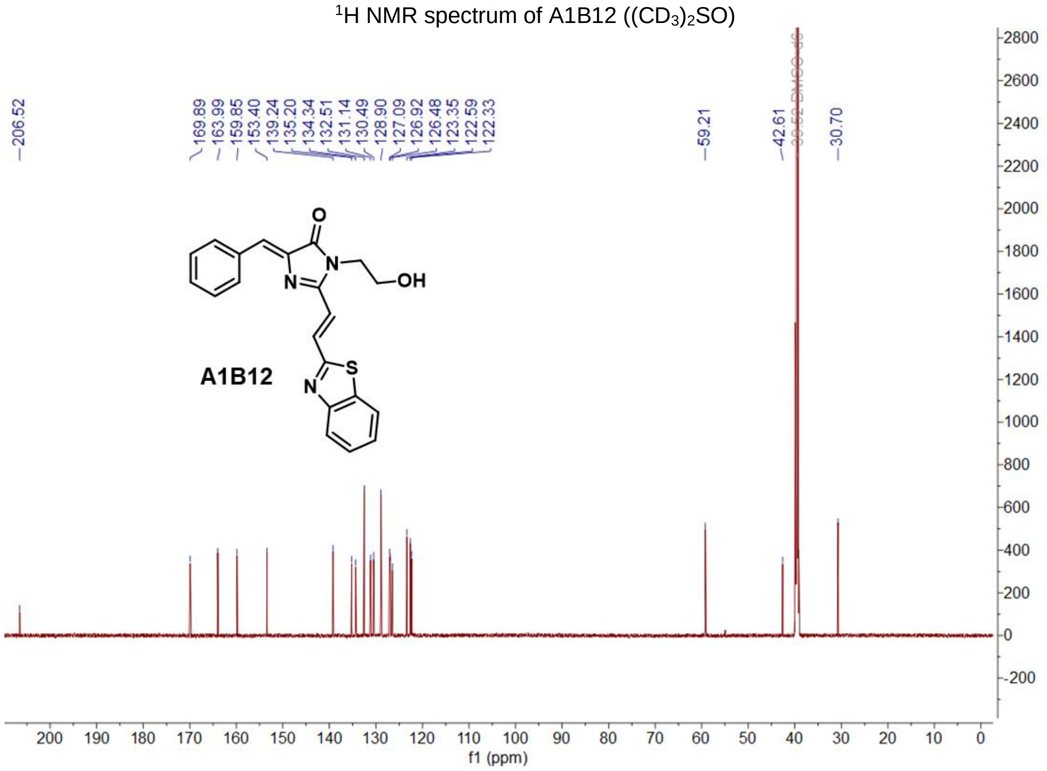

^{13}C NMR spectrum of A1B12 ($(CD_3)_2SO$)

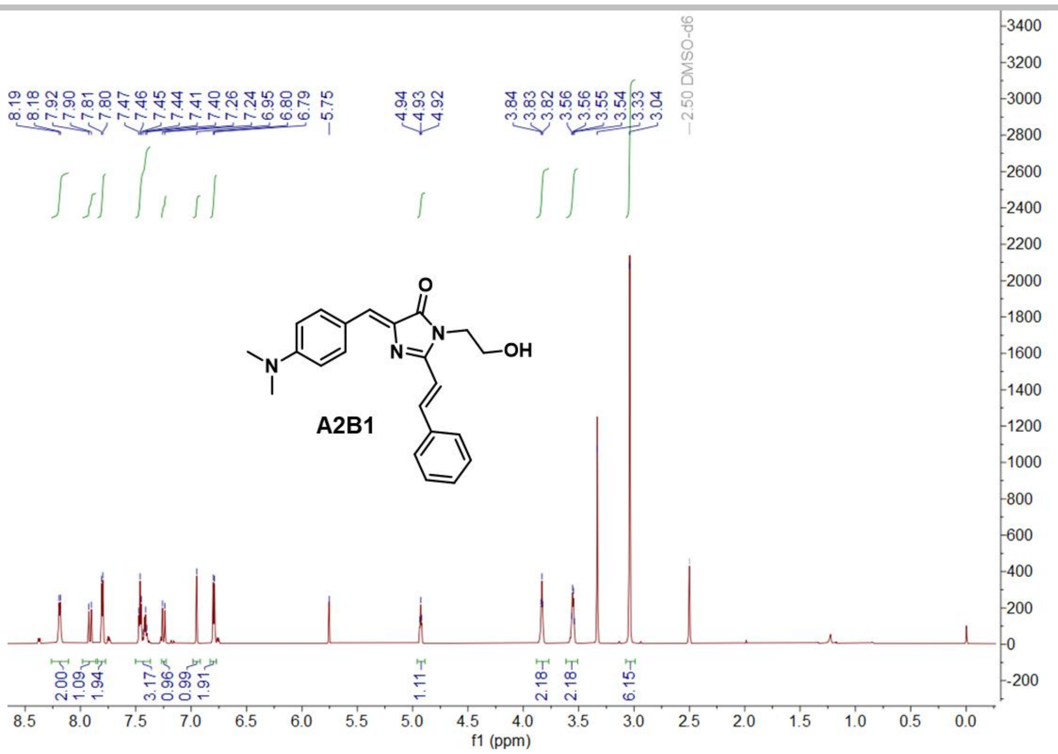

^{1}H NMR spectrum of A2B1 ($(CD_3)_2SO$)

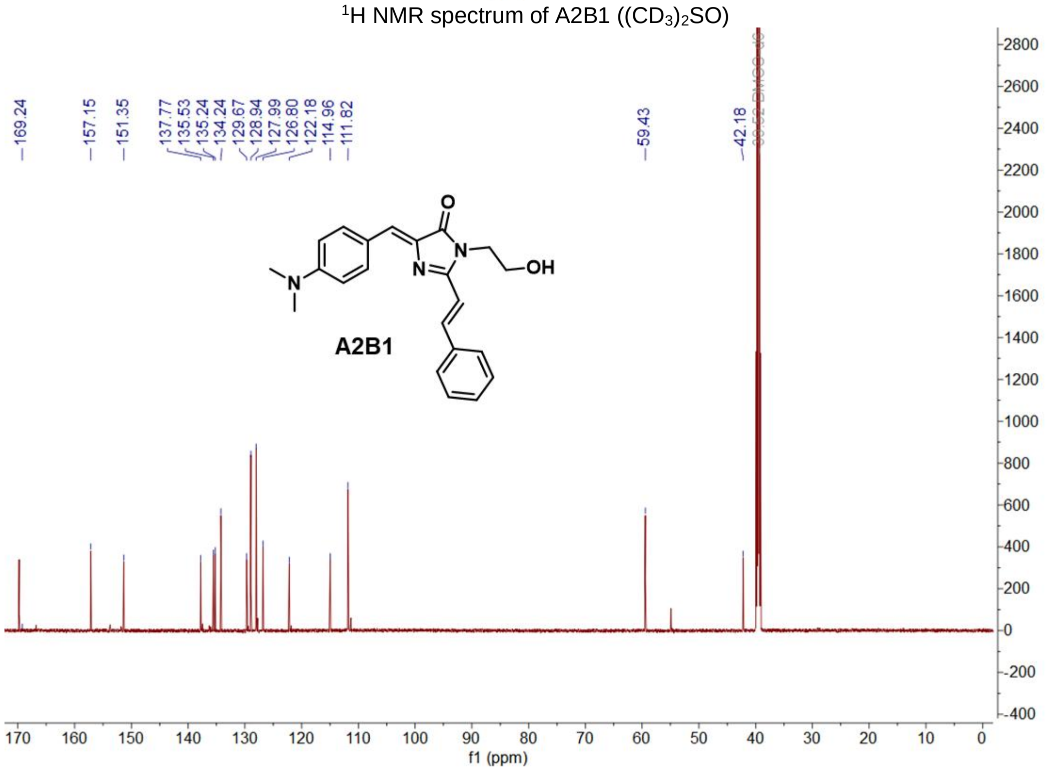

^{13}C NMR spectrum of A2B1 ($(CD_3)_2SO$)

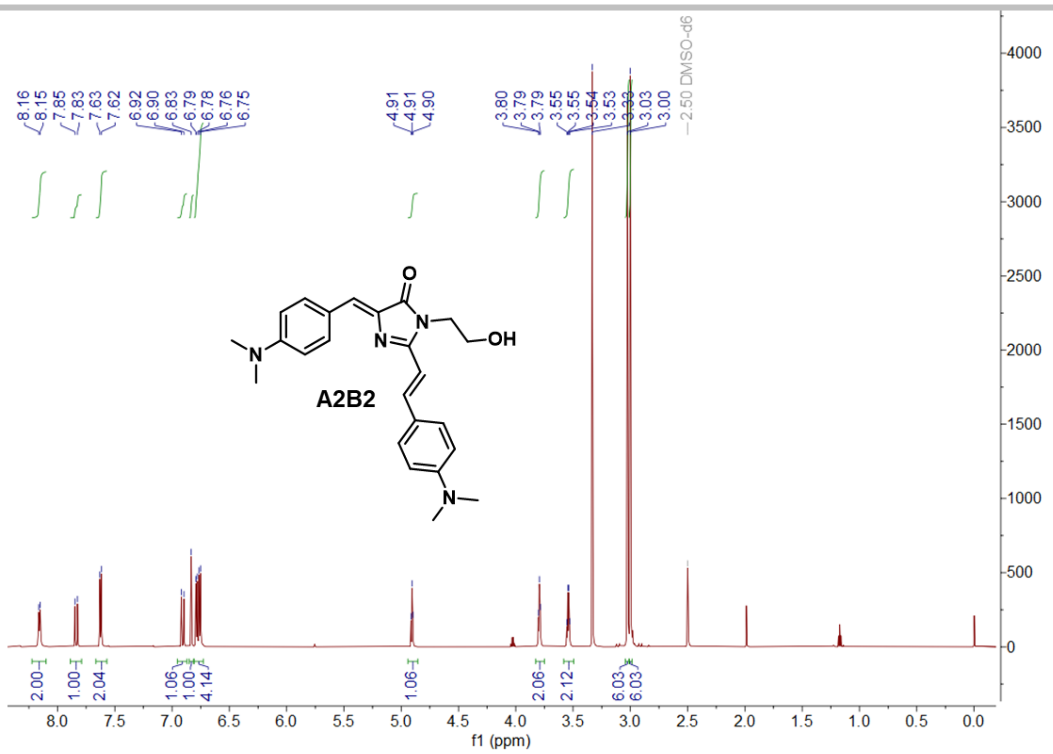

^{1}H NMR spectrum of A2B2 ($(CD_3)_2SO$)

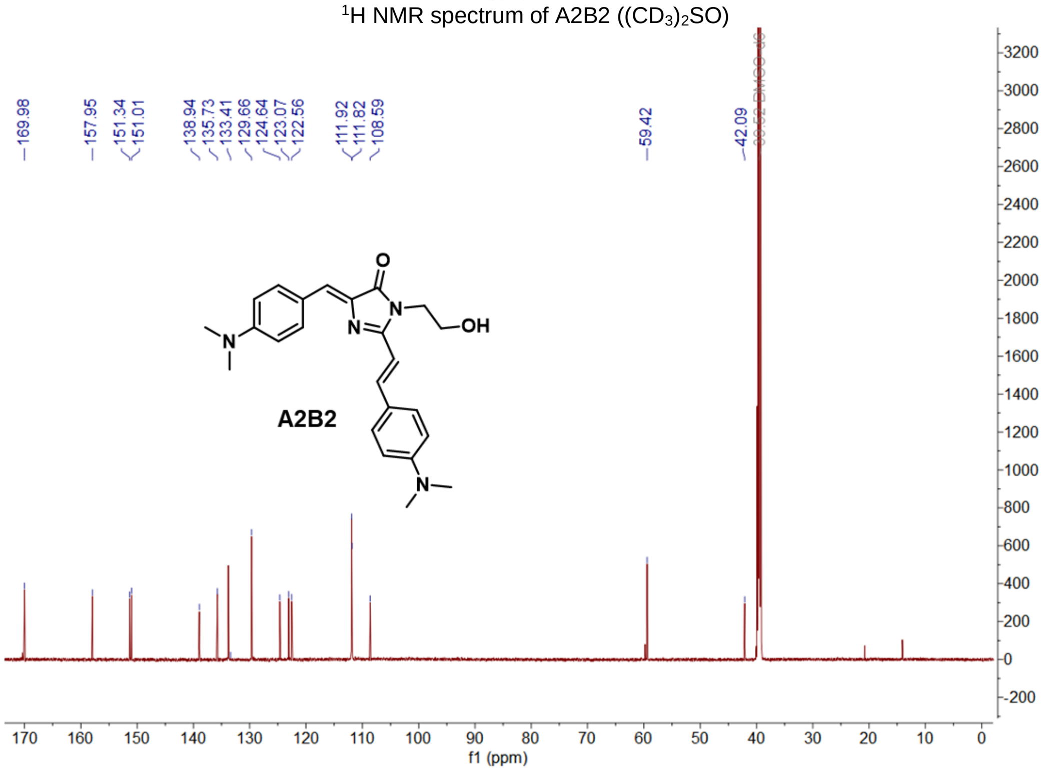

^{13}C NMR spectrum of A2B2 ($(CD_3)_2SO$)

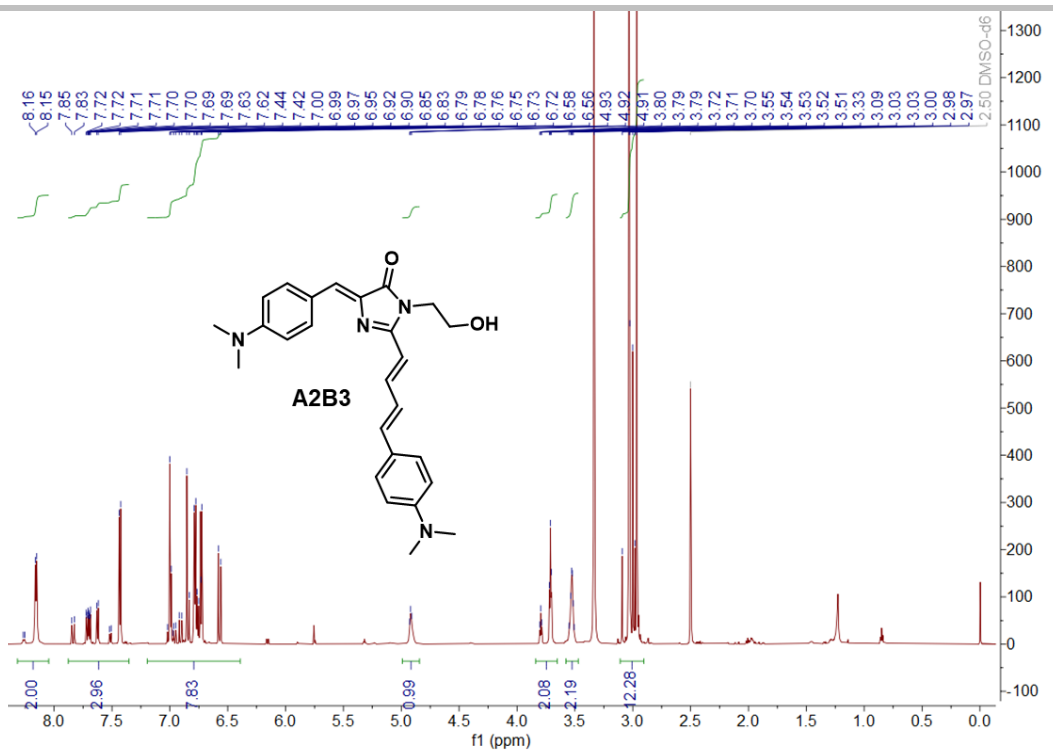

^{1}H NMR spectrum of A2B3 ($(CD_3)_2SO$)

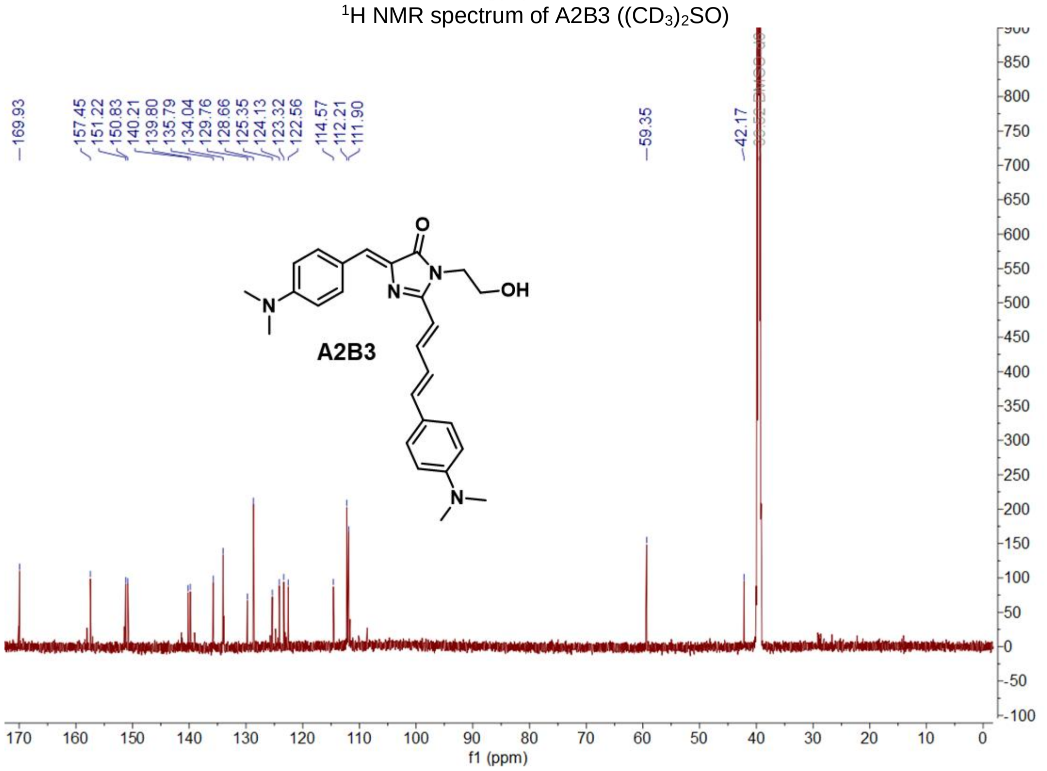

^{13}C NMR spectrum of A2B3 ($(CD_3)_2SO$)

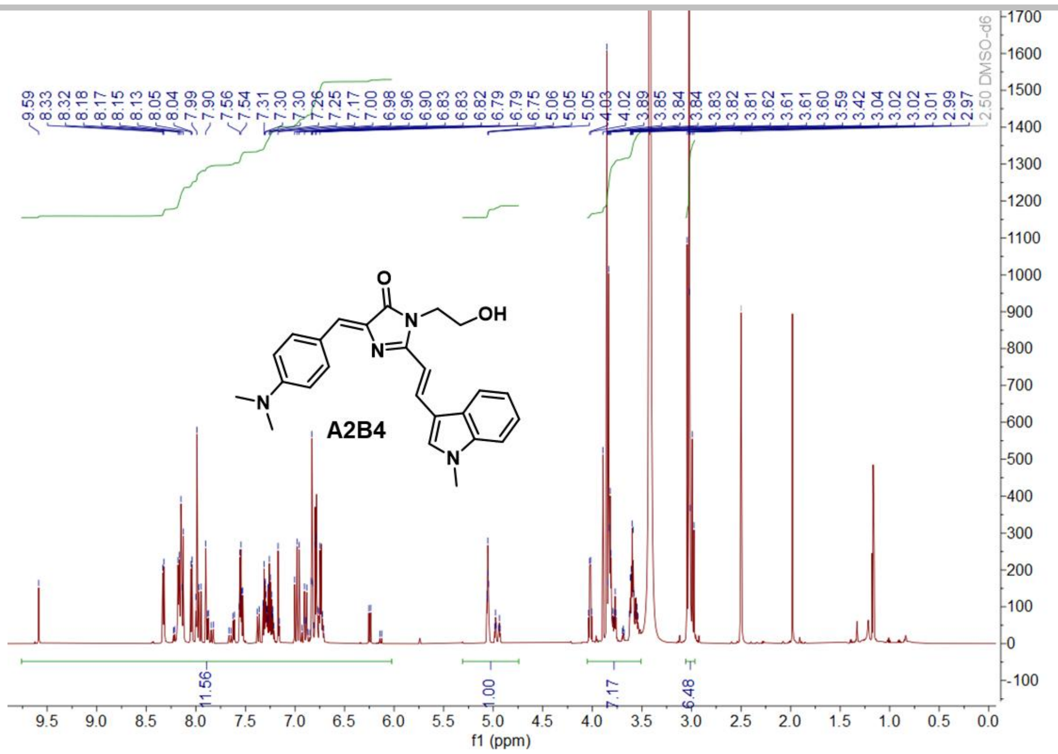

^{1}H NMR spectrum of A2B4 ($(CD_3)_2SO$)

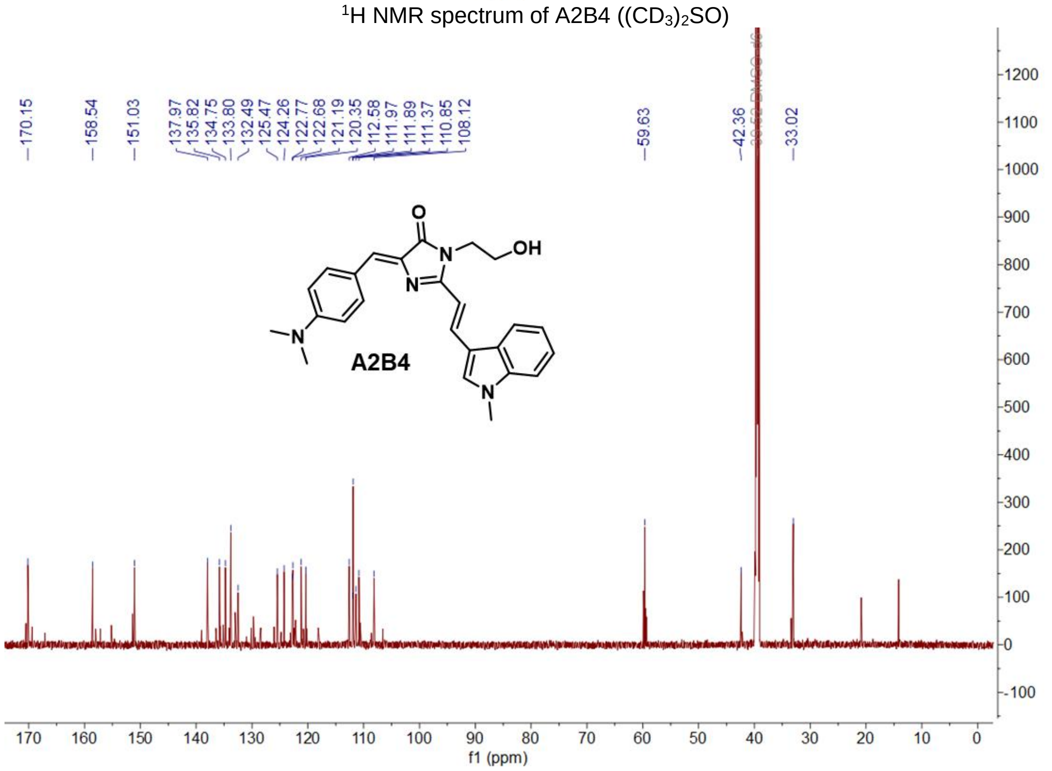

^{13}C NMR spectrum of A2B4 ($(CD_3)_2SO$)

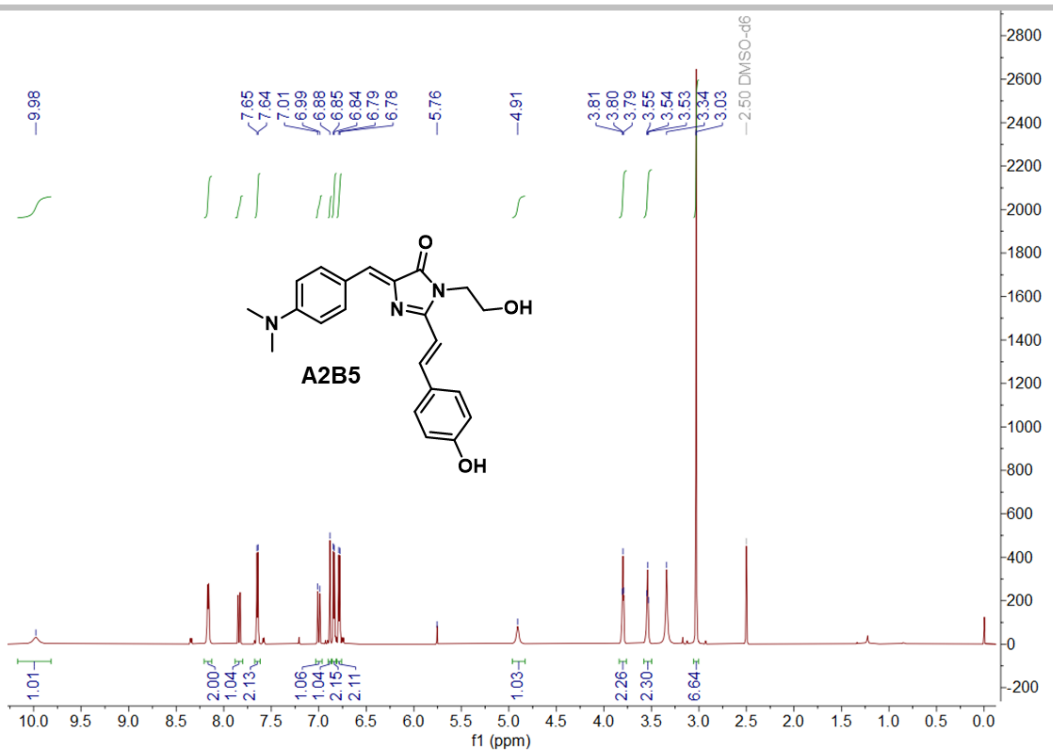

^{1}H NMR spectrum of A2B5 ($(CD_3)_2SO$)

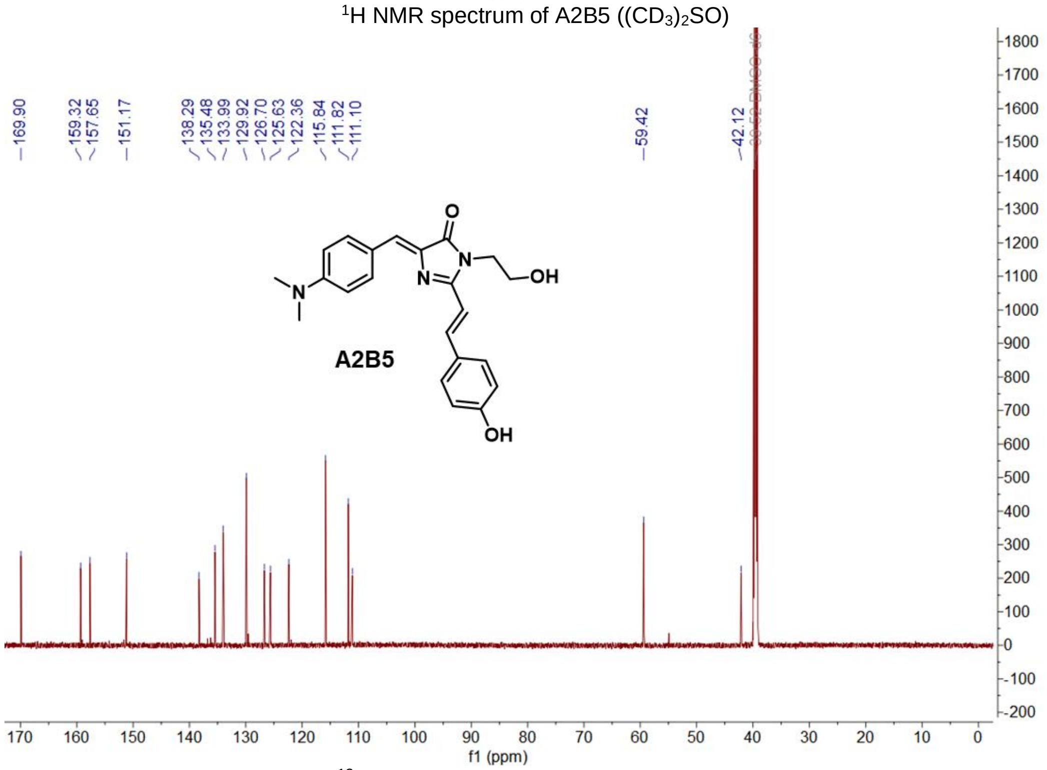

^{13}C NMR spectrum of A2B5 ($(CD_3)_2SO$)

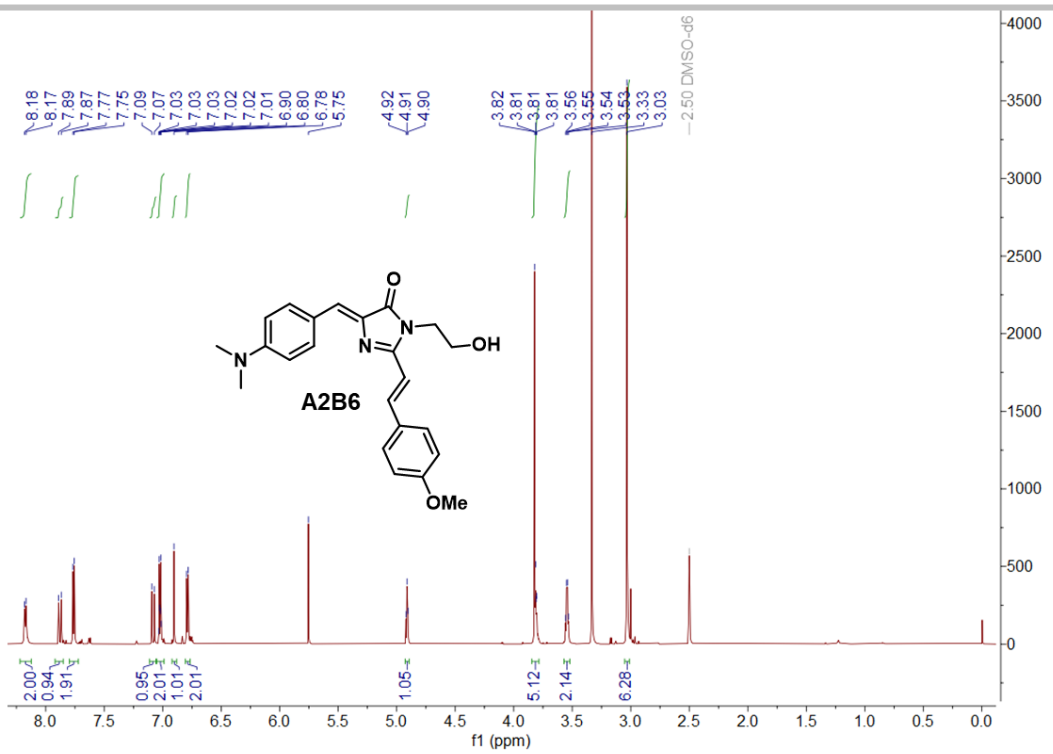

^{1}H NMR spectrum of A2B6 ($(CD_3)_2SO$)

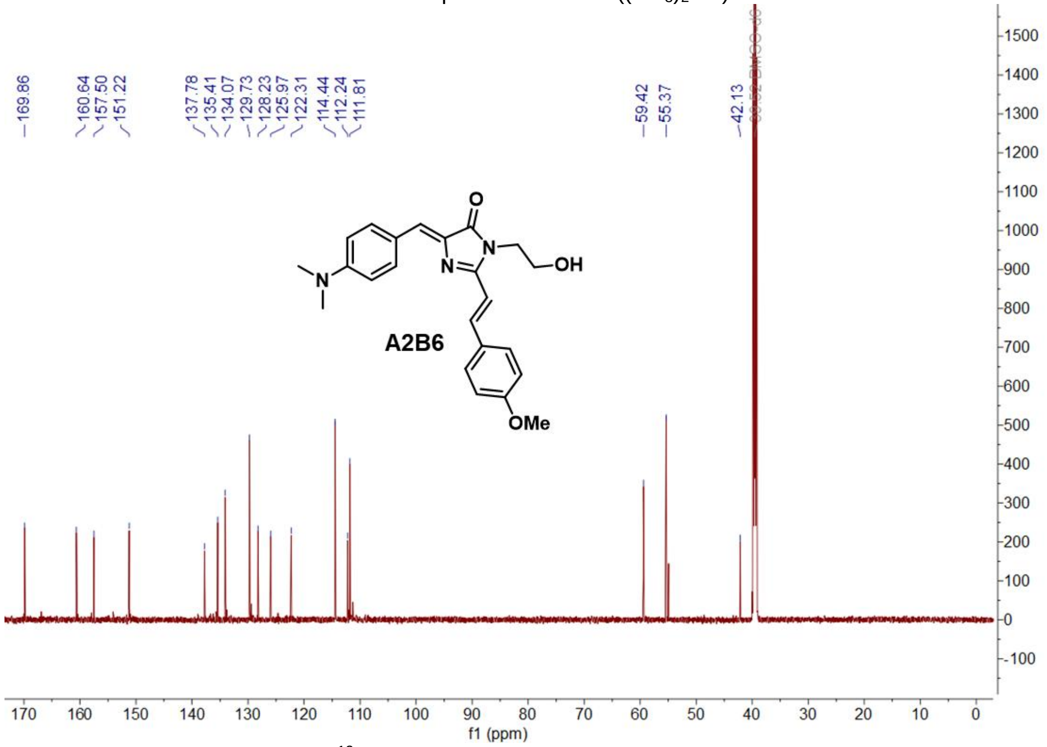

^{13}C NMR spectrum of A2B6 ($(CD_3)_2SO$)

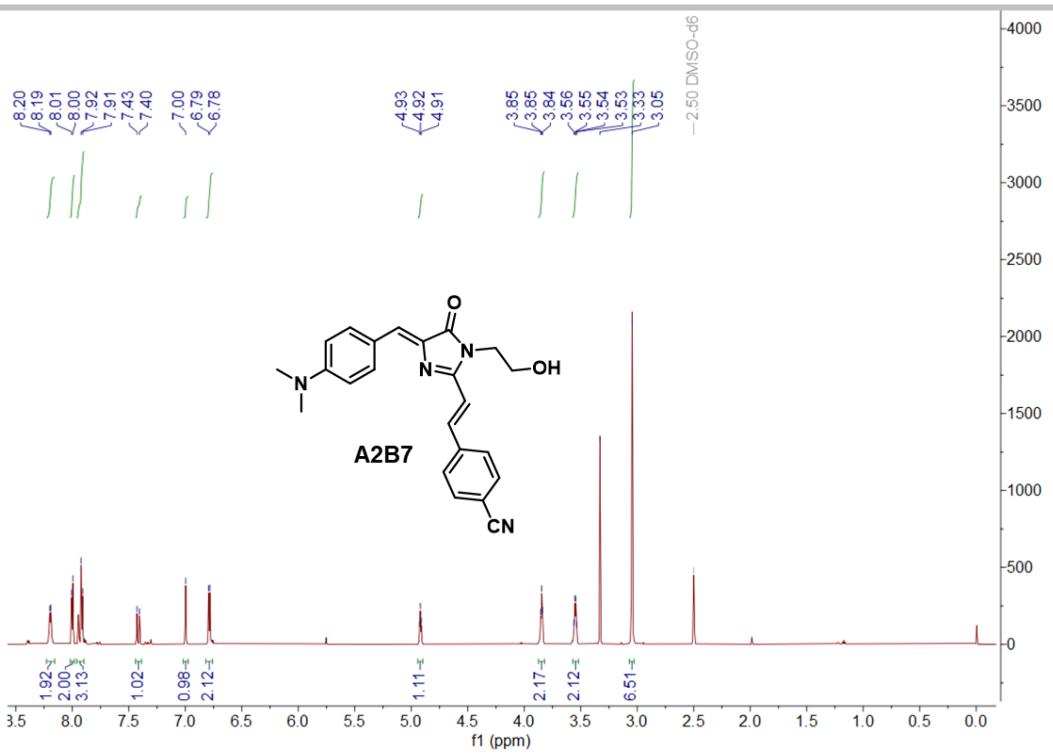

^1H NMR spectrum of A2B7 ($(CD_3)_2SO$)

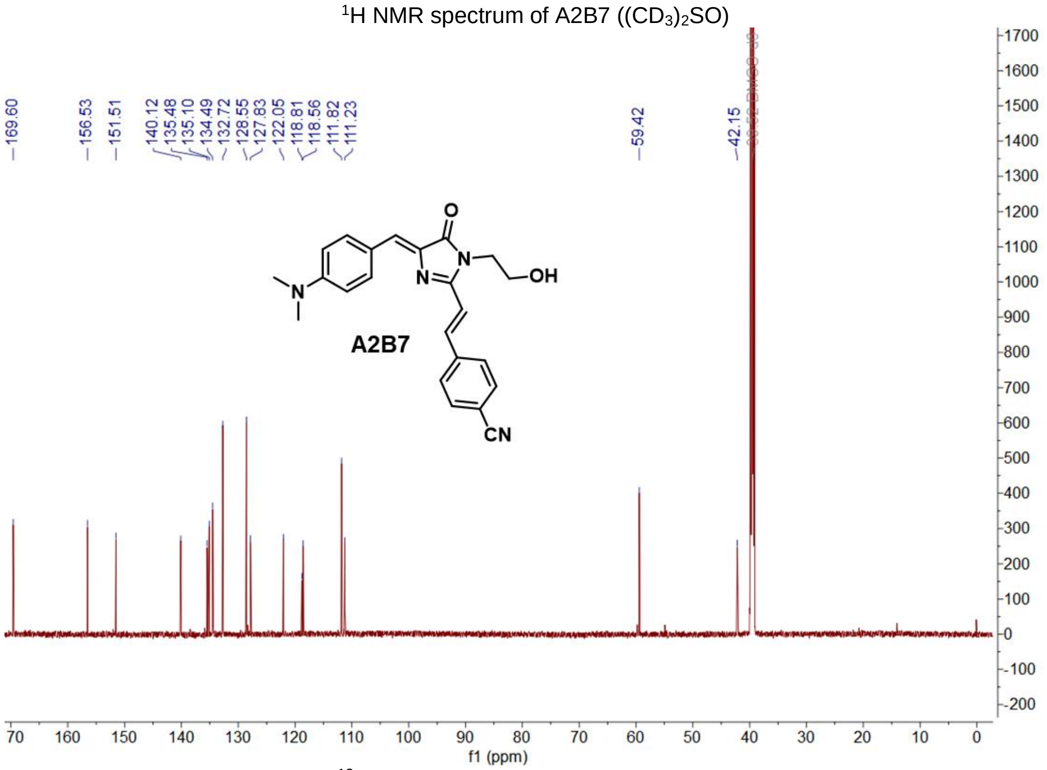

^{13}C NMR spectrum of A2B7 ($(CD_3)_2SO$)

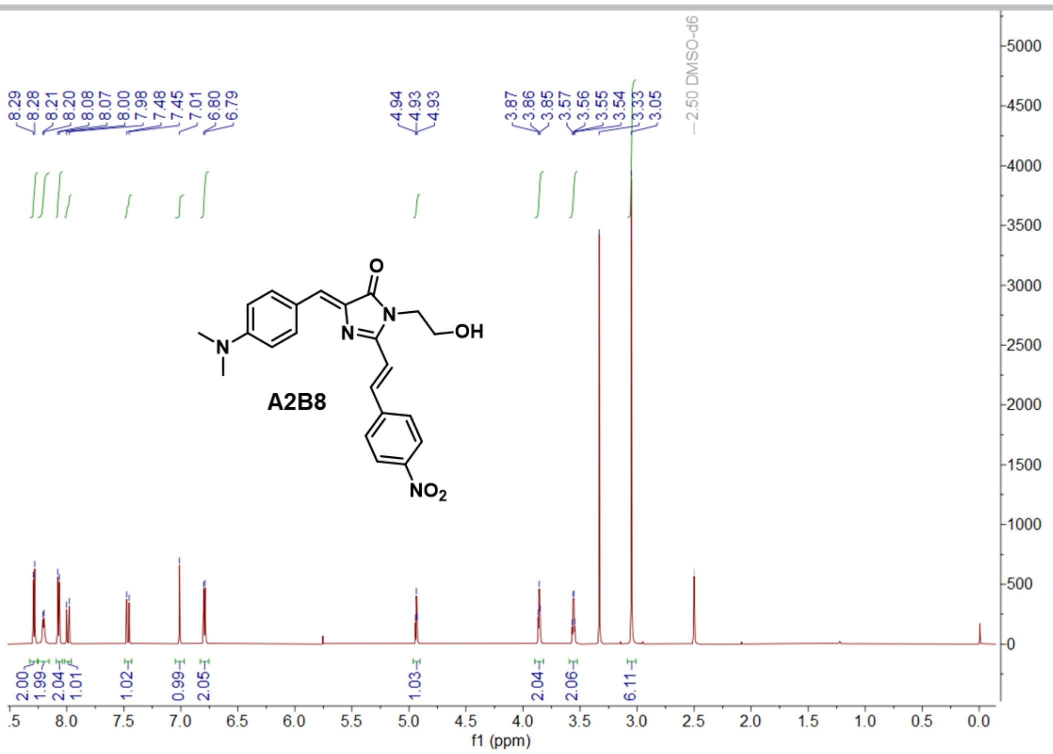

^{1}H NMR spectrum of A2B8 ($(CD_3)_2SO$)

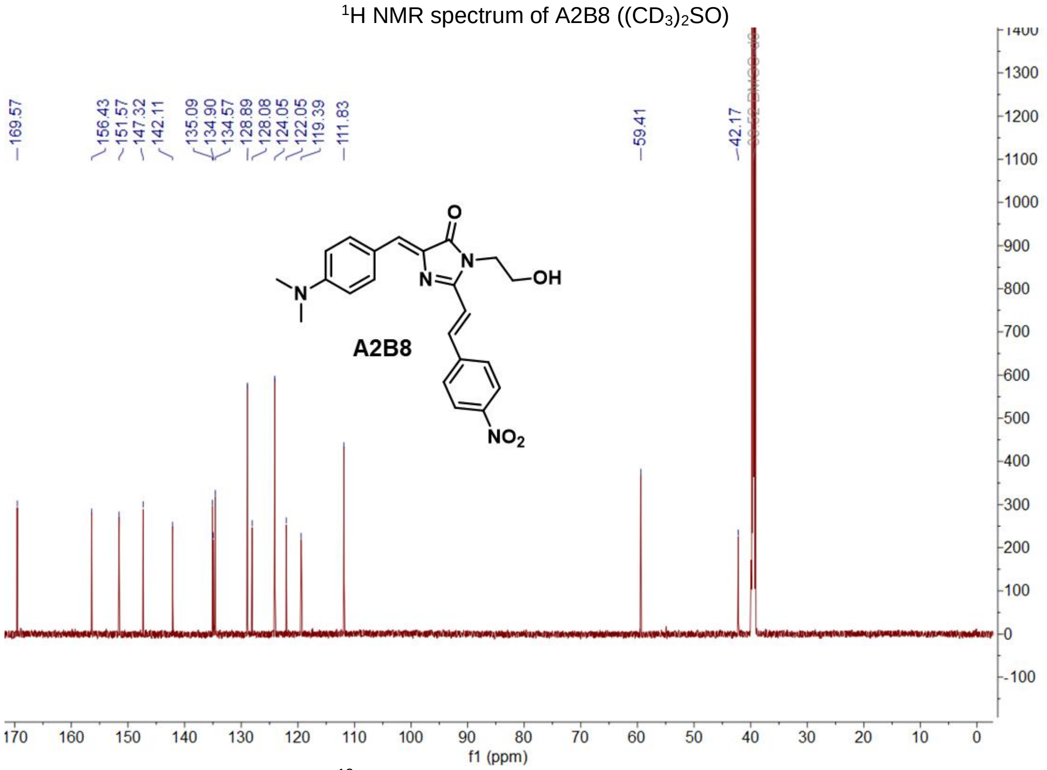

^{13}C NMR spectrum of A2B8 ($(CD_3)_2SO$)

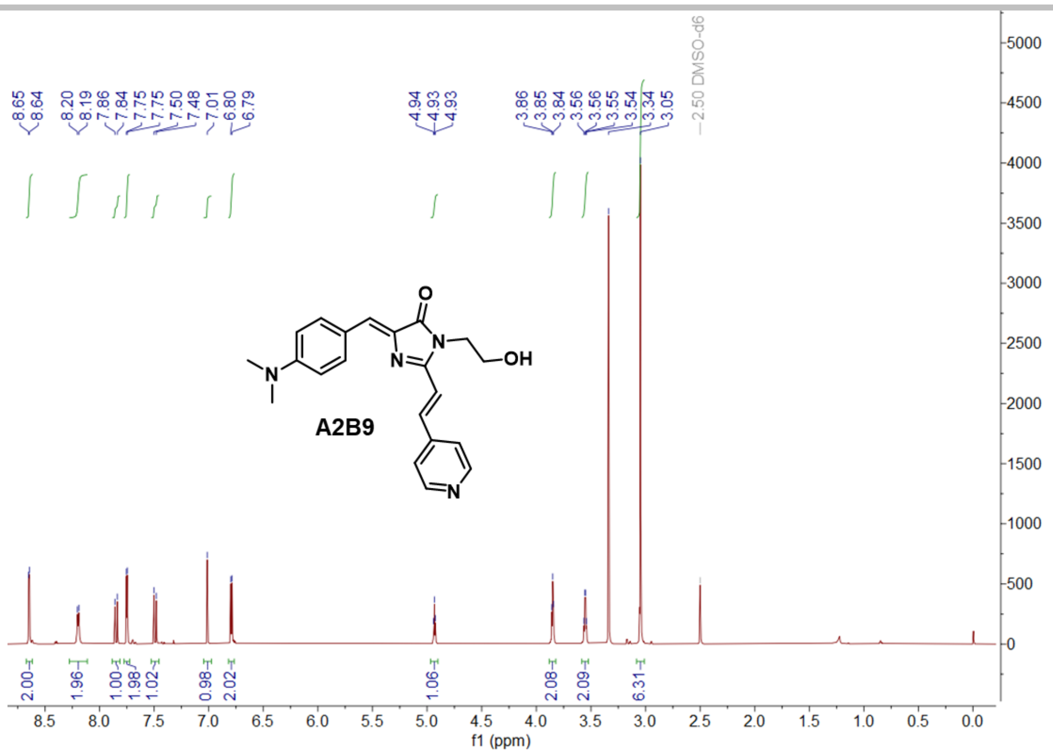

^{1}H NMR spectrum of A2B9 ($(CD_3)_2SO$)

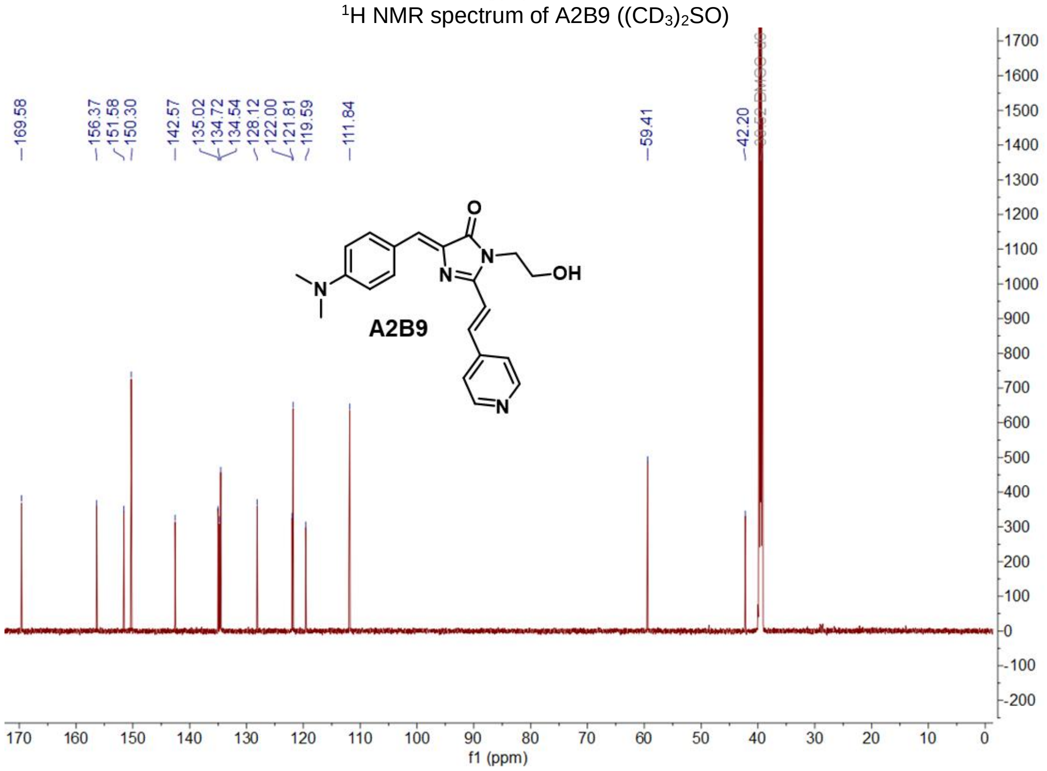

^{13}C NMR spectrum of A2B9 ($(CD_3)_2SO$)

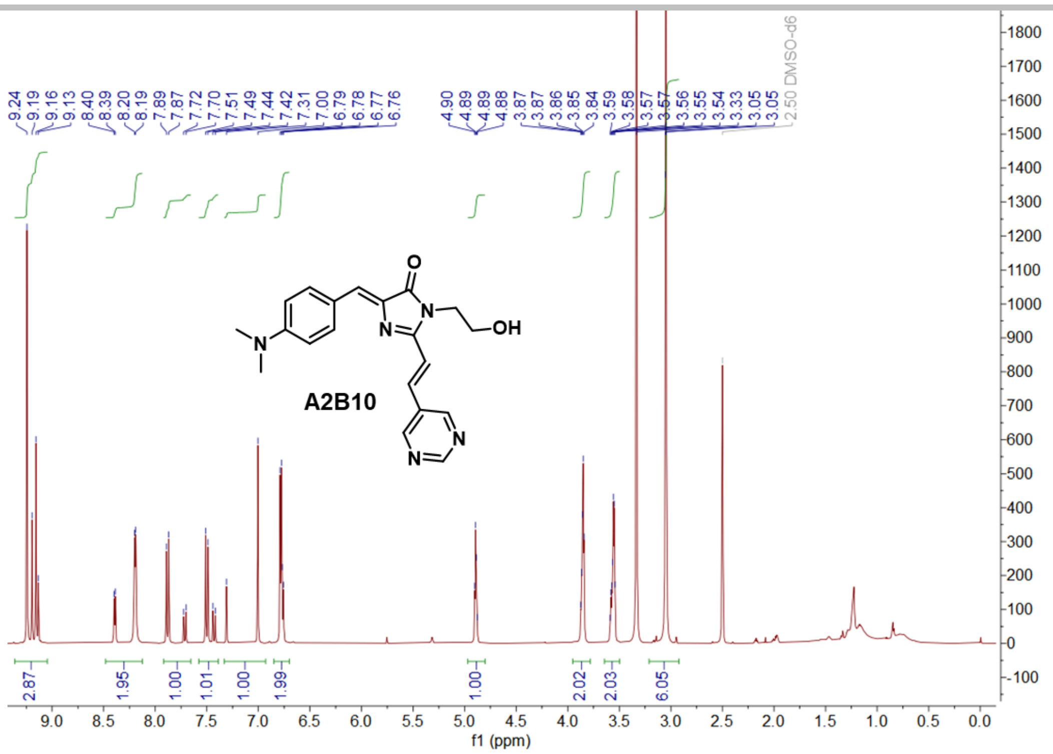

^{1}H NMR spectrum of A2B10 ($(CD_3)_2SO$)

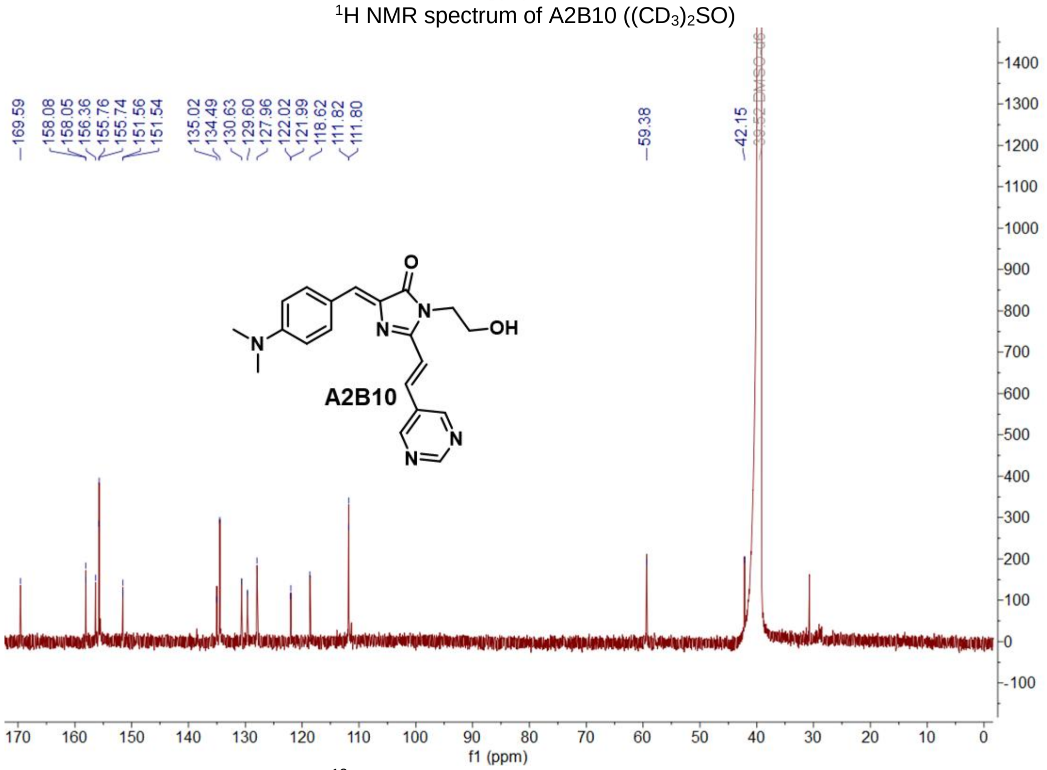

^{13}C NMR spectrum of A2B10 ($(CD_3)_2SO$)

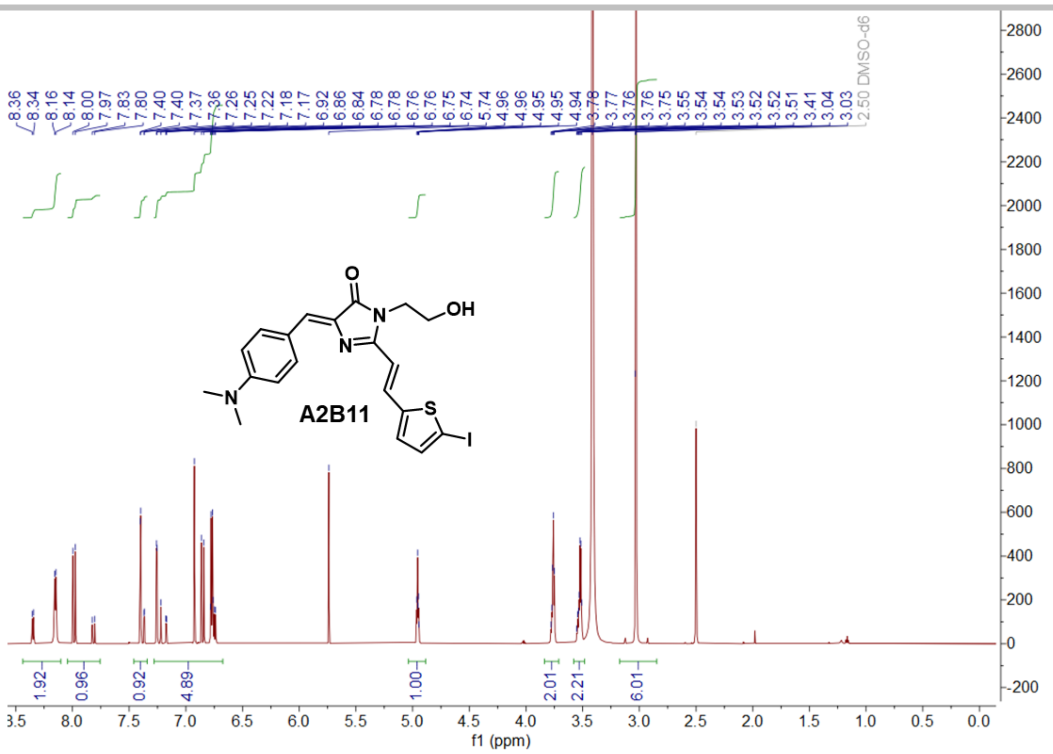

^{1}H NMR spectrum of A2B11 ($(CD_3)_2SO$)

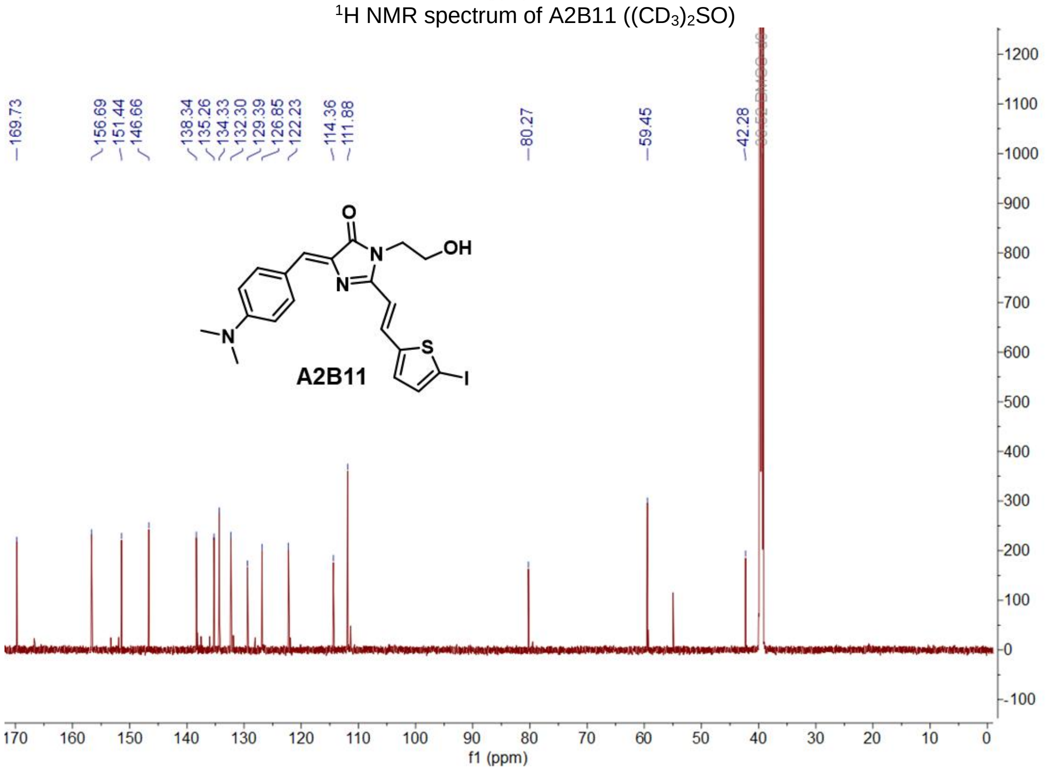

^{13}C NMR spectrum of A2B11 ($(CD_3)_2SO$)

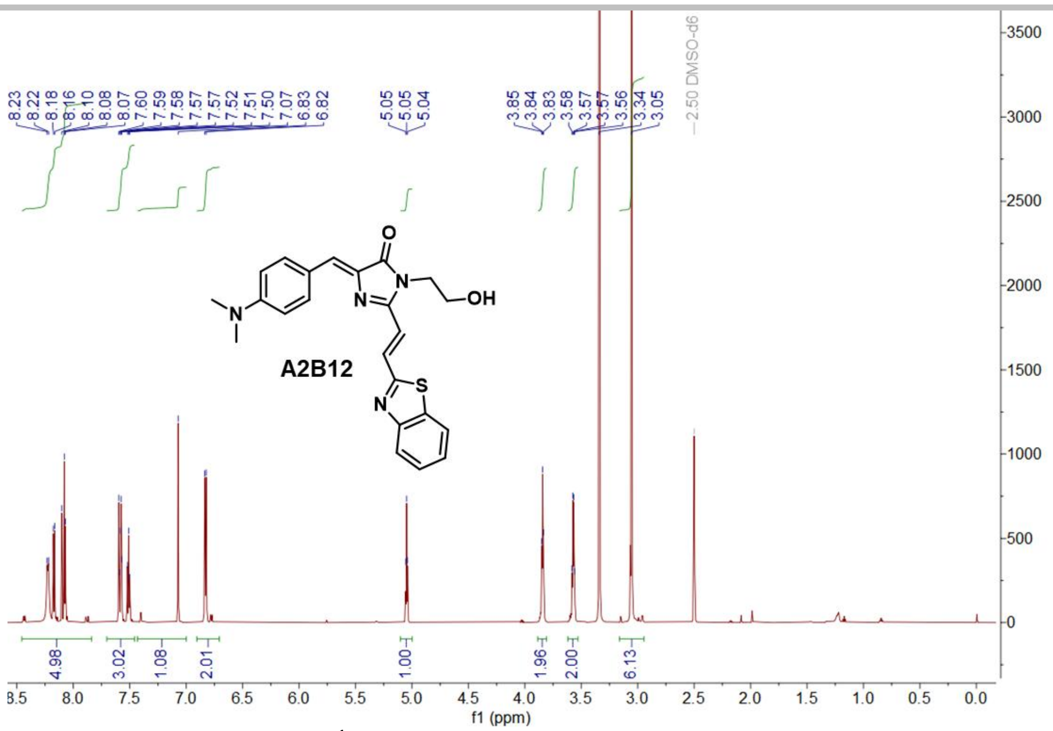

^{1}H NMR spectrum of A2B12 ($(CD_3)_2SO$)

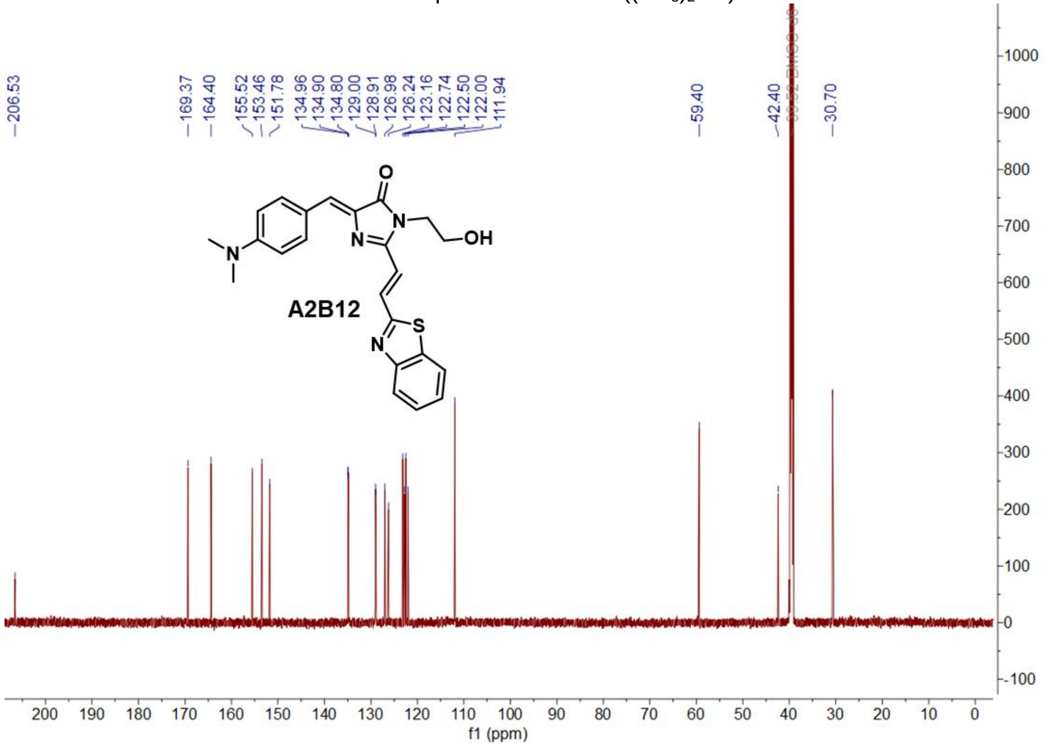

^{13}C NMR spectrum of A2B12 ($(CD_3)_2SO$)

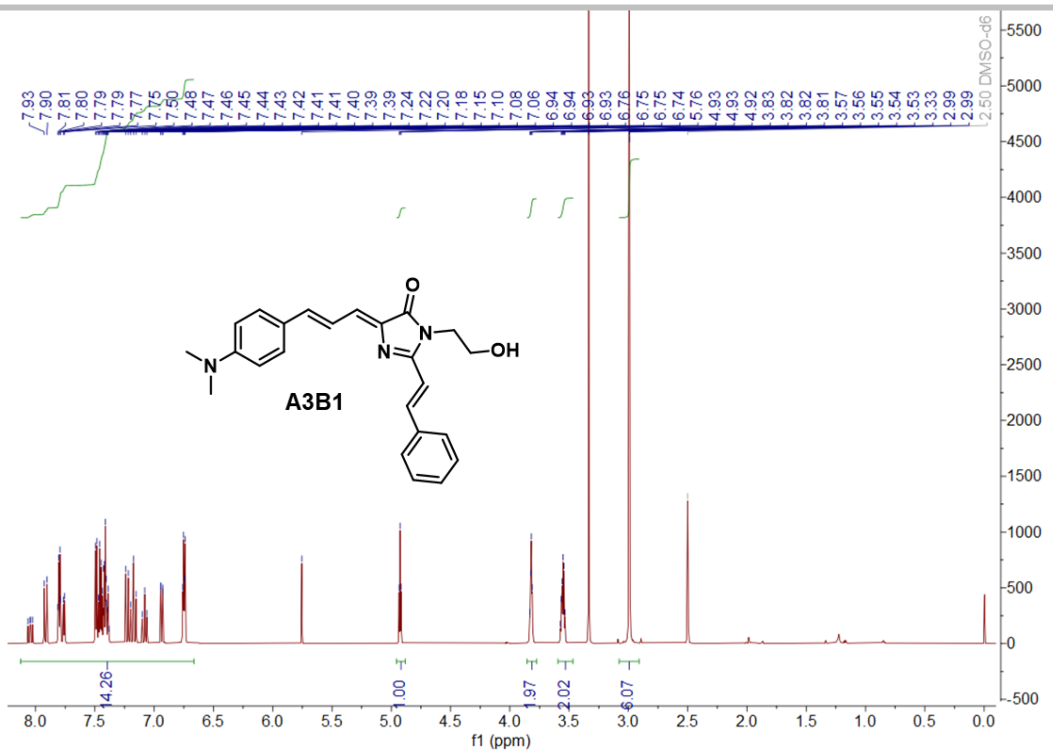

^{1}H NMR spectrum of A3B1 ($(CD_3)_2SO$)

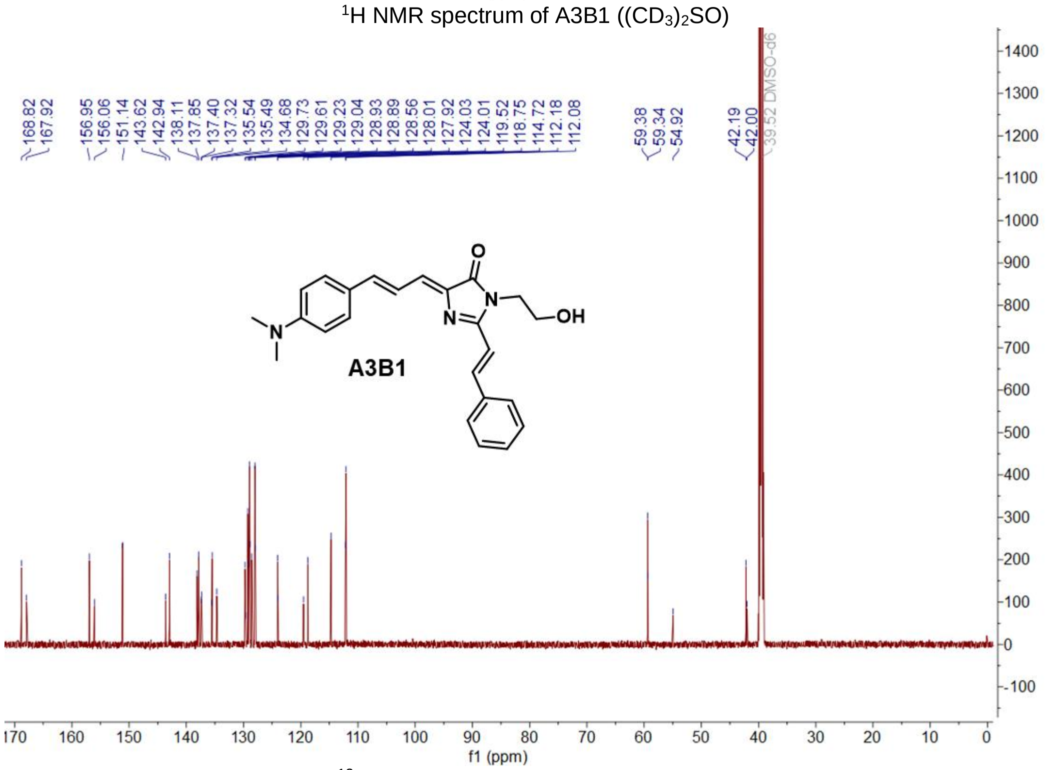

^{13}C NMR spectrum of A3B1 ($(CD_3)_2SO$)

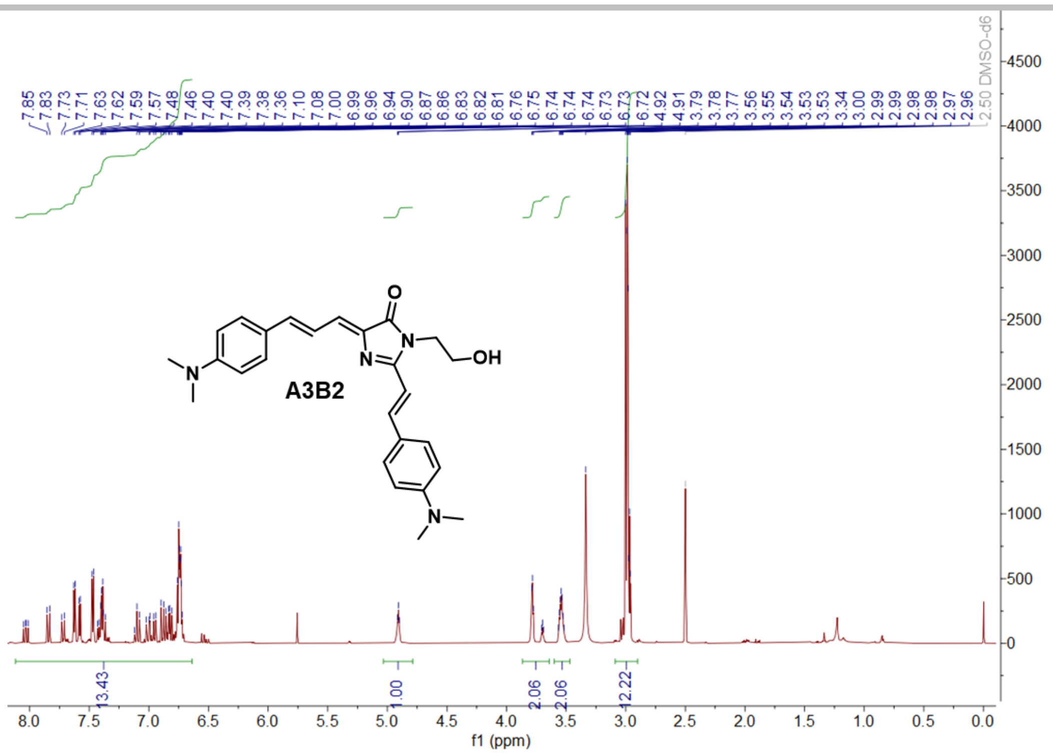

^{1}H NMR spectrum of A3B2 ($(CD_3)_2SO$)

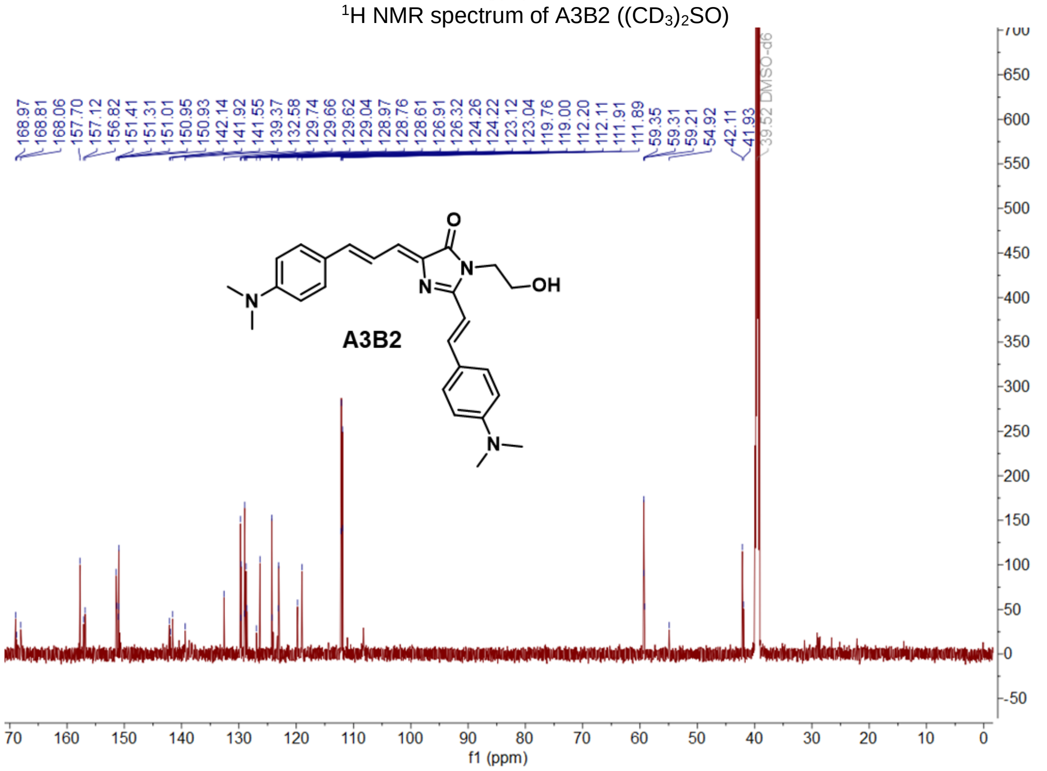

^{13}C NMR spectrum of A3B2 ($(CD_3)_2SO$)

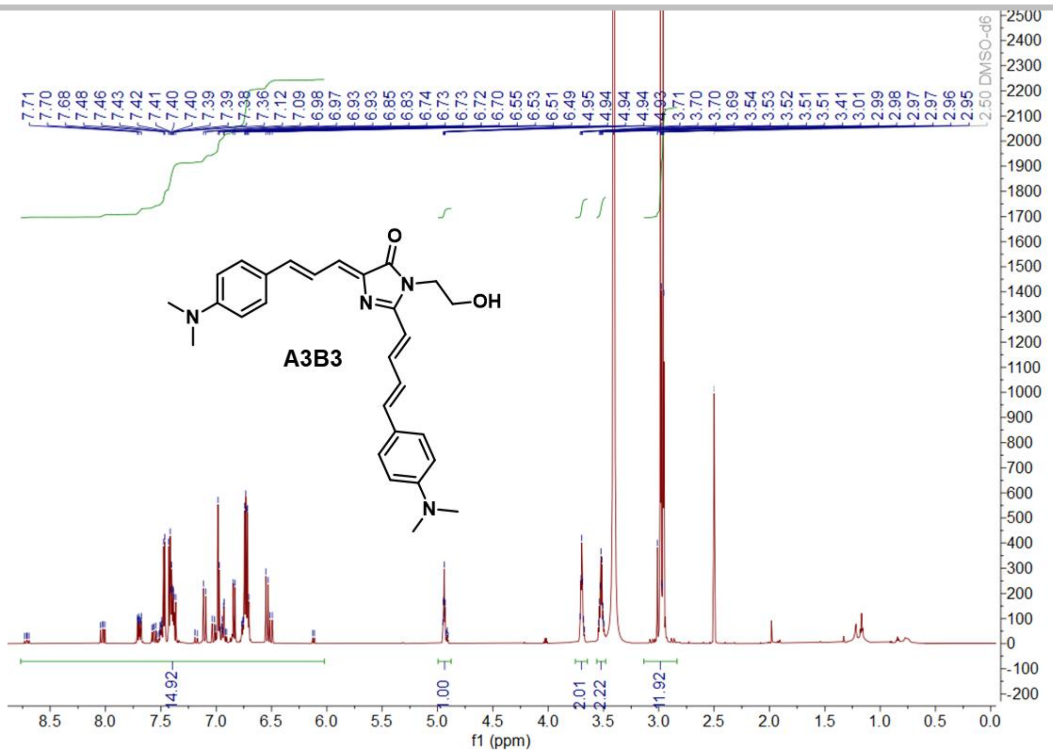

^{1}H NMR spectrum of A3B3 ($(CD_3)_2SO$)

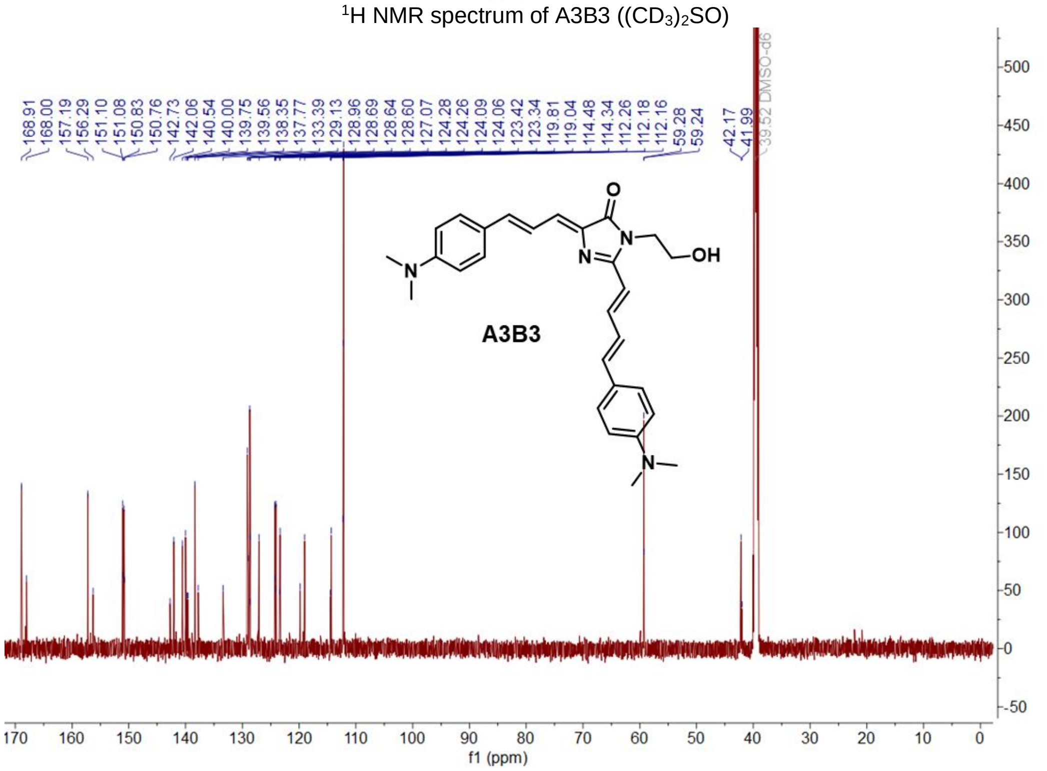

^{13}C NMR spectrum of A3B3 ($(CD_3)_2SO$)

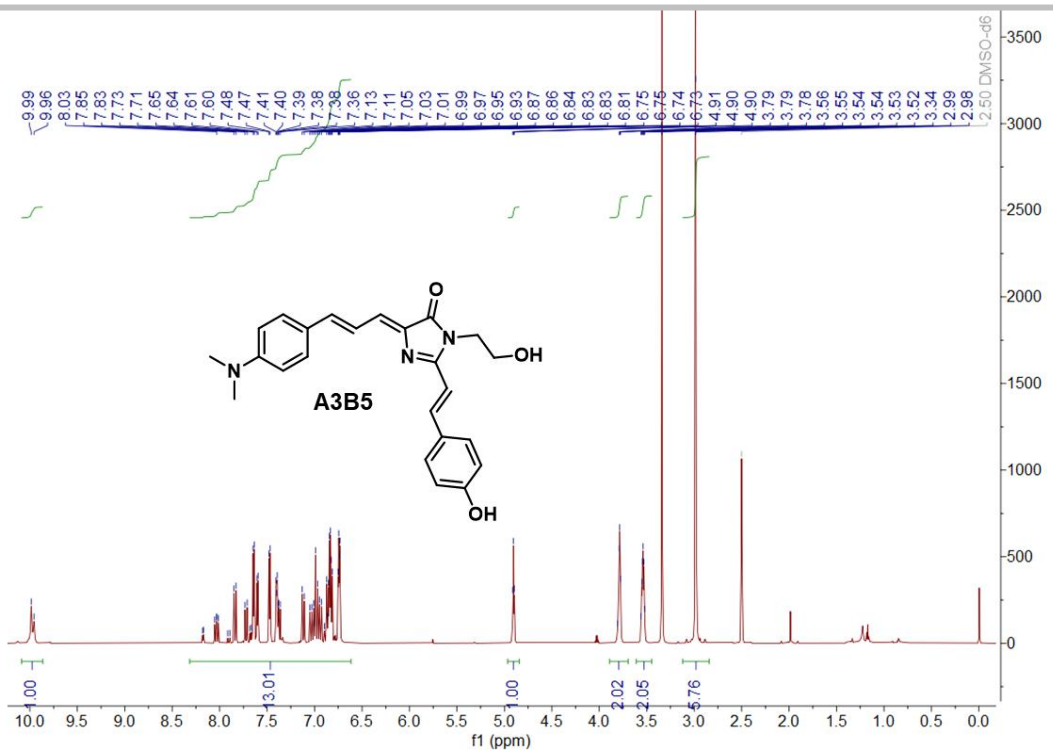

^1H NMR spectrum of A3B5 ($(CD_3)_2SO$)

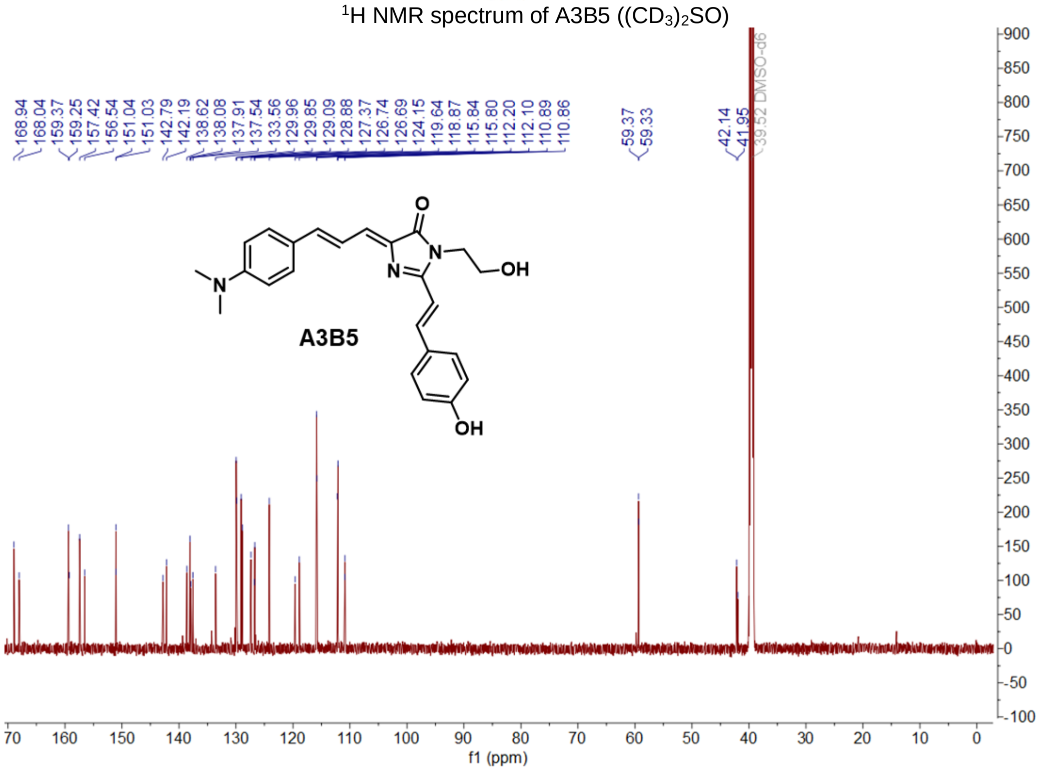

^{13}C NMR spectrum of A3B5 ($(CD_3)_2SO$)

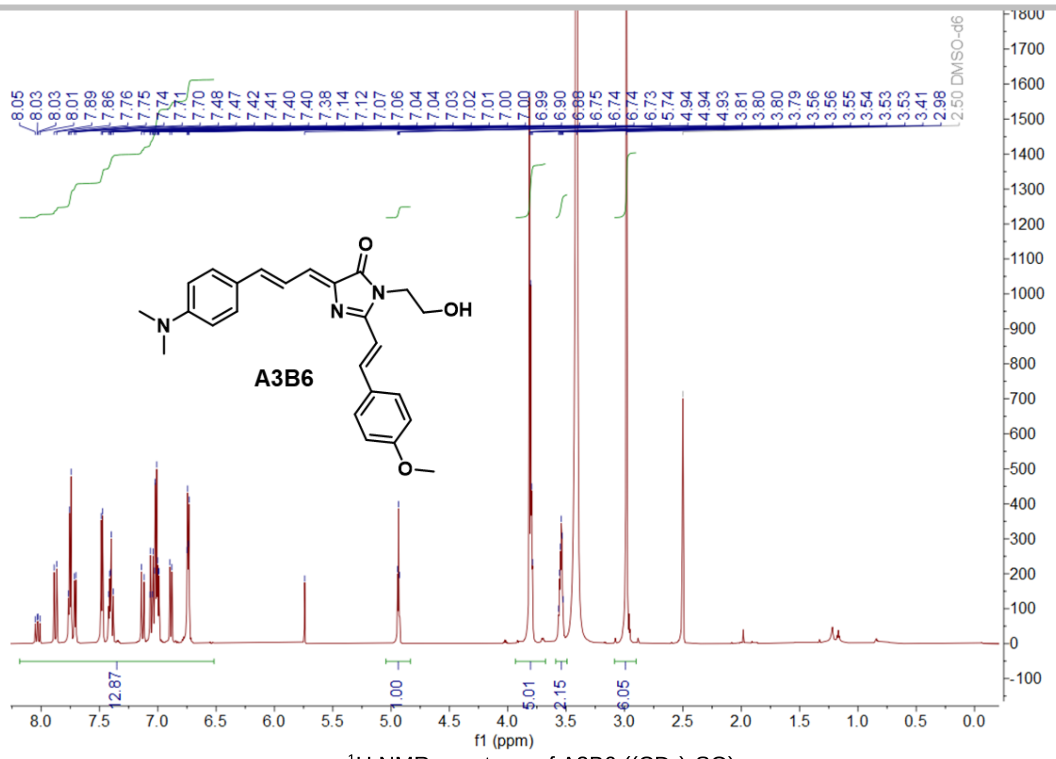

^{1}H NMR spectrum of A3B6 ($(CD_3)_2SO$)

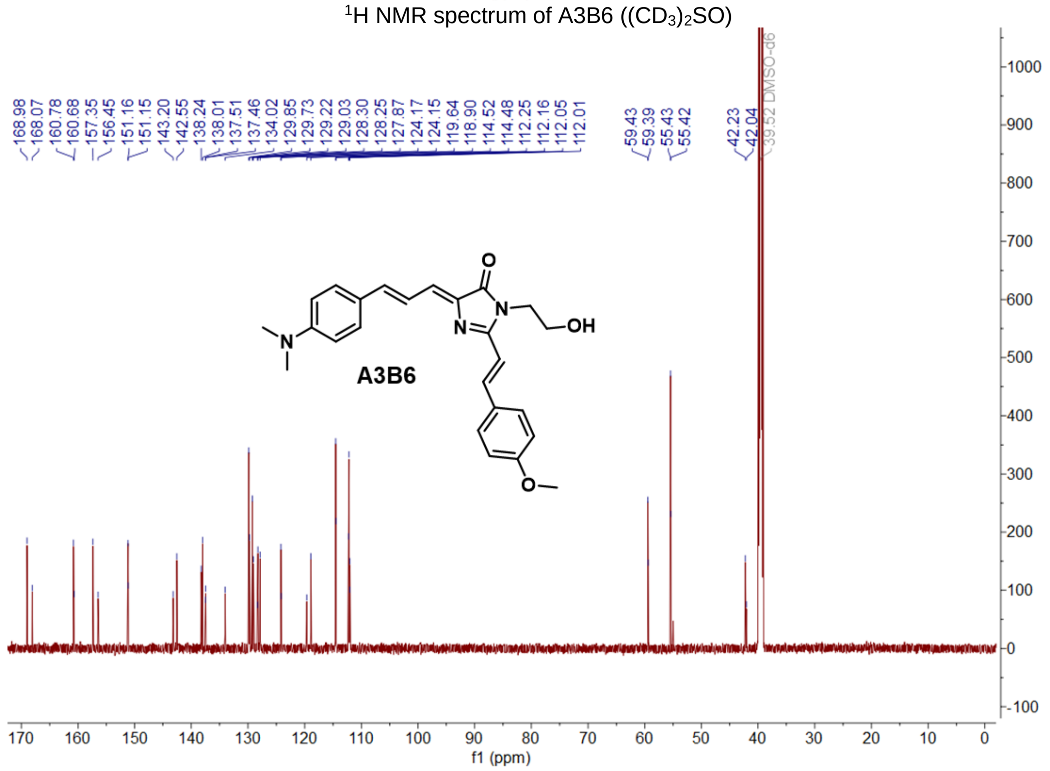

^{13}C NMR spectrum of A3B6 ($(CD_3)_2SO$)

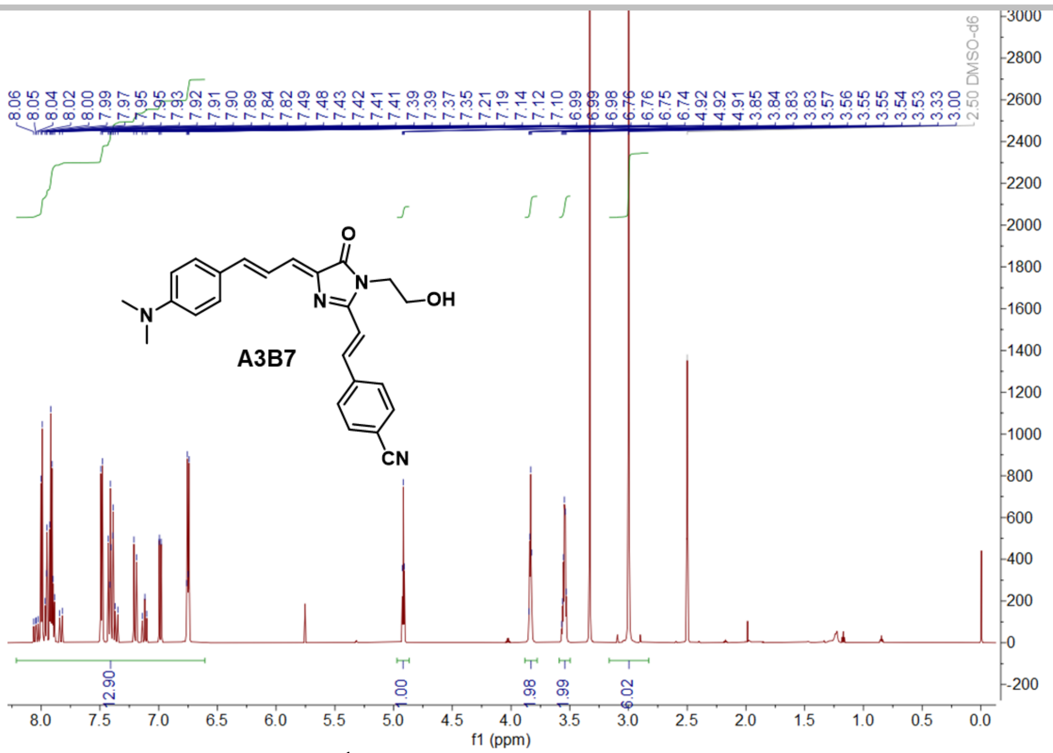

^{1}H NMR spectrum of A3B7 ($(CD_3)_2SO$)

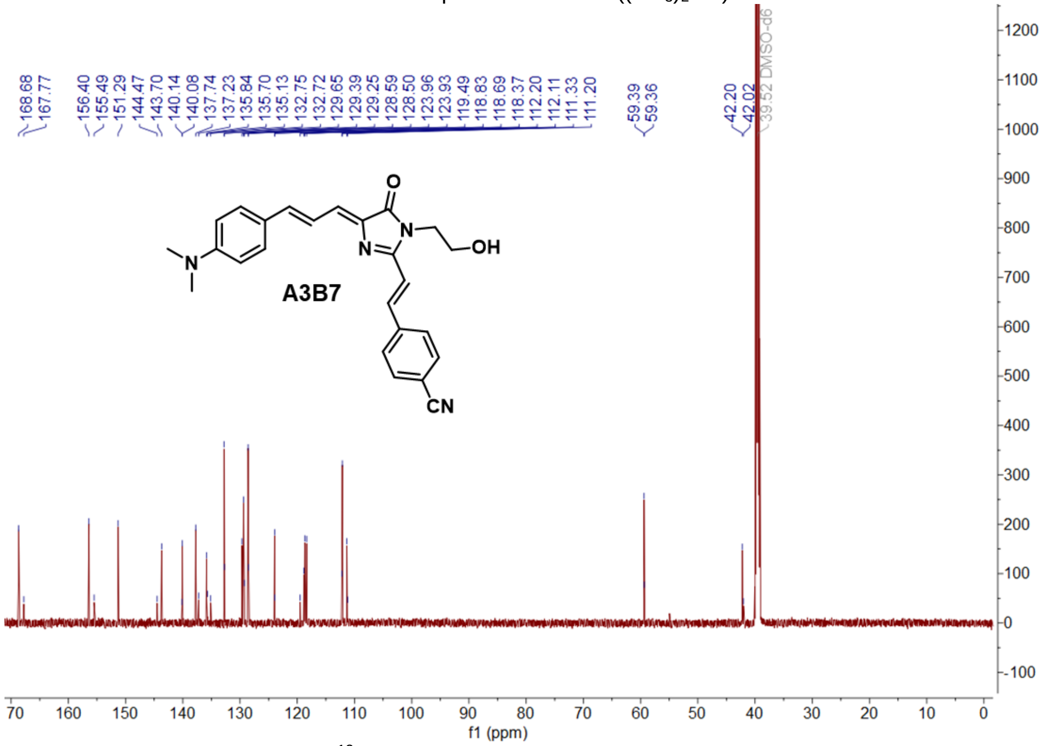

^{13}C NMR spectrum of A3B7 ($(CD_3)_2SO$)

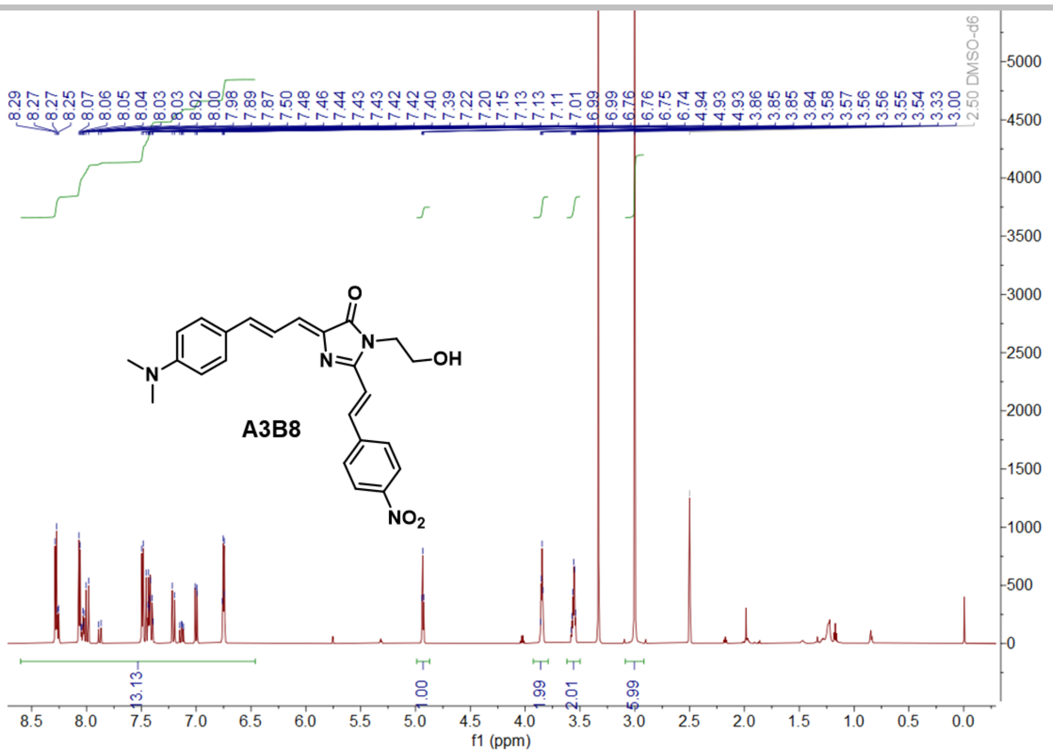

^{1}H NMR spectrum of A3B8 ($(CD_3)_2SO$)

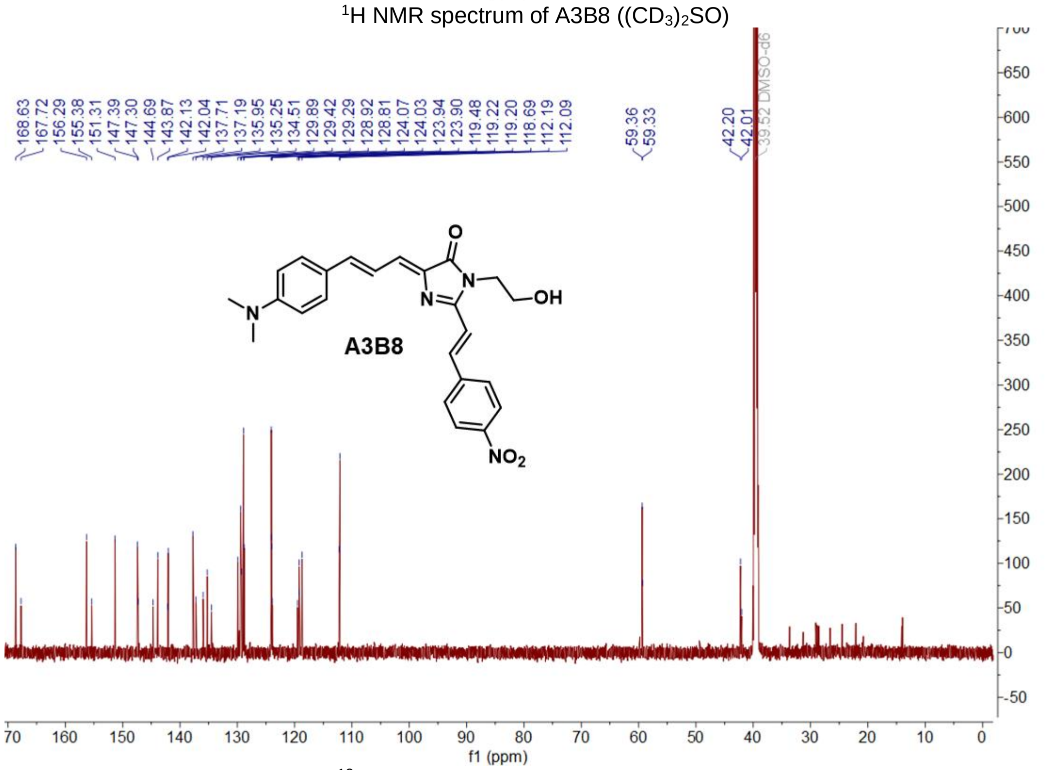

^{13}C NMR spectrum of A3B8 ($(CD_3)_2SO$)

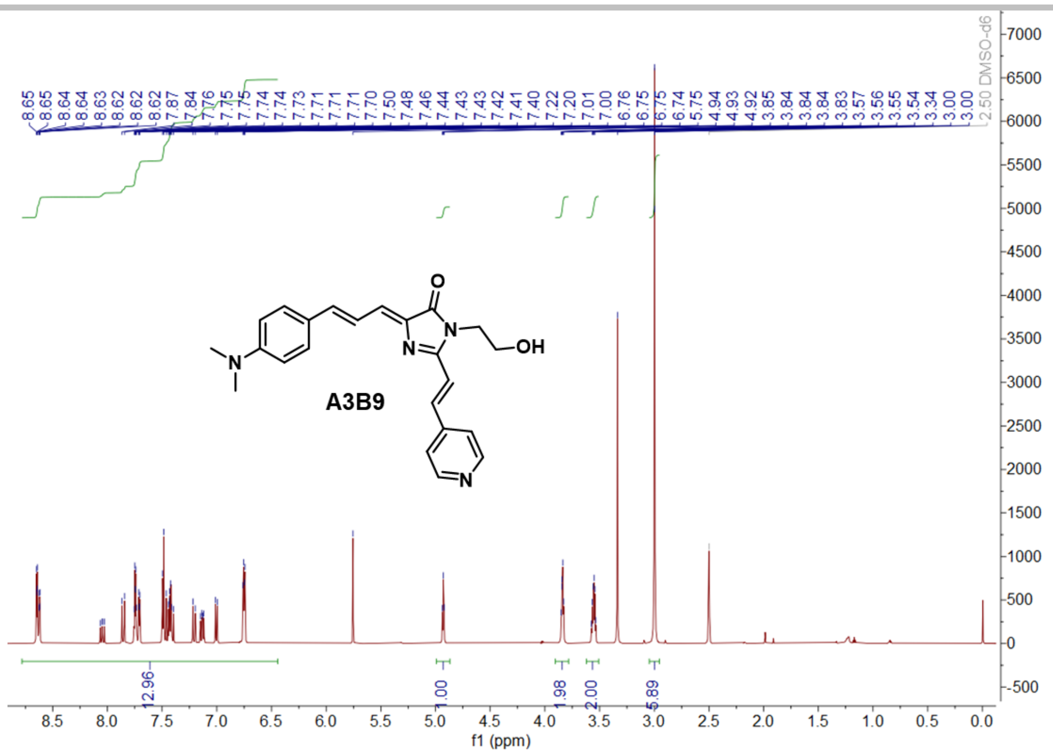

^{1}H NMR spectrum of A3B9 ($(CD_3)_2SO$)

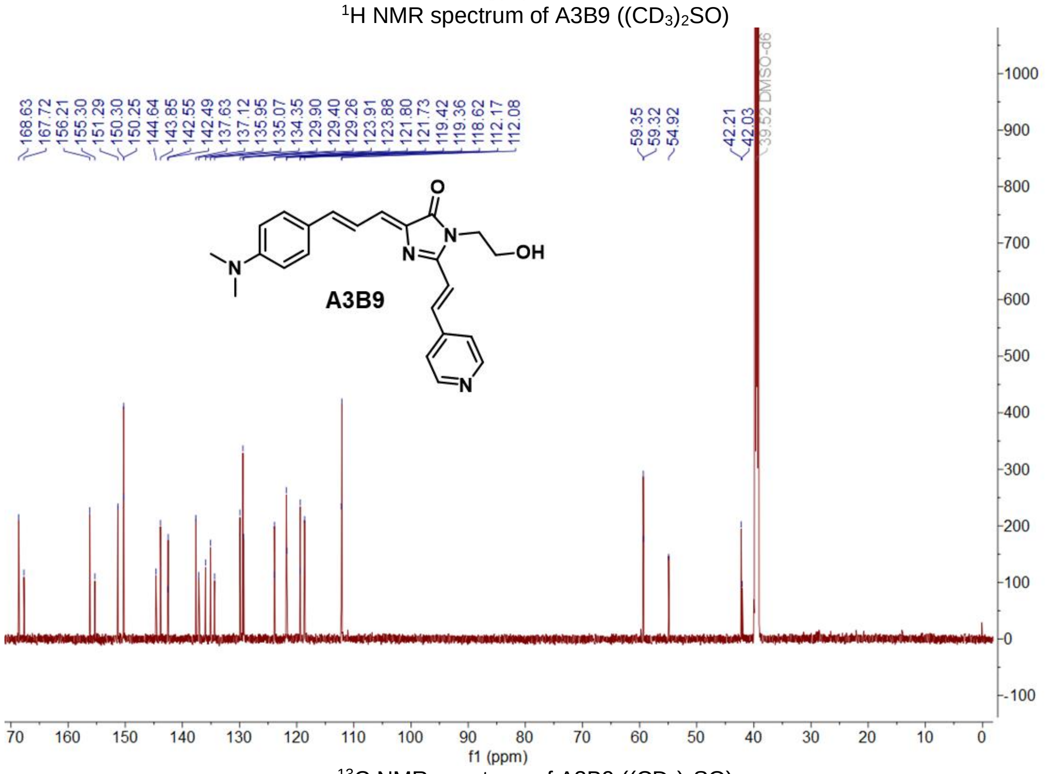

^{13}C NMR spectrum of A3B9 ($(CD_3)_2SO$)

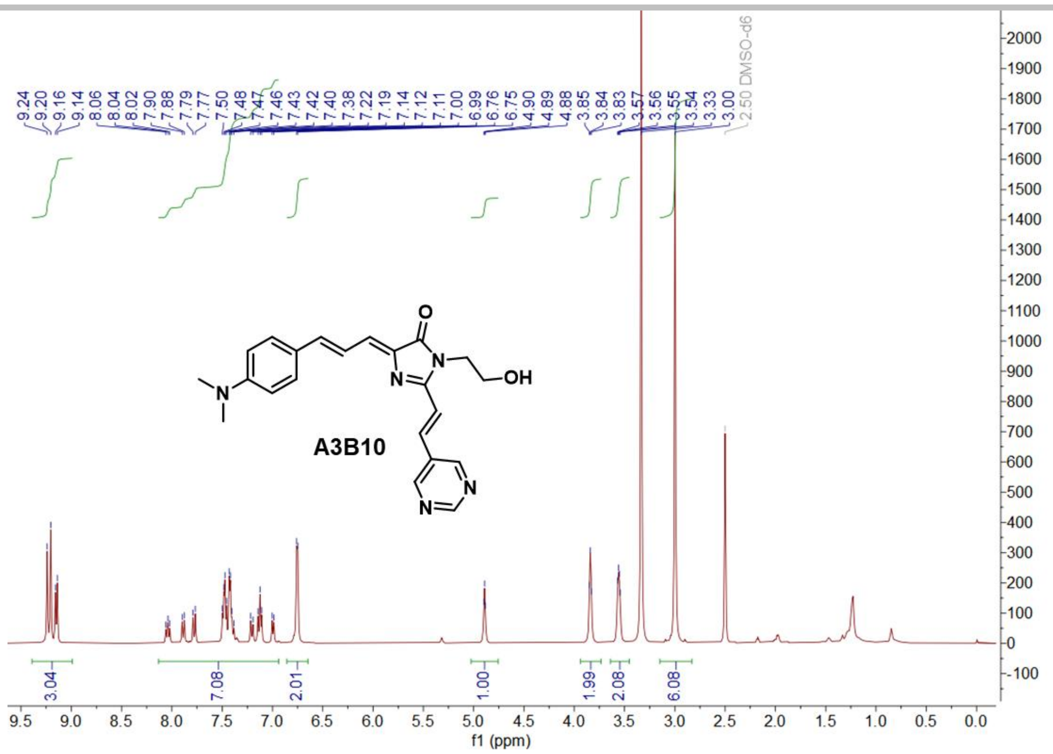

^{1}H NMR spectrum of A3B10 ($(CD_3)_2SO$)

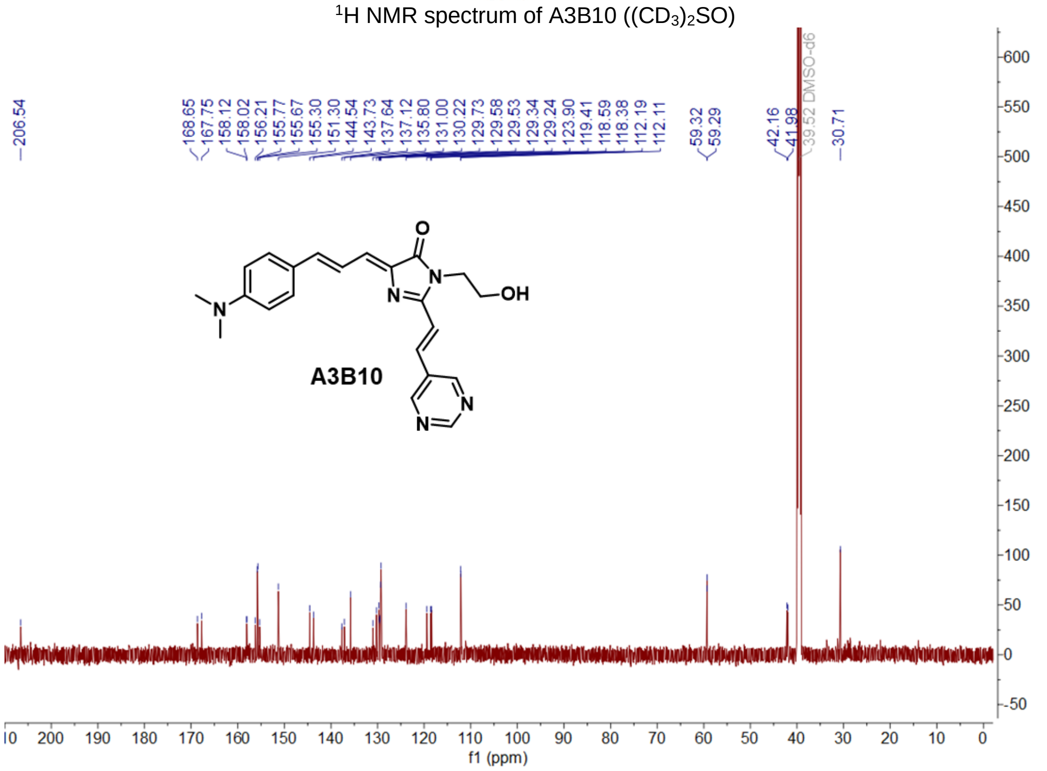

^{13}C NMR spectrum of A3B10 ($(CD_3)_2SO$)

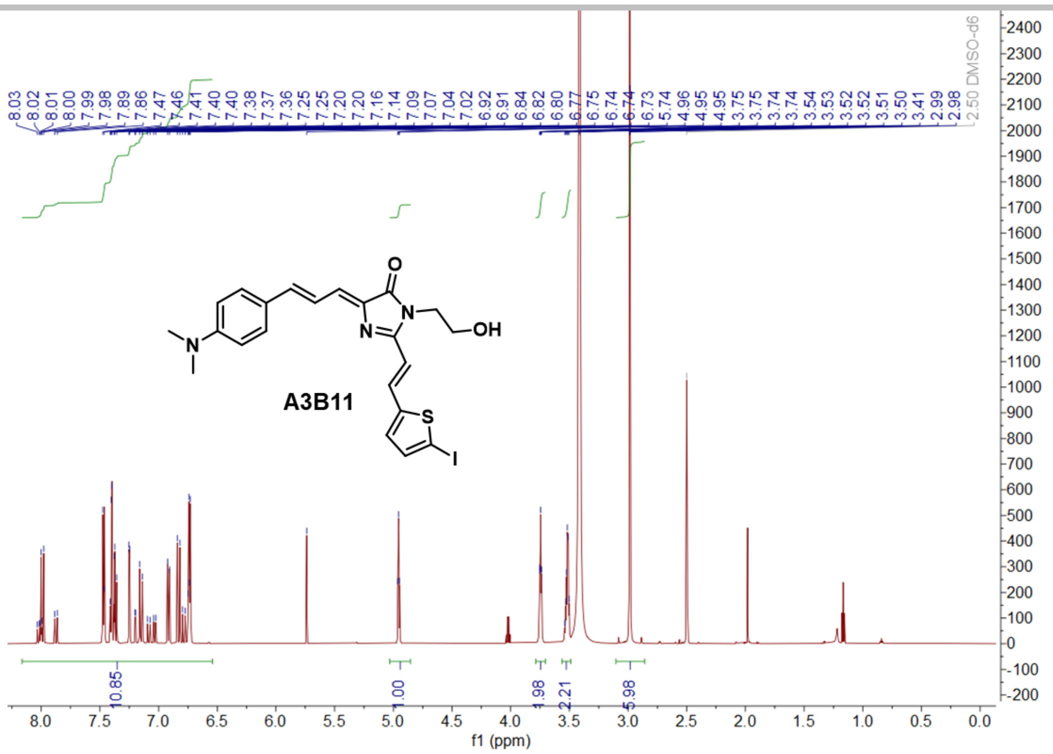

^{1}H NMR spectrum of A3B11 ((CD_3)$_2$SO)

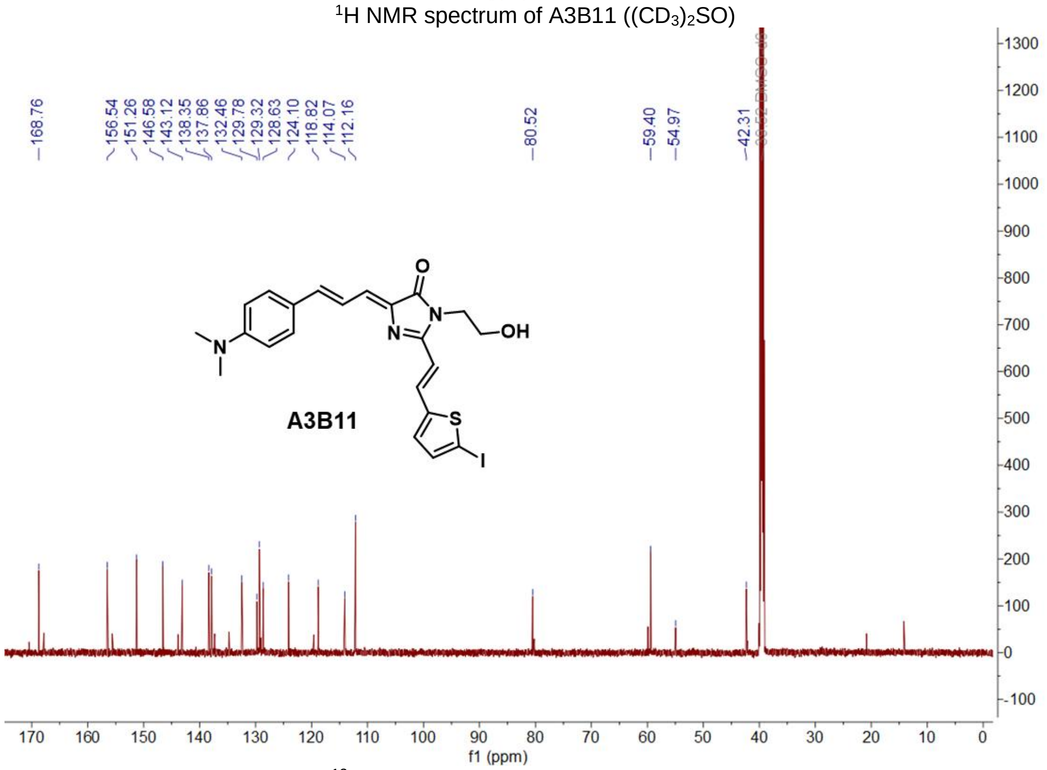

^{13}C NMR spectrum of A3B11 ((CD_3)$_2$SO)

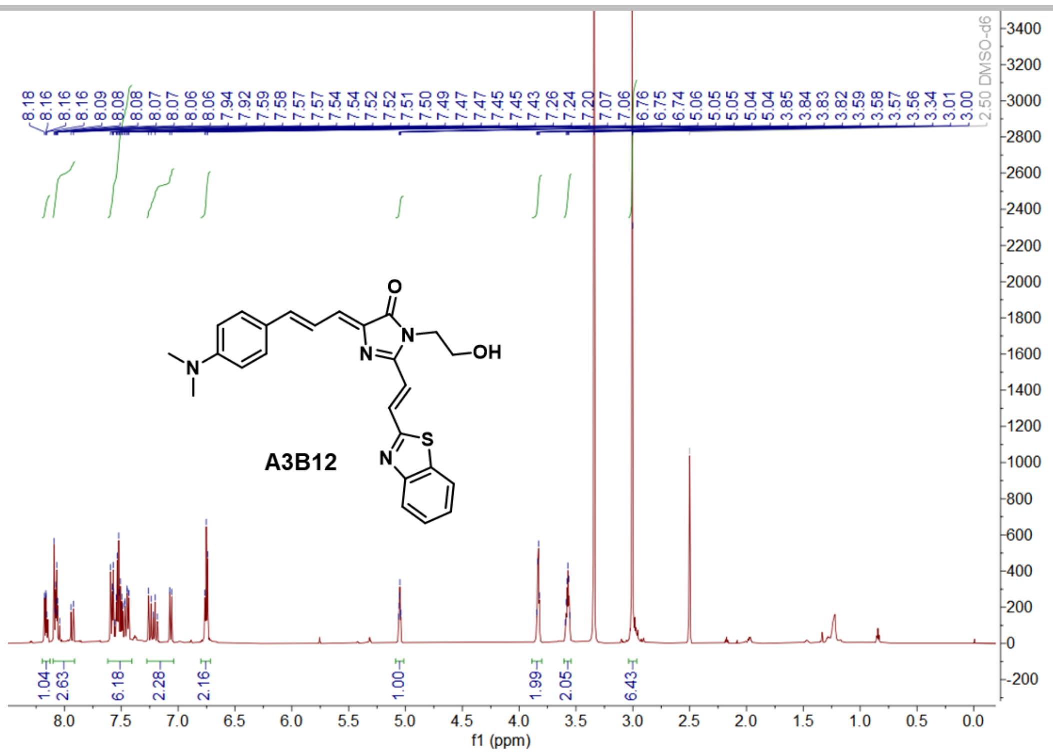

^{1}H NMR spectrum of A3B12 ($(CD_3)_2SO$)

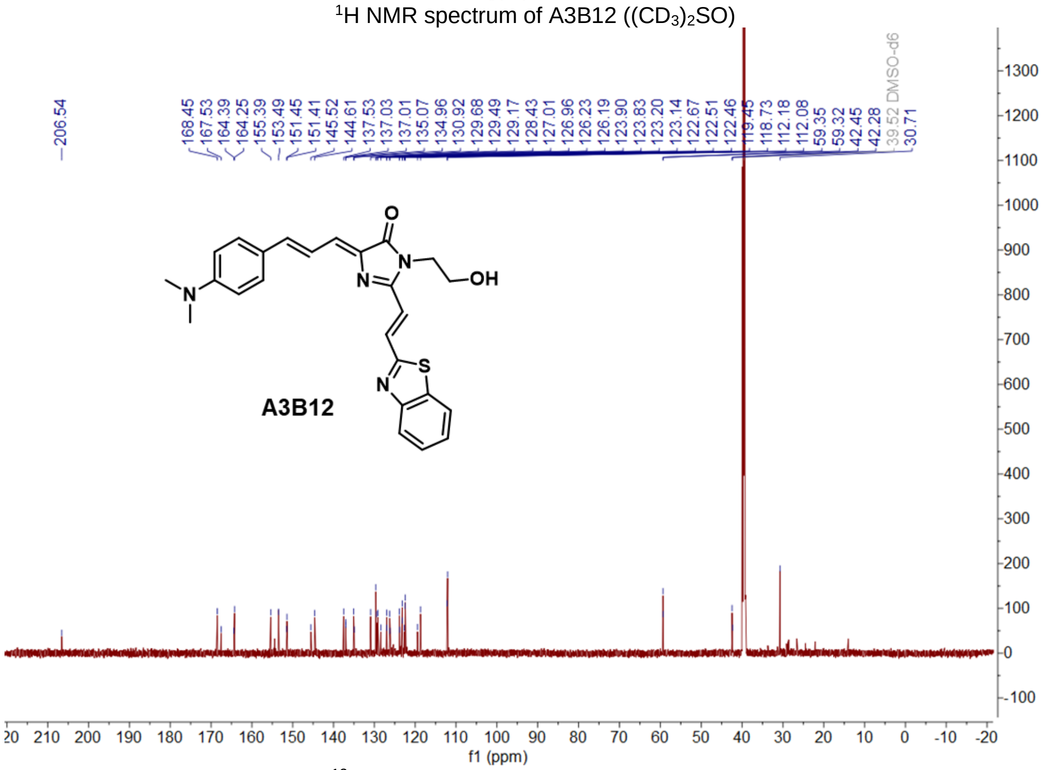

^{13}C NMR spectrum of A3B12 ($(CD_3)_2SO$)

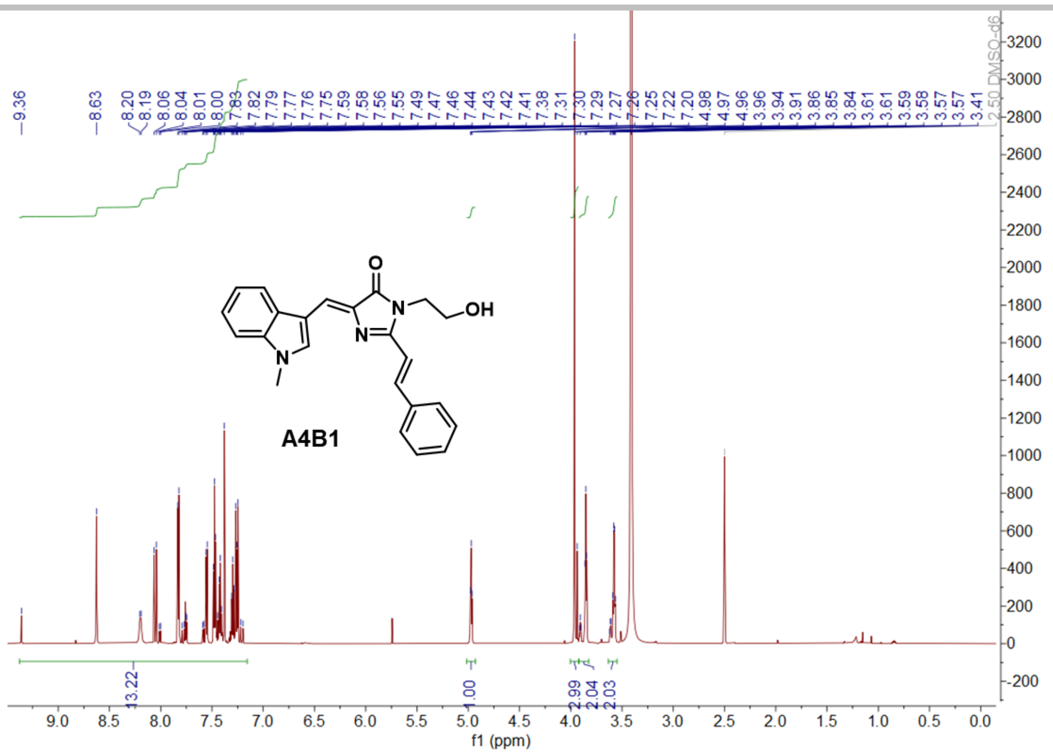

^{1}H NMR spectrum of A4B1 ($(CD_3)_2SO$)

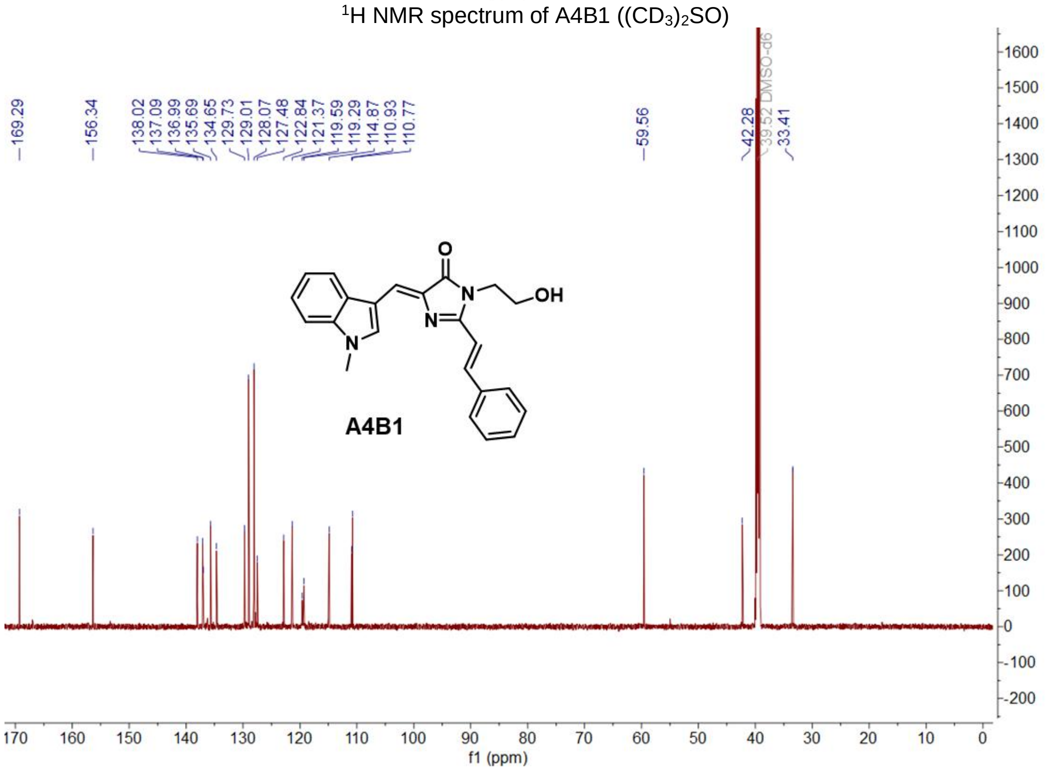

^{13}C NMR spectrum of A4B1 ($(CD_3)_2SO$)

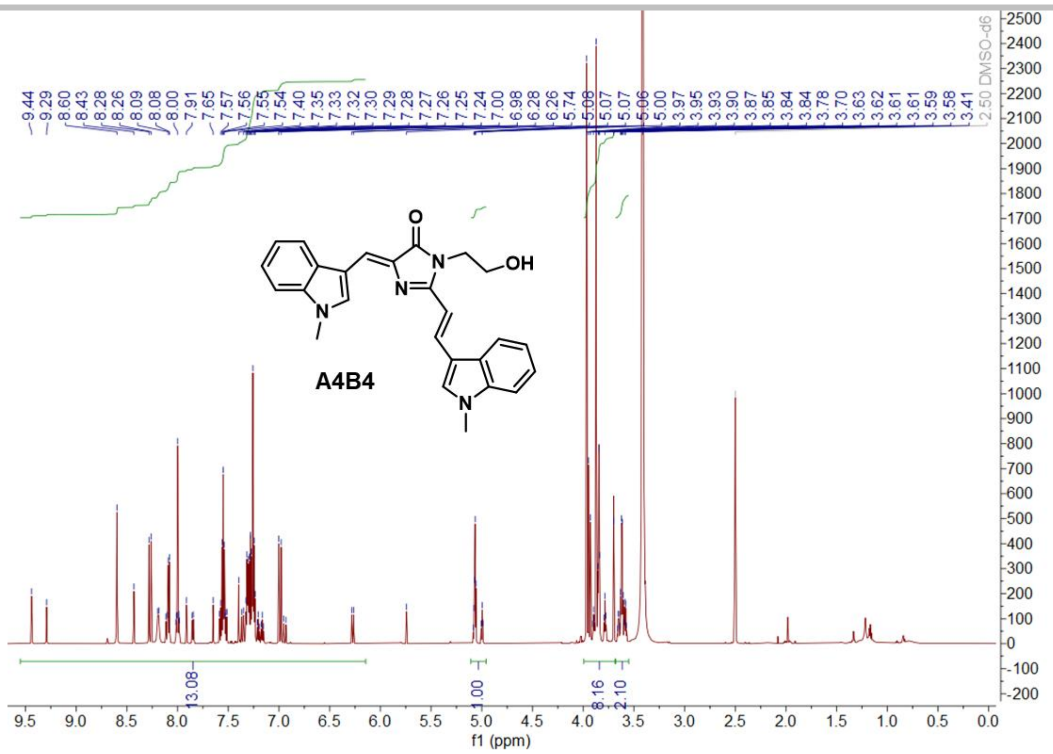

^{1}H NMR spectrum of A4B4 ($(CD_3)_2SO$)

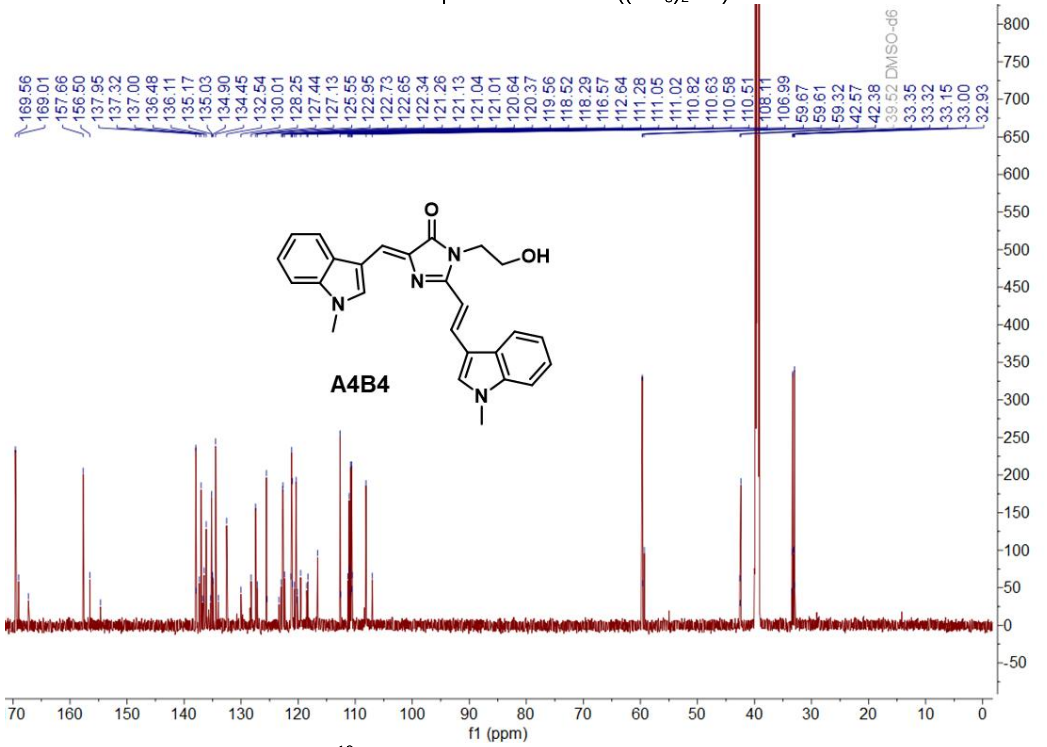

^{13}C NMR spectrum of A4B4 ($(CD_3)_2SO$)

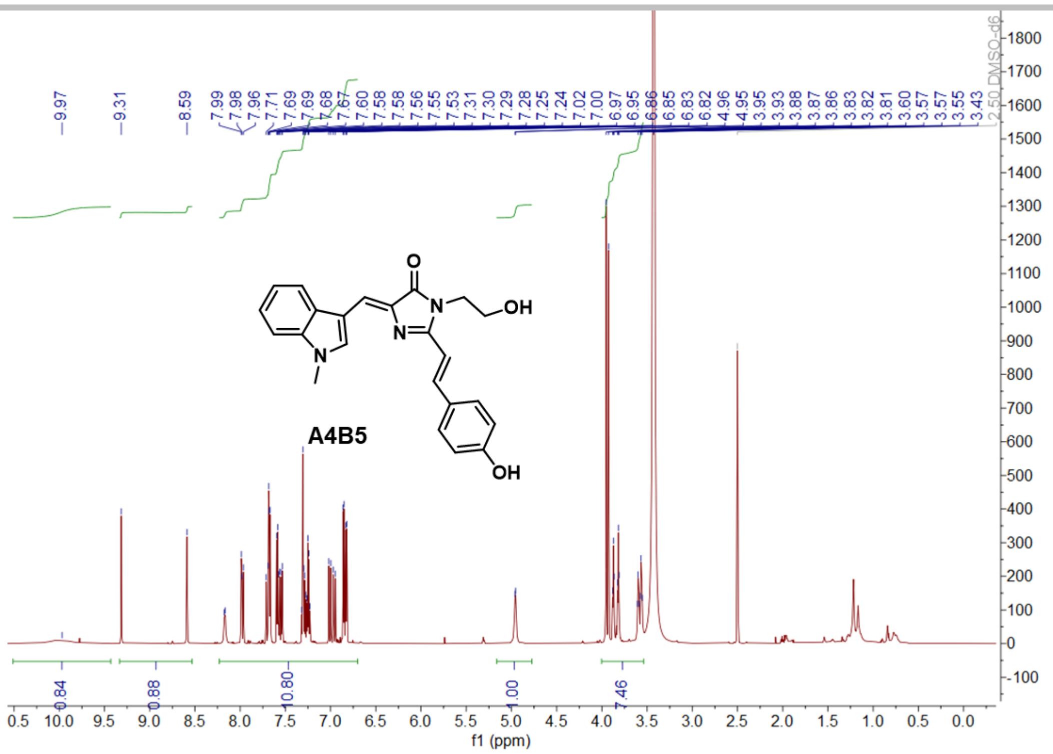

^{1}H NMR spectrum of A4B5 ($(CD_3)_2SO$)

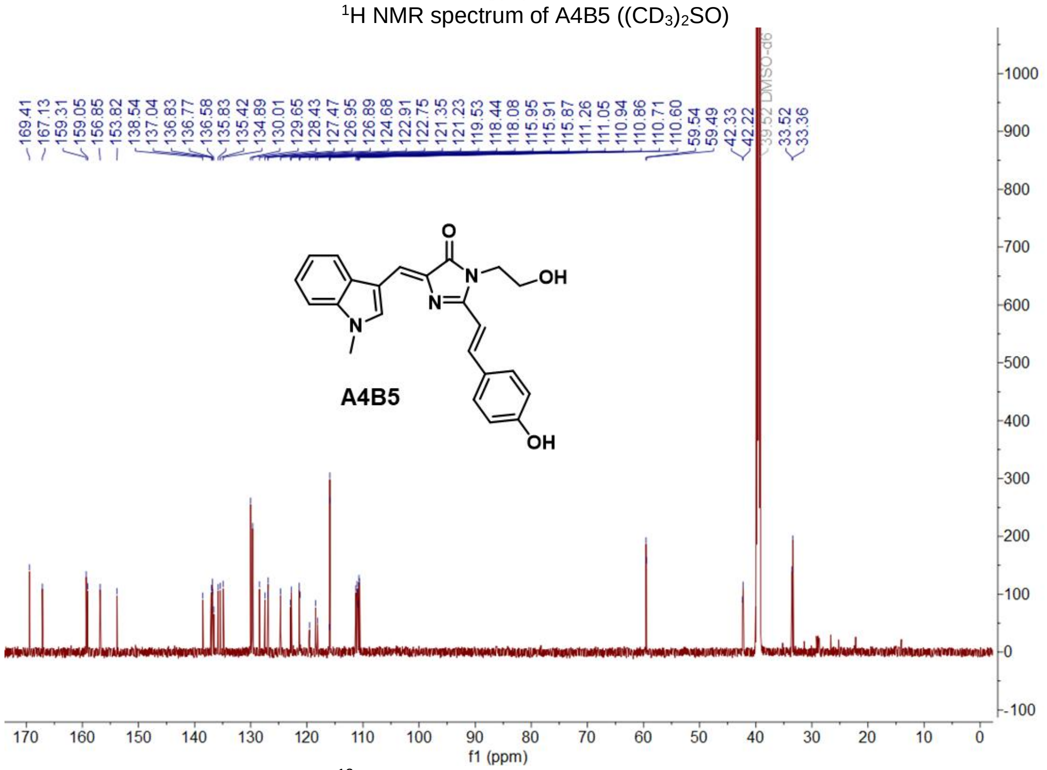

^{13}C NMR spectrum of A4B5 ($(CD_3)_2SO$)

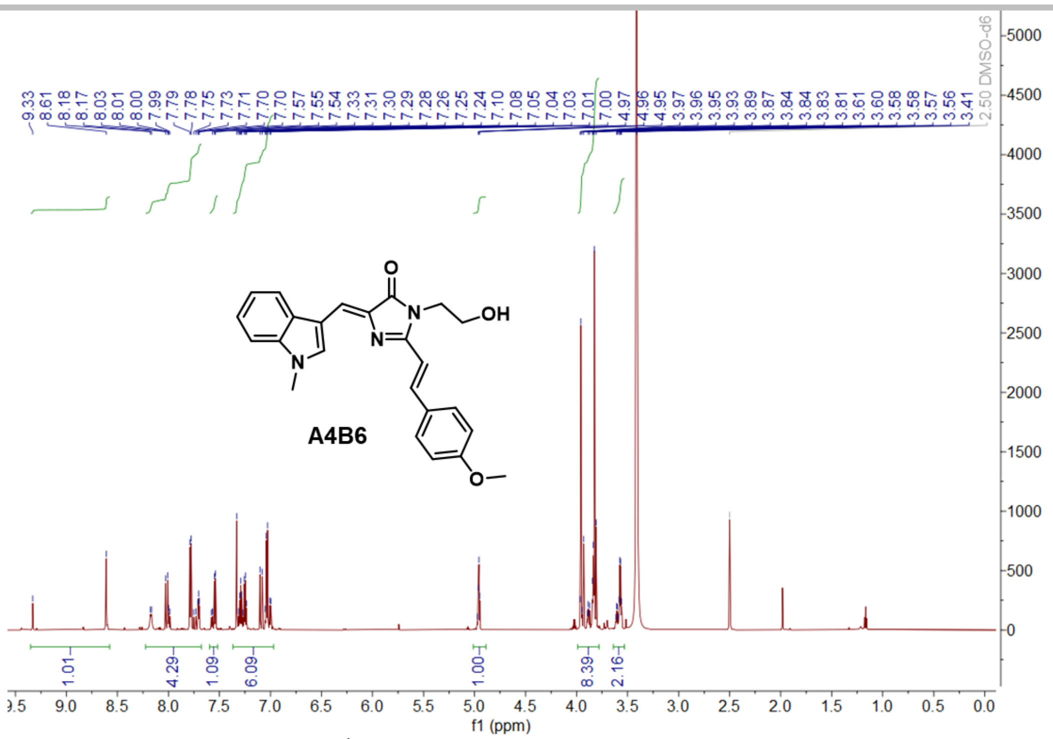

^{1}H NMR spectrum of A4B6 ($(CD_3)_2SO$)

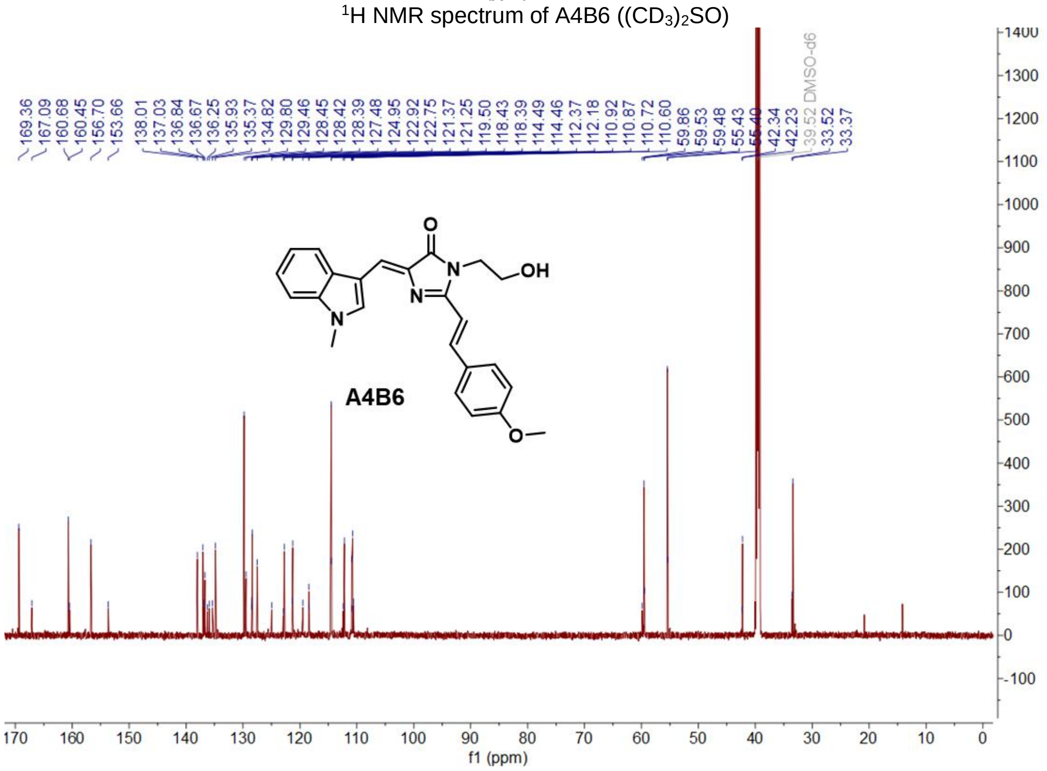

^{13}C NMR spectrum of A4B6 ($(CD_3)_2SO$)

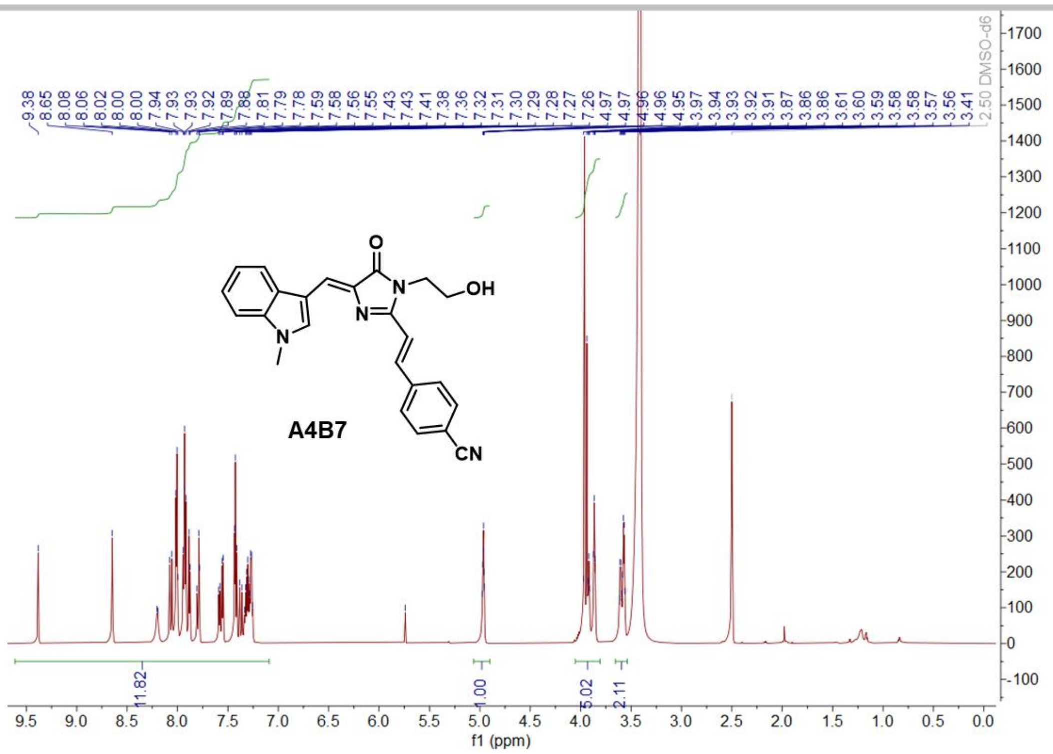

^{1}H NMR spectrum of A4B7 ($(CD_3)_2SO$)

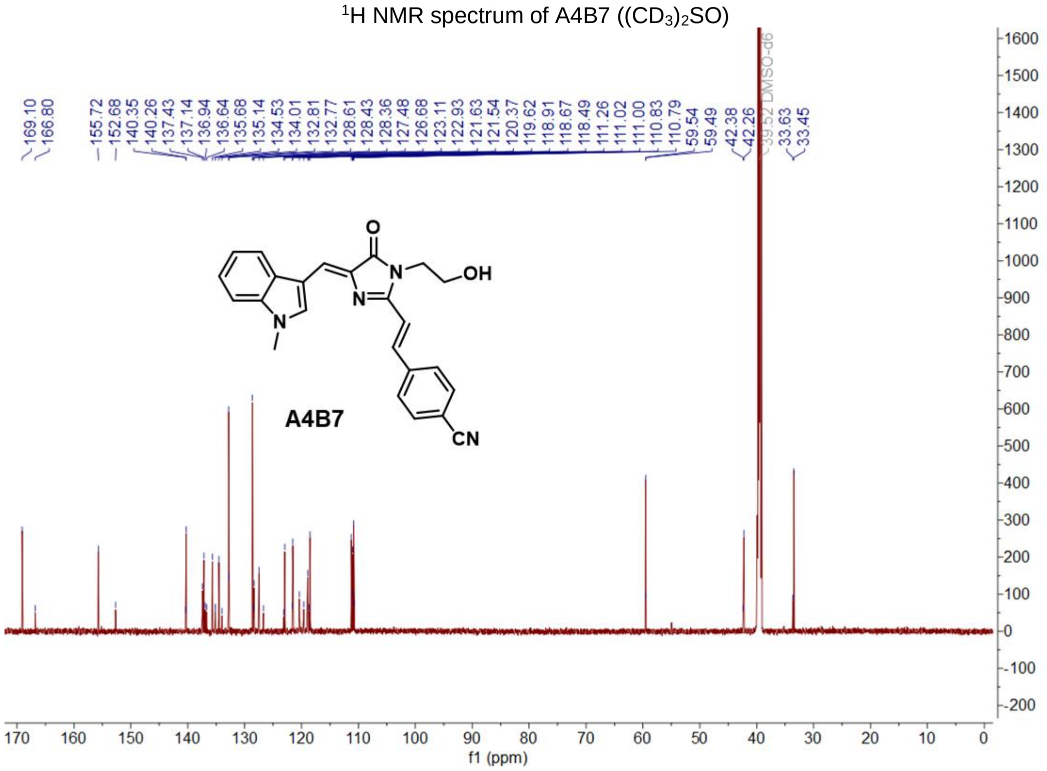

^{13}C NMR spectrum of A4B7 ($(CD_3)_2SO$)

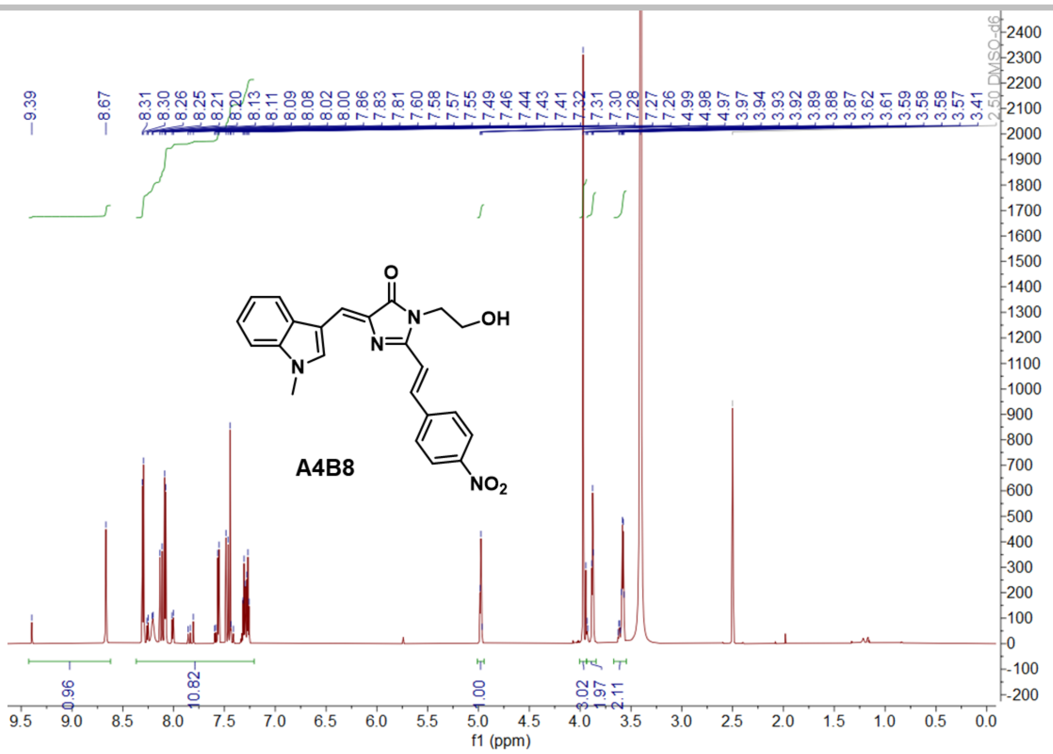

^{1}H NMR spectrum of A4B8 ($(CD_3)_2SO$)

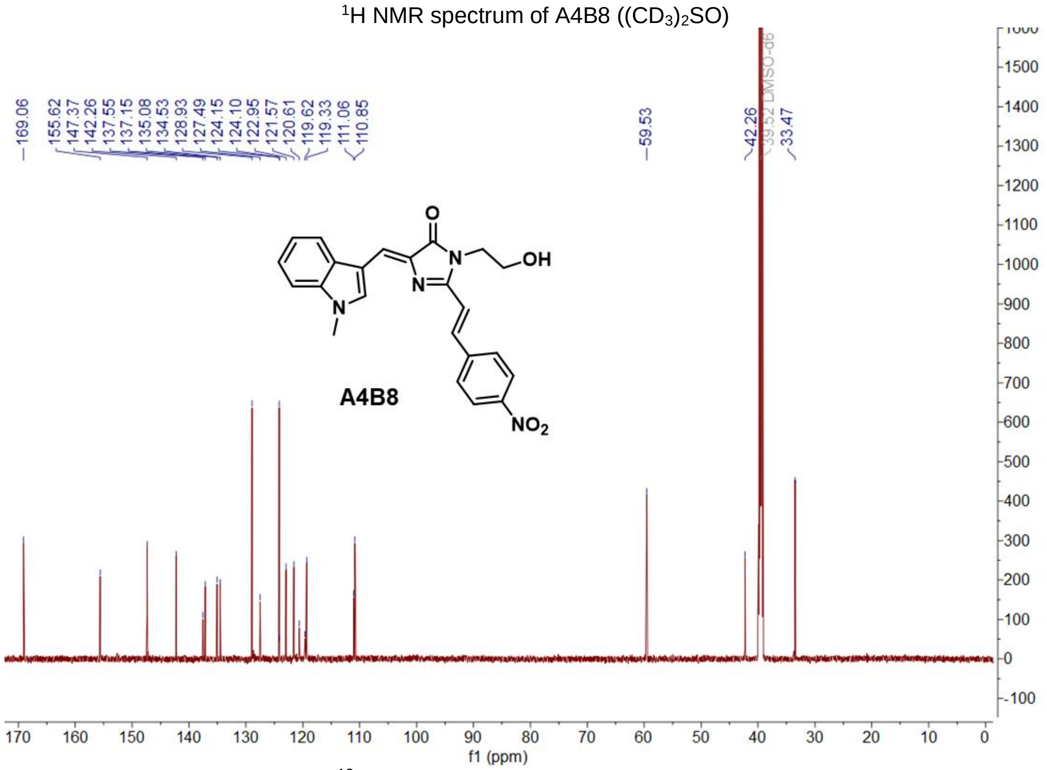

^{13}C NMR spectrum of A4B8 ($(CD_3)_2SO$)

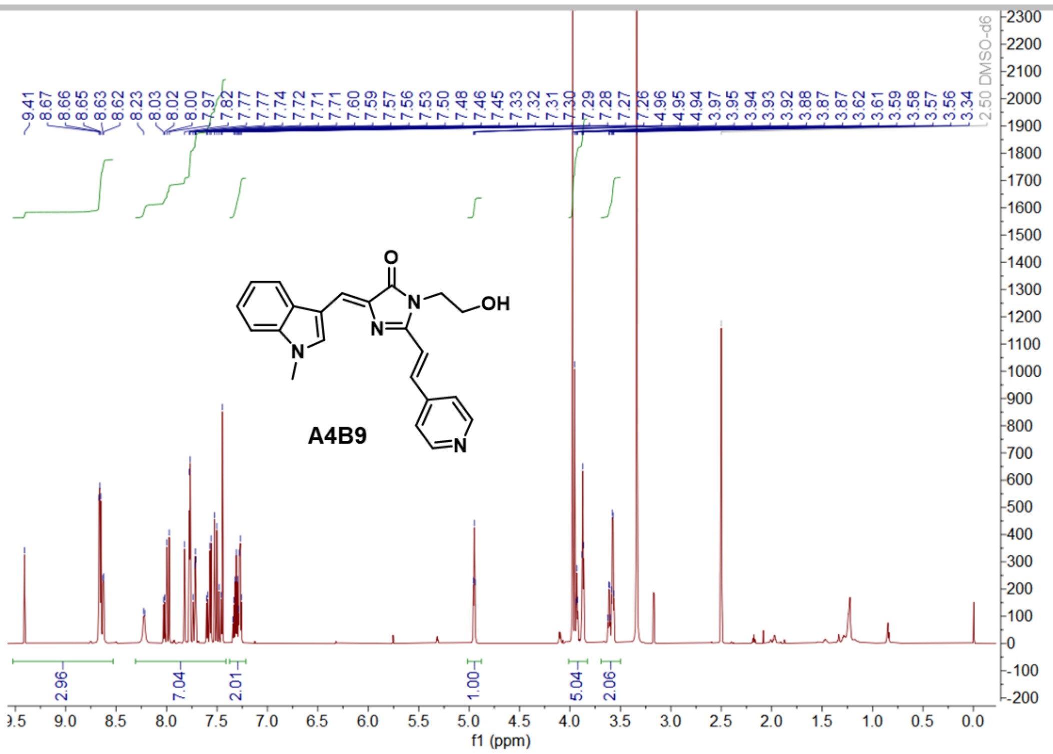

^{1}H NMR spectrum of A4B9 ($(CD_3)_2SO$)

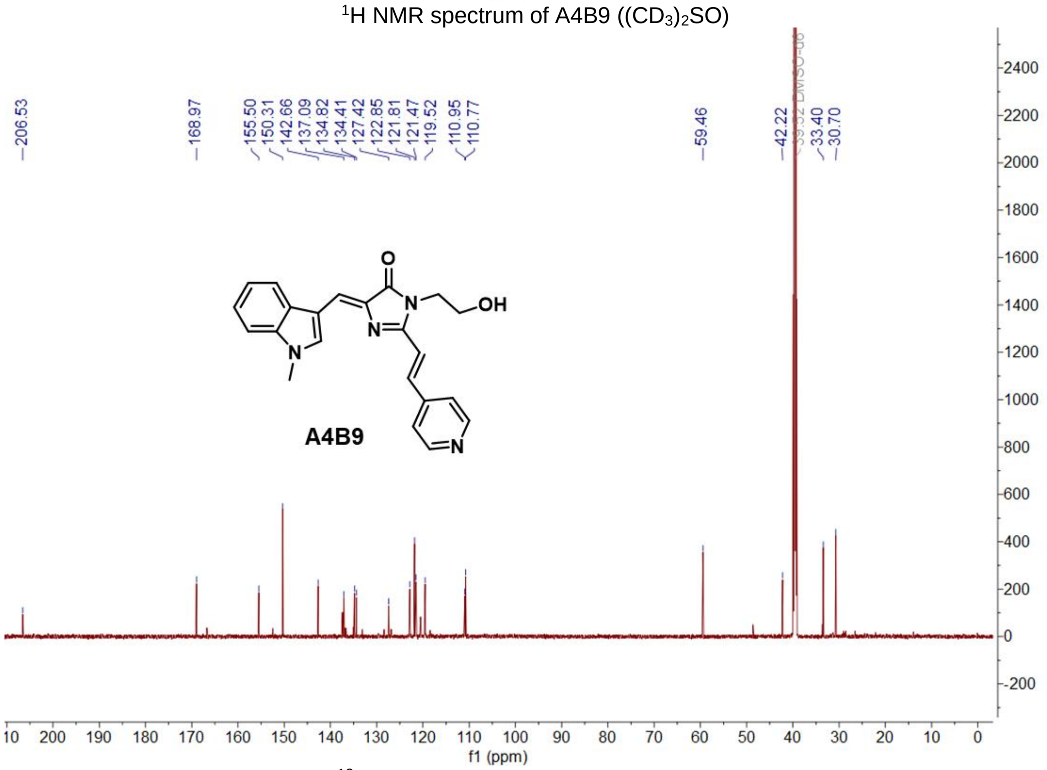

^{13}C NMR spectrum of A4B9 ($(CD_3)_2SO$)

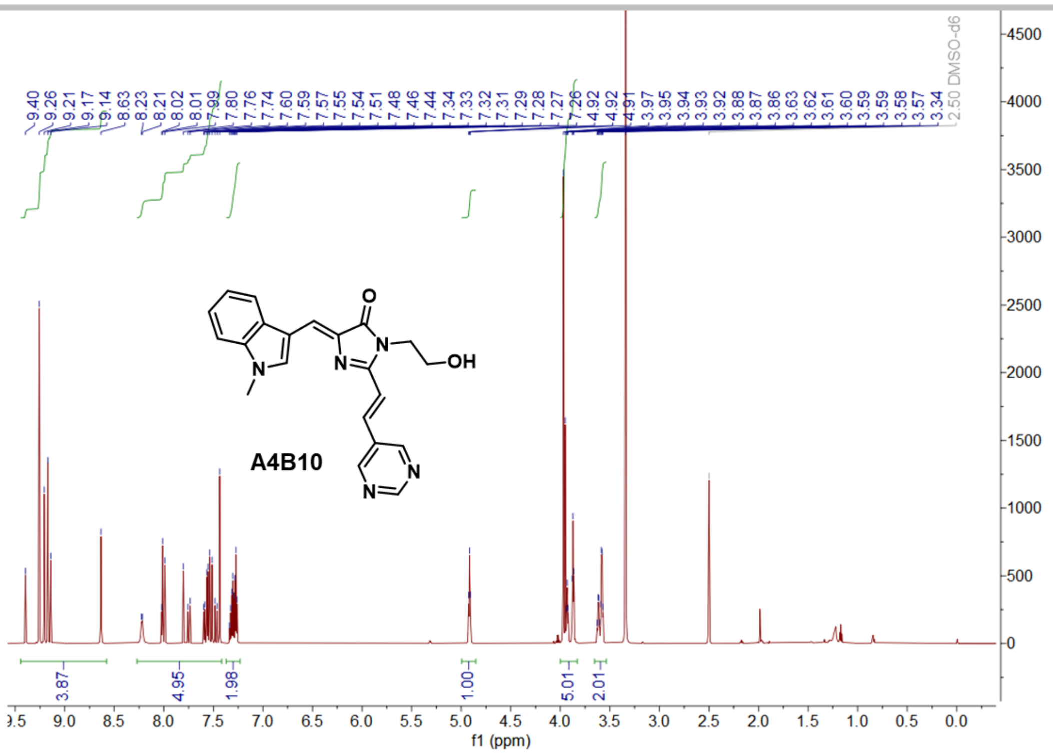

^1H NMR spectrum of A4B10 ($(CD_3)_2SO$)

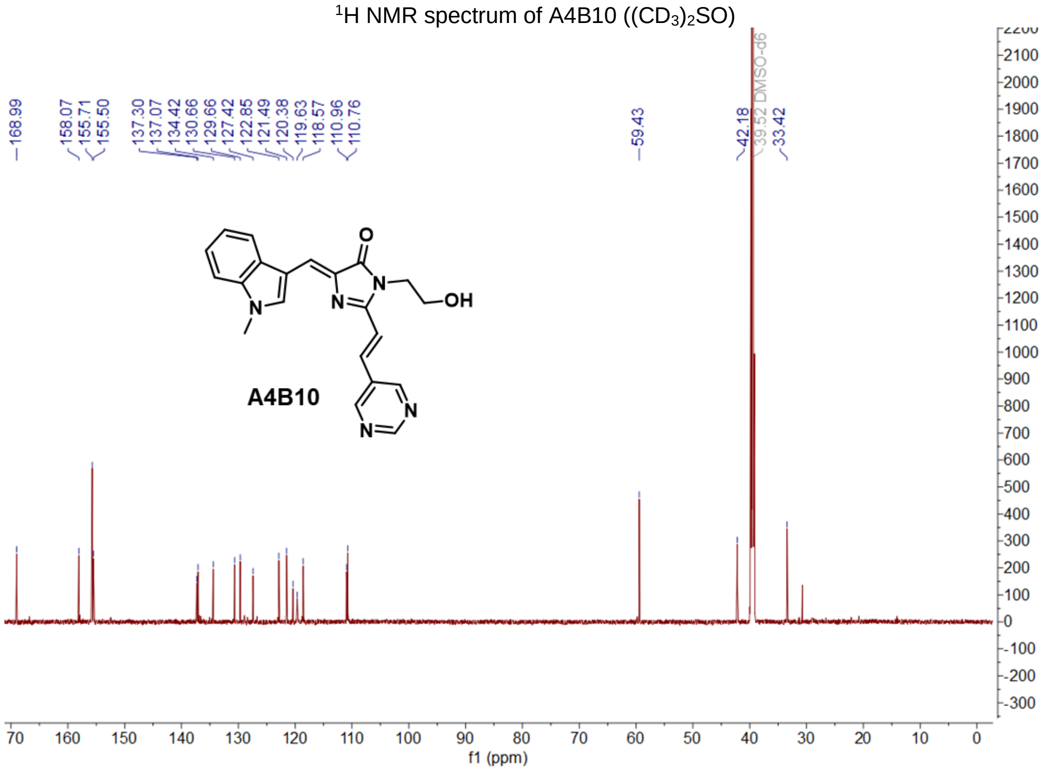

^{13}C NMR spectrum of A4B10 ($(CD_3)_2SO$)

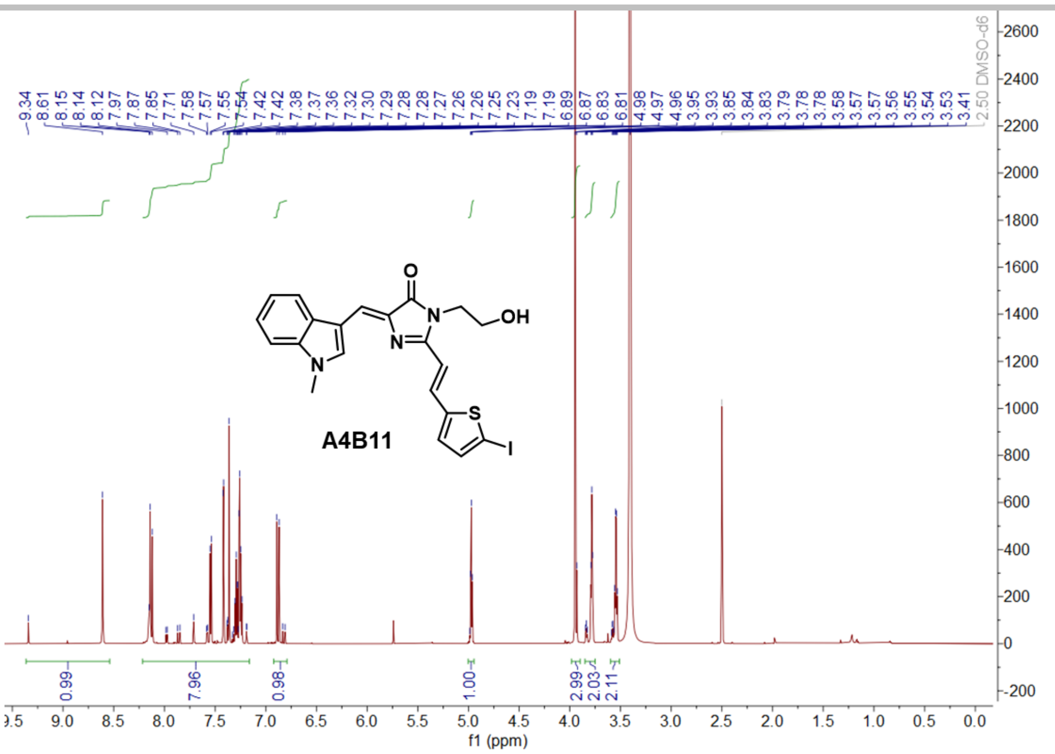

^{1}H NMR spectrum of A4B11 ($(CD_3)_2SO$)

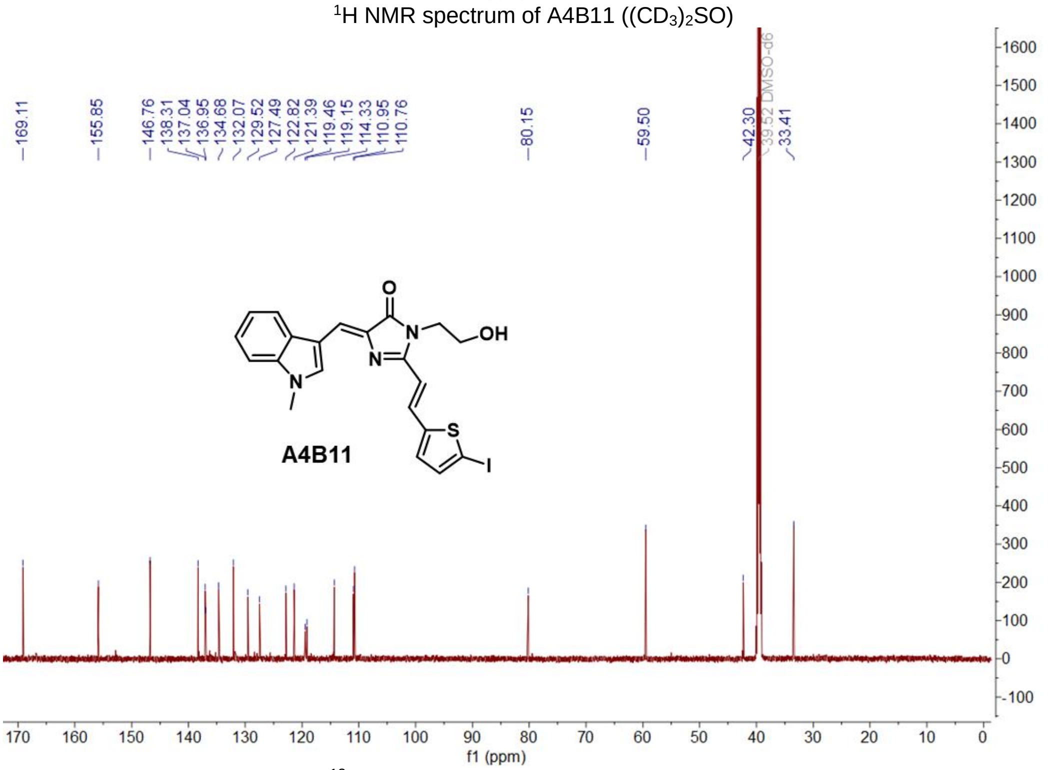

^{13}C NMR spectrum of A4B11 ($(CD_3)_2SO$)

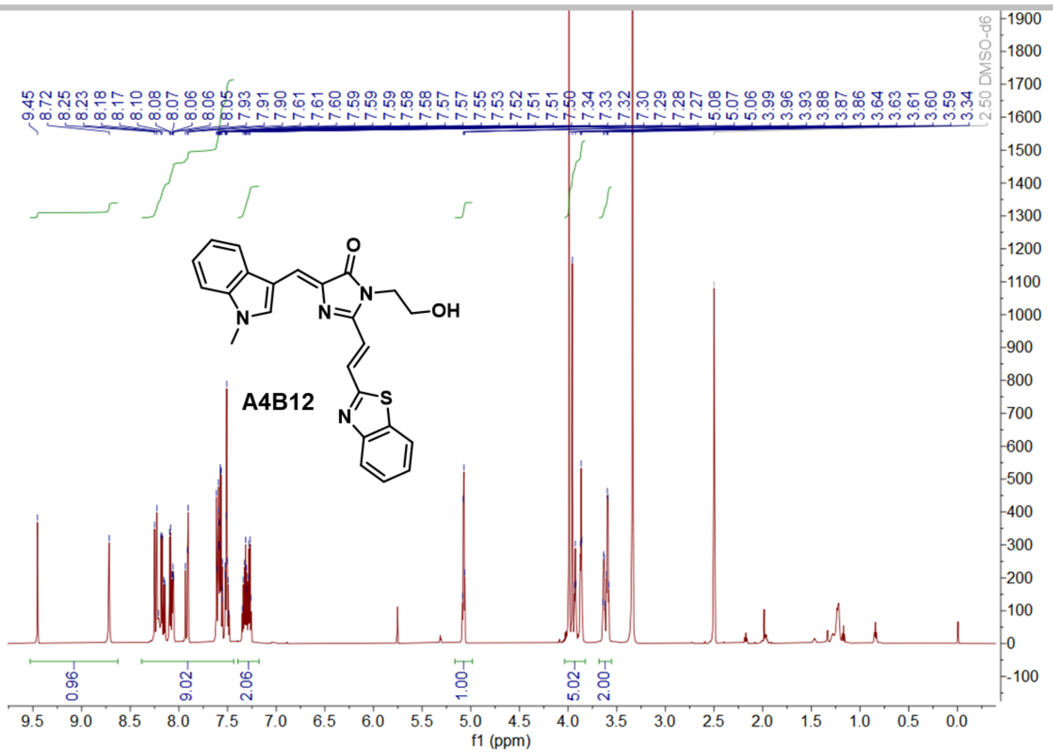

^1H NMR spectrum of A4B12 ($(CD_3)_2SO$)

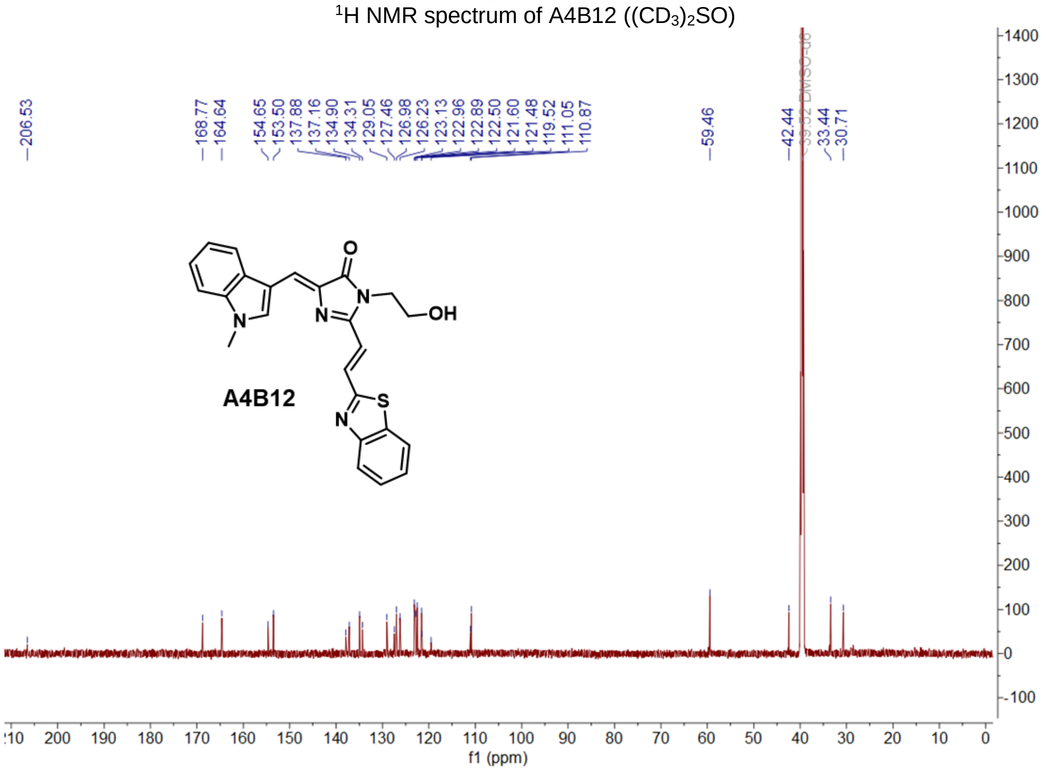

^{13}C NMR spectrum of A4B12 ($(CD_3)_2SO$)

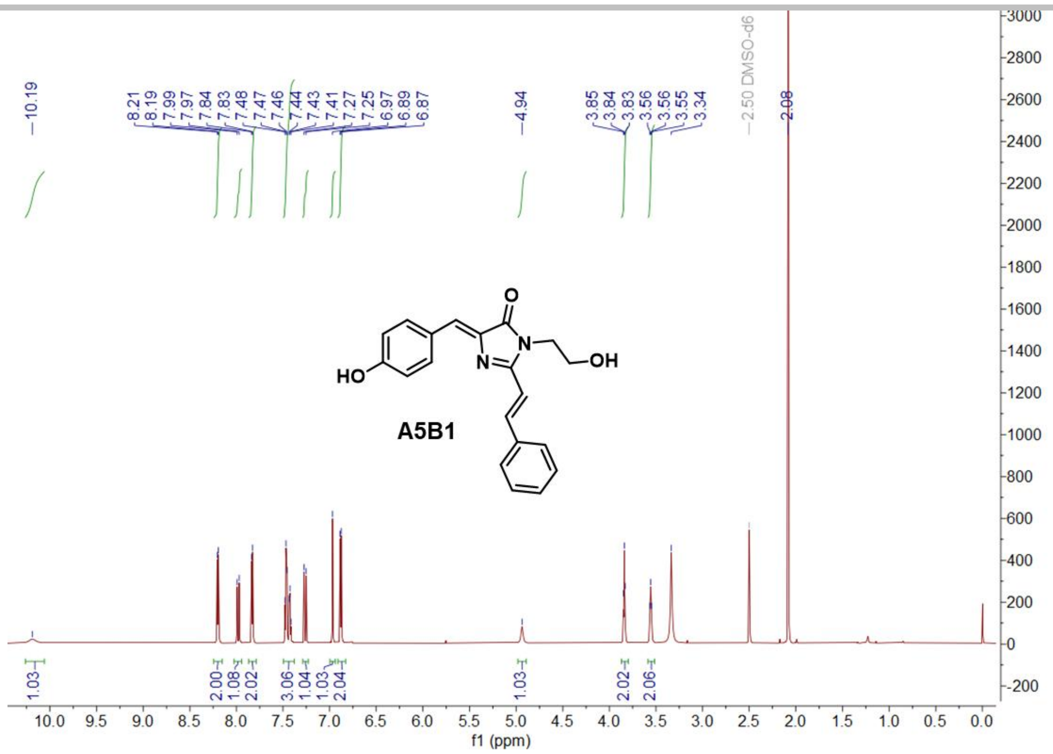

^{1}H NMR spectrum of A5B1 ($(CD_3)_2SO$)

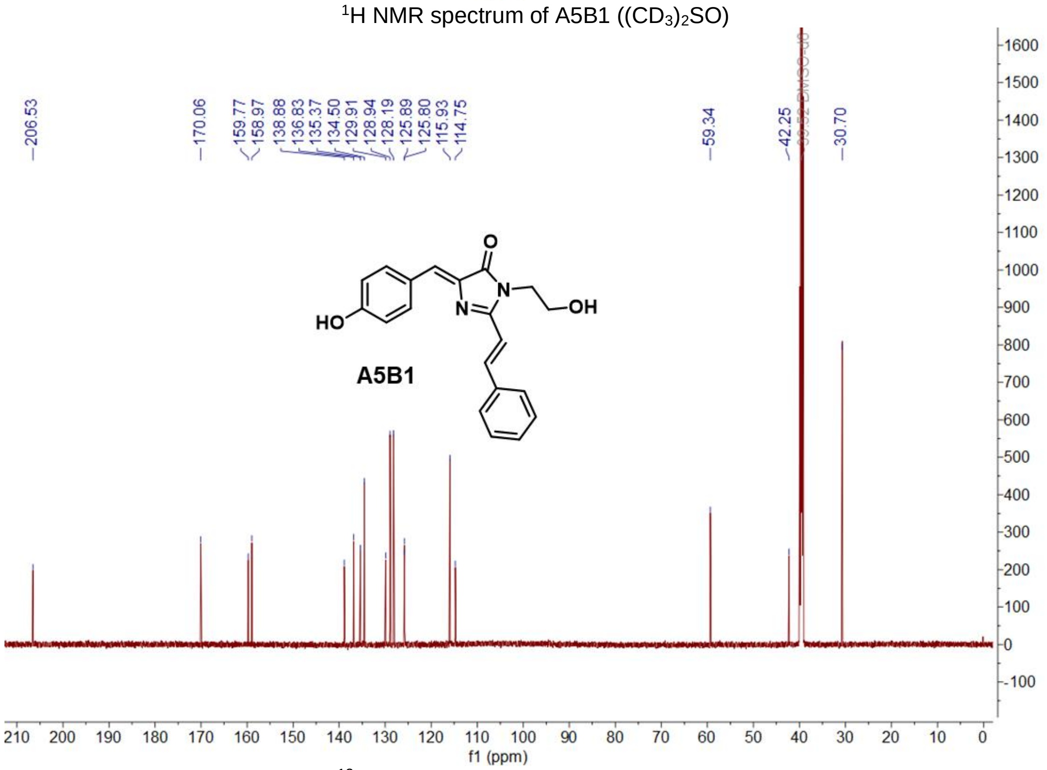

^{13}C NMR spectrum of A5B1 ($(CD_3)_2SO$)

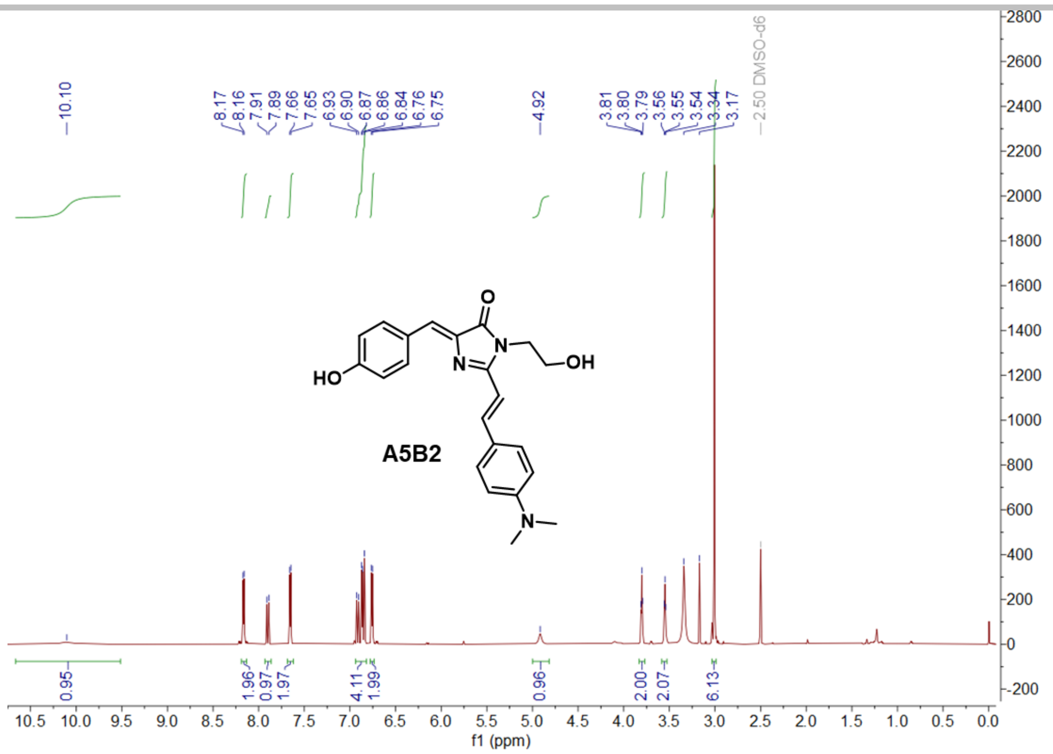

^{1}H NMR spectrum of A5B2 ($(CD_3)_2SO$)

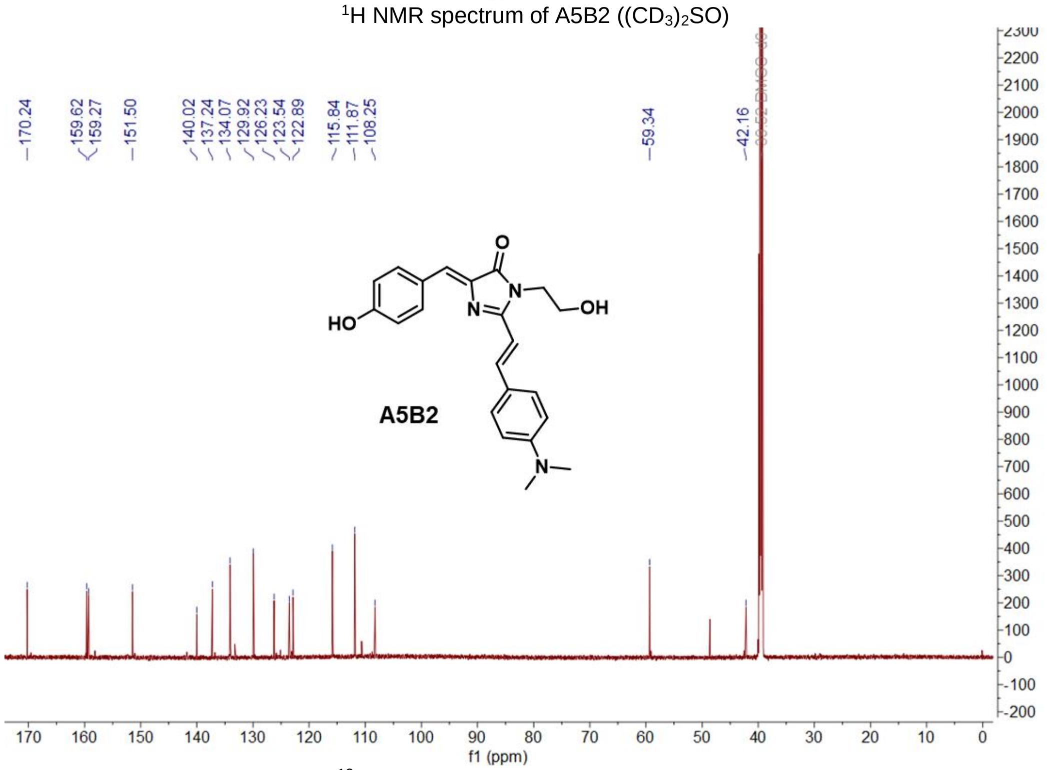

^{13}C NMR spectrum of A5B2 ($(CD_3)_2SO$)

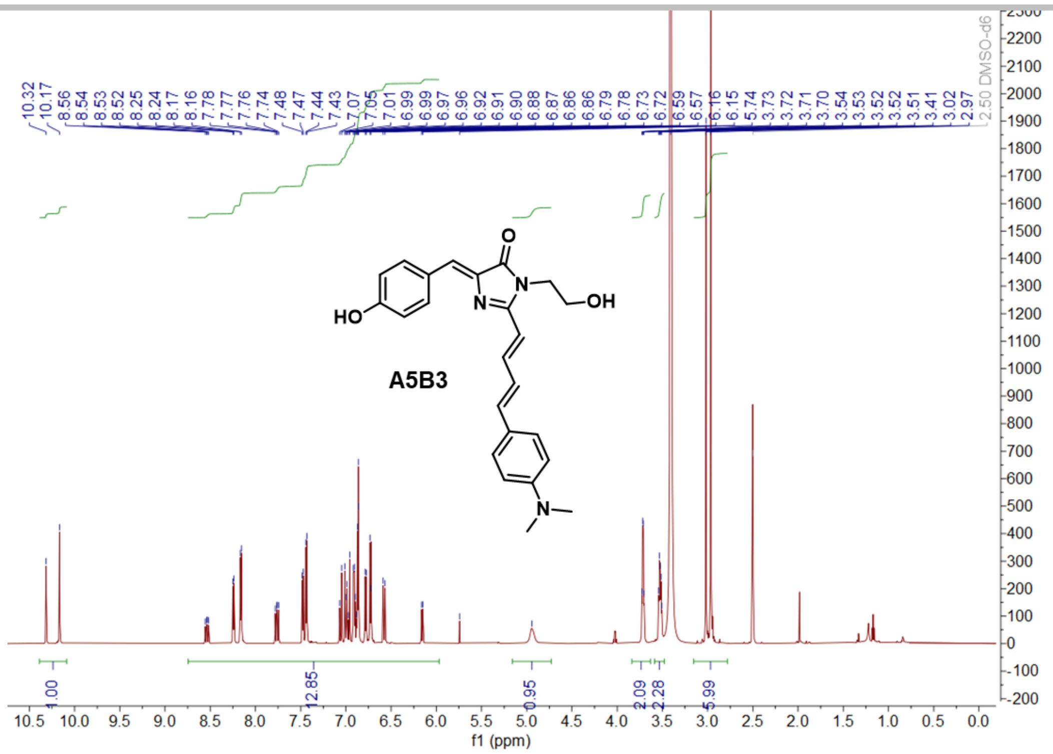

^{1}H NMR spectrum of A5B3 ($(CD_3)_2SO$)

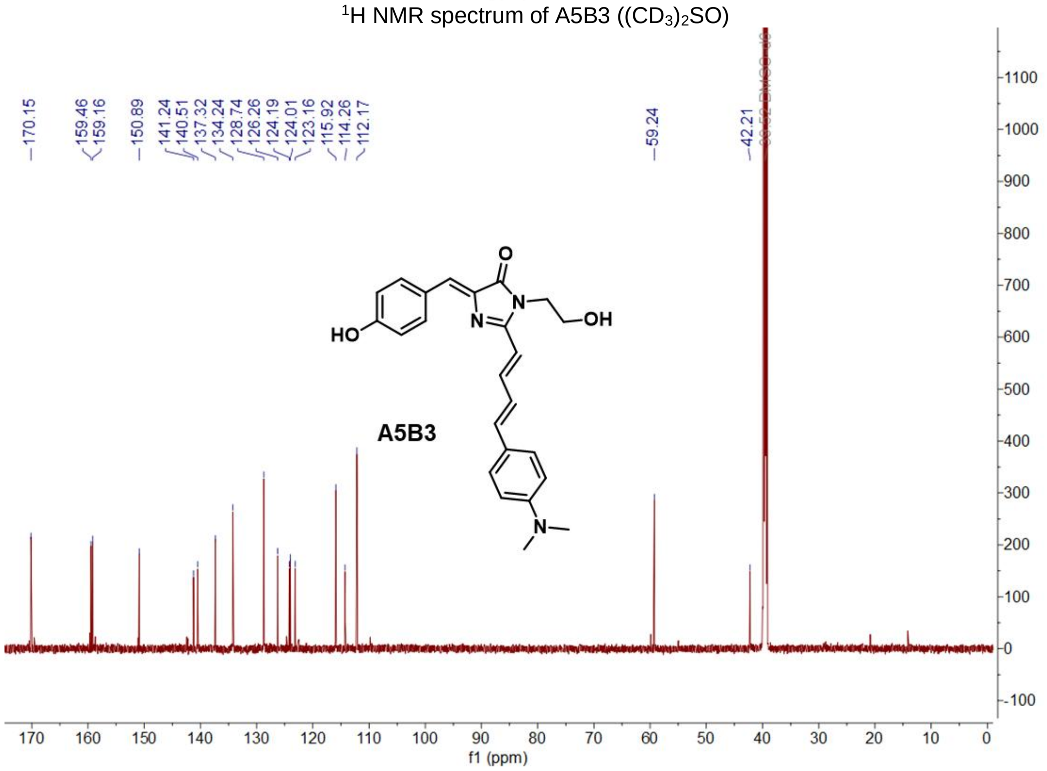

^{13}C NMR spectrum of A5B3 ($(CD_3)_2SO$)

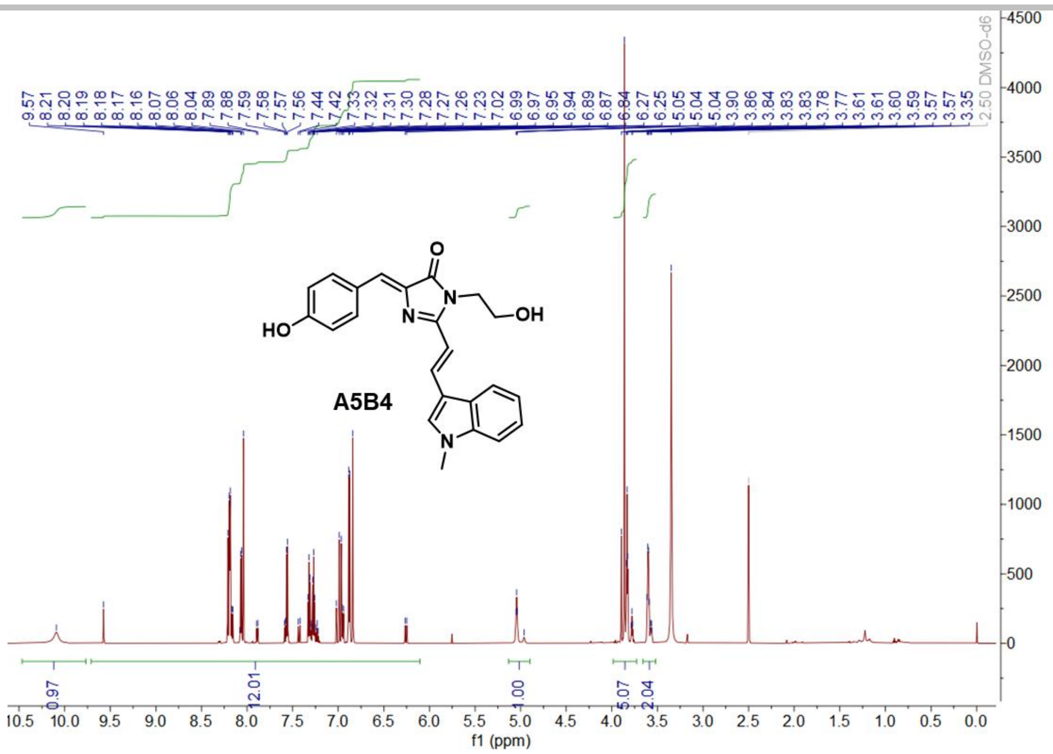

^{1}H NMR spectrum of A5B4 ($(CD_3)_2SO$)

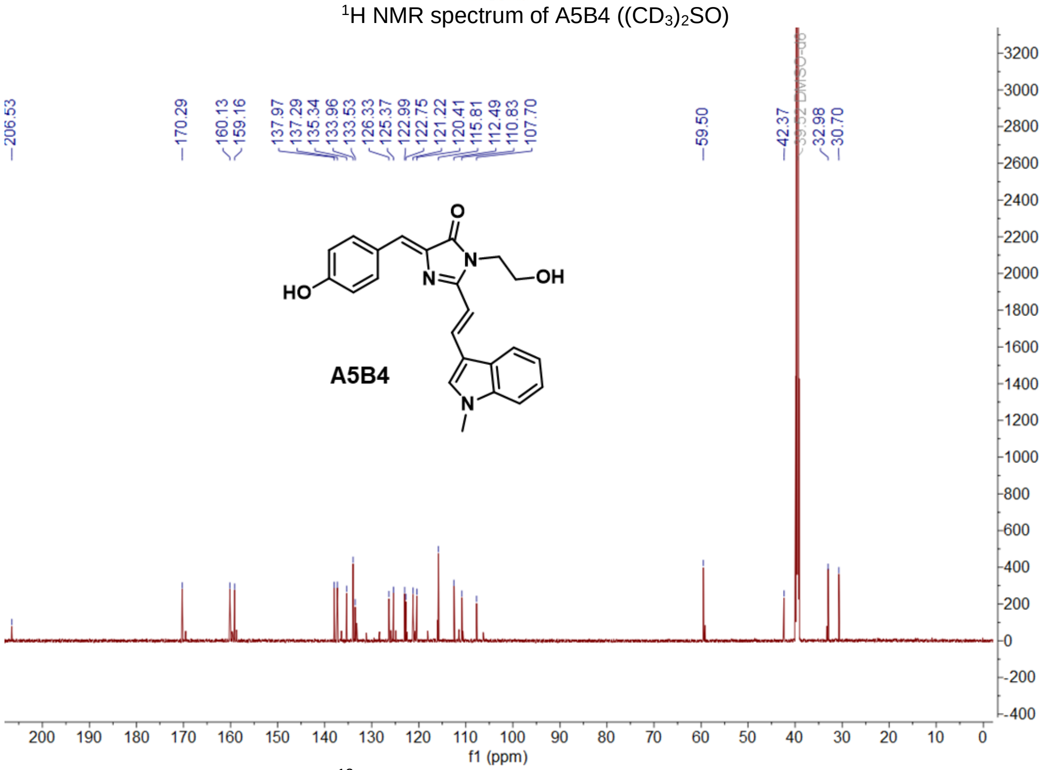

^{13}C NMR spectrum of A5B4 ($(CD_3)_2SO$)

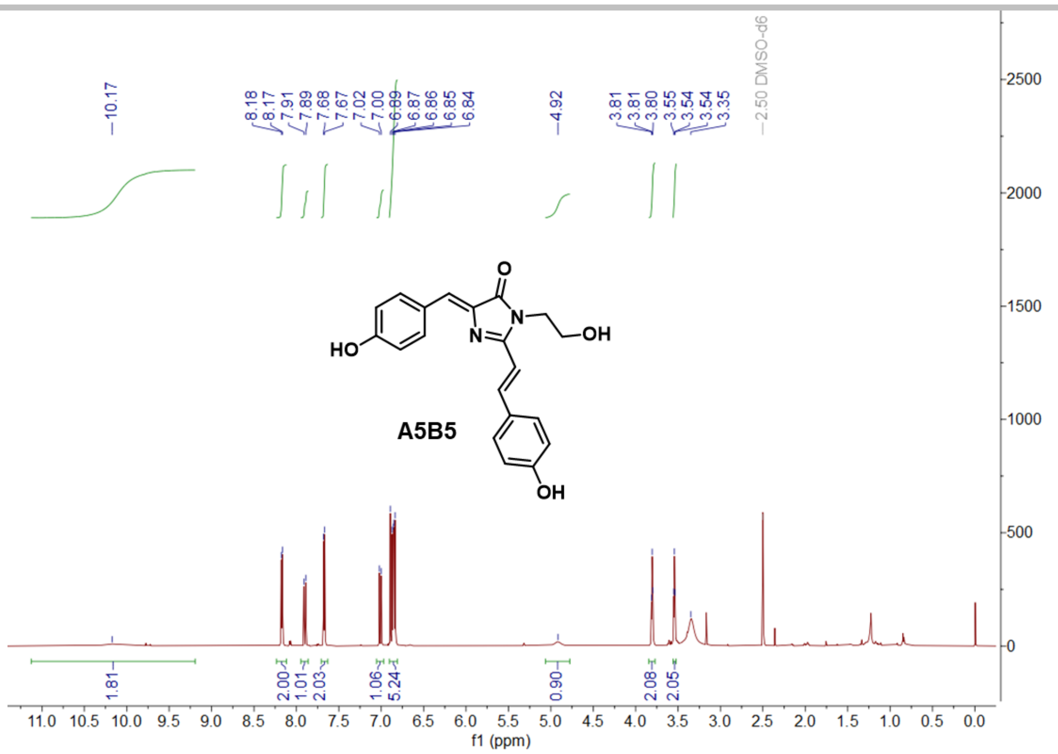

^{1}H NMR spectrum of A5B5 ($(CD_3)_2SO$)

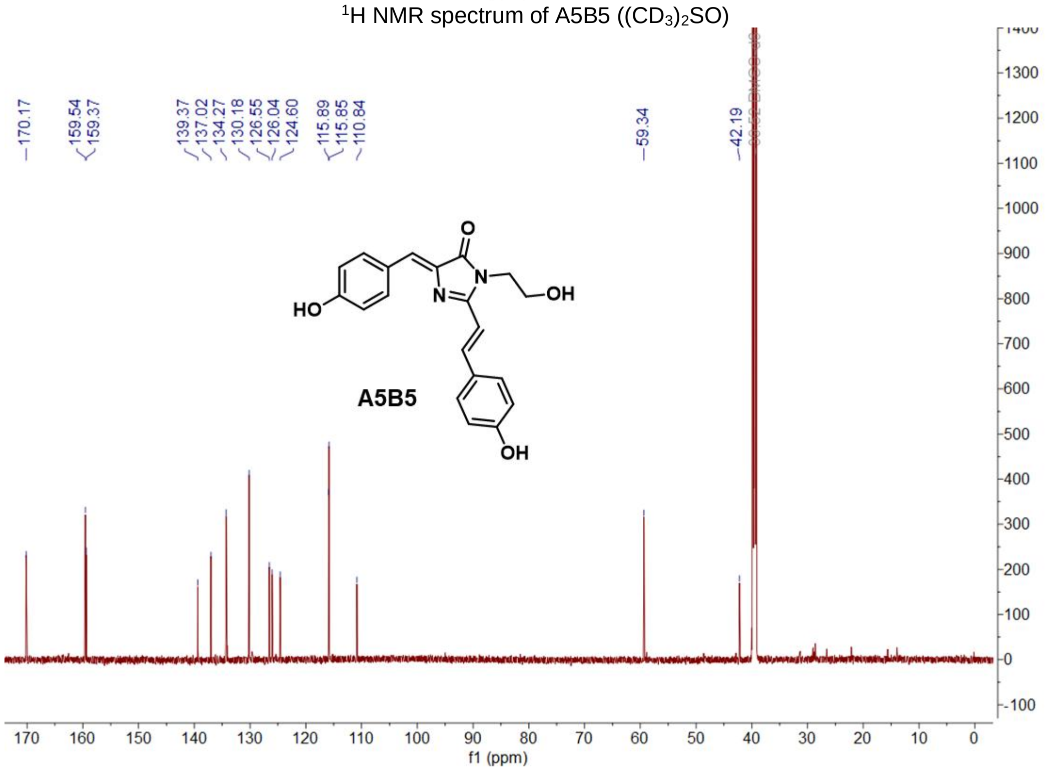

^{13}C NMR spectrum of A5B5 ($(CD_3)_2SO$)

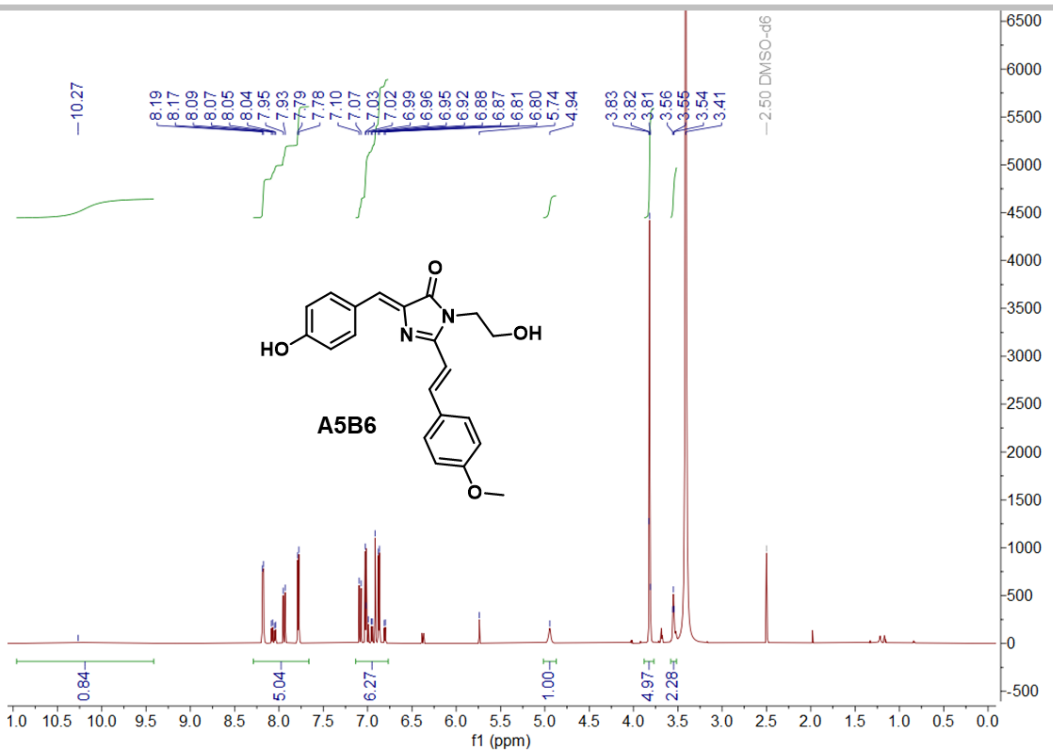

^{1}H NMR spectrum of A5B6 ($(CD_3)_2SO$)

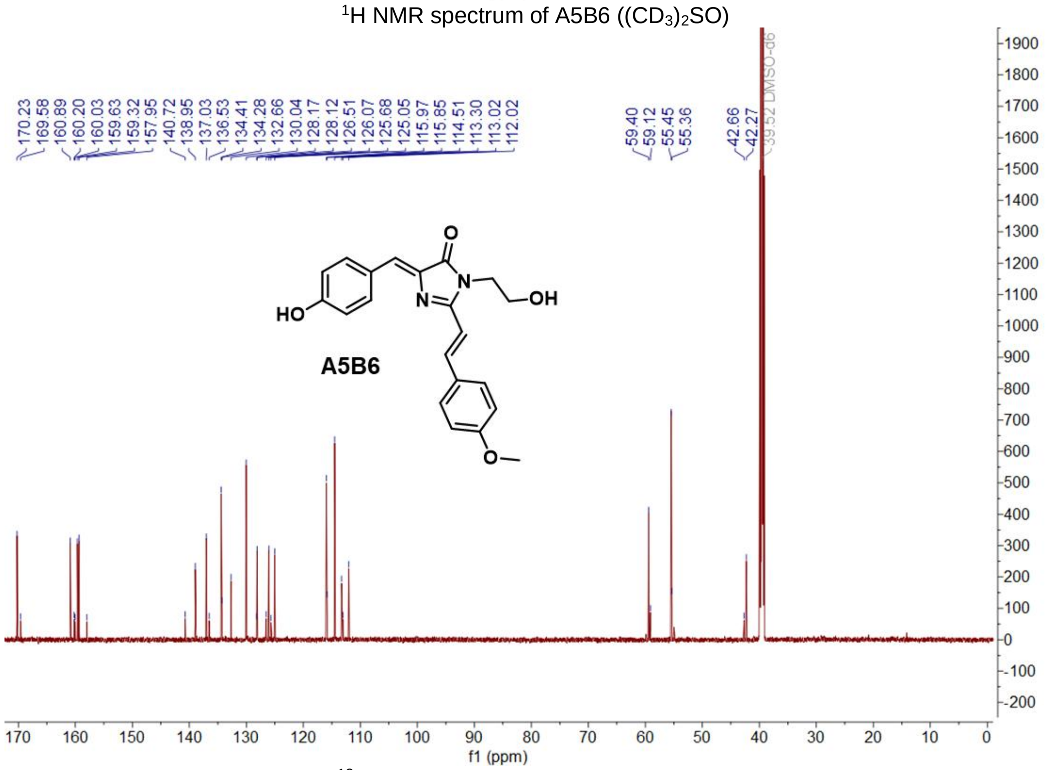

^{13}C NMR spectrum of A5B6 ($(CD_3)_2SO$)

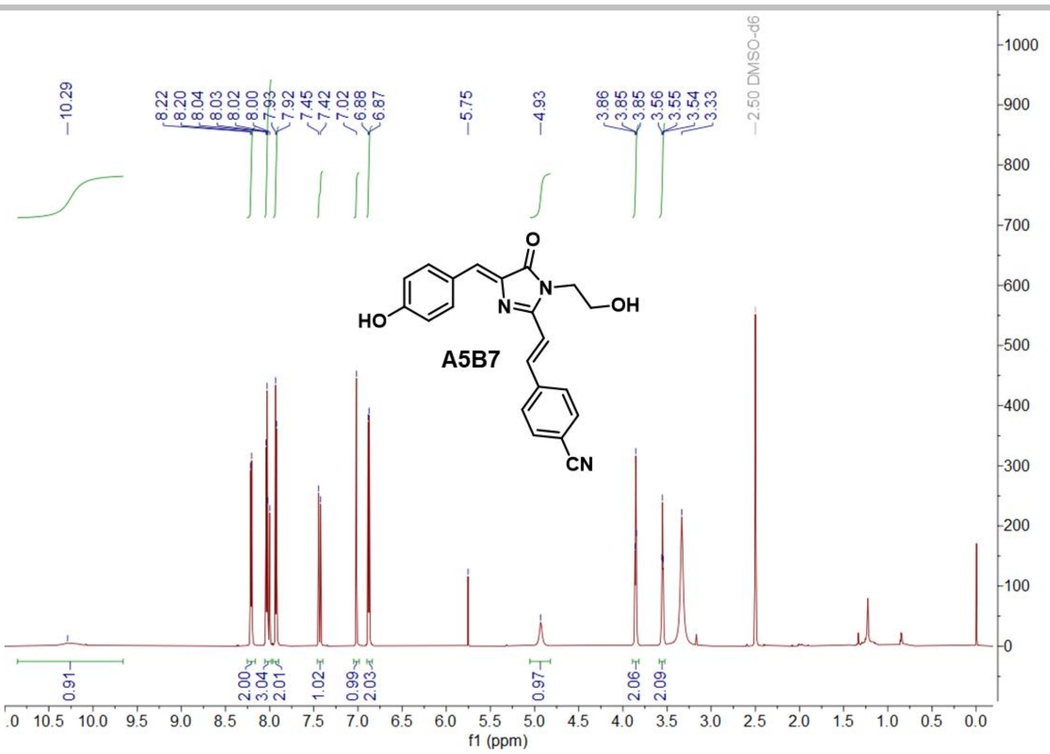

^{1}H NMR spectrum of A5B7 ($(CD_3)_2SO$)

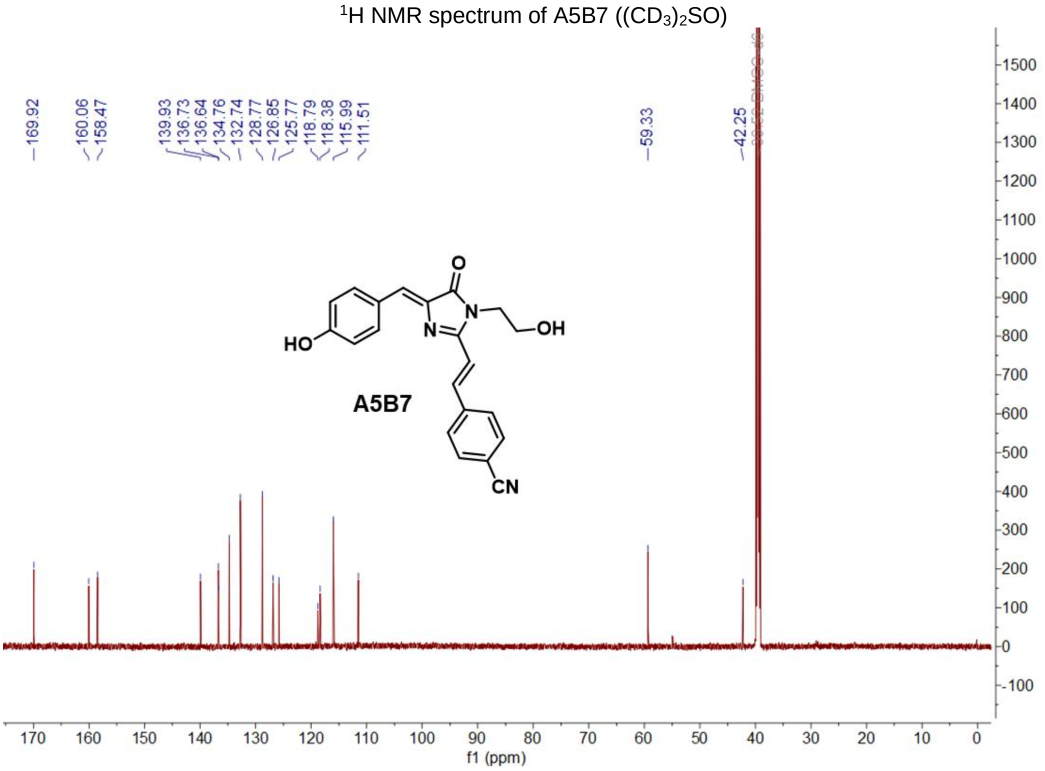

^{13}C NMR spectrum of A5B7 ($(CD_3)_2SO$)

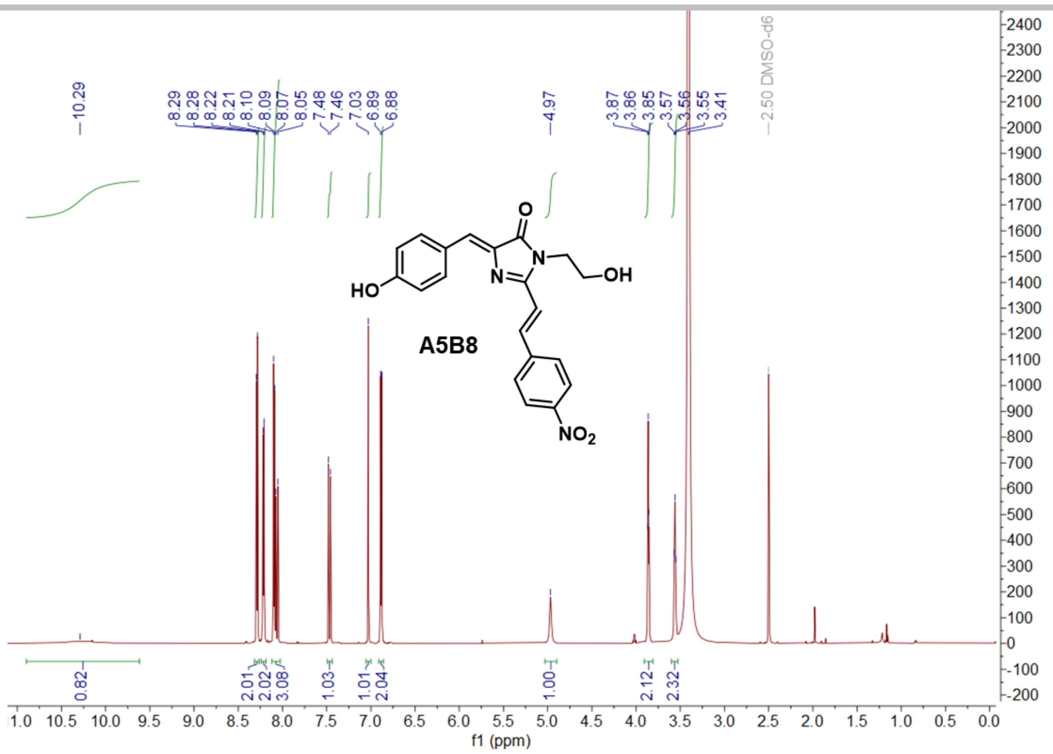

^{1}H NMR spectrum of A5B8 ($(CD_3)_2SO$)

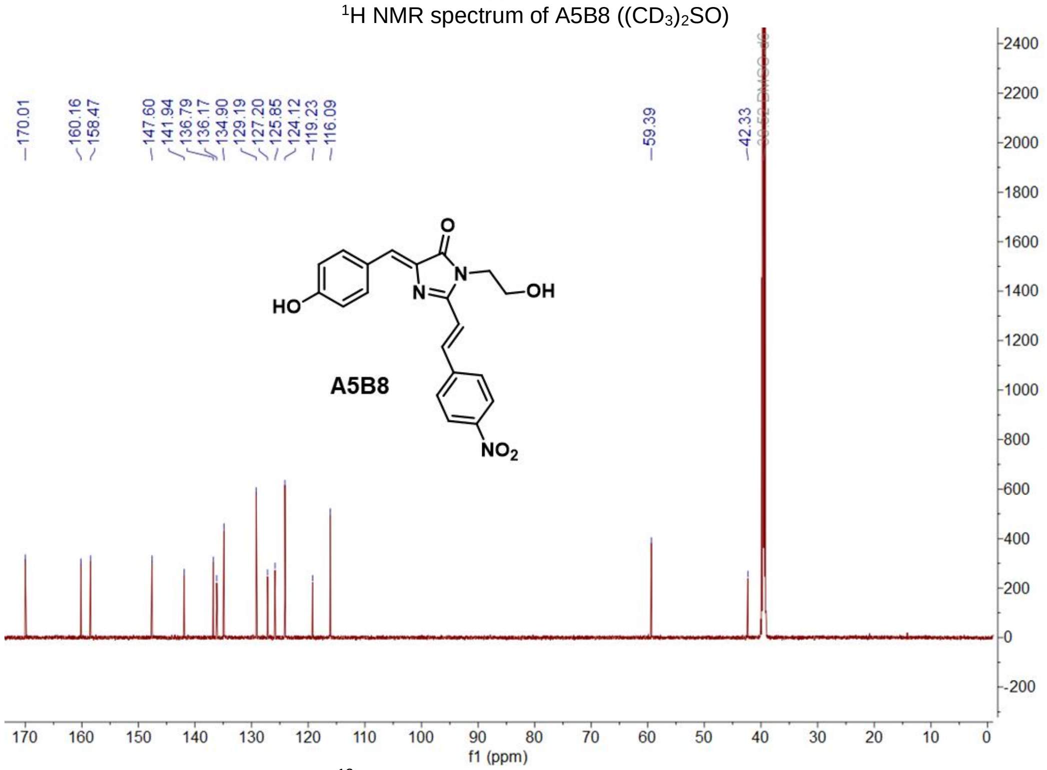

^{13}C NMR spectrum of A5B8 ($(CD_3)_2SO$)

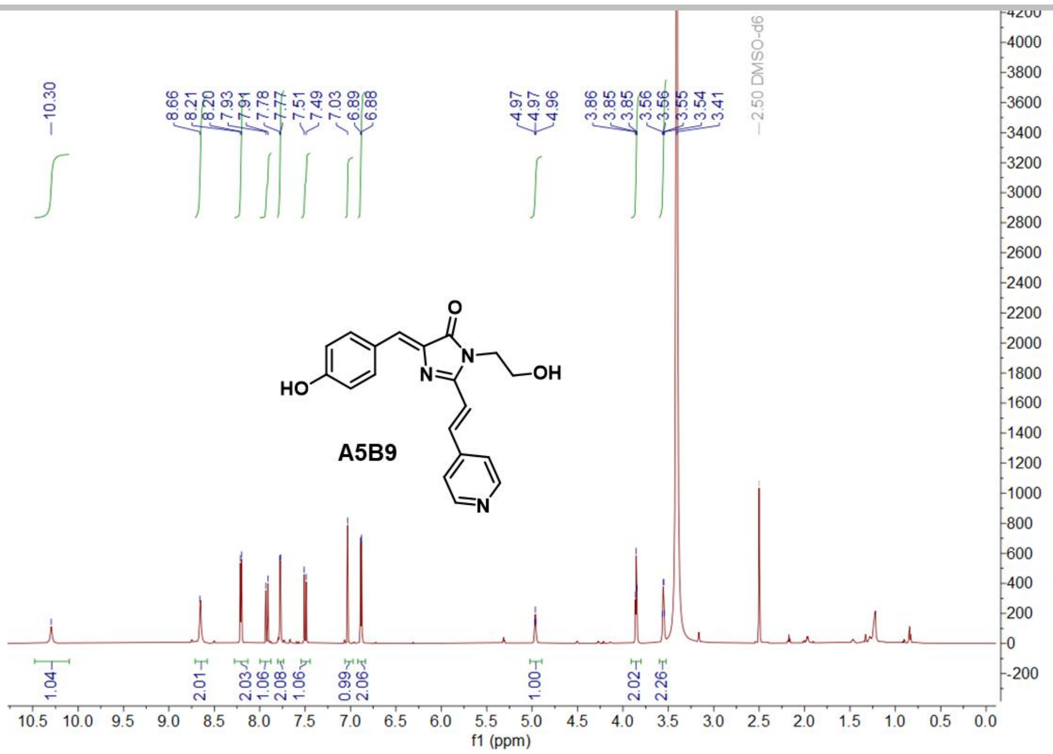

^{1}H NMR spectrum of A5B9 ($(CD_3)_2SO$)

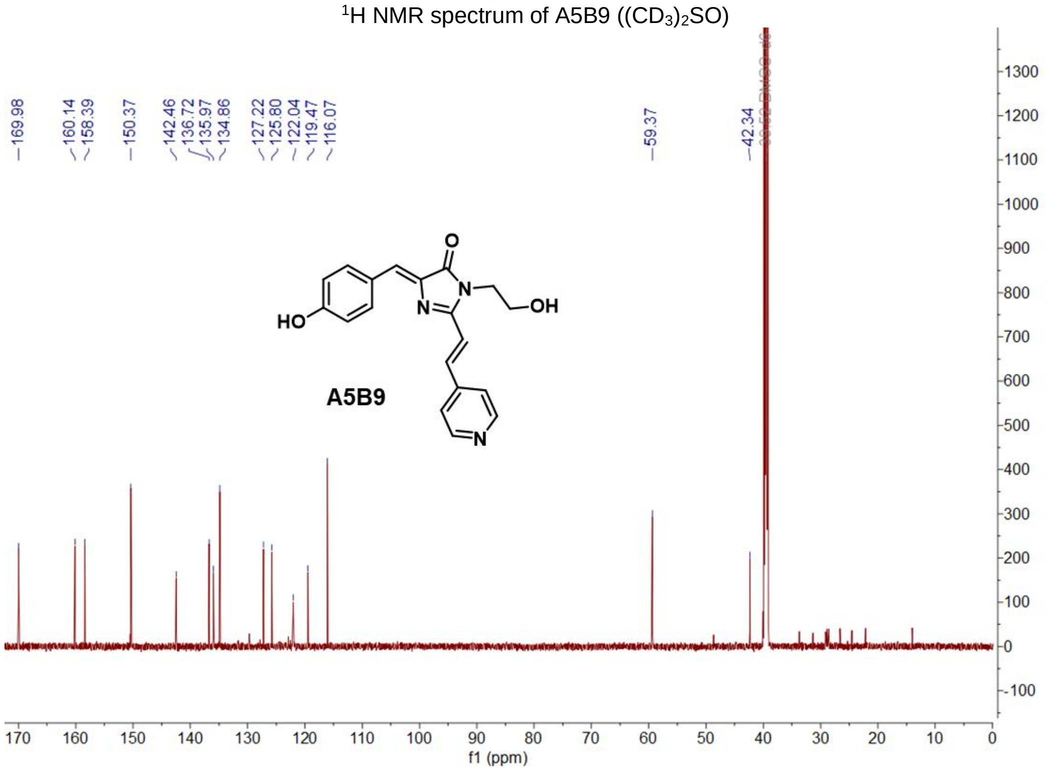

^{13}C NMR spectrum of A5B9 ($(CD_3)_2SO$)

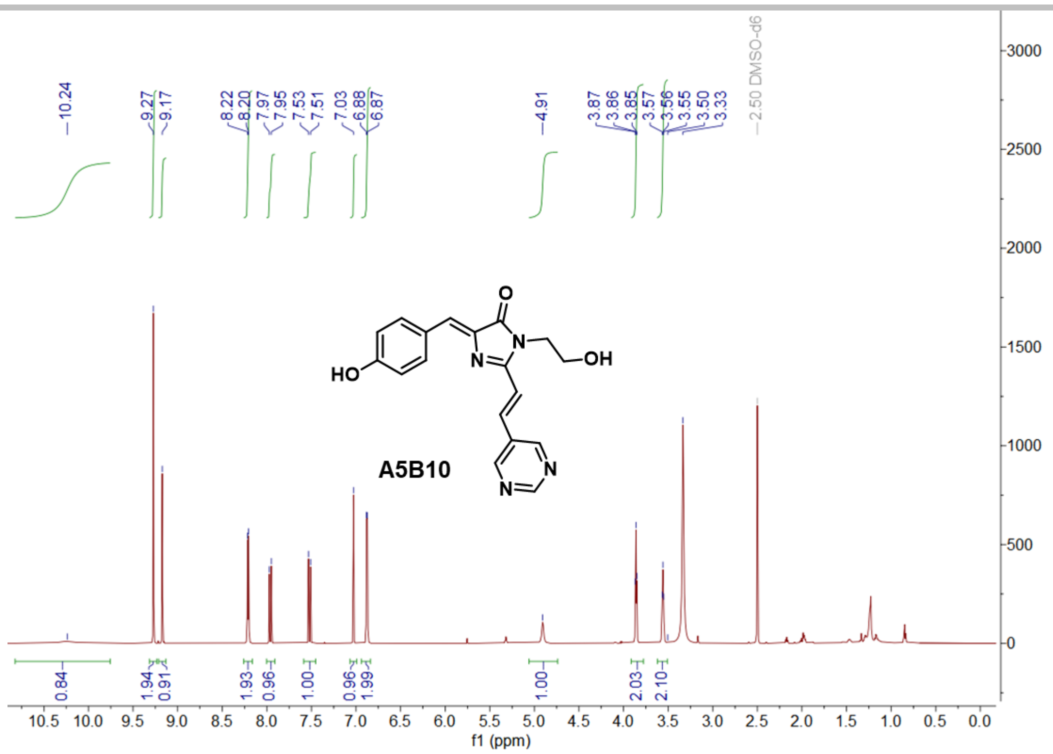

^1H NMR spectrum of A5B10 ($(CD_3)_2SO$)

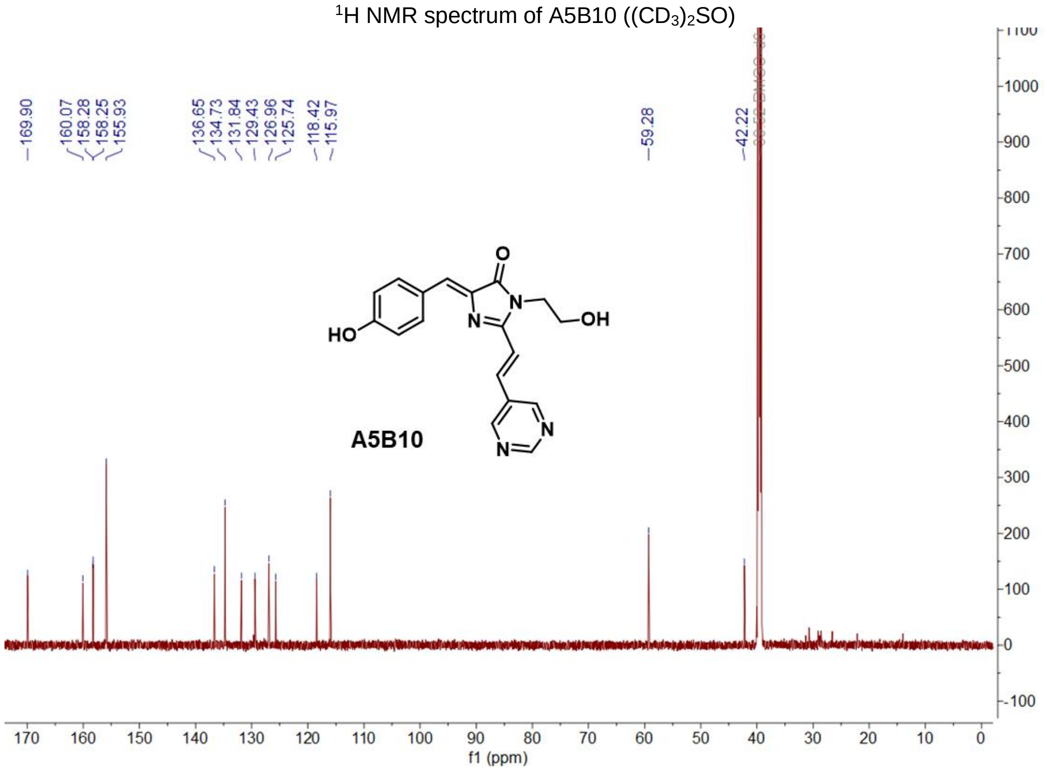

^{13}C NMR spectrum of A5B10 ($(CD_3)_2SO$)

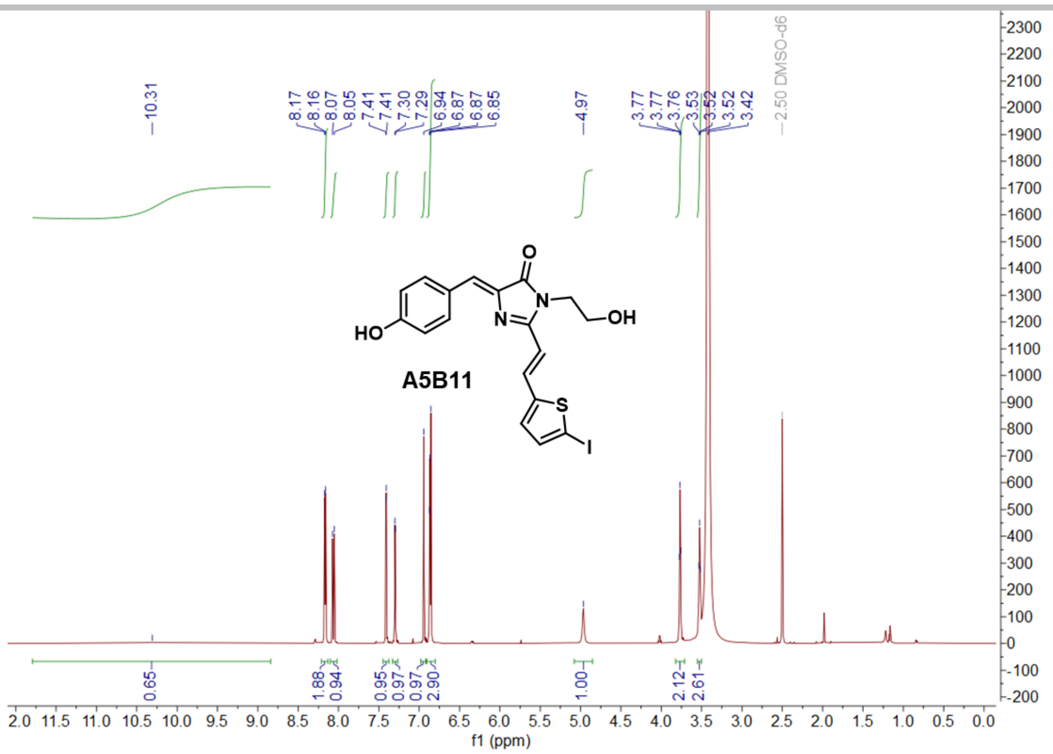

^{1}H NMR spectrum of A5B11 ($(CD_3)_2SO$)

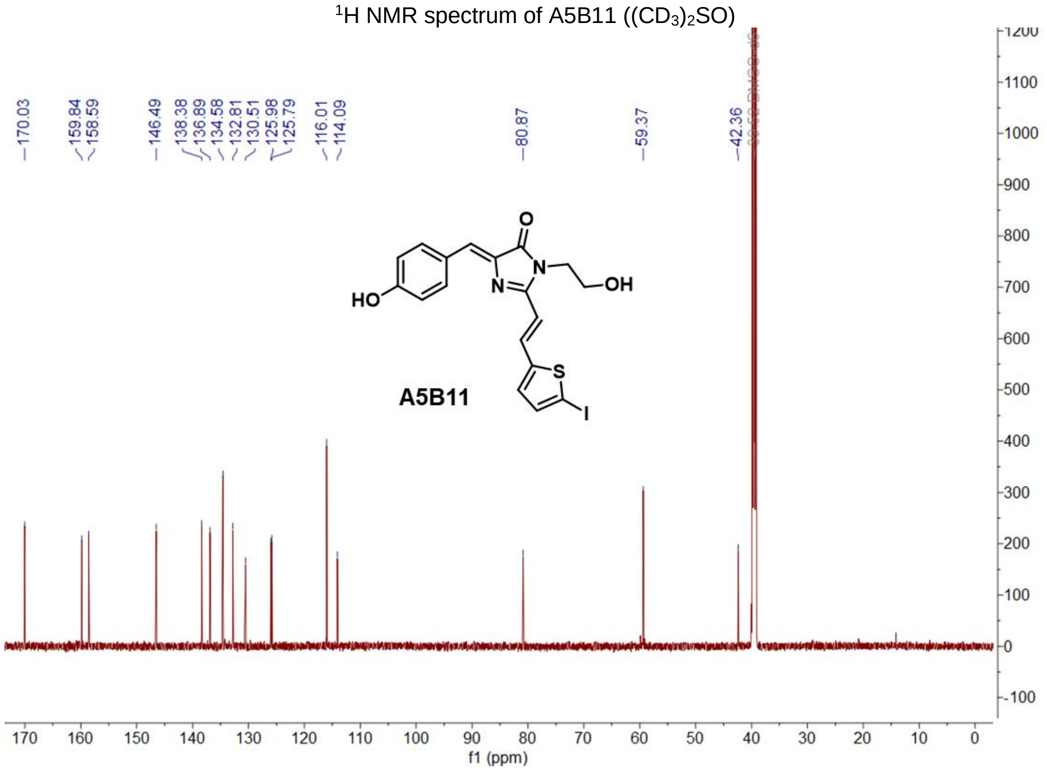

^{13}C NMR spectrum of A5B11 ($(CD_3)_2SO$)

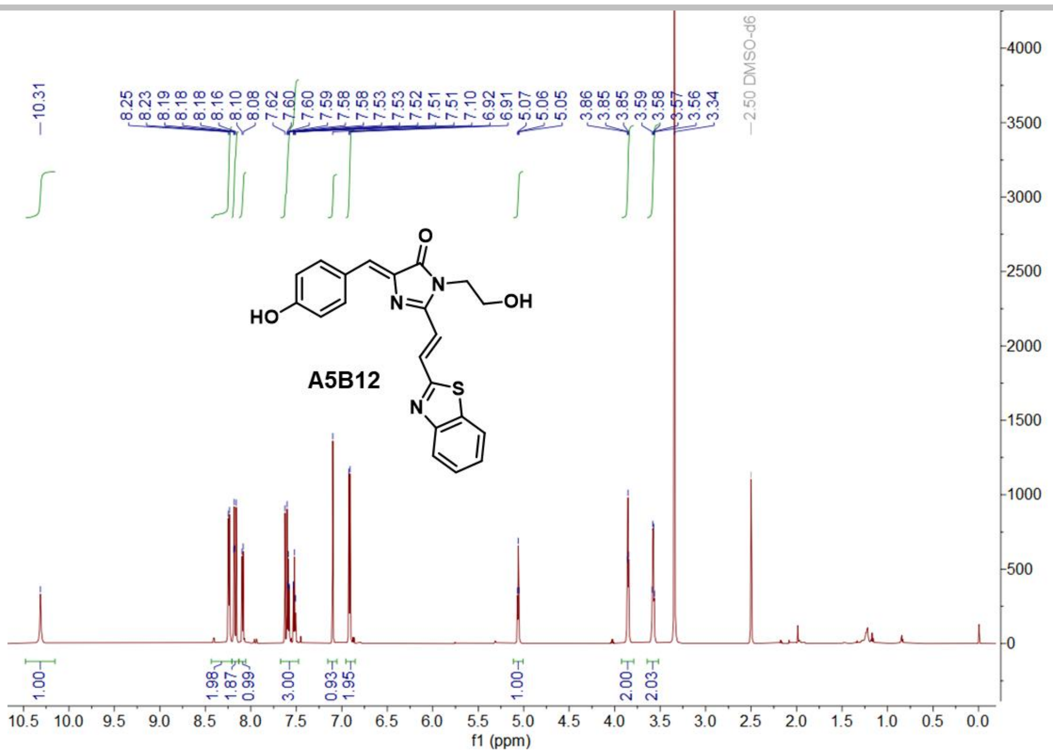

^{1}H NMR spectrum of A5B12 ($(CD_3)_2SO$)

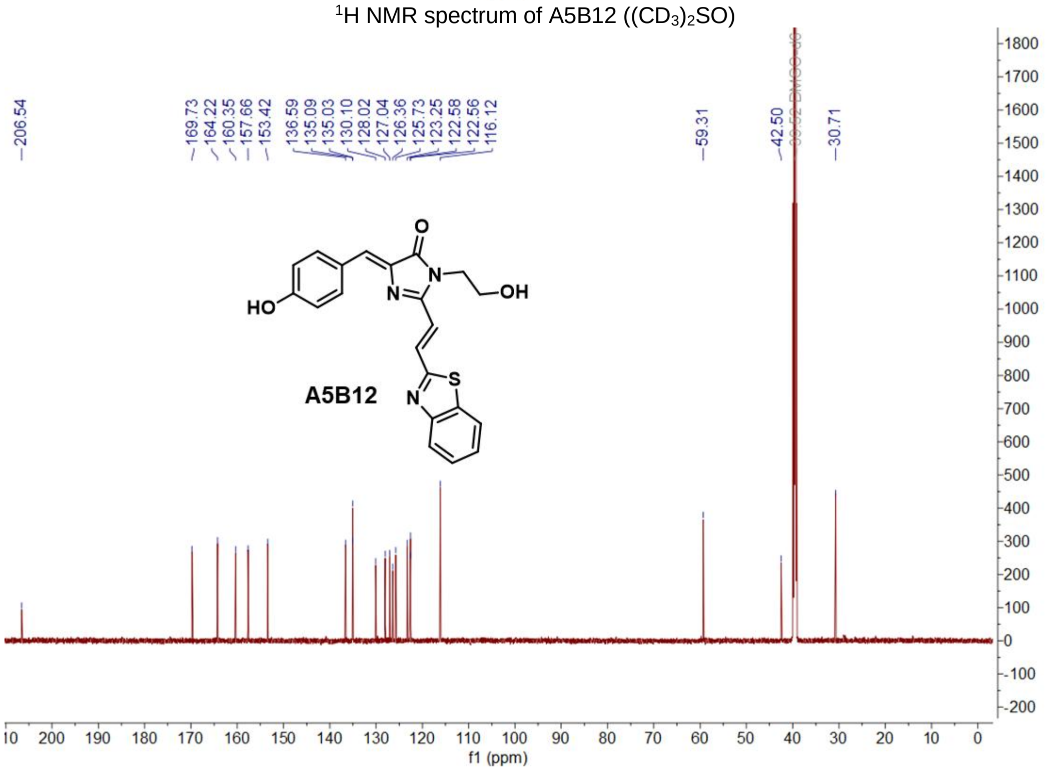

^{13}C NMR spectrum of A5B12 ($(CD_3)_2SO$)

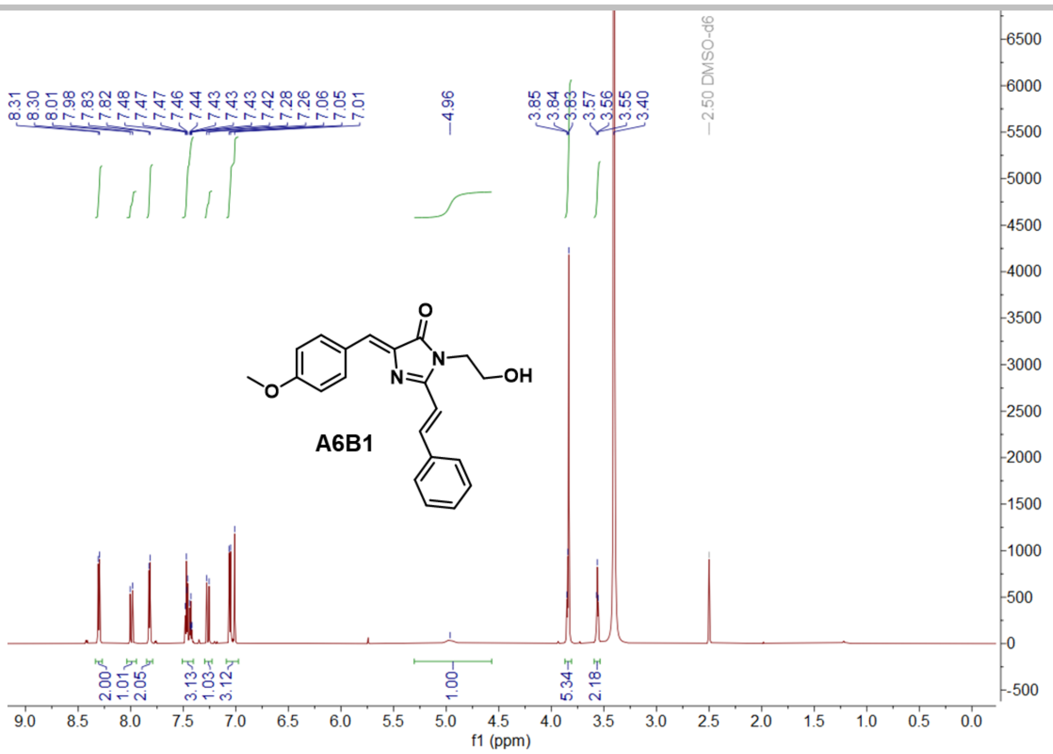

^{1}H NMR spectrum of A6B1 ($(CD_3)_2SO$)

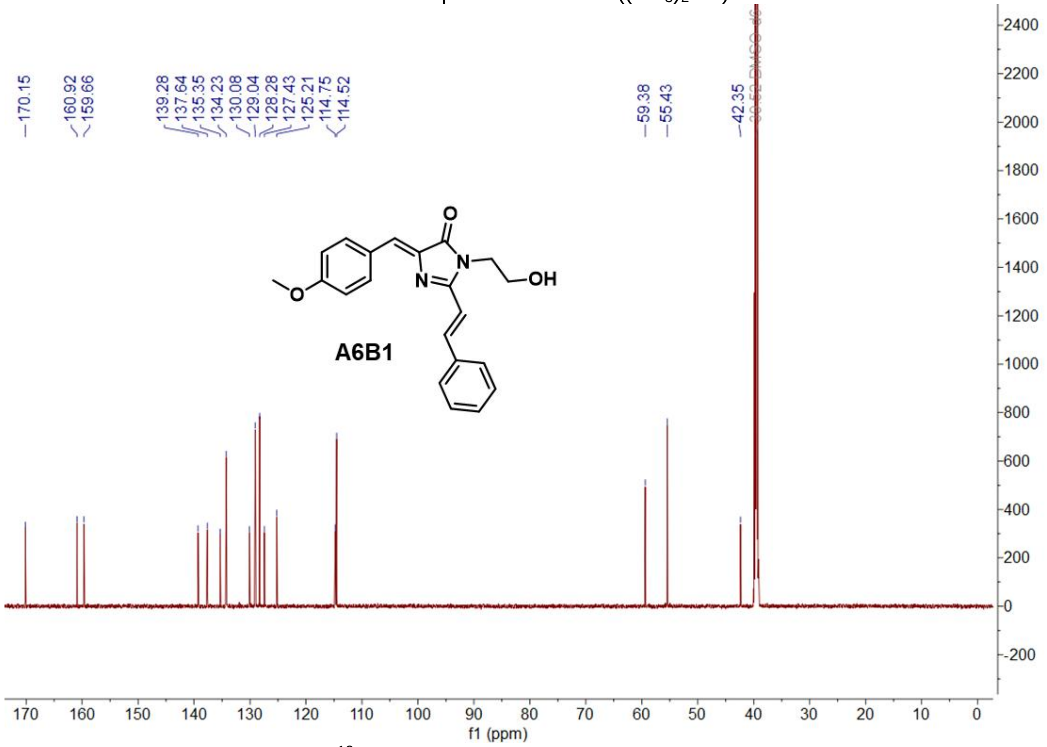

^{13}C NMR spectrum of A6B1 ($(CD_3)_2SO$)

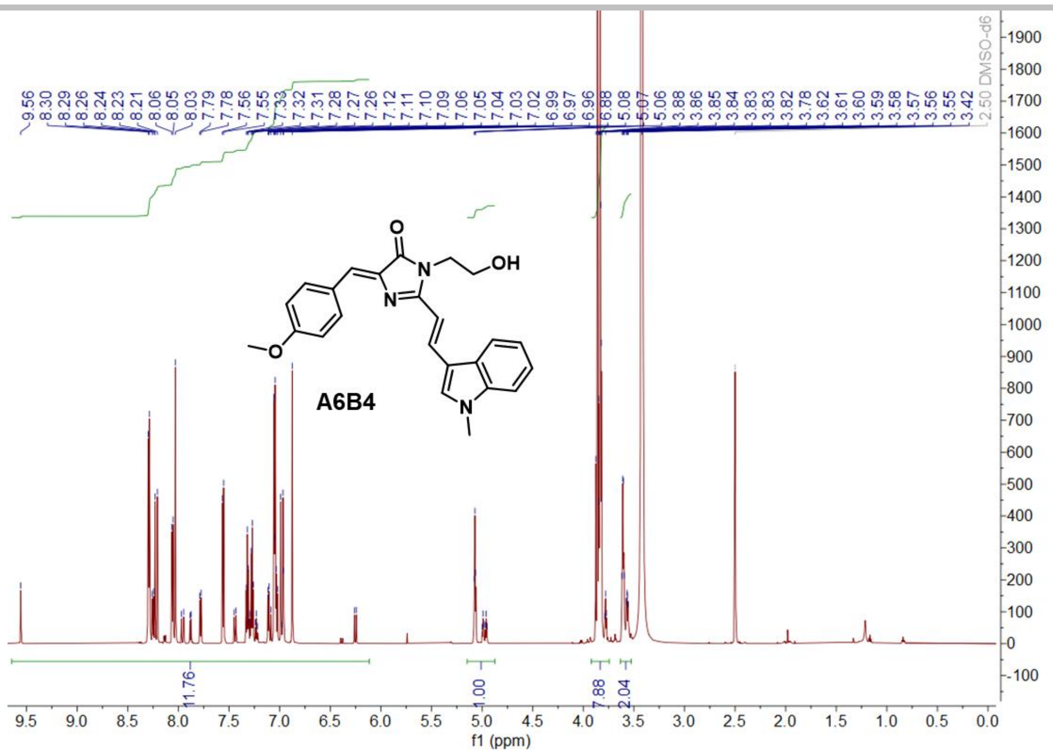

^{1}H NMR spectrum of A6B4 ($(CD_3)_2SO$)

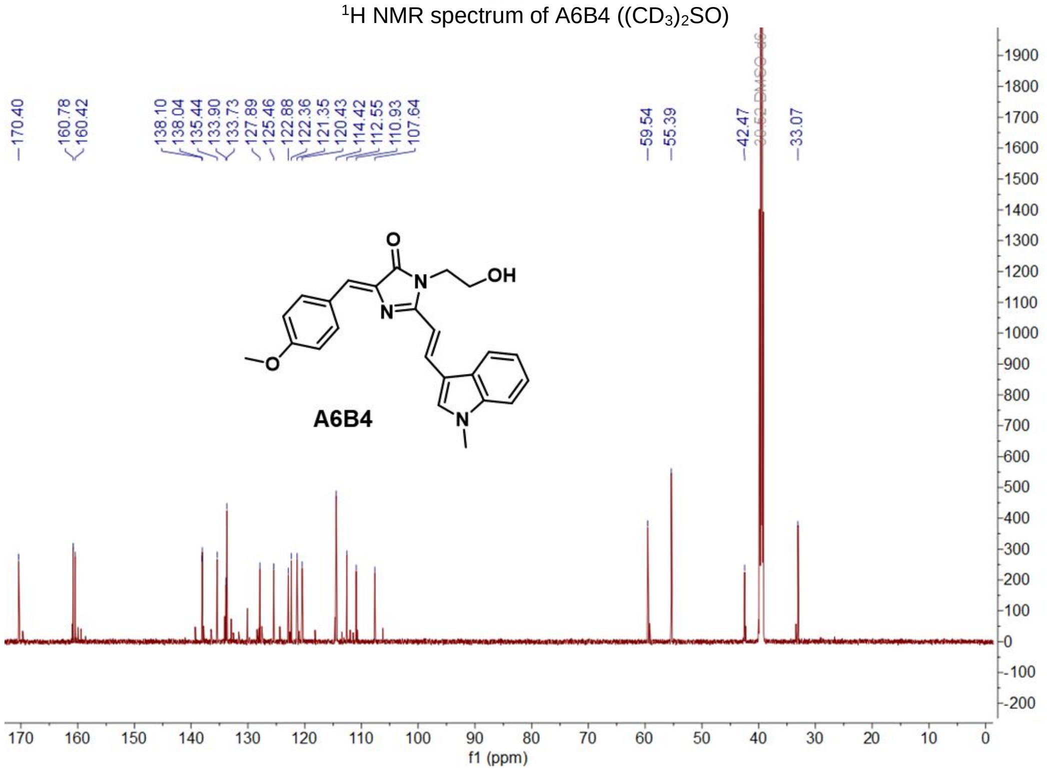

^{13}C NMR spectrum of A6B4 ($(CD_3)_2SO$)

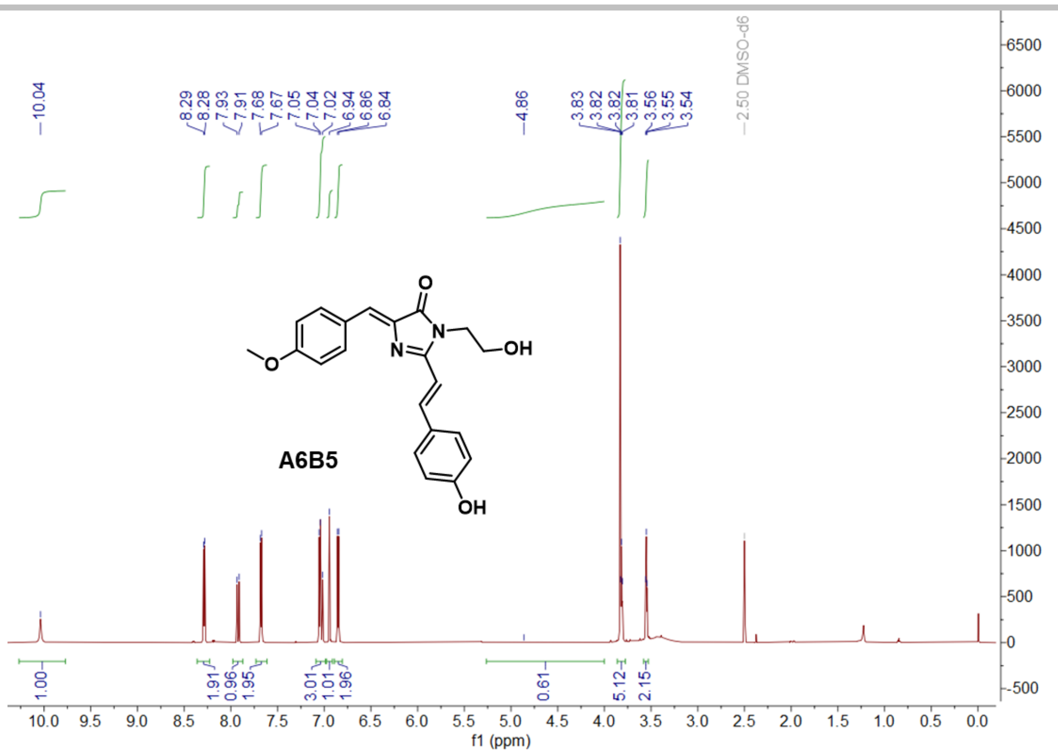

^{1}H NMR spectrum of A6B5 ($(CD_3)_2SO$)

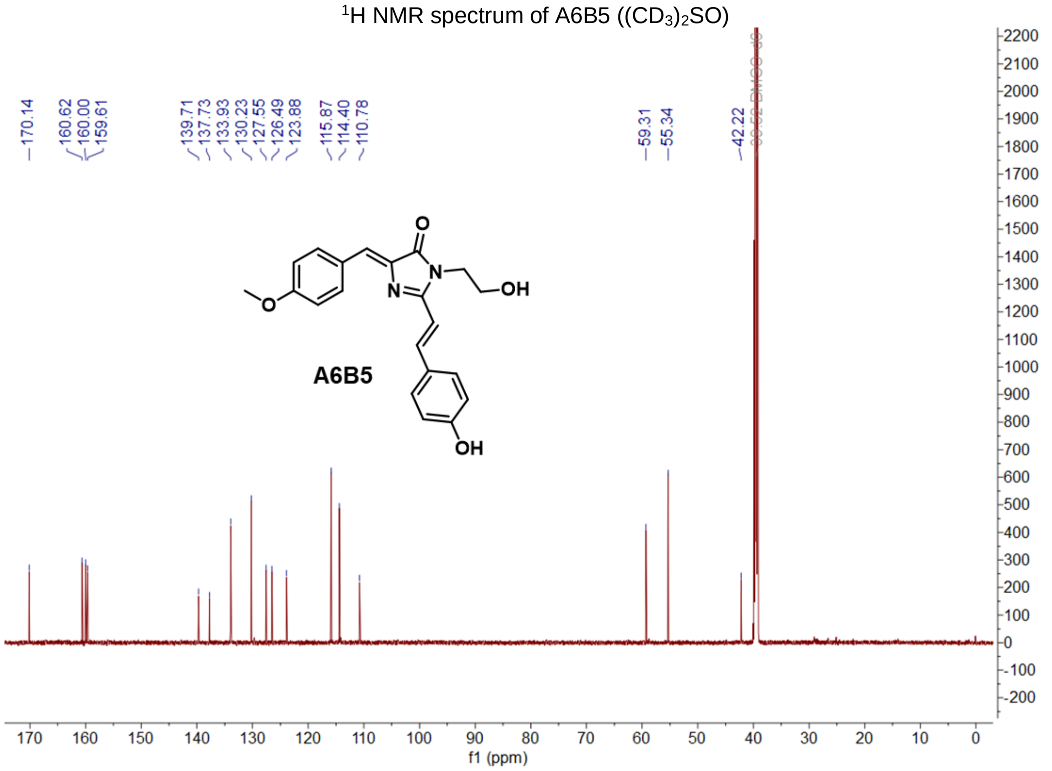

^{13}C NMR spectrum of A6B5 ($(CD_3)_2SO$)

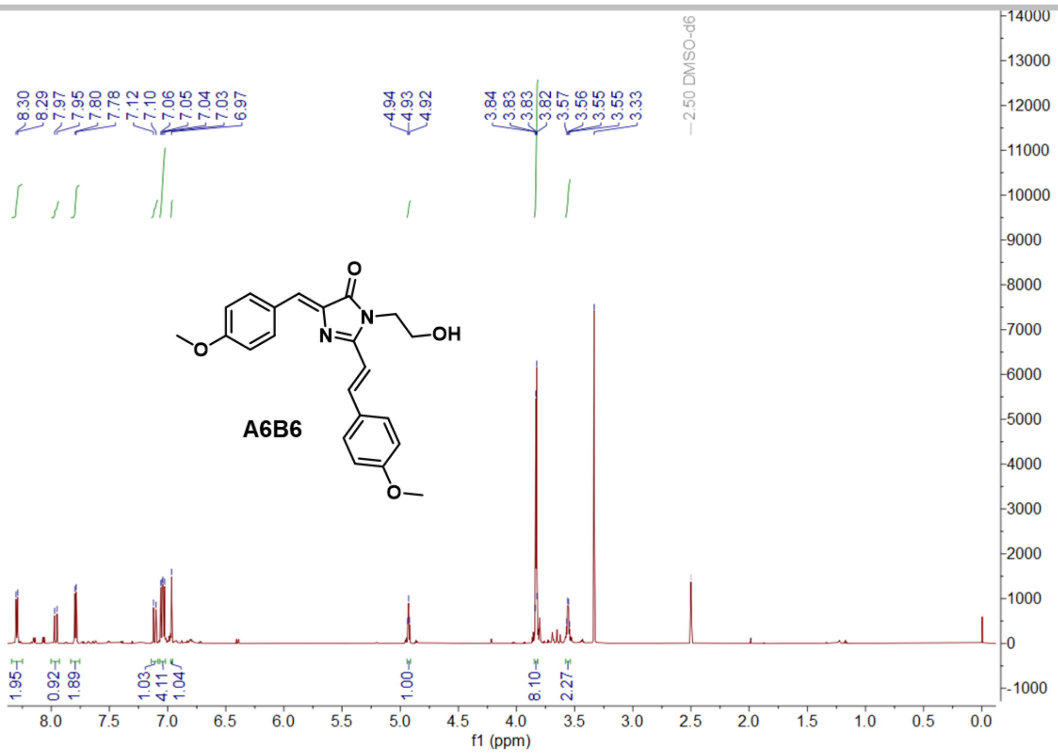

^{1}H NMR spectrum of A6B6 $((CD_3)_2SO)$

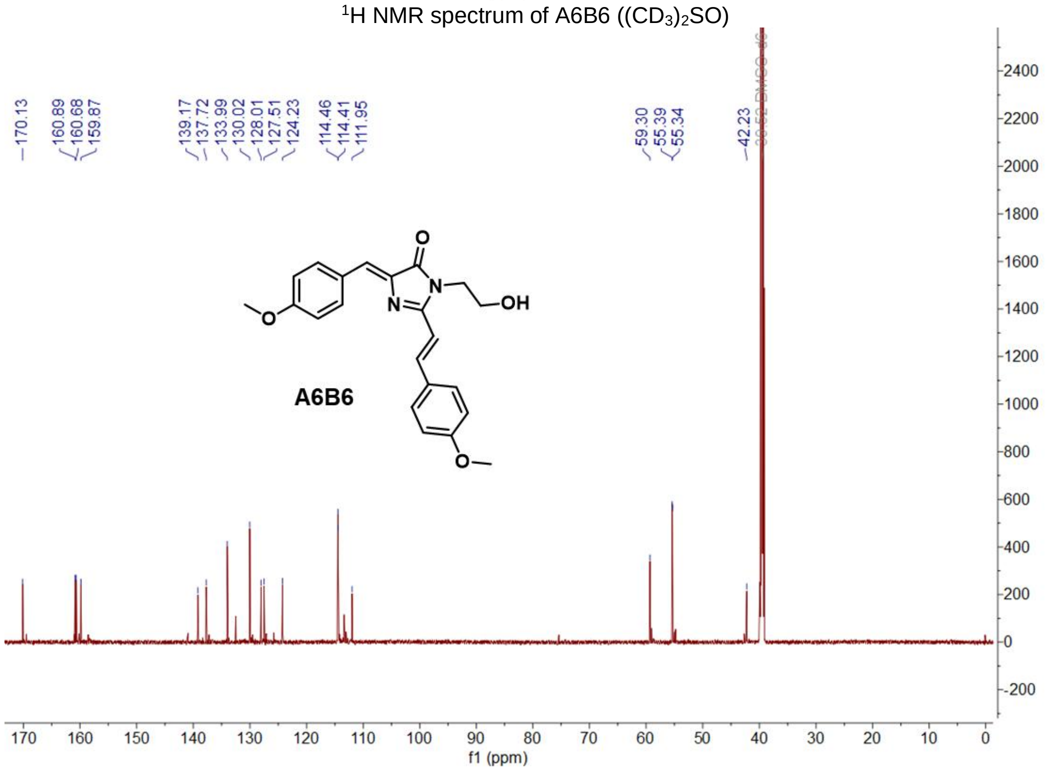

^{13}C NMR spectrum of A6B6 $((CD_3)_2SO)$

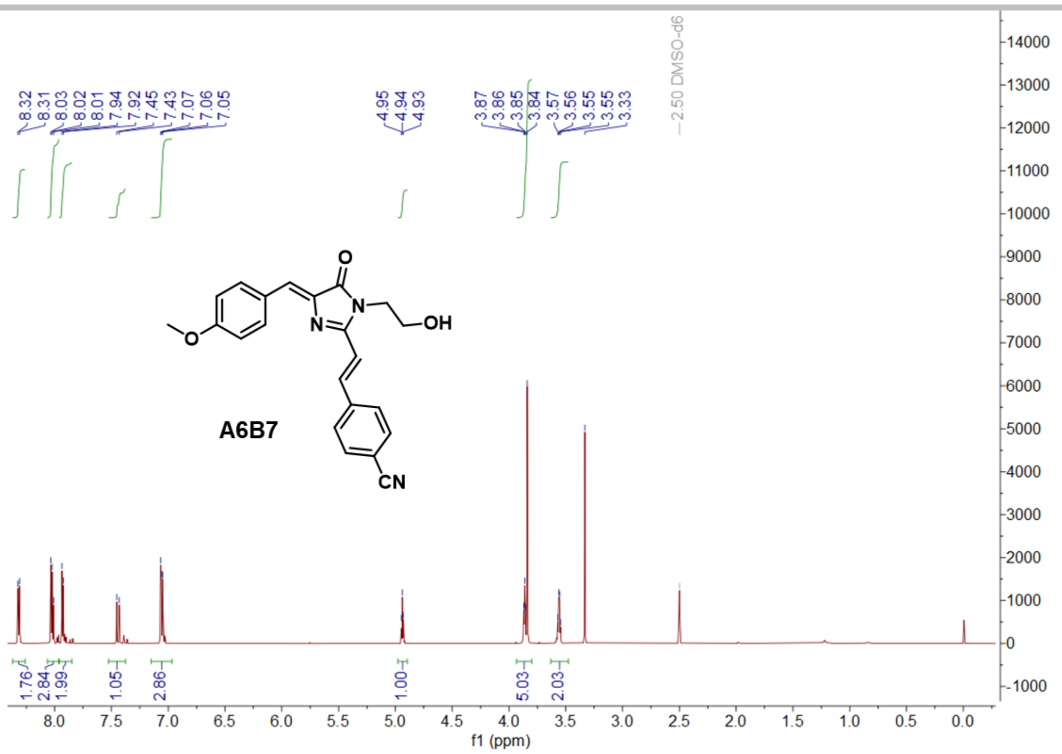

^{1}H NMR spectrum of A6B7 ($(CD_3)_2SO$)

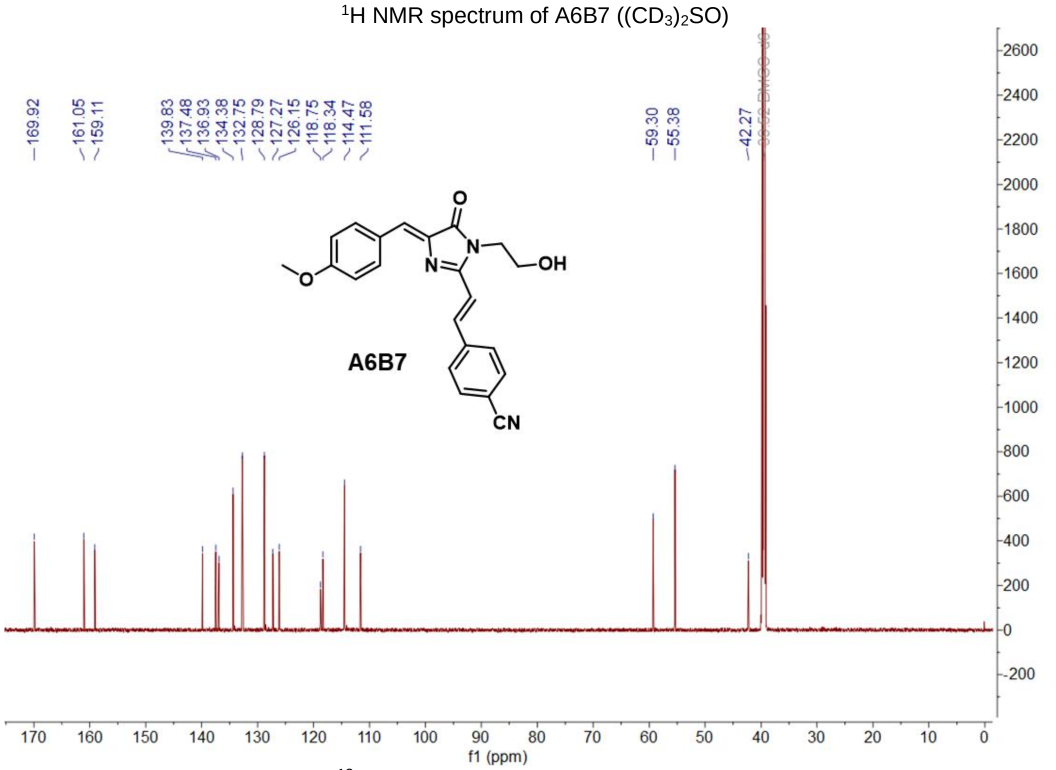

^{13}C NMR spectrum of A6B7 ($(CD_3)_2SO$)

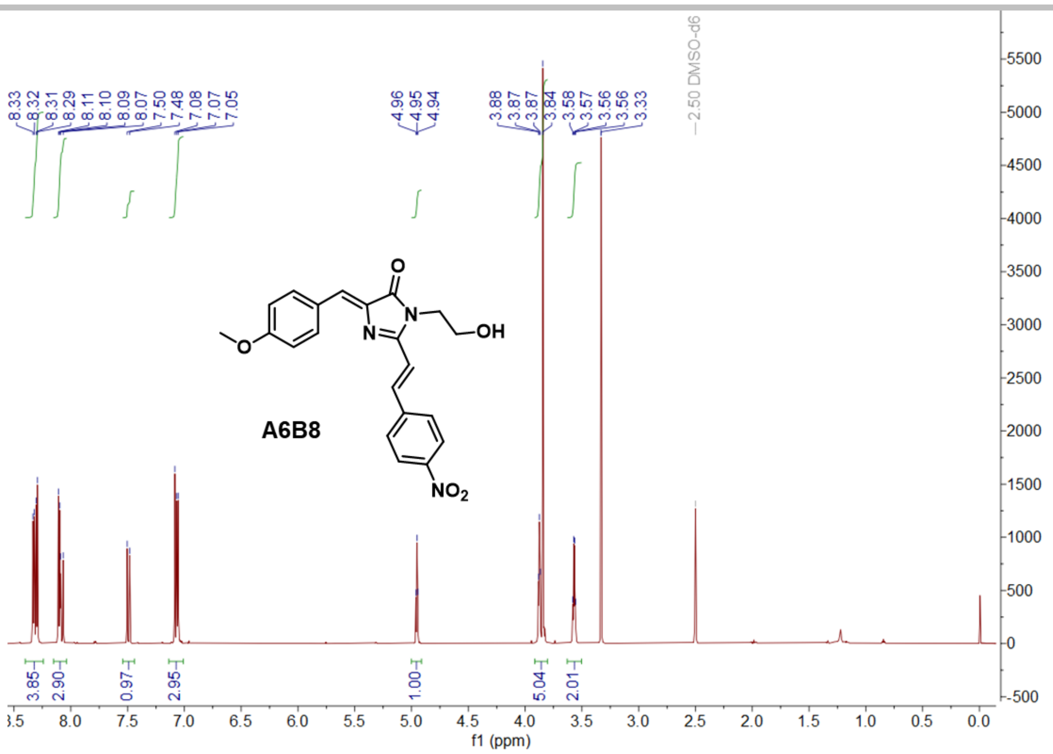

^{1}H NMR spectrum of A6B8 ($(CD_3)_2SO$)

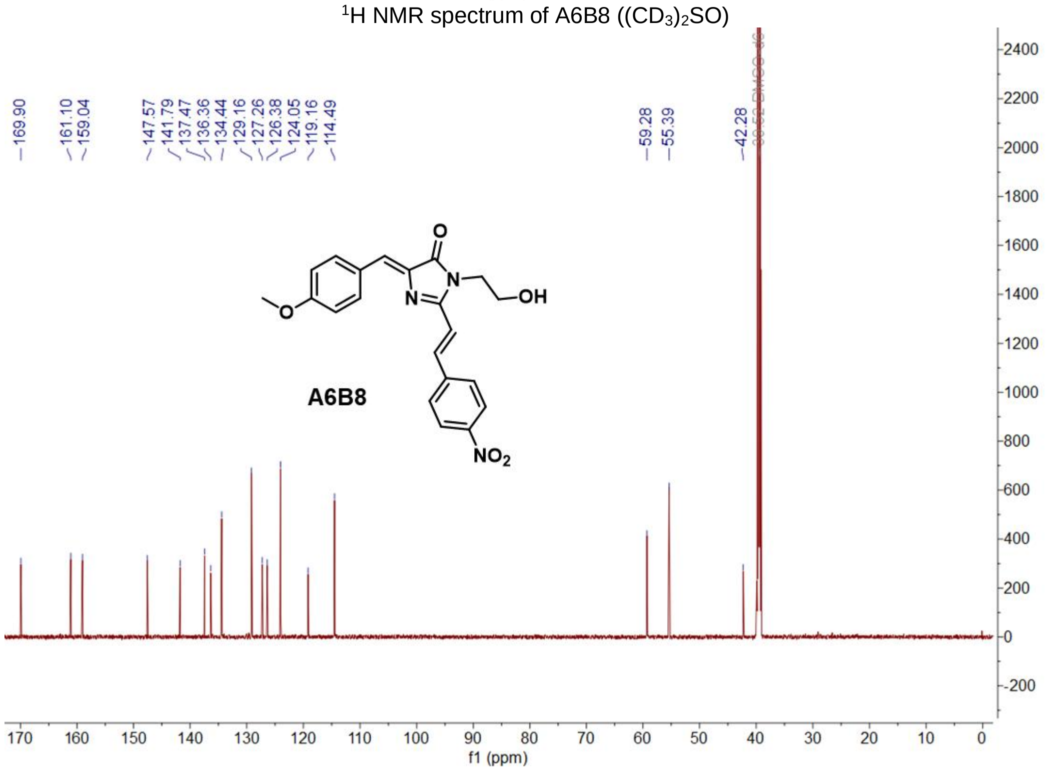

^{13}C NMR spectrum of A6B8 ($(CD_3)_2SO$)

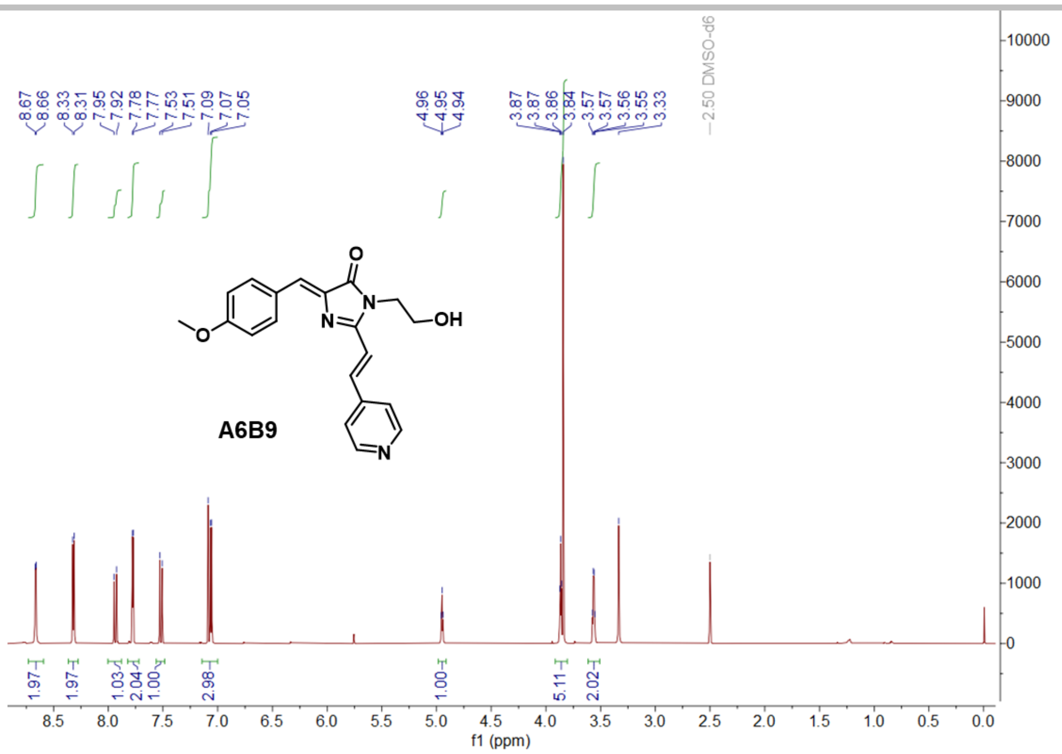

^1H NMR spectrum of A6B9 ($(CD_3)_2SO$)

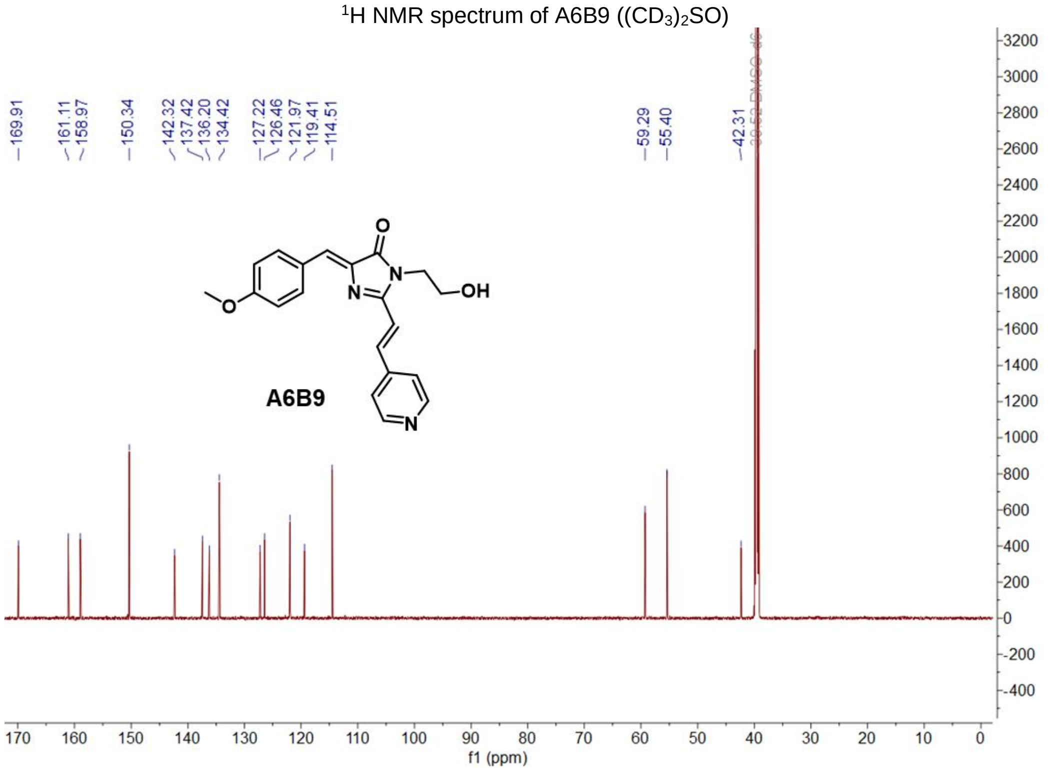

^{13}C NMR spectrum of A6B9 ($(CD_3)_2SO$)

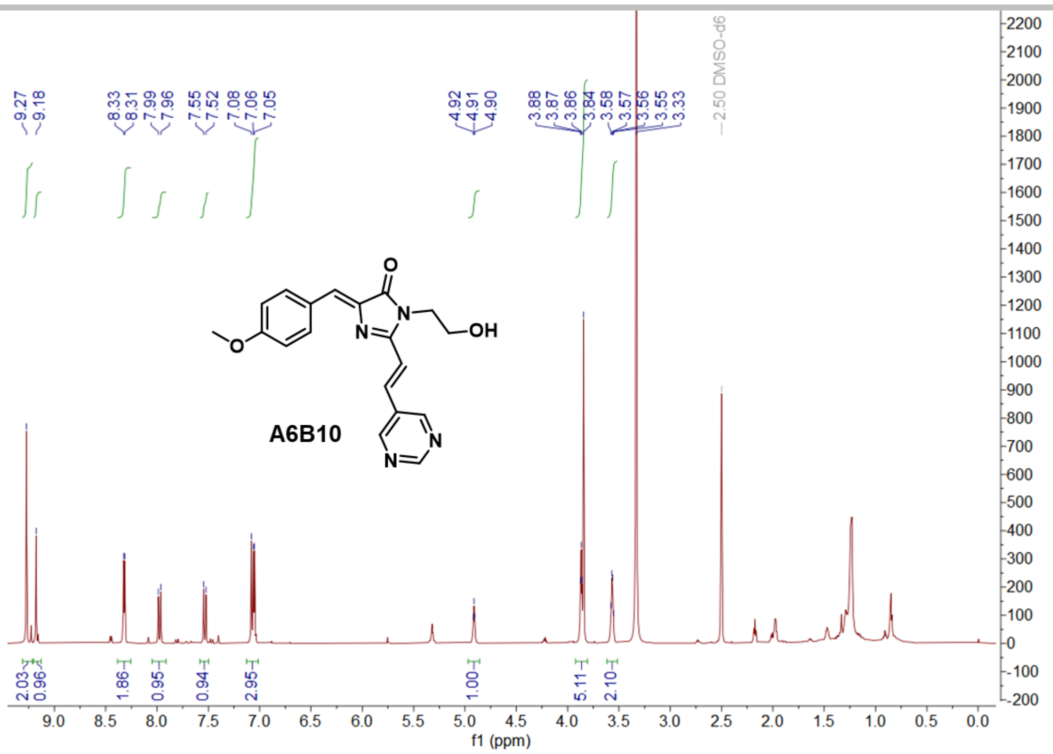

¹H NMR spectrum of A6B10 ((CD$_3$)$_2$SO)

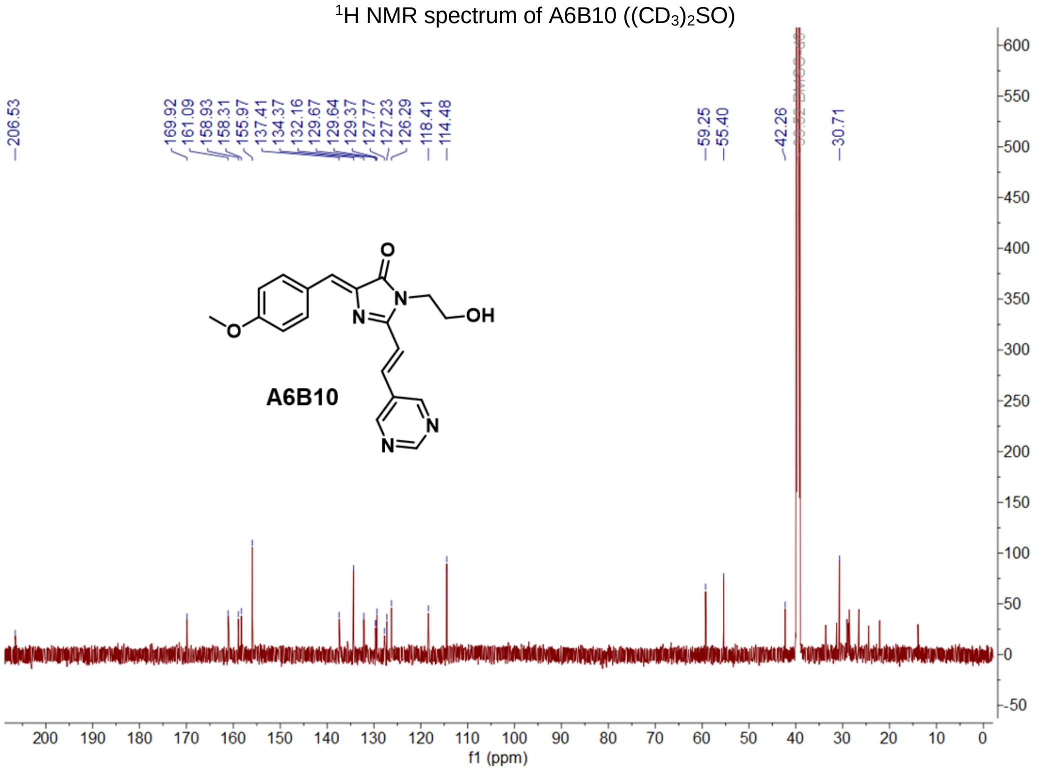

¹³C NMR spectrum of A6B10 ((CD$_3$)$_2$SO)

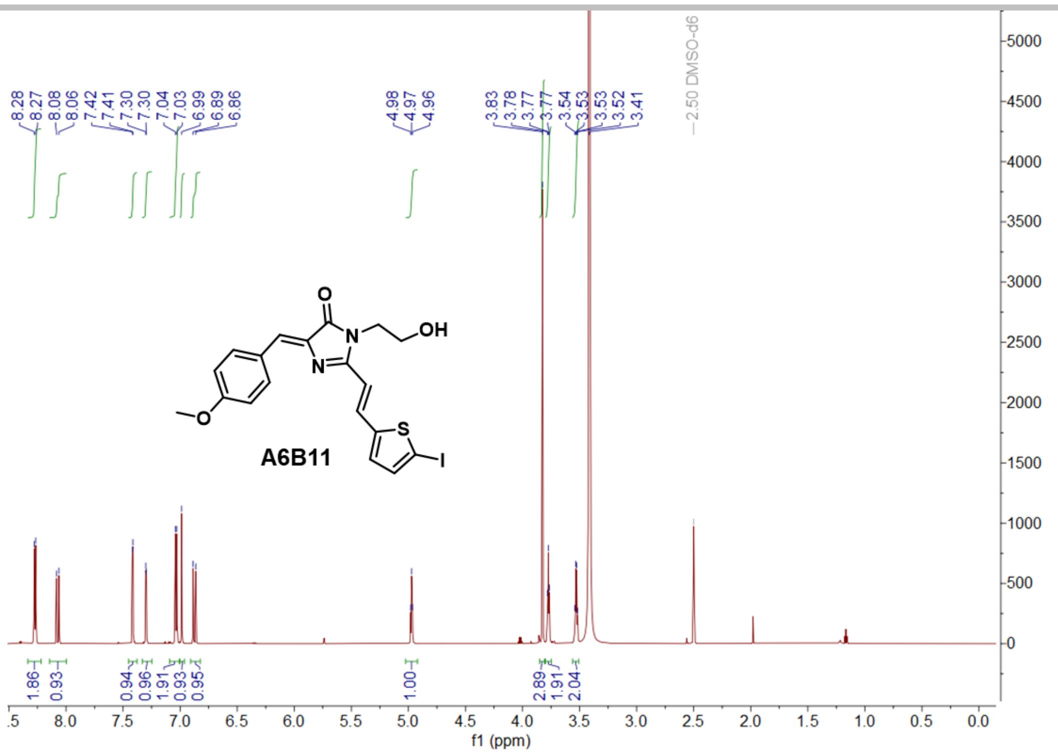

^{1}H NMR spectrum of A6B11 ($(CD_3)_2SO$)

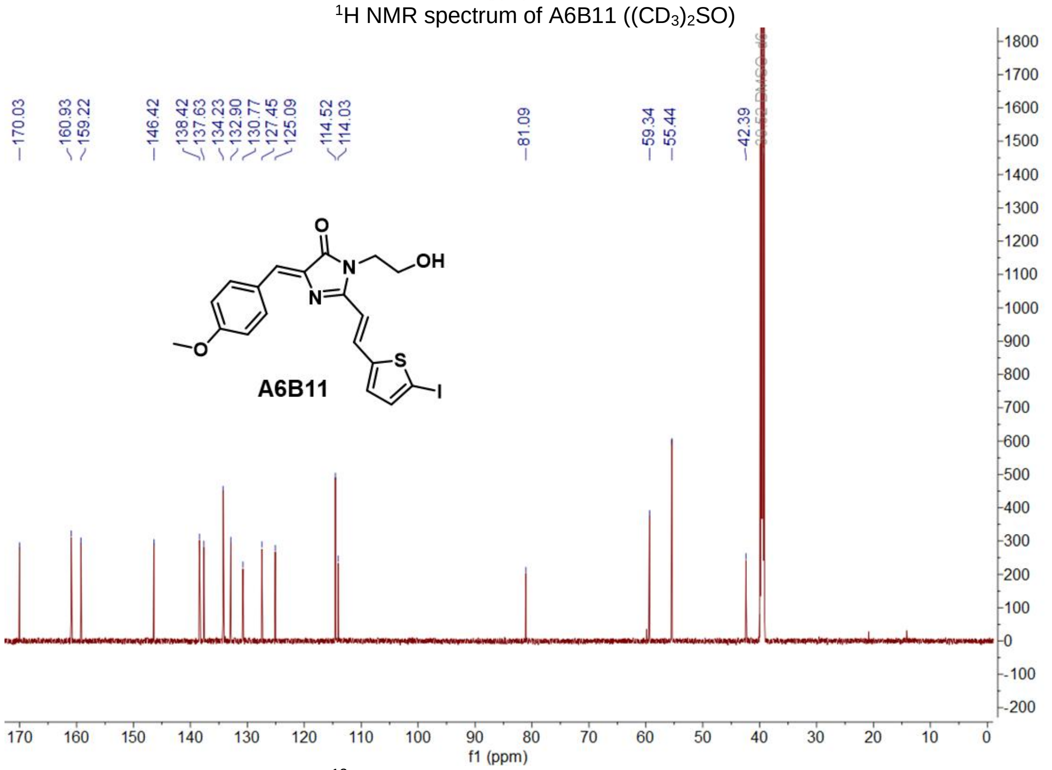

^{13}C NMR spectrum of A6B11 ($(CD_3)_2SO$)

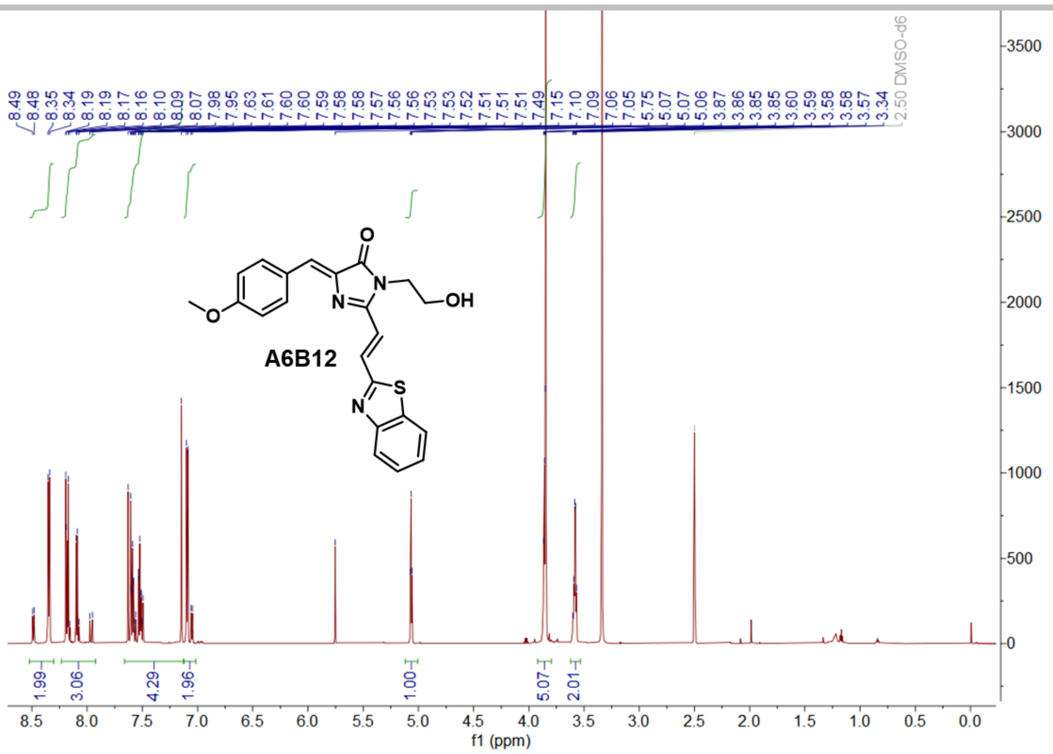

^1H NMR spectrum of A6B12 ($(CD_3)_2SO$)

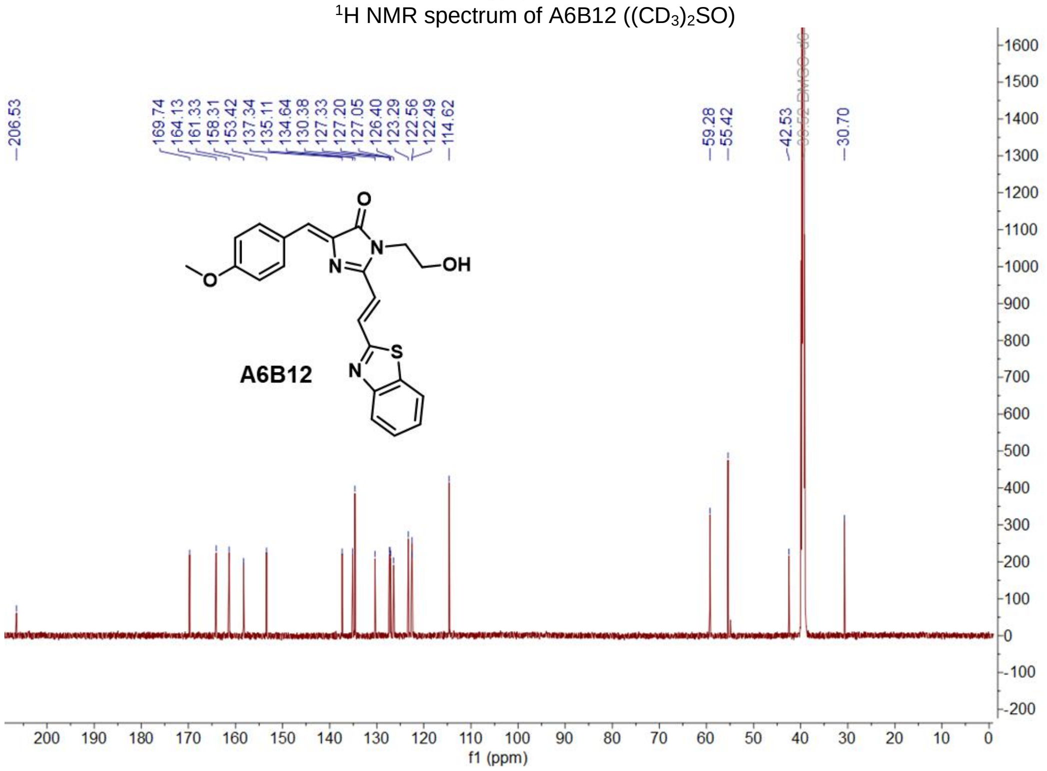

^{13}C NMR spectrum of A6B12 ($(CD_3)_2SO$)

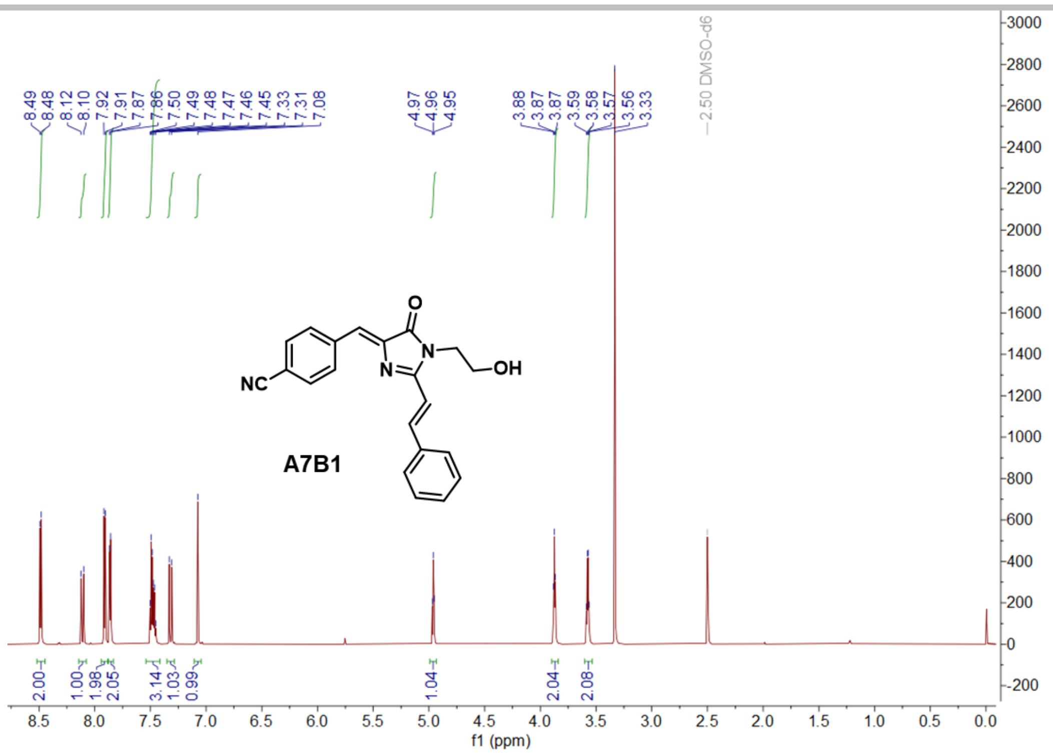

^{1}H NMR spectrum of A7B1 ($(CD_3)_2SO$)

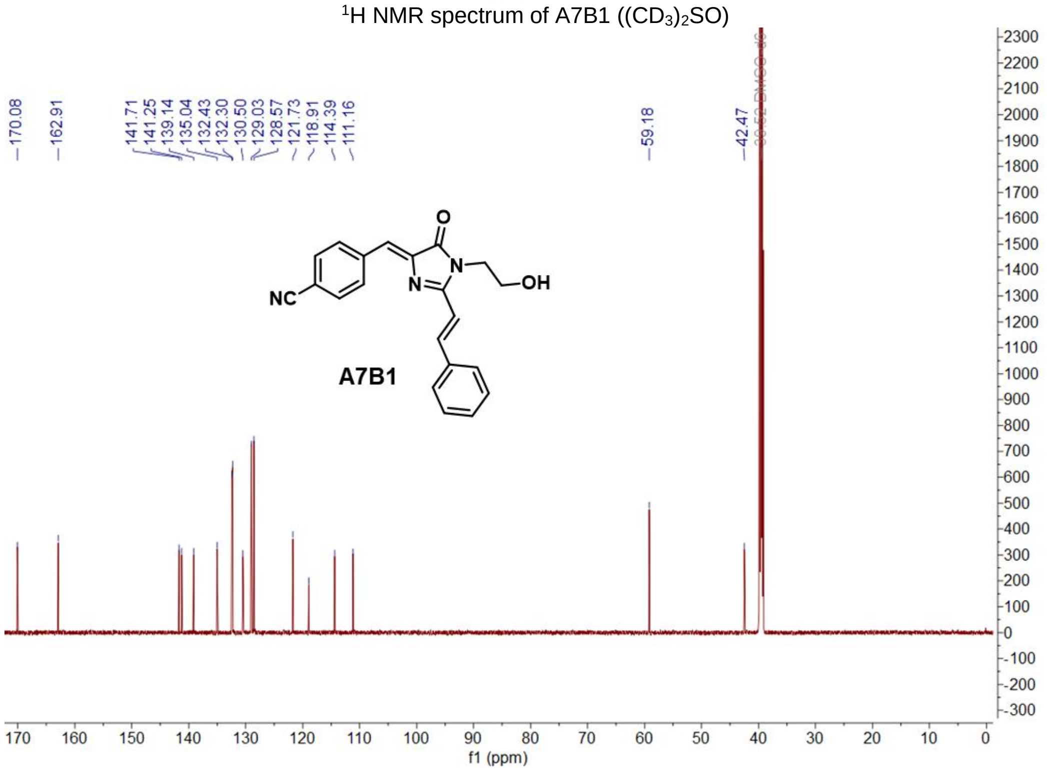

^{13}C NMR spectrum of A7B1 ($(CD_3)_2SO$)

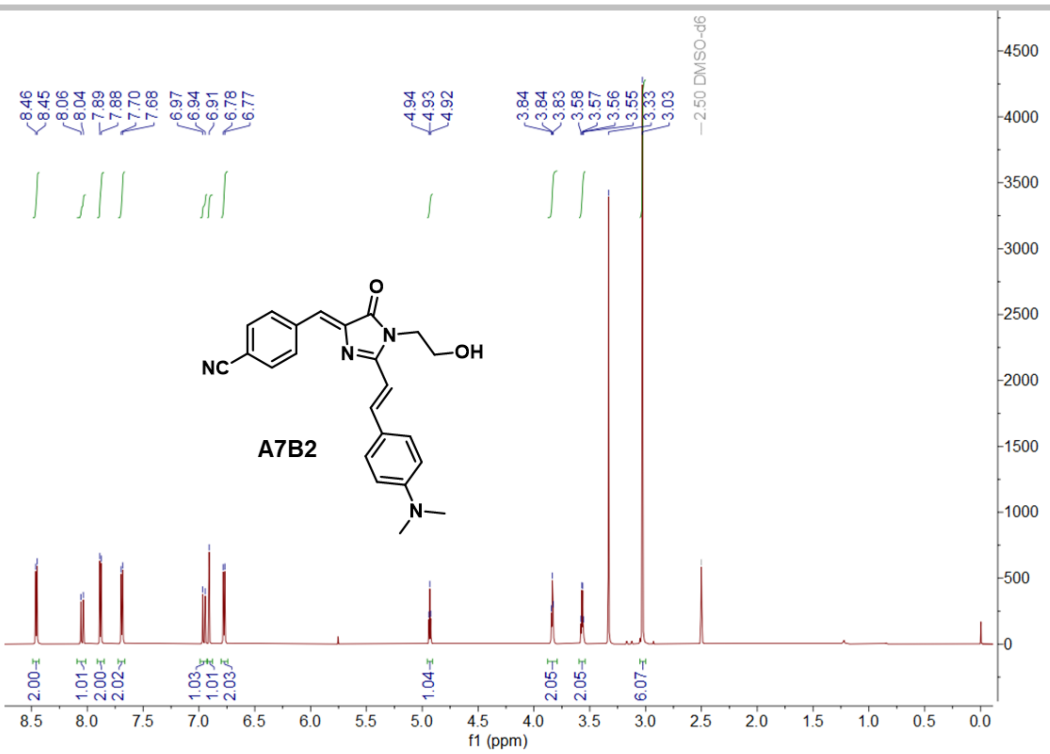

^{1}H NMR spectrum of A7B2 (($(CD_3)_2SO$))

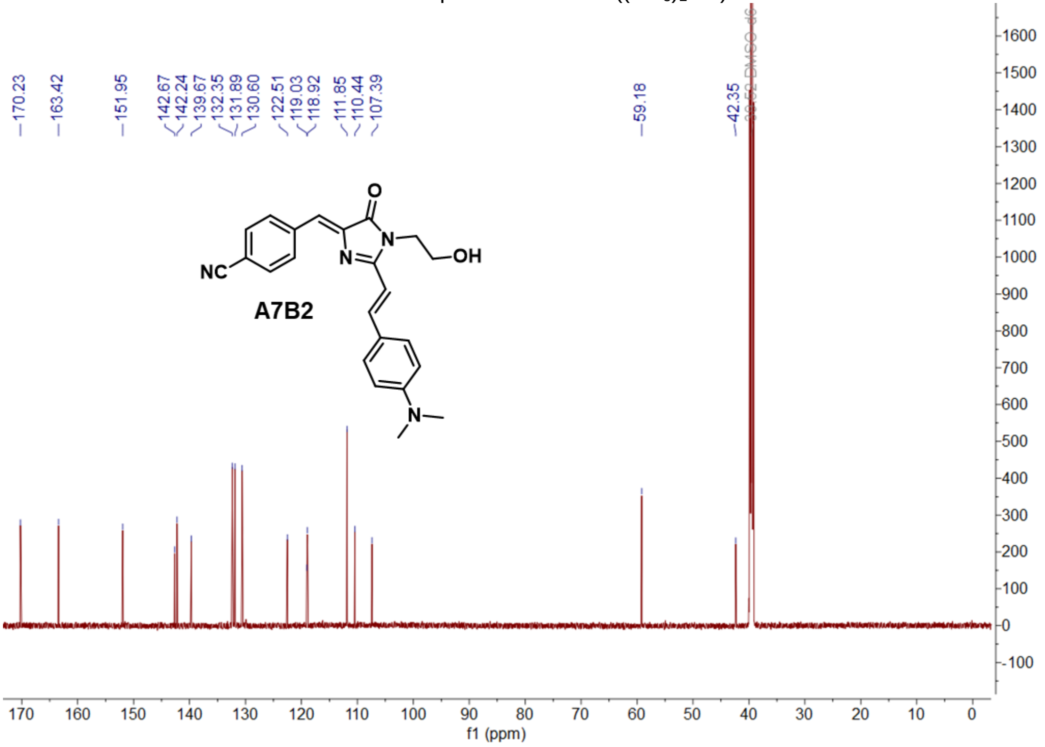

^{13}C NMR spectrum of A7B2 (($(CD_3)_2SO$))

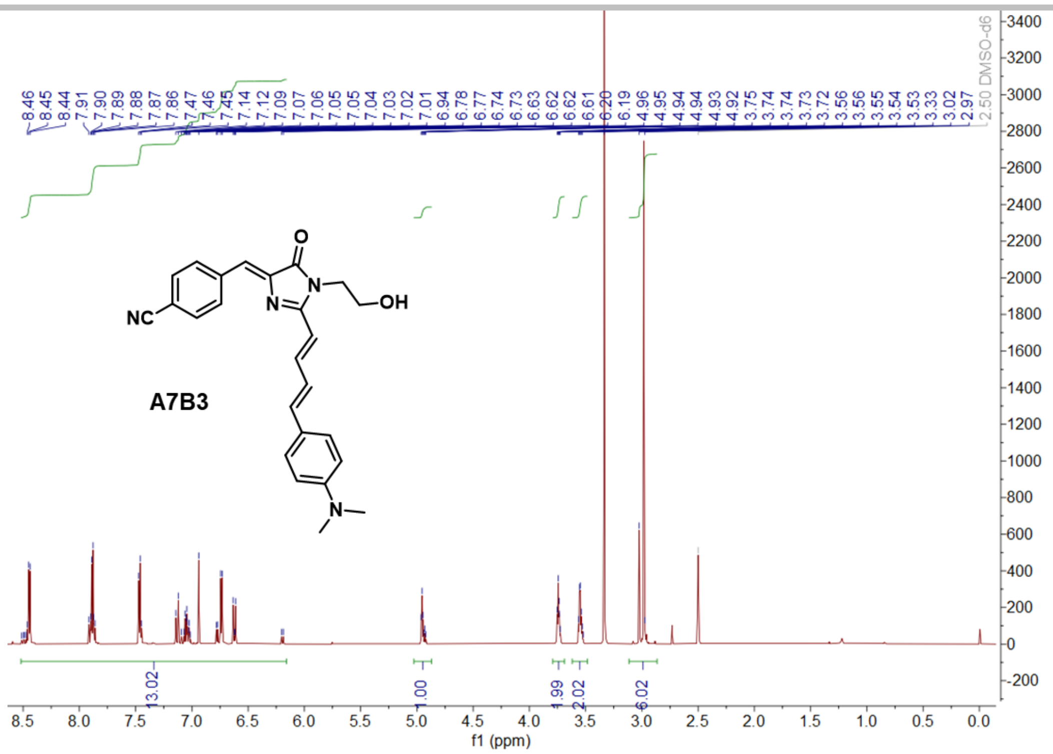

^{1}H NMR spectrum of A7B3 (($(CD_3)_2SO$)

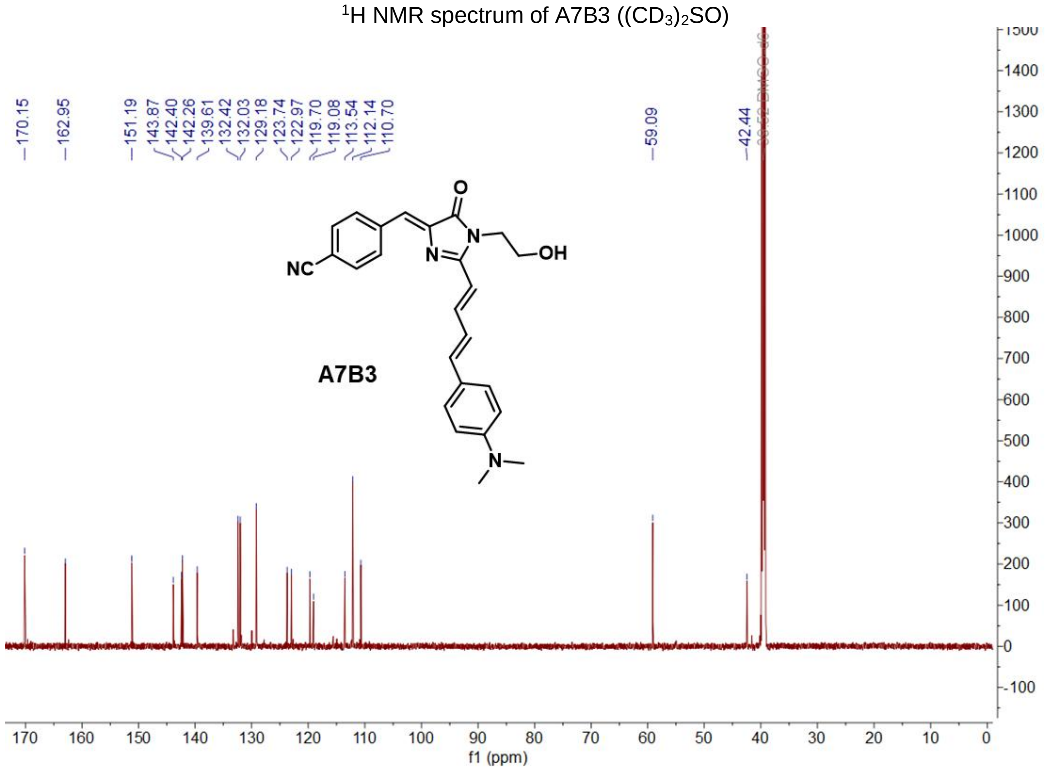

^{13}C NMR spectrum of A7B3 (($(CD_3)_2SO$)

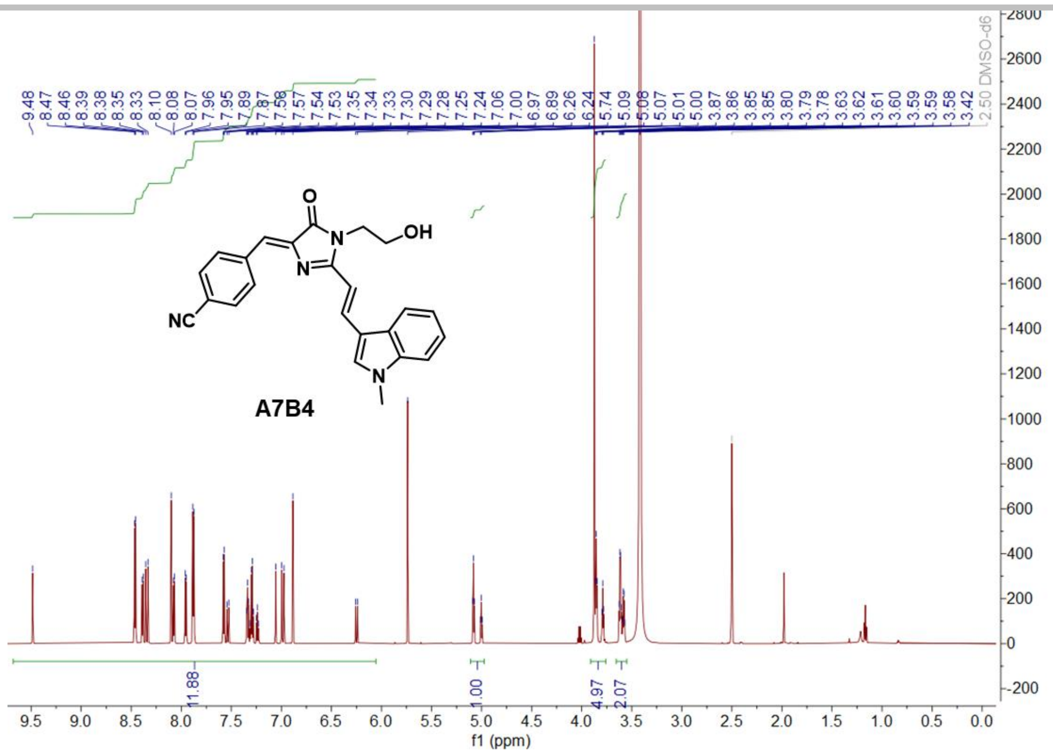

^{1}H NMR spectrum of A7B4 ($(CD_3)_2SO$)

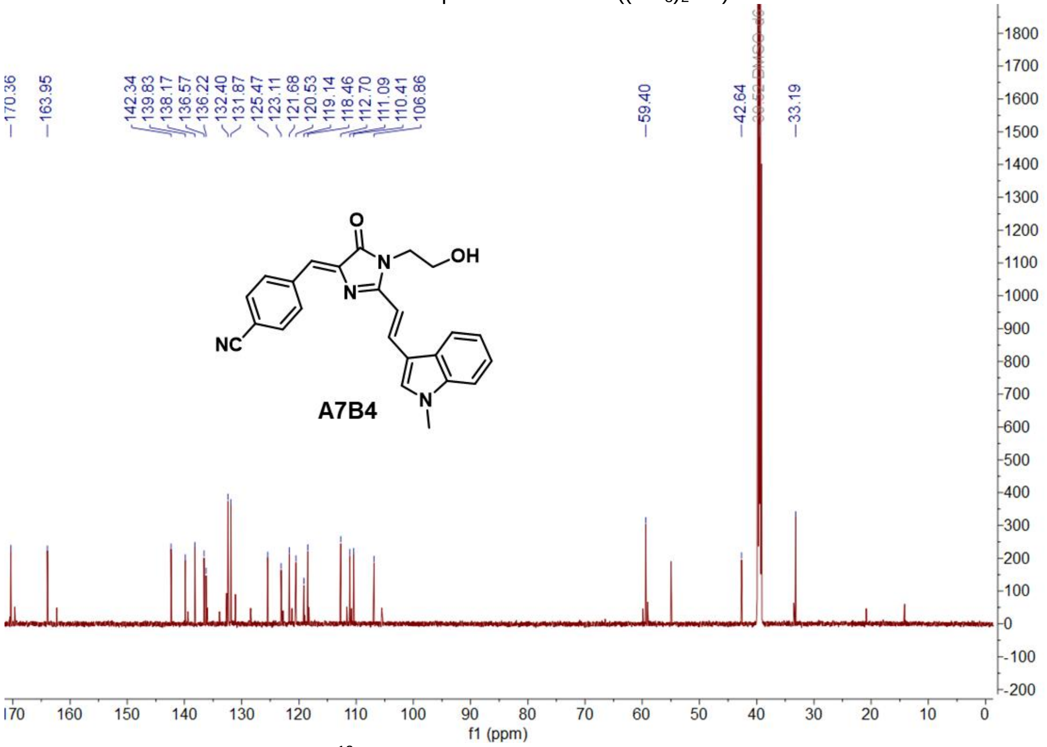

^{13}C NMR spectrum of A7B4 ($(CD_3)_2SO$)

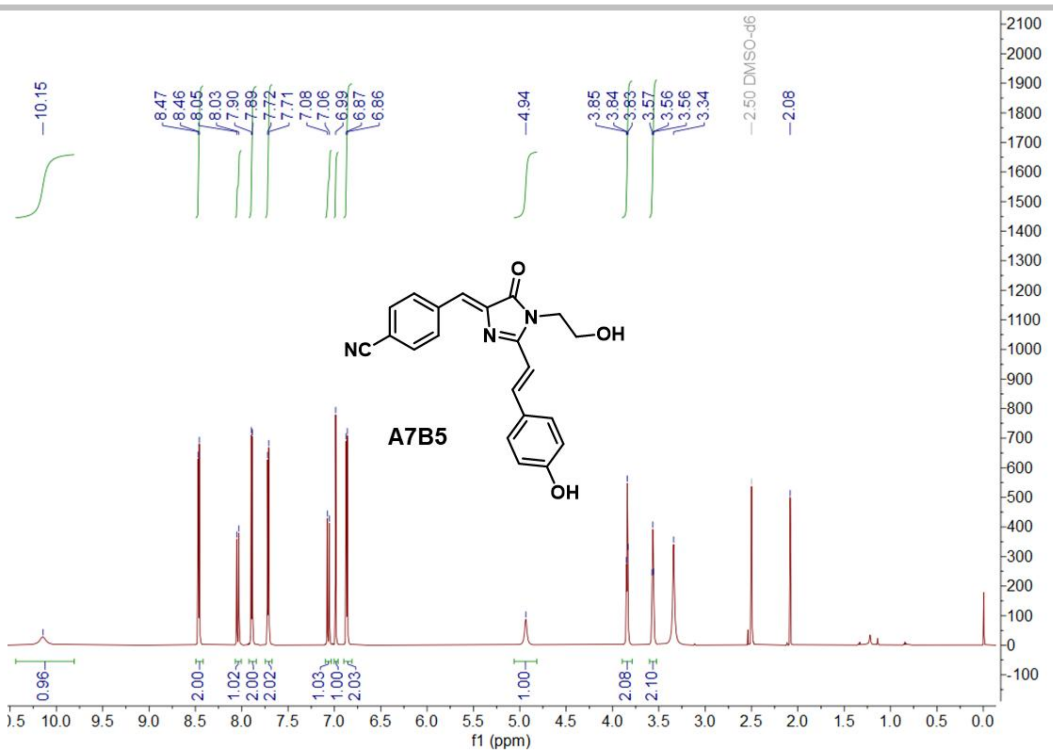

^{1}H NMR spectrum of A7B5 ($(CD_3)_2SO$)

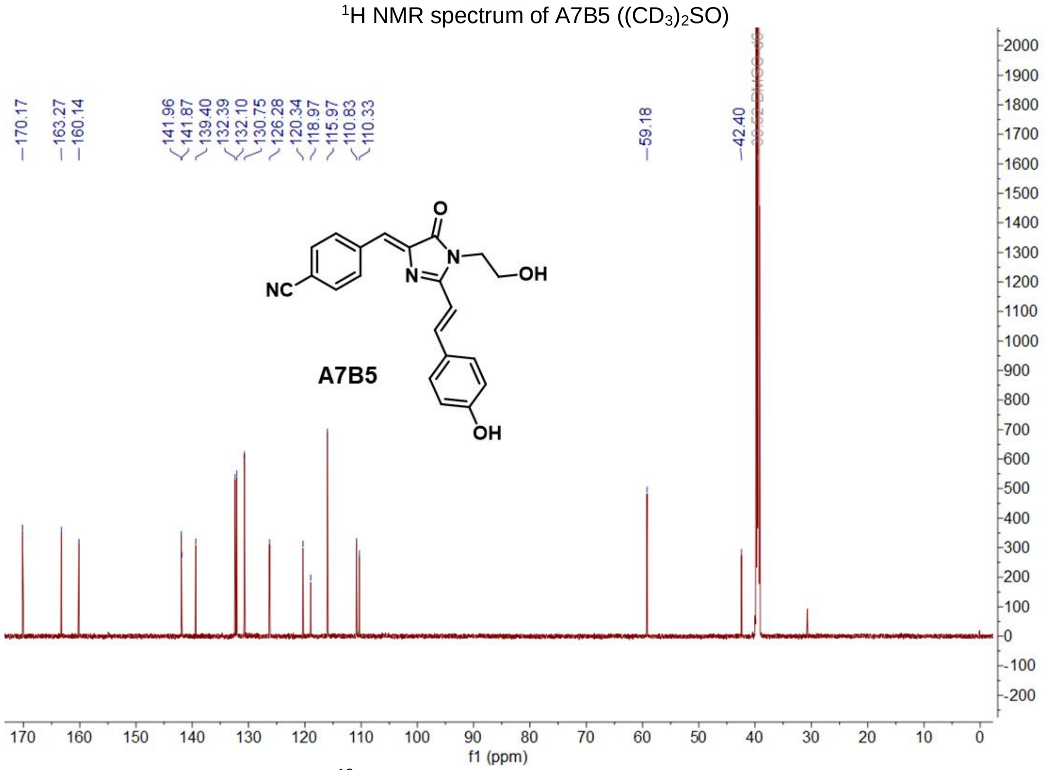

^{13}C NMR spectrum of A7B5 ($(CD_3)_2SO$)

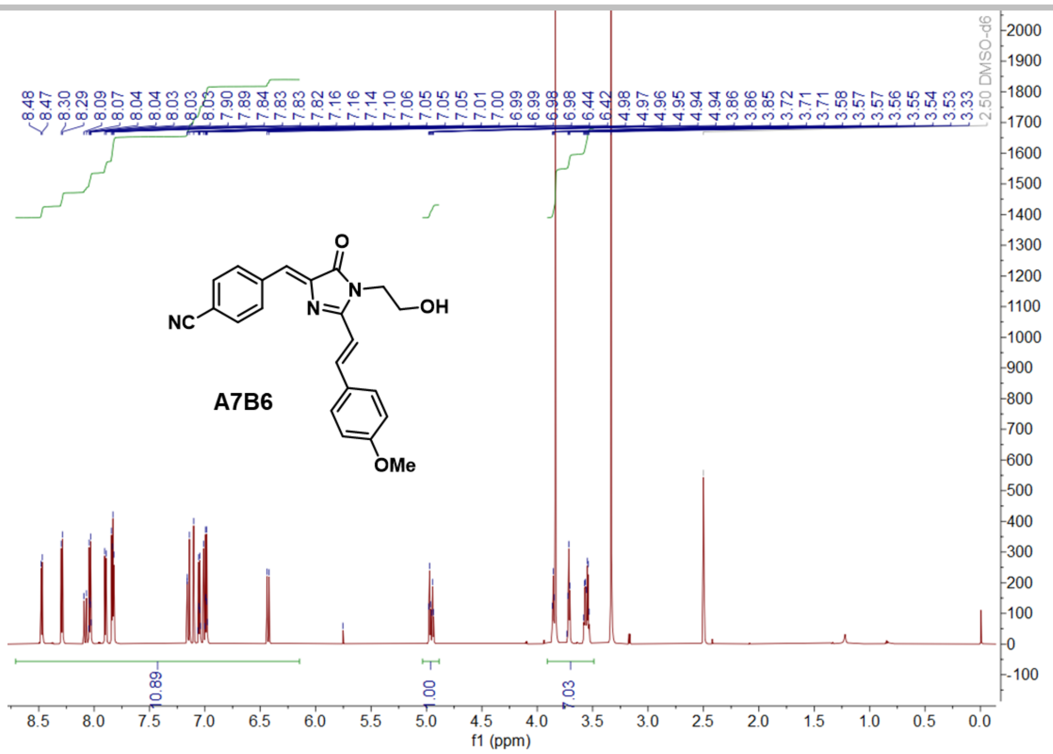

^{1}H NMR spectrum of A7B6 ($(CD_3)_2SO$)

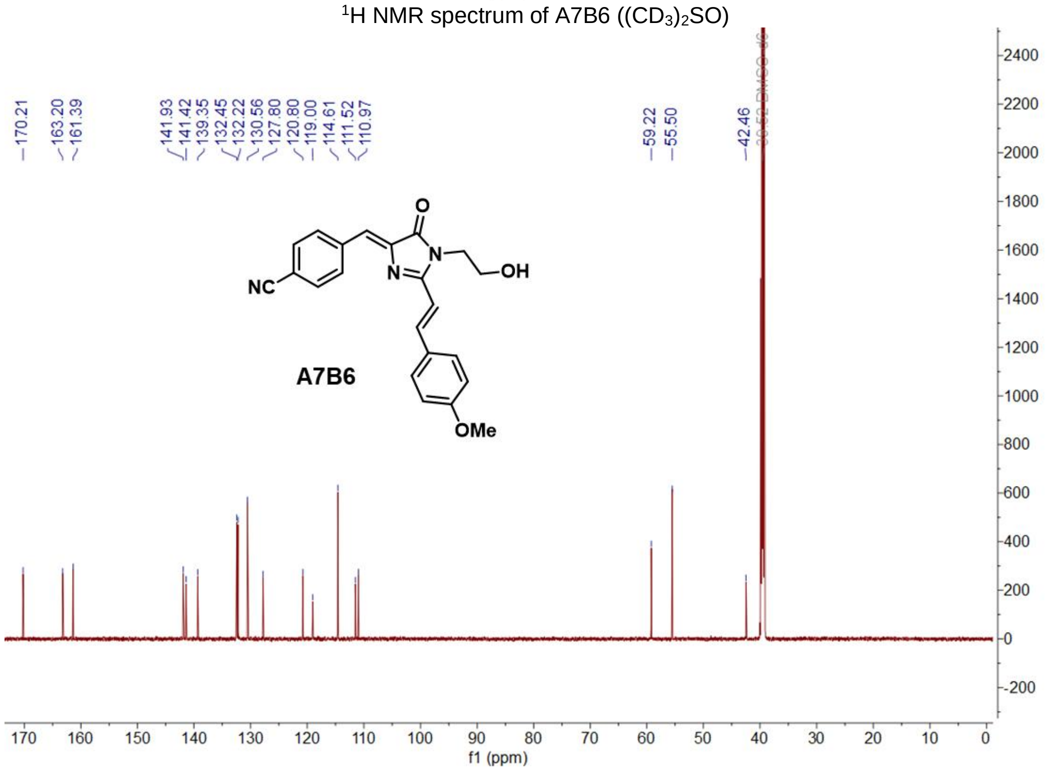

^{13}C NMR spectrum of A7B6 ($(CD_3)_2SO$)

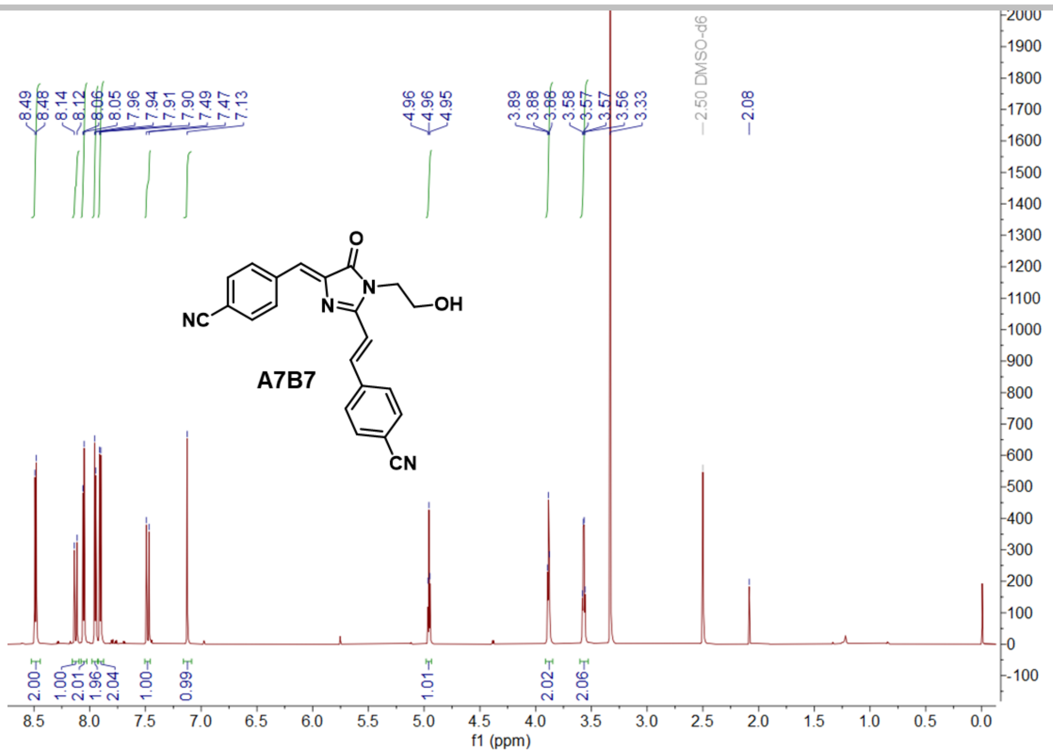

^{1}H NMR spectrum of A7B7 ($(CD_3)_2SO$)

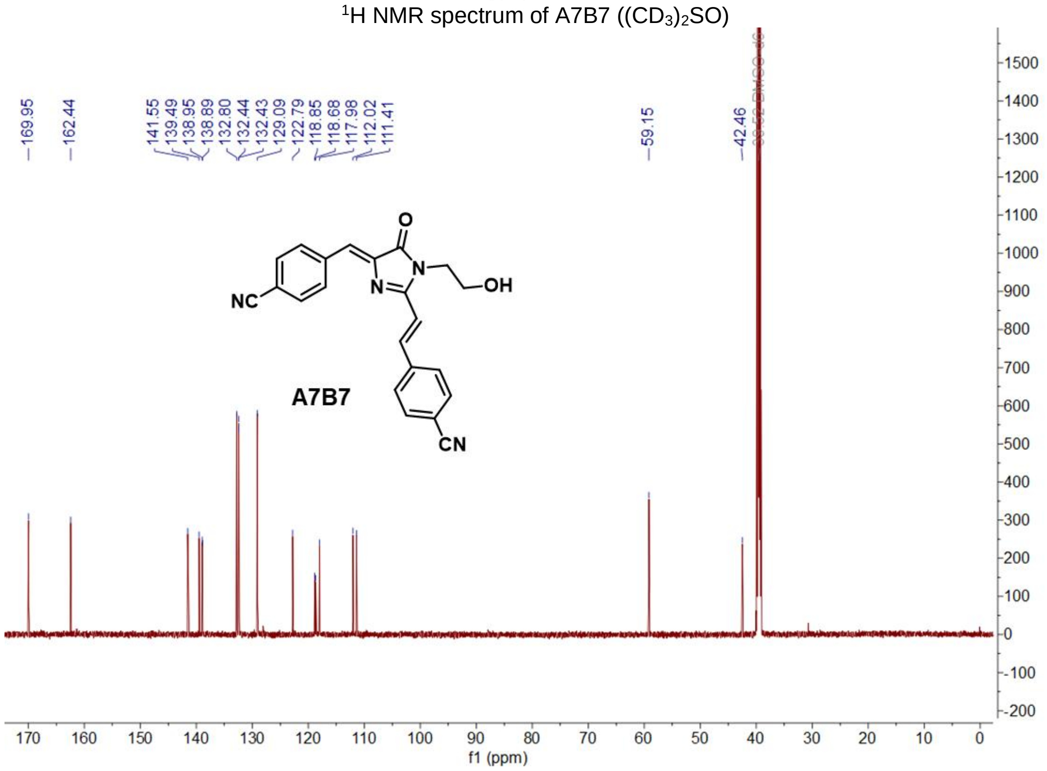

^{13}C NMR spectrum of A7B7 ($(CD_3)_2SO$)

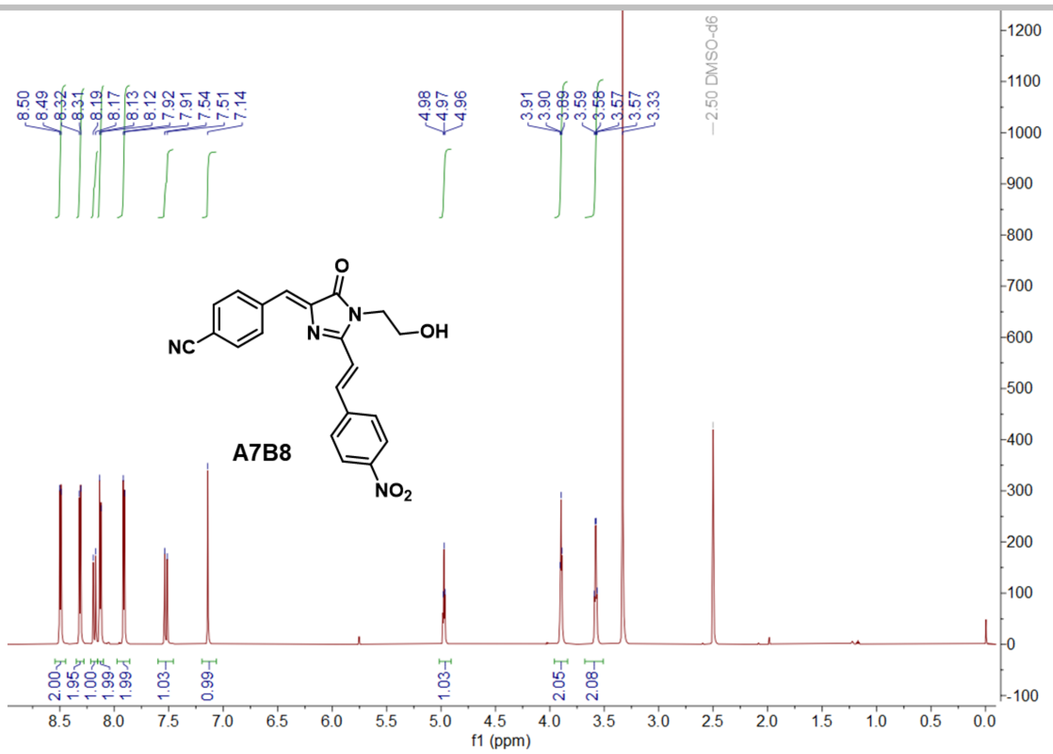

^{1}H NMR spectrum of A7B8 ($(CD_3)_2SO$)

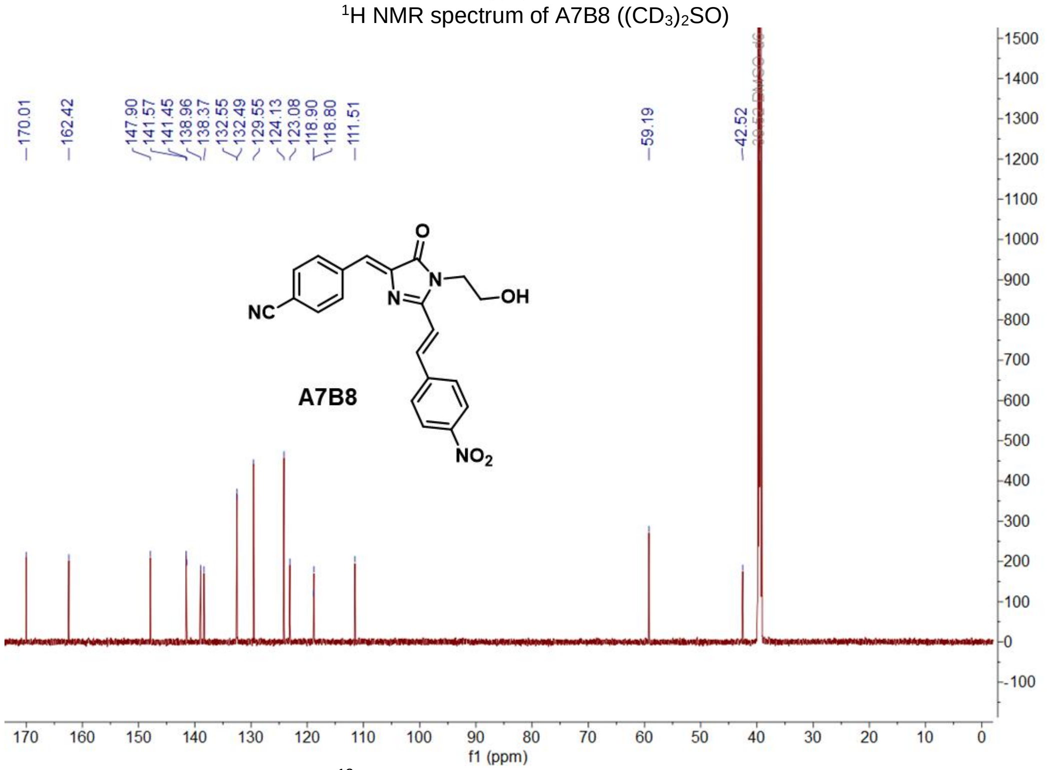

^{13}C NMR spectrum of A7B8 ($(CD_3)_2SO$)

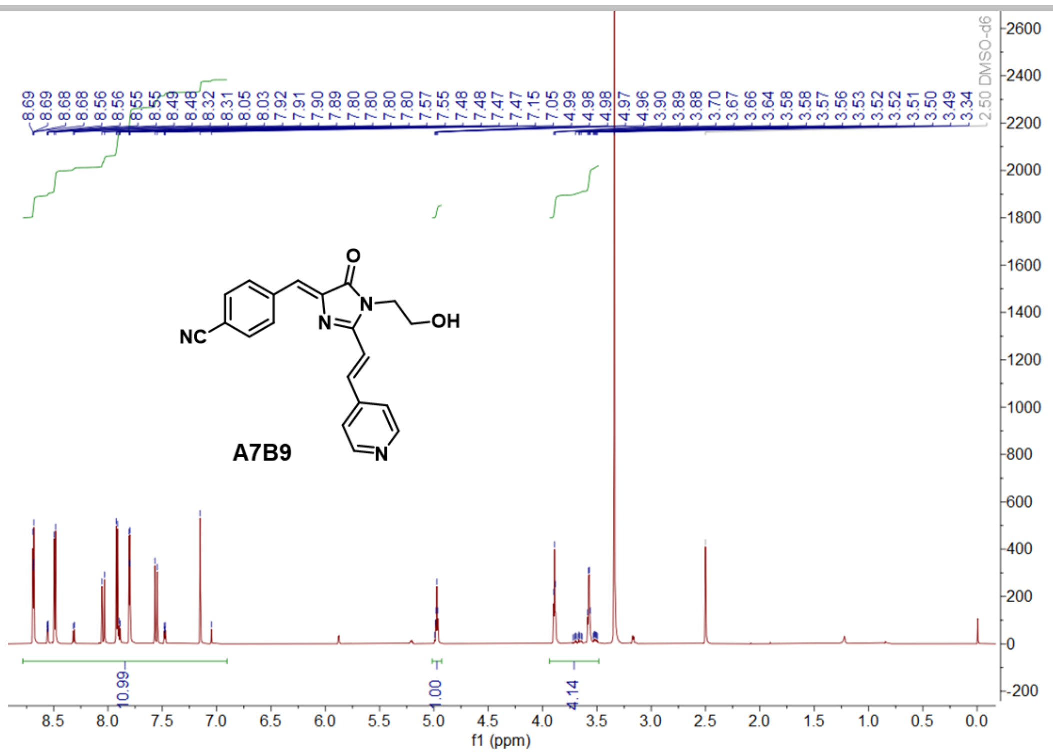

^{1}H NMR spectrum of A7B9 ($(CD_3)_2SO$)

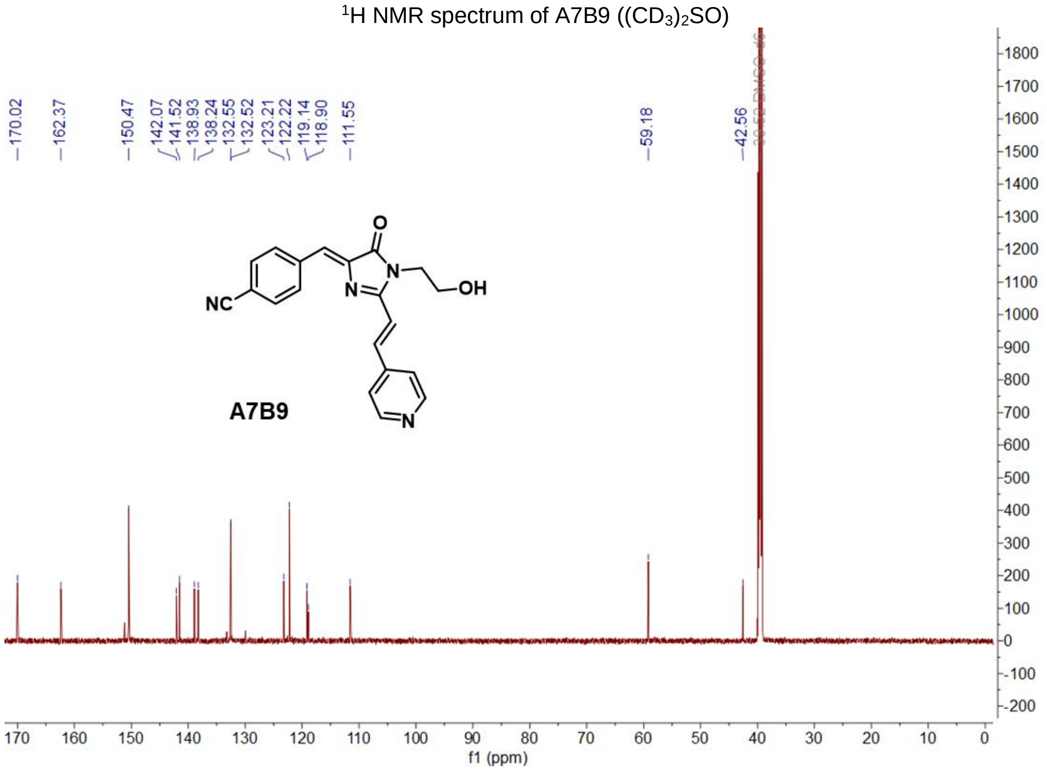

^{13}C NMR spectrum of A7B9 ($(CD_3)_2SO$)

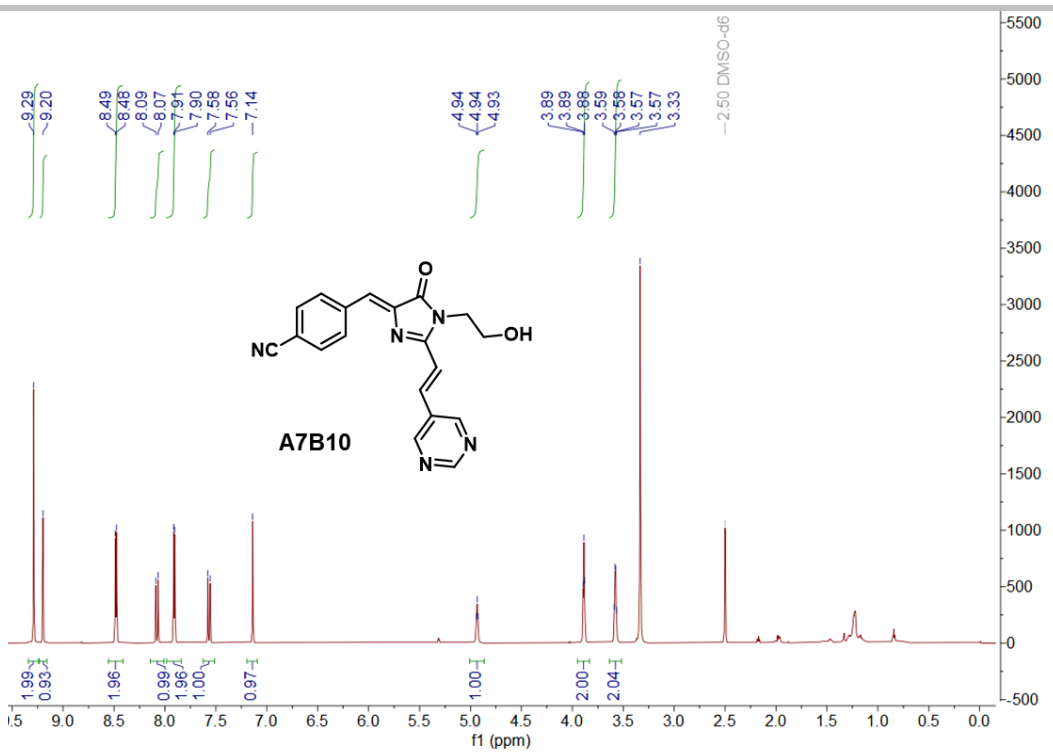

^{1}H NMR spectrum of A7B10 ($(CD_3)_2SO$)

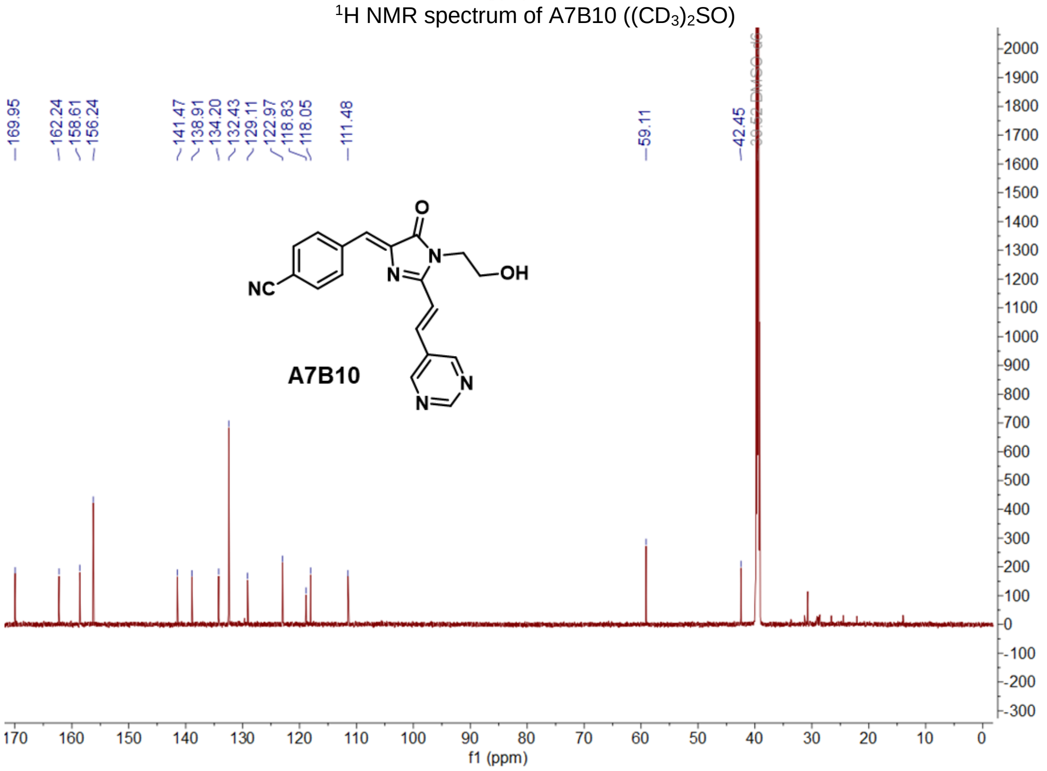

^{13}C NMR spectrum of A7B10 ($(CD_3)_2SO$)

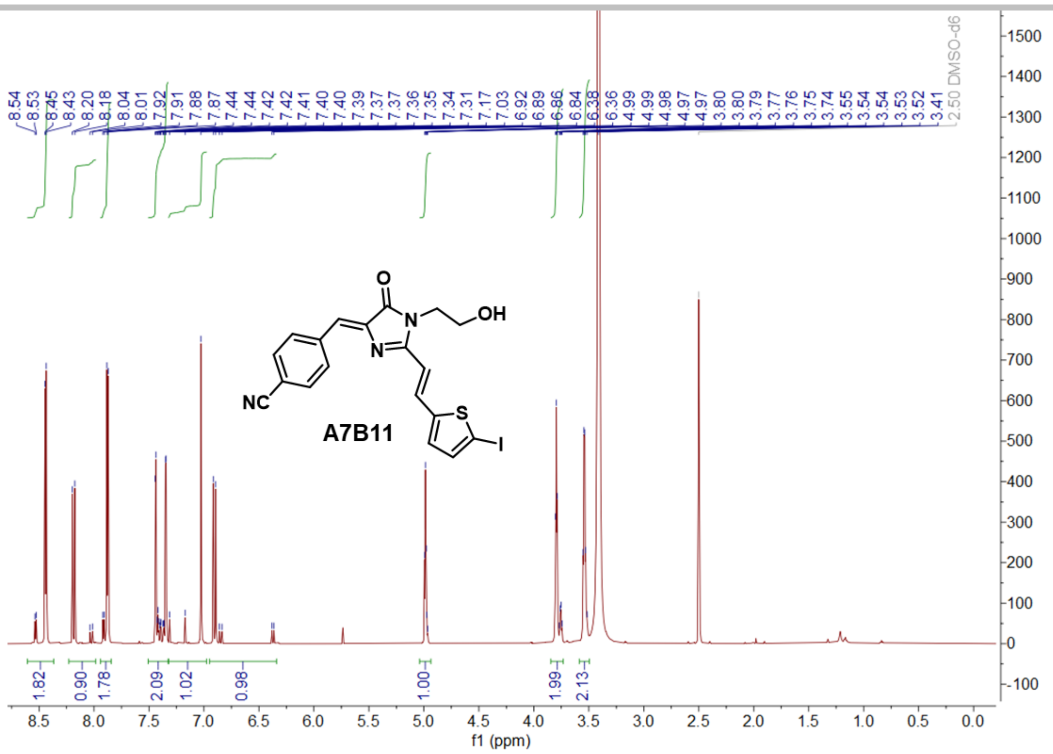

^{1}H NMR spectrum of A7B11 ($(CD_3)_2SO$)

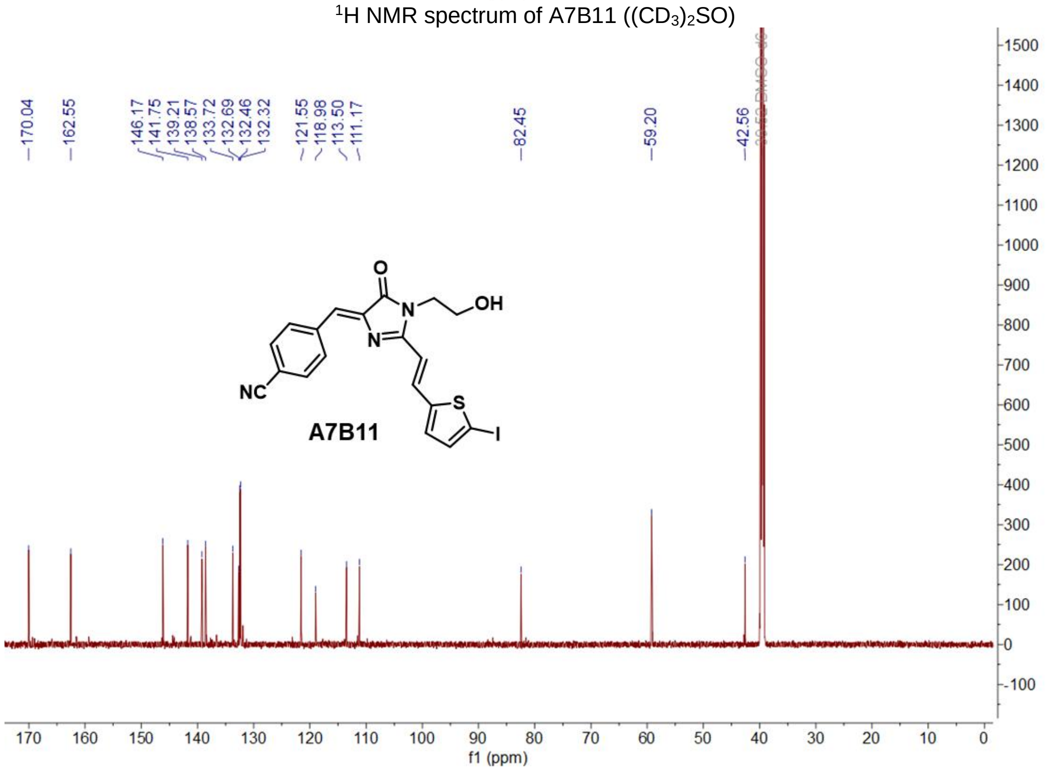

^{13}C NMR spectrum of A7B11 ($(CD_3)_2SO$)

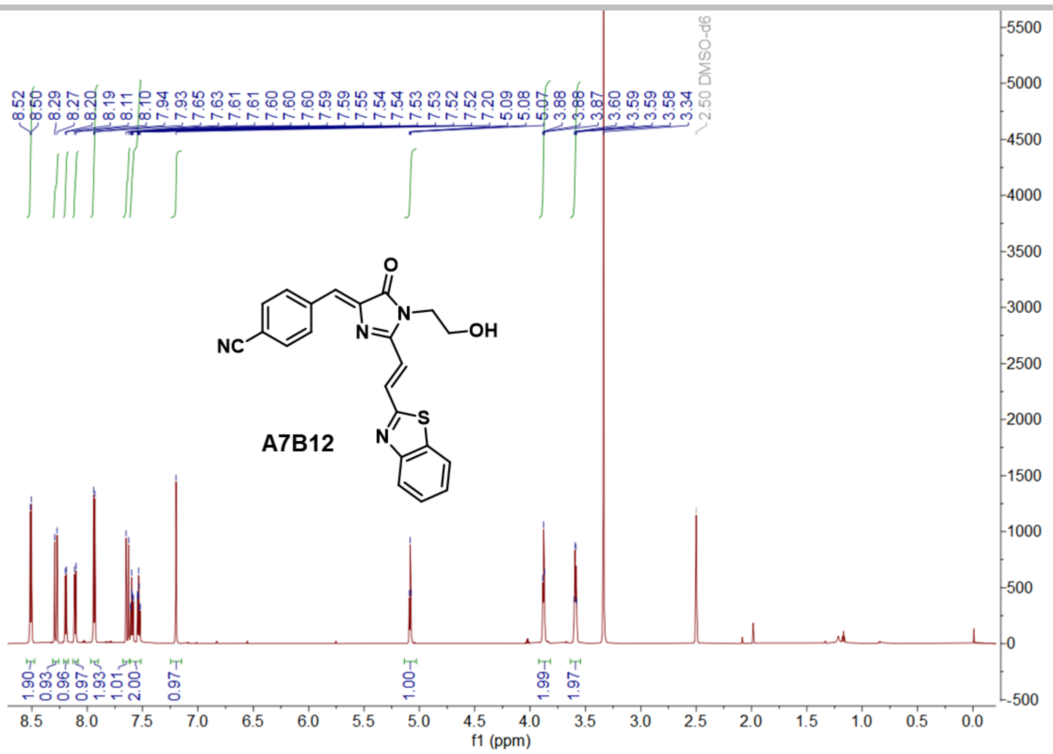

^{1}H NMR spectrum of A7B12 ($(CD_3)_2SO$)

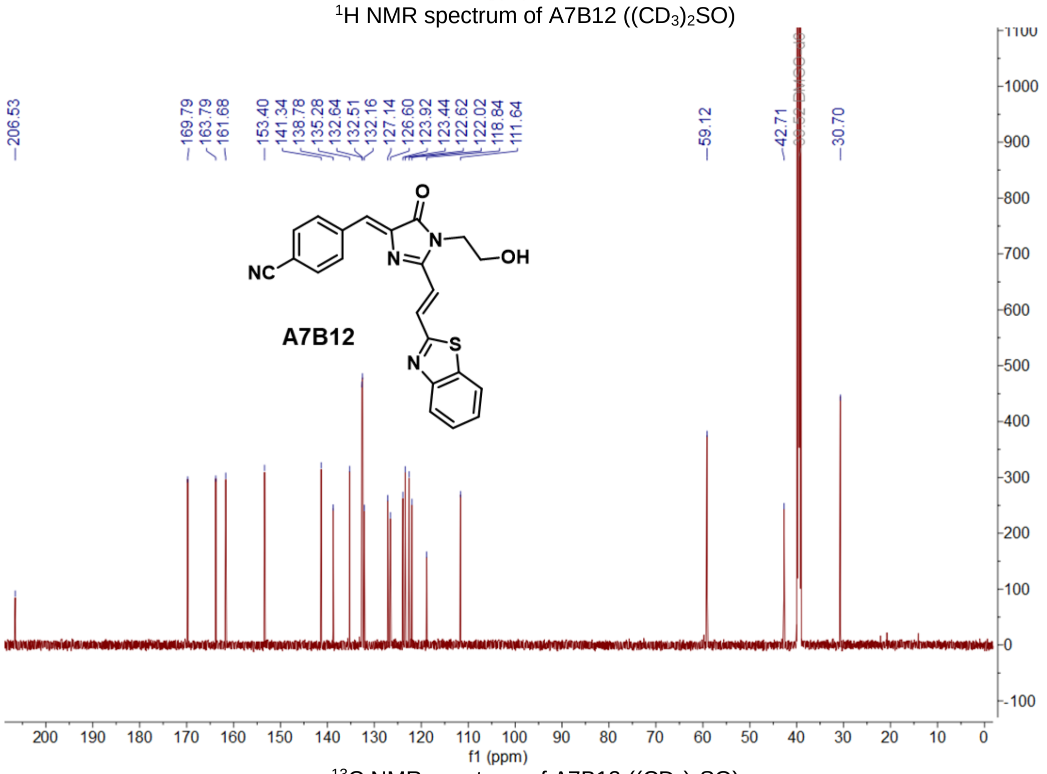

^{13}C NMR spectrum of A7B12 ($(CD_3)_2SO$)

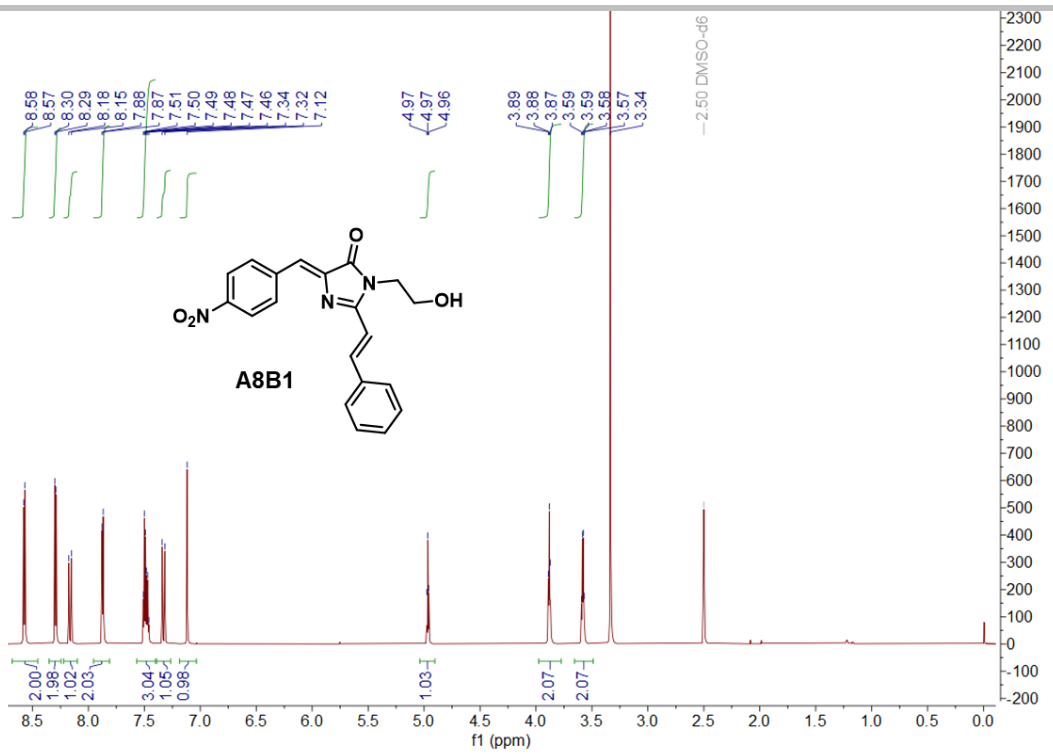

^{1}H NMR spectrum of A8B1 ($(CD_3)_2SO$)

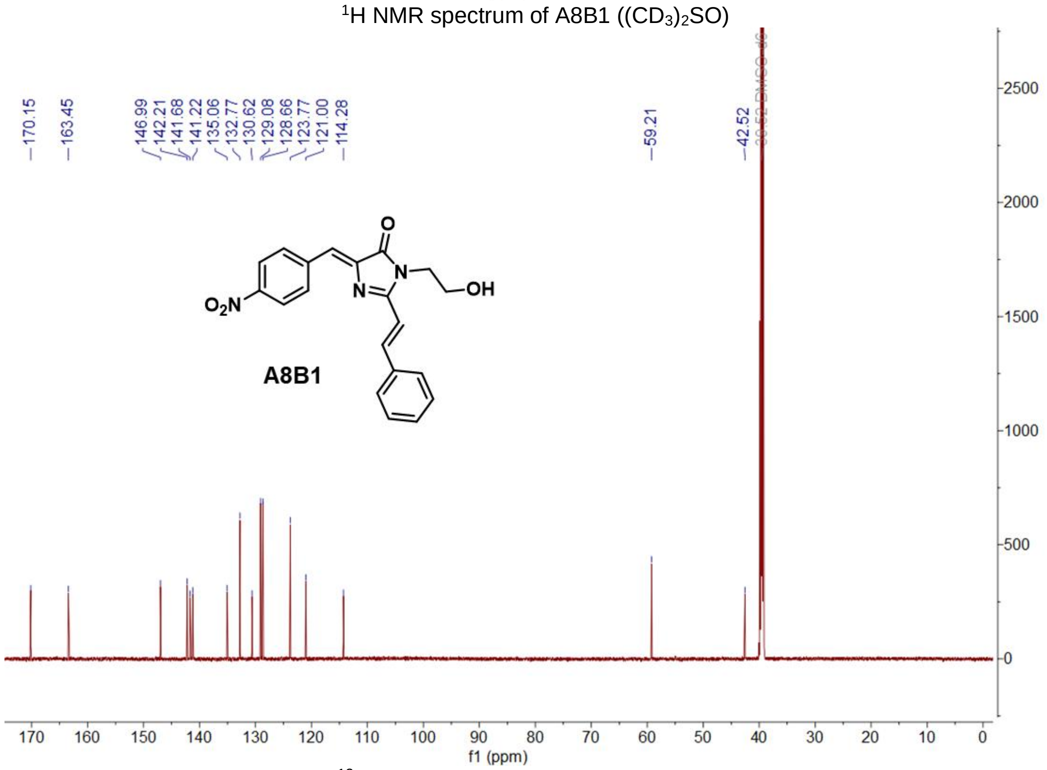

^{13}C NMR spectrum of A8B1 ($(CD_3)_2SO$)

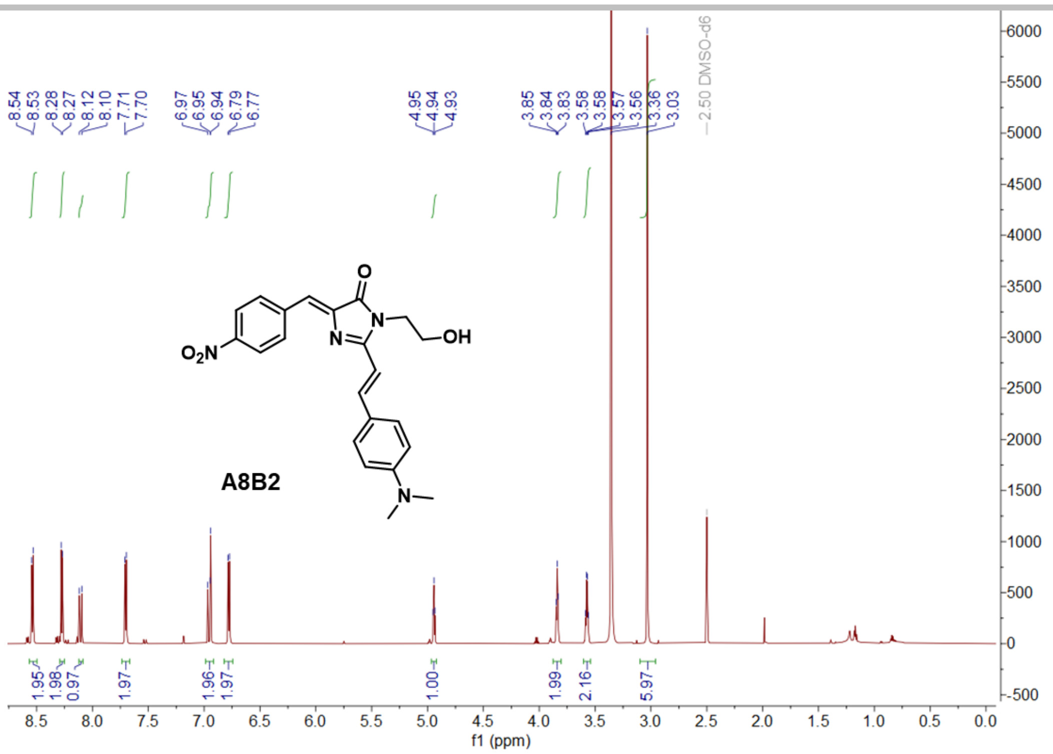

^{1}H NMR spectrum of A8B2 ($(CD_3)_2SO$)

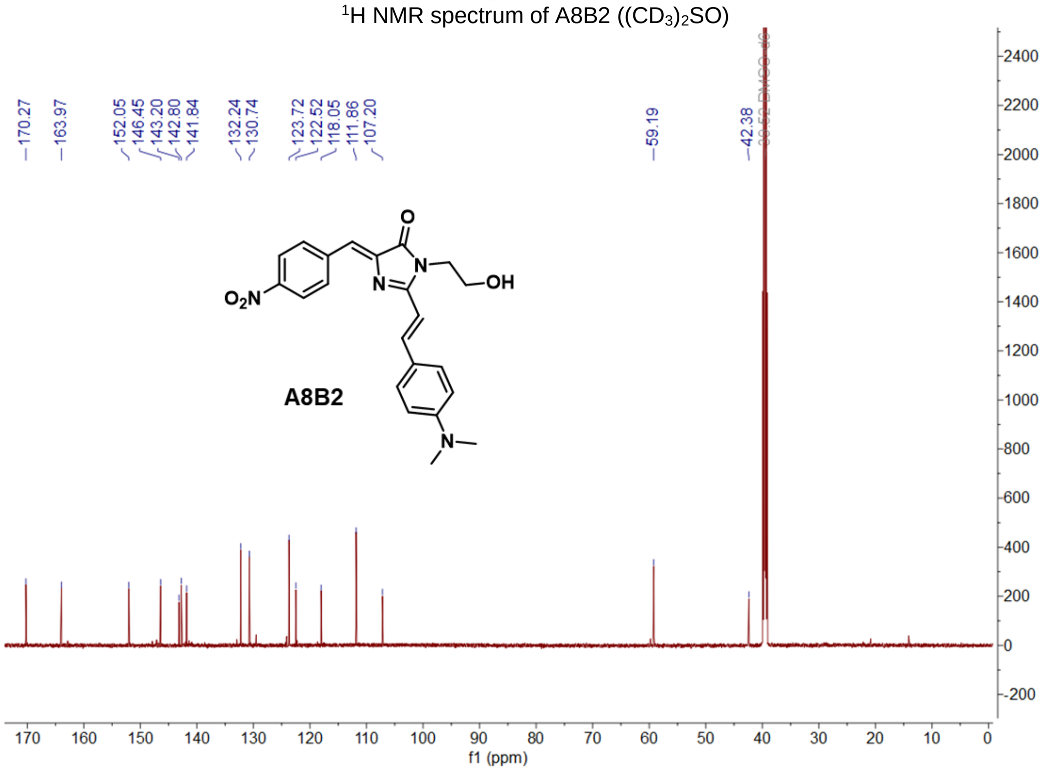

^{13}C NMR spectrum of A8B2 ($(CD_3)_2SO$)

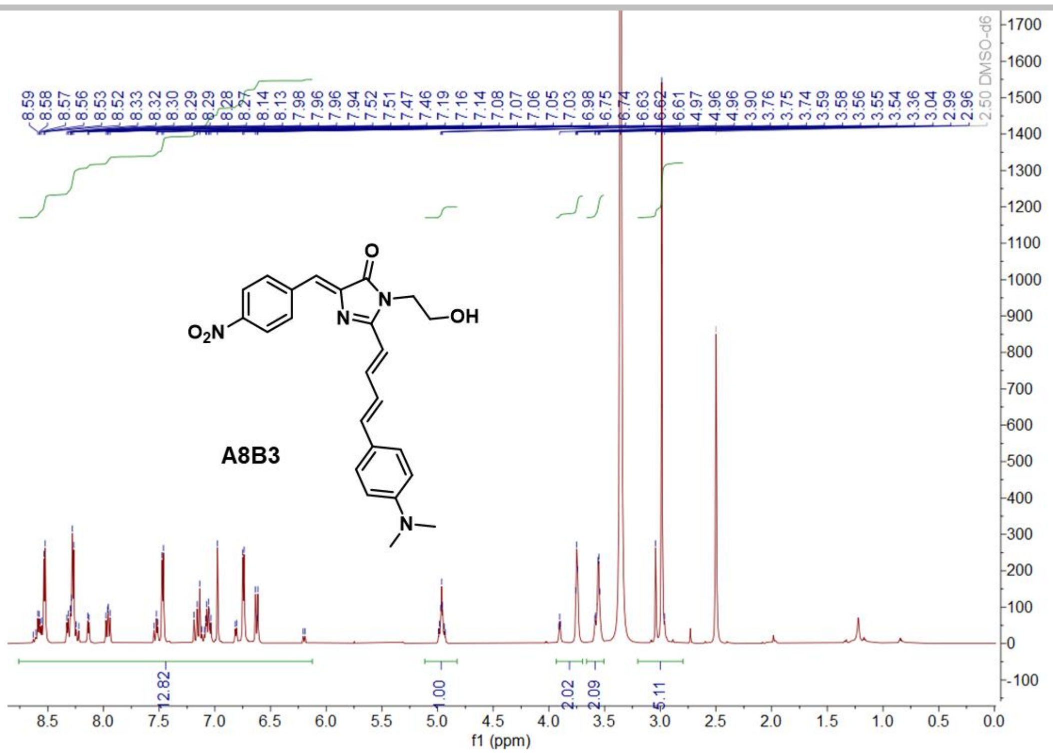

^{1}H NMR spectrum of A8B3 (($(CD_3)_2SO$)

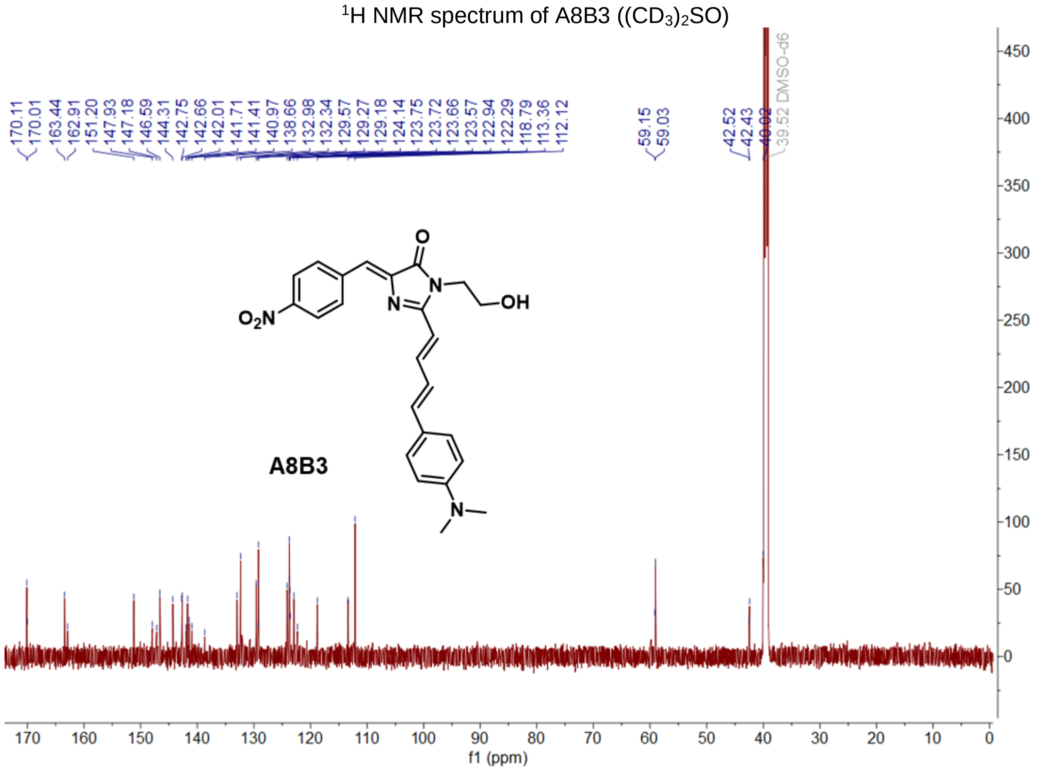

^{13}C NMR spectrum of A8B3 (($(CD_3)_2SO$)

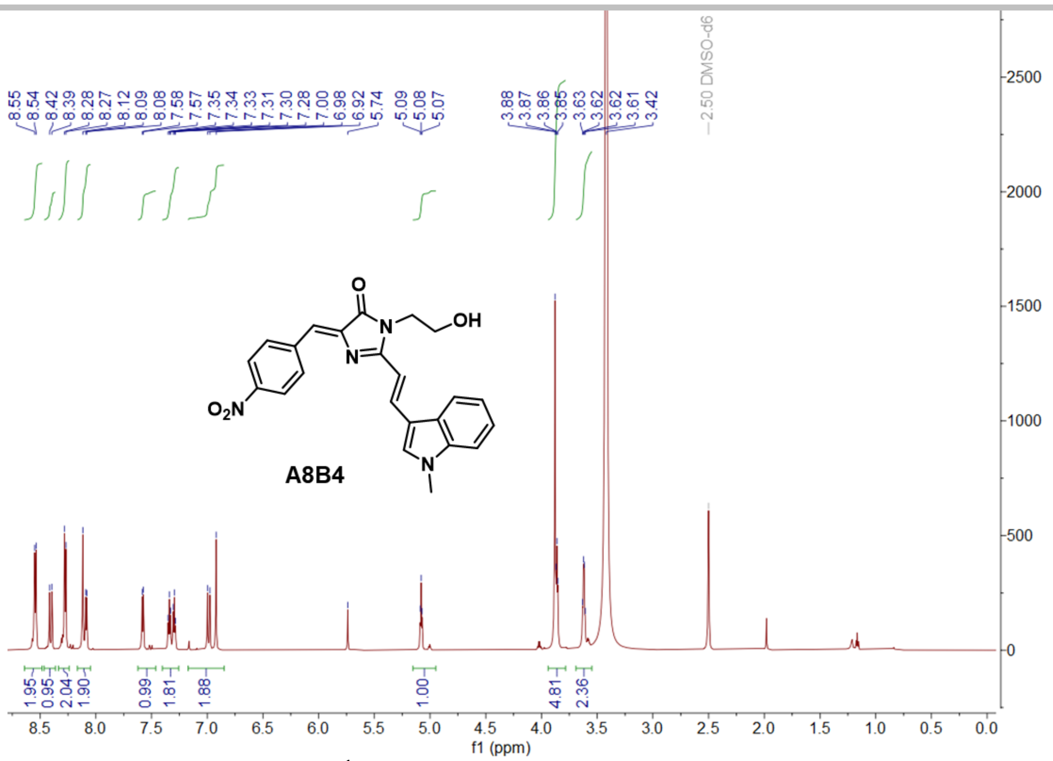

^{1}H NMR spectrum of A8B4 ($(CD_3)_2SO$)

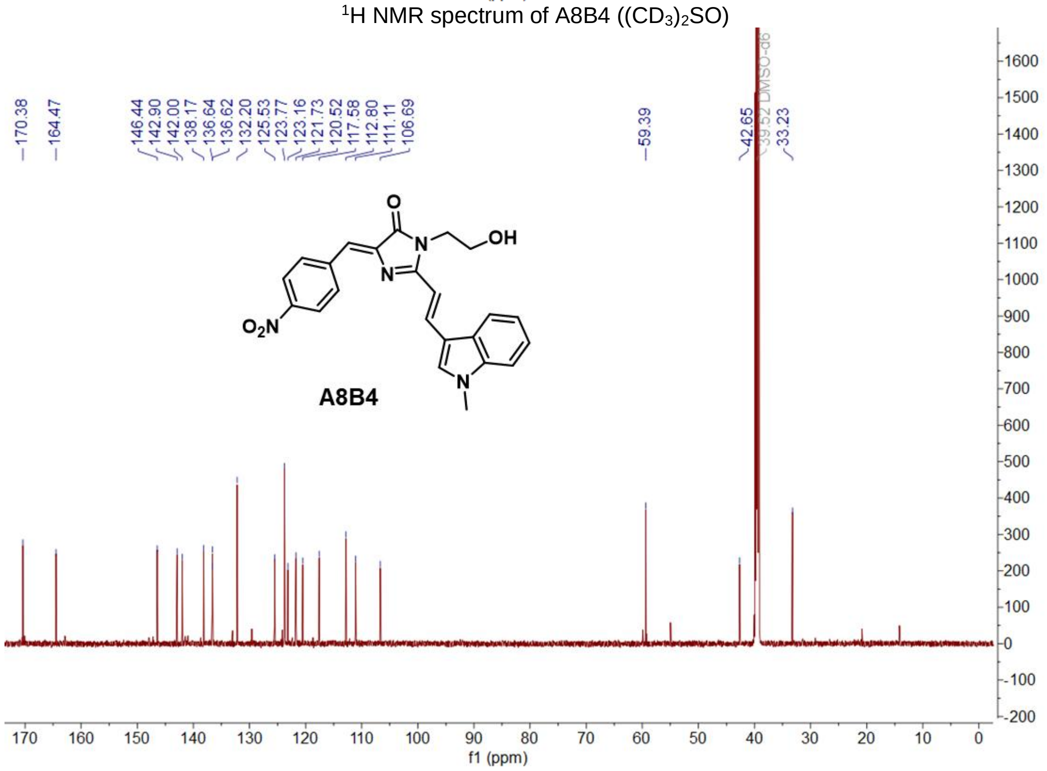

^{13}C NMR spectrum of A8B4 ($(CD_3)_2SO$)

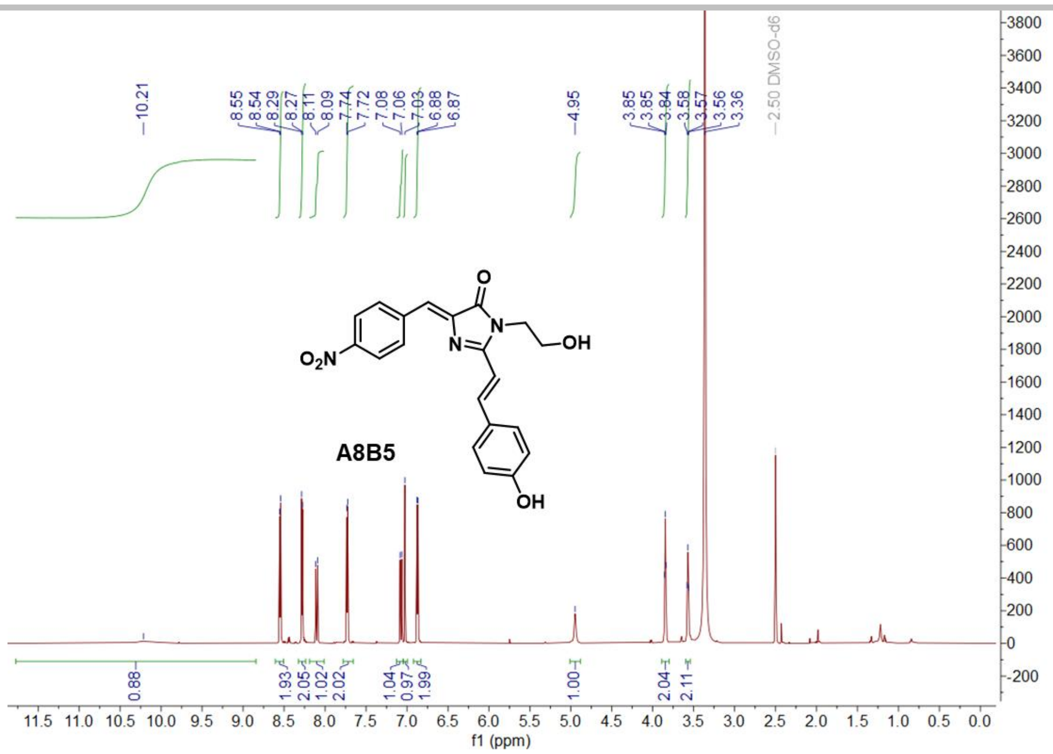

^{1}H NMR spectrum of A8B5 ($(CD_3)_2SO$)

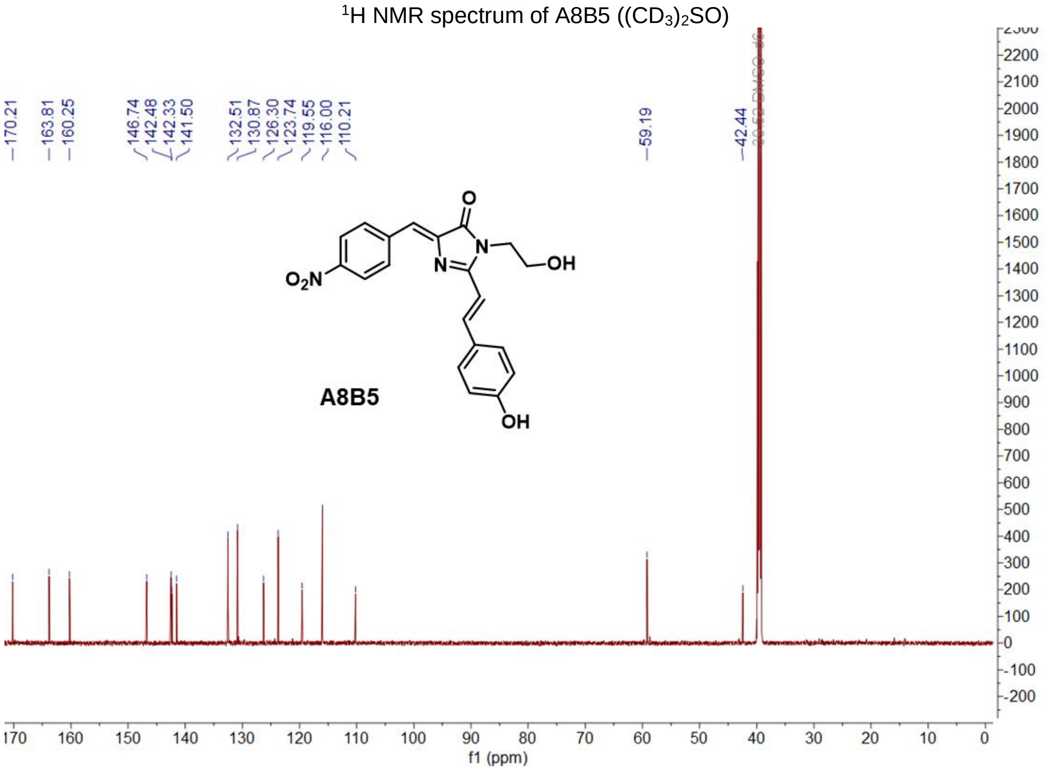

^{13}C NMR spectrum of A8B5 ($(CD_3)_2SO$)

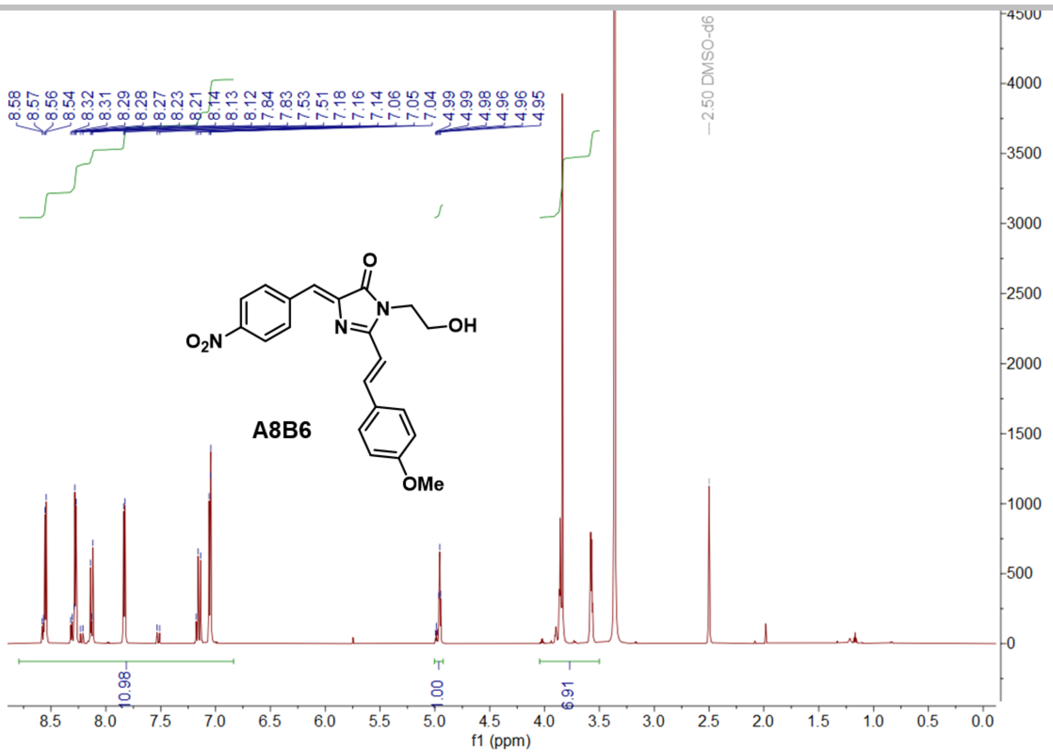

^{1}H NMR spectrum of A8B6 ($(CD_3)_2SO$)

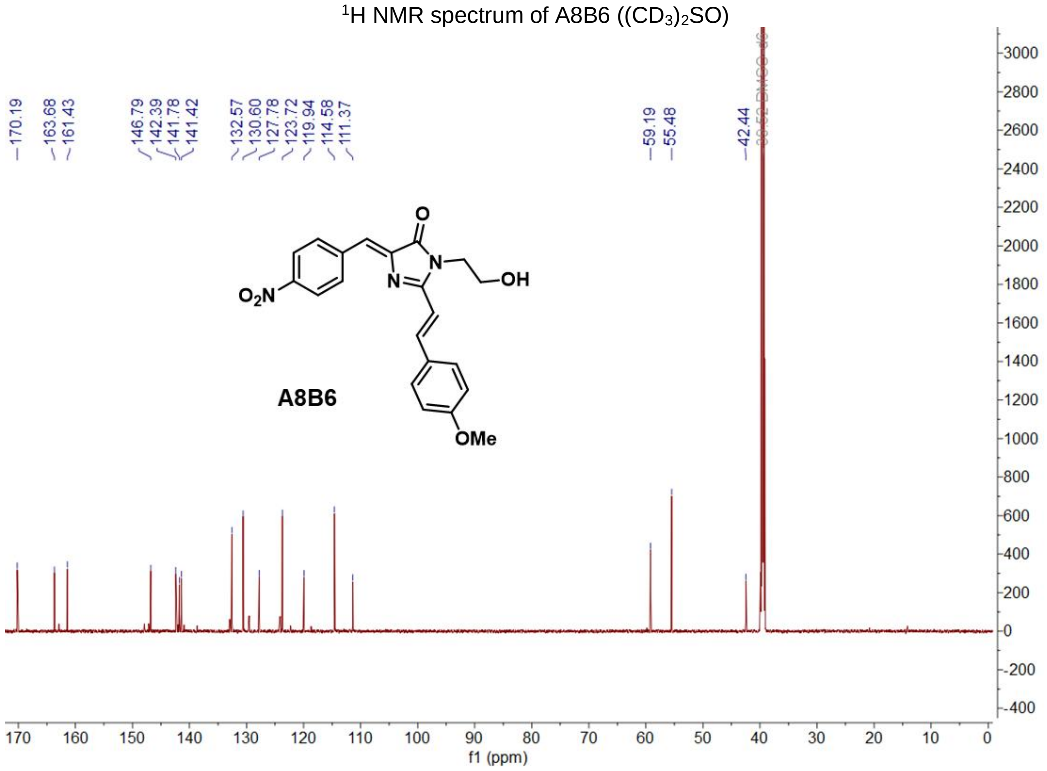

^{13}C NMR spectrum of A8B6 ($(CD_3)_2SO$)

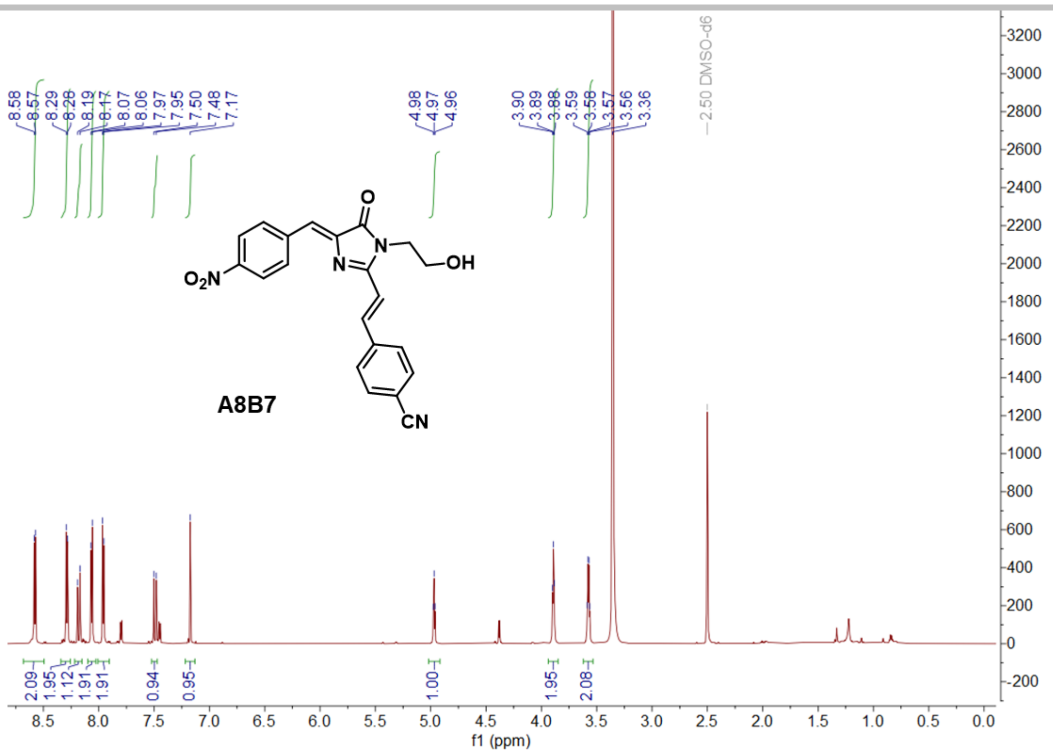

^1H NMR spectrum of A8B7 ($(CD_3)_2SO$)

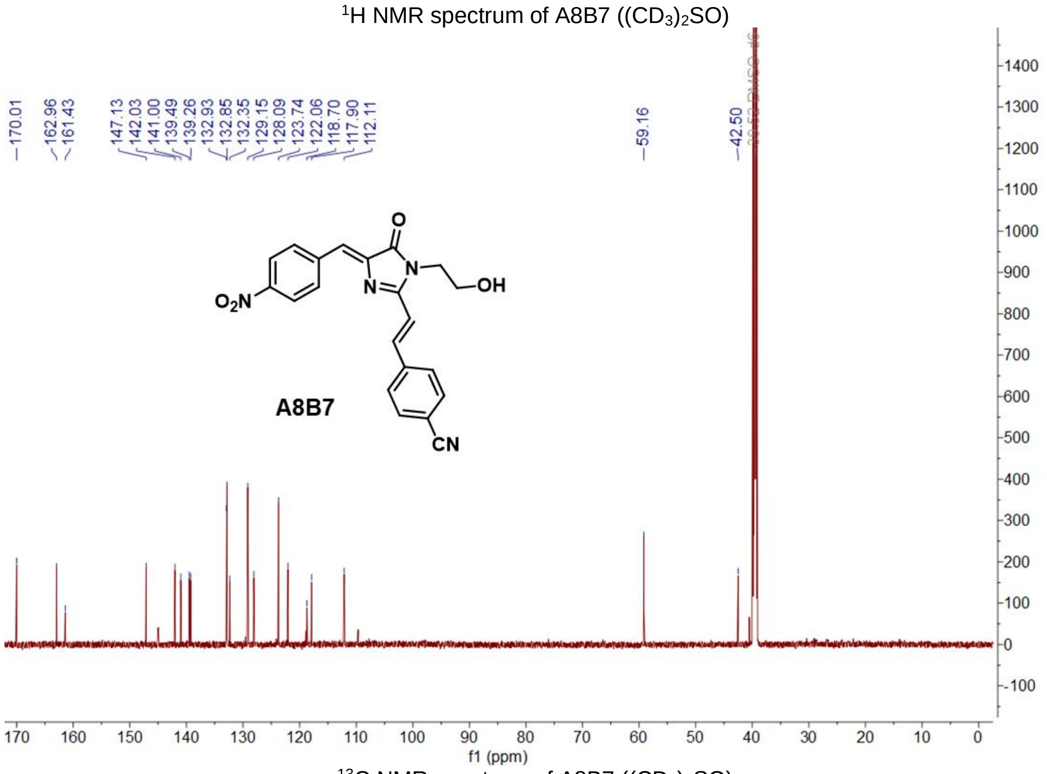

^{13}C NMR spectrum of A8B7 ($(CD_3)_2SO$)

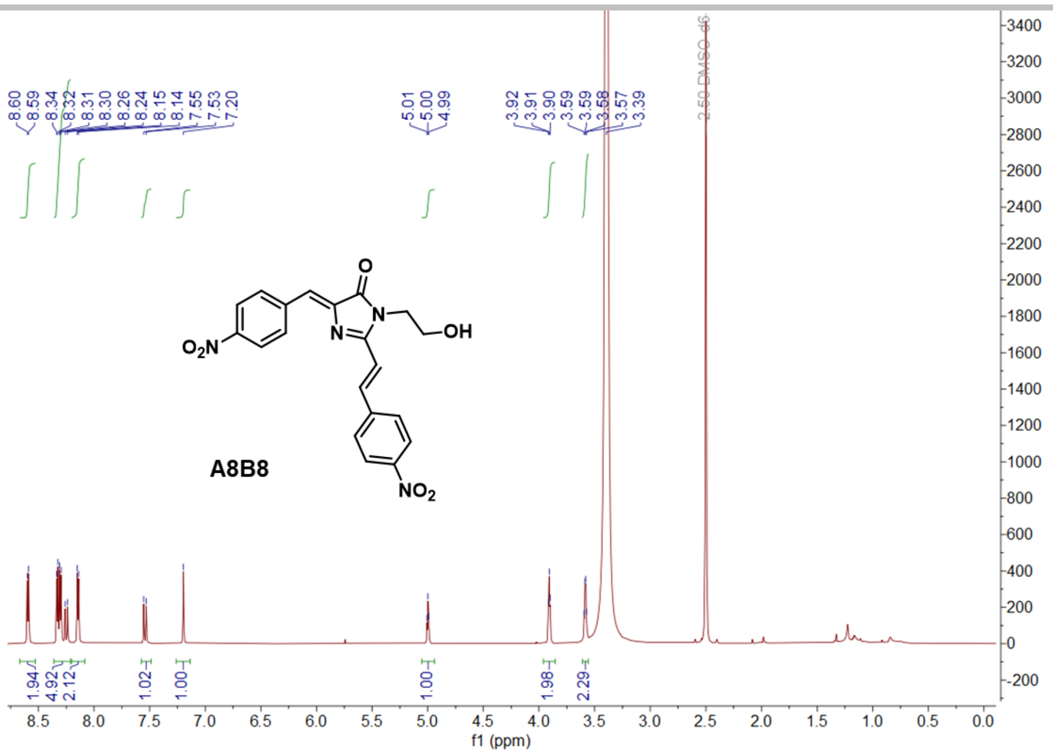

^{1}H NMR spectrum of A8B8 ($(CD_3)_2SO$)

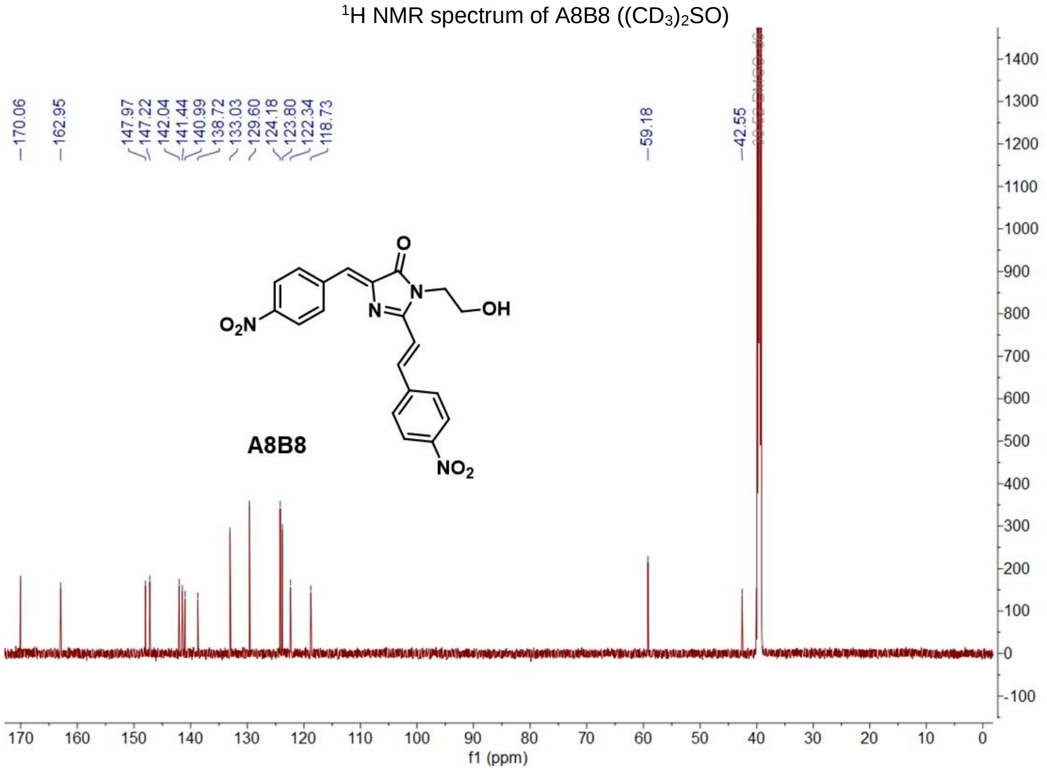

^{13}C NMR spectrum of A8B8 ($(CD_3)_2SO$)

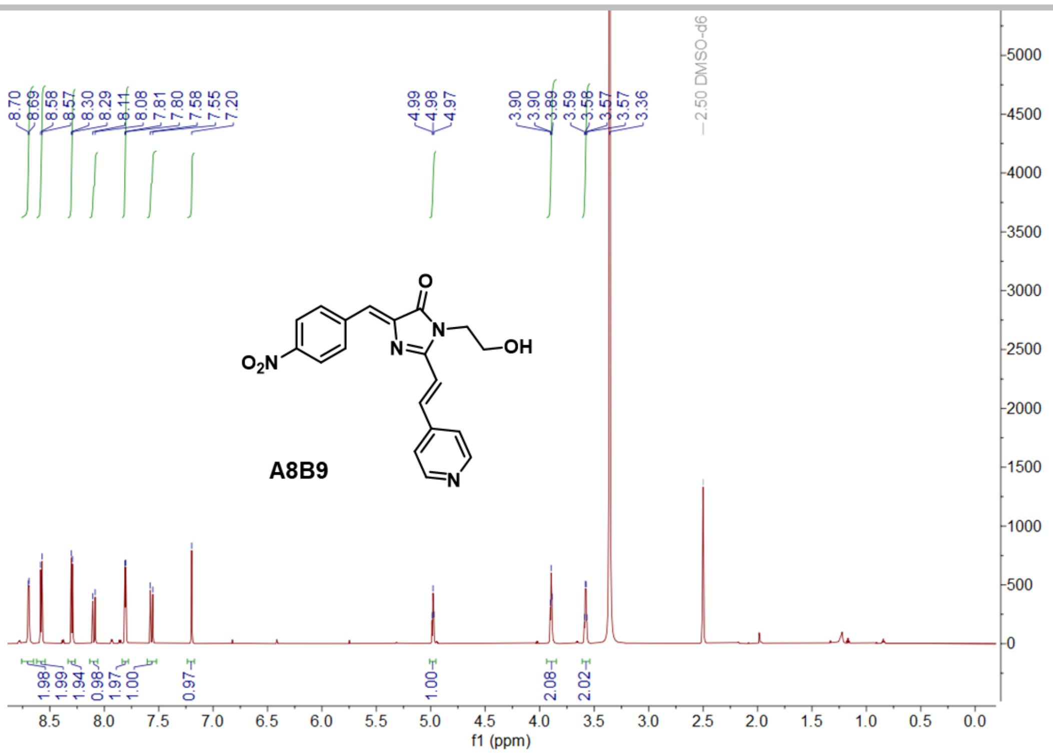

^{1}H NMR spectrum of A8B9 ($(CD_3)_2SO$)

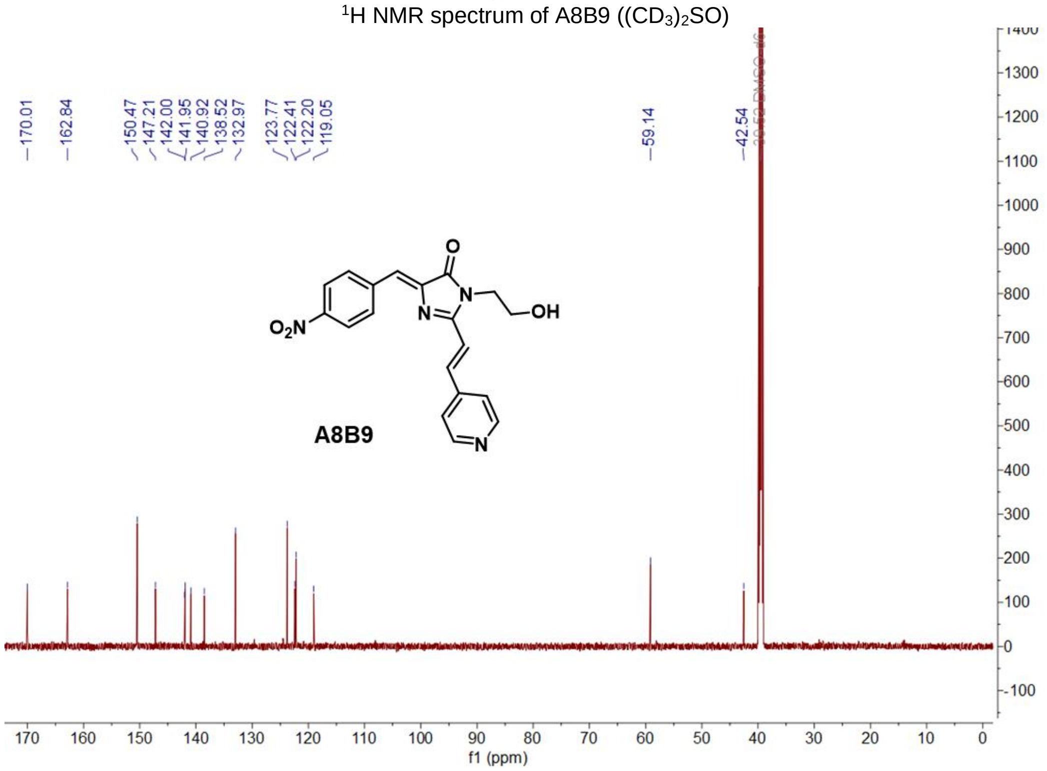

^{13}C NMR spectrum of A8B9 ($(CD_3)_2SO$)

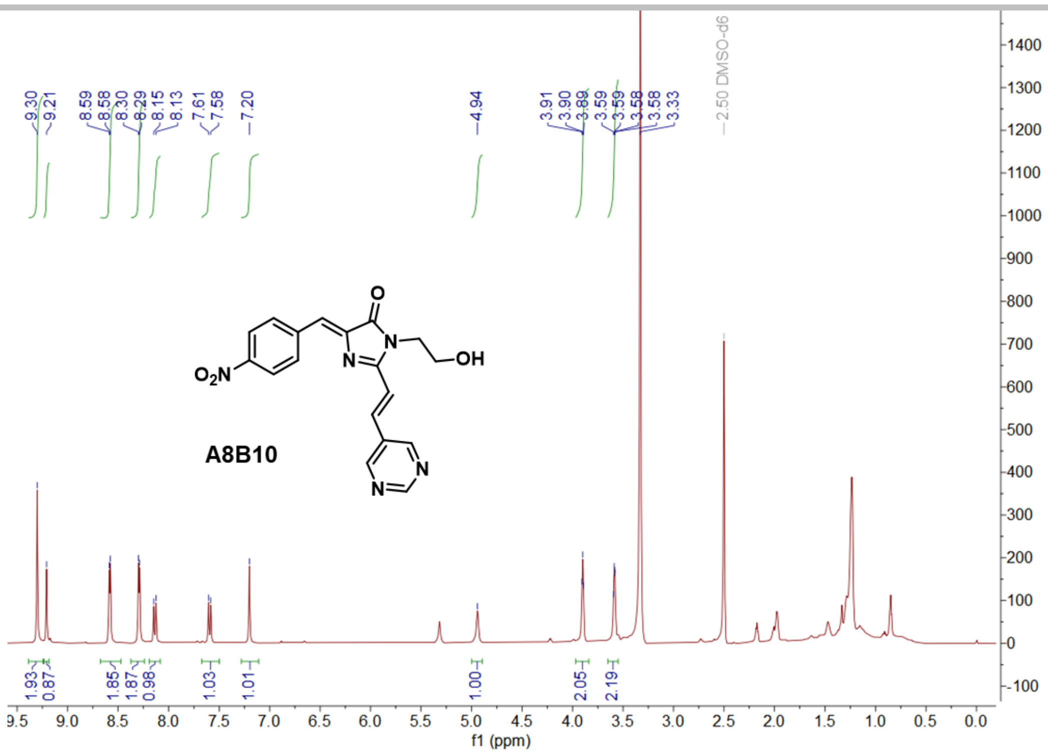

^{1}H NMR spectrum of A8B10 ($(CD_3)_2SO$)

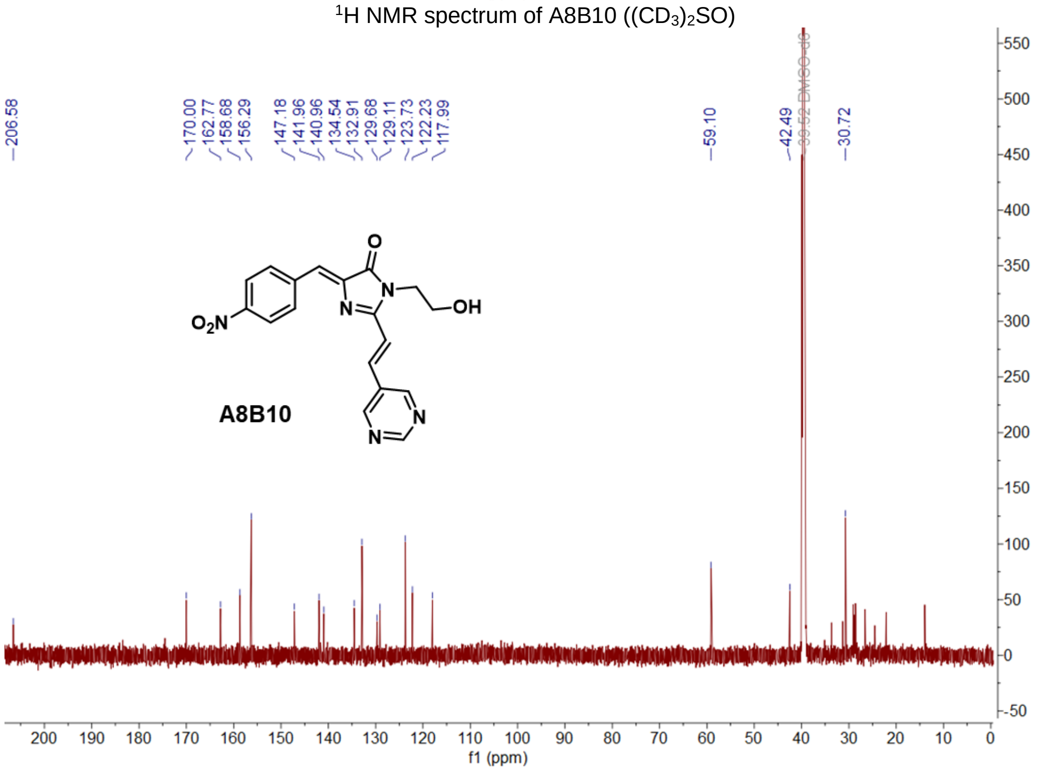

^{13}C NMR spectrum of A8B10 ($(CD_3)_2SO$)

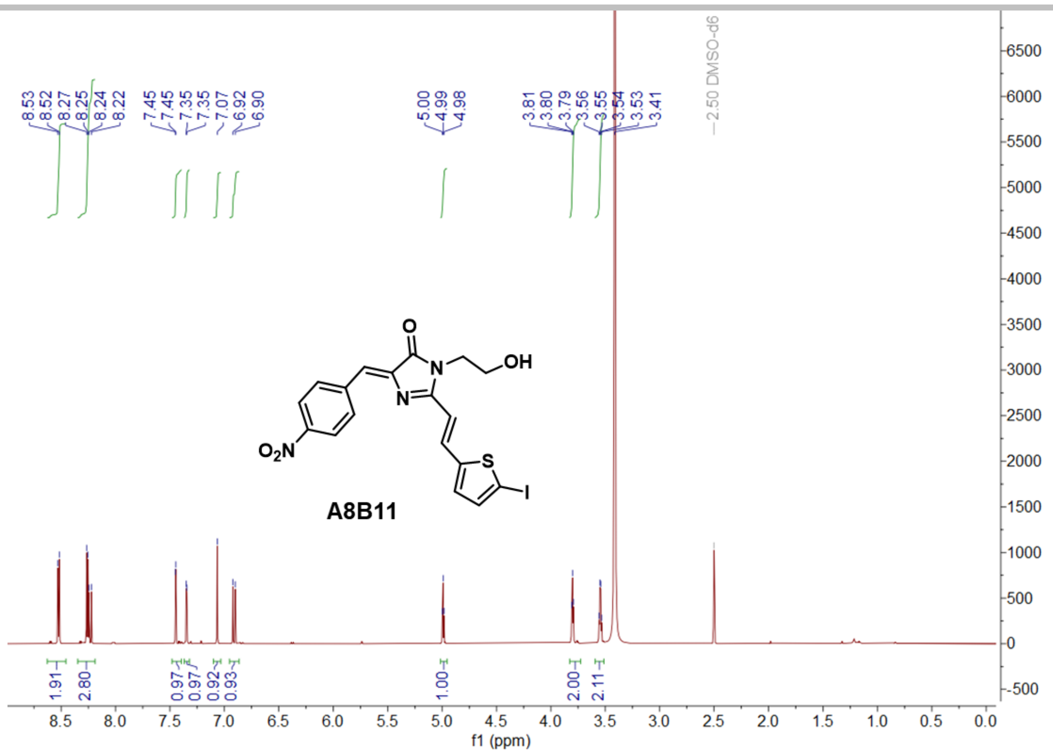

^1H NMR spectrum of A8B11 ($(CD_3)_2SO$)

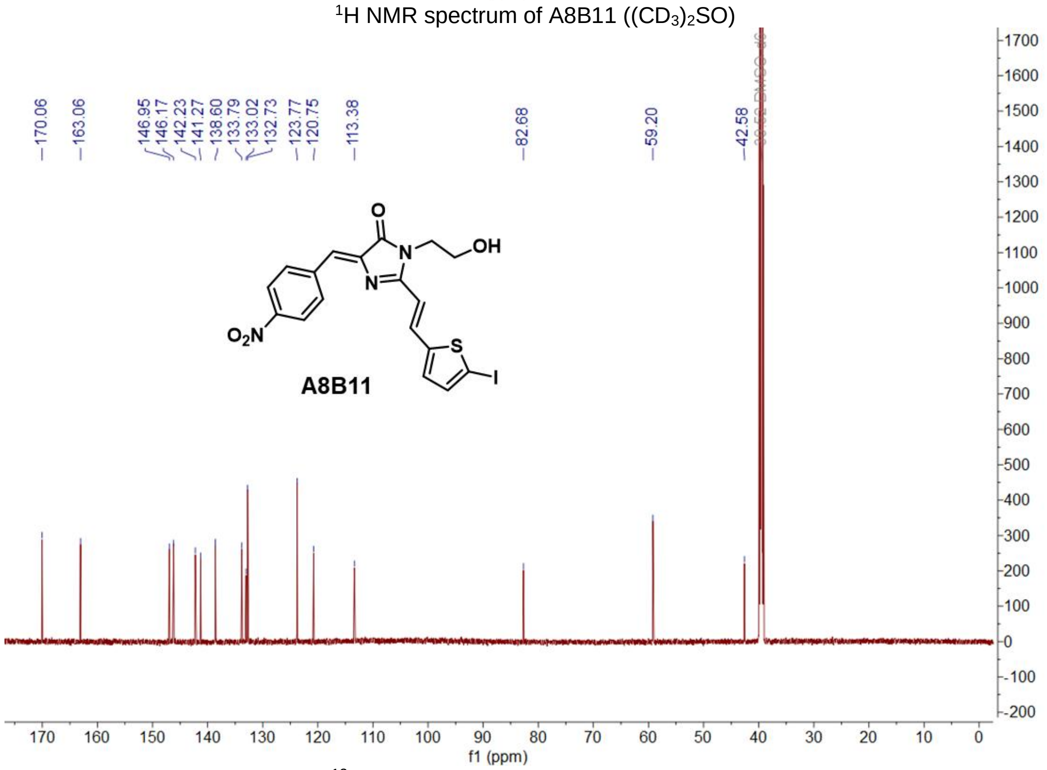

^{13}C NMR spectrum of A8B11 ($(CD_3)_2SO$)

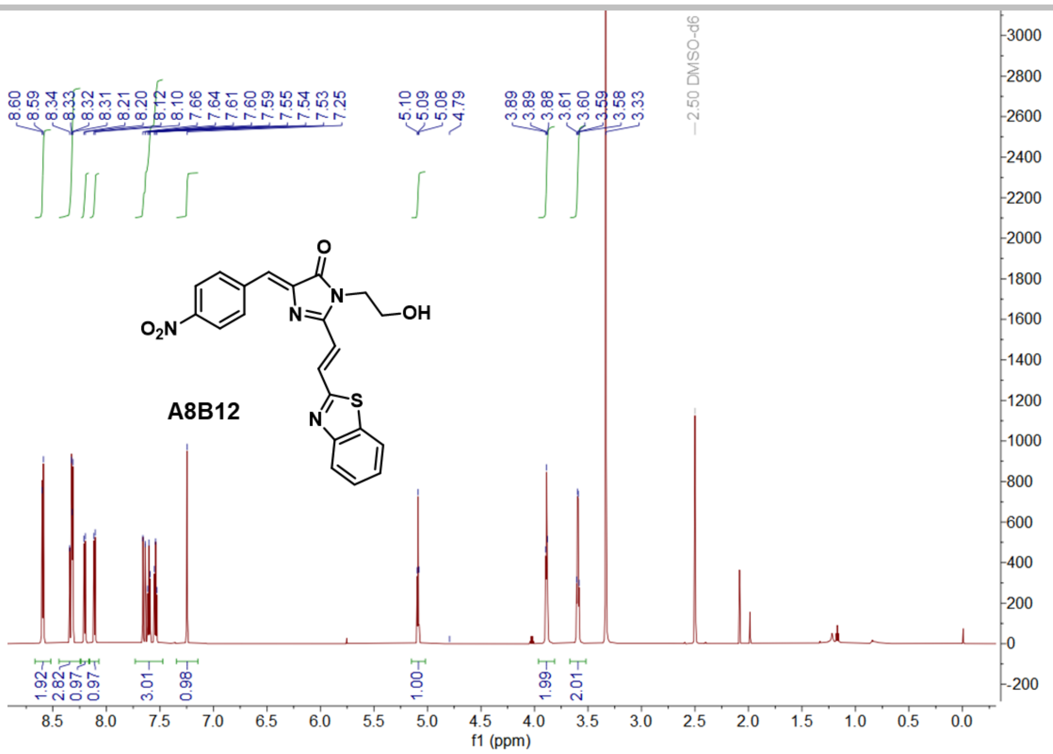

^1H NMR spectrum of A8B12 ($(CD_3)_2SO$)

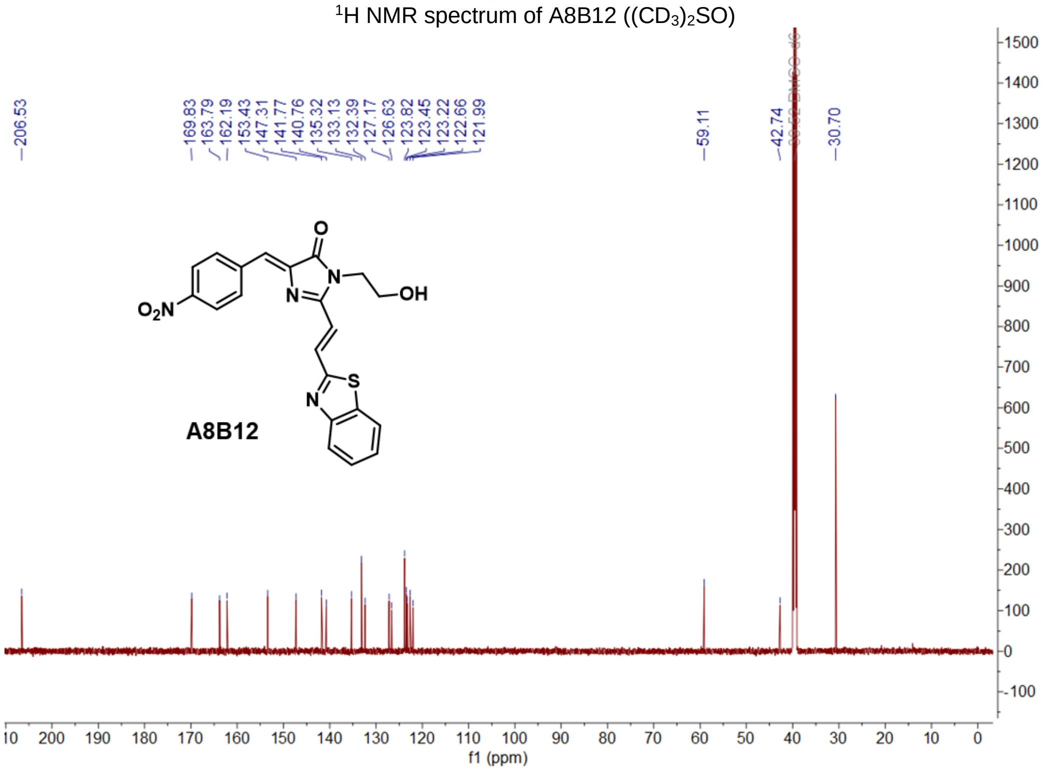

^{13}C NMR spectrum of A8B12 ($(CD_3)_2SO$)

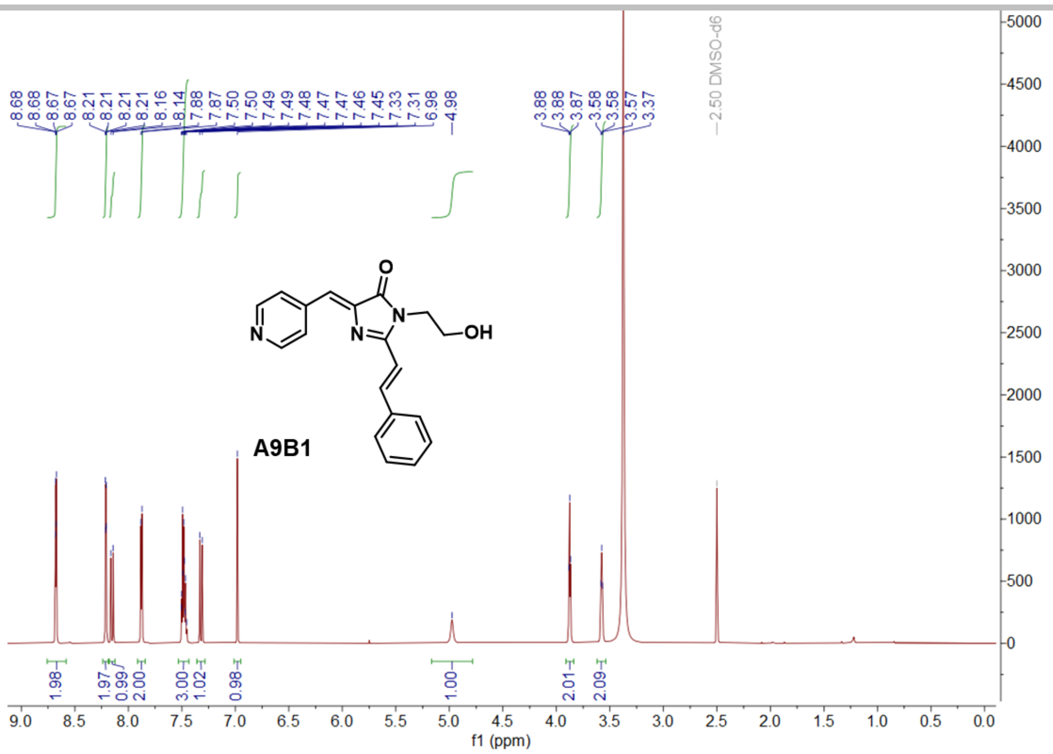

^{1}H NMR spectrum of A9B1 ($(CD_3)_2SO$)

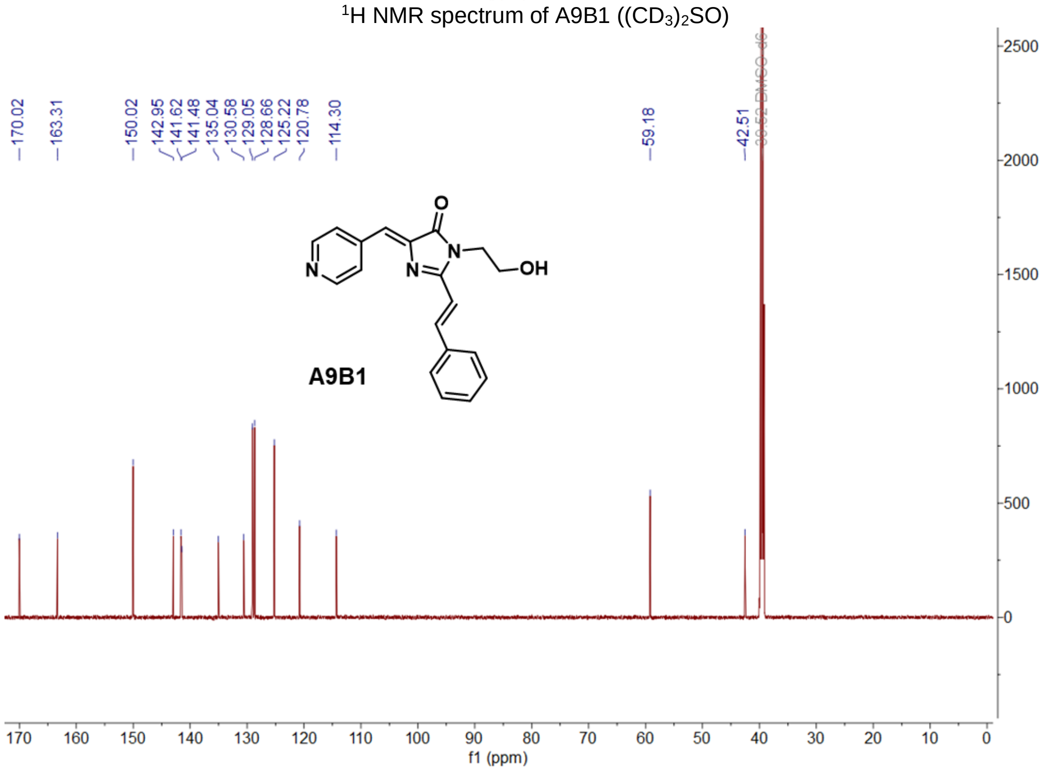

^{13}C NMR spectrum of A9B1 ($(CD_3)_2SO$)

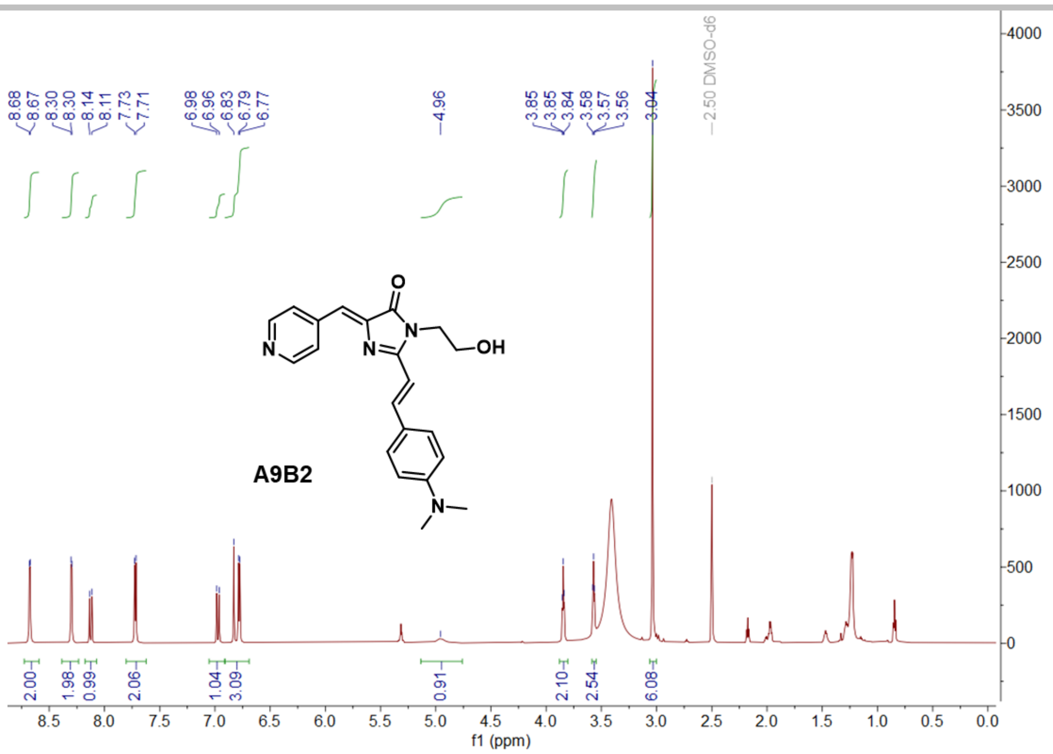

^{1}H NMR spectrum of A9B2 ($(CD_3)_2SO$)

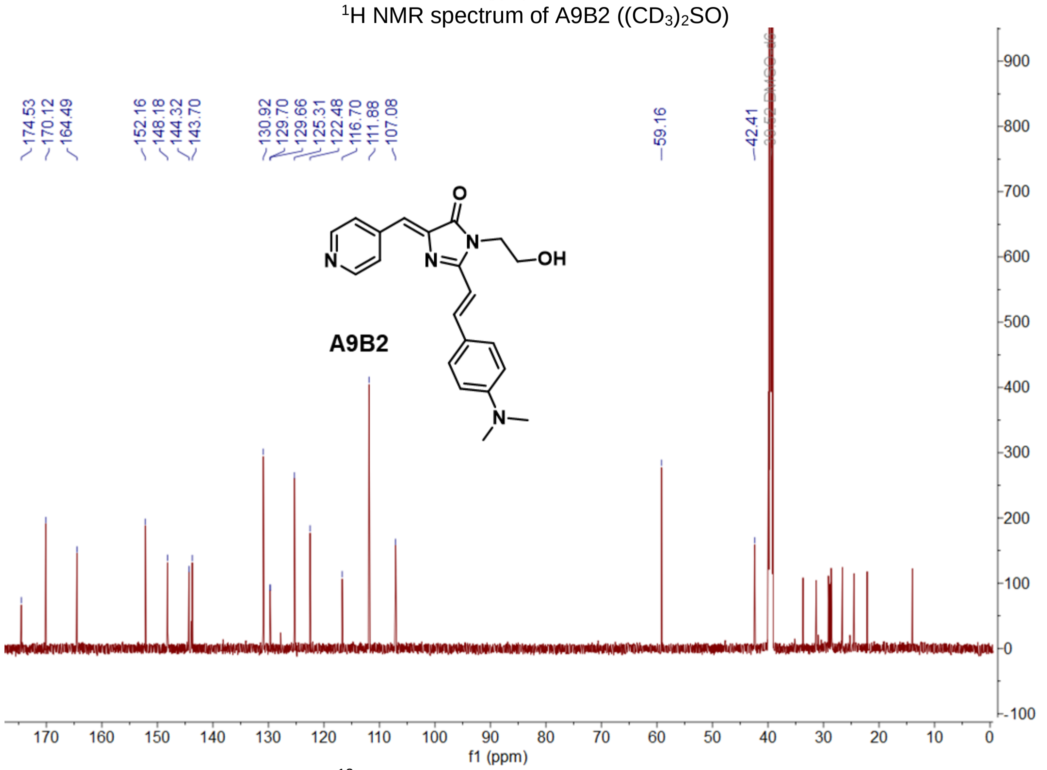

^{13}C NMR spectrum of A9B2 ($(CD_3)_2SO$)

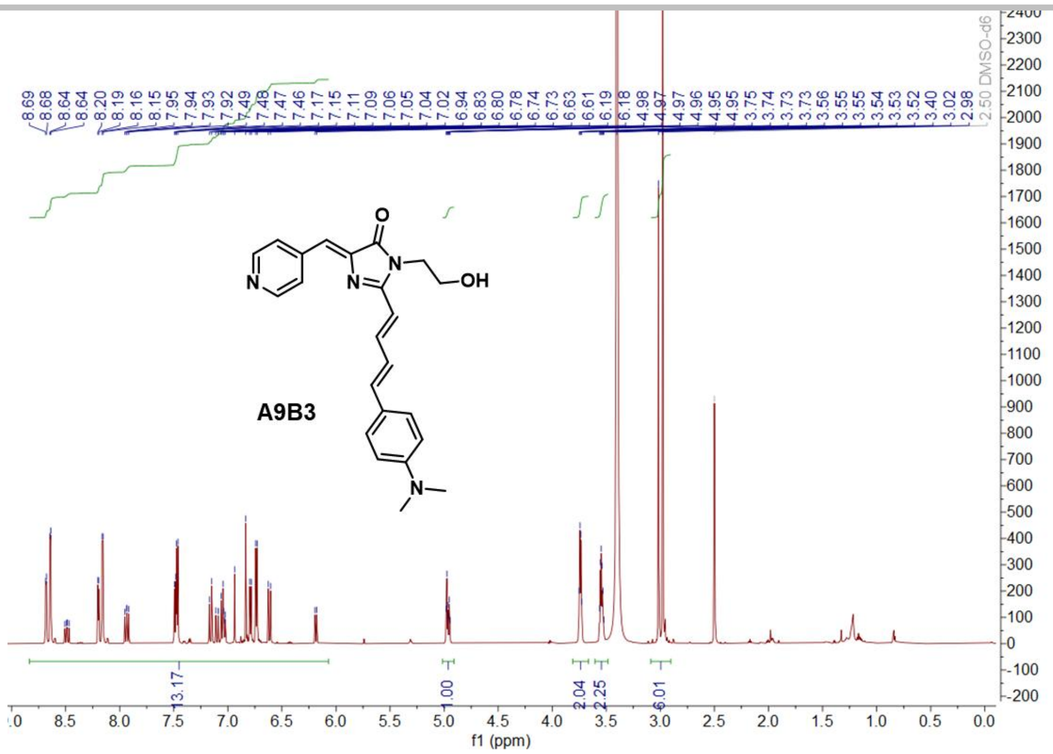

^{1}H NMR spectrum of A9B3 ($(CD_3)_2SO$)

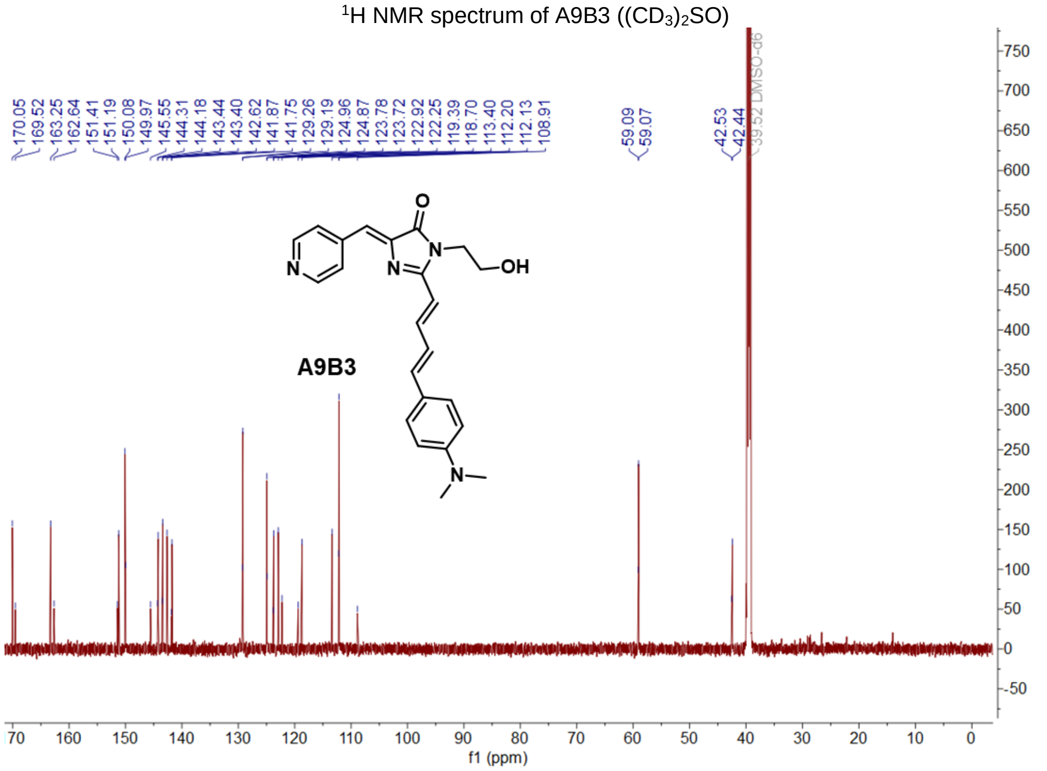

^{13}C NMR spectrum of A9B3 ($(CD_3)_2SO$)

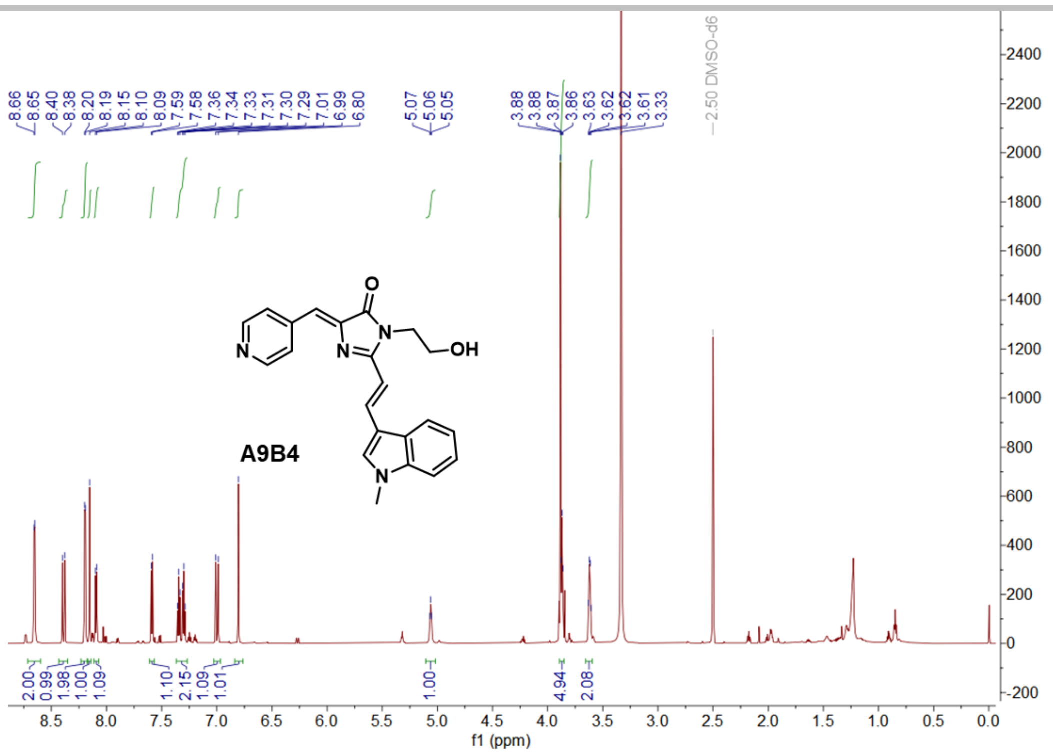

^{1}H NMR spectrum of A9B4 ($(CD_3)_2SO$)

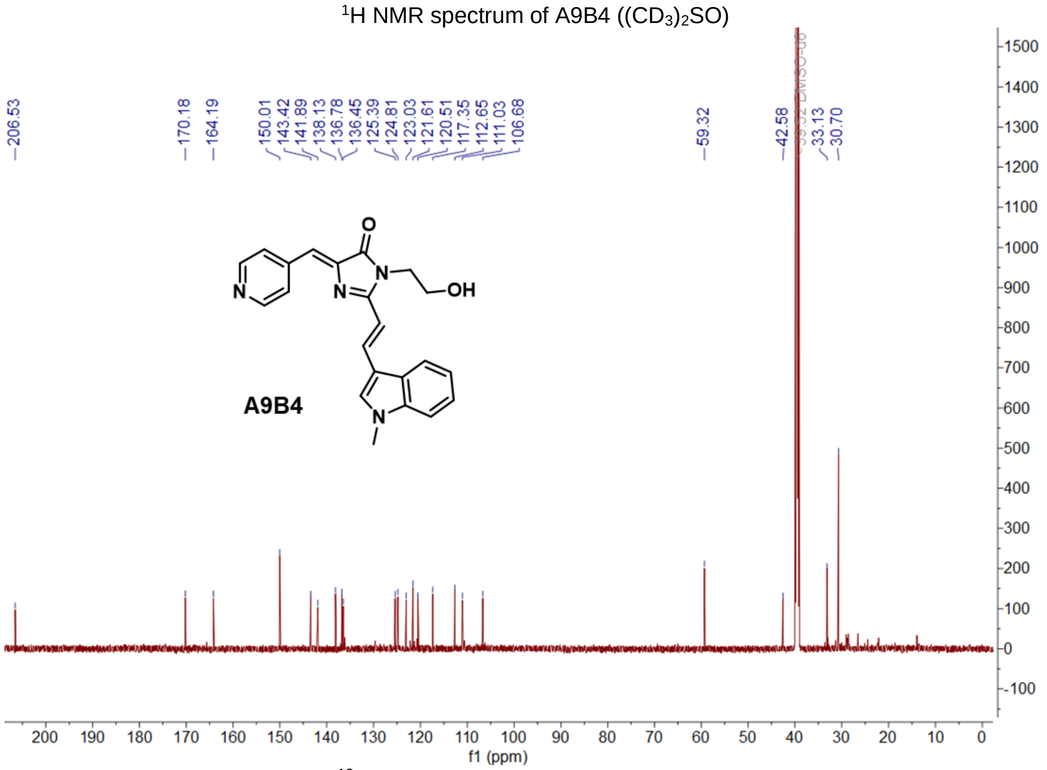

^{13}C NMR spectrum of A9B4 ($(CD_3)_2SO$)

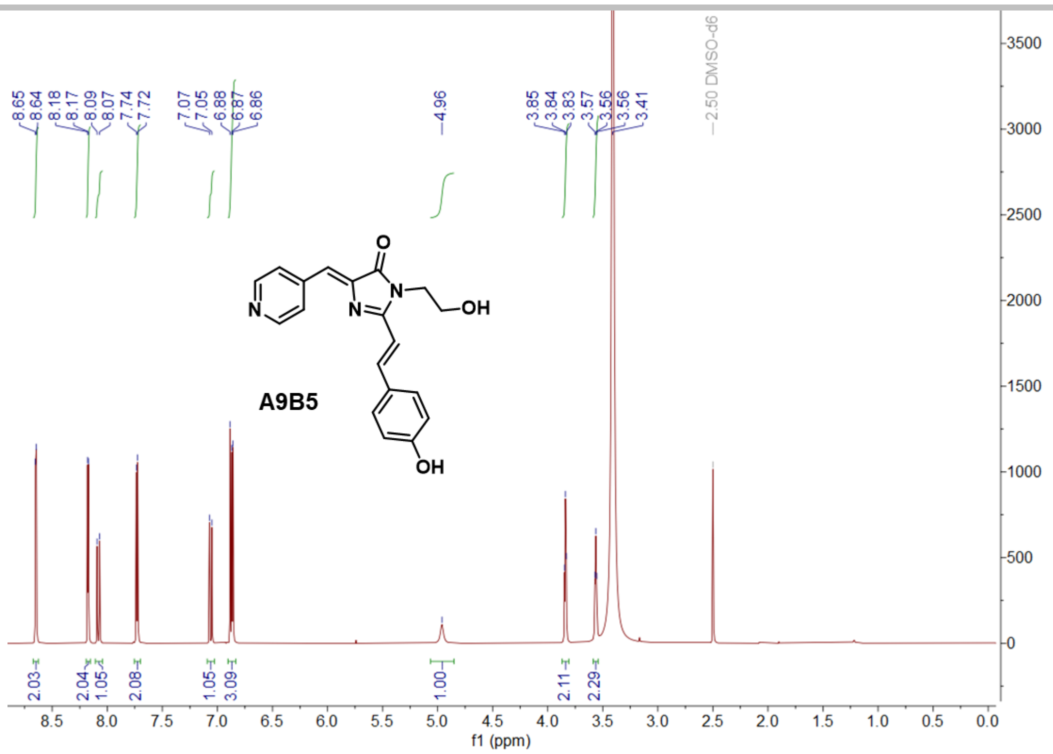

^{1}H NMR spectrum of A9B5 ($(CD_3)_2SO$)

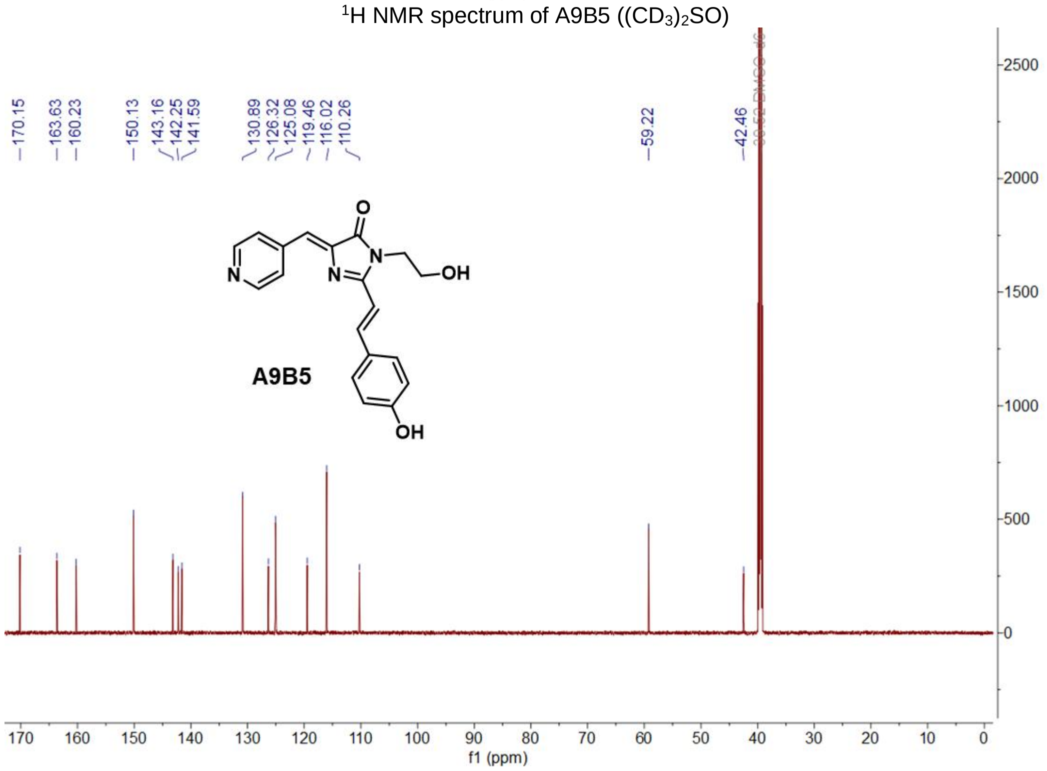

^{13}C NMR spectrum of A9B5 ($(CD_3)_2SO$)

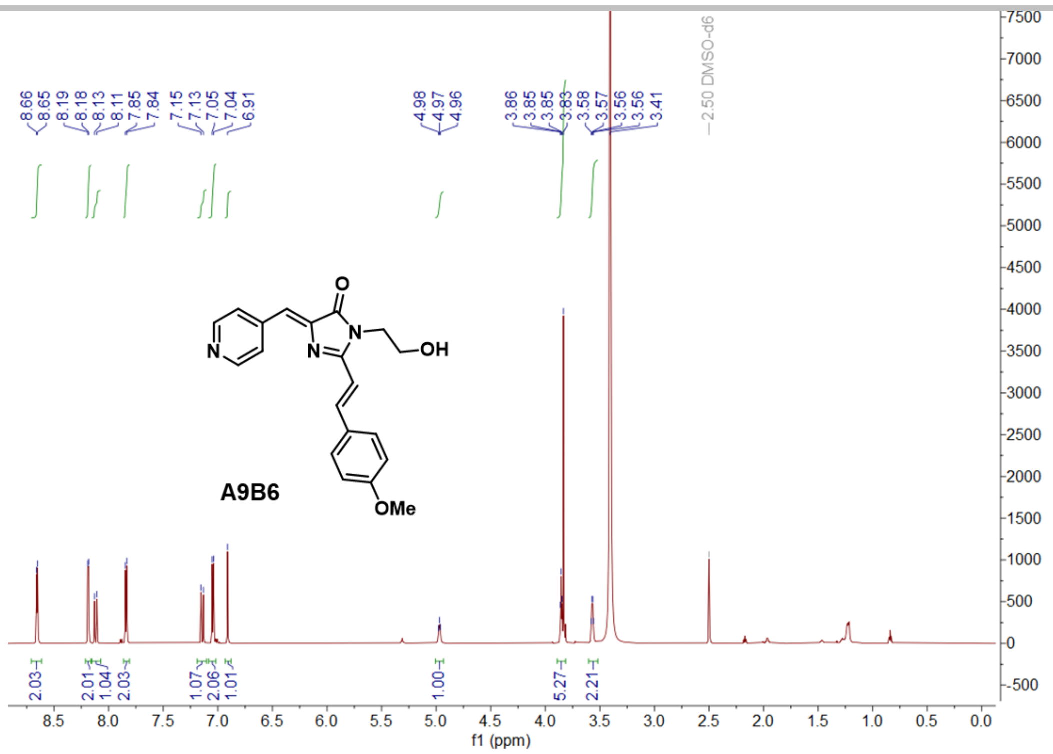

^{1}H NMR spectrum of A9B6 ($(CD_3)_2SO$)

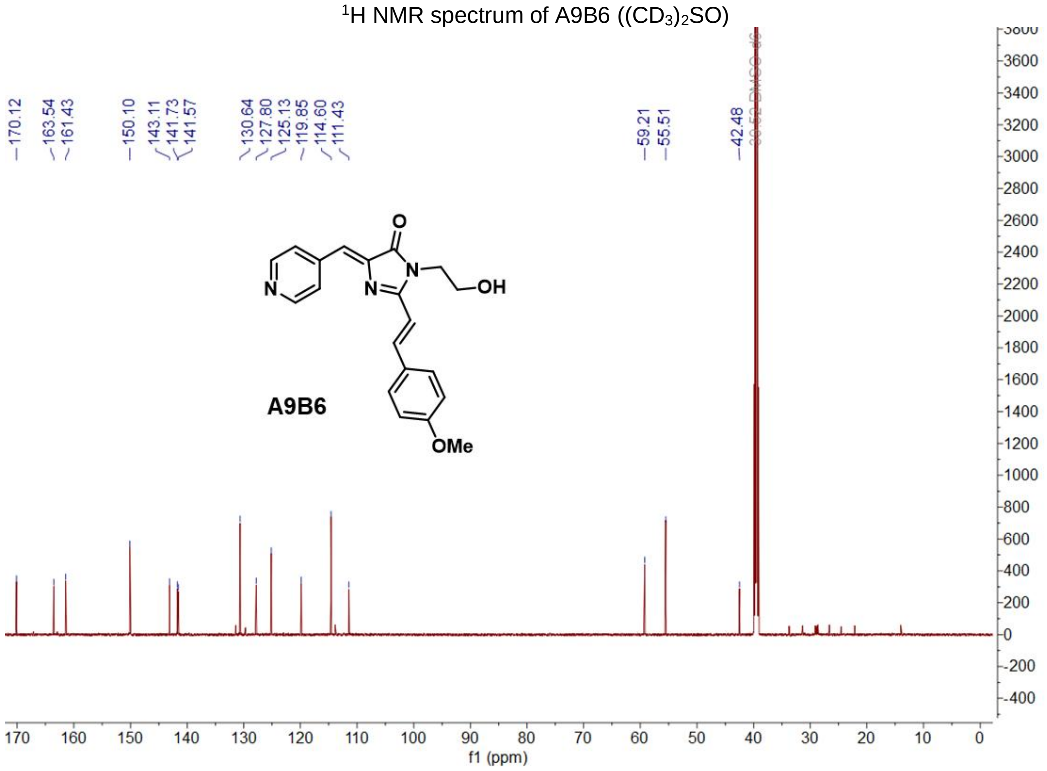

^{13}C NMR spectrum of A9B6 ($(CD_3)_2SO$)

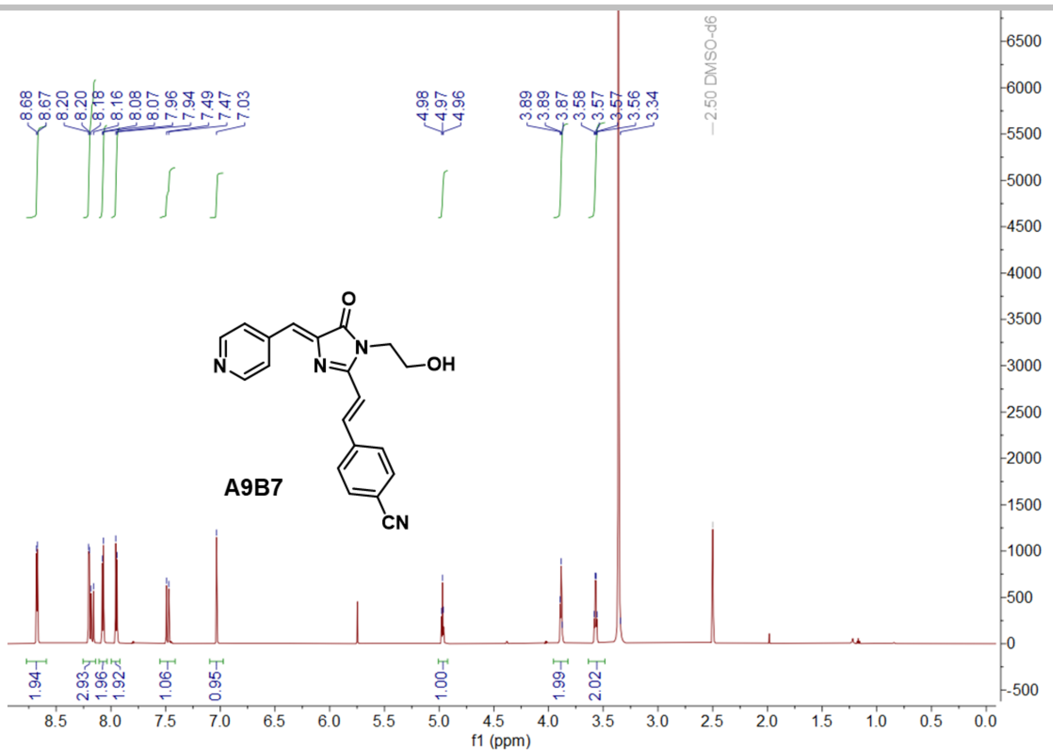

^{1}H NMR spectrum of A9B7 ($(CD_3)_2SO$)

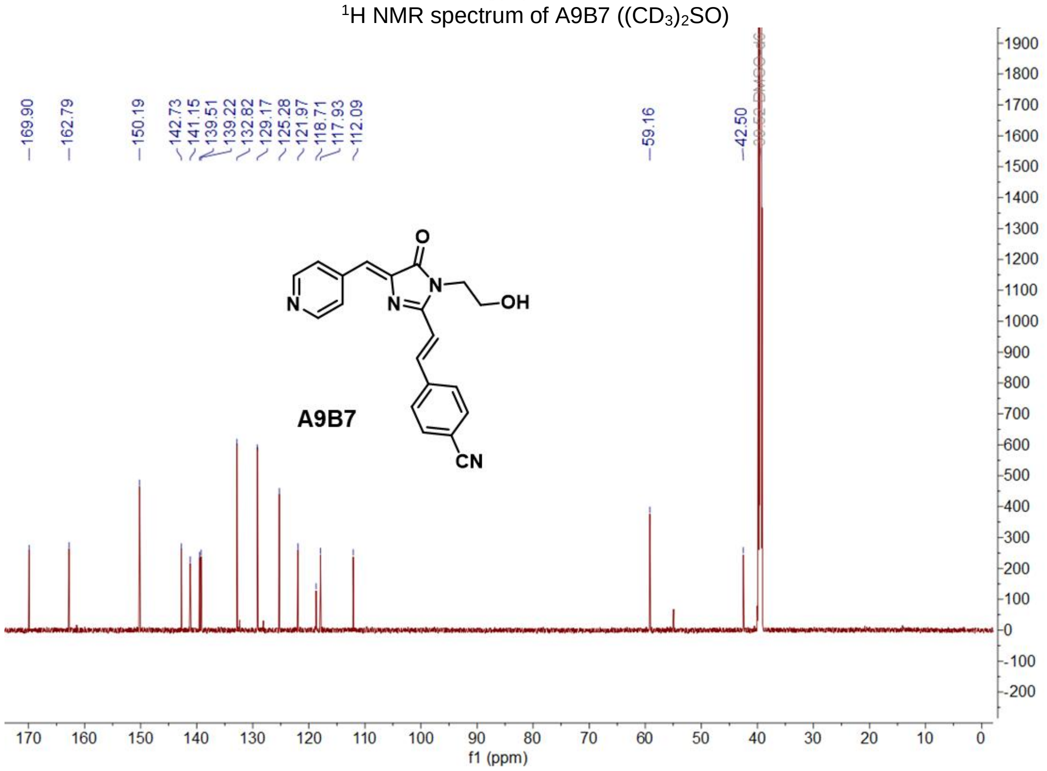

^{13}C NMR spectrum of A9B7 ($(CD_3)_2SO$)

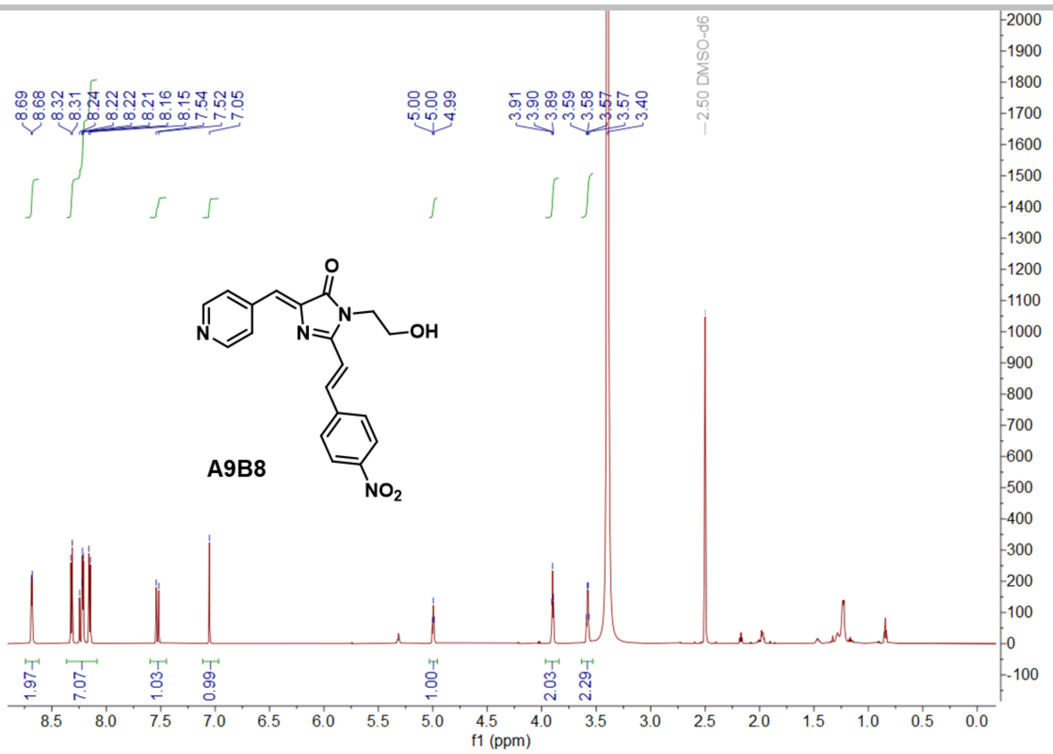

^1H NMR spectrum of A9B8 ($(CD_3)_2SO$)

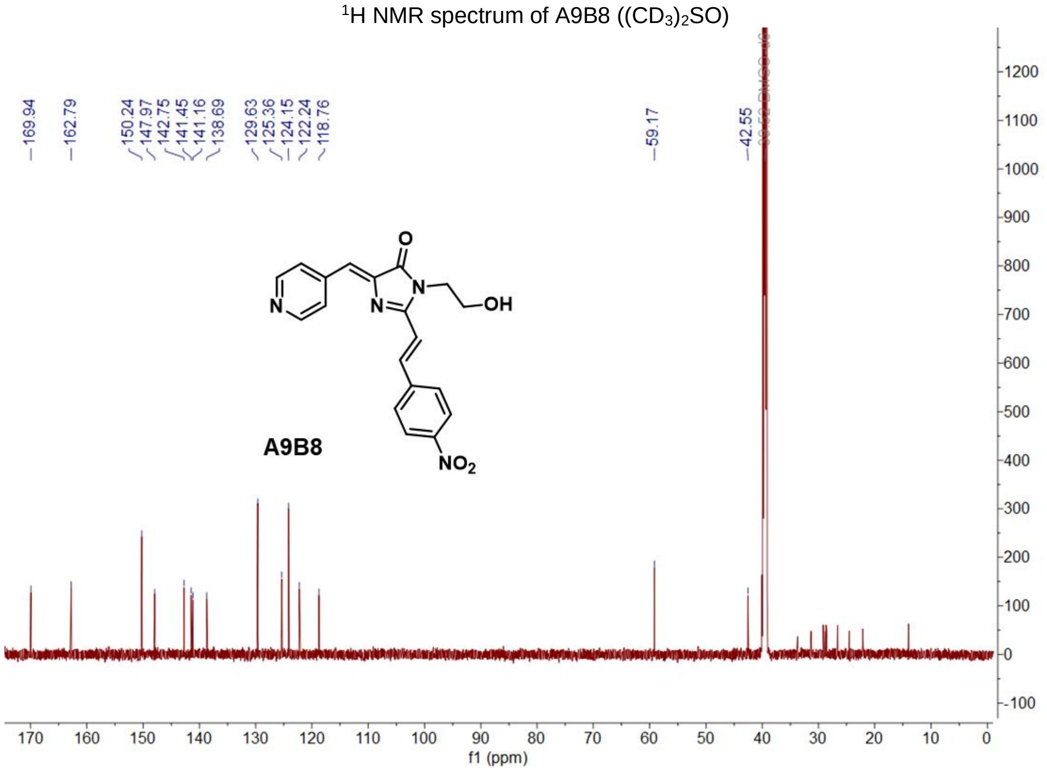

^{13}C NMR spectrum of A9B8 ($(CD_3)_2SO$)

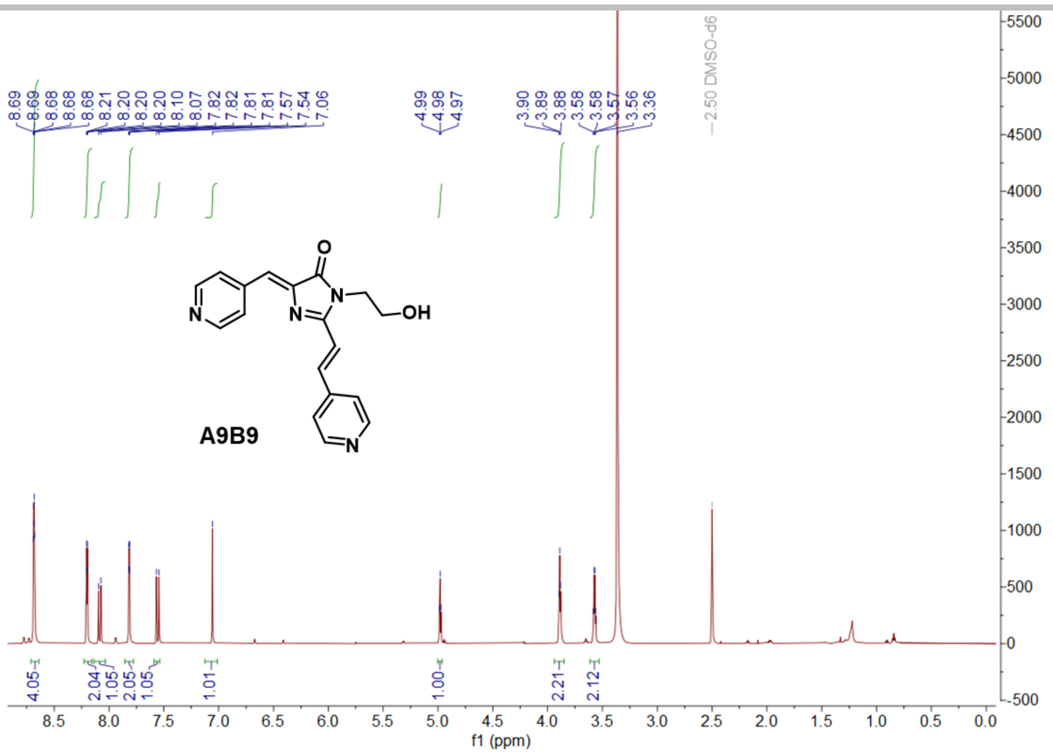

^{1}H NMR spectrum of A9B9 ($(CD_3)_2SO$)

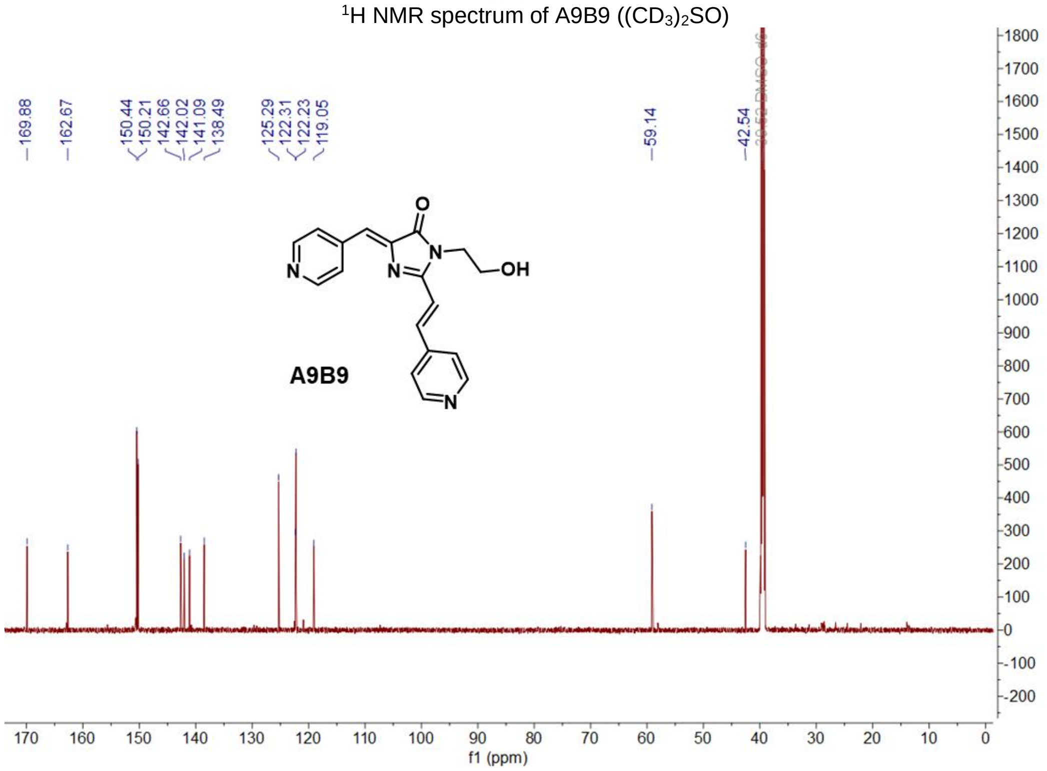

^{13}C NMR spectrum of A9B9 ($(CD_3)_2SO$)

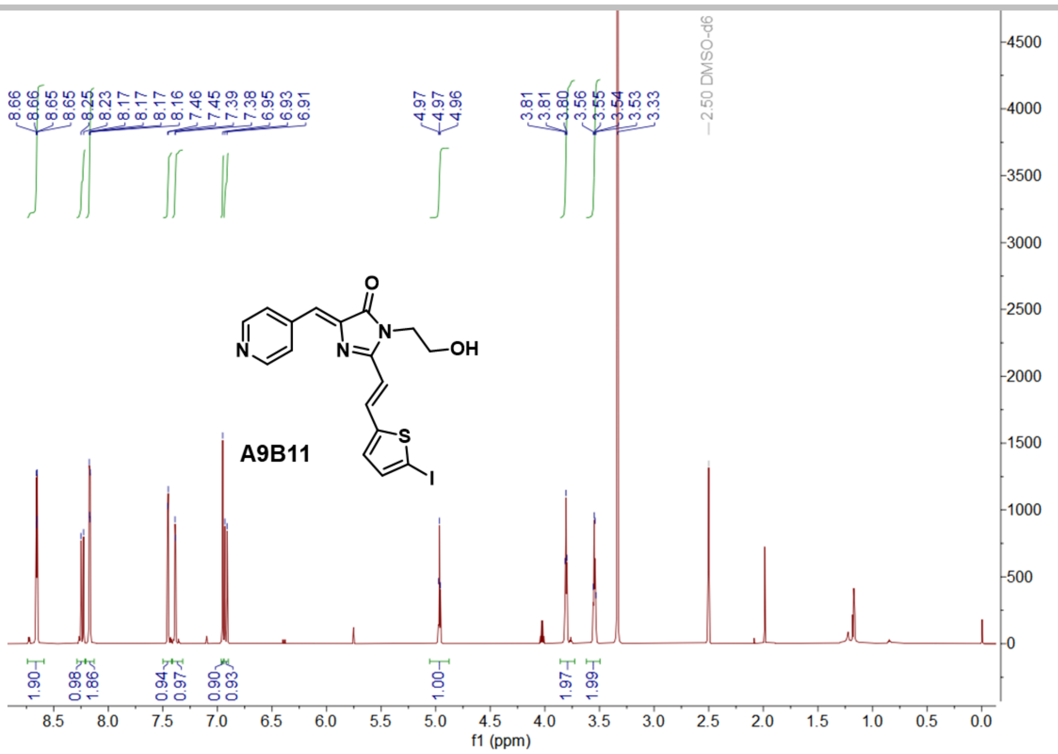

^{1}H NMR spectrum of A9B11 ((CD$_3$)$_2$SO)

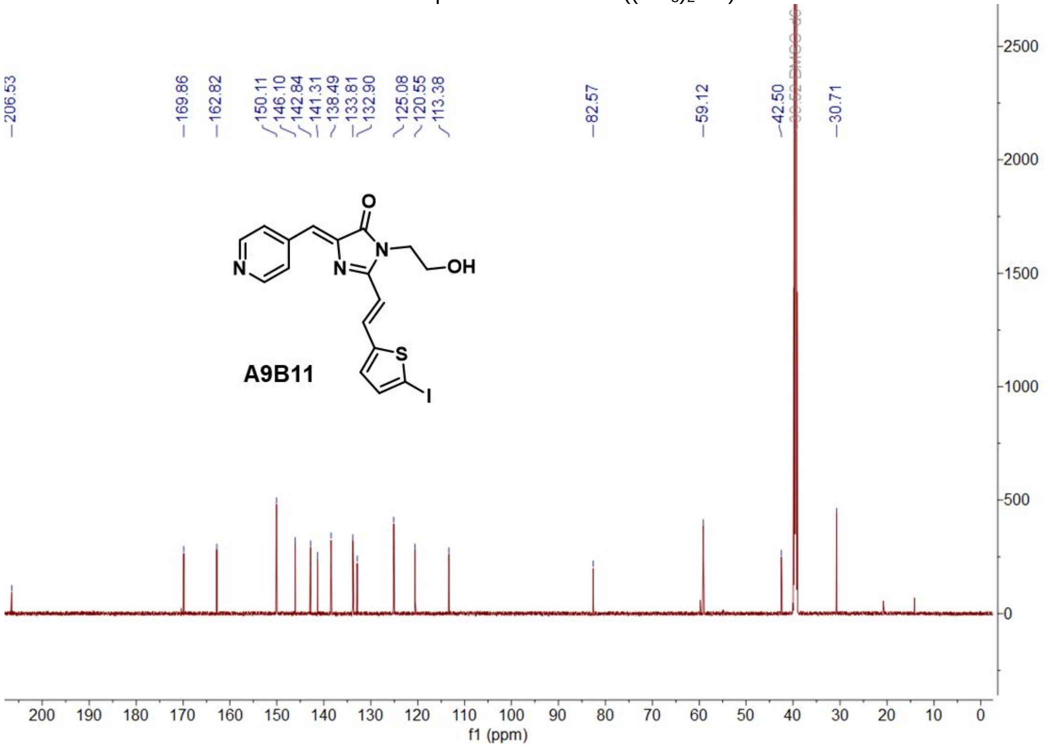

^{13}C NMR spectrum of A9B11 ((CD$_3$)$_2$SO)

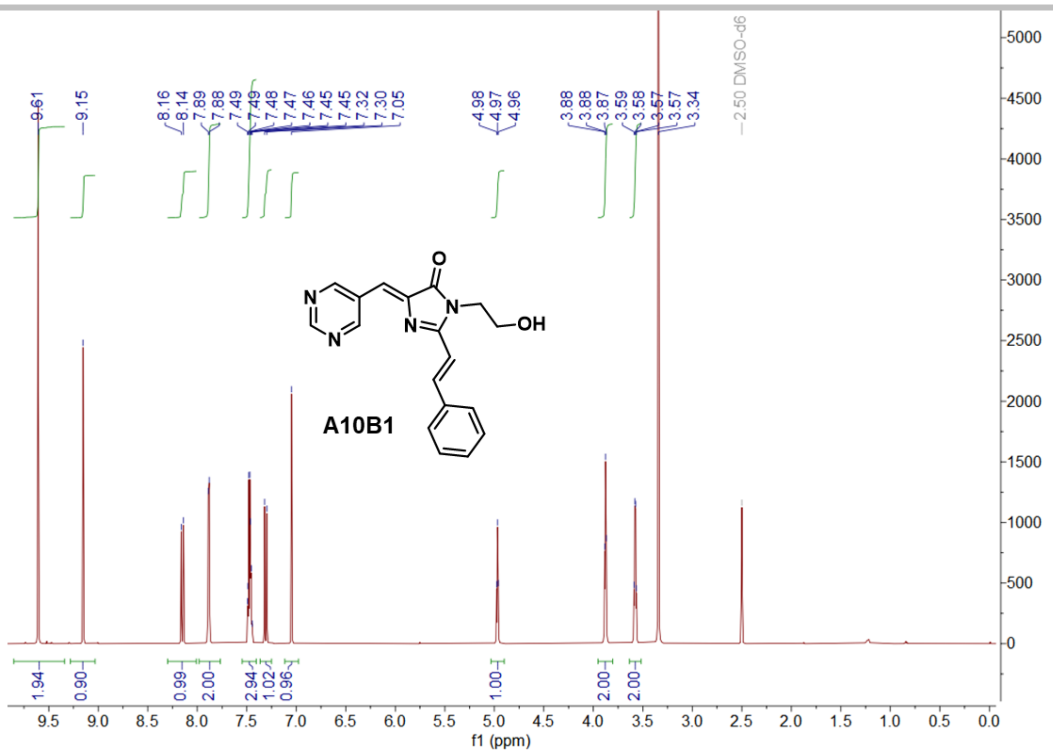

^{1}H NMR spectrum of A10B1 ($(CD_3)_2SO$)

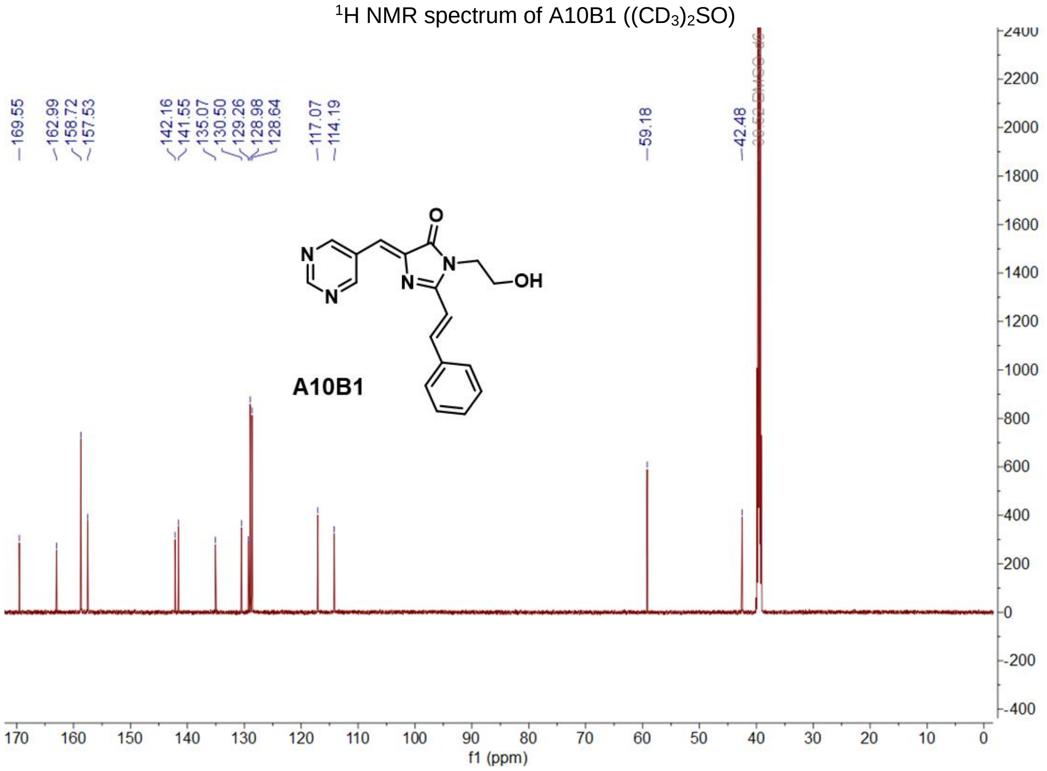

^{13}C NMR spectrum of A10B1 ($(CD_3)_2SO$)

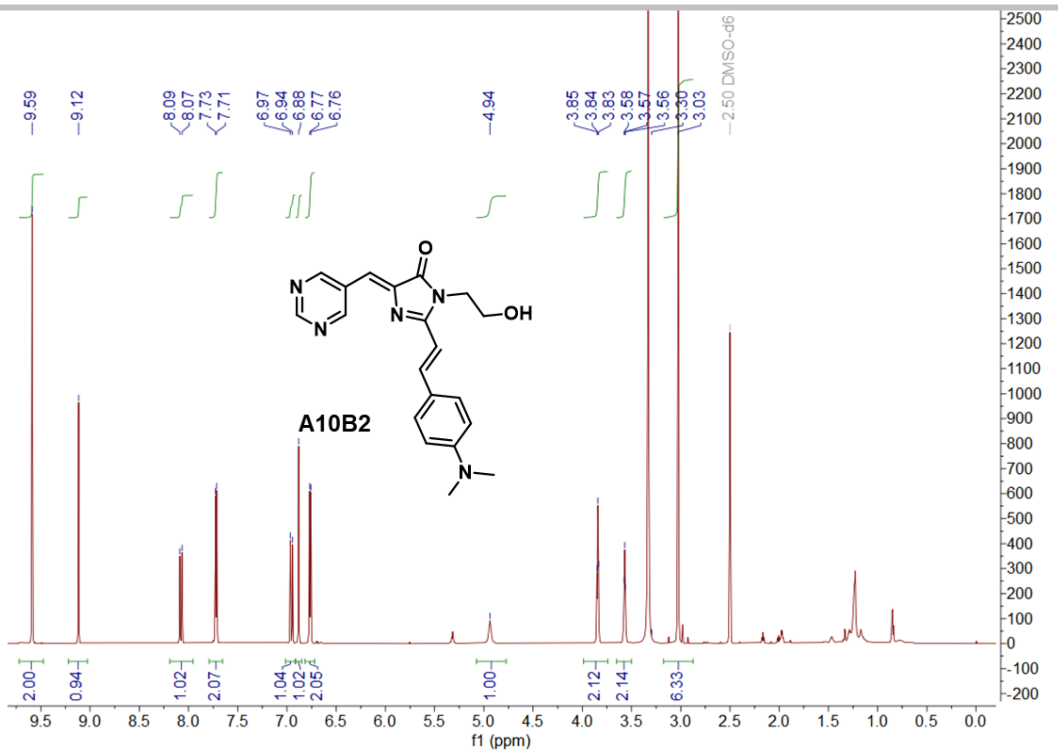

^{1}H NMR spectrum of A10B2 ($(CD_3)_2SO$)

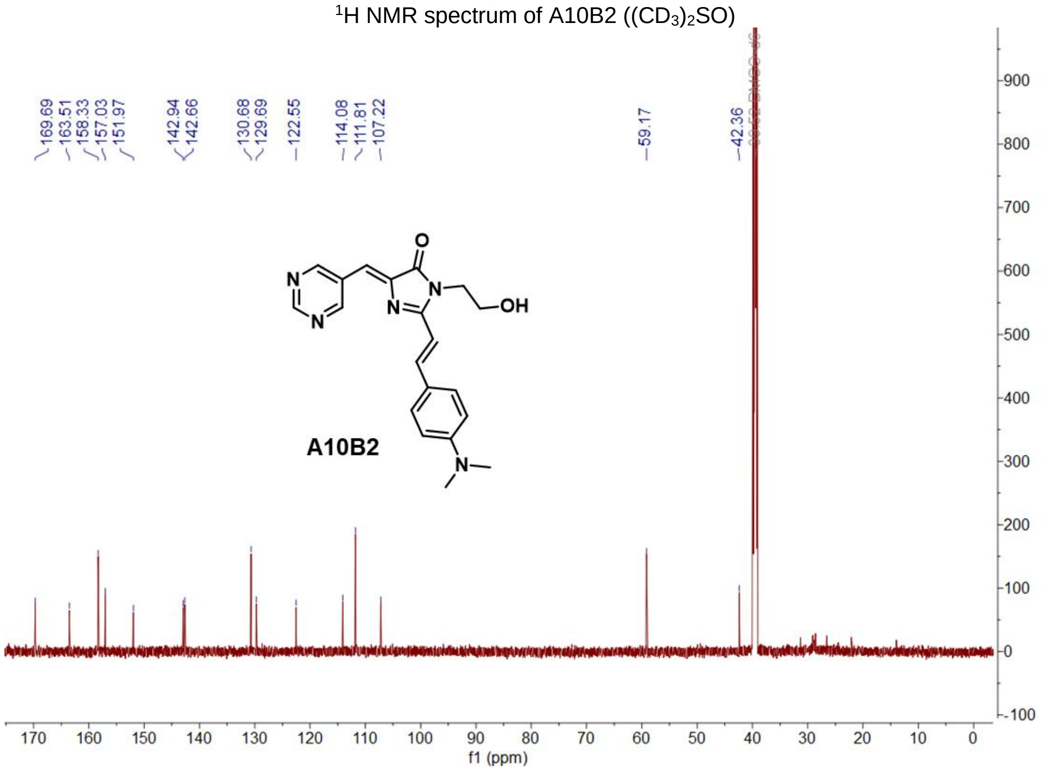

^{13}C NMR spectrum of A10B2 ($(CD_3)_2SO$)

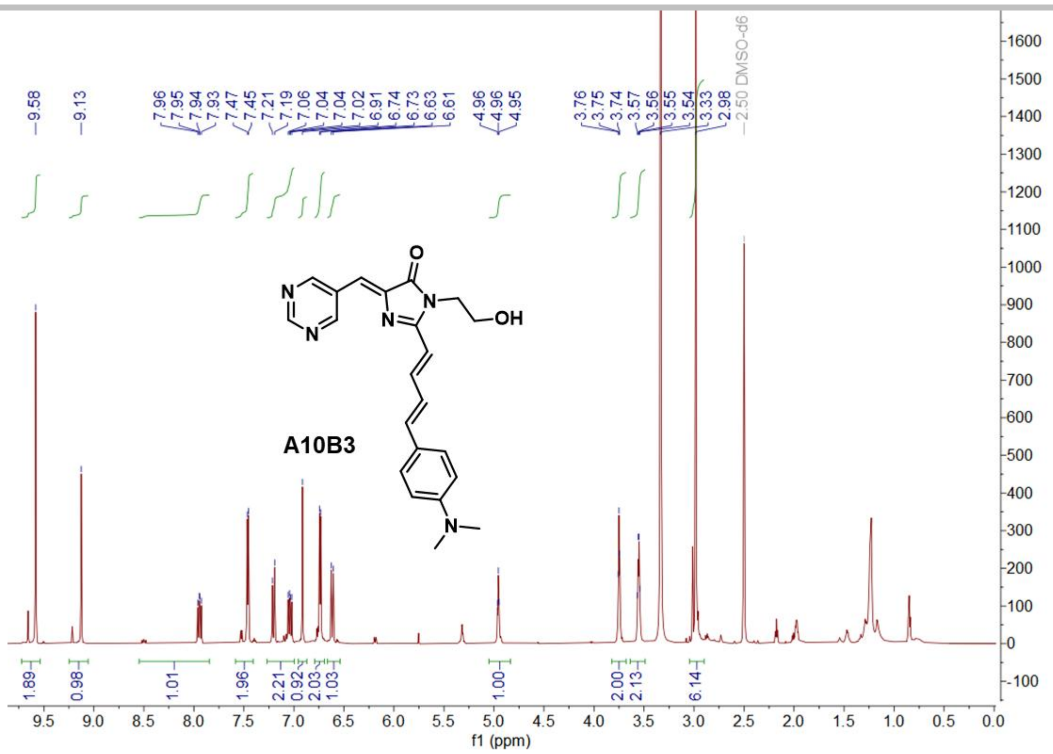

^{1}H NMR spectrum of A10B3 ($(CD_3)_2SO$)

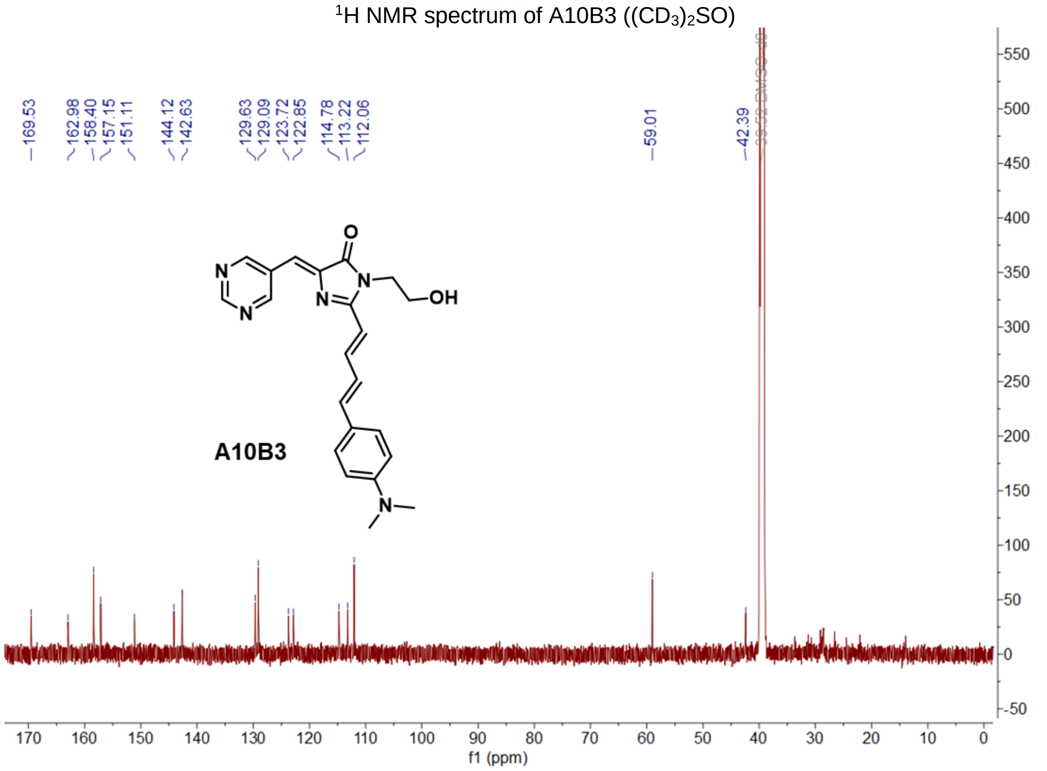

^{13}C NMR spectrum of A10B3 ($(CD_3)_2SO$)

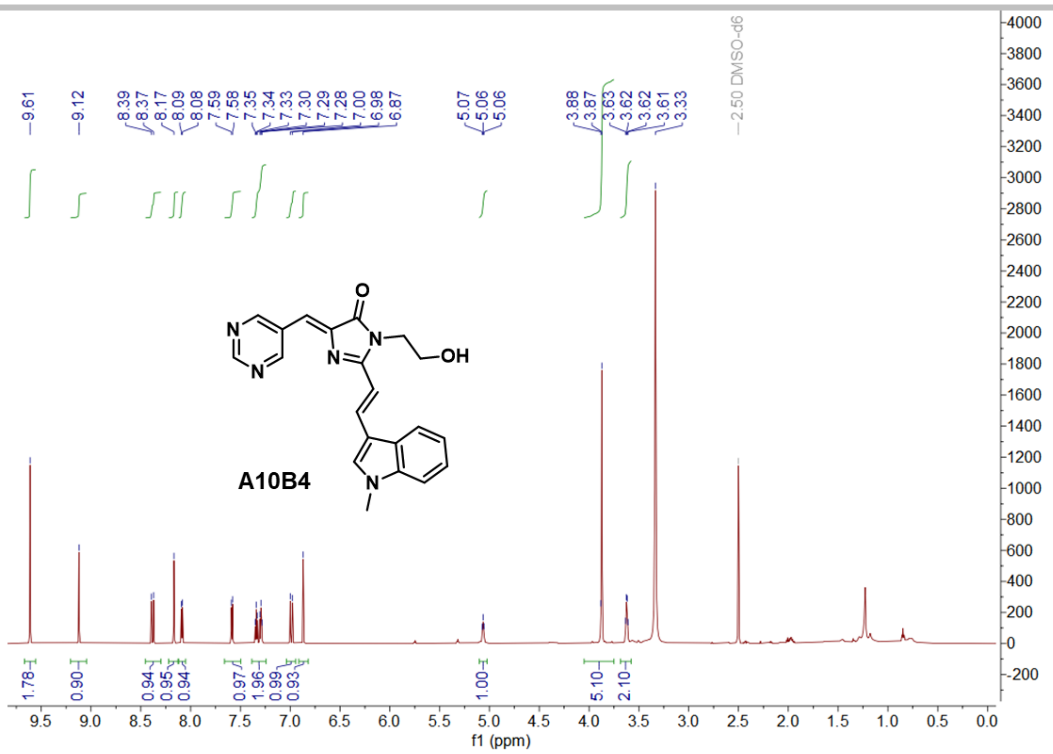

^{1}H NMR spectrum of A10B4 ($(CD_3)_2SO$)

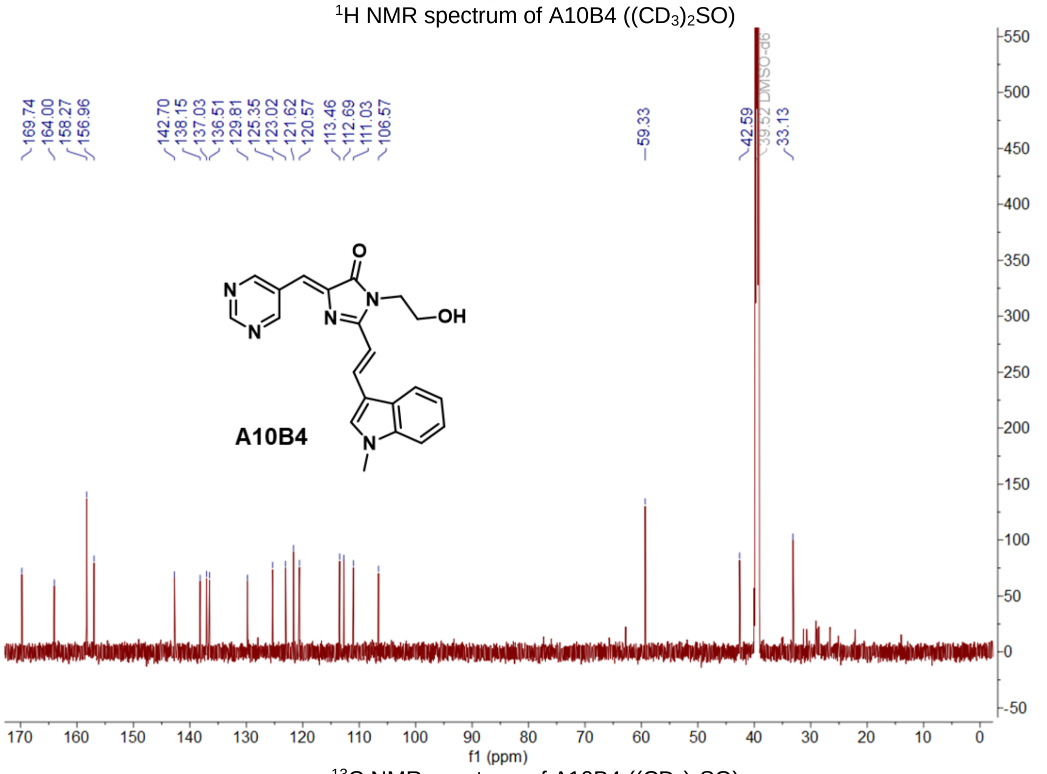

^{13}C NMR spectrum of A10B4 ($(CD_3)_2SO$)

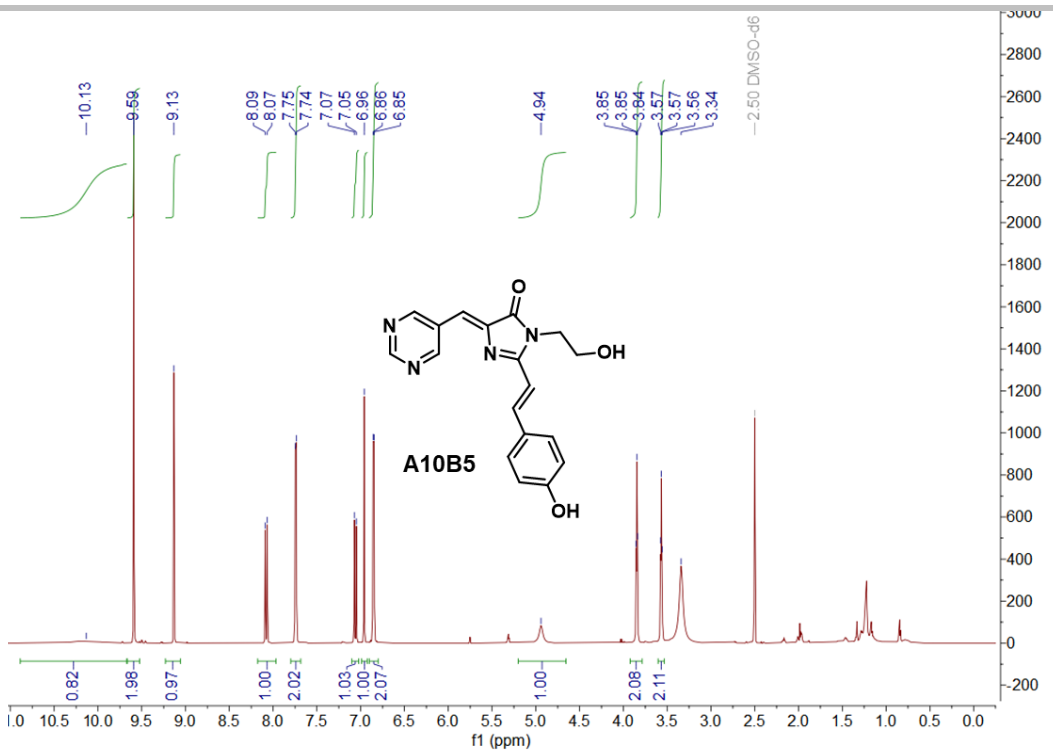

^1H NMR spectrum of A10B5 ($(CD_3)_2SO$)

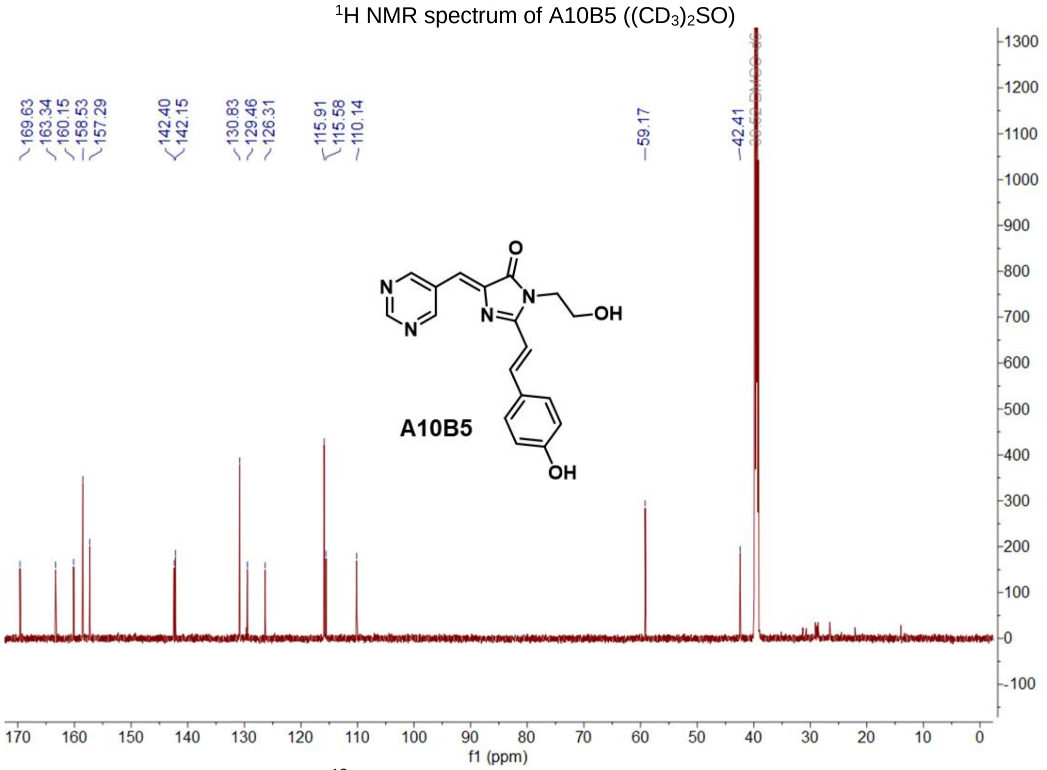

^{13}C NMR spectrum of A10B5 ($(CD_3)_2SO$)

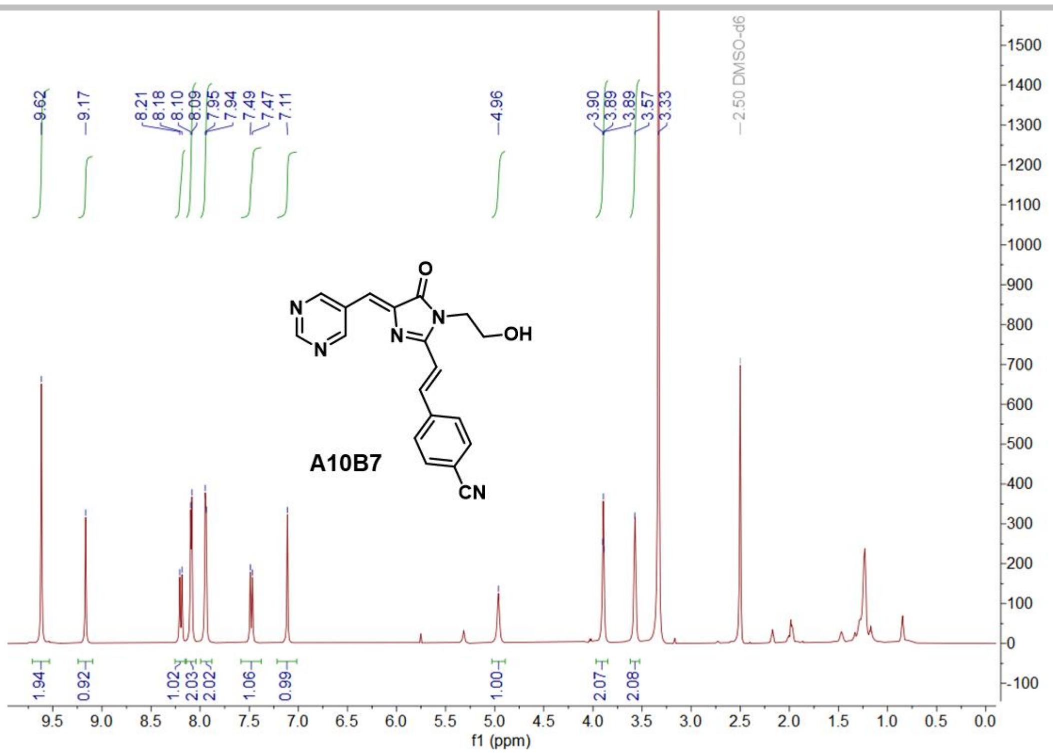

^{1}H NMR spectrum of A10B7 ($(CD_3)_2SO$)

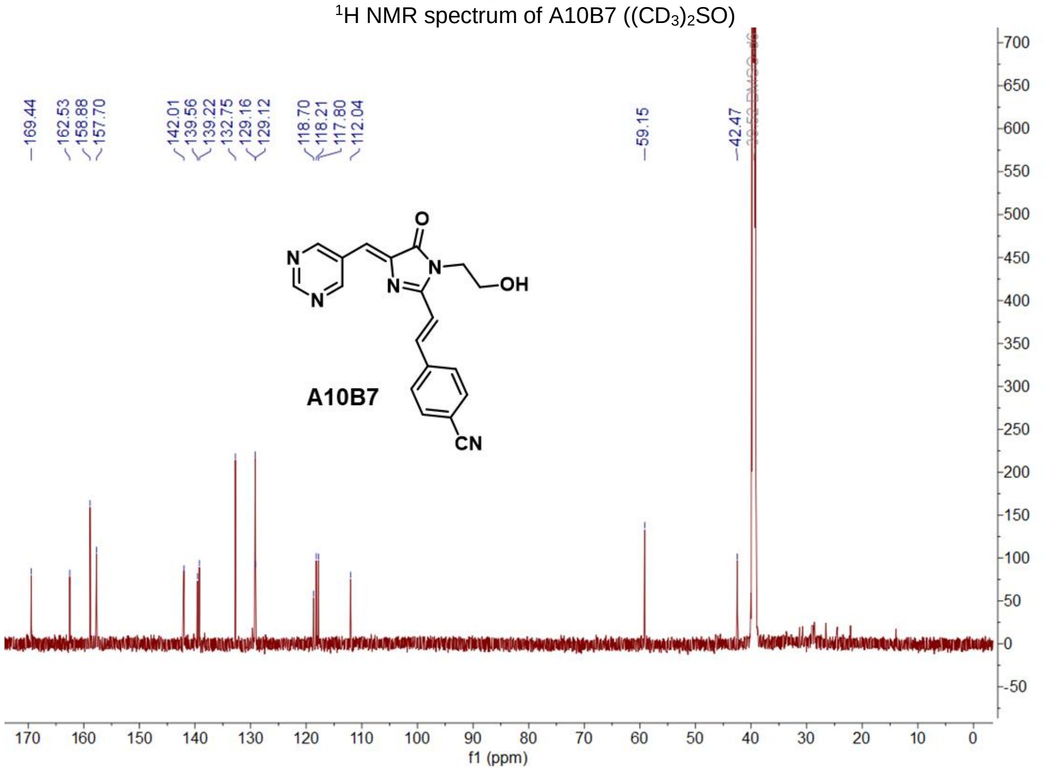

^{13}C NMR spectrum of A10B7 ($(CD_3)_2SO$)

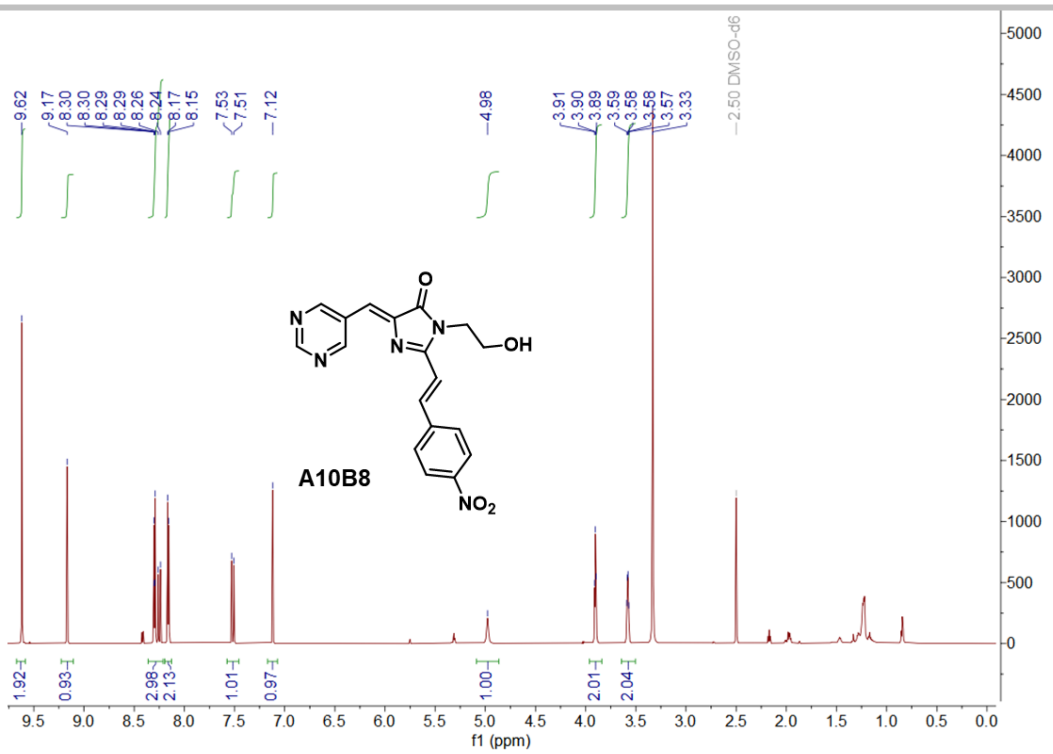

^1H NMR spectrum of A10B8 ($(CD_3)_2SO$)

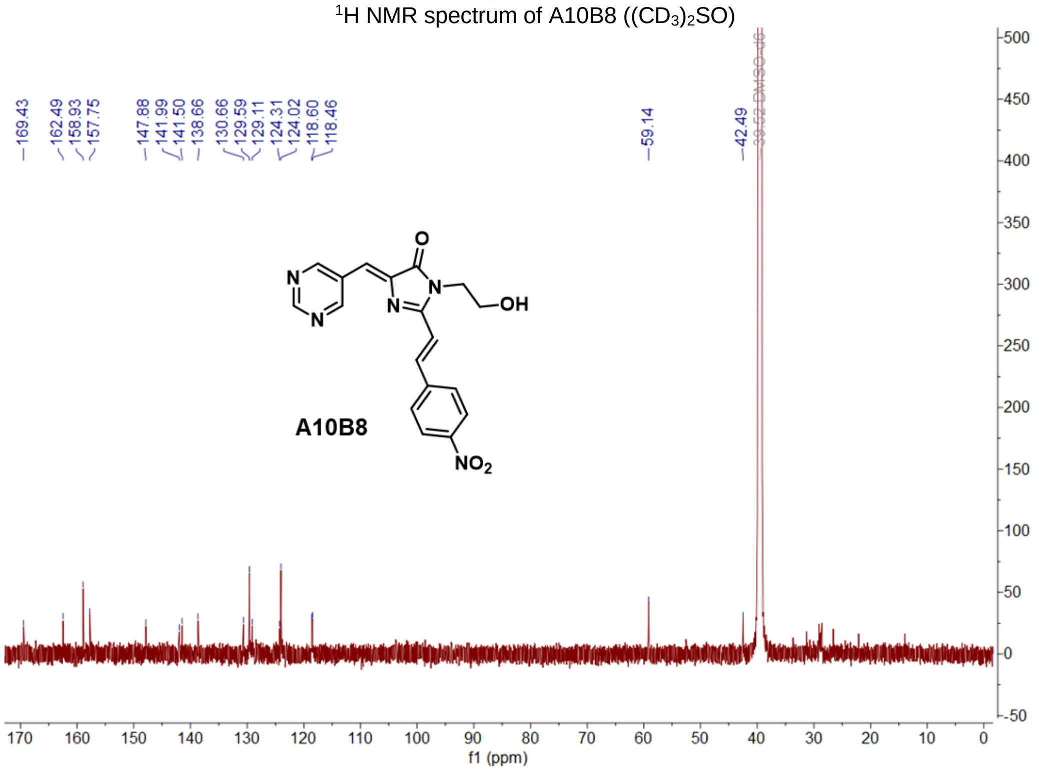

^{13}C NMR spectrum of A10B8 ($(CD_3)_2SO$)

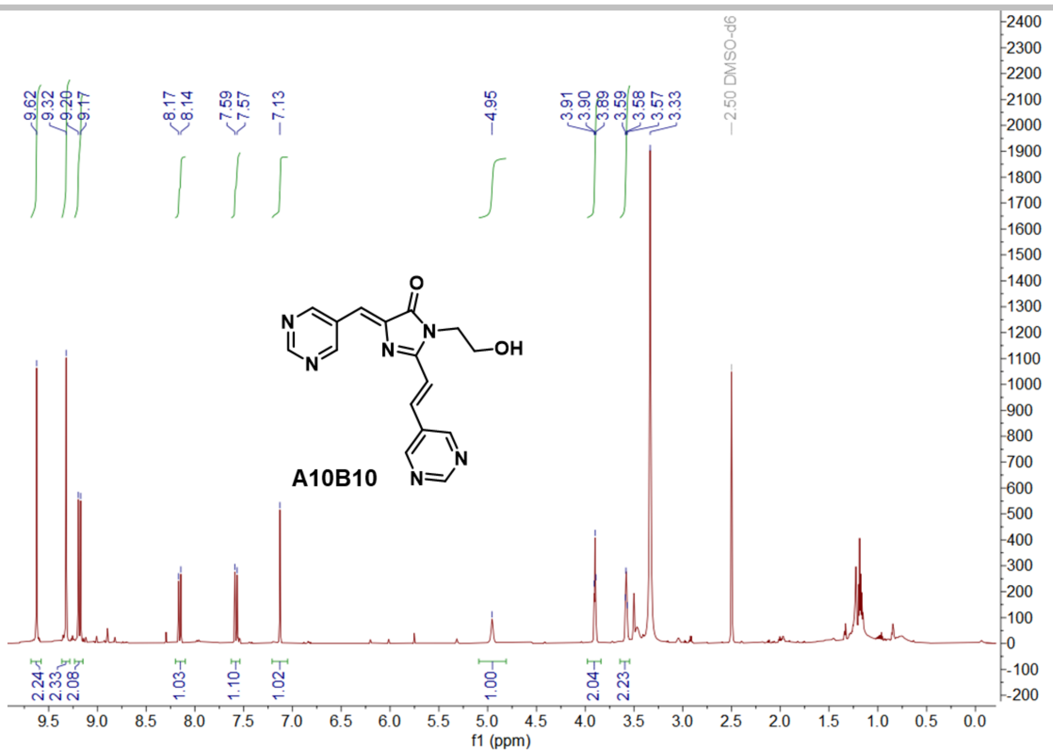

^{1}H NMR spectrum of A10B10 ($(CD_3)_2SO$)

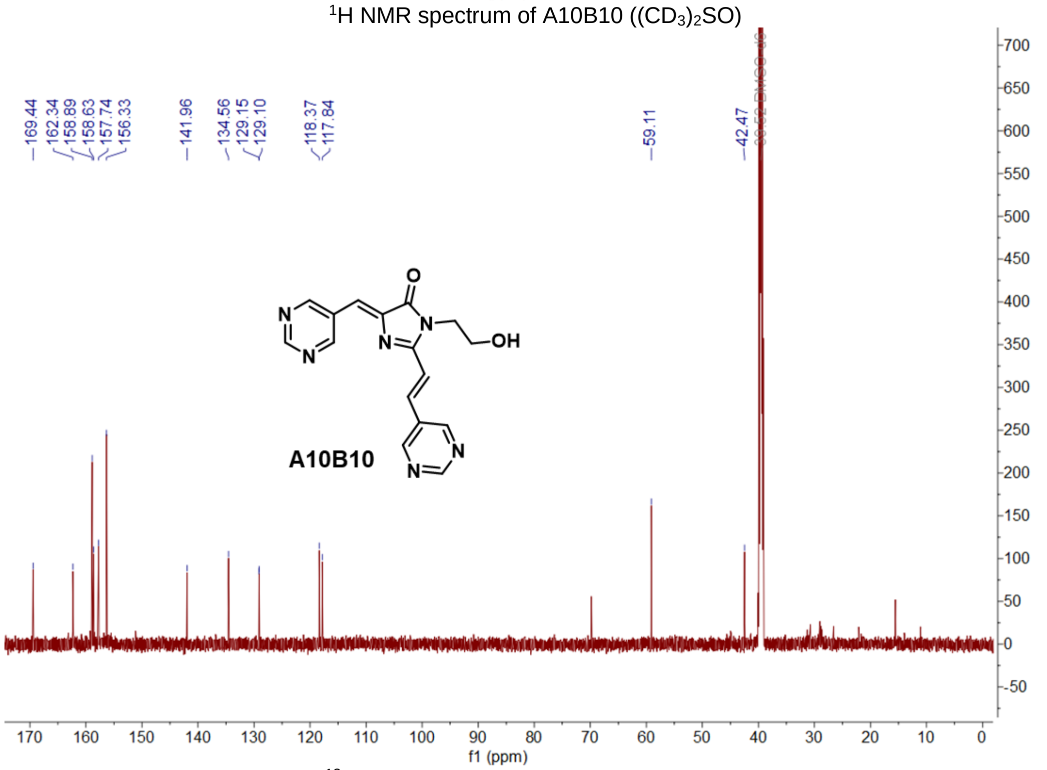

^{13}C NMR spectrum of A10B10 ($(CD_3)_2SO$)

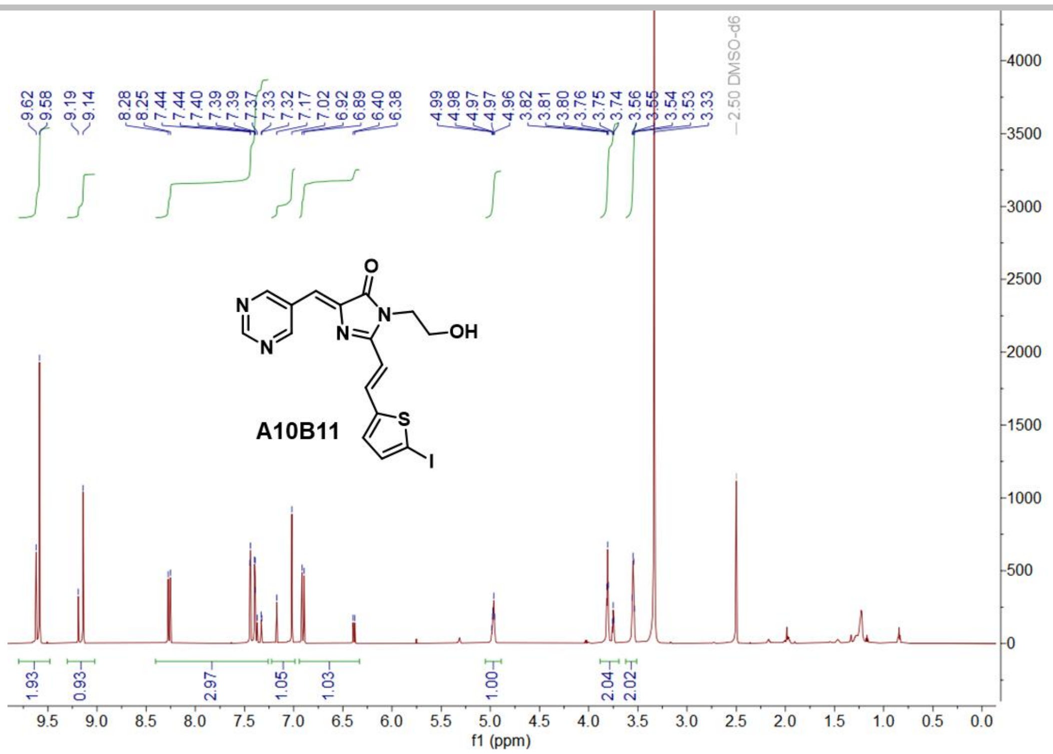

^1H NMR spectrum of A10B11 ($(CD_3)_2SO$)

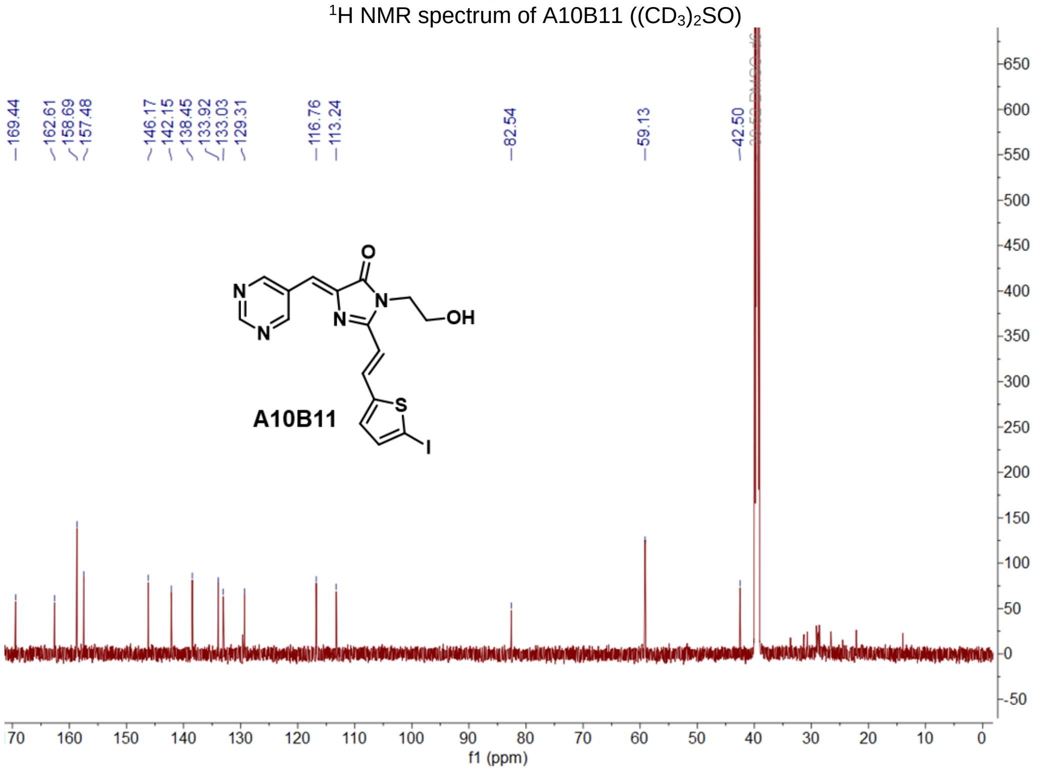

^{13}C NMR spectrum of A10B11 ($(CD_3)_2SO$)

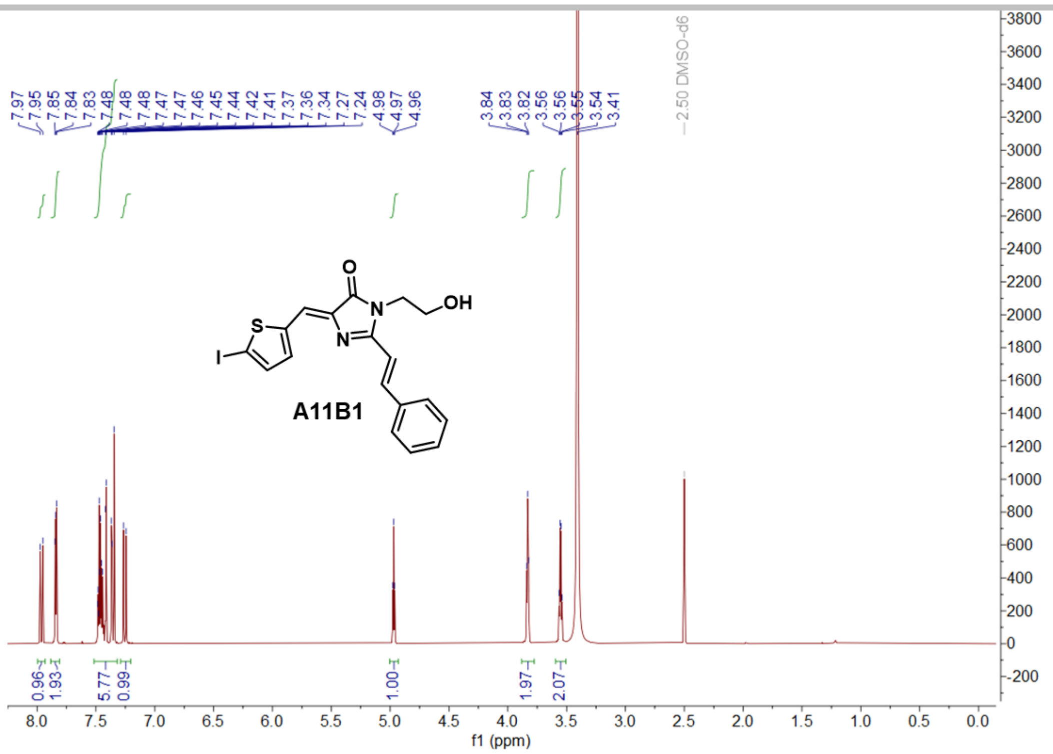

^{1}H NMR spectrum of A11B1 ($(CD_3)_2SO$)

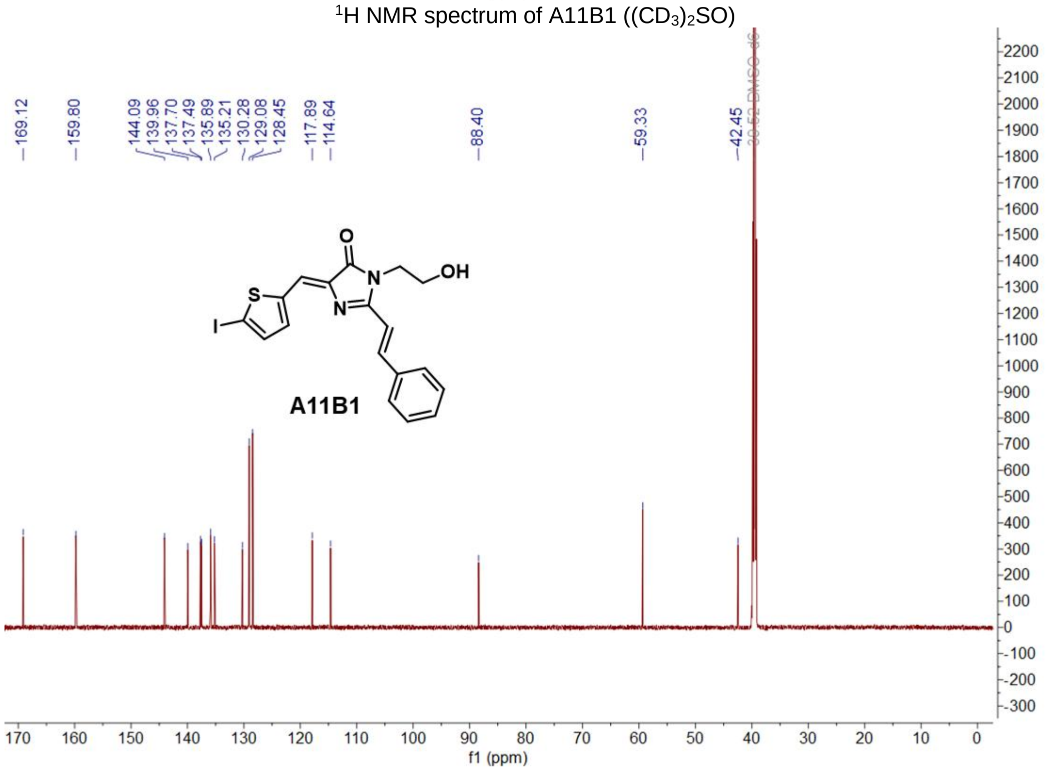

^{13}C NMR spectrum of A11B1 ($(CD_3)_2SO$)

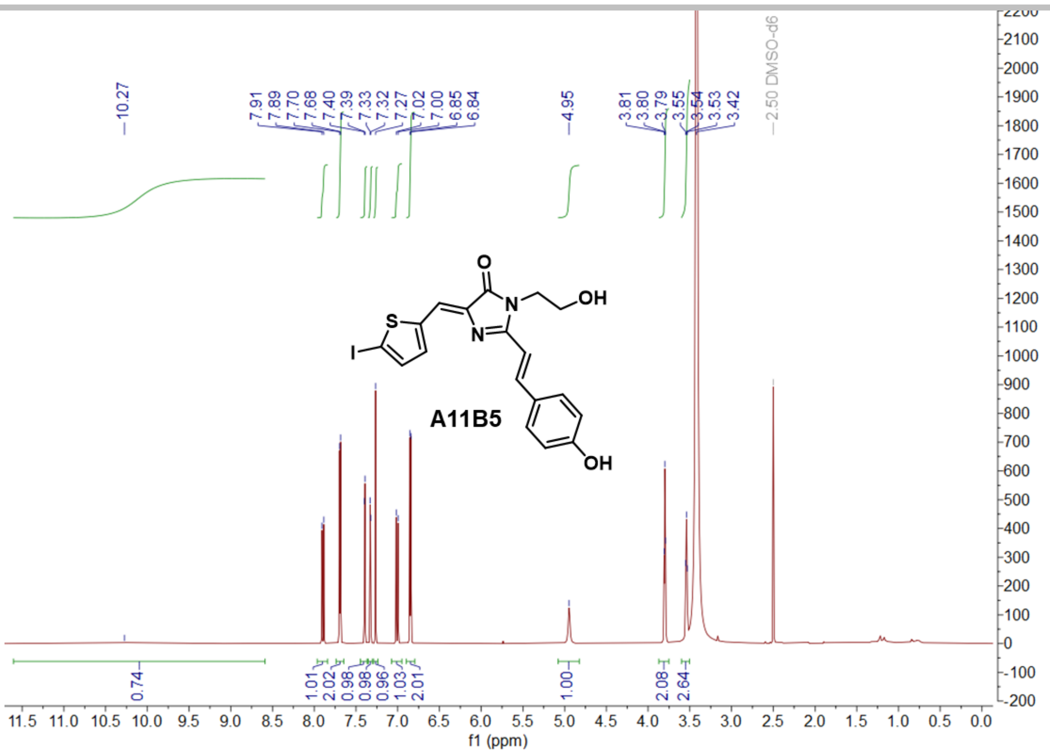

^{1}H NMR spectrum of A11B5 ($(CD_3)_2SO$)

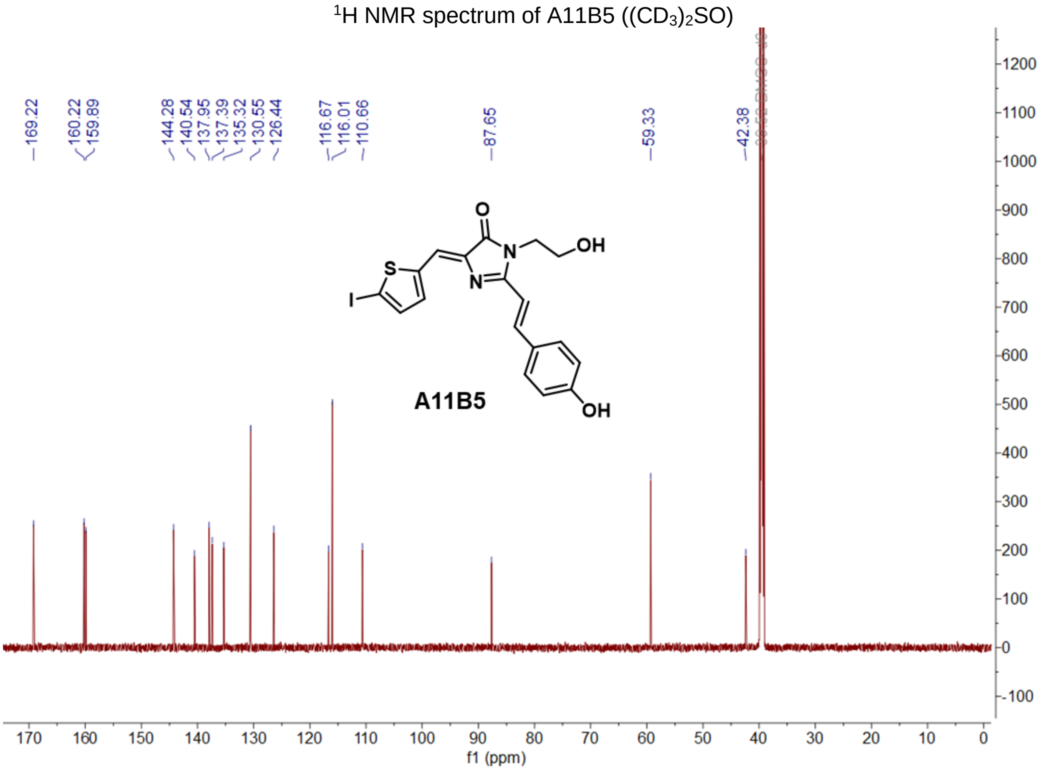

^{13}C NMR spectrum of A11B5 ($(CD_3)_2SO$)

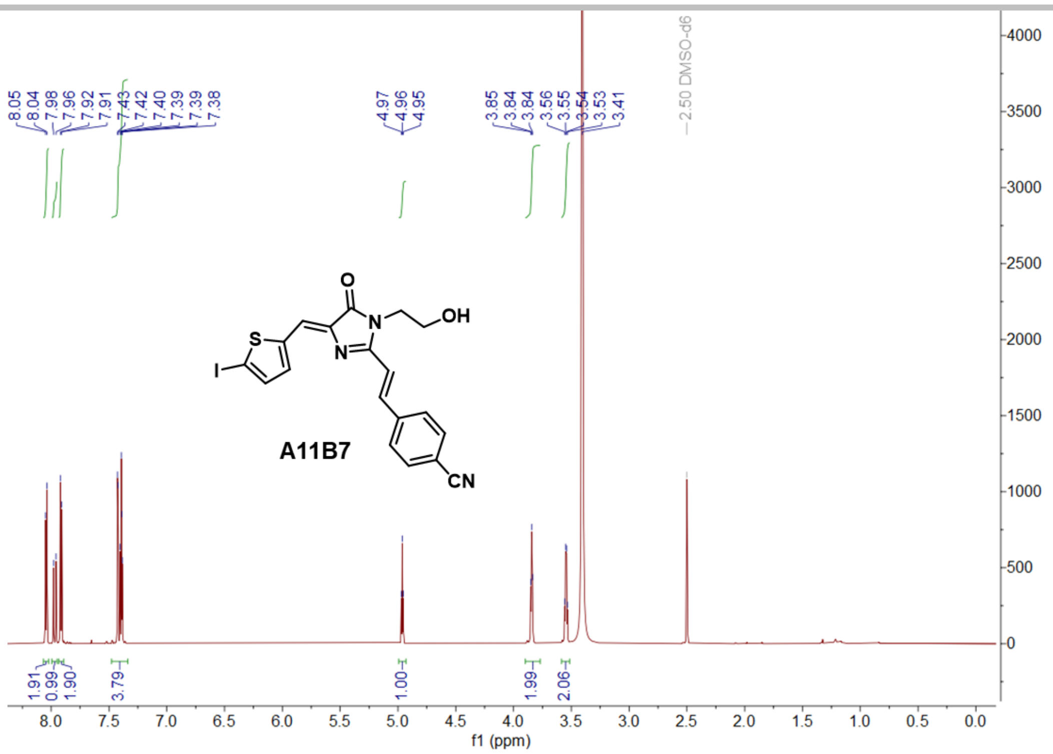

^{1}H NMR spectrum of A11B7 ($(CD_3)_2SO$)

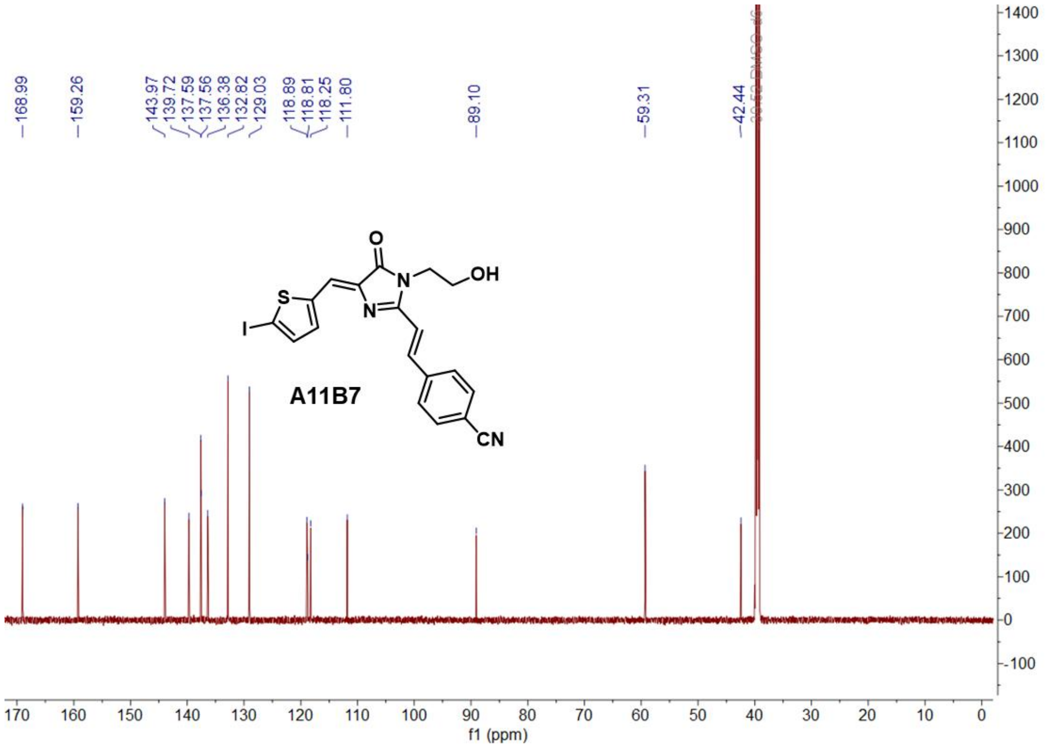

^{13}C NMR spectrum of A11B7 ($(CD_3)_2SO$)

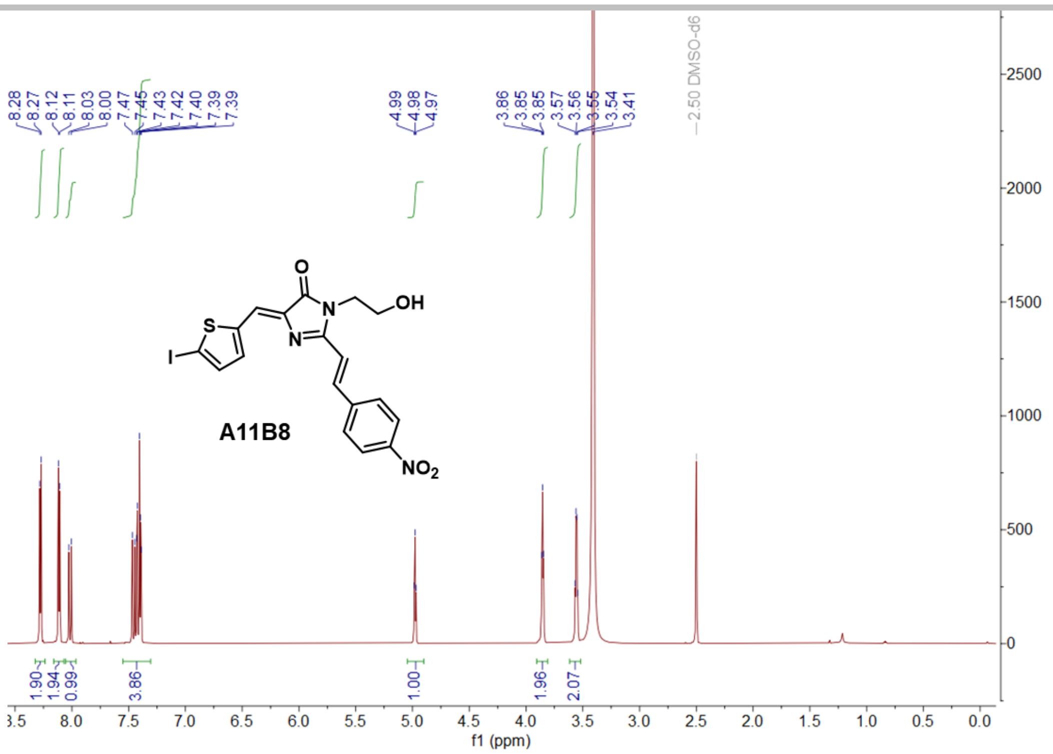

^{1}H NMR spectrum of A11B8 ($(CD_3)_2SO$)

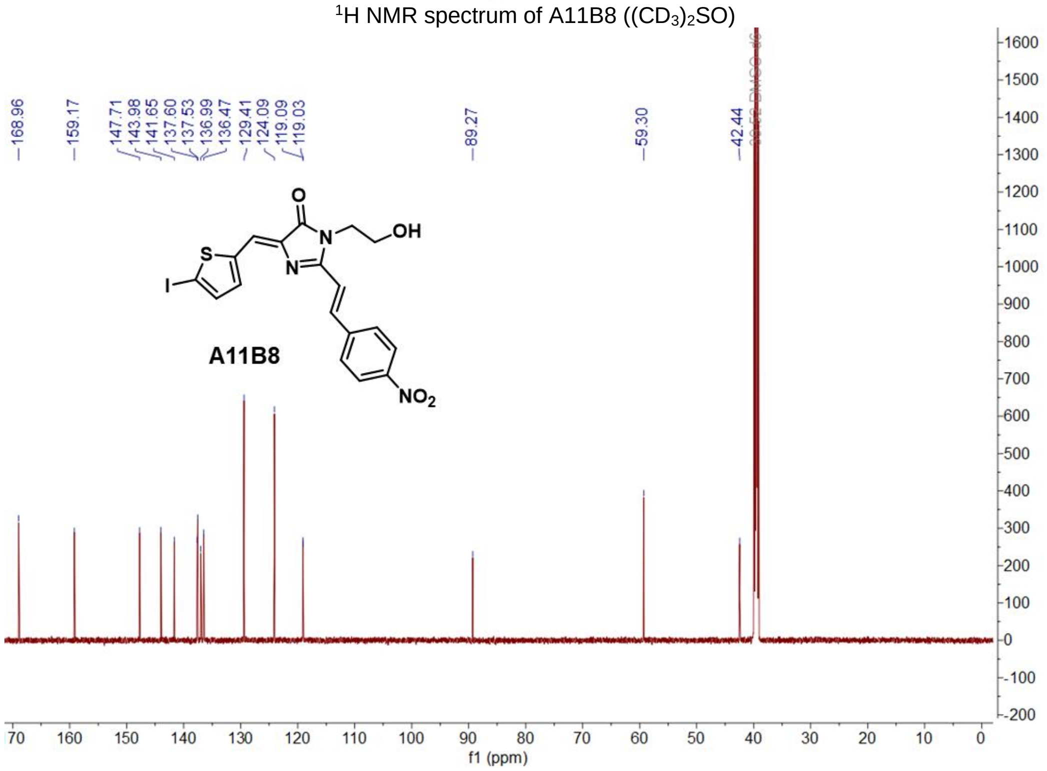

^{13}C NMR spectrum of A11B8 ($(CD_3)_2SO$)

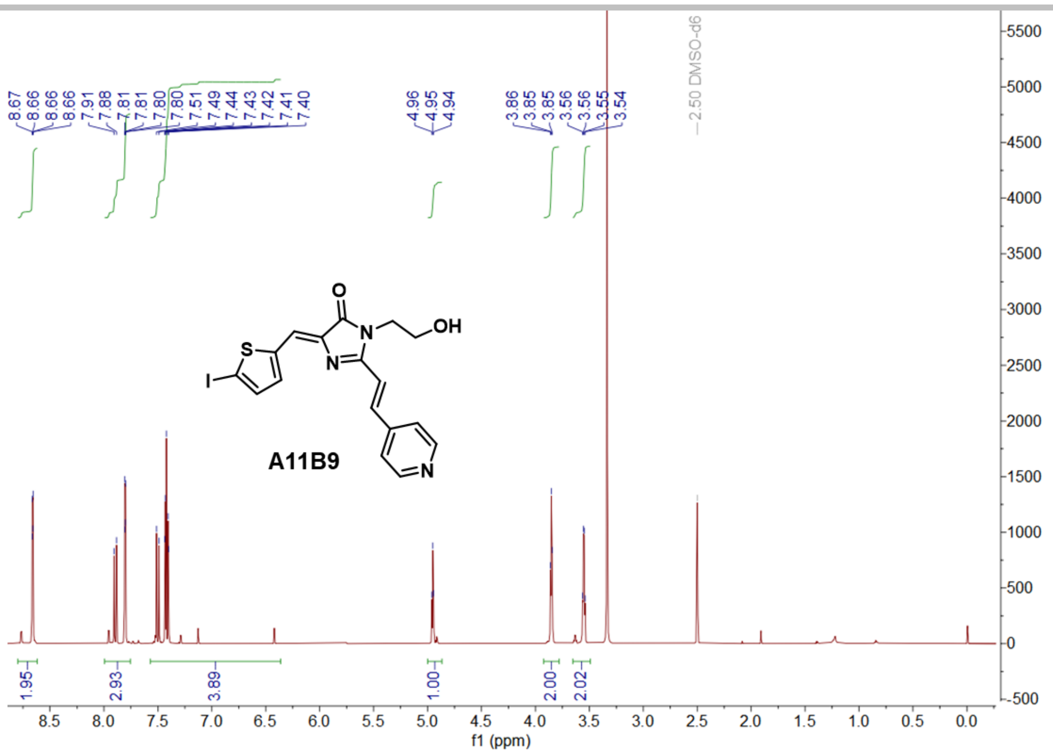

^{1}H NMR spectrum of A11B9 ($(CD_3)_2SO$)

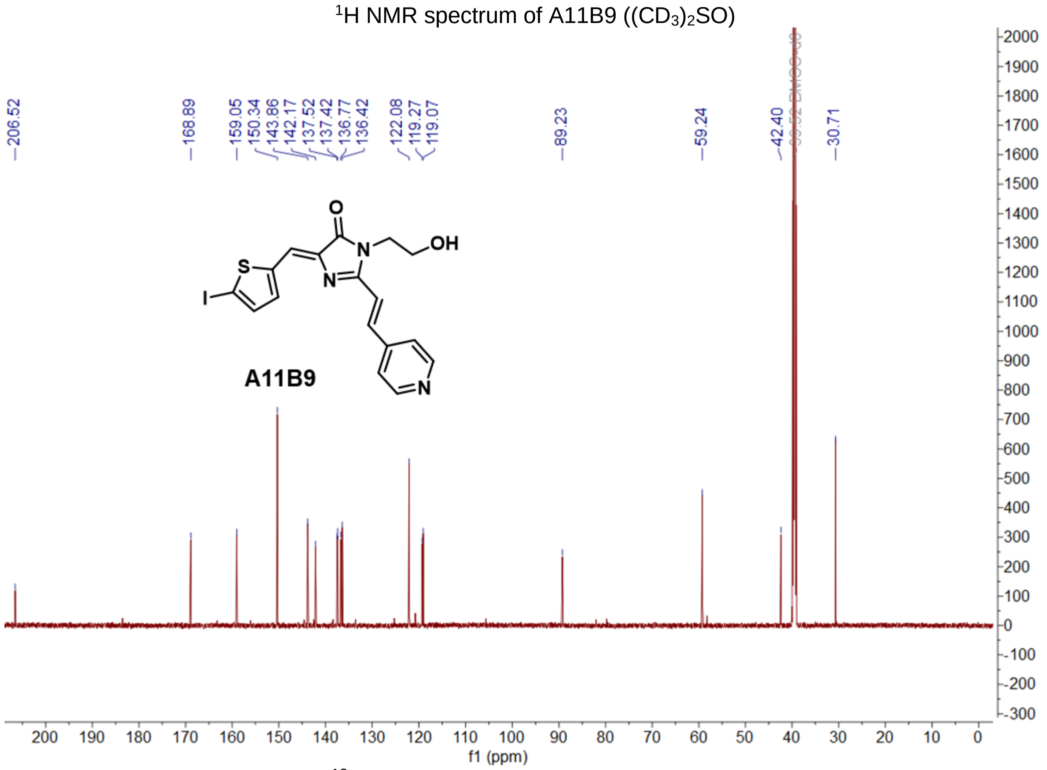

^{13}C NMR spectrum of A11B9 ($(CD_3)_2SO$)

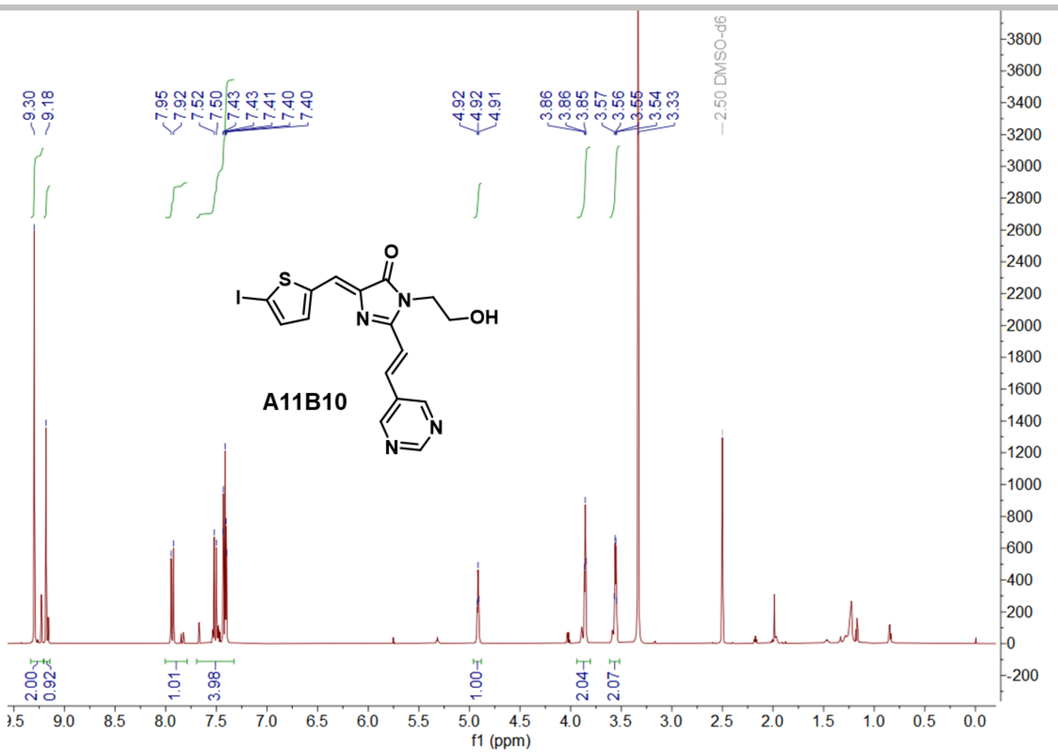

^{1}H NMR spectrum of A11B10 ($(CD_3)_2SO$)

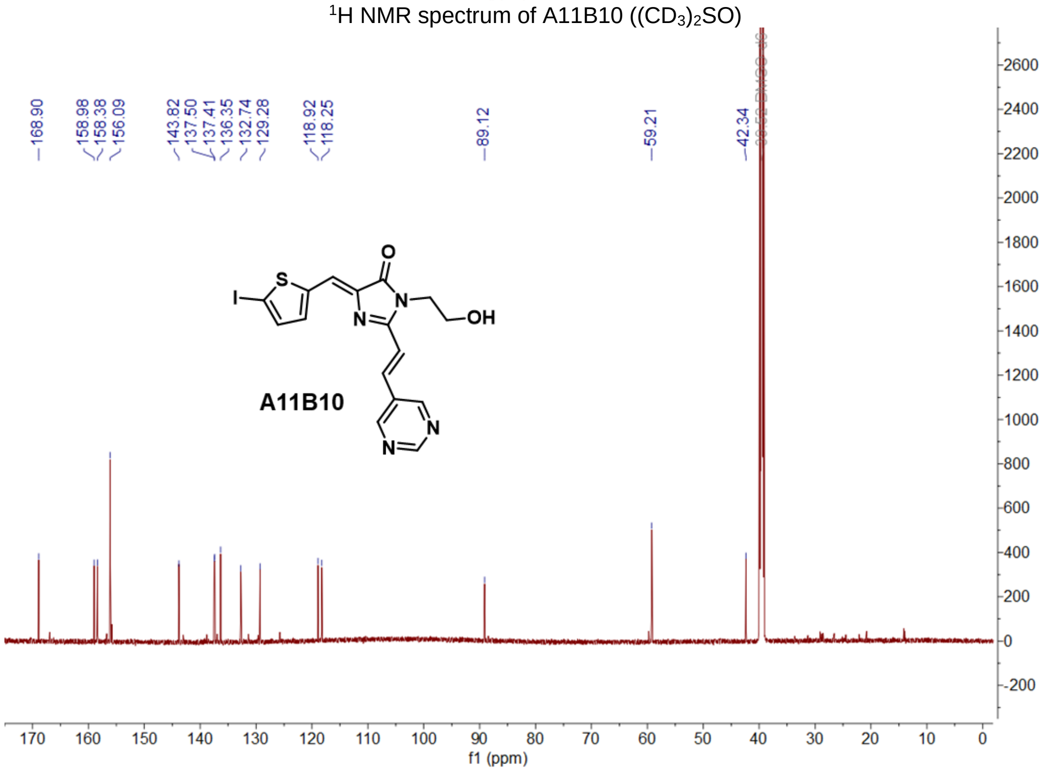

^{13}C NMR spectrum of A11B10 ($(CD_3)_2SO$)

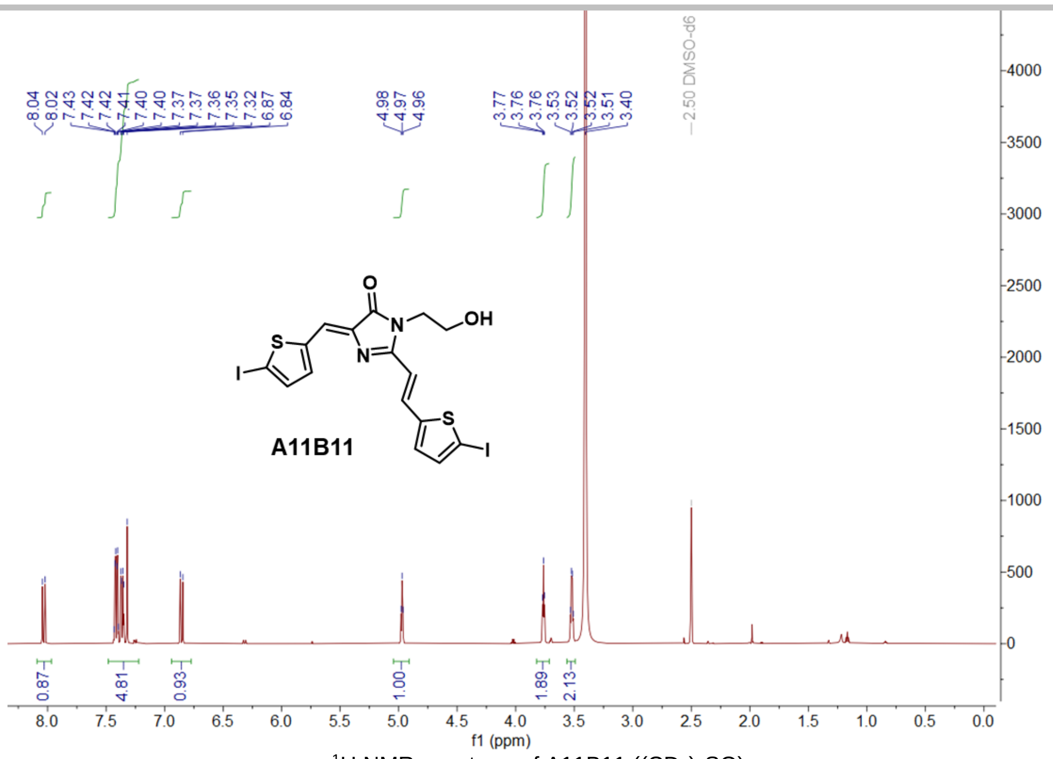

^{1}H NMR spectrum of A11B11 ($(CD_3)_2SO$)

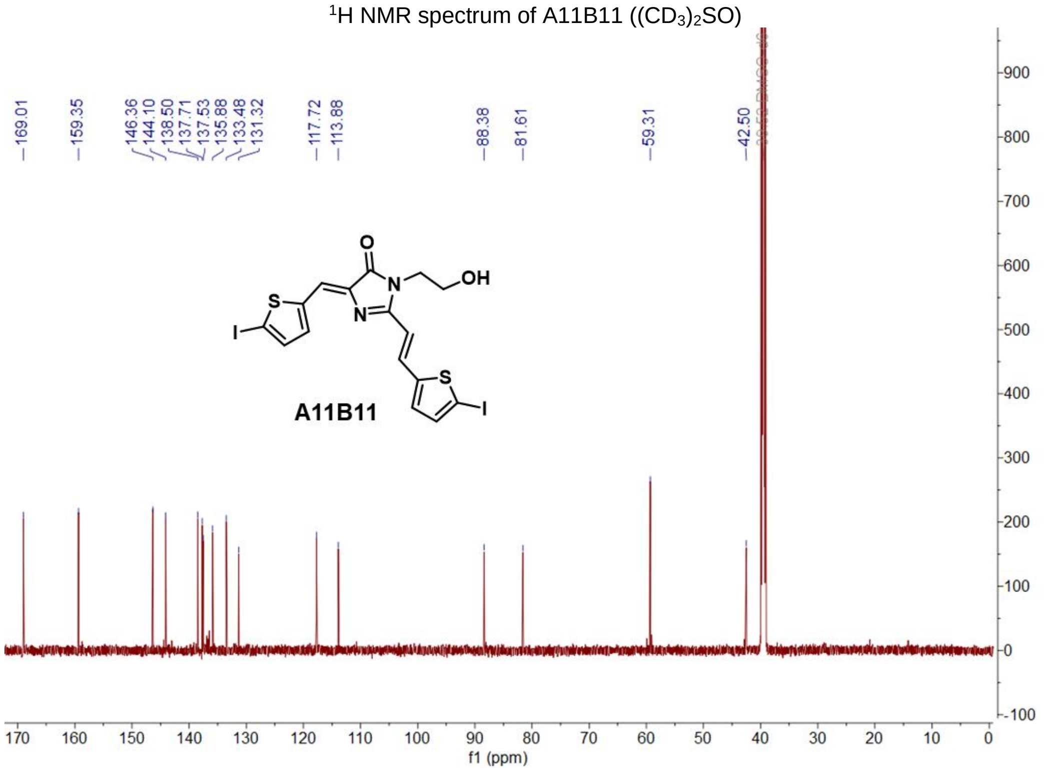

^{13}C NMR spectrum of A11B11 ($(CD_3)_2SO$)

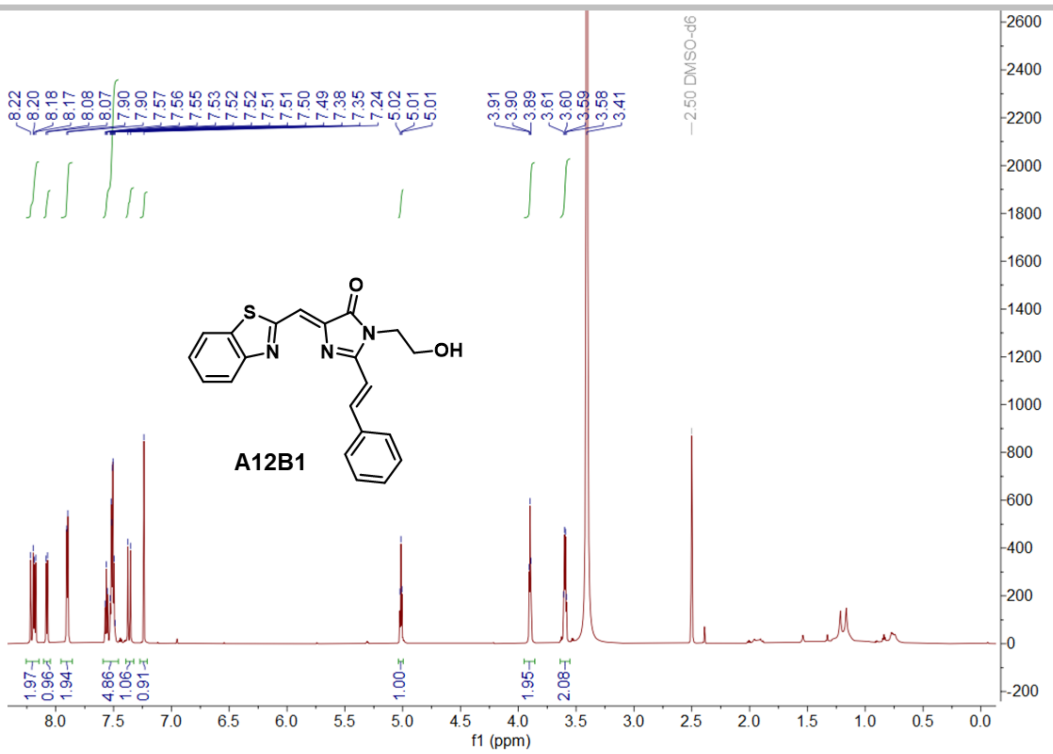

^{1}H NMR spectrum of A12B1 ($(CD_3)_2SO$)

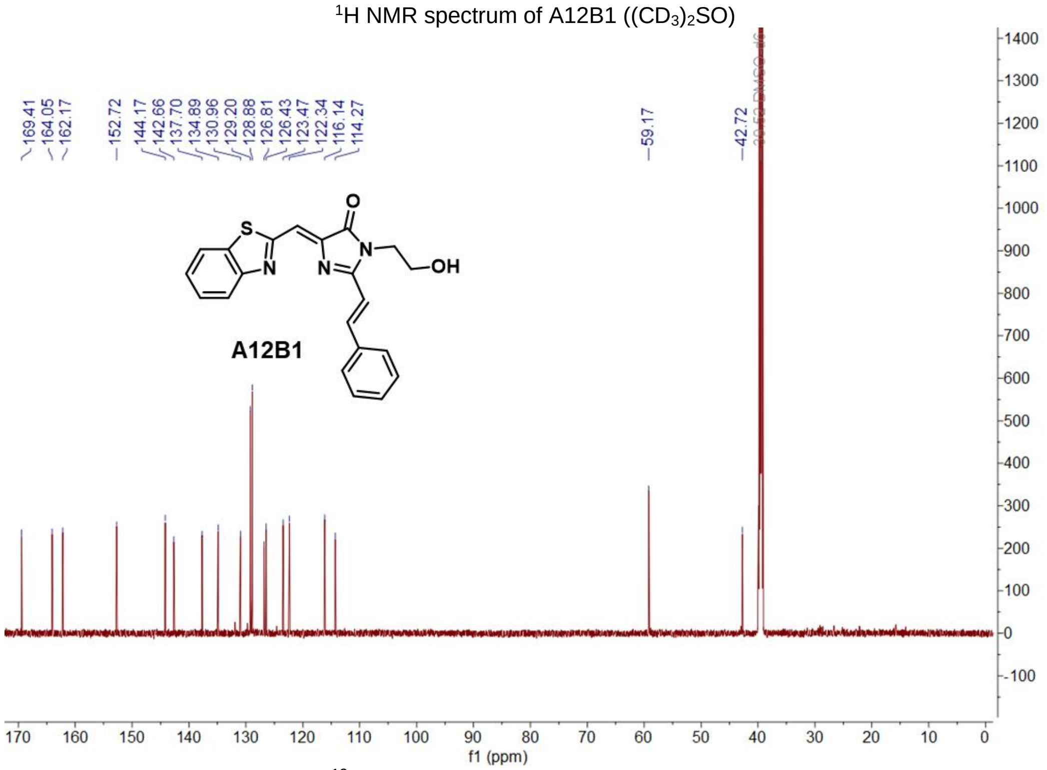

^{13}C NMR spectrum of A12B1 ($(CD_3)_2SO$)

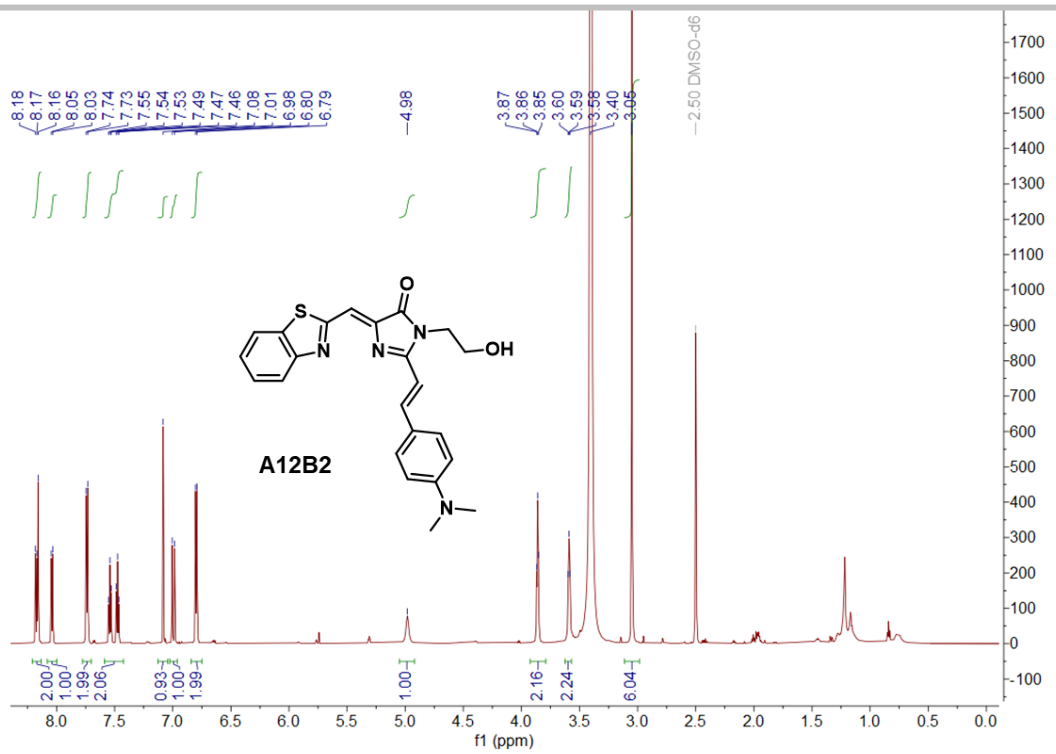

^{1}H NMR spectrum of A12B2 ($(CD_3)_2SO$)

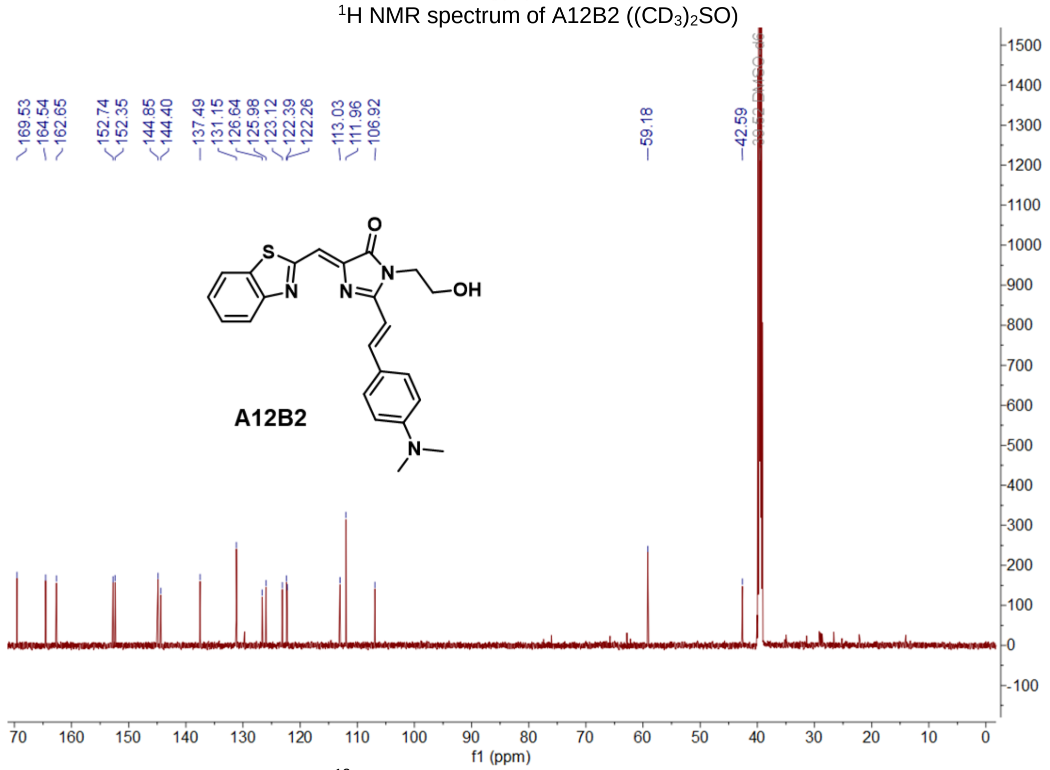

^{13}C NMR spectrum of A12B2 ($(CD_3)_2SO$)

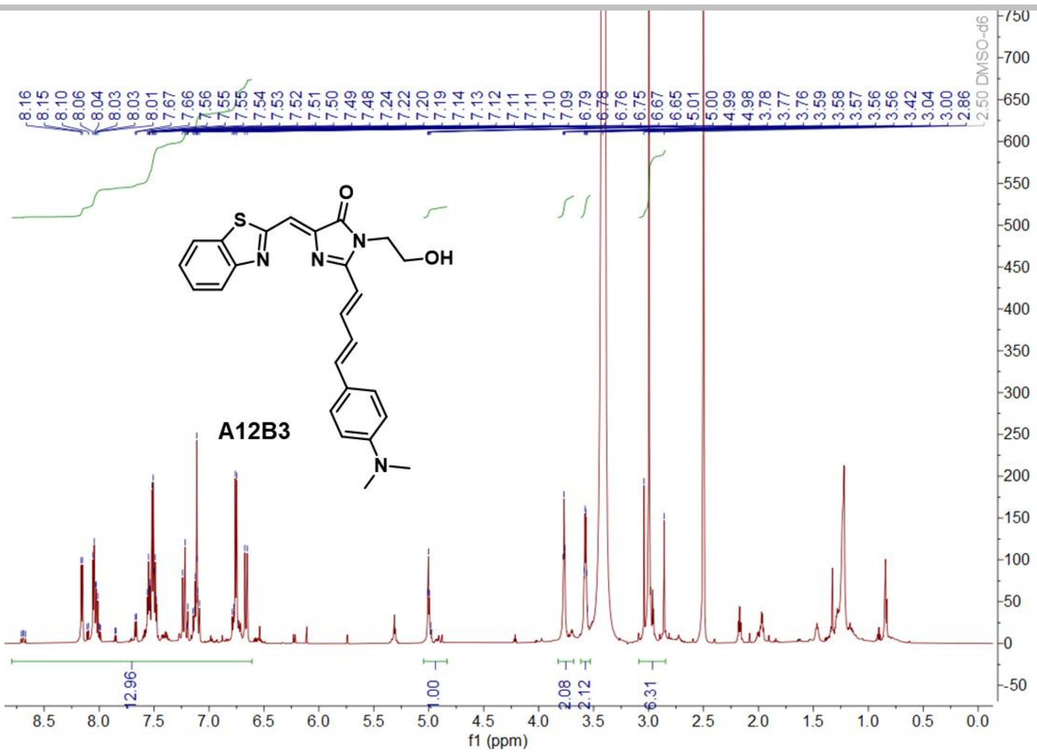

^{1}H NMR spectrum of A12B3 ($(CD_3)_2SO$)

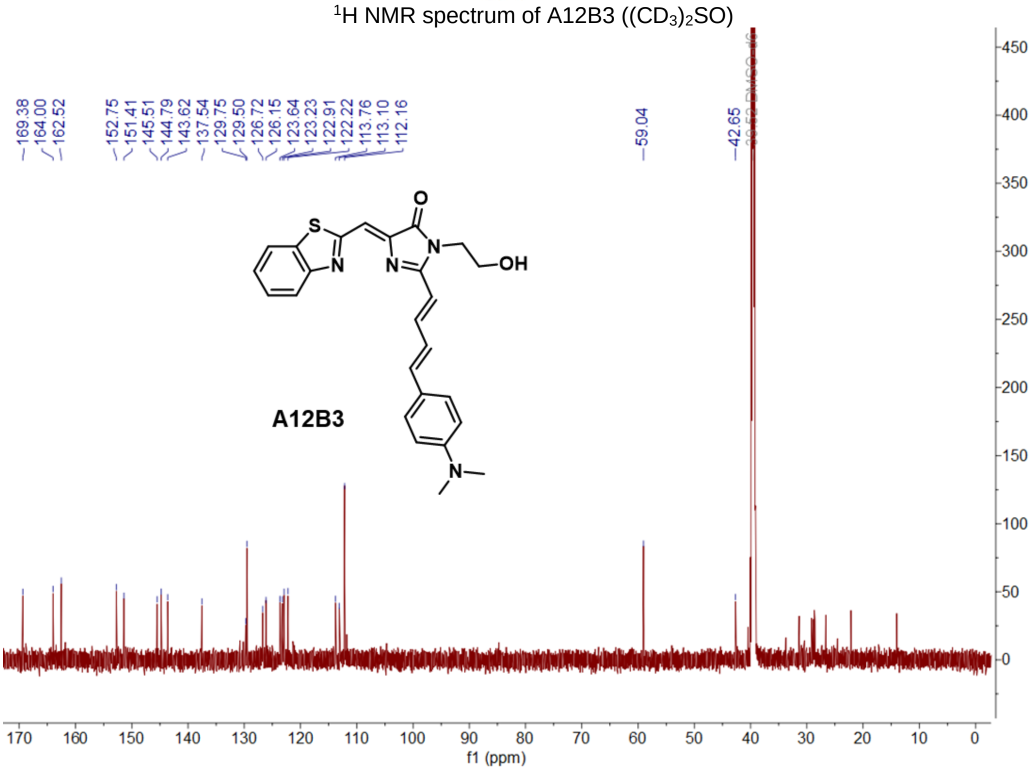

^{13}C NMR spectrum of A12B3 ($(CD_3)_2SO$)

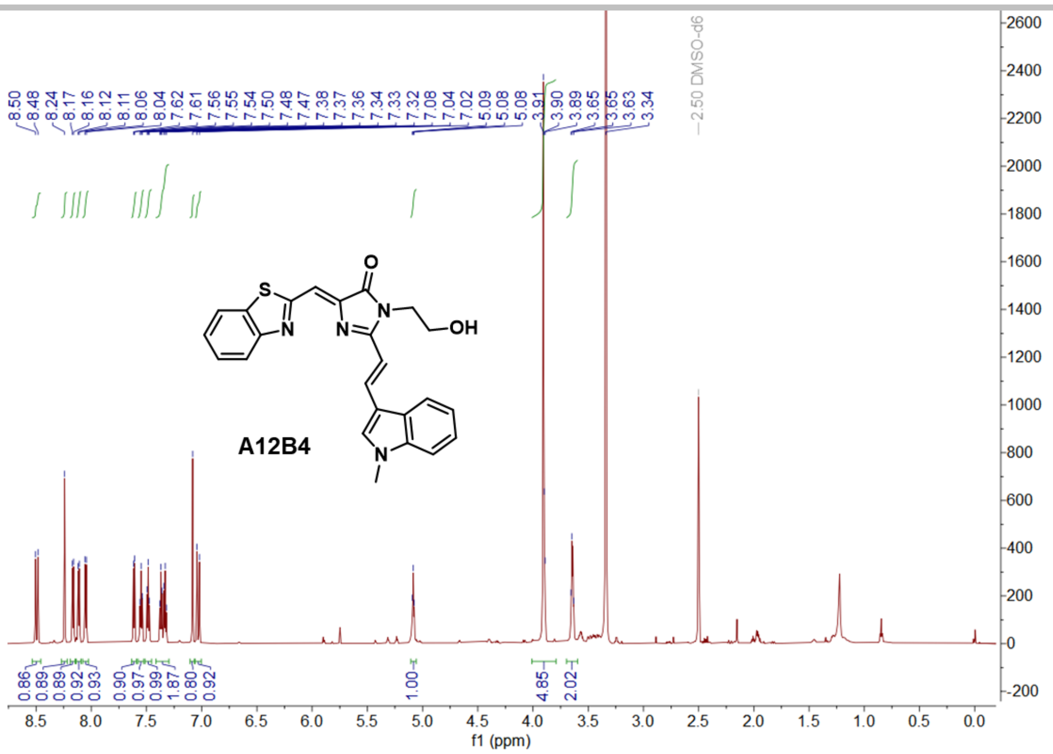

^{1}H NMR spectrum of A12B4 ($(CD_3)_2SO$)

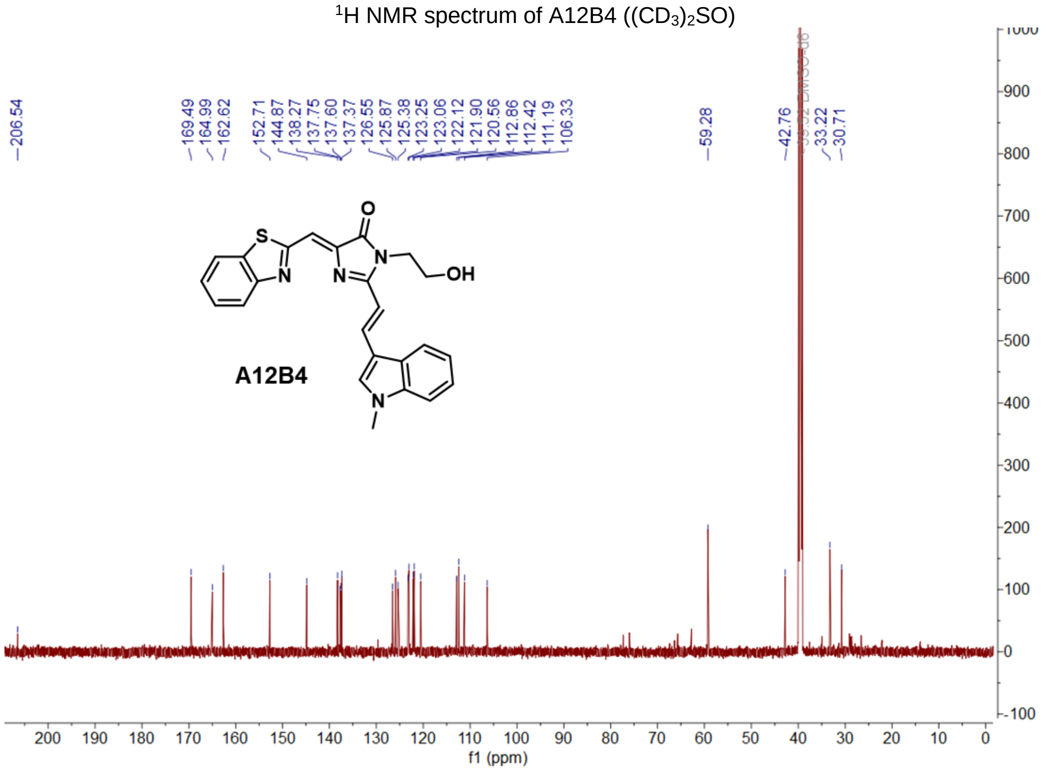

^{13}C NMR spectrum of A12B4 ($(CD_3)_2SO$)

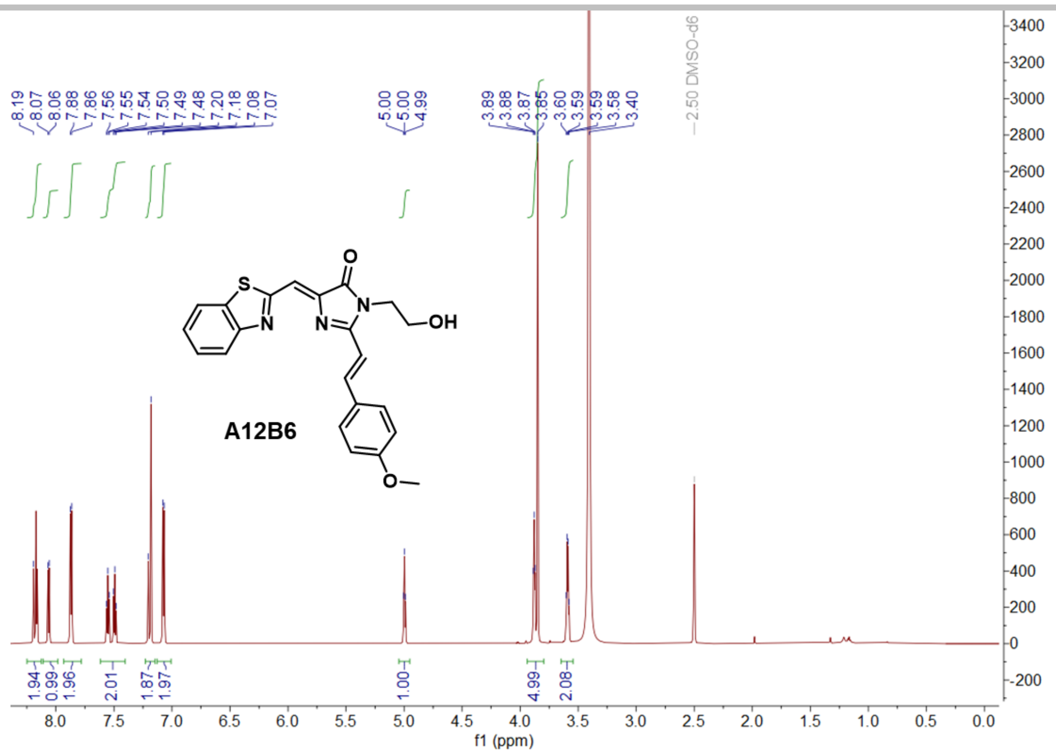

^{1}H NMR spectrum of A12B6 ($(CD_3)_2SO$)

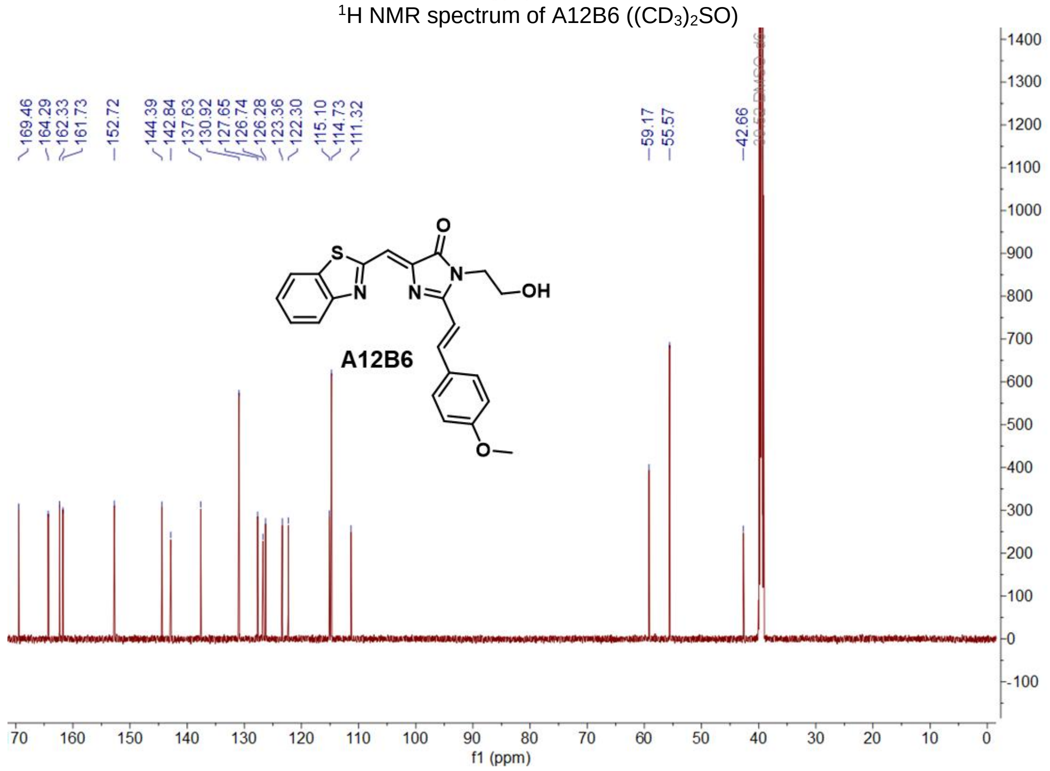

^{13}C NMR spectrum of A12B6 ($(CD_3)_2SO$)

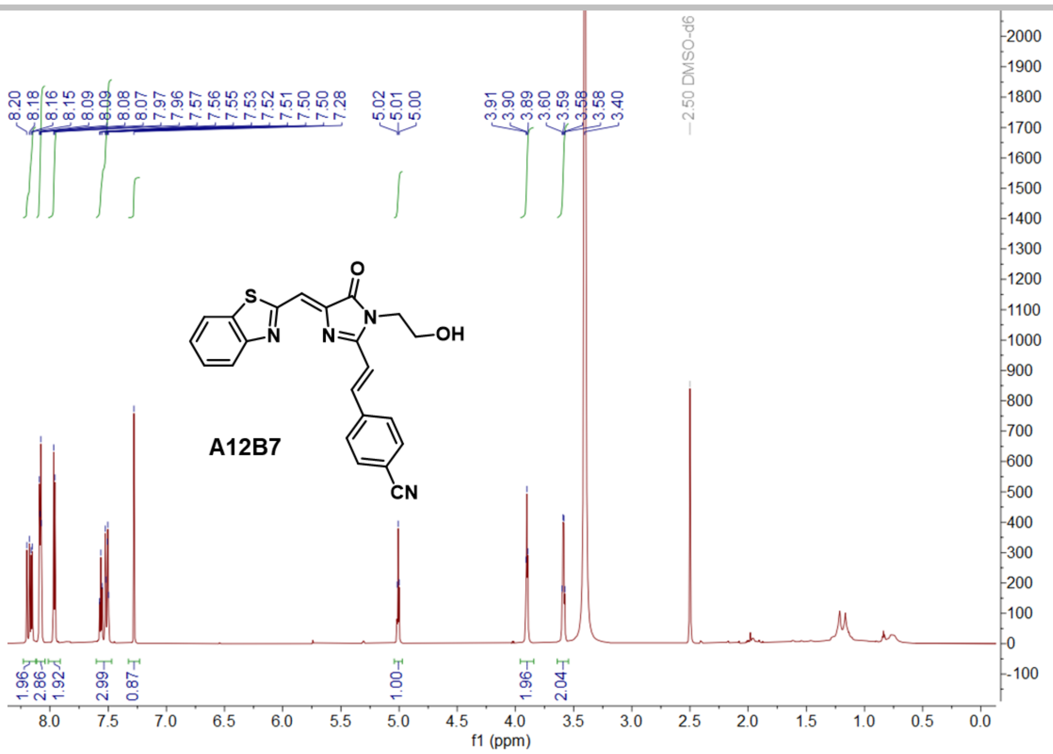

^{1}H NMR spectrum of A12B7 ($(CD_3)_2SO$)

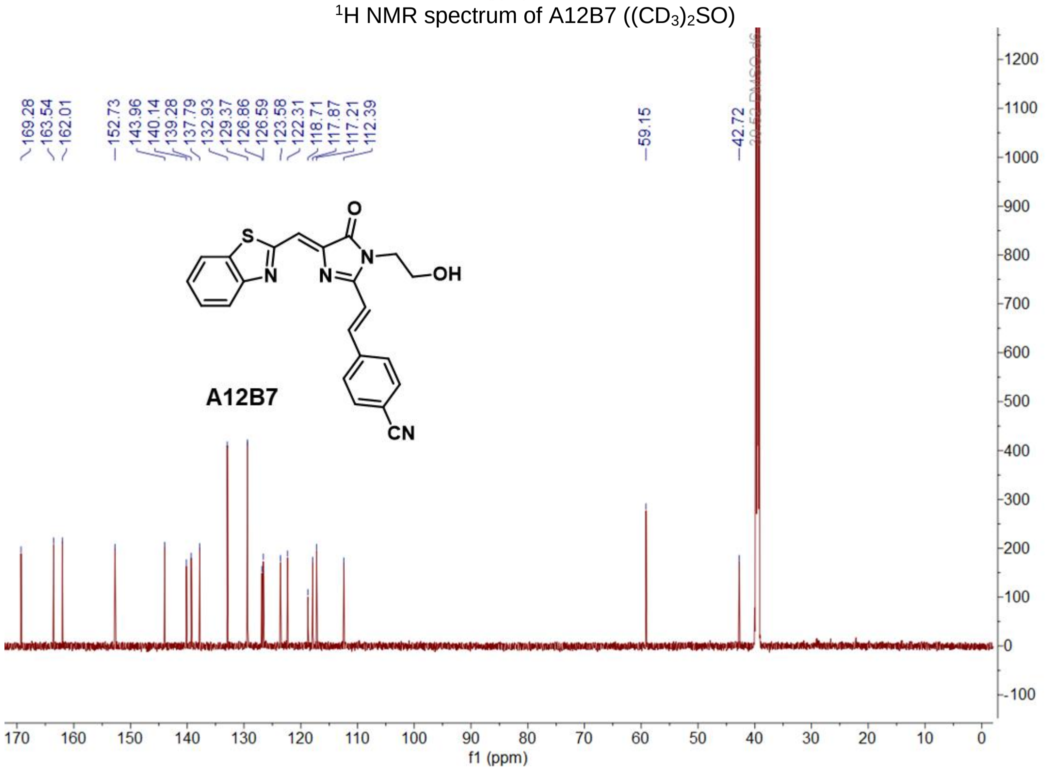

^{13}C NMR spectrum of A12B7 ($(CD_3)_2SO$)

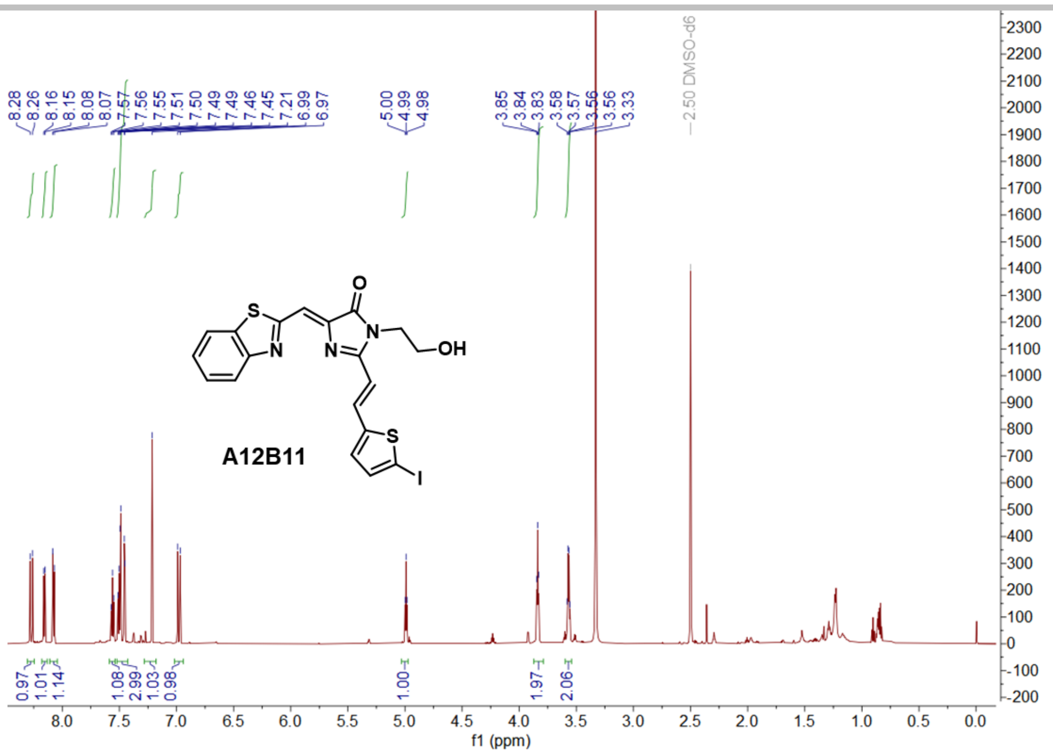

^{1}H NMR spectrum of A12B11 ($(CD_3)_2SO$)

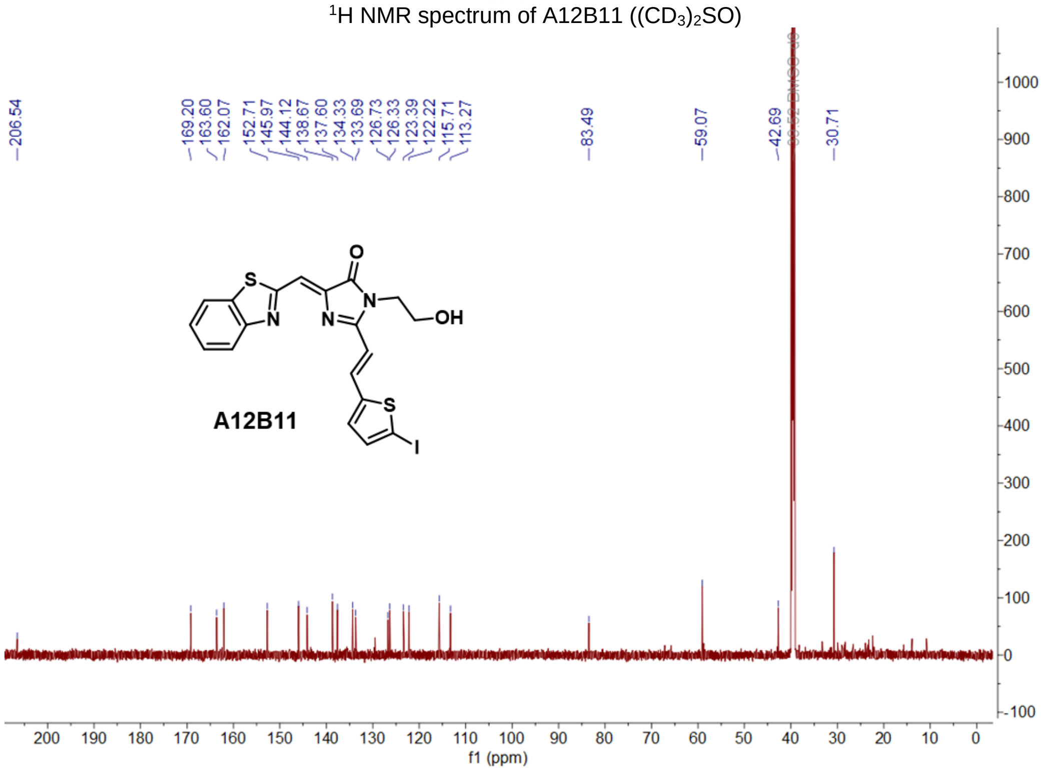

^{13}C NMR spectrum of A12B11 ($(CD_3)_2SO$)

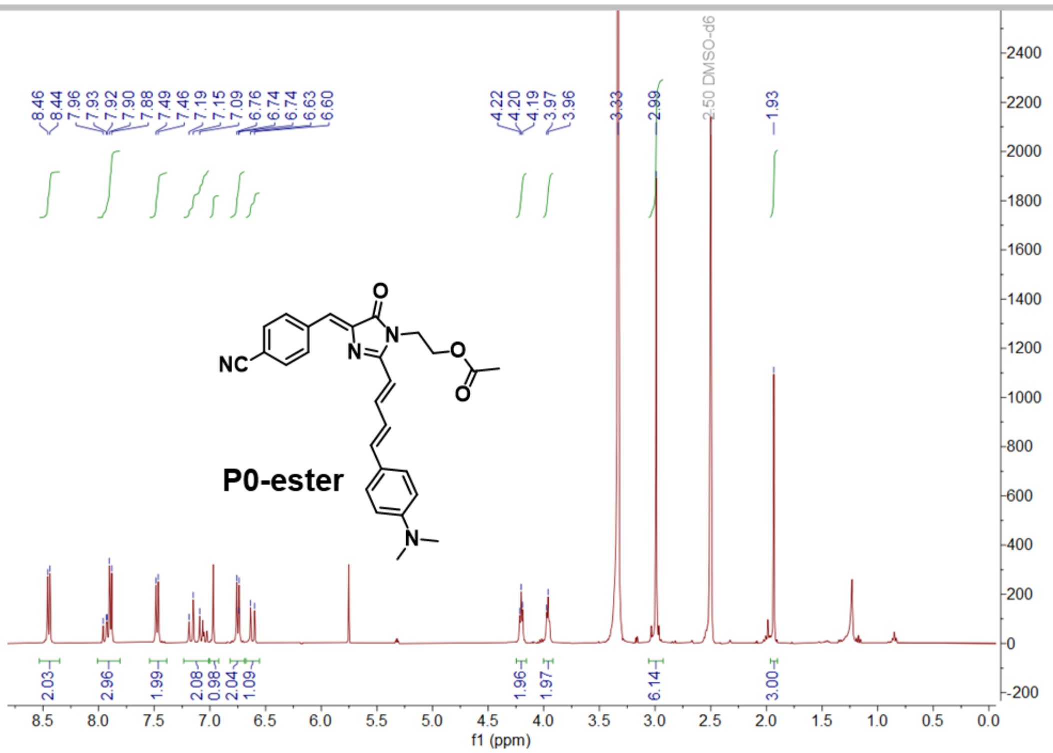

^{1}H NMR spectrum of P0-ester ($(CD_3)_2SO$)

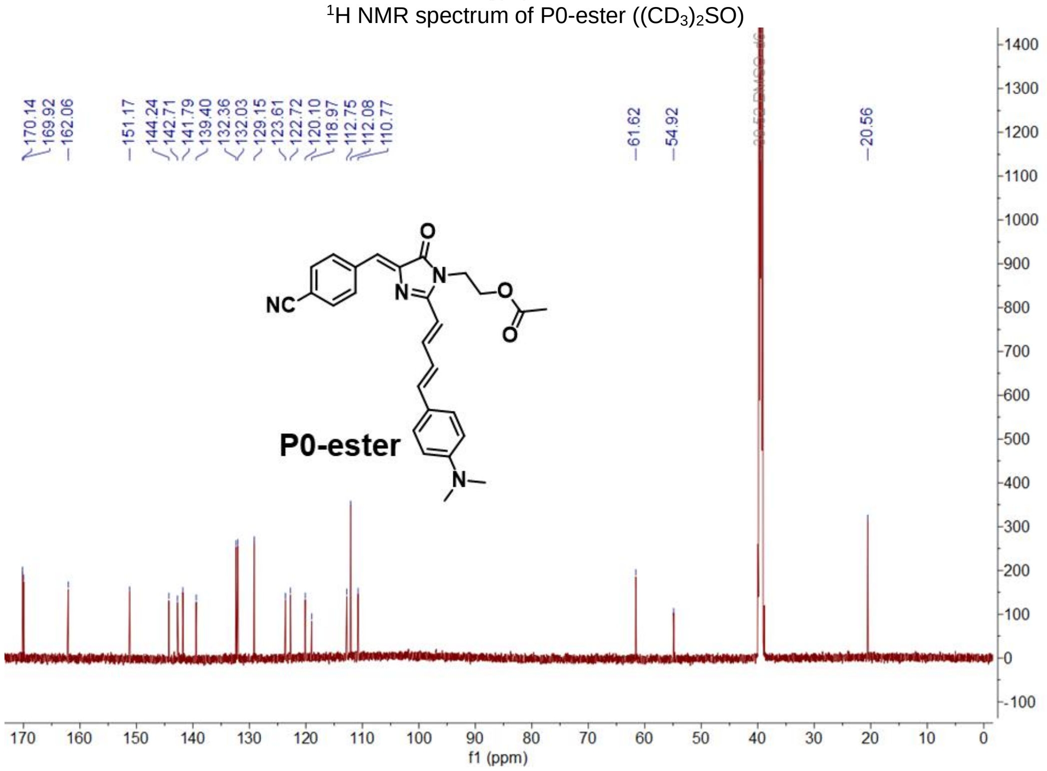

^{13}C NMR spectrum of P0-ester ($(CD_3)_2SO$)

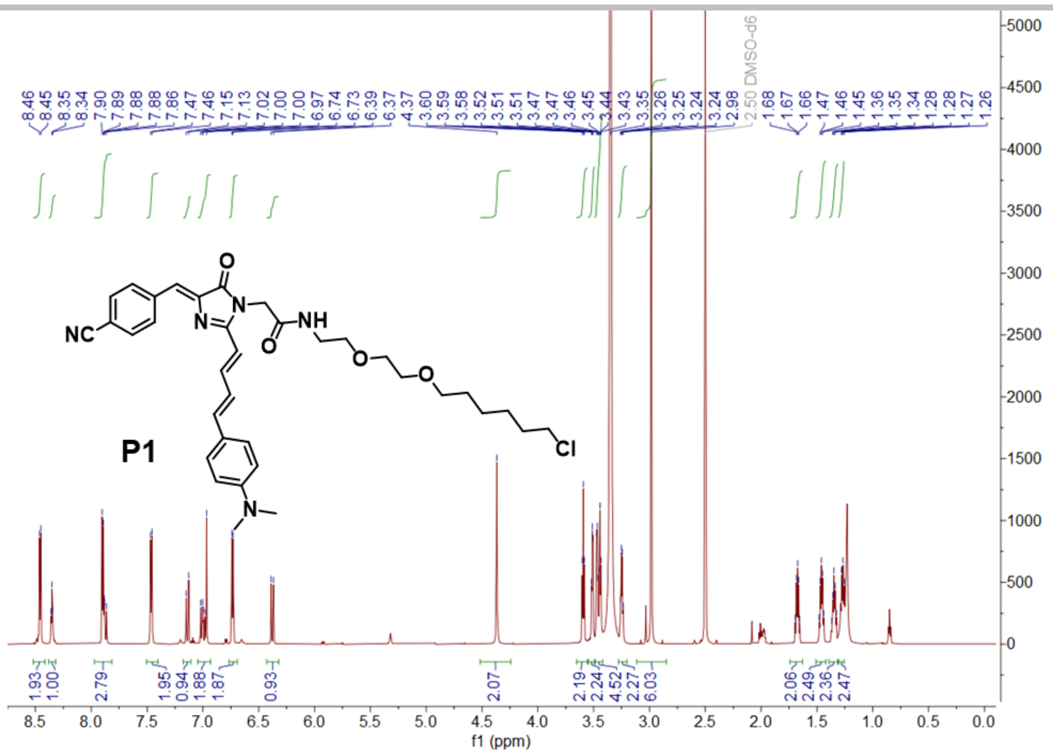

^{1}H NMR spectrum of P1 ($(CD_3)_2SO$)

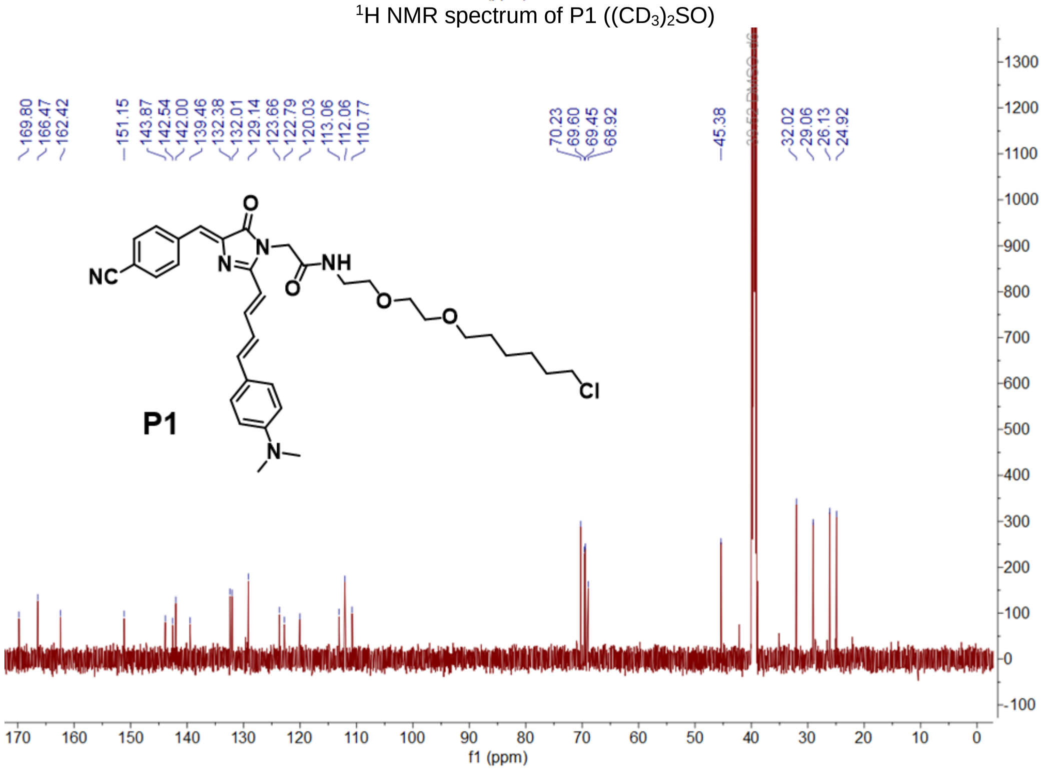

^{13}C NMR spectrum of P1 ($(CD_3)_2SO$)

6. References

[1] X. Shu, V. Lev-Ram, T. J. Deerinck, Y. Qi, E. B. Ramko, M. W. Davidson, Y. Jin, M. H. Ellisman, R. Y. Tsien, *PLOS Biology* **2011**, *9*, e1001041.

[2] N. Keller, D. Bessinger, S. Reuter, M. Calik, L. Ascherl, F. C. Hanusch, F. Auras, T. Bein, *J. Am. Chem. Soc.* **2017**, *139*, 8194-8199.

[3] A. Baldridge, J. Kowalik, L. M. Tolbert, *Synthesis* **2010**, *2010*, 2424-2436

7. Author Contributions

R. Sun performed the experiments and wrote the manuscript draft. H. Feng, W. Wan and D, Shen contributed to photochemical characterizations. Y. Huang and X. Zhang contributed to the phase separation experiments. N, Zhao, B, Zhong and Q, Zhao contributed to the proteomic analysis. Y, Zhang, L. Zhang and Y. Liu conceptualized this work and edited the manuscript.
